# blenderFaceUV: Toward Marker‐Based Precision and Flexibility in Facial Tracking With “Invisible” Markers

**DOI:** 10.1111/psyp.70394

**Published:** 2026-09-21

**Authors:** Axel Zinkernagel, Rainer W. Alexandrowicz, Dennis Küster, Manfred Schmitt, Tanja Lischetzke

**Affiliations:** ^1^ University of Wuppertal Wuppertal Germany; ^2^ RPTU University of Kaiserslautern‐Landau Landau Germany; ^3^ University of Klagenfurt Klagenfurt Austria; ^4^ University of Bremen Bremen Germany

**Keywords:** blenderFace, facial movement measurement, ultraviolet light, UV recording, video recording

## Abstract

blenderFace is a highly precise marker‐based approach for measuring facial activity. Unlike most camera‐based automated facial expression recognition (AFER) systems, which rely on fixed natural landmarks, blenderFace flexibly tracks virtually any point of interest using painted markers. However, visible markers, like EMG electrodes, may alter appearance, increase self‐awareness, and affect spontaneous responding in face‐to‐face interactions. To address these limitations, we introduce blenderFaceUV, which uses sunscreen‐based markers that are visible only under ultraviolet (UV) light (365 nm). In Study 1, we assessed UV‐marker tracking accuracy using parallel visible‐light and UV recordings from two participants. In Study 2, we examined the scalability of blenderFaceUV in *N* = 18 lay participants prompted to produce a broad range of facial movements using common emotion terms. We also compared identical facial movements measured with blenderFaceUV and OpenFace, a widely used AFER system based on a point distribution model (PDM). Study 1 showed that UV markers could be tracked with similarly high accuracy as visible markers (reprojection/solve error < 0.3 pixels). Across 10 markers and two participants, with more than 3800 measurement occasions per participant, the mean correlation between visible and UV marker movements across the x‐ and y‐coordinates was *r* = 0.957, while centered RMSE values indicated average deviations of approximately 1 mm. Study 2 demonstrated the scalability of the method and revealed PDM‐related artifacts in OpenFace measurements, particularly during eye blinks and large mouth movements. PDM‐interpolated landmarks, such as upper‐lip markers, also deviated substantially from marker‐based blenderFaceUV measurements. Practical challenges remain, including the complexity of applying invisible markers and the relatively high cost of UV cameras (≈$2000–$6000). We discuss technical requirements, limitations, and a recommended workflow for implementing blenderFaceUV.

## Impact Statement

BlenderfaceUV is a new approach for measuring facial activity with invisible facial markers. Compared to markerless camera‐based measures, it provides exceptionally precise geometrically based measurements while avoiding many of the social awareness effects that constrain experimental psychophysiological research relying on either facial electromyography or marker‐based methods. We present the results of two methodological studies employing BlenderfaceUV and discuss how it could become a valuable new addition to the toolkit of psychophysiological laboratories.

blenderFace (Zinkernagel et al. 2019) is a marker‐based toolkit^1^ for precise facial movement and activity detection, a task that is most frequently performed via electromyography (EMG, Küster et al. 2025; Liu et al. 2026), using markerless automatic facial expression recognition (AFER) algorithms (e.g., OpenFace Baltrušaitis et al. 2016; Noldus Facereader), or expert ratings based on visible facial muscle movements as defined by the facial action coding system (FACS, Ekman et al. 2002).

Measurement of facial muscle activity has played a central role in affective science, psychophysiology, and social neuroscience because facial responses provide important insights into emotional, motivational, and interpersonal processes. Facial EMG has been used extensively to investigate affective evaluation and emotional valence, with a large body of work demonstrating systematic relationships between facial muscle activity and the hedonic qualities of emotional stimuli (e.g., Cacioppo et al. 1986; Lang et al. 1993; Larsen et al. 2003). Importantly, facial behavior is not merely redundant with other indicators of emotion. Contemporary theories conceptualize emotions as multicomponent processes comprising experiential, physiological, expressive, motivational, and cognitive components (Scherer 2005), while empirical work suggests that the concordance among these response systems is often partial rather than complete (e.g., Hollenstein and Lanteigne 2014; Mauss et al. 2005; Michalska and Díaz 2025). Facial behavior therefore represents a distinct and theoretically informative component of emotional and social functioning.

Facial measures have been employed to study implicit attitudes and social cognition (Vanman et al. 1997; Vanman et al. 2004), as well as facial mimicry, emotion communication, and interpersonal coordination during social interaction (see, e.g., Fridlund 1991, 1994; Hess and Fischer 2013, 2014; Kappas 2013). More broadly, contemporary accounts increasingly view facial behavior as one component of a wider system of nonverbal emotional communication that includes facial, vocal, bodily, and other expressive signals involved in social coordination and interpersonal understanding (e.g., Sauter 2017). At the same time, research has increasingly emphasized the importance of dynamic facial behavior for social perception and interaction (e.g., Krumhuber et al. 2013, 2023), while advances in machine learning and affective computing have stimulated a growing interest in understanding facial expressions in naturalistic and context‐rich environments (Bian et al. 2024). Collectively, this body of work has contributed to theoretical debates on the relationship between emotion and expression, the social functions of emotional behavior, and the mechanisms through which emotions are communicated and regulated in interpersonal contexts. Together, these lines of research highlight the need for a broad methodological toolbox that can capture facial behavior across diverse research contexts, including socially meaningful and increasingly naturalistic settings. More generally, the development of visually unobtrusive measurement approaches is consistent with a longstanding interest in minimizing reactivity in behavioral and psychophysiological research (Webb et al. 1966). This issue may be particularly relevant in facial‐expression research because facial behavior is known to be influenced by social context and the physical or implied presence of an audience (Fridlund 1991). Accordingly, a central motivation of the present work was to expand the methodological toolbox by implementing marker‐based facial tracking in a visually unobtrusive manner while retaining the methodological advantages of direct marker tracking.

Facial electromyography (EMG) has been considered the gold standard for laboratory research for years (Fridlund and Cacioppo 1986; Veldanda et al. 2024) due to its superior temporal resolution and sensitivity to subtle expressions (van Boxtel 2010; Wingenbach et al. 2024). blenderFace allows simultaneous recording of facial movements across the entire face with relatively little additional effort when increasing the number of measurement sites. Perhaps more importantly, facial EMG electrodes can only be placed in a limited number of locations on the skin, and the interpretation of EMG signals is complicated by the issue of crosstalk, i.e., the detection of electrical activity that did not originate from the muscle closest to where the recording electrodes are placed (Tassinary et al. 2007; van Boxtel and Jessurun 1993; van Boxtel et al. 1998). Although crosstalk can, to some extent, be addressed through careful experimental design or machine recognition to identify muscle‐specific activation patterns (Küster et al. 2025), complex movement analysis across the face is typically still beyond the scope of EMG research. In addition, facial EMG recordings are dependent on maintaining stable contact between the electrode and the skin, and methodological guidelines have long emphasized the importance of electrode placement, impedance, and movement‐related artifacts for signal quality (Fridlund and Cacioppo 1986; Tassinary et al. 2007). Facial EMG also differs from most other EMG recording sites because facial muscles are relatively small and close together, while the facial surface is highly deformable and changes substantially as wrinkles form during facial expressions (Zhao et al. 2021). Therefore, signal quality may be affected by temporal changes in electrode‐skin impedance during longer recordings (Hewson et al. 2001). Consistent with these challenges, recent work has sought to develop more conforming electrode designs for facial electrophysiological monitoring (Zhao et al. 2021). Furthermore, recent work on facial EMG‐based action‐unit recognition suggests that recognition performance may improve when larger and more adhesive electrodes are used during repeated production of subtle and maximum‐intensity facial expressions (Küster et al. 2026). For optimal recording conditions, EMG also requires high‐quality amplifiers and an electromagnetically shielded laboratory, making this method less accessible than camera‐based approaches. Finally, facial EMG requires both electrodes and cables to be attached directly to participants' faces. This renders facial EMG inherently obtrusive, which increases self‐awareness and reactivity, in particular in face‐to‐face settings where electrodes are visible (Fridlund and Cacioppo 1986; Kappas 2013; Tassinary et al. 2007). In contrast, painted markers may be less visually salient than conventional facial EMG montages and may fade from attention after a relatively short period of time (Zinkernagel et al. 2019). Moreover, the points used in blenderFace can be placed considerably closer to each other, which allows for much higher resolution if required.^2^

Compared with markerless AFER approaches, blenderFace is not restricted to the detection of natural facial landmarks, such as the corners of the mouth. Instead, painted facial markers can be used to include additional points of interest with little effort.

Moreover, these markers substantially increase measurement precision, allowing submillimeter accuracy (Zinkernagel et al. 2019), whereas AFER algorithms must interpolate movements in facial regions without natural landmarks.

Lastly, compared to expert FACS coding, which can take hours for a minute of video data (Bartlett et al. 2006), blenderFace offers a substantially more efficient means of measuring fine‐grained facial activity. Marker movements provide an objective measurement of physical movements without requiring intensive training to eliminate potential rating biases. In addition, blenderFace, like markerless AFER algorithms, provides a continuous measurement in mm for each location, while expert FACS coders rate intensity using a five‐level ordinal scale for each Action Unit (AU), which reflects the activation of underlying facial muscles and, at least partly, the direction of movement.

Despite these advantages, blenderFace still faces limitations. Although the standard blenderFace method may be somewhat less obtrusive than conventional facial EMG montages and substantially less obtrusive than high‐density EMG setups (cf. Cui et al. 2021; Guntinas‐Lichius et al. 2023), its use in dyadic settings remains challenging. In social contexts, visible facial recording apparatus may heighten self‐awareness (Wicklund 1975) and thereby alter spontaneous expressive behavior (Kappas et al. 2013; Küster 2020). Consistent with social‐functional accounts of facial behavior (e.g., Fridlund 1994; Hess and Fischer 2013), facial expressions are highly sensitive to social context. For example, smiling toward pleasant stimuli can be intensified by the presence of others (Philipp et al. 2012), an effect that has been shown to hold even for implicit audiences, where participants are objectively alone (Fridlund 1991). Thus, painted facial markers are still likely to be distracting, “funny”, or awkward in natural dyadic or other social interactions. In addition, painted markers or EMG electrodes worn by an interaction partner could potentially amplify the salience and recognizability of otherwise subtle expressions, which could lead to unintended social resonance effects (Kappas and Descôteaux 2003). A further methodological limitation is that visible markers may also limit an objective comparison to AFER algorithms. As AFER algorithms have generally not been trained to detect and ignore these markers, painted markers could impede pattern or landmark detection performance (Evtimov et al. 2017; Sharif et al. 2016), which limits the external validity of simultaneous performance comparisons between marker‐based methods and AFER algorithms.

To address these limitations, the present work introduces blenderFaceUV, a new variant of the blenderFace method. blenderFaceUV uses “invisible”, visually unobtrusive sunscreen markers and an enhanced recording setup for the ultraviolet (UV) range of light (320–380 nm). Our first study (*N* = 2) aimed at providing an initial validation of this method by comparing the tracking performance of blenderFaceUV markers with the original visible blenderFace markers. To this end, we employed both a regular webcam to record the human‐visual range of light (VIS, ≈380–700 nm) to verify that the sunscreen markers are not noticeable in this spectrum, and a UV camera to track the UV markers and compare their performance. Subsequently, we compared the measurement of visible and sunscreen markers directly to assess the extent to which these two measurements agree. In a second study (*N* = 18), we (a) tested the scalability of blenderFaceUV with only UV markers, (b) compared the measurement results with those obtained previously using visible black markers (see Zinkernagel et al. 2019), and (c) examined the agreement between marker movements measured with blenderFaceUV and those estimated by OpenFace (Baltrušaitis et al. 2016), which uses a point distribution model (PDM). Data, R scripts, and additional plots of the present studies are available on the OSF Supplement (https://doi.org/10.17605/OSF.IO/XTJBG). In addition, an updated version of the “Stepwise Instructions for the blenderFace method” (ver. 1.6 for Blender 2.80 and later) is provided along with training videos, training data, and training Blender files in a separate OSF repository (https://doi.org/10.17605/OSF.IO/QYVWH).

## 1 Study 1

### 1.1 Method

Two female participants from the University of Wuppertal, Germany, were recruited to simultaneously record facial movements with visible and sunscreen markers using a Logitech C920 webcam (≈380–700 nm visual‐light range) and a UV‐sensitive camera (XNiteUSB8M‐MNCUVL camera with a Llewellyn Optics fused silica lens, transparent to UV light (320–380 nm), Llewellyn Data Processing, LDP LLC; https://maxmax.com).

**FIGURE 1:**
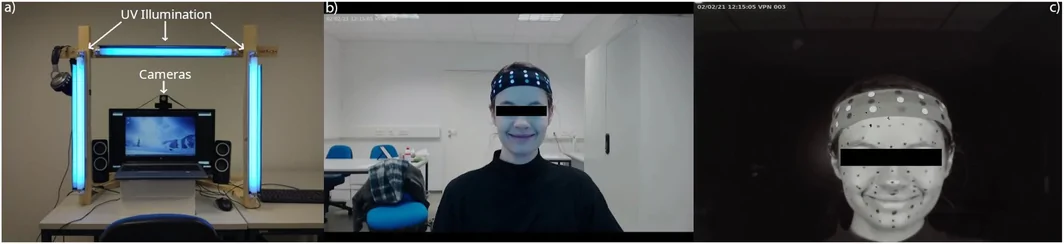
(a) Setup of Study 2 (similar to Study 1) with both cameras and UV light source. (b) Screenshot of the camera recording in the visible range of light showing no markers. (c) Screenshot of the UV camera recording showing all facial sunscreen markers.

Both cameras were positioned so that they recorded the participant from almost the same angle. More specifically, the cameras were stacked on top of each other and attached to the center of the upper frame of the screen (see Figure 1a). The two cameras were positioned approximately 2 cm apart, corresponding to a viewing‐angle difference of about 2.29° for an object located 50 cm from the cameras. As a light source for the visible range of light, we used ceiling‐mounted neon lighting, while UV illumination was provided by two tripod‐mounted UV LED floodlights (Type CHX‐FL‐A‐100 W with a radiation peak at 370 nm) positioned 2.5 m from the participant at *±*30° relative to the participant's frontal axis, allowing for shadow‐free illumination of the participant's face in both ranges of light.

**FIGURE 2:**
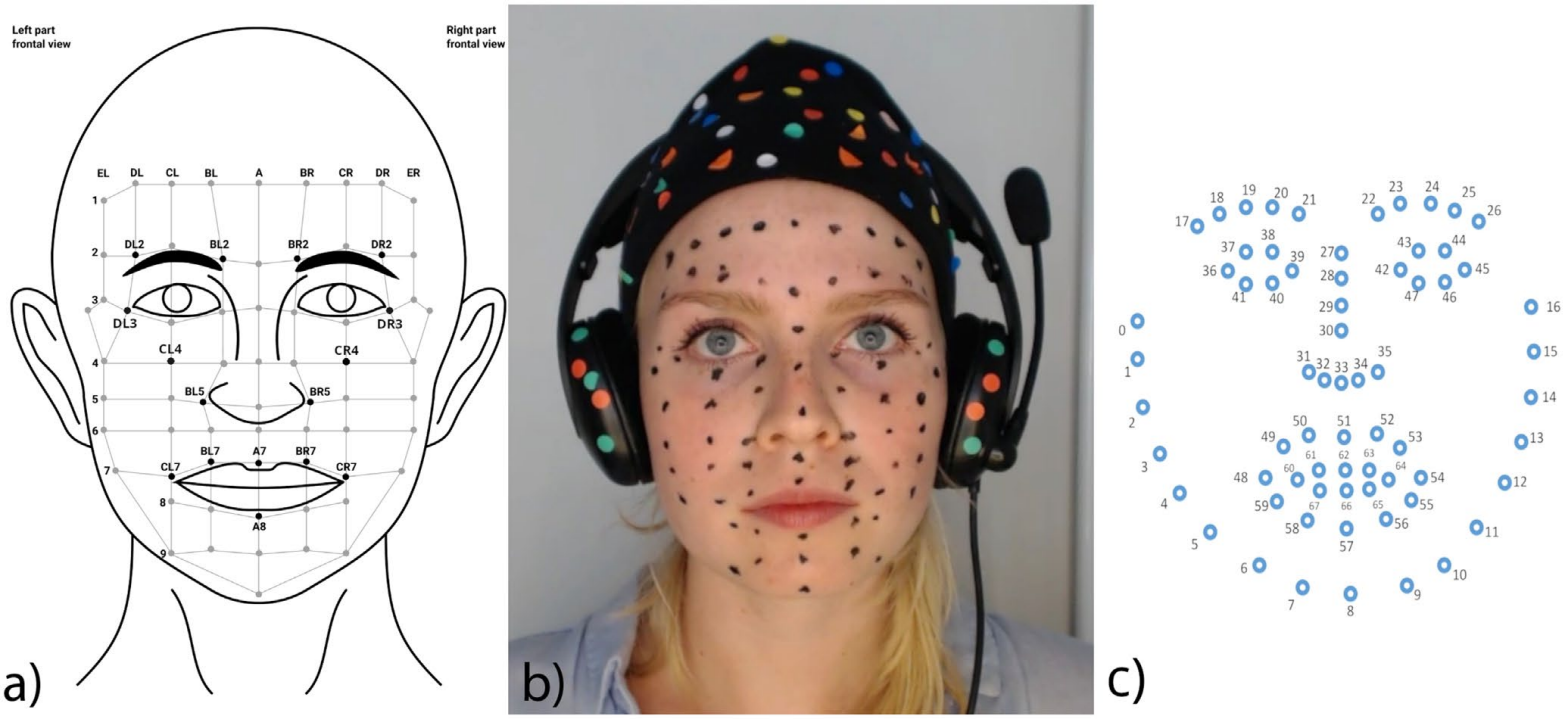
(a) Marker labels of the blenderFace toolkit. (b) Participant setup for the blenderFace toolkit: The participant was equipped with headband and headphones with colored adhesive markers, and black fluid eyeliner markers painted on the skin of the face. For UV recordings, sunscreen was used instead of eyeliner. (c) Markers of the point distribution model of OpenFace.

We ensured that the participants had no allergy to sunscreens or heightened sensitivity to UV light, and that the overall UV exposure did not exceed 15 min.^3^ First, following the blenderFace procedure (Zinkernagel et al. 2019), we applied the 68 visible markers with black liquid eyeliner to the participant's face (see Figure 2b). Subsequently, we applied additional UV markers at positions BL2, BR2, DL2, DR2, BL5, BR5, CL7, CR7, A7, and A8 (see Figure 2a) with a cotton swab and sunscreen (NIVEA Derma Skin Clear UV Fluid SPF 50+; non‐water‐resistant). We placed the UV markers with minimal offset (< 2 mm) directly next to the visible markers. The slight offset was required to prevent any potential confusion between marker types because the visible markers are also visible in the UV range.

After applying the visible and the UV markers, the participants' task was to imitate specific Action Unit (AU) movements. To support participants in performing this task, the experimenter provided verbal instructions and presented a short video clip for each AU from the MPI video database (Kleiner et al. 2004), which offers FACS‐validated AU portrayals^4^ (Ekman et al. 2002). The participants then imitated the AUs 1, 2, 4, 9, 12, 17, 20, and 24 three times each to generate facial movements. These AUs were selected because they provide good coverage of the face while still being relatively easy to produce for lay participants (Küster et al. 2025). In addition, our objective was to generate broader and less constrained facial movements to examine the robustness of the marker movements. To this end, the participants were prompted with a set of discrete lay emotion terms (happiness, sadness, fear, disgust, and anger) without any additional guiding stimulus materials to encourage a more naturalistic but instructed facial expression. The overall video recording procedure took approximately 3 min per participant.

The analysis of the webcam and UV videos followed four steps: First, since the cameras had different resolutions, the videos were rescaled to the same head size, superimposed, and controlled for exact temporal and spatial alignment by modifying the opacity of the superimposed video in the video editing software (Blender, ver. 4.5.3 LTS, https://www.blender.org). We then tracked the 68 visible markers of the webcam recording for a short episode of the participant showing no facial expression and estimated the facial surface (cf. Zinkernagel et al. 2019) of the participants. Third, we tracked both the visual and UV markers at the positions BL2, BR2, DL2, DR2, BL5, BR5, CL7, CR7, A7, and A8 in the corresponding video for each AU and emotion term prompt episodes.

Finally, the data of the visible‐ and UV marker movements were exported to a CSV file per participant for statistical analysis.

### 1.2 Analysis and Results

**FIGURE 3:**
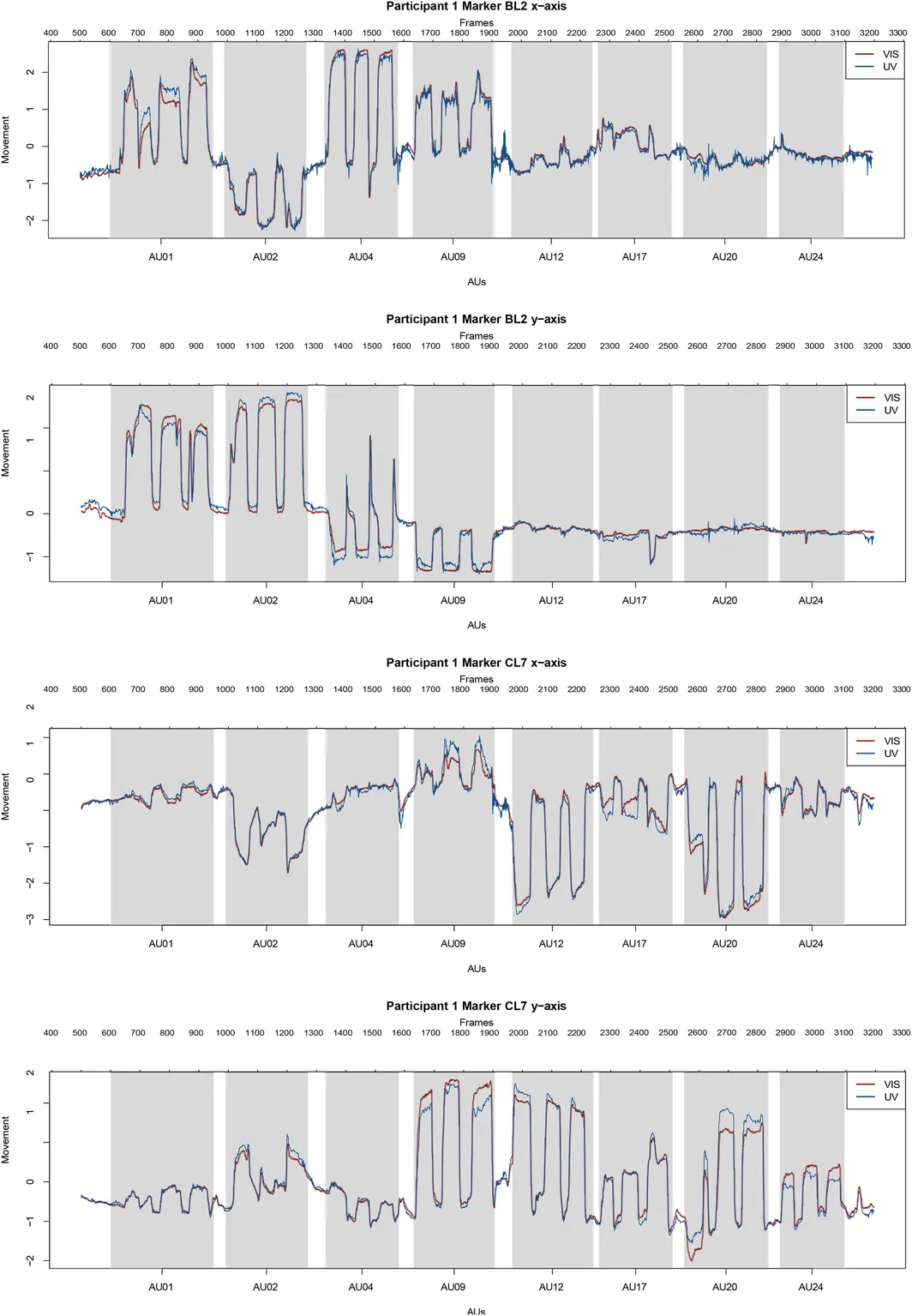
Movements of marker BL2 (left inner eyebrow) and marker CL7 (left mouth corner) along the x‐ and y‐axes for Participant 1, shown from top to bottom. The lower x‐axis and shaded gray intervals of each plot show the respective AUs (three repetitions). The upper x‐axis of each plot shows the frames of the video. The y‐axis of each plot represents the scaled deviation from zero. The red line of each plot represents the movement of the corresponding axis of the visible marker, and the blue line indicates movement of the UV marker.

First, we checked the movement data for measurement artifacts (e.g., markers dropping off the facial surface causing implausible 3D coordinates) and outliers (e.g., movements that are faster than humans are capable of) with functions from the blenderFace R package.^5^ Subsequently, we reduced the data by omitting the z‐axis, as it plays a minor role in facial movements (Zinkernagel et al. 2019). In a next step, we plotted the trajectories of the visual and UV marker movements separately for each participant, marker, and axis for visual inspection (e.g., visual BL2_x and UV BL2_x; visual BL2_y and UV BL2_y of Participant 1). Example plots for the x‐ and y‐axis trajectories of markers BL2 and CL7 during AU imitation are presented in Figure 3. A full set of plots, including expression prompts for both participants, is available in the Data S1 on OSF (https://doi.org/10.17605/OSF.IO/XTJBG).

From a measurement perspective, the visible‐light and UV trajectories should be identical because both capture the physical movement of markers on the same individuals during the same time intervals, albeit in different wavelength ranges. As illustrated in Figure 3, the trajectories are not perfectly identical, and explanations for these discrepancies are considered in the Discussion section. Although accurate AU performance was not the focus of this study and the participants had no prior FACS training, the observed marker movements were largely consistent with the expected facial actions.^6^ For example, the left inner eyebrow marker (BL2) moves upwards (positive movement along the y‐axis) and inwards to the nose (positive movement along the x‐axis^7^) when Participant 1 was instructed to display AU1 (inner brow raiser) three times. In contrast, the marker BL2 moves downward and inward when the participant was instructed to pose AU4 (brow lowerer; negative movement along the y‐axis, positive movement along the x‐axis).

To get an overall measure of the correspondence, we computed the correlations of the visible‐ and UV marker movements per participant, marker, and axis, including the AU and expression prompt episodes. These analyses included 3860 measurement occasions (frames) for Participant 1, and 4665 measurement occasions (frames) for Participant 2, respectively. Correlations between visible and UV marker movements per participant, marker, and axis are presented in Table 1. To aggregate these correlations, we applied Fisher's *z* transformation, averaged the resulting values, and back‐transformed the mean to the correlation scale. Across all axes, markers, AU episodes, and expression‐prompt episodes, the mean correlation was *r* = 0.957. Because these correlations are based on highly dependent observations, we additionally computed bootstrapped correlations for comparison with those reported in Table 1 (*n* = 25, 5000 bootstrap samples). These results are provided in the Data S1. The bootstrapped correlations closely matched the original estimates, with a maximum deviation of 0.02.

**TABLE 1:** Correlations of the visual and *UV* marker movement measurement per participant, marker, and axis.

Marker	Participant 1		Participant 2	
	x‐axis	y‐axis	x‐axis	y‐axis
BL2	0.99	0.98	0.89	0.97
BR2	0.98	0.98	0.86	0.98
DL2	0.99	0.88	0.97	0.84
DR2	0.99	0.93	0.85	0.93
BL5	0.97	0.98	0.92	0.97
BR5	0.97	0.98	0.93	0.99
CL7	0.98	0.97	0.96	0.86
CR7	0.96	0.97	0.84	0.97
A7	0.68	0.94	0.89	0.98
A8	0.91	0.96	0.85	0.98

*Note:* Correlations for Participant 1 were based on 3860 measurement occasions (frames), whereas those for Participant 2 were based on 4665 measurement occasions.

To obtain an absolute measure of alignment, we converted the marker movements of both participants to millimeters and calculated the root mean squared error (RMSE).

Because the RMSE is affected by the spatial offset between the visual and UV markers, we additionally centered the marker coordinates along the x‐ and y‐axes and calculated the RMSE based on these centered values. Table 2 presents the RMSE for the uncentered and the centered data.

**TABLE 2:** Root mean squared error (RMSE) between visual and UV marker movements for uncentered and centered coordinates.

Marker	RMSE (uncentered data)				RMSE (centered data)			
	Participant 1		Participant 2		Participant 1		Participant 2	
	x‐axis	y‐axis	x‐axis	y‐axis	x‐axis	y‐axis	x‐axis	y‐axis
BL2	2.85	1.38	1.65	3.13	0.23	0.66	0.31	0.64
BR2	2.90	2.79	0.67	4.17	0.26	0.61	0.43	0.52
DL2	2.06	2.10	3.12	2.62	0.42	0.97	0.51	0.98
DR2	1.97	1.27	1.76	7.04	0.43	1.15	0.81	0.57
BL5	0.54	0.64	2.26	0.91	0.38	0.60	0.63	0.91
BR5	1.06	1.14	1.40	1.44	0.42	0.92	0.47	0.72
CL7	3.07	5.56	6.29	6.13	0.52	1.25	0.65	2.07
CR7	1.52	2.74	5.71	3.38	0.67	1.28	1.21	1.07
A7	1.83	1.31	0.83	2.23	0.93	1.21	0.57	0.79
A8	2.19	1.38	0.96	0.91	0.54	1.18	0.83	0.89

*Note:* RMSE values are reported in mm. Uncentered RMSE values reflect both differences in marker movement and spatial offsets between the visual and UV markers. For the centered RMSE values, marker coordinates were centered separately along the x‐ and y‐axes prior to RMSE calculation, thereby removing constant spatial offsets.

### 1.3 Discussion of Study 1

In this first study, we demonstrated that the measurement of visible and “invisible” UV markers resulted in a very high average correlation. The RMSE analysis further supported the overall correspondence between visual and UV marker movements. RMSE values based on the uncentered coordinates were comparatively large and varied considerably across markers and axes, indicating that absolute spatial offsets between the two marker types contributed substantially to the observed discrepancies. After centering the marker coordinates, however, RMSE values decreased markedly for both participants and across nearly all markers. This suggests that, once constant positional offsets were removed, the trajectories recorded by the visual and UV markers showed a substantially closer correspondence. Most centered RMSE values were around or below 1 mm, although somewhat larger deviations remained for individual markers and axes. Taken together, these findings indicate that differences between the two marker types primarily concerned their absolute spatial position rather than the relative pattern of marker movement.

However, some small differences attributable to technical aspects of the setup remained between the measurements: (a) The two cameras recorded the participant from slightly different perspectives. Although we aimed to have the exact same perspective and stacked the cameras on top of each other, it is technically impossible to achieve the exact same perspective with two cameras. (b) The visible and UV markers were slightly offset on the facial skin. Even though the offset was small, the adjacent skin regions may move slightly differently, e.g., at the mouth corners. This becomes evident in the case of Participant 2, who showed stronger expression‐related facial deformations, which may explain why the overall correlations were slightly lower for this participant. For example, the visible markers were placed at the corners of the mouth, whereas the UV markers were positioned adjacent to them, farther from the vertical midline. When posing AU12, Participant 2 showed a smile‐related skin fold and the UV marker was on a raised cheek area and showed a slightly different trajectory compared to the visual marker at the mouth corner. However, this does not reflect a problem with the UV measurement method, but rather the fact that we measured two markers that were slightly offset from each other. (c) The cameras differed in the extent and timing of frame dropping. Frame dropping can have various causes (poor lighting conditions, overheating issues, automatic camera settings, camera drivers, etc.) and can be influenced only to a very limited extent by the experimental setting (e.g., increase UV illumination, increase the camera's ISO/gain settings). During the blenderFace tracking process, dropped frames are represented by duplicates of the preceding frame to maintain synchronization across the video recordings. Consequently, no movement is detected in these frames. Because the two cameras drop frames at different time points, the resulting motion trajectories differ between recordings. (d) The two cameras had different resolutions. Despite scaling the videos to the exact same head size and superimposing the videos for a visual control, the effective pixel size differed between cameras. This might arguably have slightly affected the pattern recognition algorithm. (e) In rare cases, sunscreen markers may reflect UV light so strongly that the marker pattern changes, thereby reducing tracking accuracy. This is the case for marker A7 (center of the upper lip) of Participant 1. While the tracker remained fixed to the contour of the upper lip (y‐axis), it drifted slightly along the x‐axis due to reflections, resulting in a lower correlation between the UV marker and the visible marker on the x‐axis.

In this pilot study, we found a high degree of correspondence between the movement trajectories obtained with the blenderFaceUV toolkit and those of the corresponding visible markers tracked with the blenderFace toolkit in both participants. Additionally, the substantially lower RMSE values after centering indicated that most of the absolute discrepancies between the two marker systems were attributable to spatial offsets rather than differences in the recorded movement trajectories. Thus, blenderFaceUV appeared to perform on par with the visible‐marker blenderFace toolkit for both pilot participants. The observed differences in the movement trajectories were minor and caused by the technical constraints of using two cameras. In Study 2, we examined the scalability of the blenderFaceUV toolkit, compared findings from the larger sample with those previously obtained using the visible‐marker blenderFace toolkit (Zinkernagel et al. 2019), and assessed the correspondence between marker movements measured with blenderFaceUV and those derived from OpenFace (Baltrušaitis et al. 2016), a widely used automated facial expression recognition system.

## 2 Study 2

### 2.1 Method

#### 2.1.1 Participants

Thirty‐one students from various disciplines at the University of Kaiserslautern‐Landau (RPTU, Germany) were recruited and given course credit or were paid for their participation. Data were collected during the COVID‐19 pandemic. The study was approved by the university administration and occupational health and safety authorities as part of the COVID‐19 pandemic restrictions and UV exposure setting (cf. https://doi.org/10.17605/OSF.IO/XTJBG). Prior to the experiment, we ensured that none of the participants had an allergy to sunscreens, polymorphic light eruption, neurodermatitis, eye disease, or heightened sensitivity to UV light. Because COVID‐19 contact restrictions required research assistants to conduct data collection with limited direct supervision and minimal participant contact, some procedural errors occurred.

Consequently, data from 13 participants (6 experimenter errors in positioning the UV camera, 2 incorrectly applied sunscreen markers, 1 error in headphone usage, 2 UV light errors, 2 technical camera recording failures) had to be excluded. This resulted in a final analysis sample of *N* = 18 participants (age *M* = 22.5, SD = 2.6; 66% women).^8^

#### 2.1.2 Design and Procedure

Participants entered the laboratory individually, where they were greeted and briefed by the experimenter. After providing informed consent, including consent to UV exposure and the use of video data, participants underwent a marker‐placement procedure similar to that used in Study 1. Unlike in Study 1, however, the experimenter applied the 68 facial markers using sunscreen and a cotton swab (see Figure 2b). The application of the “invisible” sunscreen markers required more practice than applying the visible markers of the original blenderFace method. Additionally, participants were provided with a headband and headphones with colored adhesive markers used to track head movement, which was necessary to separate facial movements from head movements.

Data were collected with two computers synchronized in time via the Network Time Protocol (NTP). The first computer presented the instructions and stimuli and recorded the participants' answers using PsychoPy (Peirce et al. 2019). The second computer was used for parallel UV and visible‐light video recordings via FFmpeg ver. 5.1.4 (https://ffmpeg.org/). As in Study 1, we used an XNiteUSB8M‐MNCUVL camera for UV recordings and a Logitech C920 webcam for visible‐light recordings. For both cameras, the participant's number, the current date, and timestamps were overlaid onto the video. Both cameras recorded at 30 frames per second and were stacked above each other at the top of the first computer screen so that they could capture almost exactly the same view of the participant's face (see Figure 1a). To reduce frame dropping and reflections from UV markers, in Study 2 we used an improved setup for UV illumination. Three UV fluorescent tubes positioned around the monitor served as the UV light source, and neon overhead lighting was used as the light source for the visual recording. Figure 1b shows an example frame from the visible‐light recording, where the UV markers are not visible, and Figure 1c shows a corresponding UV video image, in which the sunscreen markers are visible.

The subsequent data collection aimed to record sufficiently intense and variable facial activity to assess the tracking quality of UV markers across a broad range of facial movements. To this end, we prompted participants with familiar and easily understood emotion terms (happiness, sadness, fear, disgust, and anger)^9^ and recorded the elicited facial responses in parallel with two cameras (visual, UV) on the second computer. For each prompt, we instructed participants to imagine what they would look like when experiencing this emotion and then to hold each expression for 3 s. In the following analyses, we use these emotion labels to showcase and visualize blenderFaceUV tracking performance as broad response classes.

After completion of data collection, the participants were debriefed and asked to remove the sunscreen. Finally, the laboratory and the experimental materials were sterilized as required by the COVID‐19 guidelines and prepared for the next participant.

#### 2.1.3 Analysis

##### 2.1.3.1 Data Preparation for blenderFaceUV

As in Study 1, markers in the UV camera recordings were tracked following the blenderFace method (Zinkernagel et al. 2019). First, the markers stuck on the headband and headphones were tracked throughout the video recording to measure head movements and head turns. Second, for a short sequence of the video recording with no facial activity of the participant (≈30 frames), all 68 markers were tracked to capture the individual facial surface of the participant. Finally, the relevant markers were tracked for the phases of the video recording in which the participants responded to the expression prompts.

Figure 2a shows the positions of the markers on the face. BL2, BR2 (left and right inner eyebrows), DL3, DR3 (left and right markers below the outer corners of the eyes), CL4, CR4 (left and right markers on the cheeks), BL5, BR5 (left and right markers next to the nose), CL7, CR7 (left and right mouth corners), BL7, BR7 (left and right midpoints of the upper lip), and A7 (center of the upper lip) are highlighted in bold because they were selected for their particular relevance in distinguishing broad expression categories. Per participant, the tracking of these markers resulted in x‐, y‐, and z‐coordinates per marker and frame, with head movements accounted for via the markers on the headband and headphones.

The tracking data were post‐processed using functions from the blenderFace R package. Specifically, the coordinate data from all participants were concatenated into a single data frame, screened for artifacts and outliers, converted to millimeter units, and baseline‐corrected to zero for each marker and axis at the onset of each expression prompt. In addition, we generated a data file with movements scaled to a standardized face with a width and a height of one to eliminate the impact of different head sizes.

##### 2.1.3.2 Data Preparation for OpenFace

Video recordings from the visible‐light camera were analyzed with OpenFace (Baltrušaitis et al. 2016, version 2.2.0, Docker installation) using the standard parameters. These analyses yielded x‐, y‐, and z‐coordinates for all OpenFace markers in each frame.

OpenFace provides both 2D coordinates, indicated by lowercase labels and intended for projection onto the video image, and 3D coordinates, indicated by uppercase labels and intended for spatial analyses. For all further analyses of the OpenFace data, we used the 3D coordinates. According to the OpenFace documentation, movement of these markers is measured in mm. However, the OpenFace documentation does not explicitly address the extent to which head movements and rotations affect the reported coordinates (see Guo et al. 2018). We eliminated these confounds by developing and using the function transformOFcoords() in the blenderFace R package.^10^ Because the output from both measurement methods was scaled to mm units, the difference in camera resolution did not matter. We synchronized the frames of the two recordings by the timestamps in the videos. In addition, we extracted the timestamps marking the onsets and offsets of the instructions to pose a specific emotion from the PsychoPy logfiles and added this information to the relevant frames (e.g., adding “posing happiness” to every frame of the videos during the instructions to pose happiness for 3 s). To ensure that both the onset and offset of each facial expression were included, we added 1 s (30 frames) to the beginning and end of each expression episode. This procedure resulted in 146 frames (measurement occasions) per marker (x, y, and z coordinates) per emotion per participant for both measurements.

Subsequently, we selected the markers located at corresponding anatomical positions in the OpenFace and blenderFace measurements (see Figure 2a,c). The markers included the left (blenderFace label: BL2/OpenFace label: 21) and right (BR2/22) inner eyebrows, the center of the upper lip (A7/51), the left (CL7/48) and right (CR7/54) corners of the mouth, and the markers on the left (BL7/49) and right (BR7/53) upper‐lip landmarks located between the mouth corners and the center of the upper lip. For further analyses, we omitted the z‐axis in both measurements because it provided little additional diagnostic information (see Zinkernagel et al. 2019). As the coordinate origins of the OpenFace and blenderFace methods lie on the vertical symmetry axis of the face, the x‐coordinates of the right half of the face were mirrored so that the movements of the left and right halves of the face had the same direction. More specifically, we mirrored the x‐axis of the markers BR2/22, BR7/53, and CR7/54. Subsequently, we merged the OpenFace and blenderFace data by frame, emotion, and participant.

### 2.2 Results

Visual inspection in the laboratory and inspection of the visible‐light video recordings showed a faint overall blue tint. However, the sunscreen‐painted markers on the participants' faces were not visible (see Figure 1b). In contrast, UV markers were clearly visible in the video recordings in the UV range of light (see Figure 1c). This suggests that blenderFaceUV markers may remain visually unobtrusive in face‐to‐face settings while being reliably identifiable under UV illumination.

The UV‐marker tracking procedure closely mirrored the procedure used for visible markers and yielded a similar overall performance. More specifically, Blender's solve error, the mean reprojection error between tracked video markers and the corresponding reprojected points of the 3D model, was below 0.3 pixels in both the original blenderFace approach (cf. Zinkernagel et al. 2019) and the present study. This corresponds to an average deviation of approximately one third of a pixel between the observed video markers and the model's reprojected marker positions, indicating high precision in the reconstruction of movements.^11^

**FIGURE 4:**
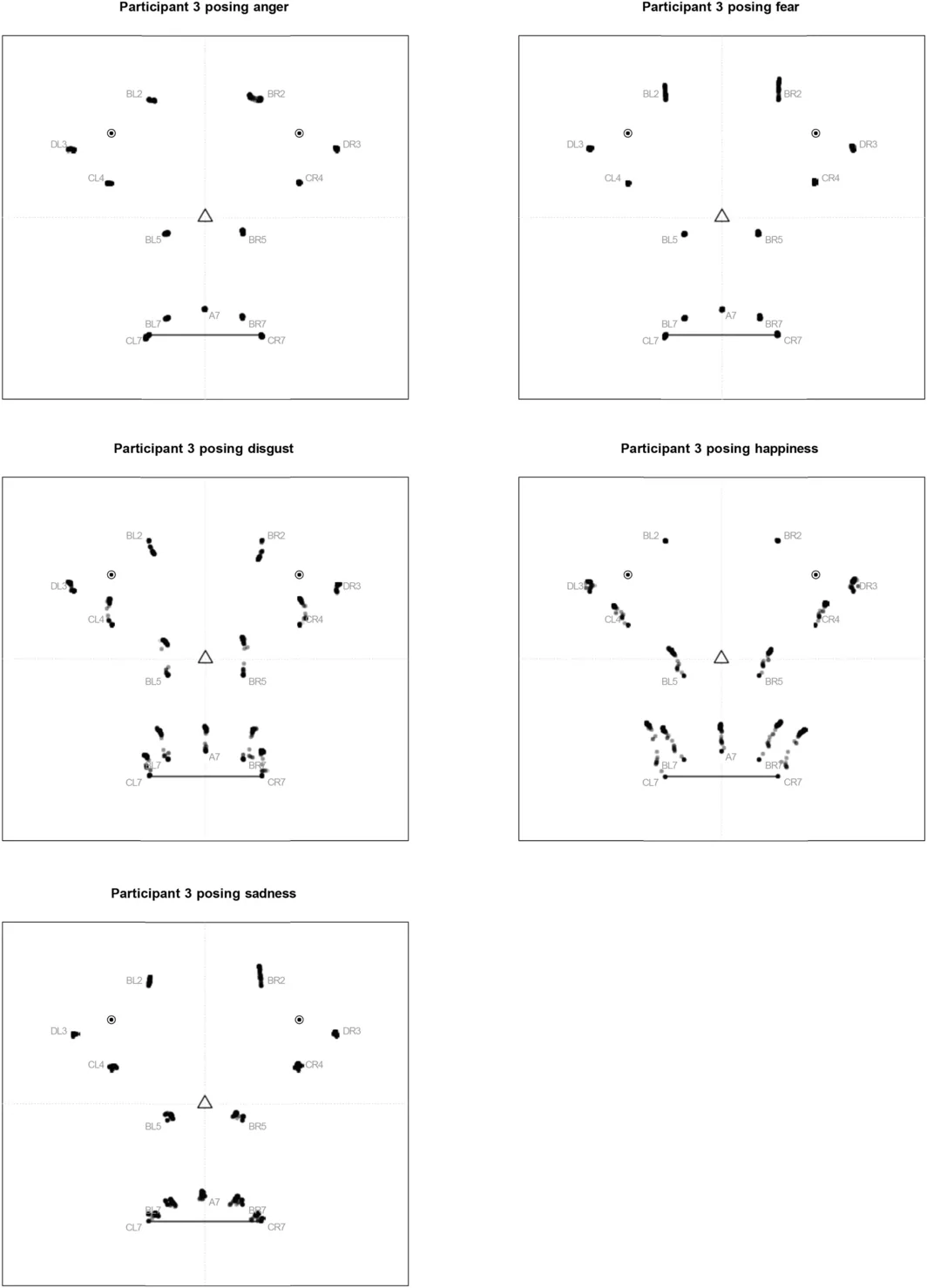
Baseline‐corrected and standardized marker movements of Participant 3, separated by expression prompt. For each expression prompt, the symmetrical (left and right) facial markers B2, D3, C4, B5, B7, C7, and marker A7 are plotted.

**FIGURE 5:**
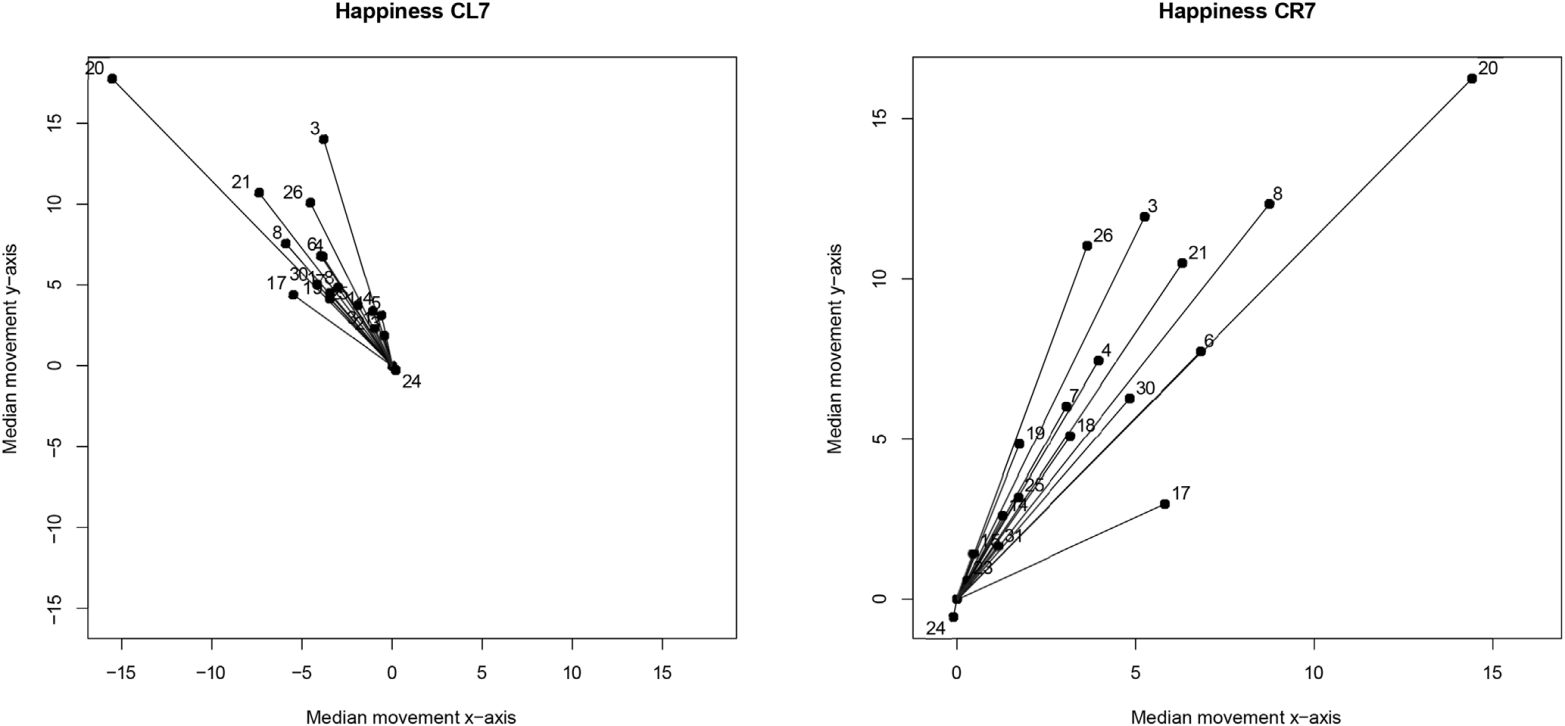
Plots of the baseline‐corrected median movement of the left and right mouth corner markers (CL7, CR7) for the happiness‐posing episode for each individual as denoted by the participant number. Movement is scaled to mm units.

**FIGURE 6:**
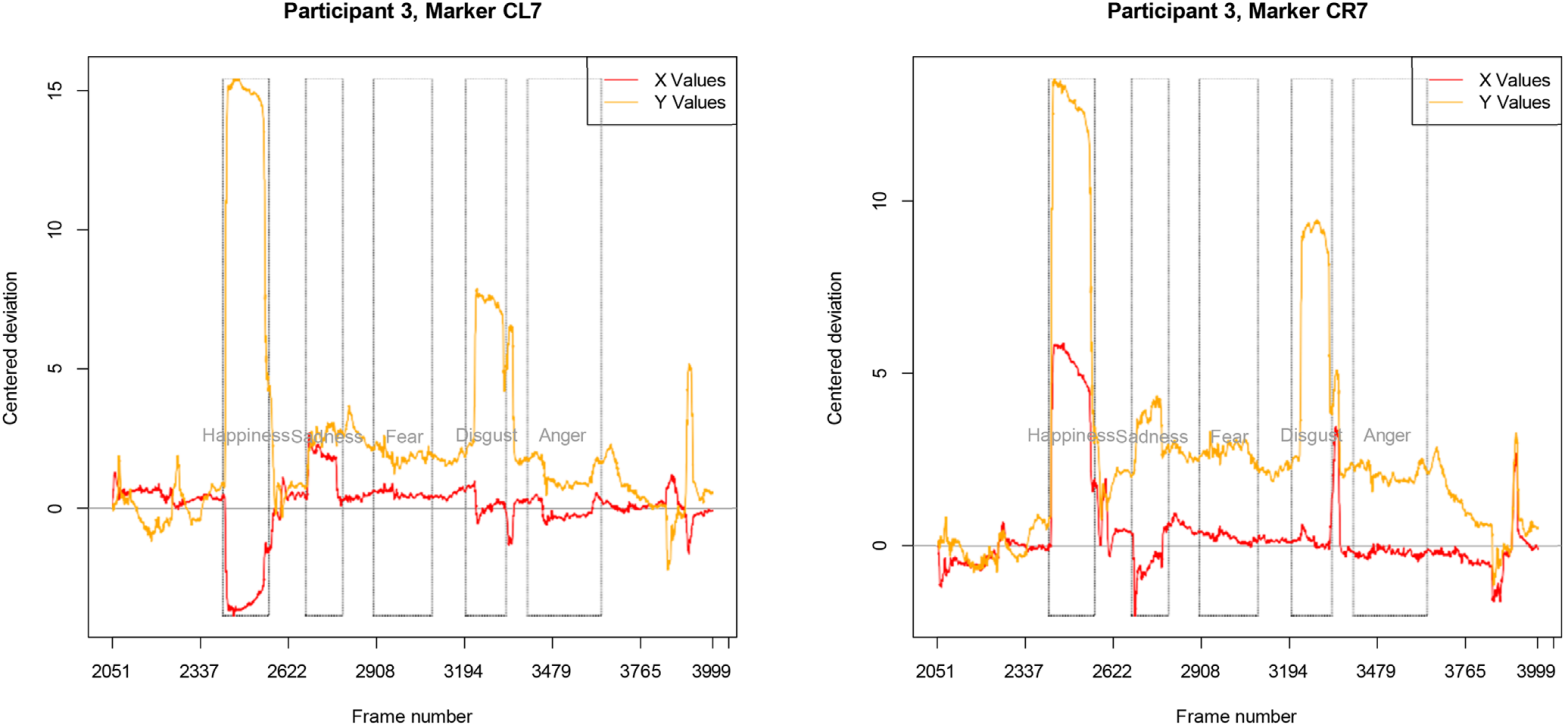
Marker movements for Participant 3 of the left (CL7) and right (CR7) mouth corner across all expression prompts. Note that the origin of the coordinate system is at the tip of the nose; therefore, the left and right markers move in opposite directions on the x‐axis (red lines). The plot's x‐axis represents the frame numbers, and the y‐axis represents the movement in mm.

**FIGURE 7:**
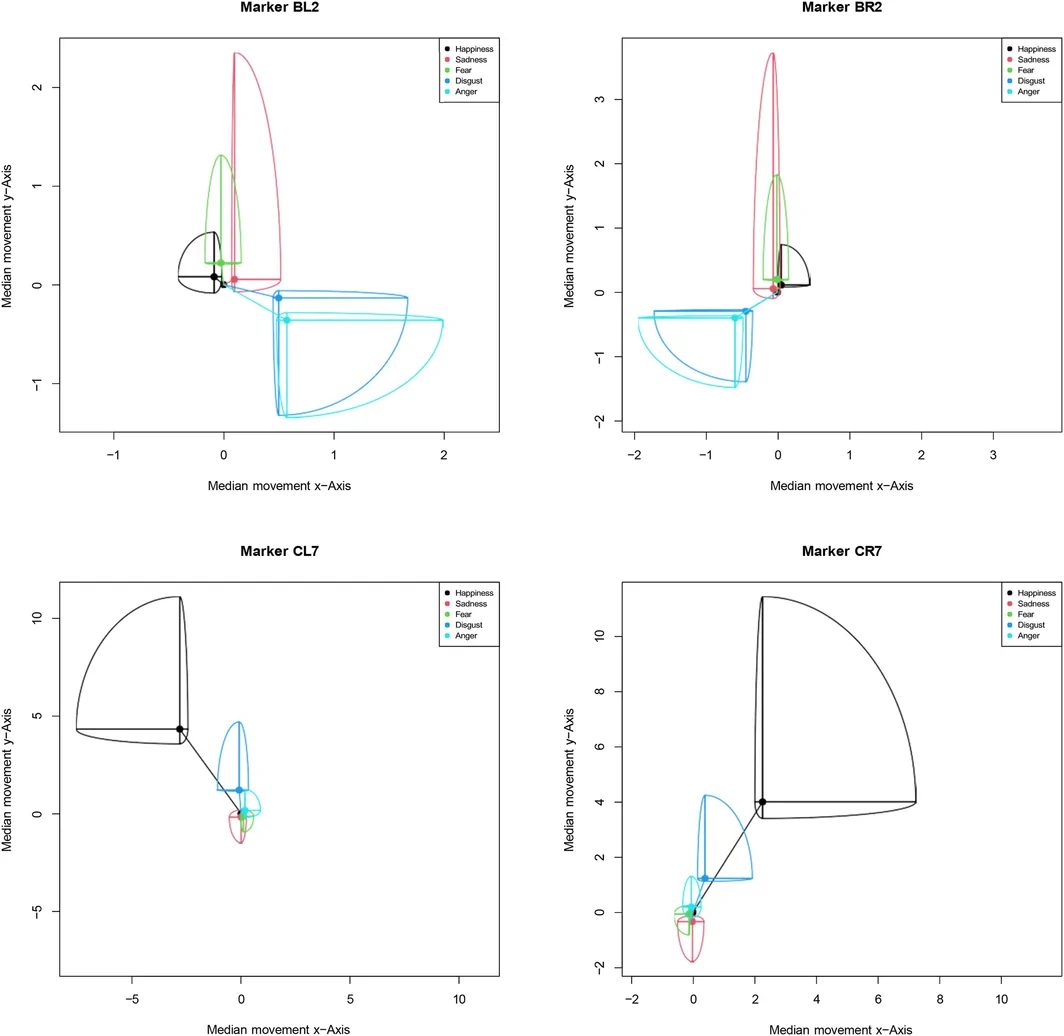
Baseline‐corrected median movement in mm of the markers BL2/BR2 (inner eyebrows) and CL7/CR7 (mouth corners) aggregated over participants in response to the different expression prompts. The ellipse represents the quartiles of the movement distribution for the x‐ and y‐axes. The x‐ and y‐axes of the plot are scaled to the same range so that the angle represents the true direction of movement. Different expression prompts are indicated by colors (black: Happiness; red: Sadness; green: Fear; dark blue: Disgust; light blue: Anger). All plots show a frontal view of the movements.

Next, we post‐processed the UV marker tracks according to the procedure of the blenderFaceUV toolkit (i. e., scaling to mm and to a standardized face; baseline setting to zero at the expression prompt onsets). The plots of Figures 4, 5, 6, 7 illustrate blenderFaceUV's sensitivity to both subtle and large marker movements throughout the face. Overall, these results show a similar level of sensitivity to marker movements assessed with visible markers in our prior work (Zinkernagel et al. 2019). Please note that only a subset of illustrative plots are presented here for clarity. A complete set of plots can also be generated using the plot functions of the blenderFace R package.

Figure 4 shows the baseline‐corrected and standardized raw facial movements of Participant 3 for each of the five expression prompts. The plots visualize the general movement trajectories per prompt and help to spot artifacts and outliers. As demonstrated by the figure, observed movements for this participant showed good face validity with no obvious outliers or artifacts.

Figure 5 shows the median movements of the left and right mouth corners (CL7, CR7) across all participants. The values were baseline‐corrected and converted to mm, allowing for direct comparison. This type of plot is particularly suitable for identifying outliers and unusual movements across participants. For example, Participant 20 had a particularly marked movement of the left and right mouth corners, whereas Participant 24 did not show any movement of the mouth corners (contrary to instructions) when instructed to pose happiness. In contrast to trained FACS coders, where we would expect more consistency, this type of visualization of the geometrically‐based movement patterns provides a simple at‐a‐glance overview of variability between participants.

Figure 6 visualizes the facial movements over time with respect to the different expression prompts. It shows an example (Participant 3) of baseline‐corrected x‐ and y‐axis movements converted to millimeters of markers CL7 and CR7 (left and right mouth corner) per frame.^12^ The gray boxes in the plots represent the respective expression prompt intervals. As illustrated by this example, different prompts successfully elicited clearly distinct movement patterns, which were consistently reflected at both marker positions. This suggests that geometric tracking can reliably track both small and large marker displacements across the range of movements observed in the present study.

Figure 7 presents the movement data at four marker positions, aggregated across all participants as a type of two‐dimensional box plot. Each panel shows the baseline‐corrected and converted to mm median movements (length of line from the baseline) of the markers.

BL2, BR2 (left and right inner eyebrows) and CL7, CR7 (left and right mouth corners) along with the quartiles (25%, 75%) of the distribution of the movements per expression prompt. These results illustrate that blenderFaceUV provides a powerful tool for separating and summarizing consistent movement patterns across participants in two‐dimensional space despite substantial variability between participants in their response to the prompts. For example, lay participants prompted with “happiness” (black) tended to move their left and right inner eyebrows (BL2, BR2) very slightly outwards, while the left and right mouth corners (CL7, CR7) tended to move upward and outward to a median of ≈5 mm. In contrast, the lay participants prompted with “sadness” (red) more typically moved their left and right inner eyebrows (BL2, BR2) upward up to 2 mm for some participants, while the left and right mouth corners (CL7, CR7) moved slightly downward. Although the present study was not designed to test any experimental hypotheses about differences between conditions, we recognize the potential of blenderFace(UV) as an approach to help characterize and summarize fine‐grained differences in facial responses between participants and/or stimulus conditions. It thus provides a detailed visual descriptive language that is closely based on the observable response and, as a method, decoupled from ongoing theoretical debates in emotion research.

#### 2.2.1 Comparison of blenderFaceUV and OpenFace Measurements

**FIGURE 8:**
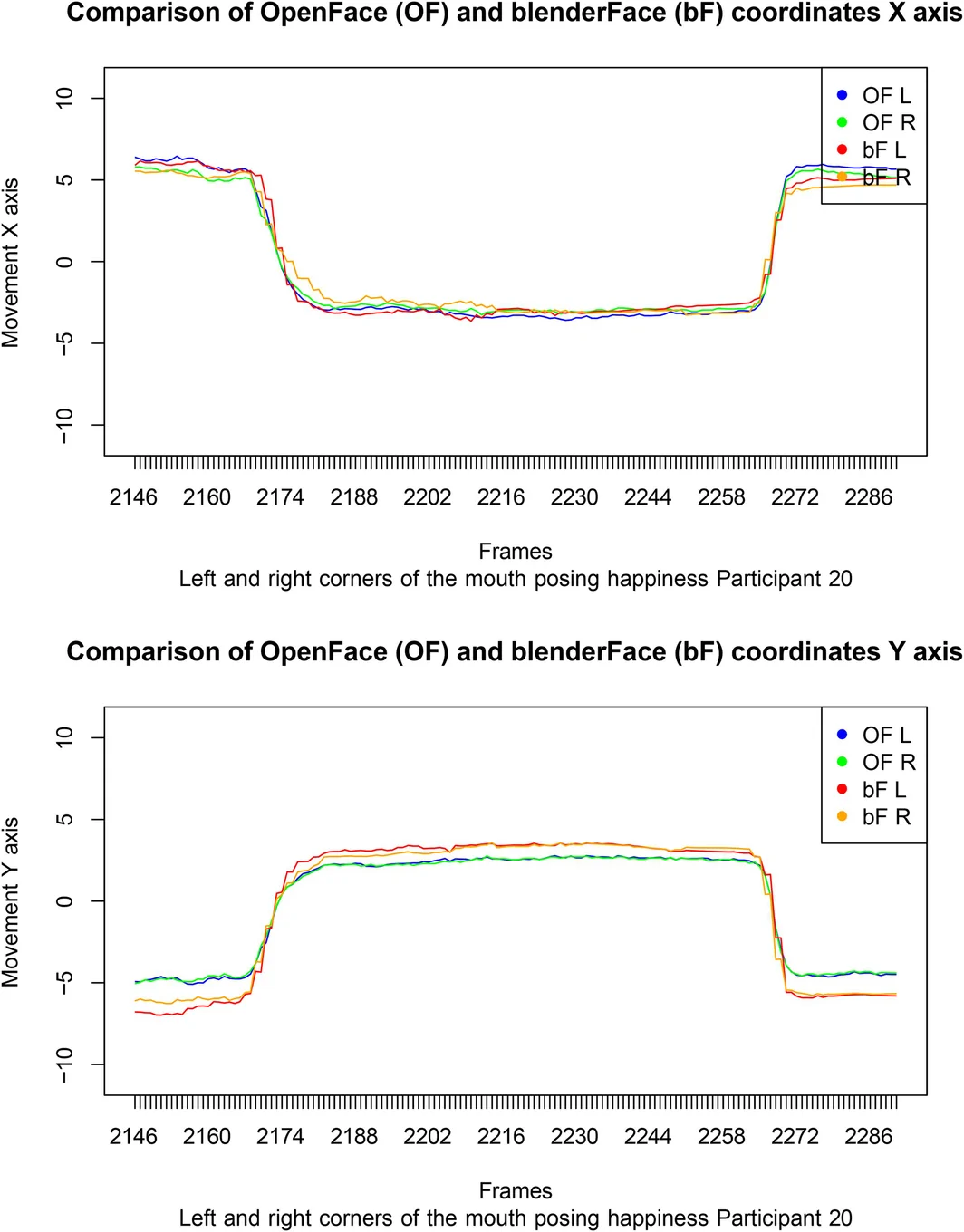
Comparison of the movement in mm of the left (L) and the right (R) corners of the mouth (BL7/49; BR7/53) per frame of a happiness‐posing episode for Participant 20 simultaneously measured with OpenFace (OF; blue, green) and blenderFace (bF; red, orange) separated for the x‐ and y‐axes.

Because both measurement methods use the same metric (mm) but different origins of their coordinate systems, we chose to center but not to scale the blenderFaceUV and OpenFace data. To visually assess the consistency of the two measurement methods we generated plots for each axis, marker, and participant (see Supplement, Study 2, Plots, 02_OpenFace_blenderFaceUV_Comparison). An illustrative plot for the movement of the left and right corners of the mouth (BL7/49; BR7/53) of Participant 20 posing happiness is shown in Figure 8, separately for the x‐ and y‐axes. Examining these plots showed that the movements along the x‐ and y‐axes in the OpenFace and blenderFace data were parallel across most frames, including during pronounced movements at the emotion onset and offset. This finding suggests that the synchronization of the two video recordings was successful on a per‐frame basis.

As in Study 1, we expected the measurements to be identical because they captured the same individuals at the same time points, albeit using two different cameras and two different analysis methods. To assess the agreement between the two measurement methods, we computed correlations between the OpenFace and blenderFace measurements, separately for happiness, sadness, fear, disgust, and anger, per participant and marker (Tables 3, 4, 5, 6, 7). To facilitate the interpretation of the large number of correlations, we computed the percentage of high correlations *r* > 0.80, at least moderate positive or negative correlations (*r* > 0.30, *r* < *−*0.30), and the percentage of small or null correlations (*−*0.30 ≤ *r* ≤ 0.30) for every marker (averaged across columns) and every participant (averaged across rows).

**TABLE 3:** Correlations between blenderFaceUV and OpenFace markers for the emotion of happiness.

Participant	BL2_x, X_21	BL2_y, Y_21	BR2_x, X_22	BR2_y, Y_22	CL7_x, X_48	CL7_y, Y_48	BL7_x, X_49	BL7_y, Y_49	A7_x, X_51	A7_y, Y_51	BR7_x, X_53	BR7_y, Y_53	CR7_x, X_54	CR7_y, Y_54	*r* > 0.80	*r* > 0.30	*−*0.30 ≤ *r* ≤ 0.30	*r* < *−*0.30
3	−0.68	0.16	0.38	−0.36	0.99	0.99	0.99	0.99	0.62	0.99	0.98	0.99	0.98	0.99	64%	78%	7%	14%
4	0.83	0.91	0.68	0.93	0.98	0.98	0.97	0.95	−0.23	0.93	0.98	0.96	0.98	0.96	85%	92%	7%	0%
6	−0.70	−0.18	0.75	0.28	0.98	0.99	0.99	0.99	0.28	0.98	0.99	0.96	0.99	0.98	64%	71%	21%	7%
7	0.31	0.15	0.31	0.31	0.99	0.99	0.93	0.97	0.56	0.96	0.98	0.98	0.97	0.99	64%	92%	7%	0%
8	0.48	−0.66	0.46	−0.55	0.99	0.99	(*)	(*)	0.13	0.98	0.98	0.98	0.98	0.98	50%	64%	7%	14%
14	0.25	−0.79	0.92	−0.64	0.99	0.94	0.99	0.89	0.49	0.80	0.99	0.95	0.98	0.98	64%	78%	7%	14%
15	0.80	0.95	0.89	0.94	0.98	0.96	0.97	1.00	−0.49	0.99	0.99	0.97	0.97	0.99	85%	92%	0%	7%
17	0.03	0.48	0.61	0.79	0.99	0.95	0.99	0.95	0.82	0.95	0.90	0.97	0.99	0.87	71%	92%	7%	0%
18	0.67	0.59	0.60	0.66	0.97	0.96	0.94	0.93	0.77	0.86	0.92	0.95	0.98	0.99	64%	100%	0%	0%
19	0.64	0.44	−0.17	0.61	0.98	0.98	0.95	0.95	0.72	0.94	0.92	0.97	0.97	0.98	64%	92%	7%	0%
20	0.29	−0.36	0.19	−0.37	0.99	1.00	0.99	1.00	−0.20	0.99	0.99	0.99	0.99	1.00	64%	64%	21%	14%
21	0.82	−0.94	0.60	−0.92	1.00	1.00	1.00	0.99	−0.74	1.00	1.00	0.99	1.00	1.00	71%	78%	0%	21%
23	−0.41	0.86	0.85	0.95	0.88	0.96	0.95	0.90	0.97	0.90	0.93	−0.43	0.84	0.73	78%	85%	0%	14%
24	0.24	−0.07	0.44	−0.60	0.69	0.43	0.54	0.22	0.17	0.43	−0.12	0.63	0.53	0.76	0%	57%	35%	7%
25	0.74	0.72	0.14	0.49	0.98	0.99	0.99	0.98	0.19	0.94	0.93	0.99	0.97	0.98	64%	85%	14%	0%
26	0.88	0.57	0.85	0.63	0.98	0.99	0.98	0.99	−0.15	0.95	0.98	0.99	0.98	1.00	78%	92%	7%	0%
30	0.69	−0.38	−0.51	−0.44	0.99	0.96	0.98	0.97	0.71	0.94	0.99	0.98	0.98	0.99	64%	78%	0%	21%
31	−0.02	−0.17	0.18	−0.53	0.99	0.99	0.99	0.99	0.57	1.00	0.98	0.99	0.97	0.98	64%	71%	21%	7%
*r* > 0.80	16%	16%	22%	16%	94%	94%	88%	88%	11%	88%	94%	88%	94%	88%				
*r* > 0.30	55%	44%	72%	50%	100%	100%	94%	88%	50%	100%	94%	94%	100%	100%				
*−*0.30 ≤ *r* ≤ 0.30	27%	27%	22%	5%	0%	0%	0%	5%	38%	0%	5%	0%	0%	0%				
*r* < *−*0.30	16%	27%	5%	44%	0%	0%	0%	0%	11%	0%	0%	5%	0%	0%				

*Note:* Correlations between blenderFaceUV and OpenFace marker movements separated for the x‐ and y‐axes based on 146 frames. In addition, we present the percentages of correlations *r* > 0.80, at least moderate positive or negative correlations (*r* > 0.30, *r* < *−*0.30), and small or null correlations (*−*0.30 ≤ *r* ≤ 0.30) for every marker (columns) and every participant (rows). An asterisk (*) indicates a marker missing for one person (see text).

**TABLE 4:** Correlations between blenderFaceUV and OpenFace markers for the emotion of sadness.

Participant	BL2_x, X_21	BL2_y, Y_21	BR2_x, X_22	BR2_y, Y_22	CL7_x, X_48	CL7_y, Y_48	BL7_x, X_49	BL7_y, Y_49	A7_x, X_51	A7_y, Y_51	BR7_x, X_53	BR7_y, Y_53	CR7_x, X_54	CR7_y, Y_54	*r* > 0.80	*r* > 0.30	*−*0.30 ≤ *r* ≤ 0.30	*r* < *−*0.30
3	0.81	0.08	0.92	0.00	0.57	0.18	0.53	0.47	0.13	0.65	0.25	0.37	0.49	−0.34	14%	57%	35%	7%
4	−0.54	−0.51	−0.34	−0.37	0.48	0.30	0.54	0.85	0.67	0.57	0.74	0.89	0.65	0.50	14%	64%	7%	28%
6	0.08	−0.51	0.08	0.13	0.29	0.60	0.71	0.48	−0.33	0.36	0.34	0.29	0.89	0.69	7%	50%	35%	14%
7	0.14	0.75	0.88	0.68	0.85	0.78	0.93	0.85	0.96	0.82	0.96	0.85	0.93	0.70	64%	92%	7%	0%
8	0.97	0.84	−0.19	0.83	0.72	0.98	(*)	(*)	0.06	0.43	0.14	0.72	0.70	0.92	35%	64%	21%	0%
14	−0.31	−0.81	0.21	−0.81	0.84	0.20	0.53	−0.52	−0.12	−0.58	0.72	−0.54	0.80	0.50	7%	35%	21%	42%
15	−0.32	−0.43	0.00	0.40	−0.02	0.86	0.17	0.59	0.30	0.70	0.48	0.73	0.19	0.92	14%	50%	35%	14%
17	0.16	0.86	0.62	0.86	0.96	0.96	0.80	0.96	0.42	0.85	0.90	0.85	0.79	0.93	64%	92%	7%	0%
18	−0.42	0.72	0.47	0.76	−0.02	0.79	−0.33	0.71	0.64	0.87	−0.19	0.80	0.71	0.79	7%	71%	14%	14%
19	−0.05	0.28	0.56	0.34	0.50	0.62	0.16	0.89	0.16	0.94	0.41	0.86	0.75	0.75	21%	71%	28%	0%
20	0.47	0.61	0.78	0.68	0.13	0.39	−0.22	0.07	0.73	−0.03	0.91	0.29	0.88	0.61	14%	64%	35%	0%
21	0.27	0.06	−0.41	0.15	−0.28	0.77	0.08	−0.22	−0.36	0.00	0.32	0.27	0.74	0.70	0%	28%	57%	14%
23	0.57	0.98	0.56	0.97	0.40	0.25	0.50	−0.50	0.74	−0.85	0.21	−0.59	−0.44	−0.11	14%	50%	21%	28%
24	0.98	−0.93	0.98	0.84	0.94	0.08	0.93	0.58	−0.36	0.39	0.93	0.02	0.90	−0.42	50%	64%	14%	21%
25	−0.35	0.58	0.52	0.01	0.19	0.00	0.51	0.24	0.61	0.38	0.22	0.07	−0.01	0.05	0%	35%	57%	7%
26	−0.02	−0.14	0.36	−0.05	0.96	0.98	0.84	0.45	0.04	0.93	0.79	0.97	0.79	0.99	42%	71%	28%	0%
30	0.31	−0.31	−0.03	−0.39	0.90	0.77	0.79	0.85	0.56	0.85	0.46	0.65	0.82	0.85	35%	78%	7%	14%
31	−0.24	0.01	0.07	0.31	0.09	0.71	−0.28	0.70	−0.26	0.71	0.54	0.68	0.65	0.74	0%	57%	42%	0%
*r* > 0.80	16%	16%	16%	22%	33%	22%	16%	27%	5%	33%	22%	27%	27%	27%				
*r* > 0.30	33%	38%	55%	55%	61%	66%	61%	66%	44%	77%	72%	61%	83%	77%				
*−*0.30 ≤ *r* ≤ 0.30	38%	27%	33%	27%	38%	33%	27%	16%	38%	11%	27%	27%	11%	11%				
*r* < *−*0.30	27%	33%	11%	16%	0%	0%	5%	11%	16%	11%	0%	11%	5%	11%				

*Note:* Correlations between blenderFaceUV and OpenFace marker movements separated for the x‐ and y‐axes based on 146 frames. In addition, we present the percentages of correlations *r* > 0.80, at least moderate positive or negative correlations (*r* > 0.30, *r* < *−*0.30), and small or null correlations (*−*0.30 ≤ *r* ≤ 0.30) for every marker (columns) and every participant (rows). An asterisk (*) indicates a marker missing for one person (see text).

**TABLE 5:** Correlations between blenderFaceUV and OpenFace markers for the emotion of fear.

Participant	BL2_x, X_21	BL2_y, Y_21	BR2_x, X_22	BR2_y, Y_22	CL7_x, X_48	CL7_y, Y_48	BL7_x, X_49	BL7_y, Y_49	A7_x, X_51	A7_y, Y_51	BR7_x, X_53	BR7_y, Y_53	CR7_x, X_54	CR7_y, Y_54	*r* > 0.80	*r* > 0.30	*−*0.30 ≤ *r* ≤ 0.30	*r* < *−*0.30
3	0.21	0.35	−0.13	0.34	0.17	0.34	0.67	−0.32	0.43	−0.44	−0.08	−0.54	−0.10	−0.69	0%	35%	35%	28%
4	0.12	−0.12	−0.73	−0.35	0.79	0.80	0.66	0.10	0.76	0.23	0.12	0.09	0.05	0.84	7%	35%	50%	14%
6	0.63	−0.68	0.41	0.75	0.90	−0.01	0.88	−0.60	0.85	−0.29	0.24	0.46	0.08	0.72	21%	57%	28%	14%
7	−0.22	0.86	0.91	0.89	0.80	0.33	0.67	0.69	0.79	0.77	0.90	0.79	0.76	0.82	35%	92%	7%	0%
8	−0.03	−0.17	−0.06	0.00	0.17	0.30	(*)	(*)	0.25	−0.04	−0.31	0.10	−0.06	0.29	0%	0%	78%	7%
14	−0.11	0.33	0.29	−0.14	−0.61	0.44	−0.50	0.35	−0.24	0.04	0.41	−0.35	0.26	−0.06	0%	28%	50%	21%
15	0.29	−0.42	0.50	0.06	0.14	0.63	0.50	0.61	0.39	0.37	0.12	0.55	0.43	0.72	0%	64%	28%	7%
17	0.69	0.49	0.84	0.33	0.55	0.81	0.36	−0.02	0.28	0.65	0.70	−0.32	0.85	0.93	28%	78%	14%	7%
18	0.35	0.98	−0.38	0.98	0.91	0.92	0.89	0.85	0.47	0.34	0.84	0.83	0.89	0.97	71%	92%	0%	7%
19	0.67	0.07	−0.31	−0.16	0.43	0.94	0.60	0.02	0.55	−0.17	0.40	0.00	0.82	0.82	21%	57%	35%	7%
20	0.78	0.48	0.66	−0.45	−0.13	−0.06	0.47	0.68	0.22	0.82	−0.24	0.71	−0.17	0.09	7%	50%	42%	7%
21	0.53	0.09	0.54	0.22	0.55	0.50	0.50	0.10	−0.18	0.30	−0.31	0.29	−0.19	0.41	0%	42%	50%	7%
23	−0.10	0.73	0.32	0.72	0.78	0.40	0.74	0.62	0.67	0.92	0.64	0.92	0.78	0.64	14%	92%	7%	0%
24	0.38	0.68	0.89	−0.22	0.98	0.98	0.96	0.57	0.49	−0.78	0.66	0.42	0.98	0.98	42%	85%	7%	7%
25	0.19	−0.02	0.42	0.23	0.27	0.06	−0.13	−0.01	0.19	0.11	0.08	0.17	−0.27	−0.01	0%	7%	92%	0%
26	−0.39	0.65	−0.17	0.66	0.87	0.66	−0.06	0.66	0.46	0.31	−0.48	0.61	0.83	0.43	14%	71%	14%	14%
30	0.80	0.93	0.93	0.87	0.91	0.87	0.59	0.22	−0.23	0.06	0.93	0.01	0.95	0.92	57%	71%	28%	0%
31	0.54	−0.35	−0.13	−0.21	0.14	0.44	−0.67	0.41	−0.48	0.84	−0.35	0.81	0.88	0.89	28%	50%	21%	28%
*r* > 0.80	0%	16%	22%	16%	27%	27%	16%	5%	5%	16%	16%	16%	38%	44%				
*r* > 0.30	50%	55%	55%	44%	61%	77%	72%	50%	55%	44%	44%	50%	55%	72%				
*−*0.30 ≤ *r* ≤ 0.30	44%	27%	27%	44%	33%	22%	11%	33%	38%	44%	33%	33%	44%	22%				
*r* < *−*0.30	5%	16%	16%	11%	5%	0%	11%	11%	5%	11%	22%	16%	0%	5%				

*Note:* Correlations between blenderFaceUV and OpenFace marker movements separated for the x‐ and y‐axes based on 146 frames. In addition, we present the percentages of correlations *r* > 0.80, at least moderate positive or negative correlations (*r* > 0.30, *r* < *−*0.30), and small or null correlations (*−*0.30 ≤ *r* ≤ 0.30) for every marker (columns) and every participant (rows). An asterisk (*) indicates a marker missing for one person (see text).

**TABLE 6:** Correlations between blenderFaceUV and OpenFace markers for the emotion of disgust.

Participant	BL2_x, X_21	BL2_y, Y_21	BR2_x, X_22	BR2_y, Y_22	CL7_x, X_48	CL7_y, Y_48	BL7_x, X_49	BL7_y, Y_49	A7_x, X_51	A7_y, Y_51	BR7_x, X_53	BR7_y, Y_53	CR7_x, X_54	CR7_y, Y_54	*r* > 0.80	*r* > 0.30	*−*0.30 ≤ *r* ≤ 0.30	*r* < *−*0.30
3	0.89	0.95	0.68	0.90	0.87	0.94	0.95	0.98	0.20	0.99	0.90	0.98	0.65	0.89	78%	92%	7%	0%
4	0.72	0.46	0.74	0.32	0.95	0.49	0.50	0.80	0.55	0.76	0.54	0.72	0.76	0.45	7%	100%	0%	0%
6	0.76	−0.38	0.46	0.69	−0.14	0.78	0.73	0.96	−0.63	0.80	0.59	0.95	0.41	0.83	21%	78%	7%	14%
7	0.47	0.66	0.86	0.67	0.90	0.70	−0.06	0.88	0.57	0.88	0.79	0.88	0.93	0.76	42%	92%	7%	0%
8	0.99	−0.08	0.99	0.75	0.60	0.51	(*)	(*)	−0.25	0.01	0.94	0.26	0.57	0.92	28%	57%	28%	0%
14	0.49	0.77	0.42	0.76	0.32	0.30	0.13	0.41	−0.26	0.49	0.87	0.66	0.36	0.48	7%	78%	21%	0%
15	−0.44	0.71	0.55	0.77	0.04	1.00	0.99	0.99	0.80	1.00	0.98	0.99	−0.29	0.94	50%	78%	14%	7%
17	0.94	0.93	0.79	0.25	0.99	0.99	0.99	0.99	0.56	0.97	0.99	0.99	0.97	0.93	78%	92%	7%	0%
18	0.45	0.94	0.70	0.96	0.80	0.40	0.81	0.57	0.68	0.75	0.16	0.54	0.57	−0.12	21%	85%	14%	0%
19	0.80	0.21	0.86	0.31	−0.26	0.25	0.25	0.84	0.20	0.87	0.78	0.86	0.72	0.71	28%	64%	35%	0%
20	−0.47	0.95	0.44	0.96	0.40	0.94	0.82	0.98	−0.72	0.99	0.99	0.99	0.79	0.98	64%	85%	0%	14%
21	0.61	0.45	0.37	0.03	0.81	−0.04	0.58	−0.50	0.14	−0.61	0.58	−0.19	0.59	−0.08	7%	50%	35%	14%
23	0.85	0.90	0.72	0.83	−0.08	0.55	0.17	0.72	0.40	0.67	0.63	0.55	0.40	−0.10	21%	78%	21%	0%
24	0.96	0.67	0.92	0.85	0.77	0.40	0.80	0.04	−0.52	0.06	0.70	0.02	0.55	0.79	21%	71%	21%	7%
25	0.77	0.81	0.75	0.15	0.23	0.94	−0.08	0.93	0.77	0.97	0.90	0.95	0.73	0.86	50%	78%	21%	0%
26	0.76	0.63	−0.70	0.91	0.94	0.96	0.90	0.93	0.63	0.88	0.45	0.93	0.79	0.96	57%	92%	0%	7%
30	0.39	0.91	0.78	0.35	0.86	0.86	0.91	0.93	−0.27	0.90	0.95	0.88	0.94	0.91	71%	92%	7%	0%
31	0.93	0.08	0.87	0.85	0.70	0.52	0.44	0.56	0.90	0.17	0.90	0.73	0.89	0.95	50%	85%	14%	0%
*r* > 0.80	33%	38%	27%	38%	38%	38%	38%	55%	5%	50%	50%	55%	22%	55%				
*r* > 0.30	88%	77%	94%	83%	72%	83%	66%	83%	50%	77%	94%	83%	94%	83%				
*−*0.30 ≤ *r* ≤ 0.30	0%	16%	0%	16%	27%	16%	27%	5%	33%	16%	5%	16%	5%	16%				
*r* < *−*0.30	11%	5%	5%	0%	0%	0%	0%	5%	16%	5%	0%	0%	0%	0%				

*Note:* Correlations between blenderFaceUV and OpenFace marker movements separated for the x‐ and y‐axes based on 146 frames. In addition, we present the percentages of correlations *r* > 0.80, at least moderate positive or negative correlations (*r* > 0.30, *r* < *−*0.30), and small or null correlations (*−*0.30 ≤ *r* ≤ 0.30) for every marker (columns) and every participant (rows). An asterisk (*) indicates a marker missing for one person (see text).

**TABLE 7:** Correlations between blenderFaceUV and OpenFace markers for the emotion of Anger.

Participant	BL2_x, X_21	BL2_y, Y_21	BR2_x, X_22	BR2_y, Y_22	CL7_x, X_48	CL7_y, Y_48	BL7_x, X_49	BL7_y, Y_49	A7_x, X_51	A7_y, Y_51	BR7_x, X_53	BR7_y, Y_53	CR7_x, X_54	CR7_y, Y_54	*r* > 0.80	*r* > 0.30	*−*0.30 ≤ *r* ≤ 0.30	*r* < *−*0.30
3	0.97	0.62	0.96	−0.78	0.18	0.91	0.33	0.71	0.58	0.59	0.18	0.29	−0.03	0.88	28%	64%	28%	7%
4	−0.44	−0.13	0.04	0.39	0.17	0.37	0.50	0.46	0.53	0.84	0.86	0.21	0.82	0.21	21%	57%	35%	7%
6	−0.14	−0.17	0.93	0.94	−0.76	0.89	−0.42	0.91	−0.55	0.86	−0.03	0.91	0.06	0.83	50%	50%	28%	21%
7	0.93	0.95	0.90	0.98	0.34	0.84	0.61	0.36	0.74	0.84	0.82	0.94	0.50	0.97	64%	100%	0%	0%
8	0.97	0.92	0.86	0.91	0.85	0.67	(*)	(*)	0.15	−0.18	0.87	0.52	0.89	0.86	57%	71%	14%	0%
14	0.22	0.19	0.36	0.19	0.04	−0.08	−0.24	−0.53	−0.05	−0.28	0.56	−0.21	0.77	0.53	0%	28%	64%	7%
15	0.97	0.93	0.97	0.94	−0.23	0.42	0.13	0.87	−0.61	0.86	0.18	0.81	−0.30	0.12	50%	57%	35%	7%
17	0.97	0.94	0.89	0.92	0.70	0.78	0.65	0.93	0.6	0.84	0.87	0.89	0.46	0.89	64%	92%	7%	0%
18	0.97	0.97	0.97	0.95	−0.12	0.95	0.53	0.94	0.06	0.95	0.38	0.98	0.19	0.97	64%	78%	21%	0%
19	0.95	−0.11	0.82	0.54	−0.27	−0.42	0.50	0.74	0.02	0.86	−0.77	0.71	−0.41	0.23	21%	50%	28%	21%
20	0.98	0.95	0.98	0.84	0.31	0.61	0.31	0.88	0.65	0.67	0.75	0.88	0.92	0.73	50%	100%	0%	0%
21	−0.09	0.93	0.45	0.61	0.56	−0.13	0.55	−0.38	0.23	−0.07	0.21	0.17	0.18	0.44	7%	42%	50%	7%
23	0.91	−0.58	0.94	0.60	0.31	−0.54	0.74	−0.29	0.19	−0.14	−0.58	−0.45	−0.72	−0.68	14%	35%	21%	42%
24	0.30	−0.22	−0.03	−0.10	−0.16	−0.22	0.46	−0.01	0.57	0.17	0.61	−0.35	0.52	−0.22	0%	28%	64%	7%
25	0.72	−0.34	0.05	−0.02	0.22	−0.03	0.22	0.11	0.02	0.13	−0.18	−0.02	−0.27	0.40	0%	14%	78%	7%
26	−0.48	0.80	0.78	0.67	0.51	0.31	0.11	−0.09	0.62	−0.30	0.51	0.06	0.80	0.38	0%	64%	28%	7%
30	0.95	0.95	0.90	0.93	0.18	−0.13	0.26	−0.16	0.23	−0.40	0.21	−0.35	0.49	−0.12	28%	35%	50%	14%
31	0.93	0.70	0.97	0.94	−0.15	−0.67	0.08	−0.71	−0.25	−0.66	0.19	−0.67	0.57	−0.53	21%	35%	28%	35%
*r* > 0.80	61%	44%	66%	50%	5%	22%	0%	27%	0%	38%	22%	33%	16%	33%				
*r* > 0.30	66%	61%	83%	77%	38%	55%	55%	50%	33%	50%	50%	44%	55%	61%				
*−*0.30 ≤ *r* ≤ 0.30	22%	27%	16%	16%	55%	27%	33%	27%	55%	38%	38%	33%	33%	27%				
*r* < *−*0.30	11%	11%	0%	5%	5%	16%	5%	16%	11%	11%	11%	22%	11%	11%				

*Note:* Correlations between blenderFaceUV and OpenFace marker movements separated for x‐ and y‐axes based on 146 frames. In addition, we present the percentages of correlations *r* > 0.80, at least moderate positive or negative correlations (*r* > 0.30, *r* < *−*0.30), and small or null correlations (*−*0.30 ≤ *r* ≤ 0.30) for every marker (columns) and every participant (rows). An asterisk (*) indicates a marker missing for one person (see text).

The correlation tables show mainly high positive correlations up to *r* = 1.00 between marker movements on both the x‐ and y‐axes. However, strong negative correlations also occurred, up to a maximum of *r* = *−*0.94. This observation was initially unexpected, but we thoroughly investigated it in subsequent analyses.

Happiness was the emotion with the highest correlations between the OpenFace and blenderFaceUV measurements. On average, 64% of all correlations were *r* > 0.80, ranging from 16% to 94%. Looking only at the correlations between markers on the lower part of the face (corners of the mouth and upper lip, markers CL7/X_48 to CR7_y/Y_54), the mean and the range of values were higher (83%, range = 11%–94%). By contrast, in the upper part of the face (eyebrows, markers BL2_x/X_21 to BR2_y/Y_22), large negative correlations occurred (24%, range = 5%–44%). One finding that applied to all emotions, however, was that the correlation for the x‐axis of marker A7/X_51 (center of the upper lip) was very low. The emotions with the next highest percentage of correlations (*r* > 0.80) were disgust (38%, range = 5%–55%), anger (29%, range = 0%–66%), sadness (22%, range = 5%–33%), and fear (18%, range = 5%–44%). Whereas disgust had high correlations (*r* > 0.80) across almost all markers, anger showed high correlations mainly in the upper part of the face (eyebrows, markers BL2_x/X_21 to BR2_y/Y_22).

At the individual level as well, a low percentage of high correlations (*r* > 0.80) occurred for some participants. For example, for Participant 24, no marker correlation was *r* > 0.80 for the instructions to pose happiness. Participants 14 and 21 also had a very low percentage of high correlations (*r* > 0.80) across emotions.

**FIGURE 9:**
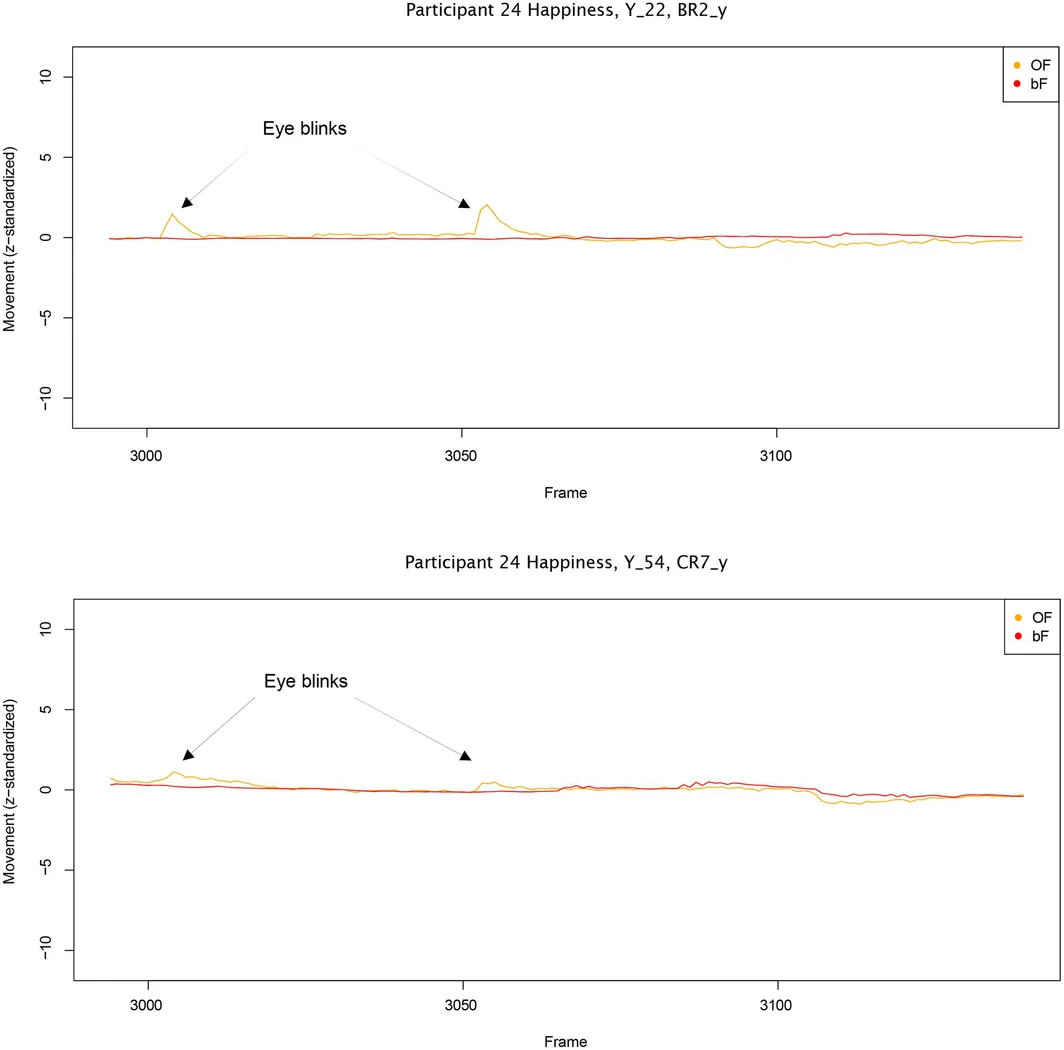
Comparison of the movement in mm of Participant 24's left inner eyebrow (22_Y, BR2_Y; upper part) and the left corner of Participant 24's mouth (54_Y, CR7_Y; lower part). The orange line represents OpenFace (OF) measurement; the red line represents blenderFaceUV (bF) measurement.

Closer inspection of negative correlations for specific markers suggested that low to moderate correlations reflected either measurement noise when little or no movement occurred in both measurements or artifacts introduced by the OpenFace PDM. For example, contrary to the instructions, Participant 24 did not pose happiness but showed a neutral expression. The correlations for this emotion episode of Participant 24 ranged from *r* = *−*0.60 (BR2_Y/22_Y; Figure 9, upper part) to *r* = 0.76 (CR7_Y/54_Y; Figure 9, lower part). The peaks in the OpenFace measurement were caused by eye blinks (see Guo et al. 2018). These eye blinks led to artificial movement on the y‐axis of up to 3 mm for marker BR2/22 (inner eyebrows) and around 1.5 mm for marker CR7/54 (right mouth corner, see Figure 9; cf. Guo et al. 2018).

Tables 3, 4, 5, 6, 7 also show a low correlation between Marker A7_x and X_51. This likely reflects an additional artifact introduced by the PDM, because the x‐coordinate of OpenFace landmark 51 was interpolated as the midpoint between the two mouth corners. In contrast, the corresponding blenderFaceUV marker movement was measured directly.

Consequently, asymmetric mouth movements reduced the correspondence between the two measures.

**FIGURE 10:**
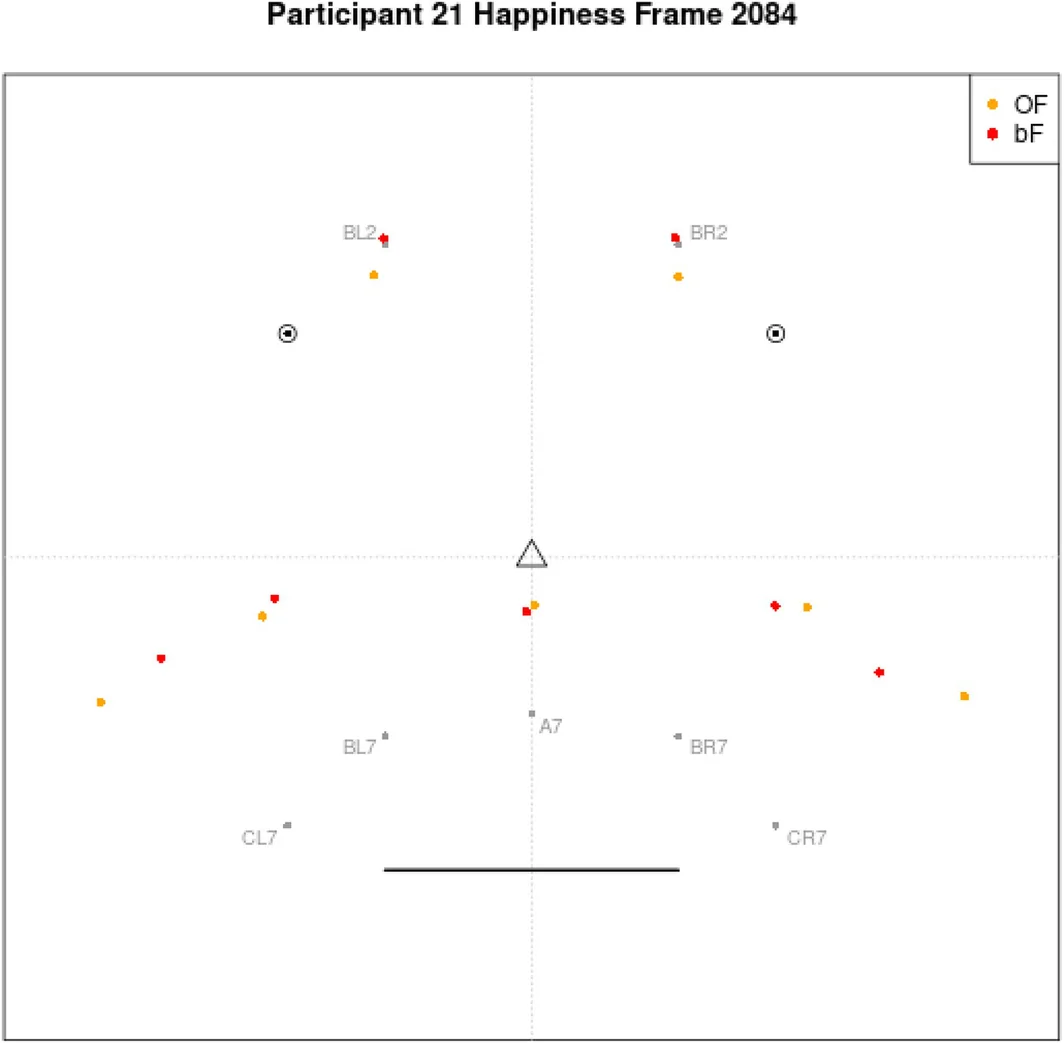
Standardized face plot for Participant 21 posing happiness at frame 2084. OpenFace markers are plotted in orange (OF), blenderFaceUV markers are plotted in red (bF). Origin of the movement is marked in gray with blenderFace labels.

To facilitate the identification of these artifacts and to provide a more comprehensive view of the temporal correspondence between the two measurement approaches, we visualized the x‐ and y‐coordinates of all markers for each frame, emotion, and participant on a standardized face in an animation. For each frame, we used the plotXhead() function from the blenderFace R package. To ensure that the marker trajectories remained within the plot boundaries, the coordinates from both measurements were scaled by a factor of 0.3. We then used FFmpeg to generate separate videos for each emotion and participant, thereby visualizing the progression of marker movements over time. These videos are available in the Supplement (Study 2, Videos, 02_OpenFace_blenderFaceUV_Comparison, OF_bF_Xheads). Figure 10 shows a still image of the video of Participant 21 posing happiness at frame 2084. Inspecting these videos allowed us to reveal the artifacts associated with the OpenFace PDM. Specifically, they show that the entire PDM appeared to deform during eye blinks and pronounced mouth movements (cf. Guo et al. 2018). This effect is illustrated in Figure 10, where the smiling movement of the mouth is accompanied by an apparent downward displacement of the inner eyebrow landmarks (BL2, BR2) in the OpenFace measurement. This effect likely results from the structure of the PDM, which represents facial shape through.

Interdependent rigid and nonrigid parameters. Consequently, OpenFace landmarks are not estimated independently; instead, the estimated position of each landmark can be influenced by movements in other facial regions. However, this effect is most evident in the temporal progression shown in the videos.

### 2.3 Discussion of Study 2

In Study 2, we evaluated the scalability of the blenderFaceUV measurement approach, compared the results with previous findings obtained using visible markers (Zinkernagel et al. 2019), and assessed their correspondence with measurements derived from OpenFace, a widely used AFER algorithm. Overall, we demonstrated the scalability of the blenderFaceUV method, although COVID‐19‐related contact restrictions resulted in substantial participant attrition. At both the descriptive and the aggregated level, data obtained using the blenderFaceUV measurement approach was also comparable to findings previously obtained with visible markers (see Zinkernagel et al. 2019). These findings suggest that the use of visually unobtrusive UV markers does not substantially alter the underlying measurement approach compared with the original blenderFace method. Thus, blenderFaceUV may provide a promising means of combining marker‐based precision with a visually unobtrusive recording setup. This may be particularly relevant for research contexts in which the measurement apparatus itself may influence behavior, such as research on implicit or explicit sociality (Fridlund 1991; Hess et al. 1995), facial mimicry (Hess and Fischer 2013, 2014), and the measurement of subtle or dynamic facial behavior (Krumhuber et al. 2023), where the visibility or weight of electrodes or markers on the skin may be a practical concern.

In particular, this issue is relevant to the psychological facial EMG literature, where methodological discussions have emphasized electrode placement and participant comfort (Wingenbach 2023), while more recent work has sought to develop increasingly conformal electrode designs for facial electrophysiological monitoring (e.g., Zhao et al. 2021).

Consistent with these concerns, recent comparisons of facial EMG electrode configurations suggest that larger and more adhesive electrodes may improve action‐unit recognition performance during repeated production of subtle and maximum‐intensity facial expressions (e.g., Küster et al. 2025, 2026).

To enable a fair comparison with OpenFace, as a prominent representative of AFER algorithms based on a PDM, we ensured that the blenderFaceUV markers did not interfere with OpenFace tracking. Participants were therefore recorded simultaneously with two cameras: a UV camera captured sunscreen‐based markers for the blenderFaceUV method, whereas a conventional webcam recorded the face in the visible spectrum for subsequent analysis with OpenFace. As in Study 1, we expected strong correlations between the x‐ and y‐coordinate movements measured by OpenFace and blenderFaceUV because both systems captured the same facial movements at the same time. Contrary to this expectation, the observed correlations were surprisingly low. However, their magnitude depended strongly on the amplitude of the movement: larger facial movements were associated with stronger correlations, whereas smaller movements were associated with weaker correspondence. For example, posed happiness and disgust produced comparatively large facial movements and correspondingly strong correlations, whereas posed sadness, anger, and fear involved smaller movements and showed weaker correspondence. This pattern was also evident for emotion‐specific markers and at the level of individual expressiveness. Posed happiness, for instance, produced strong correlations among mouth markers, whereas posed anger yielded stronger correlations among eyebrow markers. Visual inspection further indicated that participants 14 and 21 displayed very little facial movement, which was accompanied by weak correlations between the OpenFace and blenderFaceUV trajectories. When true movement is minimal or absent, the correlation is largely determined by measurement noise and may therefore be positive, close to zero, or even negative.

In addition to differences in movement amplitude, artifacts introduced by the OpenFace PDM further reduced the correlations and, in some cases, produced negative correlations. We identified two distinct types of such artifacts: (a) Interpolation of OpenFace markers: This artifact was particularly evident for the x‐axis of Marker 51, which had a very low correlation with its corresponding blenderFaceUV marker. This artifact is diagnostically relevant because the lateral and asymmetric movement of the center of the upper lip forms part of the repertoire of emotional expression (e.g., contempt). (b) Artifacts introduced by movements of distinct parts of the PDM, such as eye blinks (cf. Guo et al. 2018) and mouth movements: Each eye blink produces a vertical displacement of the entire PDM up to several millimeters. Our data further indicated that large upward movements of the mouth induced artificial downward displacements of the eyebrow landmarks. Overall, the blenderFaceUV–OpenFace comparison videos suggest that the PDM appears to smooth the estimated facial landmark trajectories over time. As a result, it appears to underestimate rapid and/or pronounced facial movements and may therefore be less suitable for capturing precise or subtle facial dynamics, for which direct marker‐based tracking offers a precise alternative.

## 3 General Discussion

The original version of the blenderFace method (Zinkernagel et al. 2019) requires visible markers to be tracked and analyzed. However, visible markers have several limitations in certain experimental settings. First, like facial EMG electrodes, visible markers may appear awkward in face‐to‐face interaction so that participants may alter their natural facial expression behavior. Indeed, this possibility has even been reported for research with facial EMG that does not require face‐to‐face interaction, and recent facial EMG guidelines place greater emphasis on the concern that participants may be afraid that electrodes might fall off or restrict their ease of movement (Wingenbach 2023). Thus, although acceptance of wearing facial electrodes is generally assumed to be high, awareness of their presence or perceived restrictions of natural movement may bias spontaneous social facial expressions (Wingenbach 2023).

Second, similar to habituation to wearing EMG electrodes, awareness of visible facial markers may decrease over time. However, the ecological validity of using painted visible markers in psychophysiological research remains limited, as visible markers could impede the performance of current AFER algorithms, which generally have not been trained with applied facial markers. Thus, while there is some initial evidence suggesting that some AFER algorithms may be relatively robust to such small artifacts and obscured areas (e.g., due to electrodes; Kappas et al. 2016), a large number of facial EMG electrodes may pose a substantial challenge for current facial image analysis methods (Büchner et al. 2025). The use of invisible markers could therefore provide a promising approach to “triangulate” and compare the performance of fine‐grained reference tracking across different AFER algorithms and measurement methods. For example, visually unobtrusive markers could allow for a well‐controlled comparison between landmark‐based tracking and facial EMG measures. Although this question was not addressed in the present studies, future work could leverage blenderFaceUV to perform this type of benchmarking.

More generally, blenderFaceUV could contribute methodologically to a wide range of research questions, from emotion research and social psychology (e.g., racial biases, see Vanman et al. 2004) to nonverbal communication, social cognition, and facial mimicry.

Here, blenderFaceUV could be particularly useful for research questions where it is important that participants behave as spontaneously as possible. Compared to camera‐based AFER approaches using PDMs, UV markers can be applied almost anywhere, including on top of makeup, on or around facial masks, or on virtually any part of the face that might be of particular interest to a given research question. Compared with facial EMG, blenderFaceUV involves a lighter‐weight setup and is visually unobtrusive. Thus, there is no cable pull, electrode weight, or friction on the skin. While direct comparisons with methods such as facial EMG are still needed, this inherent lack of friction and weight of UV markers could provide advantages for research involving very subtle movements. In addition, large numbers of UV markers could be applied at minimal additional cost in consumables. Here, facial EMG electrodes, consumables, and amplifiers still represent a substantial cost, and high‐resolution facial EMG recordings remain rare (Guntinas‐Lichius et al. 2023).

The results of the present work suggest that the blenderFaceUV toolkit is a viable extension of the original blenderFace toolkit that is capable of accurately tracking sunscreen‐based UV markers. As demonstrated by the results of both studies, the sunscreen markers were not visible in ordinary visible‐light recordings and therefore visually less conspicuous than the visible markers used in the original blenderFace method. The visible‐light camera recordings revealed only a slight overall blue tint that was not perceived as problematic. In contrast, as demonstrated by the UV camera recordings, the sunscreen markers were clearly visible and well‐defined in the UV spectrum. Furthermore, both marker‐tracking procedures showed high precision according to the available performance metrics, including comparable reprojection/solve errors. Thus, as demonstrated by both the pilot study and the aggregated results, the overall tracking precision of blenderFaceUV remained very high and in line with that of blenderFace.

Finally, we benchmarked the blenderFaceUV method against OpenFace, a representative automated facial expression recognition (AFER) system based on a point distribution model (PDM). OpenFace showed systematic discrepancies from the directly tracked UV‐marker trajectories under the present recording and preprocessing conditions. These results suggest that the OpenFace PDM‐based measurement may smooth facial movements over time and introduce artifacts into the resulting movement estimates.

Despite these encouraging results, some limitations and challenges remain. First, the application of “invisible” sunscreen markers requires practice and is presently still somewhat more error‐prone compared to visible markers, which can generally be applied very quickly with black fluid eyeliner. In the current work, these technical challenges still resulted in a relatively high proportion of data that had to be excluded. Here, more standardized handling and dosage of the sunscreen may help improve the procedure. Based on our experience, the UV camera can also be leveraged to provide real‐time feedback to the experimenter to ensure that they can see and correctly place the markers. Future work could aim to develop automatic overlays of the intended landmark mesh onto the video of the participant in real‐time, highlighting the markers already painted and those yet to be painted. Furthermore, UV cameras, especially lenses with UV transparency, are more expensive than a high‐quality visible‐light camera (≈$2000 to *−*$6000 in 2026). Although this tool could be a useful addition to the psychophysiological laboratory, it requires a one‐time investment that is currently still roughly comparable to the cost of working with amplifiers and electrodes to record facial electromyography or establishing a shielded lab for EMG analysis.

A further limitation of the present work is that we did not directly assess whether the participants experienced reduced self‐consciousness, perceived the setup as less intrusive, or behaved more naturally when wearing UV markers. Therefore, the present findings support only the more limited conclusion that the markers were not visibly discernible in ordinary visible‐light recordings and appeared visually unobtrusive under the tested conditions. Whether this results in reduced reactivity or more naturalistic behavior in dyadic and other social interaction settings remains an important question for future research. In particular, future work involving direct face‐to‐face interaction could compare sunscreen markers with facial EMG or other types of markers to assess the impact of these methodological choices on perceived intrusiveness, self‐consciousness, and behavioral outcomes.

In conclusion, we argue that blenderFaceUV, using sunscreen markers, represents a promising new tool to study facial expressions in the psychophysiological laboratory. In particular, invisible markers could provide an alternative for measuring facial activity in situations where visible recording apparatus may influence behavior. While emerging smart‐skin electrodes are becoming increasingly lightweight (e.g., X‐trodes Ltd. 2026), invisible painted markers have no perceptible weight and impose no physical resistance on facial movements. By comparison, the original blenderFace method is less suitable for these purposes, as black markers are clearly visible. Finally, future work could also leverage blenderFaceUV (a) to provide geometry‐based points of reference for comparisons between different types of AFER algorithms, or (b) as a basis for “triangulating” EMG measures and AFER‐based measurements. In the first case, invisible markers could provide interval‐scaled measurements of facial movements that are mathematically and geometrically interpretable and do not interfere with the performance of webcam‐based AFER algorithms. As illustrated in the present work for the PDM used by OpenFace 2.0, this approach may contribute to understanding and benchmarking the performance of different AFER algorithms. In the second case, invisible markers could provide a reference measurement for specific facial movements, while additional markers could be added on the electrodes themselves. Finally, future work with this approach in combination with facial electromyography research could thus help bridge the gap between landmark‐based measures of facial activity and the rich body of findings accumulated over decades of psychophysiological research on facial activity, AUs, and competing emotion theories.

## Author Contributions

**Axel Zinkernagel:** conceptualization, software, methodology, data curation, writing – original draft, investigation, formal analysis, supervision, visualization. **Dennis Küster:** writing – review and editing, supervision, methodology, validation. **Rainer W. Alexandrowicz:** conceptualization, writing – review and editing, supervision, methodology, formal analysis. **Manfred Schmitt:** funding acquisition, supervision. **Tanja Lischetzke:** supervision, formal analysis, resources.

## Funding

The contribution of Axel Zinkernagel to this research was supported by a research grant from the German Research Association (DFG) awarded to Manfred Schmitt (SCHM 1092/16‐1).

## Ethics Statement

This study was carried out in accordance with the principles of the Declaration of Helsinki.

## Consent

All participants gave their consent to participate and their consent to the recording and analysis of their video data. Because the study was exploratory and focused on descriptive outcomes, it was not preregistered.

## Conflicts of Interest

The authors declare no conflicts of interest.

## Supporting information


**Data S1:** psyp70394‐sup‐0001‐Supinfo.docx.

**Data S2:** psyp70394‐sup‐0002‐Supinfo1.pdf.

**Data S3:** psyp70394‐sup‐0003‐Supinfo2.zip.

**Data S4:** psyp70394‐sup‐0004‐Supinfo3.zip.

## Data Availability

Approval was granted within the specifications for the special permit for data collection during the COVID‐19 pandemic, taking into account the occupational health and safety requirements with regard to UV irradiation (see https://doi.org/10.17605/OSF.IO/XTJBG). Materials and analysis code are available on the OSF and GitHub: Instructions, scripts, and materials regarding the blenderFace toolkit are available at the OSF (https://doi.org/10.17605/OSF.IO/QYVWH) and at GitHub (https://github.com/axzinker/blenderFace). The datasets and the R scripts from the present studies are publicly available on the OSF (https://doi.org/10.17605/OSF.IO/XTJBG).

## Endnotes

^1^
Originally, blenderFace was referred to as a method. In the course of development, however, step‐by‐step instructions, an R package, and R and Python scripts were added, so that the term “toolkit” seems more appropriate.

^2^
The present blenderFace implementation uses the 68 marker positions shown in Figure 2a, but the procedure can be extended to include additional markers if higher spatial resolution is required.

^3^
In Study 1, two 100‐W UV‐LED spotlights were used. Based on UV‐LED intensity measurements, an exposure duration of 16 min at a distance of 2.5 m was considered harmless to the eyes (cf.Report_UV_LED.pdf in the Supplement). In Study 2, we used three Blacklight F15W T8 BL368 G14 UV tubes as the UV light source. These tubes had lower radiation intensity (15 W each), allowed for a more uniform and diffuse UV illumination of the face, and were therefore positioned closer to the participant (see Figure 1a).

^4^
As the participant was not a certified FACS encoder, some variance in execution may remain despite these instructions.

^5^

https://github.com/axzinker/blenderFace.

^6^
In fact, any facial movement would be sufficient to verify the consistency between visual and UV marker movement measurement.

^7^
The origin of blenderFace's coordinate system is the nose tip. Therefore, positive movements of left markers indicate movements toward the vertical mirror axis of the face.

^8^
For Participant 8, one marker was missing (see Figure 2a, BL7, the midpoint of the left upper lip), and this marker was therefore excluded from the analysis. Because all other 12 markers could be tracked, this participant was retained.

^9^
While these terms have been featured prominently in “basic” emotion theory (Ekman 1992) and the FACS (Ekman et al. 2002), we did not expect the resulting non‐expert expressions to be FACS‐accurate and do not make any emotion‐theoretical claims. In the following, prompt labels are retained only for illustrative grouping of expressions.

^10^
A video with a stepwise transformation of OpenFace coordinates into R coordinates is available in the Supplemental Material (OFcoord_transformations.mkv).

^11^
According to the Blender manual, a solve error below 0.3 pixels indicates an accurate reprojection. https://docs.blender.org/manual/en/latest/movie_clip/tracking/clip/editing/track.html.

^12^
The plot is based on data that were not mirrored along the x‐axis; therefore, corresponding movements of the left and right markers appear in opposite directions. This is reflected by the red lines (x‐axis), which show opposite directional changes in the left and right panels. In contrast, the orange lines (y‐axis) are unaffected by horizontal mirroring of the face and therefore exhibit similar, approximately parallel movement patterns.
