# Supplementary material for: blenderFaceUV: Toward Marker‐Based Precision and Flexibility in Facial Tracking With “Invisible” Markers
Source: Psychophysiology. 2026 Sep 21;63(9):e70394. doi: 10.1111/psyp.70394 (PMC13594373; doi:10.1111/psyp.70394)
Supplement: Supplementary file 1 [file PSYP-63-e70394-s003.pdf]

Bootstrapped Correlations for Study 1

Because the correlations are based on highly dependent observations arising from consecutive video frames, we additionally computed bootstrapped correlations for comparison with those reported in Table 1 of the manuscript. The bootstrap analysis used a sample size of $n = 25$, assuming a large effect ($\rho > .60$), a one-tailed test, $\alpha = .05$, and a power of $1 - \beta = .95$. For each correlation, 5,000 bootstrap samples were drawn using a fixed random seed of 123. The bootstrapped correlations closely matched the original estimates, with a maximum deviation of .02.

Table 1: Bootstrapped Correlations of the Visual and UV Marker Movement Measurement per Participant, Marker, and Axis

	Participant 1		Participant 2	
Marker	x-axis	y-axis	x-axis	y-axis
BL2	.99	.99	.89	.96
BR2	.98	.98	.86	.97
DL2	.99	.89	.97	.83
DR2	.99	.93	.85	.93
BL5	.97	.98	.92	.97
BR5	.97	.98	.93	.99
CL7	.99	.97	.96	.88
CR7	.96	.97	.84	.97
A7	.68	.94	.88	.98
A8	.92	.97	.87	.98

Note. Bootstrapped correlations for Participant 1 were based on 3,860 measurement occasions (frames), whereas those for Participant 2 were based on 4,665 measurement occasions (frames).
