# Supplementary material for: blenderFaceUV: Toward Marker‐Based Precision and Flexibility in Facial Tracking With “Invisible” Markers
Source: Psychophysiology. 2026 Sep 21;63(9):e70394. doi: 10.1111/psyp.70394 (PMC13594373; doi:10.1111/psyp.70394)
Supplement: Supplementary file 2 [file PSYP-63-e70394-s002.docx]

**Supplemental Material**

blenderFace and blenderFaceUV toolkit:

https://doi.org/[10.17605/OSF.IO/QYVWH](https://doi.org/10.17605/OSF.IO/QYVWH)

blenderFaceUV Study 1 and 2 data and code:

https://doi.org/[10.17605/OSF.IO/XTJBG](https://doi.org/10.17605/OSF.IO/XTJBG)
