# Supplementary material for: blenderFaceUV: Toward Marker‐Based Precision and Flexibility in Facial Tracking With “Invisible” Markers
Source: Psychophysiology. 2026 Sep 21;63(9):e70394. doi: 10.1111/psyp.70394 (PMC13594373; doi:10.1111/psyp.70394)
Supplement: Supplementary file 4 [file PSYP-63-e70394-s004.zip › Supplemental material/blenderFace_Vignette.pdf]

blenderFace

Axel Zinkernagel

2018-01-26

Contents

1 Workflow of Blender-data post processing with the blenderFace package **1**
1.1 Overview of the package functions . . . 2

2 Functions to process the raw data from Blender **2**
2.1 Concatenating Blender's raw data files into a single RData file with the function `concatBlenderFiles` . . . 2
2.2 Scale the Blender units to millimeters by using the function `bu2mm` . . . 3
2.3 Scale facial movements to a standardized face by using the function `face2stdFace` . . . 5
2.4 Scan the data for outliers with the `plotOutliers` function . . . 7
2.5 Center the start values of the markers per stimulus condition with the function `centerCond` . 8

3 Functions to visually represent data **10**
3.1 Plot the x- and y-axis movement per marker over the frames with the function `plotXYmmpf` . 10
3.2 Plot the movement of the markers on a standardized head with the function `plotXhead` . . . 13
3.3 Plot individual median movement per participant using the function `plotIndmm` . . . 16
3.4 Plot aggregated marker movement per stimulus condition using the function `plotMmpCond` . . 17

4 Functions to compute higher order variables **18**
4.1 Compute angle and distance per stimulus condition with the function `angleDist` . . . 18

1 Workflow of Blender-data post processing with the blenderFace package

To use the `blenderFace` package, it is assumed, that you have tracked facial movements for at least two participants, following the "Step__by__Step__Instructions.pdf." The output of this procedure is a csv file for each participant, which includes the x,y,z-axis movement of the tracked facial markers. In additionally, you need to mark the frames for which a stimulus was presented to the participants. This must be done for each participant (a.k.a. csv file) in a column, for example, labeled "Stimulustype," which marks the frames for which a stimulus was presented to the participant (e.g., "posing happiness" or viewing a specific emotion eliciting film clip). Unfortunately, there is no procedure or function that helps you with this step because it depends to a large extent on how you presented the stimuli and how you recorded the participant's video clips. If you know the start- and stop-frames of the presented stimuli, for example, because you recorded the computer screen via a mirror behind the participant, you can fill in the stimulustype columns by hand using a spreadsheet program. Make sure that each stimulus condition label occurs only once per subject.

The `blenderFace` package comes with raw-data sets from two participants (in fact, it is one participant who was recorded and tracked twice) for which the stimulustype column is already attached.

First, be sure you have installed and loaded the package:

```
install.packages("devtools")
library(devtools)
install_github("axzinker/blenderFace", built_vignettes = TRUE)
library(blenderFace)
```

1.1 Overview of the package functions

The functions of the `blenderFace` package can be divided into three types, which should subsequently be applied:

- Functions to process Blenders raw data:
  - `concatBlenderFiles`: concatenate Blender files into one large R Data-file
  - `bu2mm`: scale the Blender data into millimeters
  - `face2stdFace`: scale the Blender data into a standardized face
  - `plotOutlier`: scan the marker movement for potential outliers
  - `centerCond`: set the start values for the markers to x = 0, y = 0 at the beginning of each stimulus presentation
- Functions to visually represent the data:
  - `plotXhead`: plot the data on a standardized face
  - `plotIndmm`: plot the individual mean marker movement
  - `plotXYmmpf`: plot the X/Y marker movement per frame
  - `plotMmpCond`: plot the aggregated marker movement per stimulus condition
- Functions to compute higher order variables:
  - `angleDistance`: compute the angle and the distance of median marker movement

2 Functions to process the raw data from Blender

2.1 Concatenating Blender's raw data files into a single RData file with the function `concatBlenderFiles`

To perform the concatenation of Blenders csv output files, use the `concatBlenderFiles` function. If you have followed the "Step-by-Step" instructions, the subject number is part of the file name. However, the file name ends with "_Step_03" for each participant. Please rename the files so that the subject number is the last number before the file type ending (e.g., ".csv"). For example, if you have the file "Subject_39_Step_03.csv," rename it into "Subject_39.csv" and ignore the "_Step_03." The package includes two sample csv files in the `./inst/extdata` directory ("Subject_01.csv";"Subject_02.csv"). Although the `concatBlenderFiles` function does some basic input checks, be aware that this function writes on your hard disk and may change/overwrite/delete files if messed up input strings are given!

The path specifications are adapted for the package example. Please change the paths according to your needs.

```
inputdir <- paste(system.file(package = "blenderFace"),"/extdata/",sep="")
outputdir <- paste(system.file(package = "blenderFace"),"/data/",sep="")
filenames <- c("Subject_01.csv","Subject_02.csv")

# If all files in a directory should be processes, use:
# filenames <- list.files(inputdir, pattern = paste("[0-9]",".csv","$",sep=""))

concatBlenderFiles(dataFileNames = filenames, inputDirectory = inputdir,
                   colNameSubj = "", outputFilename = "rawdata.rda",
                   outputDirectory = outputdir, verbose = TRUE)
```

The console output of the `concatBlenderFiles` function for the two sample data files shows:

```
Step 1: Determine unique column names and number of rows of the files to be concatenated.
Reading 2 files:
Reading file Subject_01.csv (1/2)
  Adding 1714 rows to data frame of actually 0 rows.
```

```
Reading file Subject_02.csv (2/2)
  Adding 1692 rows to data frame of actually 1714 rows.

The final data frame will have 51 columns and 3406 rows.

These are the unique column names of all files to be concatenated. Check whether they are correct.
 [1] "A7_x"          "A7_y"          "A7_z"          "A8_x"          "A8_y"          "A8_z"
 [7] "BL2_x"         "BL2_y"         "BL2_z"         "BL4_x"         "BL4_y"         "BL4_z"
[13] "BL5_x"         "BL5_y"         "BL5_z"         "BL7_x"         "BL7_y"         "BL7_z"
[19] "BR2_x"         "BR2_y"         "BR2_z"         "BR4_x"         "BR4_y"         "BR4_z"
[25] "BR5_x"         "BR5_y"         "BR5_z"         "BR7_x"         "BR7_y"         "BR7_z"
[31] "CL4_x"         "CL4_y"         "CL4_z"         "CL7_x"         "CL7_y"         "CL7_z"
[37] "CR4_x"         "CR4_y"         "CR4_z"         "CR7_x"         "CR7_y"         "CR7_z"
[43] "DL2_x"         "DL2_y"         "DL2_z"         "DR2_x"         "DR2_y"         "DR2_z"
[49] "Frame"         "Stimulustype"
Abort Script? (Press 'y' to abort or any other key to continue)
?
Step 2: Concatenate files.

Preallocating data frame of a 51x3406 matrix.
Concatenating file Subject_01.csv (1/2)
Concatenating file Subject_02.csv (2/2)
Step 3: Savie output file (saving large data files takes some time).
```

As the main output of this function a file with the file name given in the `outputFilename` parameter is saved in the directory given as `inputDirectory`, or given in the `outputDirectory` parameter, if given. The data frame stored in this file contains the data of all concatenated input data files. The output file of the two sample subjects attached to the `blenderFace` package is also attached to this package and labeled `rawdata`.

The correct spelling of the columns (= tracked markers) of the input data files has already been completed by the `concatBlenderFiles` function. It is also important, to check the stimulus conditions for correctness. This can be done with standard R code:

```
table(rawdata$Stimulustype, rawdata$subject)
```

```
##
##                     1   2
##                   863 791
##   posed_disgust 215 240
##   posed_fear    235 220
##   posed_happy   215 200
##   posed_neutral 186 241
```

Because the labels used for the stimulus episodes are the same for the two participants (e.g., no misspellings), the `rawdata` file can be used for further scaling, testing, and statistical analyses.

2.2 Scale the Blender units to millimeters by using the function `bu2mm`

The movements of the facial markers (respectively the x,y, and z- coordinates) measured with the Blender procedure are scaled in Blender Units (BUs). One BU should roughly correspond to one meter in reality; however, Blender's scaling algorithm is fairly arbitrary. If you have followed the "Step-by-Step" instructions, you have also generated a file called "Blender_Scalingdata.csv," which contains the scaling parameters (e.g., the glue dot diameter). These parameters can be used to rescale the BUs into millimeters. In principle, the

rescaling is done by the rule of proportion:

$$\frac{\text{Diameter in Blender units}}{\text{Diameter in millimeters}} = \text{Factor to divide BU by, to obtain mm}$$

For example, a glue dot has a diameter of 8 mm and is measured in Blender with a diameter of 1 BU:

$$\frac{1 \text{ BU}}{8 \text{ mm}} = 0.125$$
$$\frac{2 \text{ BU}}{0.125} = 16 \text{ mm}$$

In the sample videos, "Subject_01.mp4" and "Subject_02.mp4" glue-dots with diameters of 8 mm were used. To rescale the `rawdata` data set, the glue dot diameter measurements in BU are needed for each participant. For the two example subjects, the file "Blender_Scalingdata.csv" is included in the package and should be loaded to gain access to the scaling parameters. Please note that the paths used in the example below are specified for the package data sets. Therefore, please adapt the paths that match your work environment accordingly.

```r
# Load the file "Blender_Scalingdata.csv"
scaledata <- read.csv(system.file("extdata", "Blender_Scalingdata.csv",
                                  package = "blenderFace"), header = TRUE, sep = ",")
# To ensure proper matching, make sure to have the data sorted by subjects
scaledata <- scaledata[with(scaledata, order(scaledata$subject)), ]
```

To scale BUs into millimeters, only the glue dot diameter measured in Blender is needed. The column name of this parameter is `GlueDotDiameter` and can be determined by:

```r
# Get the column names of the scaledata dataframe
names(scaledata)
```

```
## [1] "subject"
## [2] "GlueDotDiameter"
## [3] "PupilPupilDistance"
## [4] "MouthcornerMouthcornerDistance"
## [5] "LeftPupilLeftMouthcornerDistance"
## [6] "RightPupilRightMouthcornerDistance"
## [7] "Comment"
```

Subsequently, load the `rawdata` data file generated by the function `concatBlenderFiles`.

```r
# Load the file "rawdata"
data(rawdata, package = "blenderFace") # for the package example, please comment out
# load("path/to/your/directory/rawdata.rda") # uncomment and adapt to your work environment
# To ensure proper matching, make sure to have the data sorted by subjects
rawdata <- rawdata[with(rawdata, order(rawdata$subject)), ]
```

Not all columns of the `rawdata` data frame must be rescaled (e.g., the subject or the frames column). Therefore, the column names of the columns that should be rescaled must be passed to the function via the `colNames` parameter.

```r
# Determine the dataframe columns that should be scaled:
names(rawdata)
```

```
##  [1] "A7_x"          "A7_y"          "A7_z"          "A8_x"
##  [5] "A8_y"          "A8_z"          "BL2_x"         "BL2_y"
##  [9] "BL2_z"         "BL4_x"         "BL4_y"         "BL4_z"
## [13] "BL5_x"         "BL5_y"         "BL5_z"         "BL7_x"
```

```
## [17] "BL7_y"        "BL7_z"        "BR2_x"        "BR2_y"
## [21] "BR2_z"        "BR4_x"        "BR4_y"        "BR4_z"
## [25] "BR5_x"        "BR5_y"        "BR5_z"        "BR7_x"
## [29] "BR7_y"        "BR7_z"        "CL4_x"        "CL4_y"
## [33] "CL4_z"        "CL7_x"        "CL7_y"        "CL7_z"
## [37] "CR4_x"        "CR4_y"        "CR4_z"        "CR7_x"
## [41] "CR7_y"        "CR7_z"        "DL2_x"        "DL2_y"
## [45] "DL2_z"        "DR2_x"        "DR2_y"        "DR2_z"
## [49] "Frame"        "Stimulustype" "subject"
```

```
# -> Frame, Stimulustype and subject should not be scaled -> removed for variable colNames
colNames <- c("A7_x",  "A7_y",  "A7_z",  "A8_x",  "A8_y",  "A8_z",
              "BL2_x", "BL2_y", "BL2_z", "BL4_x", "BL4_y", "BL4_z",
              "BL5_x", "BL5_y", "BL5_z", "BL7_x", "BL7_y", "BL7_z",
              "BR2_x", "BR2_y", "BR2_z", "BR4_x", "BR4_y", "BR4_z",
              "BR5_x", "BR5_y", "BR5_z", "BR7_x", "BR7_y", "BR7_z",
              "CL4_x", "CL4_y", "CL4_z", "CL7_x", "CL7_y", "CL7_z",
              "CR4_x", "CR4_y", "CR4_z", "CR7_x", "CR7_y", "CR7_z",
              "DL2_x", "DL2_y", "DL2_z", "DR2_x", "DR2_y", "DR2_z")

# To ensure that you will not overwrite existing data, use a new data frame
# (dataSmm means data scaled in millimeters)
dataSmm <- bu2mm(data = rawdata, colNames = colNames, colNameSubj = "subject",
                 scaleFactor = scaledata$GlueDotDiameter, rwMeasure = 8, verbose = TRUE)
```

```
## Perform scaling to millimeters for Subject 1 of 2. Individual scale factor is 0.00541.
## Perform scaling to millimeters for Subject 2 of 2. Individual scale factor is 0.006371.
```

```
# You have the option to save your data at this stage of the analysis
save(dataSmm, file = "path/to/your/directory/dataSmm.rda")
```

The data frame `dataSmm`, which is included in the package, is scaled to millimeters. If this data frame is used in other functions, e.g., the median distance of a marker movement can be interpreted in millimeters.

2.3 Scale facial movements to a standardized face by using the function `face2stdFace`

However, it might not be useful to scale the movements to an absolute measure (e.g., millimeters). Instead, it might be better to standardize the movements in relation to a standardized face. Imagine that you want to compare the facial expressions of a child sample with the facial expressions of an adult sample. Because the head sizes of the children are smaller, the facial movements of the children are also smaller in comparison with the adult sample. Therefore, the standardization of different face sizes with respect to a standard face is important. The function `face2stdFace` performs the standardization of individual face sizes to a standard face. . In the standardized face the length and the height of the head each have a value of 1, whereas the left-pupil – right-pupil distance and the left-pupil – left-mouth-corner distance are scaled to 1/3rd of the face height and face width.

As individual measures of face width and face height, the individual left-pupil – right-pupil and the left-pupil – left-mouth-corner distances are used. If you have followed the "Step-by-Step" instructions, you have measured and saved these distances in the file "Blender_Scalingdata.csv." To have comparable measures for all subjects, the left-pupil – right-pupil distance and the left-pupil – left-mouth-corner distance are set to a proportion of 1/3rd to achieve a factor by which the x-axis and the y-axis must be divided to obtain a standardized scaling. In addition, if the left-mouth-corner – right-mouth-corner distance or the right-pupil – right-mouth-corner distance are given, the `face2stdFace` function allows the user to compute a mean of these distances for rescaling the x-axis and the y-axis to have a more reliable distance measure. However, in our experience, the

mouth corner distance is not as reliable a measure as the pupil distance is.

Standardizing the z-axis has not (yet) been implemented, mainly due to the lack of an appropriate facial distance measure for rescaling the z-axis. As a consequence, at the moment, no meaningful rescaling of the z-axis is possible. Therefore the z-axis is omitted from further analyses.

Similar to function `bu2mm`, the rescaling is done via the rule of proportion. For Subject 1 in the example data in the file "Blender_Scalingdata.csv," the left-pupil – right-pupil distance is measured as 0.3346 BU in Blender. This distance is set to a proportion of 1/3 to obtain a scaling factor for the x-axis.

$$\frac{0.3346 \text{ BU}}{0.\bar{3}} = 1.0038$$

as a scaling factor for the x-axis. The left-pupil – left-mouth-corner distance for Subject 1 is measured as 0.36611 BU. Therefore, the scaling factor for the y-axis is

$$\frac{0.36611 \text{ BU}}{0.\bar{3}} = 1.09833$$

If you have not yet done so, load the files `scaledata` and `rawdata` into the R environment:

```
# Load the file "Blender_Scalingdata.csv"
scaledata <- read.csv(system.file("extdata", "Blender_Scalingdata.csv",
                                  package = "blenderFace"), header = TRUE, sep = ",")
# Make sure to have the data sorted by subjects
scaledata <- scaledata[with(scaledata, order(scaledata$subject)), ]

# Load the file "rawdata"
data(rawdata, package = "blenderFace") # for the package example, please comment out
# load("path/to/your/directory/rawdata.rda") # uncomment and adapt to your work environment
# Make sure to have the data sorted by subjects
rawdata <- rawdata[with(rawdata, order(rawdata$subject)), ]
```

Subsequently, prepare the parameters and call the function `face2stdFace`:

```
# Get the column names of the scaledata dataframe
names(scaledata)
```

```
## [1] "subject"
## [2] "GlueDotDiameter"
## [3] "PupilPupilDistance"
## [4] "MouthcornerMouthcornerDistance"
## [5] "LeftPupilLeftMouthcornerDistance"
## [6] "RightPupilRightMouthcornerDistance"
## [7] "Comment"
```

```
# Determine the dataframe columns that should be scaled:
names(rawdata)
```

```
##  [1] "A7_x"         "A7_y"         "A7_z"         "A8_x"
##  [5] "A8_y"         "A8_z"         "BL2_x"        "BL2_y"
##  [9] "BL2_z"        "BL4_x"        "BL4_y"        "BL4_z"
## [13] "BL5_x"        "BL5_y"        "BL5_z"        "BL7_x"
## [17] "BL7_y"        "BL7_z"        "BR2_x"        "BR2_y"
## [21] "BR2_z"        "BR4_x"        "BR4_y"        "BR4_z"
## [25] "BR5_x"        "BR5_y"        "BR5_z"        "BR7_x"
## [29] "BR7_y"        "BR7_z"        "CL4_x"        "CL4_y"
## [33] "CL4_z"        "CL7_x"        "CL7_y"        "CL7_z"
## [37] "CR4_x"        "CR4_y"        "CR4_z"        "CR7_x"
```

```
## [41] "CR7_y"        "CR7_z"        "DL2_x"        "DL2_y"
## [45] "DL2_z"        "DR2_x"        "DR2_y"        "DR2_z"
## [49] "Frame"        "Stimulustype" "subject"
```

```
# Exclude the columns "Frame," "Stimulustype," "subject," and z-axis columns
colNames <- c("A7_x",  "A7_y",  "A8_x",  "A8_y",
              "BL2_x", "BL2_y", "BL4_x", "BL4_y",
              "BL5_x", "BL5_y", "BL7_x", "BL7_y",
              "BR2_x", "BR2_y", "BR4_x", "BR4_y",
              "BR5_x", "BR5_y", "BR7_x", "BR7_y",
              "CL4_x", "CL4_y", "CL7_x", "CL7_y",
              "CR4_x", "CR4_y", "CR7_x", "CR7_y",
              "DL2_x", "DL2_y", "DR2_x", "DR2_y")

# To ensure that you will not overwrite existing data, use a new data frame
# (dataStdF means data of standaradized faces)
dataStdF <- face2stdFace(data = rawdata, colNames = colNames, colNameSubj = "subject",
                         pupilDist = scaledata$PupilPupilDistance,
                         leftPMDist = scaledata$LeftPupilLeftMouthcornerDistance)
```

```
# You have the option to save your data at this stage of the analysis
save(dataStdF, file = "path/to/your/directory/dataStdF.rda")
```

2.4 Scan the data for outliers with the `plotOutliers` function

To ensure that the data is valid and does not contain any artifacts (e.g., tracks "dropping off" the facial surface; tracks jumping between two different positions, because the pattern matching algorithm very probable matchings) a function to plot potential outliers is provided. The principle of detecting an outlier is based on the maximal plausible movement from one frame to the next frame of the video footage. The fastest human movement is the eye blink, which takes about 300 - 400 ms (Robert A.Moses (Ed.), (1981). Adler's Physiology of the eye clinical application. Mosby, Chapter 1, p. 1-15), whereas the cornea of the eye has a diameter of 11.5 mm. The speed of the eye-blink movement can be therefore (11.5mm / 150 ms) = 0.0766 mm/ms. Based on the frame rate (fps), the maximal plausible movement from one frame to the next is computed, and taken as a cut off value. For example, a video clip recorded at 30 fps contains an image taken every 33 ms. Therefore the maximal distance from one frame to the next is 0.0766 mm/ms * 33 ms = 2.53 mm. Every larger distance is marked as an outlier. The function computes the 2D distance (Pythagoras) of a marker coordinates pair (x and y) of frame t and frame t + 1. Per default, any distance larger than 2.53 mm is marked as a potential outlier. This function needs data scaled to mm because the cut off value is scaled in mm. Therefore, use the function `bu2mm` beforehand, otherwise the outlier detection is not meaningful. If the function does not find any outliers, it prints the result (no outliers) together with the maximal movement at the corresponding frame:

```
plotOutliers(subset(dataSmm, subset = (dataSmm$subject == 1)), colNameFrames = "Frame",
             colNameData = c("DR2_x","DR2_y"), colNameCond = "Stimulustype")
```

```
## No outliers found. Maximal movement is 0.93 mm at Frame 995.
```

Because the example data set dataSmm does not contain any outliers, we have to create one:

```
outlierTest <- subset(dataSmm, subset = (dataSmm$subject == 1))
outlierTest[995,"DR2_x"] <- outlierTest[995,"DR2_x"] + 2
```

The function now detects one movement which is larger than the cut off value (2.53 mm). Because lots of outliers may be found, the function asks if the outliers should be plotted:

```
> plotOutliers(outlierTest, colNameFrames = "Frame", colNameData = c("DR2_x","DR2_y"),
               colNameCond = "Stimulustype")
1 possible outlier(s) found:
          Dist     Frames
[1,]   2.741625 996.000000
Plot each outlier? ('y', 'n', or 'c' to cancel)
(y,n,c)?
```

If the plotting is confirmed, the function plots for each potential outlier the movement of the centered marker coordinates (x as black and y as brown line), the Pythagorean distance from t to t + 1 (green line), the cut off value (horizontal red line), and marks the frame with the outlier (vertical blue line).

```
plotOutliers(outlierTest, colNameFrames = "Frame", colNameData = c("DR2_x","DR2_y"),
             colNameCond = "Stimulustype", title = "Subject 1, DR2")
```

If outliers are identified, the wrong movement has to be corrected in Blender. Subsequently, again the corrected data has to be exported and post processed by the blenderFace functions.

2.5 Center the start values of the markers per stimulus condition with the function `centerCond`

Because the markers painted on the participants' faces are not exactly at the same positions for all participants (e.g., because participants' faces differ in their shape and size, markers are drawn by different experimenters, etc.), it is meaningful to center the start values for the markers at x = 0, y = 0 and z = 0 at the beginning of each stimulus episode. This will allow users to aggregate facial movements over participants or compare the marker movements of participants for different stimulus episodes, because the movement always begins at the same origin. Otherwise, a very standardized, accurate, and time consuming marker drawing procedure would have been necessary. However, the extent and direction of the marker movements are not affected by the centering procedure. Technically, the function `centerCond` sets the first frame of each stimulus episode (e.g., the "posing happiness" episode in the "stimulustype" column of the example data frames) to x/y/z = 0 and subtracts the offset of the first frame from all remaining frames of this stimulus episode. Because the procedure is computationally very intensive for larger data sets (~ 100 participants, video clips ~ 10 minutes, several stimulus conditions), the function is parallelized and runs on $n-1$ CPU cores. Nevertheless, it may take time. Use the `verbose` option to get feedback on the progress of this function.

```
colNames <- c("A7_x",  "A7_y",  "A8_x",  "A8_y",
              "BL2_x", "BL2_y", "BL4_x", "BL4_y",
              "BL5_x", "BL5_y", "BL7_x", "BL7_y",
              "BR2_x", "BR2_y", "BR4_x", "BR4_y",
              "BR5_x", "BR5_y", "BR7_x", "BR7_y",
              "CL4_x", "CL4_y", "CL7_x", "CL7_y",
              "CR4_x", "CR4_y", "CR7_x", "CR7_y",
              "DL2_x", "DL2_y", "DR2_x", "DR2_y")
# To ensure that you will not overwrite existing data, use a new data frame
# (dataStdFCen means data of standardized faces, centered)
# (only 1 Core is used to follow CRAN and Travis prerequisites)
dataStdFCen <- centerCond(dataStdF, colNames = colNames, colNameSubj = "subject",
                          colNameFrames = "Frame", colNameCond = "Stimulustype",
                          maxCPUcores = 1, verbose = TRUE)
```

```
## Starting up CPU cluster: Using 1 CPU-cores.
## Step 1: Get condition start frames per subject.
## Step 2: Get offset values per condition per subject.
## Step 3: Subtract offset values per condition per subject.
```

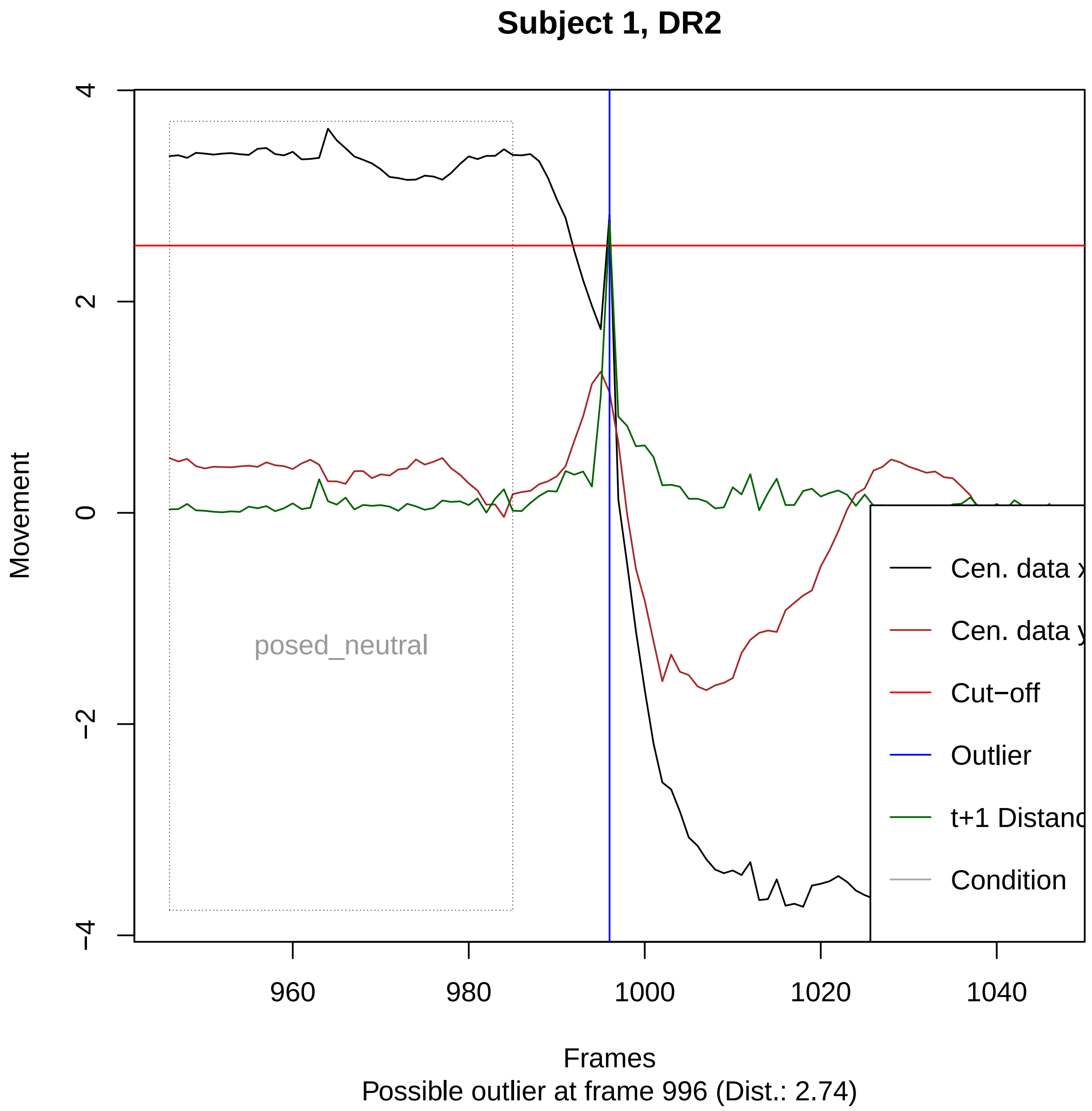

Figure 1: Outlier Plot

```
## Step 4: Replace centered values in the original data.
##
## Plausibility check: ColSums of centered start frames per condition should be 0:
##  A7_x  A7_y  A8_x  A8_y BL2_x BL2_y BL4_x BL4_y BL5_x BL5_y BL7_x BL7_y
##     0     0     0     0     0     0     0     0     0     0     0     0
## BR2_x BR2_y BR4_x BR4_y BR5_x BR5_y BR7_x BR7_y CL4_x CL4_y CL7_x CL7_y
##     0     0     0     0     0     0     0     0     0     0     0     0
## CR4_x CR4_y CR7_x CR7_y DL2_x DL2_y DR2_x DR2_y
##     0     0     0     0     0     0     0     0
##
## Time to complete each step of the function:
## Step1: Get condition start frames per subject:
## Time difference of 0.36 secs
## Step2: Get offset values per condition per subject:
## Time difference of 0.05 secs
## Step3: Subtract offset values per condition per subject:
## Time difference of 0.28 secs
## Step4: Replace centered values in the original data:
## Time difference of 0.02 secs
## Overall time:
## Time difference of 0.72 secs
## Shut down CPU cluster
```

```r
# You have the option to save your data at this stage of the  analysis
save(dataStdFCen, file = "path/to/your/directory/dataStdFCen.rda")
```

3 Functions to visually represent data

Since the data file can get quite large, graphical representations are a good and practical way to check the (processed) data for plausibility, outliers, artifacts, etc. The `blenderFace` package provides several functions to visually represent the data at different levels of aggregation.

3.1 Plot the x- and y-axis movement per marker over the frames with the function `plotXYmmpf`

This function plots the x-, and y-axis movement of a marker per frame for a single participant. The plot may be used to find artifacts (e.g., sliding of a tracker, scratching the facial skin and therefore moving markers, etc.) or to rescale plausibility and errors. The parameter set of this function differs from the parameter sets of the other functions because it is intended to be used in loops, for example, to generate many plots for the left and right markers of a subject and arrange them together in a pdf file.

The frames are presented on the x-axis and the marker movement is presented on the y-axis. The example below shows movement of BR4, the marker right of the nose, when looking at the participant's face, in millimeters for Subject 2 and marked with stimulus episodes:

```r
# Select data for Subject 2
# In addition, omit untracked frames at the start and the end of the video clip
data_Subj2 <- subset(dataSmm, subset = ((dataSmm$subject == 2) &
                                        (dataSmm$Frame >= 690)& (dataSmm$Frame <= 1610)))
plotXYmmpf(colFrames = data_Subj2$Frame, colX = data_Subj2$BR4_x,
           colY = data_Subj2$BR4_y, colCond = data_Subj2$Stimulustype,
           center = FALSE, title = "Subject 2, BR4")
```

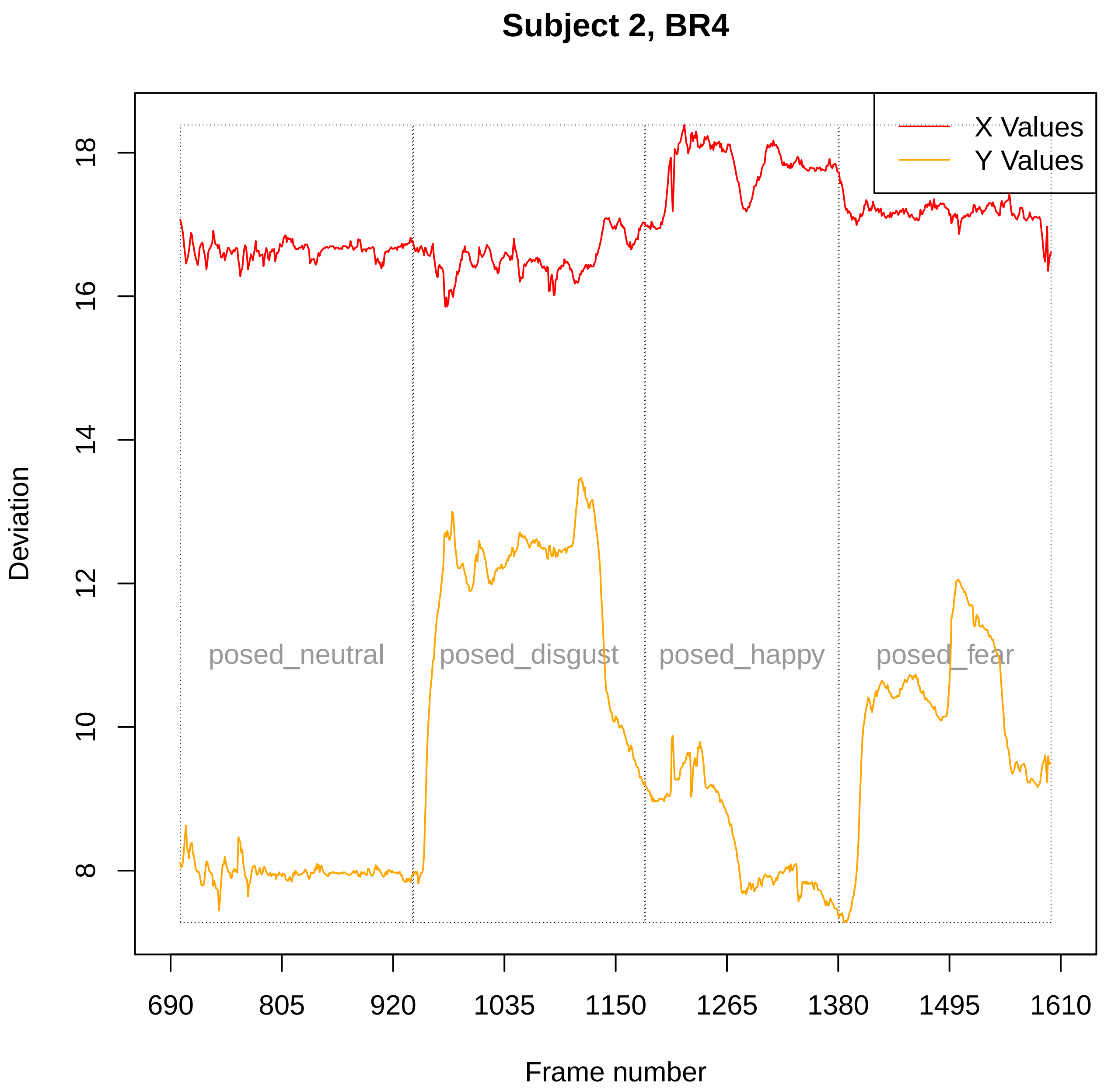

The next example allows the user to compare left and right movements (e.g., to judge the symmetry of the facial expression) by combining two plots with the `overplot` parameter. To achieve an appropriate scaling, also use the `center` switch. Note that for the left and the right markers the movement on the x-axis is pointed in the opposite direction.

```
# Plot right marker of BR4 (Centered)
plotXYmmpf(colFrames = data_Subj2$Frame, colX = data_Subj2$BR4_x,
           colY = data_Subj2$BR4_y, center = TRUE, title = "Subject 1,
           BR4 (red, orange), BL4 (blue, green)")
# Add left marker of BR4
plotXYmmpf(colFrames = data_Subj2$Frame, colX = data_Subj2$BL4_x,
           colY = data_Subj2$BL4_y, center = TRUE, color = c("blue", "green"),
           overplot = TRUE)
```

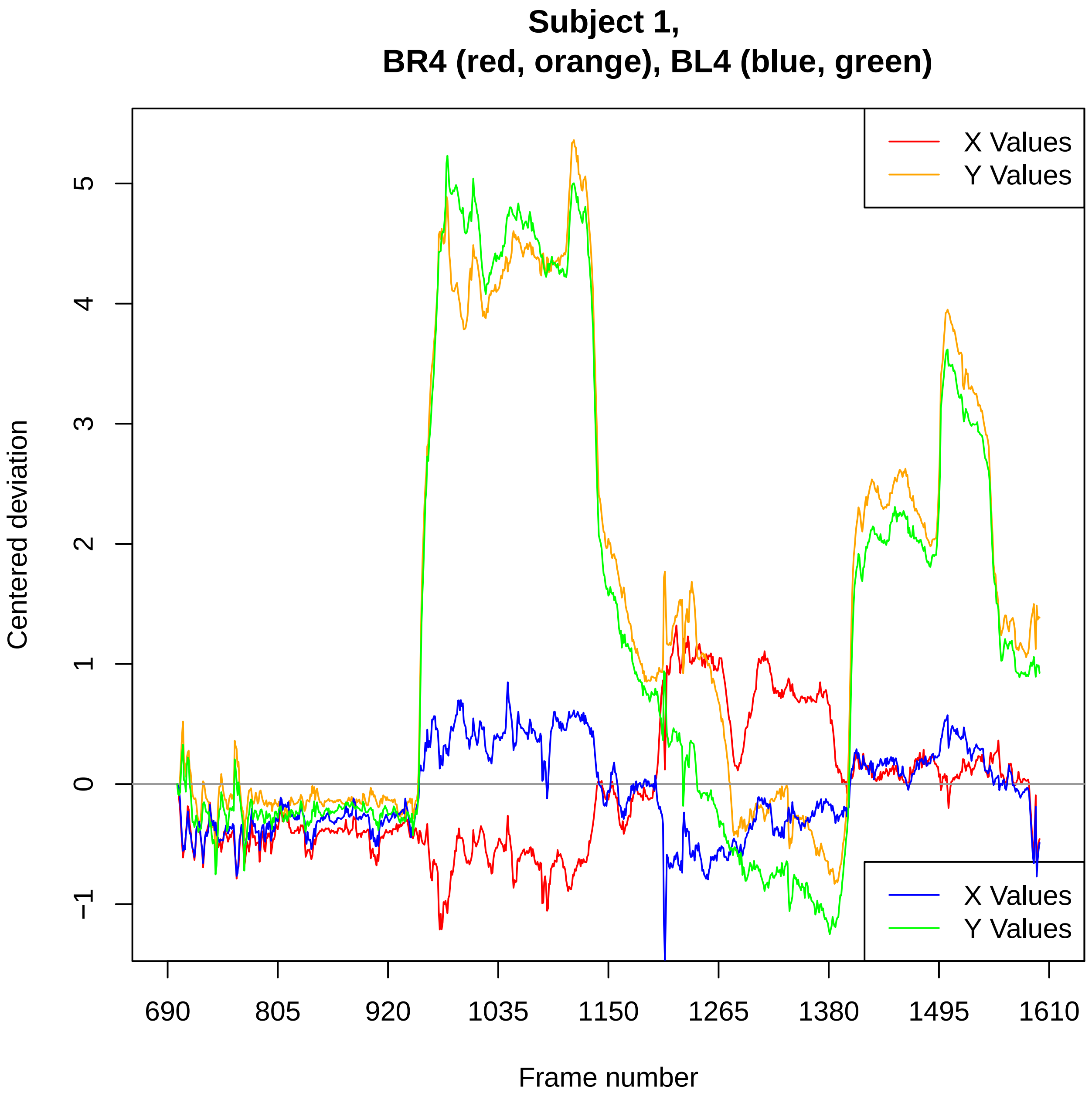

This function also allows the user to check whether centering the stimulus episodes via the `centerCond` function worked correctly:

```
data_Subj2Cen <- subset(dataStdFCen, subset = ((dataStdFCen$subject == 2) &
                                                (dataStdFCen$Frame >= 690)&
                                                (dataStdFCen$Frame <= 1610)))
plotXYmmpf(colFrames = data_Subj2Cen$Frame, colX = data_Subj2Cen$BR4_x,
           colY = data_Subj2Cen$BR4_y, colCond = data_Subj2Cen$Stimulustype,
           center = FALSE, title = "Subject 2, BR4")
```

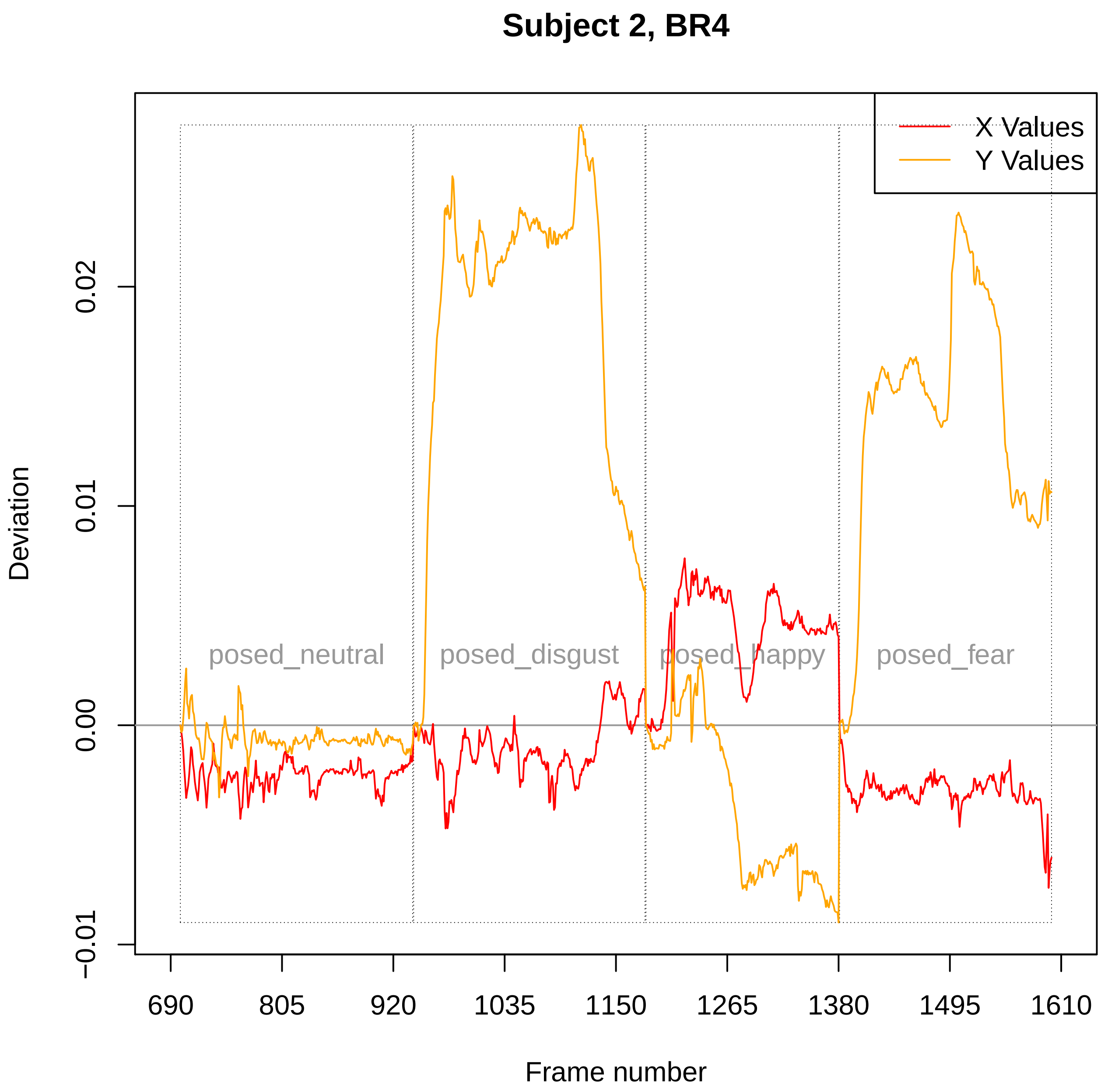

3.2 Plot the movement of the markers on a standardized head with the function `plotXhead`

This function plots the data of several facial markers for a single subject or for aggregated subjects on a standardized head model. To get meaningful and well-scaled plots, it is important to use the functions `face2stdFace` and `centerCond` first. Because the number and the position of markers are different for varying research questions, the starting positions of the markers have to be defined by the parameter `dataPos`. The names and the order of the variables in the `dataPos` list must be equal to the names and the order of the columns in the `data` data frame.

```
colNames <- c("A7_x",  "A7_y",  "A8_x",  "A8_y",
              "BL2_x", "BL2_y", "BL4_x", "BL4_y",
              "BL5_x", "BL5_y", "BL7_x", "BL7_y",
              "BR2_x", "BR2_y", "BR4_x", "BR4_y",
```

```
               "BR5_x", "BR5_y", "BR7_x", "BR7_y",
               "CL4_x", "CL4_y", "CL7_x", "CL7_y",
               "CR4_x", "CR4_y", "CR7_x", "CR7_y",
               "DL2_x", "DL2_y", "DR2_x", "DR2_y")

# Select data for plotting (selecting stimulus type and omit z-axis)
data_Subj_happy <- subset(dataStdFCen, subset = (dataStdFCen$Stimulustype == "posed_happy"),
                          select = c("subject",colNames))
data_Subj_disgust <- subset(dataStdFCen, subset = (dataStdFCen$Stimulustype == "posed_disgust"),
                            select = c("subject",colNames))

# Define the positions for the markers for the standardized face of x (-1,1)
# and y (-1,1) size as named list
dataPos <- list(BL2 = c(-.3,.7), BR2 = c(.3,.7),
                DL2 = c(-.7,.7), DR2 = c(.7,.7),
                BL4 = c(-.2,.2), BR4 = c(.2,.2),
                CL4 = c(-.5,.2), CR4 = c(.5,.2),
                BL5 = c(-.2,-.1), BR5 = c(.2,-.1),
                BL7 = c(-.2,-.6), BR7 = c(.2,-.6),
                CL7 = c(-.3,-.7), CR7 = c(.3,-.7),
                A7 = c(0,-.55),
                A8 = c(0,-.8))

# For debugging purposes the marker names and start positions may also be plotted
plotXhead(data = data_Subj_happy[-1], dataPos = dataPos,
          title = "All Subjects, happy", plotDataPos = TRUE)
```

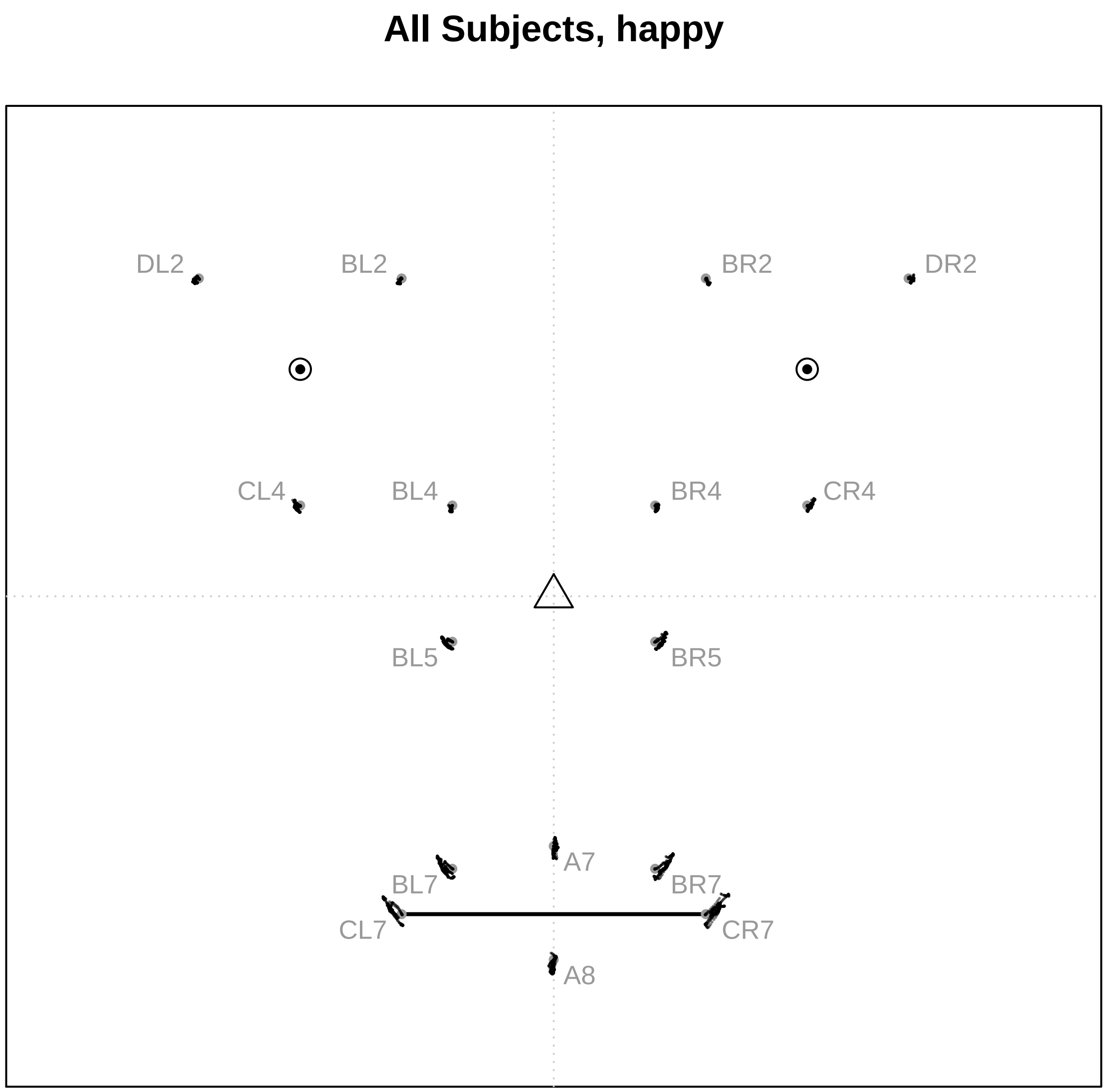

This function can also be used to compare facial movements in the sense of raw data plots via the `overplot` parameter:

```
plotXhead(data = data_Subj_happy[-1], dataPos = dataPos,
          title = "All Subjects, happy (black) vs. disgust (red)")
plotXhead(data = data_Subj_disgust[-1], dataPos = dataPos, overplot = TRUE, color = "red")
```

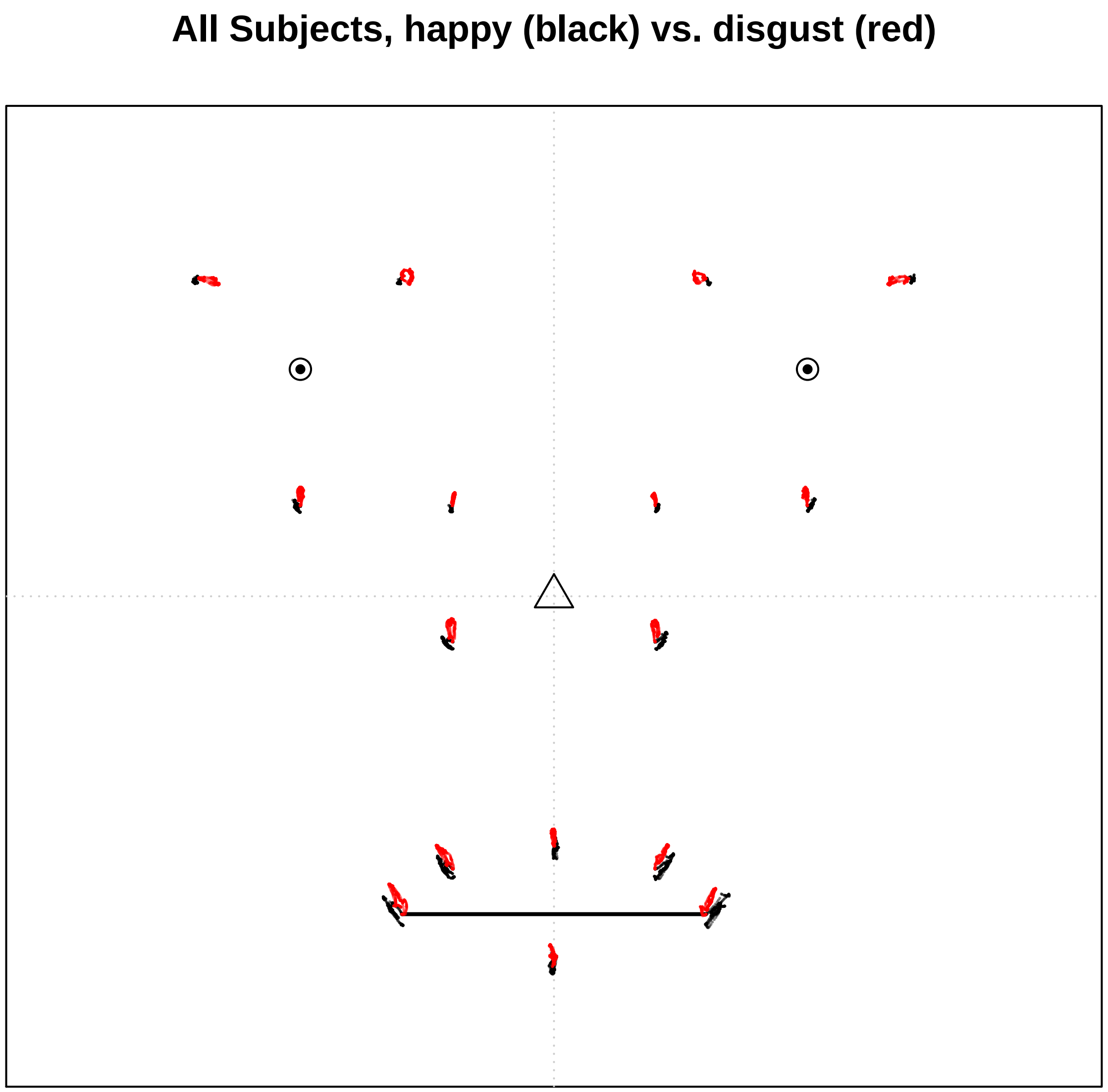

3.3 Plot individual median movement per participant using the function `plotIndmm`

The plots generated by the function `plotIndmm` may be used to detect individual outliers. For example, it is possible that some subjects will not follow the instructions to pose the facial expression disgust. On the basis of the plots, these individuals can be identified. Because the movement of the markers is most likely not normally distributed, the median of movement is computed and plotted. The subjects can be identified by the subject number plotted next to their median. To unify the scaling, use the `xlim` and `ylim` parameters. In the following example, the markers at the left and right mouth corners (CL7, CR7) were used.

```
plotIndmm(data = data_Subj_happy, colNames = c("CL7_x", "CL7_y"),
          colNameSubj = "subject", title = "Posed Happy CL7")
plotIndmm(data = data_Subj_happy, colNames = c("CR7_x", "CR7_y"),
          colNameSubj = "subject", title = "Posed Happy CR7",xlim = c(-.05,.05),
          ylim = c(-.05,.05), verbose = TRUE)
```

```
## Compute median for Subject 1
## Compute median for Subject 2
```

```
## Plot Subject 1
## Plot Subject 2
```

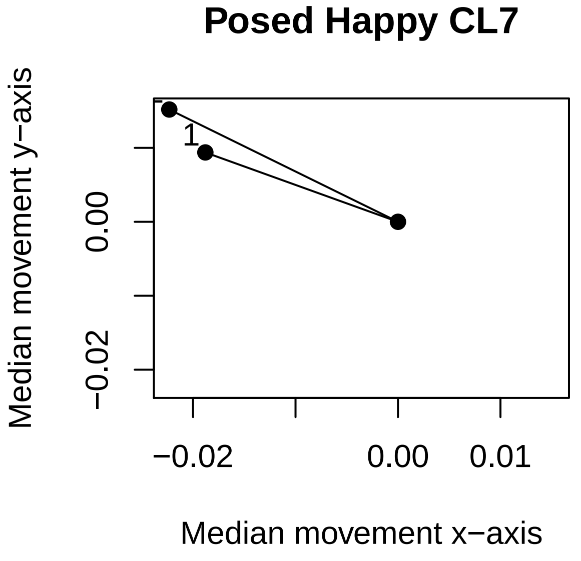

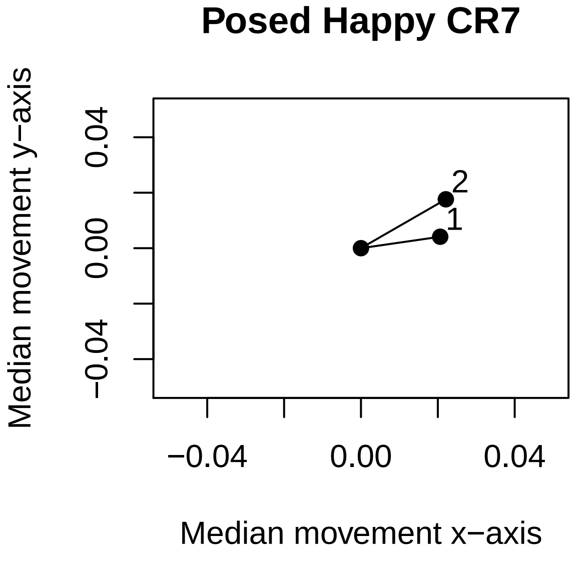

3.4 Plot aggregated marker movement per stimulus condition using the function `plotMmpCond`

This plot facilitates the comparison of the movement of a marker for different stimulus episodes. The function plots aggregated data of the median movement of one or more subjects per marker and per condition. In addition, distribution quartiles are plotted as ellipses around the median. The median and the quartiles are plotted in different colors for each stimulus episode. To achieve meaningful plots, make sure you have used the function `centerCond` first.

```
plotMmpCond(data = dataStdFCen, colNames = c("CL7_x", "CL7_y"),
            colNameCond = "Stimulustype", title = "CL7")
```

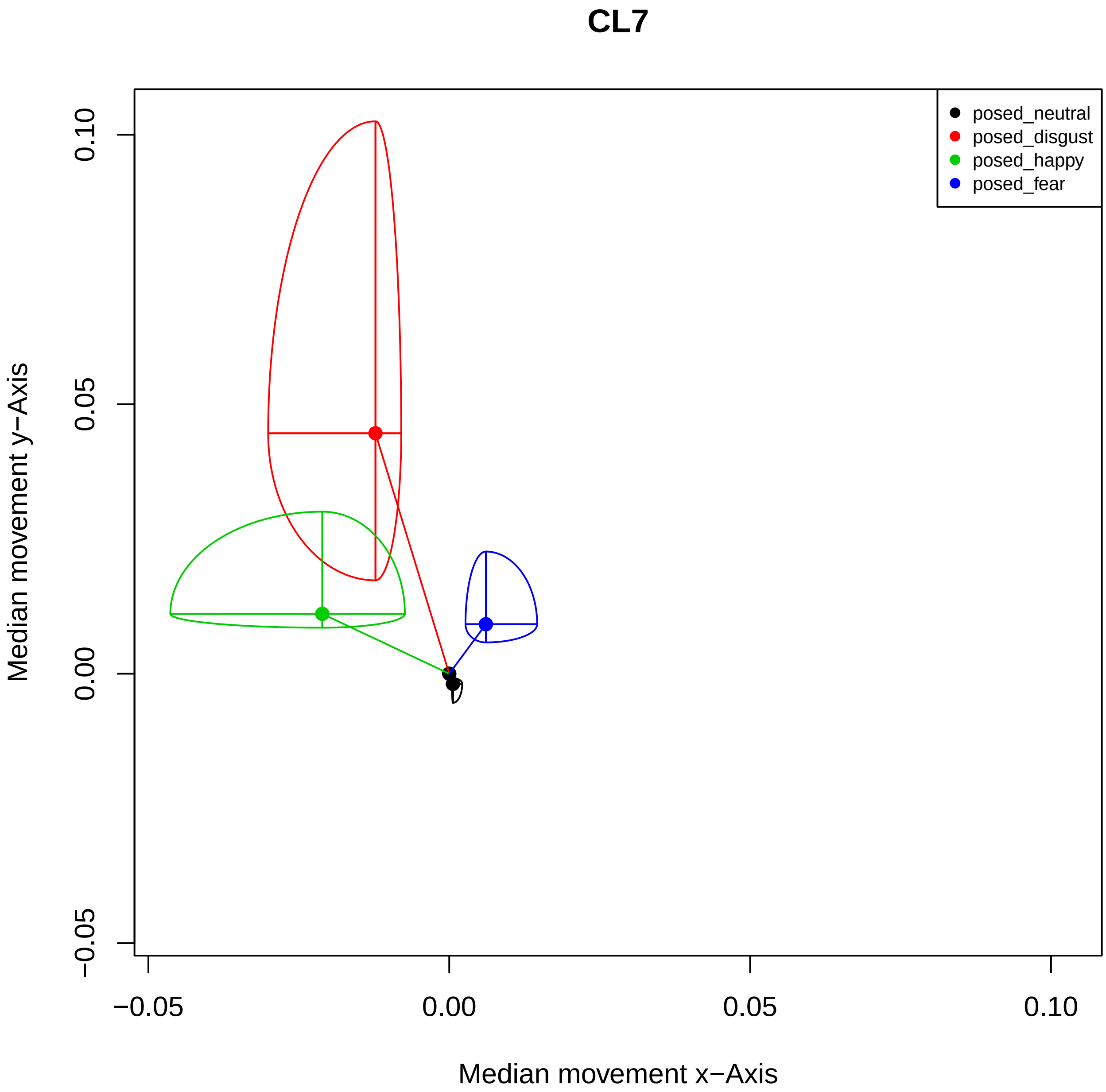

4 Functions to compute higher order variables

4.1 Compute angle and distance per stimulus condition with the function `angleDist`

This function computes the angle in degrees and the distance of a marker movement to facilitate the verbal description of the movement in the sense of the direction and extent of movement. It is assumed that the origin of the movement is at x = 0, y = 0, so be sure to have used the function `centerCond` before. Keep in mind that the angles are computed in degrees in the mathematical sense. This means that a movement to the right is around 0 or 360 degrees, a movement upwards is in the direction of 90 degrees, a movement to the left is around 180 degrees, and a movement downwards is around 270 degrees.

```r
# Data preparation
data_Subj_happy <- subset(dataSmm, subset = dataSmm["Stimulustype"] == "posed_happy",
                          select = c("subject",colNames))
data_Subj_disgust <- subset(dataSmm, subset = dataSmm["Stimulustype"] == "posed_disgust",
                            select = c("subject",colNames))

angleDist(data_Subj_happy, colNames = c("CL7_x", "CL7_y"),
          colNameSubj = "subject", rndDig = 3)
```

```
##   subject   angle distance
## 1       1 221.526   53.654
## 2       2 218.793   48.896
```

```r
angleDist(data_Subj_happy, colNames = c("CR7_x", "CR7_y"),
          colNameSubj = "subject", rndDig = 3, verbose = TRUE)
```

```
## Compute angle and distance for Subject 1
## Compute angle and distance for Subject 2
```

```
##   subject   angle distance
## 1       1 318.065   50.009
## 2       2 321.329   47.761
```

It also facilitates a comparison of the marker movements. For example, you could compare the movement of a posed happy facial expression and a posed disgust facial expression for the marker BL4.

Disgust stimulus episode:

subject	angle	distance
1	150.14	19.10
2	139.16	20.73

Happy stimulus episode:

subject	angle	distance
1	169.79	18.03
2	152.21	18.57

The angles suggest that the BL4 marker tends to move to the left (to 180 degrees) when happiness is posed and tends to move upwards (to 90 degrees) when disgust is posed. Because data scaled to mm was used (`dataSmm`) the distance represents the median movement in millimeters.
