# Supplementary material for: blenderFaceUV: Toward Marker‐Based Precision and Flexibility in Facial Tracking With “Invisible” Markers
Source: Psychophysiology. 2026 Sep 21;63(9):e70394. doi: 10.1111/psyp.70394 (PMC13594373; doi:10.1111/psyp.70394)
Supplement: Supplementary file 4 [file PSYP-63-e70394-s004.zip › Supplemental material/Blender_Stepwise_Instructions_V1.6.pdf]

Step-by-Step Instructions for the blenderFace method

21.02.2025
Version 1.6
For Blender version 2.80 and later

Axel Zinkernagel
University of Koblenz-Landau, Germany
zinkernagel@uni-landau.de
ORCID: 0000-0003-0404-1195

Rainer W. Alexandrowicz
Alps-Adria-University Klagenfurt, Austria
rainer.alexandrowicz@aau.at
ORCID: 0000-0001-9846-8936

These instructions belong to the supplemental material of the article:

Zinkernagel, A., Alexandrowicz, R. W., Lischetzke, T., & Schmitt, M. (2018). The blenderFace method: Video-based measurement of raw movement data during facial expressions of emotion using open-source software. *Behavior Research Methods*. https://doi.org/10.3758/s13428-018-1085-9. The supplemental material for this article is available at OSF (https://osf.io/qyvwh/).

We thank Dorothee Kohl-Dietrich and Dominik Vollbracht for testing and reviewing this instruction.

Contents

1 Method overview **3**

2 Requirements for an efficient tracking **3**
2.1 Recording requirements 3
2.2 Hardware requirements 4

3 A note on blenderFace marker labels and Action Unit labels **5**

4 A short introduction into Blender's user interface **6**
4.1 Conventions, abbreviations, tips & tricks 7
4.2 Blender's user interface 7
4.3 Initial Blender setup 11

5 Tracking and exporting facial movements **11**
5.1 Tracking static markers 11
5.2 Tracking facial markers and building the facial surface 14
5.3 Tracking emotion markers 16
5.4 Measurements for scaling blenderFace data 17

6 Data postprocessing with the R-package *blenderFace* **19**

7 Keyboard shortcuts **19**

8 Changelog **21**

List of Figures

1 Position of facial markers. 5
2 Labeling scheme of surface- and facial markers. 6
3 Blender's user interface. 8
4 Blender's editor types. 9

List of Tables

1 Keyboard shortcuts for the blenderFace method 21

1 Method overview

The aim of the blenderFace method is to 3D measure the video recorded movement of markers painted on a persons face and save it, so that the movement can be analyzed via statistical methods. To implement this, it is necessary to (a) control for head movements, and (b) to control for the individual facial surface. The blenderFace method relies on these requirements and can be divided into four consecutive steps:

1. *Static markers* on the headphones and a cap are tracked throughout the footage for which the facial movement of the markers should be measured, including a short parallax episode (see below) and the emotion relevant episode(s). These markers are needed to control for head movements.

2. Subsequently, *facial markers* are tracked for a short parallax episode. Based on the parallax between the tracked markers of two frames showing a person's face from two different perspectives (e.g., via head turns) the individual facial surface is computed.

3. In the following step, *emotion markers* are tracked for the duration of the emotionally relevant episode(s). The x, y, and z movement of these markers is the intended outcome of the method.

4. Finally, scaling measures are needed to convert the blenderFace measurement into real-world units (e.g., millimeter) or into a standardized face (i.e., left eye – right eye distance and left eye – left mouth corner distances are set equal for each participant).

In the demo-video *Step_by_Step_Instructions_280.mp4* the complete tracking procedure of the blenderFace method is shown. Time references to the demo-video are given in a box at the right margin of this manual.

2 Requirements for an efficient tracking

2.1 Recording requirements

To efficiently track the movement of the markers on the footage of a participant, some requirements have to be met:

- **Illumination:** The face of the participant on the footage should be well illuminated from all sides. That means that there should not be strong shadows (e.g., from the eyebrows or the nose). This can easily be achieved by a ring light, or by LED-stripes placed around the computer monitor, which is used to give instructions to the participant. Be aware that neon tubes and some LEDs flicker at 50/60 Hz, which may interfere with the sampling frequency of the camera and may lead to jitter when tracking the markers. Therefore, use stabilized constant light sources and switch off flickering lights. Be aware that the frame rate at which the camera records, depends on the illumination strength (e.g., at low light, the frame rate is reduced). Therefore, a constant illumination of sufficient strength to record at the desired frame rate should be used (e.g., control for illumination changes caused by sunlight on a partly cloudy day).

- **Camera:** Different web- and action-cams connected via USB to a computer have been successfully used. Recording of these cameras was performed via scripting of FFmpeg (https://ffmpeg.org/). The footage recorded by the camera should be at least at 1280 × 720 pixels (px) with 30 frames per second (fps). This offers a good balance between quality and file size of the footage, and also allows fast movements to be visible. However, this is a rule of thumb and higher/lower resolutions and other frame rates are also possible. Since Blender uses FFmpeg, all codecs FFmpeg supports (https://www.ffmpeg.org/general.html#Video-Codecs) can be used for recording the footage. To prevent tracking issues due to compression artifacts, uncompressed or lossless footage formats should be preferred. The synchronization of the footage recording and the stimulus presentation was performed via time stamp of the computer clock, which was burnt into the footage and recorded in the log file of the presentation software. In case the video recording and stimulus presentation were carried out on different computers, these were synchronized via network time protocol (NTP). In addition, a mirror in the back of the participants reflected the content of the computer screen, which was also recorded on the footage and served as a fallback.

- **Markers:** Blender uses a pattern-matching algorithm to track the movement of the markers on the frames of the footage. It is beneficial to have a high contrast between the marker and the background. The markers do not have to be placed or painted very accurately. It is more important that they display a uniquely identifiable pattern (cf. left part of Figure 1). For *static markers*, a minimum of 8 coloured glue dots are placed on black headphones and a black cap. These markers have to be static on the head throughout the footage (e.g., the headphones and the cap should *not* be moved) to be able to accurately track the head movements. 68 *Facial markers*, some of which also serve as *emotion markers*, should be painted by fluid eyelid liner on the facial skin. Care should be taken that the emotion markers are visible during emotionally relevant episode(s) and do not disappear in skin folds (e.g., when smiling). In the parallax episode (see below), the facial markers should also be static, which means that a neutral face must be shown by the participant during the parallax episode. In addition, all *facial markers* should be able to be connected into rectangles (cf. right part of Figure 1), which facilitates the construction, interpolation, and smoothing of the individual 3D facial surface. An emotionally relevant subset of the *facial markers* (for an example see left part of Figure 2) is reused as *emotion markers* and tracked during the emotionally relevant episode(s) of the footage. The movement of these markers is exported for later statistical analyses.

- **Parallax episode:** To allow the construction of the individual facial surface based on the 68 *facial markers*, a short episode (≈ 100 to 200 frames) with slight head movements is needed on the footage. For this short episode, we instructed the participants to show a neutral expression, face the camera with the nose tip and subsequently turn the nose tip pointing toward the middle of the right side of the computer monitor.

2.2 Hardware requirements

Basically, the blenderFace method can be performed on any computer with graphical display. However, because the pattern recognition of the tracking procedure is computationally intense, the recommended or optimal hardware as defined on the Blenders website (https://www.blender.org/download/requirements/) is endorsed. The tracking process can be performed in parallel, whereas multiprocessor systems are advantageous. Storing the video clips on a SSD accelerates

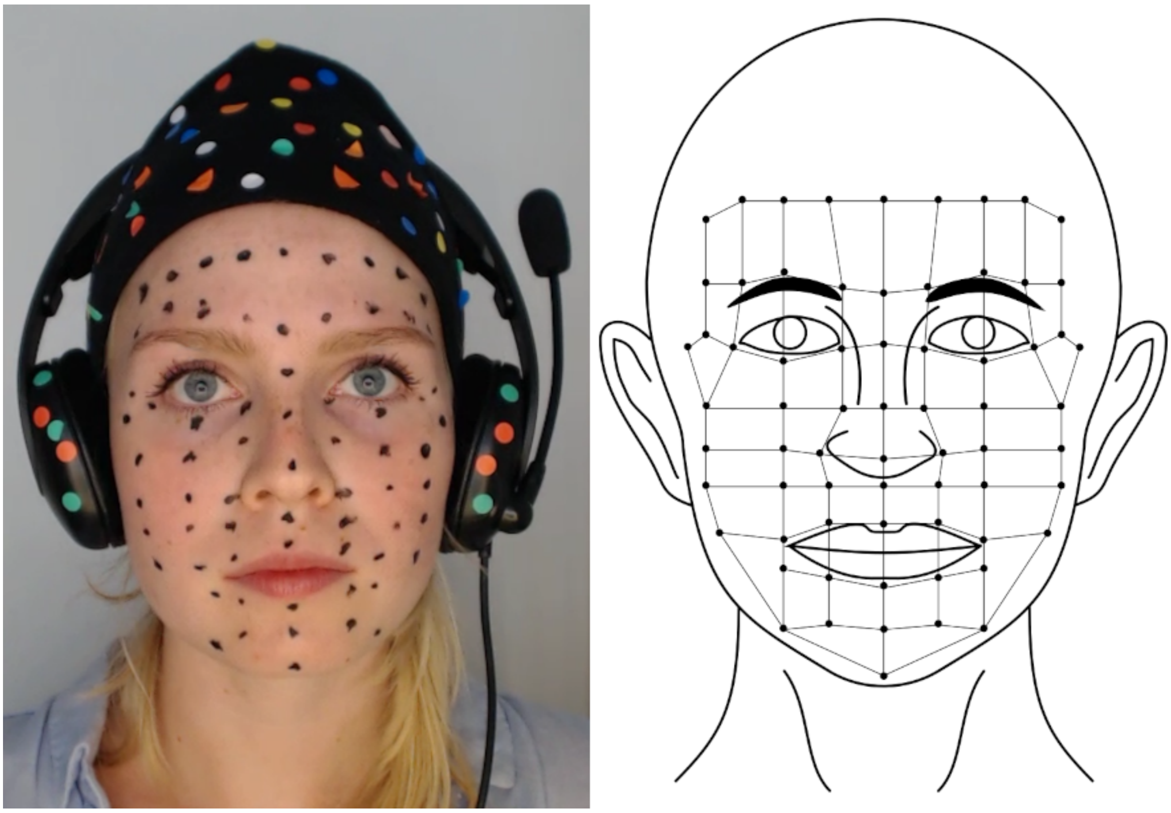

Figure 1: Position of facial markers.

the video clip processing speed considerably, compared to a hard disk. In terms of speed, it is also advantageous to have the complete footage in the computer memory (RAM), therefore, the advice is: the more, the better. For some computers/processors the graphic card as computing device is faster than having the computations performed on the processor. However, this may only be determined by performance tests. Finally, for interacting efficiently with Blender's user interface, a Full HD Monitor (1920 × 1080 px) and a 3-button-mouse with a mouse-wheel as middle button are recommended.

3 A note on blenderFace marker labels and Action Unit labels

A labeling scheme based on the FACS (Ekman & Friesen, 1978; Ekman et al., 2002) using Action Units (AUs) as marker labels is not particularly useful, because an AU defines the position of a marker on the face together with the direction of the movement. In contrast, the blenderFace method measures the visible movement of a marker drawn on the facial skin, which may move in virtually any direction. Therefore, the blenderFace method provides a straightforward labeling scheme defining only the position of the marker on the face (see left part of Figure 2): Letters define the x-axis position, whereas numbers define the y-axis position of a marker for the surface marker mesh. The face is divided vertically by the "A"-axis—going form the center of the forehead, via the tip of the nose to the chin—in two symmetrical parts, which were labeled as "left" and

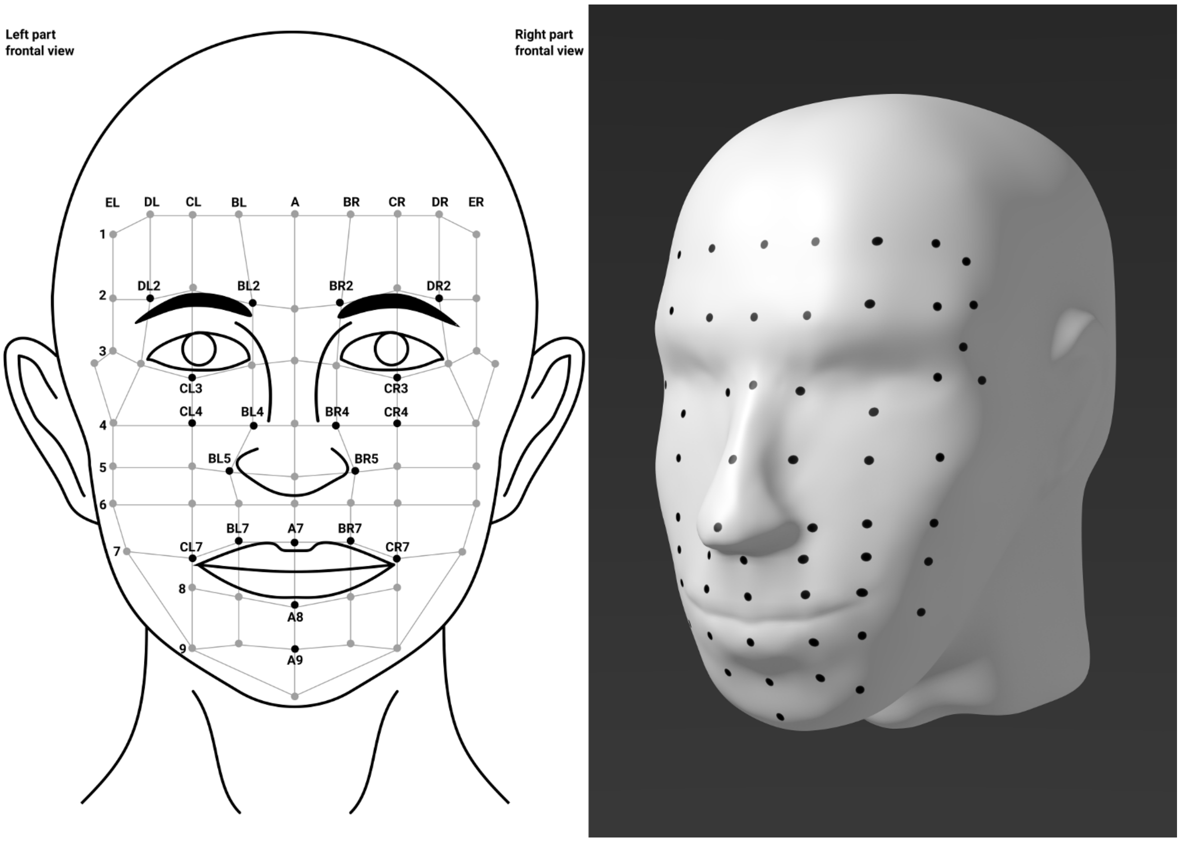

Figure 2: Labeling scheme of surface- and facial markers.

"right" part of the face. Left and right refers to your own perspective, not the perspective of the person on the footage. Starting from the "A"-axis, the subsequent vertical axes are labeled "B,C,D," and "E" in direction to the ears, combined with the label "L" for the left part, and "R" for the right part of the face. This labeling scheme facilitates the comparison of symmetrical markers. The horizontal axes are labeled starting from "1" at the upper forehead, via "5" intersecting the tip of the nose, to "10" at the bottom of the chin. For example, the marker "A5" denotes the tip of the nose, and "CL7" the left corner of the mouth.

In addition, the conversion of blenderFace marker labels into AUs is possible. For example, if "CL7" and "CR7" both move upwards, "CL7" to the left and "CR7" to the right, it represents the activation of left and right AU 12 (lip corner puller) which is part of a smile expression. Contrary, if "CL7" and "CR7" both move downwards ("CL7" to the left, "CR7" to the right), it represents the activation of left and right AU 15 (lip corner depressor) which is part of a sad expression. The distance of the blenderFace marker movement corresponds to the strength of the activation of the AU which is rated from A to E.

4 A short introduction into Blender's user interface

Blender (https://www.blender.org) is a professional open-source 3D computer graphics software intended to be used for animated films, rendering, visual effects, 3D printed models and video

games. It is licensed under the GNU GPL v2. These instructions refer to Blender user interface version 2.80 and later, the Blender version used in this step-by-step instruction is 2.93 LTS.

4.1 Conventions, abbreviations, tips & tricks

- Only the required functions of the blenderFace method are explained in this instruction. For further information, please refer to the online Blender Reference Manual (https://docs.blender.org/manual/en/latest/).
- UI means user interface, BU means Blender Units, the internal measurement unit in Blender (1 BU represents roughly 1 meter in the real world), AU means Action Unit as defined by Ekman and Friesen (1978) and Ekman et al. (2002).
- Important: The mouse pointer has to be within the area for which a keyboard command should be executed. Otherwise, it will have no -, or an unintended effect.
- In this instruction, labels of Blender's UI and special terms are printed in *slanted letters*, for example *Properties Editor* and *facial marker*.
- Keyboard commands and mouse buttons are framed by a box and printed in typewriter font. For example, pressing the `n` key toggles the *Properties Editor*.
- `LMB` means press the left mouse button, `RMB` press the right mouse button, and `MMB` means press the middle mouse button or move the mouse wheel upwards or downwards, respectively.
- `CTRL` means the left or right control-key, `ALT` means the left or right Alt/Alt Gr-key, `SHIFT` means the left or right shift-key, `ESC` means the escape-key, `DEL` means the delete-key, `SPACE` the space-bar, and `TAB` means the tabulator key. `←`, `↑`, `↓`, and `→` means the arrows next to the number pad.
- The `ESC` in Blender is generally a key that quits a tool-action. For example, if you press the `r` key to rotate an object, the degree of rotation is attached to the mouse so that the movement of the mouse changes the rotation. If you decide to not rotate, pressing the `ESC` key cancels the action and sets the object back to its initial degree of rotation. Also the tracking of a marker can be stopped by hitting `ESC`. The undo function is reached via the keyboard command `CTRL z`.
- References to the demo-video are given in a box at the right margin of the manual as `Start Min:Sec--Stop Min:Sec`. `01:22--01:47`
- Keyboard shortcuts for the blenderFace method are summarized in Section 7, p. 19.

4.2 Blender's user interface

Blender is a very powerful but complex software. However, in order to measure facial movements, only a small part of Blender's functionality is needed. When starting Blender, the default layout should look similar to Figure 3, after closing the splash screen by `LMB` clicking somewhere in the UI.

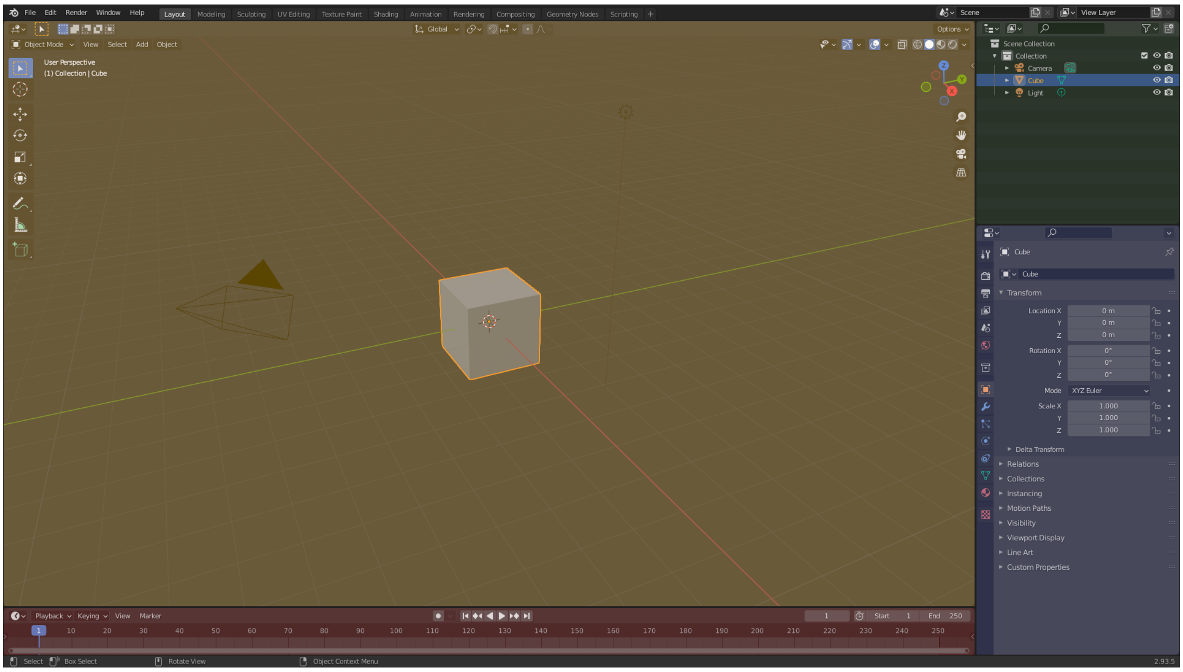

Figure 3: Blender's user interface.

The Blender window is divided into several rectangle *areas* which contain different *editors*. At startup, the *3D Viewport* editor (yellow), the *Ouliner* editor (green), the *Properties* editor (blue), and the *Timeline* editor (red) are shown.

Areas can be resized by LMB clicking and dragging its border. Each area can also be split into two areas with identical editor types by LMB dragging an area corner (the cursor becomes a "+") in the desired horizontal or vertical split direction. Areas can also be collapsed again by LMB dragging an area corner over the area which should be closed, which shows then a dark overlaid arrow. Areas can also be toggled to be window filling by pressing CTRL SPACE with the mouse within the desired area. Toggling back (CTRL SPACE) shows the previous arrangement of areas.

The *Editor Type* selector in the upper left corner of each area (marked red in Figure 4) opens a drop down menu which allows to select the editor type for this area. The editor type can also be changed via keyboard shortcuts (e.g., SHIFT F5 for the 3D Viewport) for the area within which the mouse is.

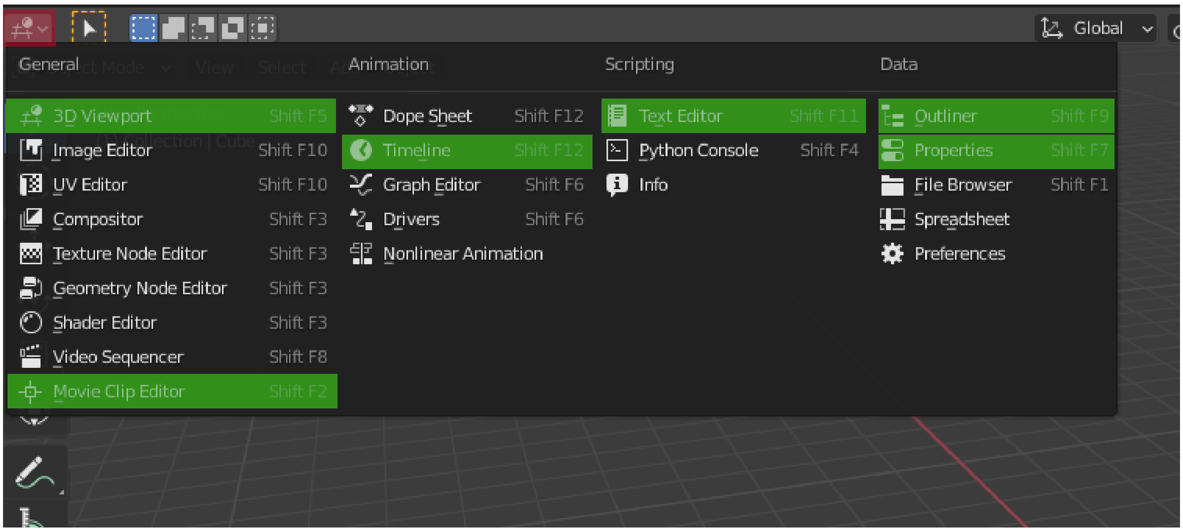

Figure 4: Blender's editor types.

Only a few of Blender's editors are needed for the blenderFace method, which are highlighted green in Figure 4:

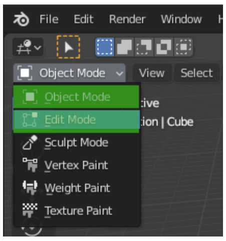

3D Viewport: In this editor the Blender's 3D scene is shown. In case of the blenderFace method, the individual 3D surface of a person and the 3D movements of the emotion markers are represented. The 3D coordinates of the emotion markers in this coordinate system are exported per frame for later analyses. This editor has also got several modes of which the *Object Mode* and the *Edit Mode* are relevant for the blenderFace method. Between these modes can be toggled with the TAB key.

The number pad is ideal for changing the views in the 3D Viewport (mouse within the 3D Viewport): 5 toggles between perspective and orthogonal view (should be orthogonal), 0 switches to a view through the camera (you should see the footage with Vertices over the facial markers), 1 switches to a view along the y-axis, 3 switches to a view along the x-axis, and 7 switches to a view along the z-axis. The view can also be changed with the mouse: MMB scroll zooms in and out in the 3D scene, MMB dragged while pressed rotates the view around the origin of the coordinate system, and SHIFT MMB dragged while pressed moves the view sideways. For further information about navigating in the 3D Viewport please refer to https://docs.blender.org/manual/en/latest/editors/3dview/navigate/index.html.

Movie Clip Editor: This editor allows to track markers in the footage, to re-attribute head movements into camera movements, to perform parallax computations for the individual facial surface, and to export the emotion marker movements into the 3D Viewport. This editor has several View types: In addition to the Clip view, in which the footage is shown, this editor also offers a Dopesheet view that shows the frames for which the markers were successfully tracked, and a Graph View, in which the x- (red) and y-axis (green) distance for the markers from one frame to the next are plotted.

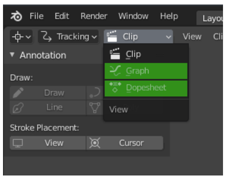

Timeline: This editor contains the footage player controls and allows to define the start and stop frames of the footage episode to be tracked. It also allows to scroll forward and backward in footage by `LMB` dragging the blue *Playhead* (active frame selector).

Text Editor: This editor allows to run scripts using Blender's python API. In the context of the blenderFace method it is used to export the 3D coordinates per frame of the emotion markers and save it as a CSV-file by a provided script.

Outliner: All elements of a Blender scene are shown in this editor. In the blenderFace method, these are the objects in the 3D Viewport (emotion markers, facial surface mesh, camera, light source, etc.). This editor allows to easily mark or delete elements in the 3D Viewport.

Properties: Finally, this editor allows to edit the properties of the objects in the Outliner. For example, the camera in the 3D Viewport can be constrained to follow the "inverted" head movements of a tracked person (i.e., whereas in reality the head moves in front of a static camera, in Blenders 3D Viewport the camera moves in front of a static head).

Some editors (e.g., the 3D Viewport and the Movie Clip Editor) provide *regions* within its area, that is *panels* with *tabs*, for example.

Toolbar: This region appears in the upper left of the editor's area and can be toggled with the `t` key. It contains some relevant tools for the editor, for example, setting the 3D cursor, moving or scaling an object, or measuring distances in the 3D Viewport editor.

Sidebar: This region appears in the upper right side of the editor's area and can be toggled with the `n` key. It is disabled per default. It shows settings of the active object or tool, sometimes divided into several tabs. For example, in the Movie Clip Editor, this region contains tabs for settings of footage properties (e.g., the path to the footage file) and track settings (e.g., Motion Model settings).

Adjust Last Operation: For some tools, this panel appears in the lower left corner of the editor's area. As the name implies, it allows the adjustment of the last operation performed with a tool and has to be unfold to access the parameter values. For example, the subdivision of a mesh (e.g., a facial mesh generated from the facial surface of a person) can be smoothed after subdividing by setting the Smoothness parameter to 1.

4.3 Initial Blender setup

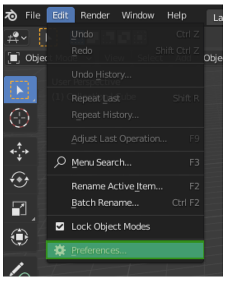

These settings need to be made only once in a freshly installed or updated Blender program. Open the *Preferences* by selecting *Edit* in the main top menu and LMB click on *Preferences*. In the newly opened window LMB select *System* in the left menu.

- Cycles Render Devices: Select the fastest compute device for tracking. "None" performs the tracking-computations on the CPU and is a good choice, whereas CUDA, OptiX and OpenCL use the computers graphic card chip for computations. There is no rule of thumb, it has to be tested which is the fastest device.

- Video Sequencer, Memory Cache Limit: For performance reasons it is advisable to allocate as much memory as possible to Blender. For example, if 32 GB RAM are available on the computer, 28 GB may be allocated to Blender, and 4 GB to the operating system. The *Memory Cache Limit* parameter has to be entered in MB which is then 1024*28 = 28672.

Subsequently LMB select *Add-ons* in the left menu and make sure to enable the Add-ons

- *3D View: MeasureIT*

- *Video Tools: Refine tracking solution*, and

by LMB click on the checkbox. The preferences are automatically saved when closing the window.

5 Tracking and exporting facial movements

Referring to Section 1 Method overview, in this section the steps of the method are described hands on in Blender.

5.1 Tracking static markers

The tracks of the static markers on the cap and the headphones (cf. left part of Figure 1) are 00:02 – 05:02
needed to disentangle the head movements from the facial movements: The head movement in front of a static camera is re-attributed in Blender to a camera movement in front of a static head in the 3D Viewport. Therefore, in the 3D Viewport the head is not moving and does not affect the measurement of movement of the emotional markers.

Beginning with a freshly started Blender (without splash screen):

1. **Switch to the Movie Clip Editor:** Switch the editor in the large main area (yellow in Figure 3) from the 3D Viewport to the Movie Clip Editor, either by LMB click on the Editor type selector in the upper left corner of this area and select the Movie Clip Editor, or by using SHIFT F2 with the mouse within this area.

2. **Open the footage:** Press ALT o, or LMB click on the *Open* button in the top center of the Movie Clip Editor area and select the footage file in the file selection dialog.

3. **Set the start- and stop-frames of the footage to be tracked:** LMB click on Set Scene Frames at the top of the left Toolbar. Press the p key to prefetch the footage into the RAM. With the mouse within the Timeline area at the bottom of the Blender window, MMB scroll down to zoom the Timeline, so that the beginning -, and the end frame of the footage is visible. LMB drag the blue Playhead to scroll through the footage. Eventually, adjust the start and end frames for which a tracking of static markers should be performed. The final start and end frames can be entered at the top right of the Timeline.

4. **Adjust the camera settings:** LMB click on Clip Display in the top right of the Movie Clip Editor area and enable the checkboxes Render Undistorted and Display Thin. In the right down Properties area (highlighted blue in Figure 3) select the Render Properties tab (second tab from the top, icon of a DSLR camera), open the Color Management dropdown menu and select Standard for View Transform.

   If the camera used to record the footage has a lens bias (e.g., a fisheye effect), this can be corrected in Blender: In the right Sidebar LMB click on the Track tab, drop down the Camera and Lens menu and enter the paramters of your camera. For further information please check https://docs.blender.org/manual/en/latest/movie_clip/tracking/clip/sidebar/track/camera.html.

5. **Adjust tracking settings:** Tracking settings can be made in two places: A template for all trackers can be adjusted in the left Toolbar, Track tab, Tracking Settings dropdown menu, and individual settings for each tracker can be adjusted in the right Sidebar, Track tab, Tracking Settings dropdown menu.

   First, adjust the template in the left Toolbar: Select Affine for Motion Model in the Tracking Settings dropdown menu, uncheck the Prepass box, and check the Normalize box. These settings allow a slower "conservative", but more stable and reliable tracking.

6. **Place tracker at the markers:** Go to the first frame of the frame range to be tracked by pressing SHIFT ←. Place the trackers on the markers of the cap and the headphones by CTRL LMB click on the markers on the footage. You can zoom into and out if the footage frame by scrolling the MMB, and you can MMB click and drag to move the footage frame. Place at least 8 trackers (better $\sim$ 12) on the cap and the headphones, and also make sure that these trackers are around the face and at different depth levels of the head. You can refine the placement of each tracker in the right Sidebar in the Track tab, Track drop down menu, where you can LMB drag the tracker detail more precisely. LMB clicking on this tracker detail also sets a new recognition pattern, which is important for later corrections of the track. Individual tracker properties can also be changed in the right Sidebar, Track tab, Tracking Settings dropdown menu. It is necessary to track a marker of known size (e.g., a glue dot on the headphones) which is visible frontally on a frame of the footage. This is needed later for rescaling the movement into real world measures (e.g., millimeter). 00:56 – 01:35

7. **Start tracking:** Select the 8 to $\sim$ 12 trackers by pressing a (a a quickly pressed deselects all trackers). All trackers should be highlighted. Start the tracking process by pressing CTRL-t. This function is also available via the Toolbar, Track tab, Track dropdown menu and the ↱ button in the Track line. If something goes fundamentally wrong, you can press ESC to interrupt the tracking process.

8. **If tracking fails:** Most probably, not all markers are tracked successfully till the end frame range. In order to detect, which markers failed, the Dopesheet view in combination with the Clip view is very useful. Move the mouse to the top right corner of the Movie Clip Editor area until the cursor becomes a "+" and LMB drag the corner down so that a new area (also containing the Movie Clip Editor) opens. Switch the upper area to the Dopesheet view by LMB click on the View type and select Dopesheet. Subsequently, in the Dopesheet view area MMB scroll down until the complete frame range with the trackers in the lines are visible. Move the mouse within the lower Movie Clip Editor in Clip view and deselect all marked trackers by pressing a a (the trackers are not highlighted now). In the upper Dopesheet view area LMB click on the tracker name on the left, which has stopped tracking (lines that end before the end of the frame range). This tracker becomes highlighted in the Dopesheet view and in the Clip view. LMB drag the blue Playhead in the Timeline to the end of successful tracking of this tracker. The following steps can be performed in the Clip view to re-track successfully (also in combination): (a) LMB click on the tracker detail in the right Sidebar to set a new recognition pattern (you may also correct the placement of the tracker onto the marker position), (b) change the size and rotation of the tracker by LMB dragging the Handle of the tracker (the bullet at the end of the line which protrudes from the tracker rectangle), (c) choose a different Motion Model (e.g., Location) in the individual Tracker settings in the right Sidebar, Track tab, Tracking Settings dropdown menu, (d) enable Prepass for a short range of frames, which can also be found in Sidebar tracking settings (be careful with this option as the tracker may "drift off"), (e) disable RGB colors by LMB clicking on the R, G, or B buttons below the Sidebar tracker detail window to enhance marker–background contrast. Subsequently, start tracking for this tracker again via CTRL-t. 01:39 – 03:29

   For difficult track ranges it is also possible to track frame by frame by pressing ALT →, or LMB click on the ↣ button in the left Toolbar, Track tab, Track dropdown menu, Track line. If successfully tracked to the end frame, deactivate the track by clicking on the tracker name in the Dopesheet view and repeat this procedure by LMB click on the next failed tracker until all tracks are successfully tracked.

   If a marker disappears on the footage (e.g., by a head turn) but reappears again in later frames of the footage, you can join the two trackers: Track the marker until it disappears. Use the Playhead to forward to the frame on which the marker is fully visible again. Use SRTG LMB to place a new tracker on that marker, SHIFT LMB click on the old tracker to select both, and press STRG j to join these two trackers. Alternatively, you can select both trackers and LMB click on Join Tracks in the left Toolbar, Track tab, Track dropdown menu, Merge line. There are also functions to track backwards, refine, delete, etc. tracks, please check https://docs.blender.org/manual/en/latest/movie_clip/tracking/clip/editing/track.html.

9. **Check finished tracks for outliers:** Once all markers have been successfully tracked, it is recommended to check the correctness of the movement. Switch the upper Movie Clip Editor area from Dopesheet view to Graph view. The Graph view shows the movement of the tracks along the x-axis (red) and the y-axis (green) from one frame to the next. `MMB` scroll into the Graph view and `MMB` drag the Graph view to find inhomogeneous track movements (e.g., spikes of a marker that are different from the other markers). `LMB` click on the spike (the track line in the Graph view and the tracker in the Clip view are highlighted) and `LMB` drag the Playhead in the Timeline to inspect what happened to the marker. In the case of a tracking error place the tracker to that position and retrack, if necessary.

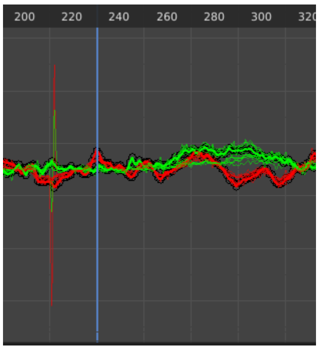

Save the status of Blender (preferably with subject number) at this stage of analysis by pressing `CTRL s`.

5.2 Tracking facial markers and building the facial surface

Next, the facial markers need to be tracked to build the individual facial surface of a person. The static markers on the head and the facial markers on the surfaces of a neutral face form a unit (as in reality, they should in Blender) and have to be tracked together. However, the facial markers need to be tracked only during the parallax episode of the footage, but will be reused as a static facial mesh throughout the head tracked footage (like the topology of the face on a moving head in reality). It is important that during the parallax episode no facial expression is shown, because moving markers will bias the estimation of the facial surface. 05:04 – 16:26

1. **Tracking facial markers:** `LMB` drag the blue Playhead in the Timeline to find a suitable start- and end-frame of the parallax episode. The parallax episode should be $\sim$ 100 to 200 frames long and contain a slight movement or turn of the head. Enter these start- and end-frame-numbers at the top right of the Timeline area. Press `SHIFT ←` to set the blue Playhead to the start of the parallax episode.

   Place trackers on the 68 facial markers (cf. Figure 1) according to step 6 in Section 5.1. 05:39 – 09:43
   Select the facial trackers (but not the static trackers, which should not be re-tracked) by pressing `b` (border select) and select all facial markers by `LMB` dragging a rectangle that contains these markers. Alternatively, you can deactivate any selection by pressing `a a`, `SHIFT LMB` select the 8 to 12 static markers, and invert the selection by pressing `CTRL i`. Subsequently track the facial markers and check for their correctness analogously following the steps 7 to 9 in Section 5.1. Please note that not all facial markers have to be tracked completely in this episode. Blender is also capable to estimate the 3D position of these markers from a subset of frames.

2. **Solving the camera motion:** Enter the start- and the stop-frames of the parallax episode as keyframe A and B in the left Toolbar, Solve tab, Solve dropdown menu of the lower Movie Clip Editor in Clip view. Subsequently, `LMB` click on the Solve Camera Motion button. After the computation has finished, in the upper right corner of the lower Clip view Movie Clip Editor a Solve Error is shown, which indicates the mean deviation in pixel of

the tracks on footage with the estimated 3D coordinates of the markers. This error should preferably be around 0.3.

If the solve error is substantially higher, (a) switch to the Graph view in the upper Movie Clip editor and inspect episodes with a raise in the blue line, which represents the average Solve Error. Identify and correct single trackers that cause a rise in Solve Error. (b) Enable the Info checkbox in the top right Clip Display settings of the lower Clip view Movie Clip Editor to show the Solve Error of individual trackers when selected. Inspect and correct trackers with a high Solve Error (disable this setting again if the display becomes too cluttered). (c) Solve the camera motion with two other keyframes A and B. (d) `LMB` click on the Refine button in the left Toolbar, Solve tab, Refine Solution dropdown menu (this button is only available if you have activated the Refine tracking solution Add-on in the Preferences). Be careful with this Button because it may bias the head track and facial surface. After corrections have been made, solve the camera motion again by `LMB` clicking the Solve Camera Motion button.

3. **Prepare the 3D Viewport:** To make use of Blender's 3D coordinate system for the coordinates of the exported emotion markers, the camera has to be placed to the correct position. In the lower area showing the Clip view of the Motion Clip Editor, switch to the 3D Viewport by pressing `SHIFT F5`. Press `x` to delete the (`LMB` selected) cube. Select the Camera by `LMB` click on it in the upper right Outliner area. Press `ALT g`, `ALT r`, and `ALT s` to place the camera to the origin of the coordinate system. With the camera selected in the Outliner, `LMB` switch to the Object Data Properties tab (green film camera icon) in the lower Properties editor area. `LMB` click on the checkbox Background Images to enable it, and also drop down the menu. `LMB` click on Add Image, `LMB` select Movie Clip and then Active Clip. `10:31 – 10:58`

4. **Switching head movements to camera movements:** The camera still selected, switch to the Object Constraint Properties tab (blue belt drive icon, above the green film camera icon) in the lower right Properties editor area, `LMB` click on Add Object Constraint and select Camera Solver. After adding the constraint, the camera has an offset to the origin of the coordinate system.

5. **Placing the nose tip to the center of the coordinate origin:** Switch from 3D Viewport to the Movie Clip Editor (`SHIFT F2`) in the lower area. Select all facial markers (`SHIFT LMB` select the static markers, and invert the selection by pressing `CTRL i`). In the left Toolbar of the lower Movie Clip Editor in Clip view `LMB` click on 3D Markers to Mesh button in the Solve tab, Geometry drop down menu. Switch back to the 3D Viewport (`SHIFT F5`) in the lower area. In the right upper Outliner area (`SHIFT LMB` select both, the Camera and the Tracks. `11:07 – 12:49`

   Press `1` on the number pad to switch to a view along the y-axis. Zoom (`MMB` scroll) and move (`SHIFT MMB` drag), so that you can see the Camera and the Tracks within the 3D Viewport. With the Camera and the Tracks selected, press `g z` (g stands for grab and z for the movement along the z-axis) and move the two objects upwards along the blue z-axis so that the nose tip vertex is on the red x-axis (zoom in to move more precisely). The objects are attached to the mouse, place them to the correct position with a `LMB` click. Press `g x` and move the nose tip vertex to the red-blue intersection of the x- and z-axis. Press

3 to switch to the view along the y-axis and move the nose tip vertex to the green-blue intersection of the y- and z-axis with g y. Finally press 0 to switch to Camera view and press r z to rotate along the z axis. Rotate until the y axis is in line with the A axis of the markers (cf. left part of Figure 2; marker line from forehead to chin). Press g to correct a possible offset of the nose tip marker and the x- y-axis intersection. Press SPACE to playback the parallax episode and check if the origin of the coordinate system stays on the top of the nose and correct the position, if necessary (stop playback by pressing SPACE again).

6. **Building the 3D surface of the face:** In the top right Outliner area, select only the Tracks object by LMB click on it. With the mouse within the 3D Viewport area, switch to Camera view via number pad 0, and press TAB to enter Edit mode. According to the right part of Figure 1, SHIFT LMB click on four vertices (points in the 3D space) to select them together and press f to build a *face*. Proceed until all sets of 4 vertices are combined into faces. Make sure to build rectangles to allow an easy subsetting without artifacts. If not all markers are visible / available, allow triangles only at the edges of the mesh. Check for plausibility of the facial surface by MMB pressing and dragging to rotate the facial mesh. If some vertices have an implausible z-value (e.g., due to solve problems in the parallax episode), select the vertex by LMB click on it (e.g., in the view along the x-axis, 3 on the number pad) and move the vertex to a plausible position by pressing g z and placing it to the new position with a LMB click. 12:54 – 16:25

   Select all vertices and faces of the facial surface by pressing b and dragging a selection rectangle around the facial surface. Press CTRL-e d (CTRL and e together, release, then d) to subdivide the facial mesh. Alternatively, LMB click on Edge in the top menu of the 3D Viewport and select Subdivide, or RMB click on the mesh and select Subdivide. In the lower left of the 3D Viewport area unfold the newly appeared Adjust Last Operation panel and set Smoothness to 1. Press CTRL-e d again to perform a second (smoothed) subdivision.

Save the status of Blender at this stage of analysis by pressing CTRL s.

5.3 Tracking emotion markers

Up to this point we have tracked the head movements, switched the head movements to camera movements, and built a mesh as a 3D surface of the person's face. In the following step, the *emotion markers* are tracked in order to export the movement for further statistical analyses. 16:27 – 22:29

1. **Select frame range of the emotionally relevant episode:** Switch from the 3D Viewport to the Movie Clip Editor (SHIFT F2). LMB drag the blue Playhead in the Timeline area to scroll through the footage to find start and end frames for the emotionally relevant episode. The frames of the emotionally relevant episode must be a subset of the static marker tracked footage (i.e., a head track must be available for the emotionally relevant episode). Enter the start and the end frame of the episode at the top right of the Timeline area. Press SHIFT ← to set the Playhead to the start of the emotionally relevant episode.

2. **Track emotional markers:** CTRL LMB click on the markers for which you want to export the movement (cf. Figure 2) in the lower Movie Clip Editor in Clip view. Which and how 15:55 – 20:33

many markers should be selected is a theoretical question and depends on the research interest. Assign a label for each tracker in the right Sidebar, Track tab, Track dropdown menu (Line above the tracker detail window). The labels should correspond to the labeling scheme in the left part of Figure 2 (e.g., rename Track.XXX into BL2 for the left inner eyebrow marker). These labels are reused as column names in the output CSV file. Follow the steps 6 to 8 in Section 5.1 to track the emotion markers. Subsequently, deselect all markers by pressing `a a`.

3. **Attach 3D objects to the movement of emotional markers:** Switch the upper Movie Clip Editor from Graph view into Dopesheet view. Select all emotion markers in the Dopesheet by `LMB` clicking on their names on the left. In the lower Movie Clip Editor in Clip view, Solve tab, Geometry dropdown menu, `LMB` click on the Link Empty to Track button. The empties (3D objects without a surface or volume) now appear in the upper right Outliner. The movement of the empties is attached to the movement of the markers, which is 2D on the footage. Therefore, we need to project the 2D movement on the facial 3D surface of the mesh generated in Section 5.2. To do this, select in the lower right Properties editor area the blue belt drive tab and `LMB` click on each emotion marker empty in the top right Outliner area. For each emotion marker empty select Tracks as a Depth Object in the Properties editor area. `20:35 – 22:07`

   Switch back the lower Movie Clip Editor in Clip view to the 3D Viewport editor (`SHIFT F5`). With the emotion marker empties selected in the Outliner press `RMB` in the 3D Viewport, and select Adjust Empty Display Size from the context menu. Drag the mouse to set the size of the empties to a reasonable value.

4. **Export the emotion marker movement:** Set the Playhead to the start of the emotionally relevant episode by pressing `CTRL ←`. `SHIFT LMB` select all emotional marker empties in the upper right Outliner area (only selected empties are exported). Switch the upper Movie Clip Editor in Dopesheet view to the Text Editor by pressing `SHIFT F11`. Open the export script *Export_Tracks_V1.23.py* by `LMB` clicking on the Open button and selecting it in the file chooser dialogue. Press `ALT p` to run the script. A file with the same filename as the Blender file, but with a csv ending is saved in the Blender file directory for further analyses. `22:10 – 22:29`

Save the status of Blender at this stage of analysis by pressing `CTRL s`.

5.4 Measurements for scaling blenderFace data

In the previous step we saved the movement data in BU, whereas 1 BU roughly represents 1 meter in reality. The blenderFace method allows for a more precise measurement by two scaling options, (a) scaling into millimeters (mm), and/or (b) scaling into a standardized face with a pupillary distance of 1, and a pupil-corner-to-the-mouth distance of 1. The parameters needed for scaling have to be measured in Blender, whereas the scaling itself is performed with the blenderFace R-package. In any case, the logic behind the scaling relies on the rule of proportion, for example, the proportion of a blender measurement and a known distance in the real world (e.g., a glue dot with a diameter of 8 mm). For the following steps, make sure that the MeasureIT add-on is activated (see Section 4.3). `22:30 – 25:18`

First, the procedure to measure the parameter to scale into mm is described below: `22:35 – 23:46`

1. Place the blue Playhead in the Timeline area to a frame in which a glue dot on the headphones is frontally visible (e.g., the start frame of the parallax episode).
2. Switch the 3D Viewport area to the Movie Clip Editor in Clip view (`SHIFT F2`). `LMB` select the tracker on that frontally visible marker and click 3D Markers to Mesh in the left Toolbar, Solve tab, Geometry drop down menu.
3. Switch the lower Movie Clip Editor area back to the 3D Viewport editor (`SHIFT F5`). Make sure you are in Camera view with the footage in the background (`0` on the numeric pad). Select the exported tracker by `LMB` click on it in the top right Outliner area (should be called "Tracks.001"). Zoom in onto the selected tracker (`MMB` scroll and `SHIFT MMB` drag).
4. In the 3D Viewport, `LMB` click on the Object menu in the upper left corner of this area and select Set Origin, and then Origin to Geometry. Press `SHIFT s` and select Cursor to Selected.
5. Add an empty by pressing `SHIFT a` and selecting Empty, Plain Axes. Press `s`, drag the mouse to scale the empty down to a reasonable size, and confirm with a `LMB` click. Press `g x` and move it to the left border of the glue dot. Add a second empty (`SHIFT a` and selecting Empty, Plain Axes), scale it down (`s`) and drag it to the right border of the glue dot (`g x`).
6. Measure the distance between these two empties by using the *MeasureIT* Add-on. Due to Blender updates, the procedure described here is more recent and different from demo-video. In the right Sidebar (`n`) `LMB`-click on the View-Tab and drop down the MeasureIT Tools entry. Activate the MeasureIT Add-on by `LMB`-click on Show. `LMB`-click on the left Empty and `SHIFT LMB`-click on the right Empty so that both Empties are activated. `LMB`-click on Link in the MeasureIT Tools entry. The value shown in blue above the distance line represents the value of an 8mm diameter glue dot measured in BU.

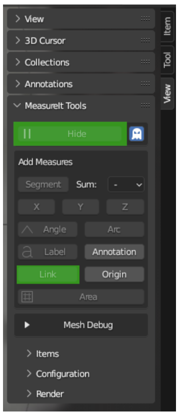

7. Save the value for this person in a separate spreadsheet table or R dataframe for later scaling procedures with the R package.

Second, the procedure to measure the parameters to scale into the standardized face (only do this if you need a standardized face) is explained, which is similar to the measurement above: `24:03 – 25:13`

1. Use the same frame as for scaling into mm, in which the person is directly looking into the camera (e.g., the start frame of the parallax episode).
2. In the 3D Viewport, toggle to Wireframe shading of the facial surface by pressing `SHIFT z`. Make sure you are in Camera view with the footage in the background (`0` on the numeric pad).
3. Zoom in onto the left eye (`MMB` scroll and `SHIFT MMB` drag) and place the 3D cursor over the center of the left eye (the persons right eye; `SHIFT-SPACE SPACE` (shift and

space together, then space again) to select the cursor and LMB click to place it). Press SHIFT a, select Empty, Plain Axes to add an empty at the cursor position. Ensure that the Empty is at the surface of the facial mesh by rotating the mesh to get a good view on that Empty and g z to move the Empty along the z-axis until it cuts the facial mesh.

4. Repeat the last step with the persons left mouth corner and the left eye.

5. With the MeasureIT Add-on activated (Show pressed, now showing Hide) LMB click on the empty of the left eye and SHIFT LMB click on the empty on the right eye. Repeat this measurement for the empty on the left eye and the left mouth corner. The measured left eye – rigth eye and left eye – left mouth corner distances are measured in BU.

6. Save these values for this person in a separate spreadsheet table or R dataframe for later scaling procedures with the R package.

Finally, save the status of Blender at this stage of analysis by pressing CTRL s.

6 Data postprocessing with the R-package *blenderFace*

The Blender software is used to record the emotional marker movement of a person and save it into a csv file. However, these are raw data that need to be postprocessed (e.g., scaled, visualized). This postprocessing can be performed with a package for the *R* language R-CoreTeam, 2021 called *blenderFace* (Zinkernagel & Alexandrowicz, 2016). The *blenderFace* package can be installed in *R* via GitHub:

```
install.packages("devtools")
library(devtools)
install_github("axzinker/blenderFace", built_vignettes = TRUE)
library(blenderFace)
```

The package includes functions for (a) attaching csv files of different participants to one large R-data file, (b) rescaling BUs into real-world measures (e.g., mm), (c) standardizing the face size, (d) computing several different diagrams to check plausibility and detect artefacts/outliers, and (e) computing mean distance and angle for a marker movement.

For more information on instructions of how to postprocess the data with the *blenderFace* package, please check the vignette in this package. The vignette is shown in *R* by executing:

```
browseVignettes("blenderFace")
```

7 Keyboard shortcuts

General shortcuts	
Mouse	Mouse pointer must be within the area for which an action is to be performed
ESC	Quit the current tool-action or computation
SPACE	Toggle playback of footage / animation
CTRL z	Undo the last step
SHIFT F2	Switch to Movie Clip Editor

SHIFT F5	Switch to 3D Viewport Editor
t	Toggle toolbar on the left side of the editor
n	Toggle sidebar on the right side of the editor
SHIFT LMB	Select multiple objects
b	Border select
a	Select all
a a (quickly)	Deselect all
LMB click in free space	Deselect all
x or DEL	Delete object or track
**Movie Clip Editor shortcuts**	
ALT o	Open video clip
CTRL t	Track active markers forward
SHIFT CTRL t	Track active markers backward
ALT →	Track single frame forward
ALT ←	Track single frame backward
ALT t	Delete track forward
SHIFT t	Delete track backward
CTRL j	Join two selected tracks
**3D Viewport shortcuts**	
5 (number pad)	Toggle orthogonal / perspective view
0 (number pad)	Camera view
1 (number pad)	View along the y-axis
3 (number pad)	View along the x-axis
7 (number pad)	View along the z-axis
TAB	Toggles between Object and Edit Mode
g	Grab object to move it
g x	Grab object to move it along the x-axis
g y	Grab object to move it along the y-axis
g z	Grab object to move it along the z-axis
r	Rotate object around the view axis
r x	Rotate object around the x-axis
r y	Rotate object around the y-axis
r z	Rotate object around the z-axis
ALT g	Sets location of selected object to 0,0,0 in the coordinate system
ALT r	Sets rotation of selected object to 0,0,0 in the coordinate system
ALT s	Sets scale of selected object to 0,0,0 in the coordinate system
CTRL-e d	Subdivide (CTRL and e together, then d)
SHIFT s	Scale a selected object
SHIFT a	Add an object to the 3D Viewport
SHIFT z	Toggle wireframe / solid shading of a mesh
**Timeline shortcuts**	
SHIFT ←	Set the Playhead to the start of the footage frame range
SHIFT →	Set the Playhead to the end of the footage frame range
SPACE	Toggle playing the footage / animation

Table 1: Keyboard shortcuts for the blenderFace method

8 Changelog

- Ver. 1.6: Use the *MeasureIT* Add-on for measuring distances needed for scaling blenderFace data in Section 5.4.
- Ver. 1.5: Adapt the procedure to the GUI of Blender 2.80 and above
- Ver. 1.0: Initial release

References

Ekman, P., & Friesen, W. (1978). *Facial action coding system: A technique for the measurement of facial movement*. Consulting Psychologists Press.

Ekman, P., Friesen, W., & Hager, J. (2002). *Facial action coding system. The manual on CD-Rom.* Network Information Research Corporation. Salt Lake City.

R-CoreTeam. (2021). *R: A language and environment for statistical computing*. R Foundation for Statistical Computing. Vienna, Austria.
