# Supplementary material for: blenderFaceUV: Toward Marker‐Based Precision and Flexibility in Facial Tracking With “Invisible” Markers
Source: Psychophysiology. 2026 Sep 21;63(9):e70394. doi: 10.1111/psyp.70394 (PMC13594373; doi:10.1111/psyp.70394)
Supplement: Supplementary file 3 [file PSYP-63-e70394-s001.zip › Approval_Ethics_Covid-19_Occupational_health_and_safety/2020-11-24 Basis GB UV Zinkernagel.pdf]

Vorlage zur Gefährdungsbeurteilung gemäß § 5 ArbSchG

Schutzziel gemäß dem Gesetz über die Durchführung von Maßnahmen des Arbeitsschutzes zur Verbesserung der Sicherheit und des Gesundheitsschutzes der Beschäftigten bei der Arbeit (Arbeitsschutzgesetz – ArbSchG):

Sicherheit und Gesundheitsschutz der Beschäftigten bei der Arbeit sichern und verbessern.

Übergeordnete Maßnahmenziele:

- Verhütung von Unfällen
- Verhütung von arbeitsbedingten Gesundheitsgefahren
- Menschengerechte Gestaltung der Arbeit

Zentrales Element des Arbeitsschutzes ist der Prozesse „Gefährdungsbeurteilung“. Dieser Prozess ist fortlaufend, um eine stetige Verbesserung von Sicherheit und Gesundheitsschutz der Beschäftigten anzustreben.

Mitwirkungspflichten liegen bei allen Beschäftigten. Die Maßnahmen sind bei allen Tätigkeiten und eingebunden in die betrieblichen Führungsstrukturen zu beachten.

☐ **Neufassung der Gefährdungsbeurteilung**	**X Aktualisierung der Gefährdungsbeurteilung**
Klicken Sie hier, um ein Datum einzugeben.	Klicken Sie hier, um ein Datum einzugeben.
	Bitte wählen Sie einen oder mehrere Gründe aus:
	wesentlichen Veränderungen:<br>☐ Umgestaltung<br>☐ Festlegung von Arbeitsplätzen<br>☐ Änderung von Arbeitsverfahren<br>☐ Änderung von Arbeitsabläufe<br>☐ Änderung der Arbeitsorganisation<br>☐ Einsatz anderer Arbeitsmittel<br>☐ Einsatz anderer Arbeitsstoffe<br>☐ Änderung oder Beschaffung von Maschinen, Geräten und Einrichtungen<br>☐ Instandhaltung
	☐ Änderung von Rechtsvorschriften
	X bei neuen arbeitswissenschaftlichen Erkenntnissen bzw. Veränderungen des Standes der Technik, Arbeitsmedizin und Hygiene
	☐ nach dem Erkennen von kritischen Situationen
	☐ nach Bekanntwerden einer Behinderung bei Beschäftigten
	☐ nach Arbeitsunfällen und Berufskrankheiten

Begriffsdefinition

- Die **Gefährdungsbeurteilung** ist die systematische Ermittlung und Beurteilung aller möglichen Gefährdungen der Beschäftigten einschließlich der Festlegung der erforderlichen Maßnahmen für Sicherheit und Gesundheit bei der Arbeit.
- Eine **Gefährdung** bezeichnet die Möglichkeit eines Gesundheitsschadens oder einer gesundheitlichen Beeinträchtigung ohne bestimmte Anforderungen an deren Ausmaß oder Eintrittswahrscheinlichkeit.
- Eine **Gefahr** bezeichnet eine Sachlage, die bei ungehindertem Ablauf des zu erwartenden Geschehens mit hinreichender Wahrscheinlichkeit zu einem Gesundheitsschaden oder einer gesundheitlichen Beeinträchtigung führt.
- **Wechselwirkung** im Sinne des Arbeitsschutzes ist die gegenseitige Beeinflussung von Gefährdungen oder Maßnahmen, wodurch sich Ausmaß und Art der Gefährdung verändern können.

Quelle: ASR V3

Allgemeine Grundsätze

1. Die Arbeit ist so zu gestalten, dass eine Gefährdung für das Leben sowie die physische und die psychische Gesundheit möglichst vermieden und die verbleibende Gefährdung möglichst geringgehalten wird;
2. Gefahren sind an ihrer Quelle zu bekämpfen;
3. bei den Maßnahmen sind der Stand von Technik, Arbeitsmedizin und Hygiene sowie sonstige gesicherte arbeitswissenschaftliche Erkenntnisse zu berücksichtigen;
4. Maßnahmen sind mit dem Ziel zu planen, Technik, Arbeitsorganisation, sonstige Arbeitsbedingungen, soziale Beziehungen und Einfluss der Umwelt auf den Arbeitsplatz sachgerecht zu verknüpfen;
5. individuelle Schutzmaßnahmen sind nachrangig zu anderen Maßnahmen;
6. spezielle Gefahren für besonders schutzbedürftige Beschäftigtengruppen sind zu berücksichtigen;
7. den Beschäftigten sind geeignete Anweisungen zu erteilen;
8. mittelbar oder unmittelbar geschlechtsspezifisch wirkende Regelungen sind nur zulässig, wenn dies aus biologischen Gründen zwingend geboten ist.

Quelle: § 4 ArbSchG

Rahmeninformationen zur Gefährdungsbeurteilung

Ersteller der Gefährdungs-beurteilung:	Axel Zinkernagel (Antragsteller)
Position:	Wiss. Mitarbeiter
Qualifikation:	Promotion
Organisationseinheit:	FB8 Psychologie, AG DDPME, Universität Koblenz-Landau, Campus Landau
Leitung der Organisations-einheit:	Eunike Wetzel, Tanja Lischetzke
Zu beurteilende Tätigkeit[1]:	Einzelerhebung für eine Laborstudie
Dauer und Häufigkeit der Tätigkeit:	Termine und Uhrzeiten werden mit den StudienteilnehmerInnen vereinbart
Zu beurteilender Arbeits-platz:	Laborräume

Welche **besonders schützenswerten Personengruppen** (vgl. § 4 Nr. 6 ArbSchG) sollten die Tätigkeit ausführen (können)? Bitte beachten Sie die ggf. zusätzlichen/abweichenden Rechtsvorschriften.
(Bitte auswählen. Mehrfachnennung möglich)

☐ Werdende Mütter (MuSchG)
☐ Stillende Mütter (MuSchG)
☐ Jugendliche (u.a. JArbSchG)
☐ Menschen mit Behinderung
(Bitte Art der Behinderung auswählen. Mehrfachnennung möglich)
☐ Sehbehinderung / Blindheit
☐ Hörbehinderung / Gehörlosigkeit
☐ Sprachbehinderung
☐ Motorische Behinderung
☐ Geistige Behinderung
☐ Mehrfachbehinderung / Schwerbehinderung
☐ Sonstige: Klicken Sie hier, um Text einzugeben.

Relevante Rechtsgrundlagen (Aufzählung):

☐ Arbeitsschutzgesetz
☐ DGUV Vorschrift 1
☐ Arbeitsstättenverordnung
☐ Mutterschutzgesetz
☐ Betriebssicherheitsverordnung
☐ Gefahrstoffverordnung
☐ Lastenhandhabungsverordnung
☐ Biostoffverordnung
☐ Lärm- und Vibrations-Arbeitsschutzverordnung
☐ PSA-Benutzungsverordnung
☐ Sonstige: Klicken Sie hier, um Text einzugeben.

1 Der Arbeitgeber hat die Beurteilung je nach Art der Tätigkeiten vorzunehmen. Bei gleichartigen Arbeitsbedingungen ist die Beurteilung eines Arbeitsplatzes oder einer Tätigkeit ausreichend.

Erstellung der Gefährdungsbeurteilung unter Beteiligung von:

	Name, Kontaktmöglichkeit, ggf. Firma
Führungskraft:	Eunike Wetzel, Tanja Lischetzke (FB 8, AG DDPME)
Betroffene Beschäftigte:	Birgit Petrzig (Empra-Tutorin)
Sicherheitsbeauftragte (gemäß § 22 SGB VII):	Klicken oder tippen Sie hier, um Text einzugeben.
Personalvertretung:	Klicken oder tippen Sie hier, um Text einzugeben.
Fachkraft für Arbeitssicherheit:	Britta Mann, Referat 52
Betriebsarzt:	
Weitere Personen:	Axel Zinkernagel (Antragsteller, FB 8, AG DDPME)

Mitgeltende Unterlagen: Klicken Sie hier, um Text einzugeben.

Sollten Sie noch nie eine Gefährdungsbeurteilung erstellt haben oder ihr Wissen grundlegend aktualisieren wollen, stehen Ihnen die Ansprechpartner*innen des Arbeitsschutzes vor Ort zur Verfügung. Einen guten Einstieg in das Thema bietet die Beleuchtung der (Arbeitsschutz-)Organisation. Die „Gemeinsame Deutsche Arbeitsschutzstrategie“ hat hierzu den GDA-ORGAcheck entwickelt und stellt diesen online bereit: https://www.gda-orgacheck.de/daten/gda/auswahl2.htm

Der nun folgende Abschnitt „Hintergrund: Prozessschritte Gefährdungsbeurteilung“ ist zur Übersichtlichkeit des Dokumentes ausgeblendet/eingeklappt. Wenn die Erläuterung des Vorgehens bei einer Gefährdungsbeurteilung ihnen bereits bekannt ist, können Sie unmittelbar mit der Prüfung der Gefährdungsfaktoren auf den folgenden Seiten beginnen.

Hintergrund: Prozessschritte Gefährdungsbeurteilung

Das Arbeitsschutzgesetz (ArbSchG) und die DGUV Vorschrift 1 sind gleichwertige Rechtsvorschriften, die die Basis für den Arbeitsschutz in Deutschland legen. Staatliches und autonomes Recht der Unfallversicherungsträger unterscheiden sich hierbei kaum. Dennoch sollte eine betriebliche Führungskraft (grob) mit beiden Regelwerken vertraut sein. TIPP: Die DGUV Regel 100-001 ist eine kommentierte Fassung der DGUV Vorschrift 1.

Die Gefährdungsbeurteilung muss als fortlaufender Prozess verstanden werden. Das folgende Schaubild zeigt die Prozessschritte der Gefährdungsbeurteilung:

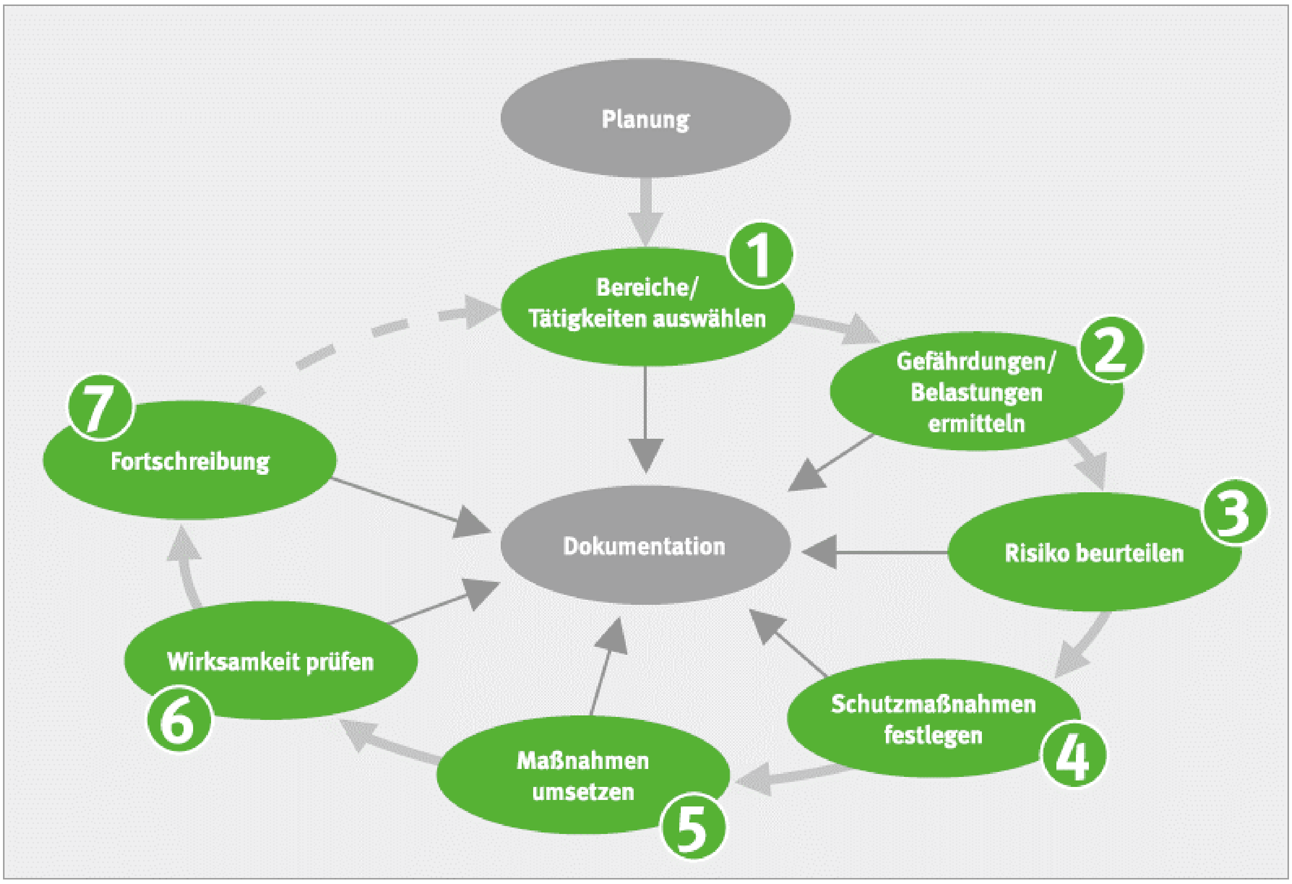

Abb. 1: Schematische Darstellung der Prozessschritte der Gefährdungsbeurteilung (Quelle: VBG – Gefährdungsbeurteilung „So geht's")

Schritt 1 haben Sie bereits über die Eintragungen in den Kopfdaten erfüllt. Die Schritte 2 „Gefährdung/Belastung ermitteln", 3 „Risiko beurteilen" und 4 „Schutzmaßnahmen festlegen", sowie 6 „Wirksamkeit prüfen" werden häufig zusammengefasst. So auch im Folgenden. Durch dieses Vorgehen wird die vorgeschriebene (schriftliche) Dokumentation vereinfacht.

Zur Ermittlung der Gefährdungen/Belastungen bearbeiten Sie einfach die nachstehenden Tabellen. Die Aufzählungen sind nie abschließend und können ergänzt werden. Es ist eine zyklische Bearbeitung empfehlenswert, indem Sie sich erst einmal einen Überblick über die möglichen Gefährdungen verschaffen, diese falls vorhanden stichpunktartig benennen (Nachvollziehbarkeit der Einschätzung) und dann in Absprachen mit den betroffenen Stellen Maßnahmen planen.

„Wichtig ist insbesondere die Einbeziehung der Mitarbeiterinnen und Mitarbeiter und der Sicherheitsbeauftragten, da diese als unmittelbar Betroffene die Gefährdungen und Belastungen im betrieblichen Alltag am besten kennen" (VBG – Gefährdungsbeurteilung „So geht's). Auch die Mitbestimmungsrechte der Personalvertretung müssen beachtet werden. Eine Gefährdungsbeurteilung an sich ist nicht mitbestimmungspflichtig. VERDI: „Das Bundesverwaltungsgericht hat 2002 festgestellt, dass die Durchführung einer Gefährdungsbeurteilung gem. § 5 Abs. 1 ArbSchG nach § 75 BPersVG nicht mitbestimmungspflichtig ist. Auch die neuere Rechtsprechung des BVerwG, etwa im Urteil vom 05.03.2012 – 6 PB 25/11 hat daran festgehalten […].[2]"

„Für das Beurteilen der Gefährdung im Hinblick auf das zu erreichende Schutzziel […] sind zunächst Beurteilungsmaßstäbe erforderlich, die in der Regel aus dem einschlägigen Vorschriften- und Regelwerk sowie aus der Fachliteratur abzuleiten sind. Fehlen solche Beurteilungsmaßstäbe müssen diese betrieblich vereinbart werden. Anhand dieser Beurteilungsmaßstäbe erfolgt danach das Be-

2 http://www.verdi-gefährdungsbeurteilung.de/page.php?k1=main&k2=aktiveinsetzen&k3=bpersvg

urteilen der Gefährdungen (Pkt 5.3 ASR V3)“. Um zu der dreistufigen Einschätzung des Risikos (Ampel-Prinzip) zu gelangen, wird i.d.R. die Risikomatrix nach Nohl (vgl. Abb. 2) eingesetzt. Diese Matrix kann zum Risiko-Würfel weiterentwickelt werden, indem auf der Z-Achse die Anzahl der betroffenen Personen hinzugefügt wird.

Eintrittswahrscheinlichkeit	Schadensschwere				
	Keine gesundheitlichen Folgen	Bagatellfolgen (die Arbeit kann fortgesetzt werden)	Mäßig schwere Folgen (Arbeitsausfall, ohne Dauerschäden)	Schwere Folgen (irreparable Dauerschäden möglich)	Tödliche Folgen
Praktisch unmöglich	gering	gering	gering	mittel	mittel
Vorstellbar	gering	gering	mittel	mittel	hoch
Durchaus möglich	gering	mittel	mittel	hoch	hoch
Zu erwarten	gering	mittel	hoch	hoch	hoch
Fast gewiss	gering	mittel	hoch	hoch	hoch

Abb. 2: Beispiel einer Risikomatrix zur Einstufung des Risikos von Gefährdungen und Belastungen (Quelle: VBG – Gefährdungsbeurteilung „So geht‘s“)

Die Einschätzung des Risikos ist direkt gekoppelt mit der Notwendigkeit von Maßnahmen. Die Zusammenhänge sind in Abb. 3 beschrieben.

grün: Das Risiko ist gering → keine Maßnahmen erforderlich, prüfen, ob Verbesserung möglich ist

gelb: Das Risiko ist vorhanden → Maßnahmen zur Minderung des Risikos sind erforderlich

rot: Das Risiko ist hoch → Maßnahmen zur Minderung des Risikos sind unverzüglich durchzuführen

Abb. 3: Zusammenhang zwischen Risikoeinschätzung und Maßnahmenbedarf (Quelle: VBG – Gefährdungsbeurteilung „So geht‘s)

Die Rangfolge der Schutzmaßnahmen basiert auf den Allgemeinen Grundsätzen gemäß § 4 ArbSchG. Es wird nach dem (S-)T-O-P-Prinzip verfahren:

- **S**ubstitution
- **T**echnische Maßnahme
- **O**rganisatorische Maßnahme
- **P**ersonenbezogene Maßnahme

In Abb. 4 wird das (S-)T-O-P-Prinzip anschaulich dargestellt. Schutzziel ist den Luftballon vor dem Kontakt mit den Nadeln der Kaktee zu bewahren. Die Gefahr liegt im möglichen Kontakt des Luftballons mit den Nadeln, was (mit hinreichender Wahrscheinlichkeit) zum Platzen führen würde.

Abb. 4: Rangfolge der Schutzmaßnahmen (Quelle: VBG – Gefährdungsbeurteilung „So geht‘s)

Die Kaktee zu entfernen (1. Substitution) beseitigt die Gefährdung auch für zukünftige Situationen.

Wenn das Entfernen keine Option ist, weil die Kaktee für das Arbeitsergebnis unbedingt erforderlich ist (z.B. als Anschauungsobjekt in einem Tropenhaus), kann diese z.B. hinter Glas gebracht werden (2. Tech. Schutzmaß-nahme).

Eine organisatorische Maßnahme (3) ist z.B. die Steuerung über den Faktor Zeit. Je kürzer der Luftballon in der Nähe der Kaktee ist, desto geringer wird die Eintrittswahrscheinlichkeit des schadhaften Ereignisses.

Eine 4. Persönliche Schutzausrüstung (PSA) für den Luftballon könnten zusätzliche widerstandsfähige Schichten sein, die bei einem Kontakt zu den Nadeln ein Platzen verhindern. Allerdings müsste jeder Luftballon damit ausgestattet werden.

Die Verhaltensbezogene Schutzmaßnahme (5) gehört zu den personenbezogenen Maßnahmen. Hierbei wäre denkbar, dass ein(e) Mitarbeiter(in) des Tropenhauses am Eingang allen Kindern einzeln erklärt, dass diese sich bei den Kakteen besonders in Acht nehmen, weil der Ballon sonst platzen könnte. Ob sich die Kinder an diesen Appel noch erinnern, wenn es so weit ist oder die Eltern darauf achten? Wenn der Ballon platzt ist der Schaden eingetreten und alle guten Worte und Absichtsbekundungen haben nicht geholfen. Das Risiko konnte nicht auf das erforderliche Maß gesenkt werden.

Die Reichweite der Maßnahme muss so hoch wie möglich sein. Daher sind individuelle Schutzmaßnahmen nachrangig zu anderen Maßnahmen (vgl. § 4 ArbSchG). Ziel jeder Maßnahme ist das Restrisiko unter das Grenzrisiko (vgl. Abb. 5) zu bringen.

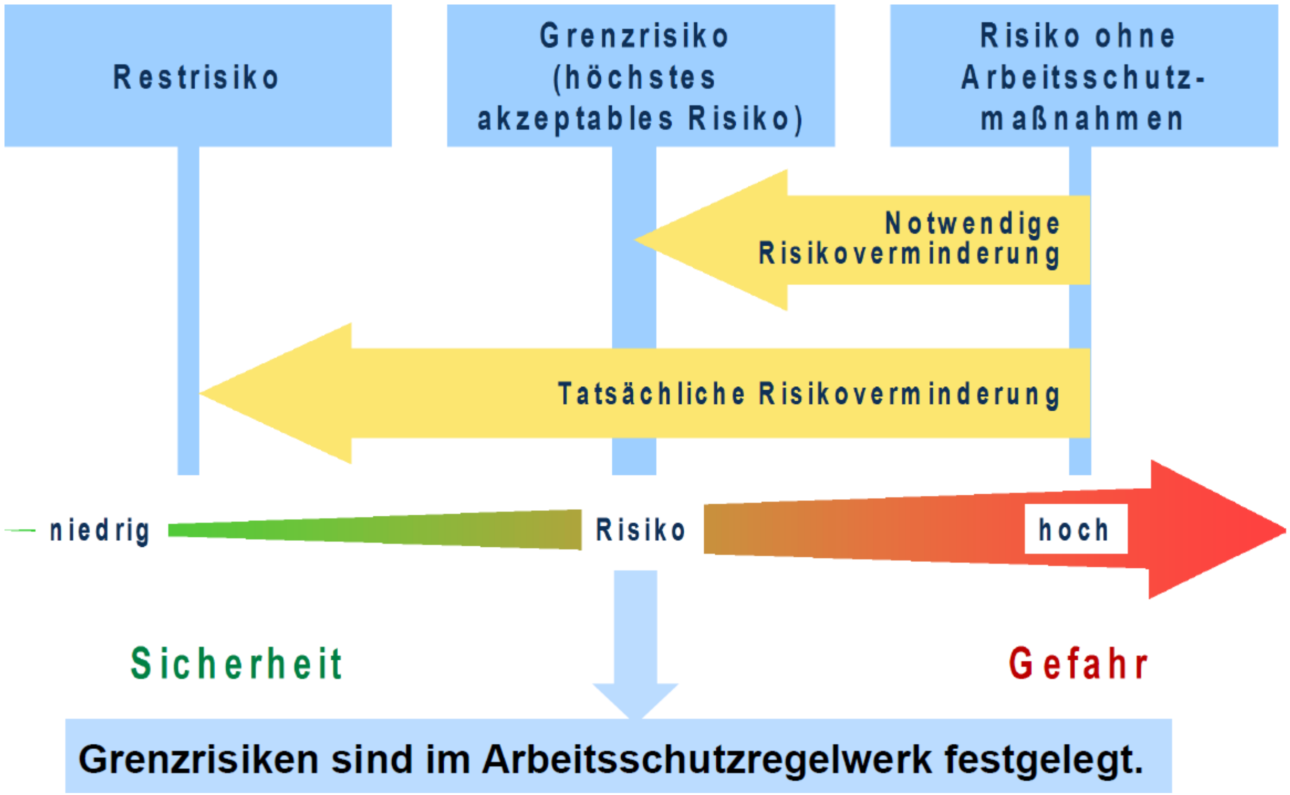

Abb. 5: Erforderliche Risikoredizierung durch Maßnahmen (Quelle: DGUV)

Maßnahmenvorschläge gibt es in der einschlägigen Literatur. Die Bundesanstalt für Arbeitsschutz und Arbeitsmedizin hat ein umfangreiches Angebot zur Gefährdungsbeurteilung erstellt. Dort können alle genannten Faktoren einzeln recherchiert werden.

https://www.baua.de/DE/Themen/Arbeitsgestaltung-im-Betrieb/Gefaehrdungsbeurteilung/_functions/BereichsPublikationssuche_Formular.html?nn=8696656

Es ist zu prüfen, ob die Maßnahmen für die betriebliche Situation geeignet sind. Beim Festlegen der Maßnahmen sollten Beschäftigte und Führungskräfte möglichst frühzeitig beteiligt werden, um eine hohe Akzeptanz der Maßnahmen zu erreichen. Evtl. Mitbestimmungs-, Mitwirkungs-, Anhörungs-, Informationsrechte der Personalvertretung sind zu beachten.

Mit Ausnahme der Schriftform ist die Gefährdungsbeurteilung formlos möglich. Es sind viele Software-Produkte am Markt verfügbar, die ebenfalls verwendet werden können. Eine kostenfreie Option wird von der Verwaltungs-BG angeboten: http://www.vbg.de/DE/3_Praevention_und_Arbeitshilfen/2_Themen/11_Gefaehrdungsbeurteilung/2_Grundlagen/Wichtiges%20vorweg_grundlagen_node.html

	1 Mechanische Gefährdungen					
☐	1.1 Ungeschützt bewegte Maschinenteile		☐	☐	☐	
☐	1.2 Teile mit gefährlichen Oberflächen		☐	☐	☐	
☐	1.3 Bewegte Transportmittel, bewegte Arbeitsmittel		☐	☐	☐	
☐	1.4 Unkontrolliert bewegte Teile		☐	☐	☐	
☐	1.5 Sturz, Ausrutschen, Stolpern, Umknicken		☐	☐	☐	
☐	1.6 Absturz		☐	☐	☐	
☐	Sonstige:<br>Klicken Sie hier, um Text einzugeben.		☐	☐	☐	

	**2 Elektrische Gefährdungen**					
☐	2.1 Elektrischer Schlag		☐	☐	☐	
☐	2.2 Lichtbögen		☐	☐	☐	
☐	2.3 Elektrostatische Aufladungen		☐	☐	☐	
☐	Sonstige:<br>Klicken Sie hier, um Text einzugeben.		☐	☐	☐	
	**3 Gefahrstoffe**					
☐	3.1 Hautkontakt mit Gefahrstoffen (Feststoffe, Flüssigkeiten, Feuchtarbeit)		☐	☐	☐	
☐	3.2 Einatmen von Gefahrstoffen (Gase, Dämpfe, Nebel, Stäube einschließlich Rauche)		☐	☐	☐	
☐	3.3 Verschlucken von Gefahrstoffen		☐	☐	☐	
☐	3.4 Physikalisch-chemische Gefährdungen – zum Beispiel Brand- und Explosionsgefährdungen, unkontrollierte chemische Reaktionen		☐	☐	☐	
☐	Sonstige:<br>Klicken Sie hier, um Text einzugeben.		☐	☐	☐	
	**4 Biologische Arbeitsstoffe**					
☒	4.1 Infektionsgefährdung durch pathogene Mikroorganismen – zum Beispiel Bakterien, Viren, Pilze	Coronavirus SARS-CoV-2	☐	☐	☒	s. ergänzende GB „SARS-CoV-2“
☐	4.2 Sensibilisierende und toxische Wirkungen von Mikroorganismen		☐	☐	☐	
☐	Sonstige:<br>Klicken Sie hier, um Text einzugeben.		☐	☐	☐	
	**5 Brand- und Explosionsgefährdungen**					
☐	5.1 Brennbare Feststoffe, Flüssigkeiten, Gase		☐	☐	☐	
☐	5.2 Explosionsfähige Atmosphäre		☐	☐	☐	
☐	5.3 Explosivstoffe		☐	☐	☐	
☐	Sonstige:<br>Klicken Sie hier, um Text einzugeben.		☐	☐	☐	
	**6 Thermische Gefährdungen**					
☐	6.1 Heiße Medien/Oberflächen		☐	☐	☐	
☐	6.2 Kalte Medien/Oberflächen		☐	☐	☐	
☐	Sonstige:<br>Klicken Sie hier, um Text einzugeben.		☐	☐	☐	
	**7 Gefährdungen durch spezielle physikalische Einwirkungen**					
☐	7.1 Lärm		☐	☐	☐	
☐	7.2 Ultraschall, Infraschall		☐	☐	☐	
☐	7.3 Ganzkörpervibrationen		☐	☐	☐	
☐	7.4 Hand-Arm-Vibrationen		☐	☐	☐	
X	7.5 Optische Strahlung – zum Beispiel Infrarote Strahlung (IR), Ultraviolette Strahlung (UV), Laserstrahlung		X	☐	☐	UV-A-Strahlung (365 nm) unter 100 J/m²
☐	7.6 Ionisierende Strahlung – zum Beispiel Röntgenstrahlen, Gammastrahlung, Teilchenstrahlung (Alpha-, Beta- und Neutronenstrahlung)		☐	☐	☐	
☐	7.7 Elektromagnetische Felder		☐	☐	☐	
☐	7.8 Unter- oder Überdruck		☐	☐	☐	
☐	Sonstige:<br>Klicken Sie hier, um Text einzugeben.		☐	☐	☐	
	**8 Gefährdungen durch Arbeitsumgebungsbedingungen**					
☐	8.1 Klima – zum Beispiel Hitze, Kälte, unzureichende Lüftung		☐	☐	☐	
☐	8.2 Beleuchtung, Licht		☐	☐	☐	
☐	8.3 Ersticken – zum Beispiel durch sauerstoffreduzierte Atmosphäre, Ertrinken		☐	☐	☐	
☐	8.4 Unzureichende Flucht- und Verkehrswege, unzureichende Sicherheits- und Gesundheitsschutzkennzeichnung		☐	☐	☐	
☐	8.5 Unzureichende Bewegungsfläche am Arbeitsplatz, ungünstige Anordnung des Arbeitsplatzes, unzureichende Pausen-, Sanitärräume		☐	☐	☐	
☐	Sonstige:<br>Klicken Sie hier, um Text einzugeben.		☐	☐	☐	
	**9 Physische Belastung/Arbeitsschwere**					

☐	9.1 Schwere dynamische Arbeit – zum Beispiel manuelle Handhabung von Lasten		☐	☐	☐	
☐	9.2 Einseitige dynamische Arbeit, Körperbewegung – zum Beispiel häufig wiederholte Bewegungen		☐	☐	☐	
☐	9.3 Haltungsarbeit (Zwangshaltung), Haltearbeit		☐	☐	☐	
☐	9.4 Kombination aus statischer und dynamischer Arbeit		☐	☐	☐	
☐	Sonstige:<br>Klicken Sie hier, um Text einzugeben.		☐	☐	☐	
	**10 Psychische Belastung/Arbeitsschwere**					
☐	10.1 Ungenügend gestaltete Arbeitsaufgabe – zum Beispiel überwiegende Routineaufgaben, Über-/Unterforderung		☐	☐	☐	
☐	10.2 Ungenügend gestaltete Arbeitsorganisation – zum Beispiel Arbeiten unter hohem Zeitdruck, wechselnde und/oder lange Arbeitszeiten, häufige Nachtarbeit, kein durchdachter Arbeitsablauf		☐	☐	☐	
☐	10.3 Ungenügend gestaltete soziale Bedingungen – zum Beispiel fehlende soziale Kontakte, ungünstiges Führungsverhalten, Konflikte		☐	☐	☐	
☐	10.4 Ungenügend gestaltete Arbeitsplatz- und Arbeitsumgebungsbedingungen – zum Beispiel Lärm, Klima, räumliche Enge, unzureichende Wahrnehmung von Signalen und Prozessmerkmalen, unzureichende Softwaregestaltung		☐	☐	☐	
☐	Sonstige:<br>Klicken Sie hier, um Text einzugeben.		☐	☐	☐	
	**11 Sonstige Gefährdungen**					
☐	11.1 Durch Menschen – zum Beispiel Überfall		☐	☐	☐	
☐	11.2 Durch Tiere – zum Beispiel gebissen werden		☐	☐	☐	
☐	11.3 Durch Pflanzen und pflanzliche Produkte – zum Beispiel sensibilisierende und toxische Wirkungen		☐	☐	☐	
☐	Sonstige:<br>Klicken Sie hier, um Text einzugeben.		☐	☐	☐	
	**12 besondere Betiebszustände und (Notfall-)Szenarien**					
☐	12.1 Wetter	außergewöhnliche Temperaturen	☐	☐	☐	
		Sturm	☐	☐	☐	
		Hagel und Starkregen	☐	☐	☐	
		Gewitter mit Blitzgefahr	☐	☐	☐	
		Hochwasser	☐	☐	☐	
		Glatteis	☐	☐	☐	
☐	12.2 Besucher-/Nutzer-/Kundenverhalten	Überklettern von Absperrungen	☐	☐	☐	
		Erklettern von Aufbauten oder Straßenmobiliar	☐	☐	☐	
		besonders ausgeprägter Enthusiasmus	☐	☐	☐	
		Besucherdruck und Gedränge	☐	☐	☐	
		Werfen von Gegenständen	☐	☐	☐	
		Vandalismus, Körperverletzung	☐	☐	☐	
		sonstige Kriminalitätsdelikte wie Drogenkonsum und -verkauf, Taschendiebstahl, Waffen etc.	☐	☐	☐	
☐	12.3 sanitäts- und rettungsdienstliche Ereignisse	Verletzung	☐	☐	☐	
		Unfall	☐	☐	☐	
		hoher Drogen- / Alkoholkonsum	☐	☐	☐	
		Reizgas	☐	☐	☐	
		vermisste Personen / Kinder	☐	☐	☐	
		Massenerkrankungen durch Lebensmittelvergiftungen	☐	☐	☐	
☐	12.4 Brandgefährdungen	Brand, Explosion	☐	☐	☐	
		Verwendung von Pyrotechnik durch Besucher	☐	☐	☐	
☐	12.5 technische Gefährdungen	Gasausströmung	☐	☐	☐	
		Gesundheitsgefährdungen durch Austritt	☐	☐	☐	

		von Gefahrstoffen, wie Kühlmittel				
		Stromausfall	☐	☐	☐	
		Einsturz von Bauteilen oder Anlagen	☐	☐	☐	
		Unfälle (Fahrgeschäfte, Motorsport, Stuntshows, Tiere, Flugshows)	☐	☐	☐	
☐	12.6 Gefährdungen für die (besucherrelevante) Infrastruktur innerhalb / außerhalb der Veranstaltung inklusive der Verkehrswege	Ausfall gastronomischer Einrichtungen und somit unzureichende Versorgung der Gäste mit Getränken	☐	☐	☐	
		Ausfall der Ver- und Entsorgung	☐	☐	☐	
		Ausfall des öffentlichen Personen(nah)-verkehrs (z. B. durch Personenschaden im Gleisbereich)	☐	☐	☐	
		Ausfall des Individualverkehrs (z. B. Unfall, Sperrung)	☐	☐	☐	
		Parkflächen nach Verkehrskonzept nicht nutzbar	☐	☐	☐	
		Glasbruch	☐	☐	☐	
☐	Grundgefährdungen bei besonderen Veranstaltungstypen	besondere politische Lage (Auftreten sicherheitsrelevanter Personengruppen)	☐	☐	☐	
		Gegenveranstaltungen	☐	☐	☐	
		hohe Anzahl nicht selbstrettungsfähiger Personen (Kleinkinder, körperlich eingeschränkte Personen)	☐	☐	☐	
		Gewaltpotenzial der Veranstaltungsteilnehmer (Rockergruppen, Hooligans, Fanatiker)	☐	☐	☐	
☐	Gefährdungen durch Anschläge und Drohungen	Massenphänomene nach Drohungen	☐	☐	☐	
		Verdächtige Gegenstände	☐	☐	☐	
		Amoklauf	☐	☐	☐	
		Amokfahrt	☐	☐	☐	
		Sprengstoffanschlag	☐	☐	☐	
		Sprengstoffanschlag mit radioaktiver Verschmutzung	☐	☐	☐	
		Anschlag mit chemischen Stoffen	☐	☐	☐	
		Anschlag mit biologischen Stoffen	☐	☐	☐	
☐	Sonstige:<br>Klicken Sie hier, um Text einzugeben.		☐	☐	☐	

Bitte bestätigen Sie als zuständige Führungskraft, dass folgende Grundsätze bei der Bearbeitung eingehalten worden sind und fortlaufend zur Anwendung gebracht werden.

X Die geplanten Maßnahmen entsprechen dem Stand der Technik, Arbeitsmedizin und Hygiene sowie den Anforderungen der Ergonomie. Gesicherte arbeitswissenschaftliche Erkenntnisse sind berücksichtigt.

X Die Unterweisung der Mitarbeiter*innen hinsichtlich der Ergebnisse der Gefährdungsbeurteilung, möglicherweise verbleibenden Gefährdungen sowie ggf. der Auswirkung der festgelegten Maßnahme bzw. deren Umsetzung findet statt und wird dokumentiert.

X Bei der Festlegung von Maßnahmen sind die Zusammenhänge bzw. die Wechselwirkungen aus den resultierenden Gefährdungsfaktoren von Arbeitsstätte, Arbeitsplatz, Arbeitsmitteln, Arbeitsstoffen, Arbeitsorganisation und Arbeitsaufgabe berücksichtigt worden.

X Mir ist bekannt, dass im Rahmen des fortlaufenden Prozesses der Gefährdungsbeurteilung die Umsetzung und Wirksamkeit der festgelegten Maßnahmen geprüft werden muss. Dabei ist festzustellen, ob die Maßnahmen vollständig umgesetzt wurden und dazu geführt haben, die Gefährdungen zu beseitigen bzw. hinreichend zu reduzieren, und ob gegebenenfalls neue Gefährdungen entstanden sind. Die Prüfung kann z. B. durch Beobachten, Messen oder Befragen erfolgen.

X Wenn keine besonderen Anlässe eine Aktualisierung der Gefährdungsbeurteilung erfordern, wird in Zeiträumen von 3-5 Jahren eine Prüfung auf Aktualität vorgenommen.

X Die Dokumentation erfolgt schriftlich und kann als Papierdokument oder in elektronischer Form vorliegen. Sie muss in einer verbindlichen Version verfügbar sein.

X Die Gefährdungsbeurteilung wird für einen Zeitraum von 10 Jahren (falls nicht mehr gültig) verwahrt und bei Bedarf vorgelegt. Bei Tätigkeiten mit KMR-Stoffen (krebserzeugenden, erbgutverändernden/keimzellmutagenen oder fortpflanzungsgefährdenden Stoffen) gilt eine Aufbewahrungsfrist von 40 Jahren.

X Fach-/Sachkunde liegt vor oder wurde eingeholt.

9.12.20 [signature]

Datum / Unterschrift Führungskraft

Datum / Kenntnisnahme Personalvertretung

Weitere, mitzeichnende Personen:
