# Supplementary material for: blenderFaceUV: Toward Marker‐Based Precision and Flexibility in Facial Tracking With “Invisible” Markers
Source: Psychophysiology. 2026 Sep 21;63(9):e70394. doi: 10.1111/psyp.70394 (PMC13594373; doi:10.1111/psyp.70394)
Supplement: Supplementary file 3 [file PSYP-63-e70394-s001.zip › Approval_Ethics_Covid-19_Occupational_health_and_safety/Report_UV_LED.pdf]

BERGISCHE
UNIVERSITÄT
WUPPERTAL

Prof. Dr. Dirk Lützenkirchen-Hecht

Faculty 4 – Condensed Matter Physics

Gaußstraße 20, 42119 Wuppertal

Raum D.09.23
Telefon +49 (0)202 439 3103
Mobil
Fax +49 (0)202 439 3101
Mail dirklh@uni-wuppertal.de
Internet https://www.uni-wuppertal.de/
Aktenzeichen

Datum 24. Oktober 2024

Bergische Universität Wuppertal, Prof. Dr. Dirk Lützenkirchen-Hecht,
Gaußstraße 20, 42119 Wuppertal

Dr. Axel Zinkernagel
Gaußstraße 20
42119 Wuppertal

Measurements on UV LED lamps / Brief report

The aim was to determine the radiant power or radiant energy of the 100 W UV LED lamps (type CHX-FL-A-100 W). A direct energy measurement with pyroelectric sensors was not successful due to the low optical radiant power, therefore two calibrated pyroelectric sensors were first measured against each other with a pulsed UV laser (wavelength 355 nm) according to the illustrated setup and using a beam splitter, whereby one of the sensors was exposed to a strongly attenuated beam. In a second step, this sensor was replaced by a UV grating spectrometer, which enables the spectral distribution of both the laser and, in a third step, the LED lamp to be measured. Using the experimentally determined calibration factors, the energy-calibrated laser radiation can then be compared with the radiation from the lamp and the radiation from the UV LED can be assessed.

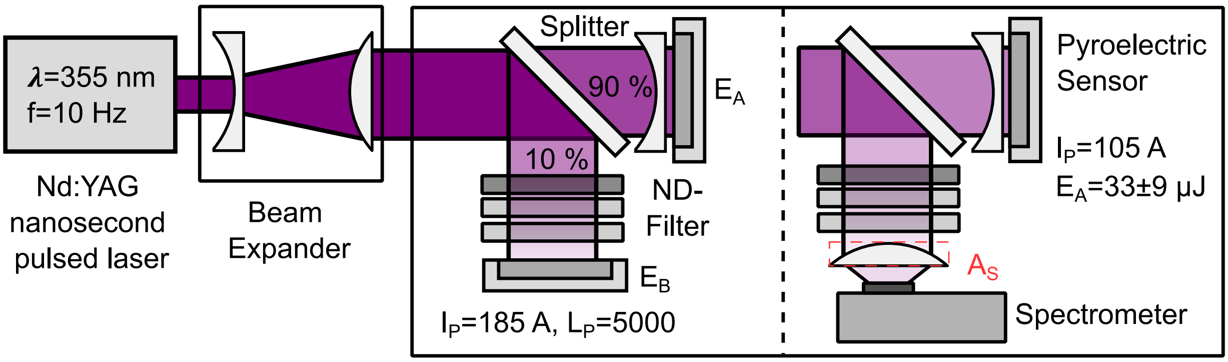

Fig. 1: Experimental setup for calibrating energy sensors and optical spectrometers

For the calibration of the two sensors, very high pump currents of the laser could be used, which allow an extremely high reproducibility from pulse to pulse of the laser. 5000 pulses were measured in order to obtain the most accurate measured values possible. With the beam splitter and the filters used, a ratio between the two sensors of 0.0004 was determined.

In the second step, the optical spectrometer was simultaneously used in the strongly attenuated branch of the setup and the laser was operated with a low pump current so as not to overdrive the sensitive spectrometer or damage it with the laser. The measurements of the laser power and the spectrum of the laser in the optical spectrometer are shown in Fig. 2. Due to the low pump current, the repeatability of the laser pulses is significantly poorer; an average pulse energy of 33 $\pm$ 9 μJ is determined.

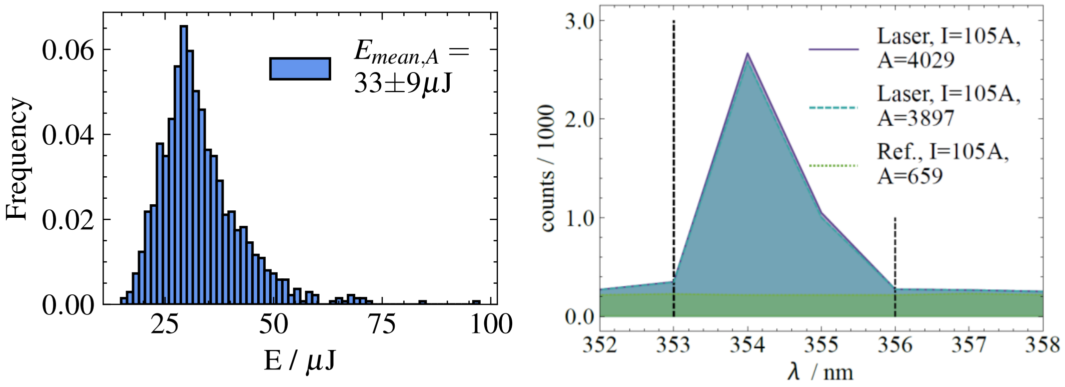

Fig. 2: Left: Laser pulses measured to calibrate the optical spectrometer (10 Hz). The average pulse energy is 33 $\pm$ 9 μJ. Right: Wavelength spectrum of the laser during the energy measurements for a pump current of 105 A. A background (green area, A = 659) is subtracted from the signals, so that the count rate for an integration time of 100 ms in the range of 355 nm is approximately 4029-659 = 3370.

The radiation spectrum measured by the UV light source is shown in Fig. 3; in accordance with Directive 2006/25/EC of the European Parliament and of the Council of April 5, 2006, the range important for radiation protection (UV-A) comprises the wavelengths from 315-400 nm; the corresponding measurement is shown in Fig. 3 for a distance of 2.5 m from the UV light source. Here too, the green-colored background was subtracted from the measurement signal, so that 78039 - 17589 = 60450 counts were measured, which must now be converted into a corresponding energy. An illuminated area of 10 mm^2 was measured.

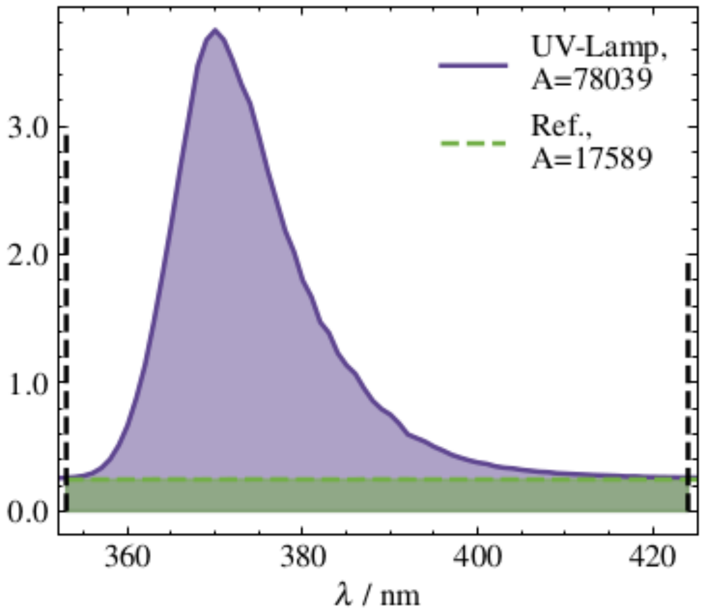

Fig. 3: Radiation distribution of the UV LED lamp measured with the optical spectrometer at a distance of 2.5 m from the lamp. The range from 352 nm to 420 nm was integrated and a total count rate of 78039 counts was measured for a measurement time of 3 ms, from which the green shaded background was subtracted (17589 counts).

If we convert the measured signals accordingly, take into account the active area of the spectrometer, assume that the laser power/energy is measured completely by the spectrometer due to the good focusing, and also take into account the 10 Hz clocking of the laser, we arrive at a total power of the LED lamp in the range between 352 nm and 420 nm of

$$P_{\mathrm{Lamp}} = 0.0004 * 33\ \mu\mathrm{J} * 10\ \mathrm{Hz} * 100\ \mathrm{ms}/3\ \mathrm{ms} * 60450/3370 = 78\ \mu\mathrm{W} \text{ on an area of } 10\ \mathrm{mm}^2\text{, i.e.}$$
$$P_{\mathrm{Lamp}} \approx 80\ \mu\mathrm{W}/10\ \mathrm{mm}^2 = 8\ \mathrm{W/m}^2$$

The legal limit for prolonged exposure (times of more than 10^4 s = approx. 3 hours) in the wavelength range of interest is 10^4 J/m^2 = 10000 J/m^2, i.e. at a radiation power of approx. 10 W/m^2, an exposure time of 1000 s = 16 min. at a distance of 2.5 m would also be considered harmless to the eye. For greater distances, the radiant power decreases accordingly, e.g., for a distance of 5 m, only $\frac{1}{4}$ of the radiant power would be expected.

(Prof. Dr. Dirk Lützenkirchen-Hecht)
