# Supplementary material for: blenderFaceUV: Toward Marker‐Based Precision and Flexibility in Facial Tracking With “Invisible” Markers
Source: Psychophysiology. 2026 Sep 21;63(9):e70394. doi: 10.1111/psyp.70394 (PMC13594373; doi:10.1111/psyp.70394)
Supplement: Supplementary file 3 [file PSYP-63-e70394-s001.zip › Study 1/Plots/Participant_1_AU_Plot_Marker_DL2_x-axis.pdf]

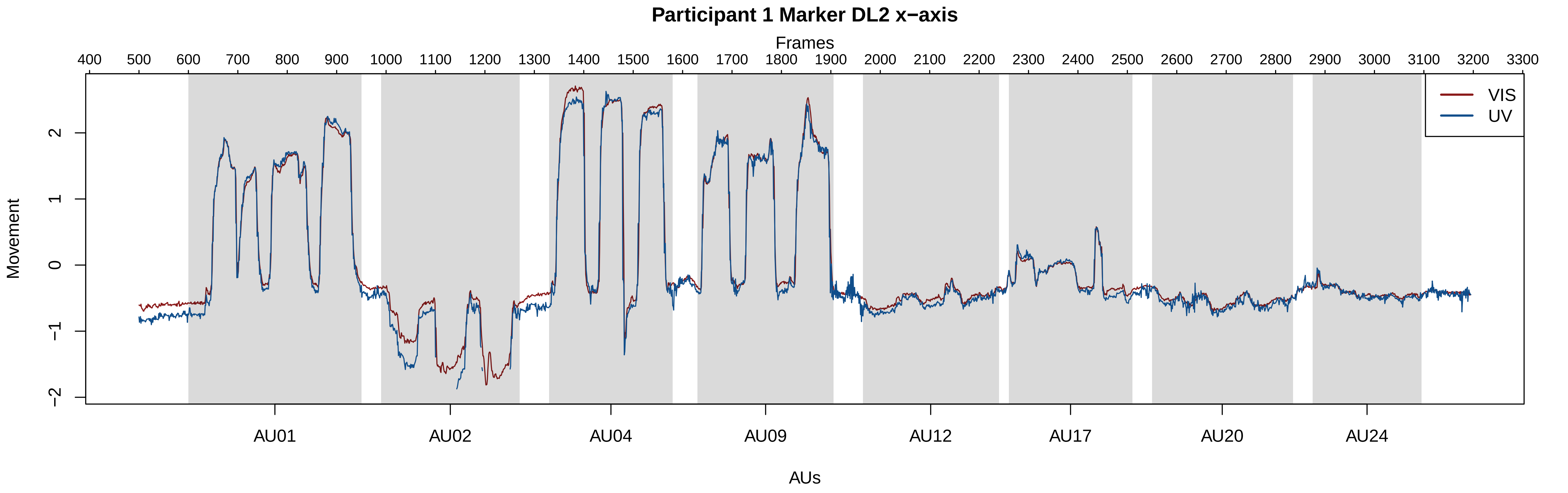
Participant 1 Marker DL2 x−axis
Frames
400
500
600
700
800
900
1000
1100
1200
1300
1400
1500
1600
1700
1800
1900
2000
2100
2200
2300
2400
2500
2600
2700
2800
2900
3000
3100
3200
3300
VIS
UV
Movement
2
1
0
−1
−2
AU01
AU02
AU04
AU09
AU12
AU17
AU20
AU24
AUs
