# Supplementary material for: blenderFaceUV: Toward Marker‐Based Precision and Flexibility in Facial Tracking With “Invisible” Markers
Source: Psychophysiology. 2026 Sep 21;63(9):e70394. doi: 10.1111/psyp.70394 (PMC13594373; doi:10.1111/psyp.70394)
Supplement: Supplementary file 3 [file PSYP-63-e70394-s001.zip › Study 1/Plots/Participant_2_Emo_Plot_Marker_DL2_x-axis.pdf]

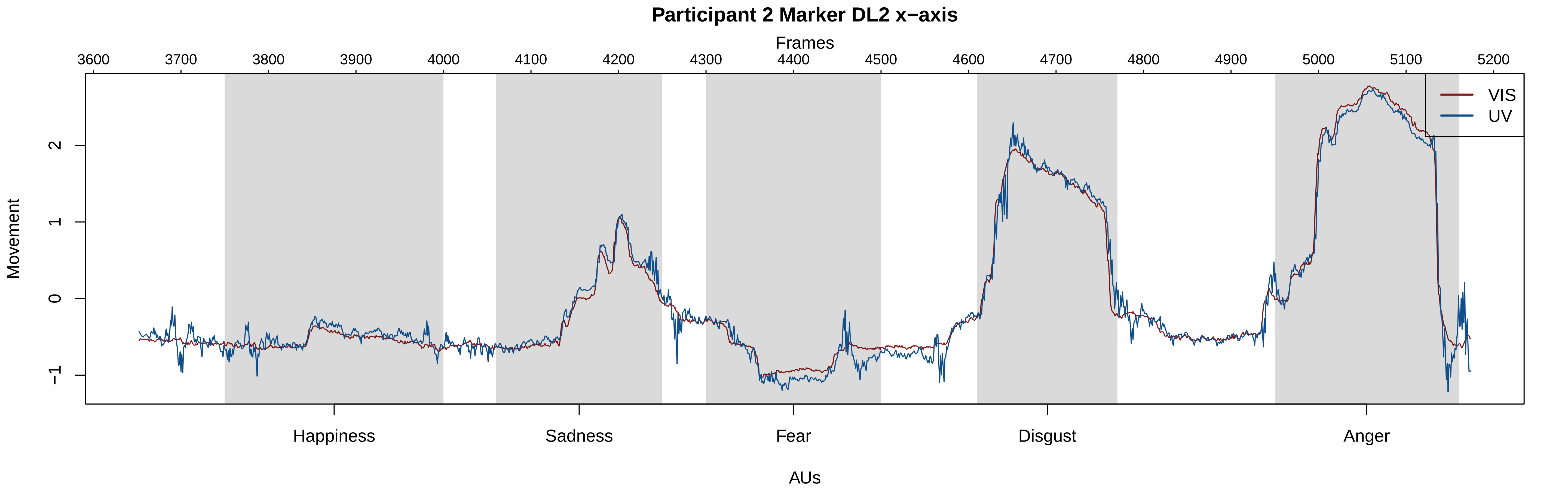

Participant 2 Marker DL2 x−axis
Frames
3600
3700
3800
3900
4000
4100
4200
4300
4400
4500
4600
4700
4800
4900
5000
5100
5200
Movement
2
1
0
−1
VIS
UV
Happiness
Sadness
Fear
Disgust
Anger
AUs
