# Supplementary material for: blenderFaceUV: Toward Marker‐Based Precision and Flexibility in Facial Tracking With “Invisible” Markers
Source: Psychophysiology. 2026 Sep 21;63(9):e70394. doi: 10.1111/psyp.70394 (PMC13594373; doi:10.1111/psyp.70394)
Supplement: Supplementary file 3 [file PSYP-63-e70394-s001.zip › Study 1/Plots/Participant_2_AU_Plot_Marker_BR5_y-axis.pdf]

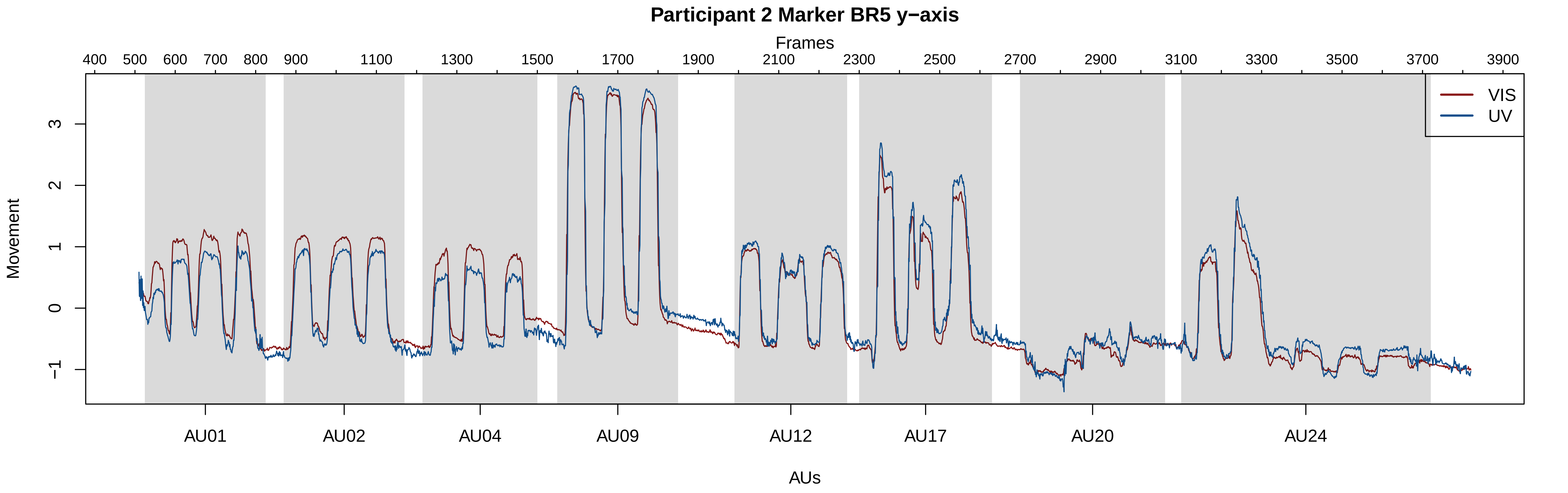

Participant 2 Marker BR5 y−axis
Frames
400 500 600 700 800 900 1100 1300 1500 1700 1900 2100 2300 2500 2700 2900 3100 3300 3500 3700 3900
Movement
−1 0 1 2 3
VIS
UV
AU01 AU02 AU04 AU09 AU12 AU17 AU20 AU24
AUs
