# Supplementary material for: blenderFaceUV: Toward Marker‐Based Precision and Flexibility in Facial Tracking With “Invisible” Markers
Source: Psychophysiology. 2026 Sep 21;63(9):e70394. doi: 10.1111/psyp.70394 (PMC13594373; doi:10.1111/psyp.70394)
Supplement: Supplementary file 3 [file PSYP-63-e70394-s001.zip › Study 1/Plots/Participant_2_AU_Plot_Marker_CL7_x-axis.pdf]

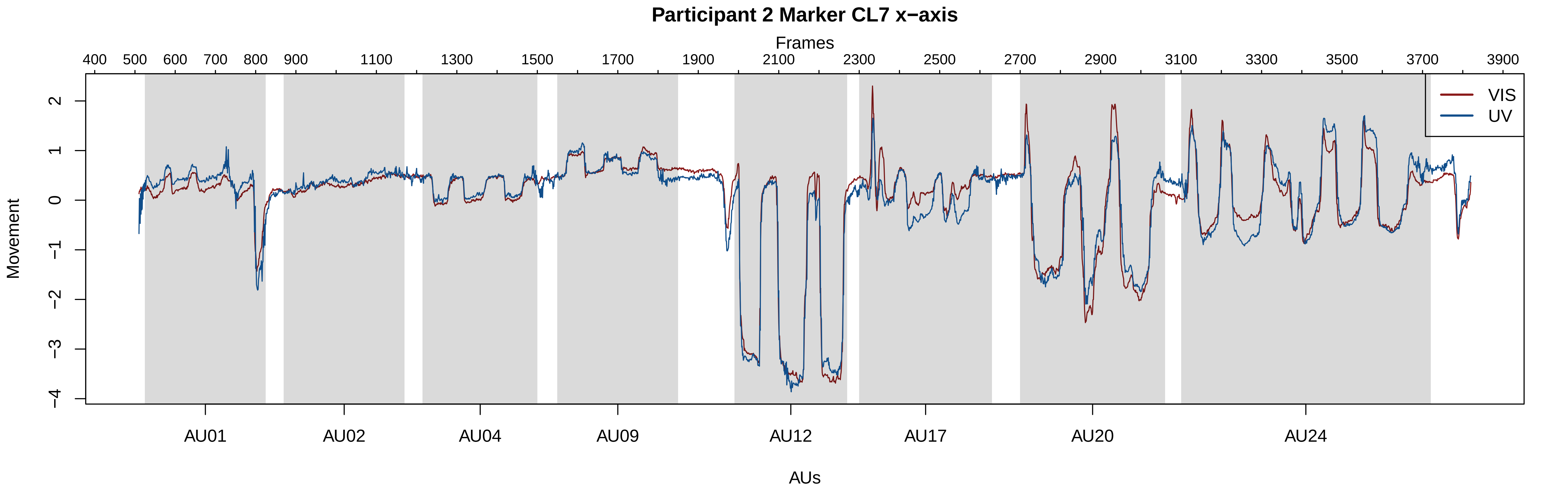

Participant 2 Marker CL7 x−axis
Frames
400
500
600
700
800
900
1100
1300
1500
1700
1900
2100
2300
2500
2700
2900
3100
3300
3500
3700
3900
Movement
2
1
0
−1
−2
−3
−4
VIS
UV
AU01
AU02
AU04
AU09
AU12
AU17
AU20
AU24
AUs
