# Supplementary material for: blenderFaceUV: Toward Marker‐Based Precision and Flexibility in Facial Tracking With “Invisible” Markers
Source: Psychophysiology. 2026 Sep 21;63(9):e70394. doi: 10.1111/psyp.70394 (PMC13594373; doi:10.1111/psyp.70394)

Participant 3 posing anger

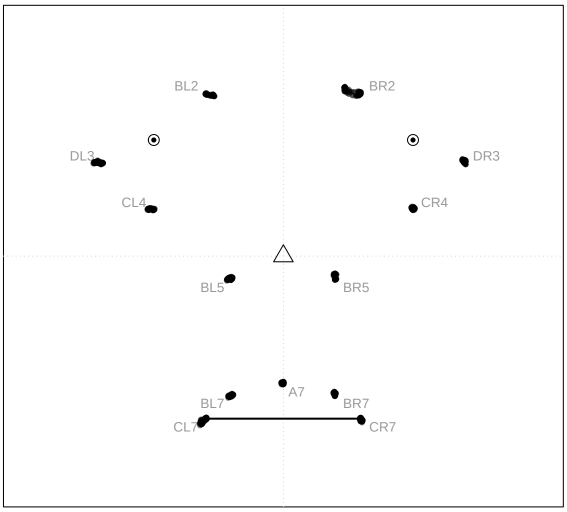

Participant 3 posing fear

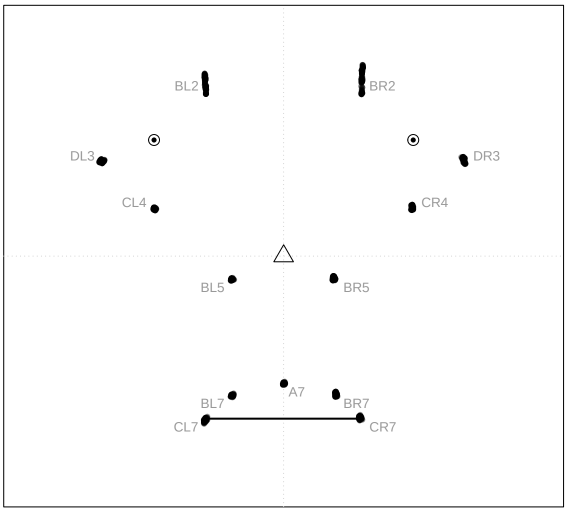

Participant 3 posing disgust

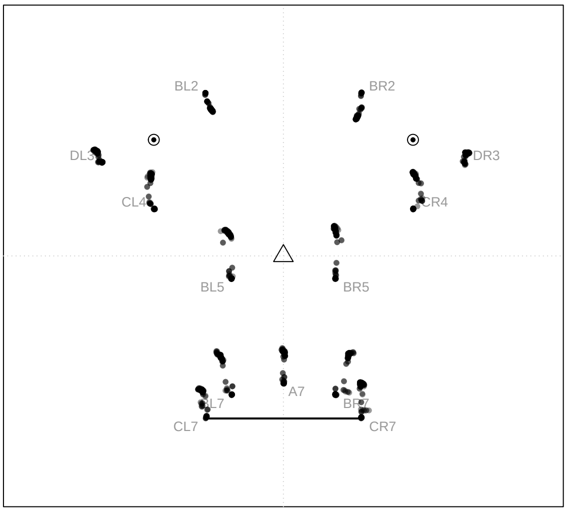

Participant 3 posing happiness

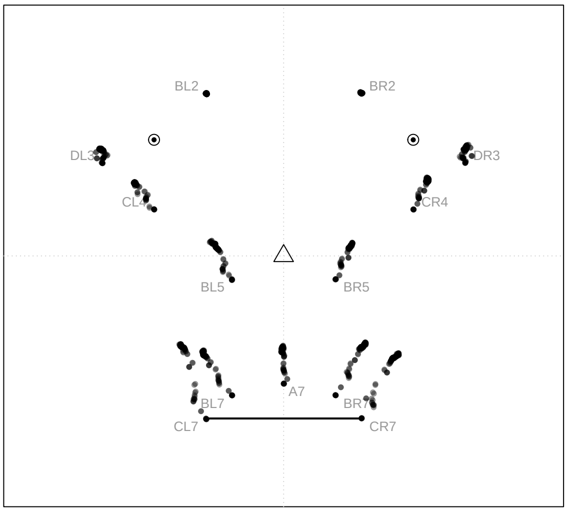

Participant 3 posing sadness

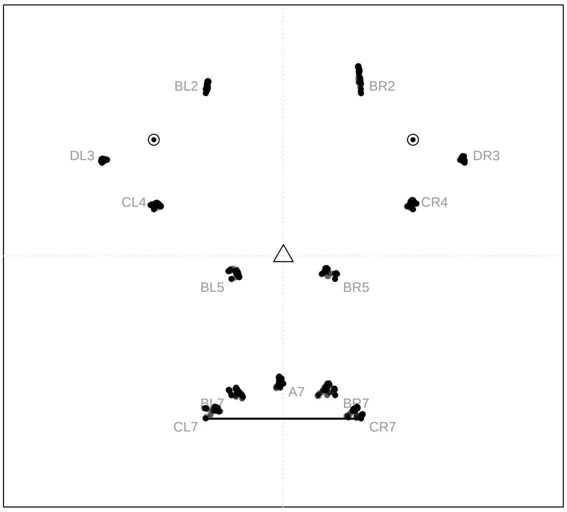

Participant 4 posing anger

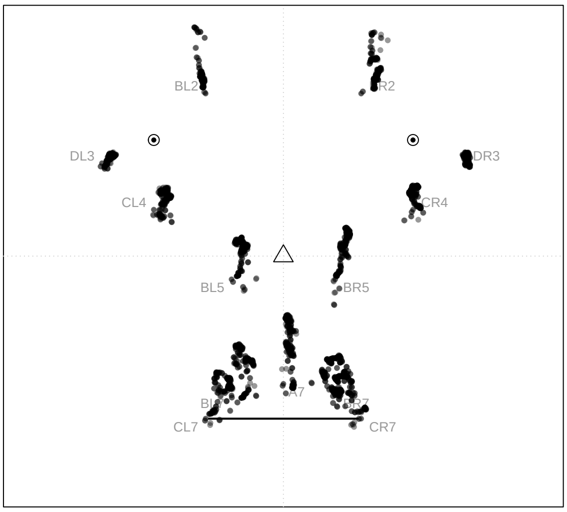

Participant 4 posing fear

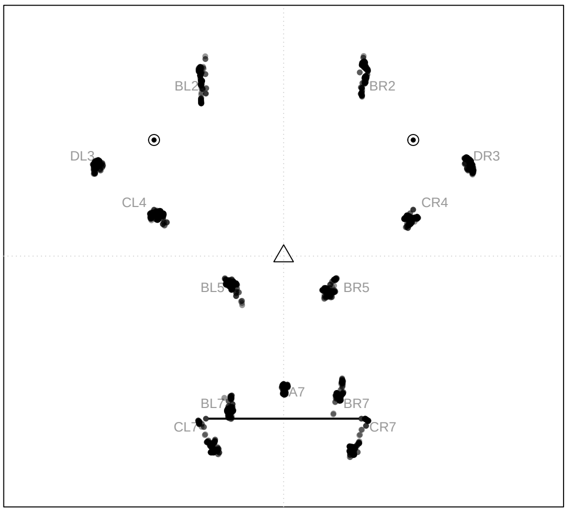

Participant 4 posing disgust

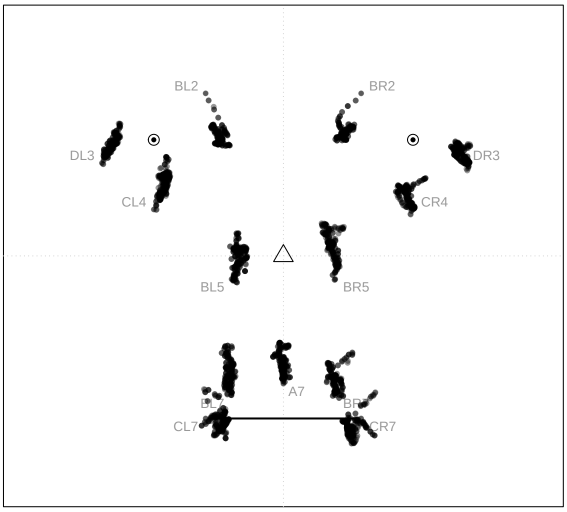

Participant 4 posing happiness

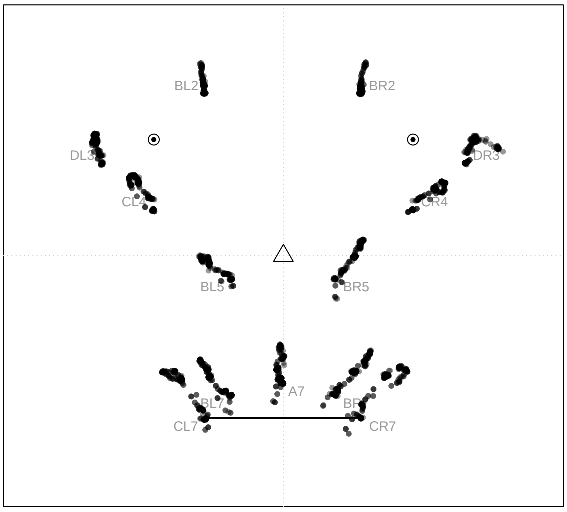

Participant 4 posing sadness

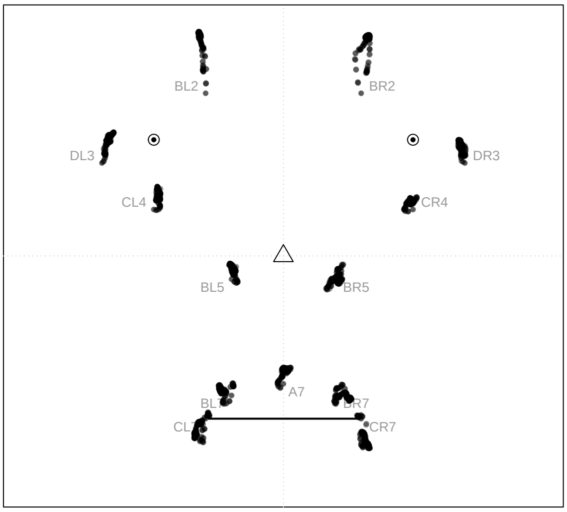

Participant 6 posing anger

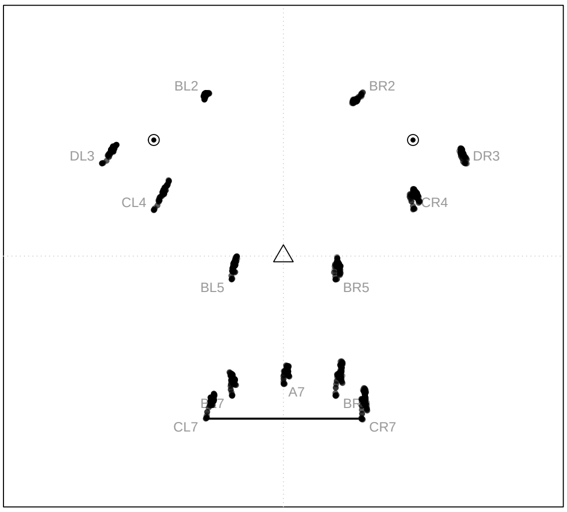

Participant 6 posing fear

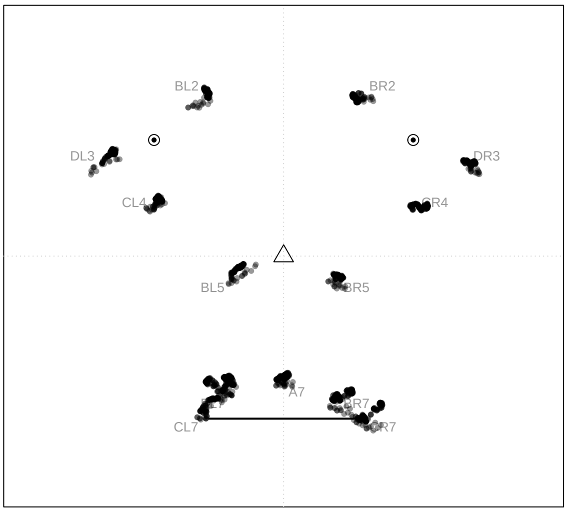

Participant 6 posing disgust

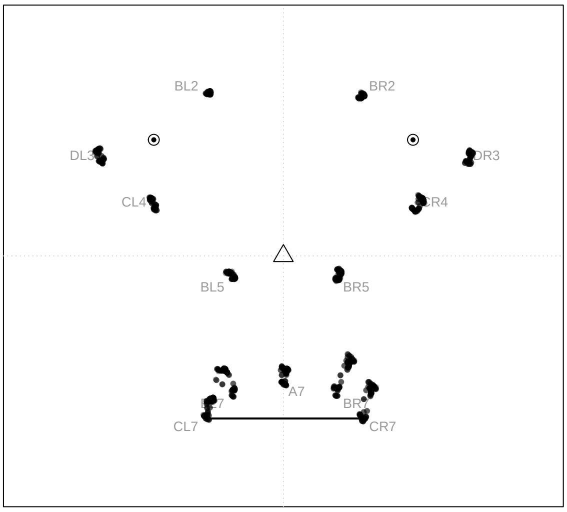

Participant 6 posing happiness

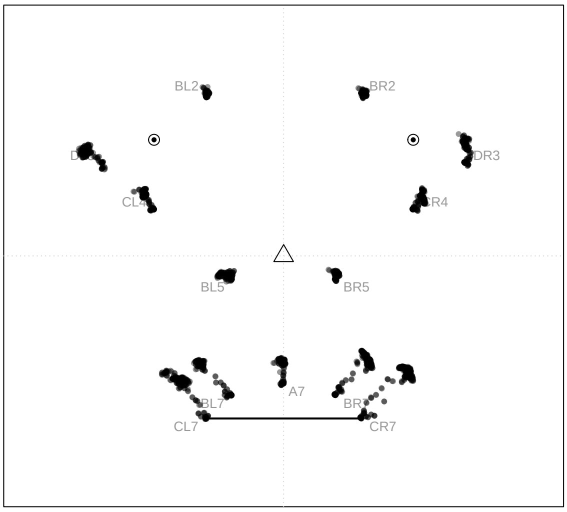

Participant 6 posing sadness

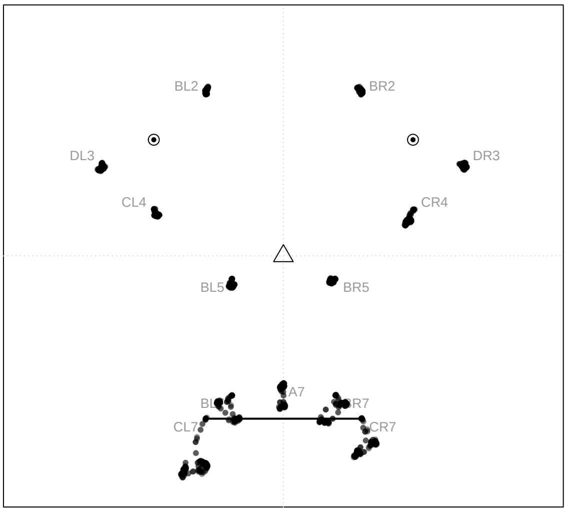

Participant 7 posing anger

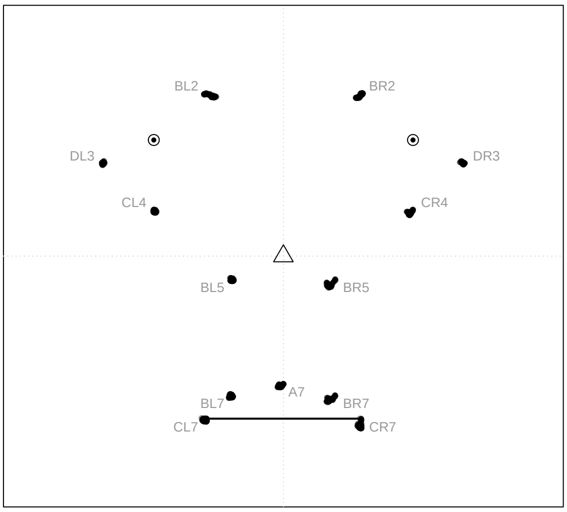

Participant 7 posing fear

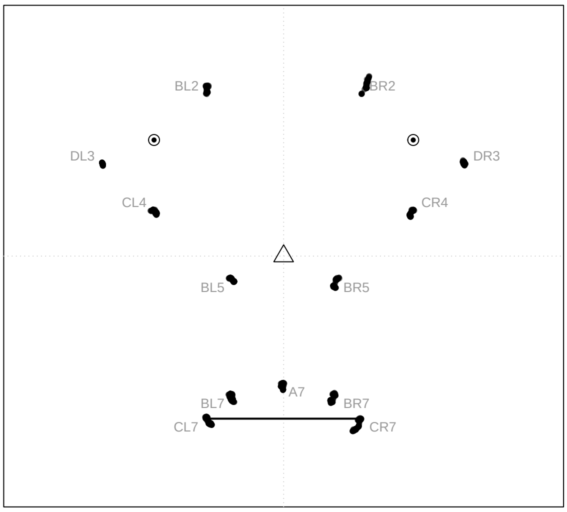

Participant 7 posing disgust

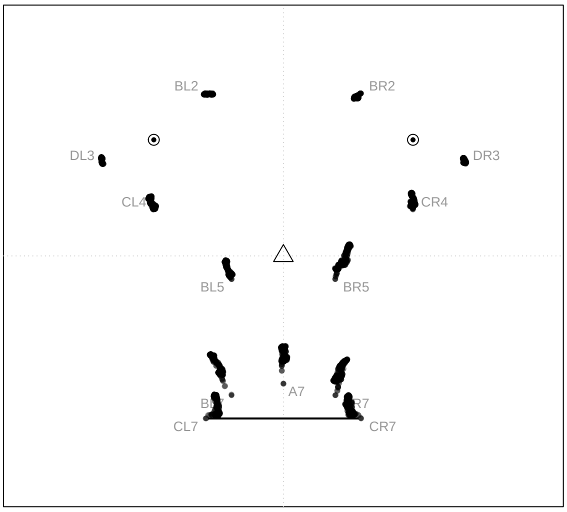

Participant 7 posing happiness

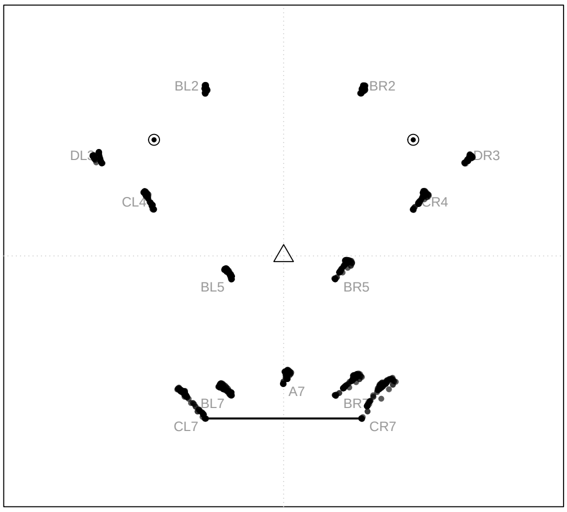

Participant 7 posing sadness

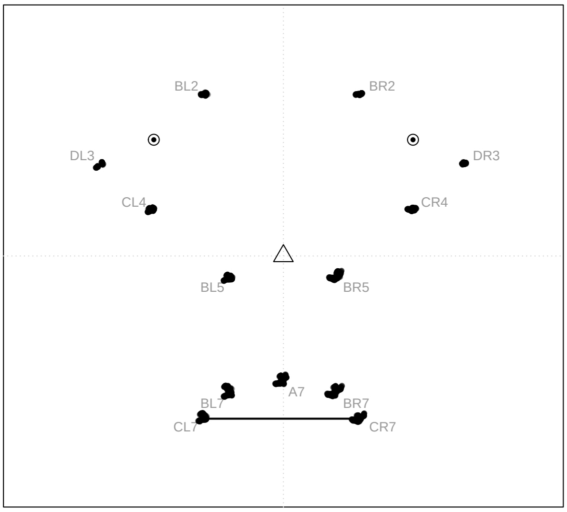

Participant 8 posing anger

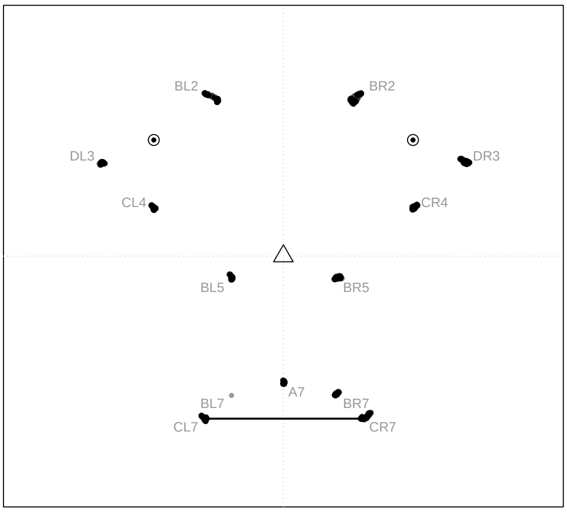

Participant 8 posing fear

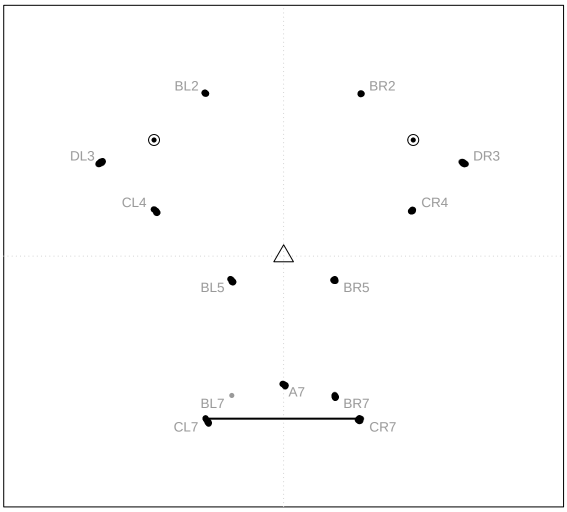

Participant 8 posing disgust

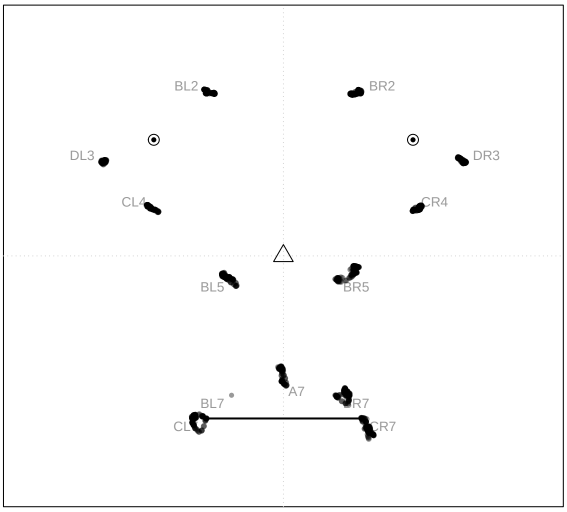

Participant 8 posing happiness

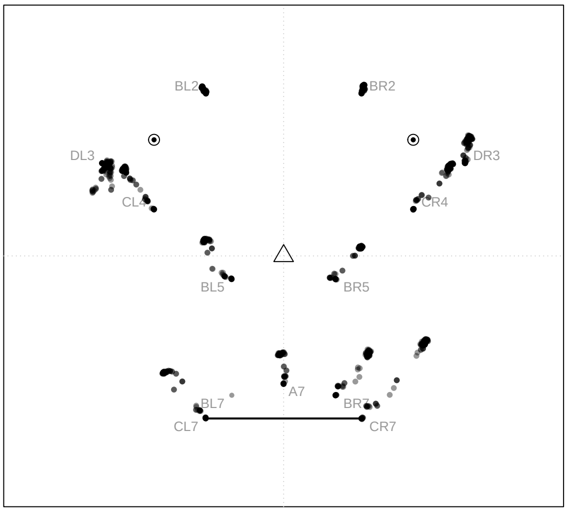

Participant 8 posing sadness

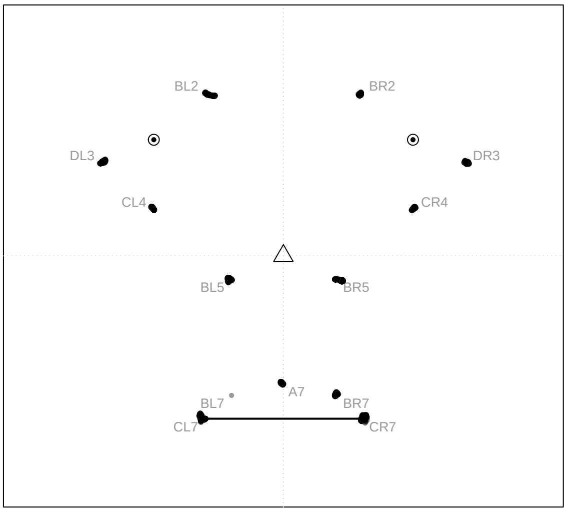

Participant 14 posing anger

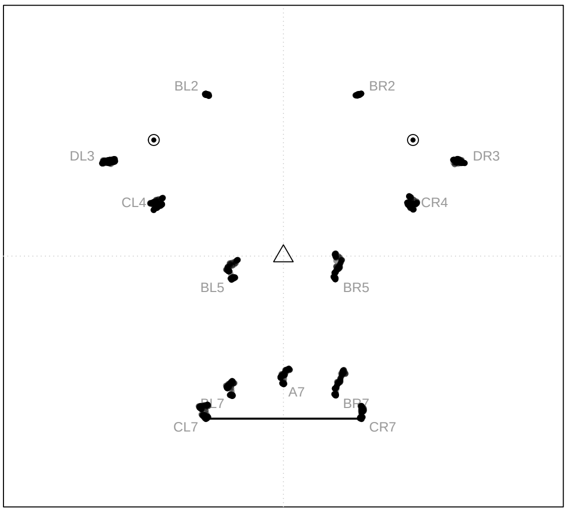

Participant 14 posing fear

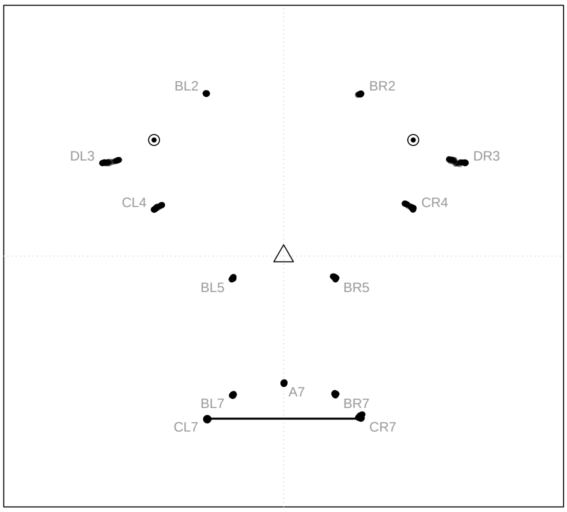

Participant 14 posing disgust

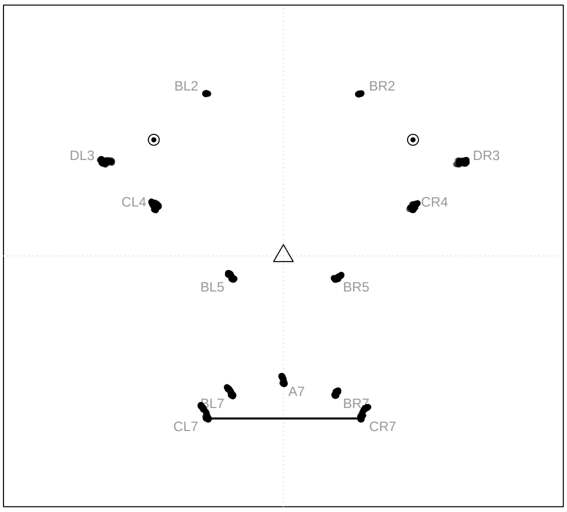

Participant 14 posing happiness

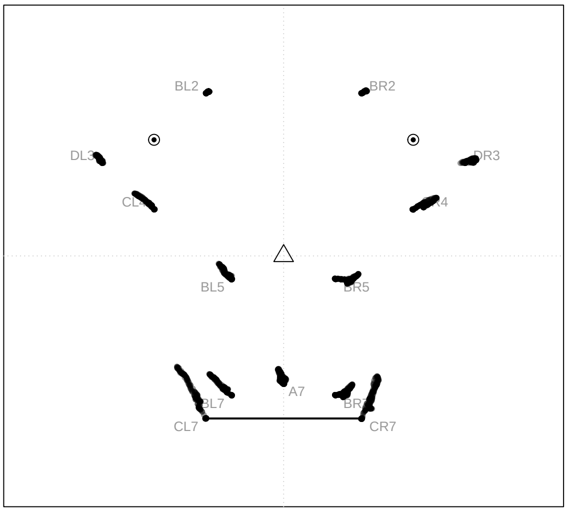

Participant 14 posing sadness

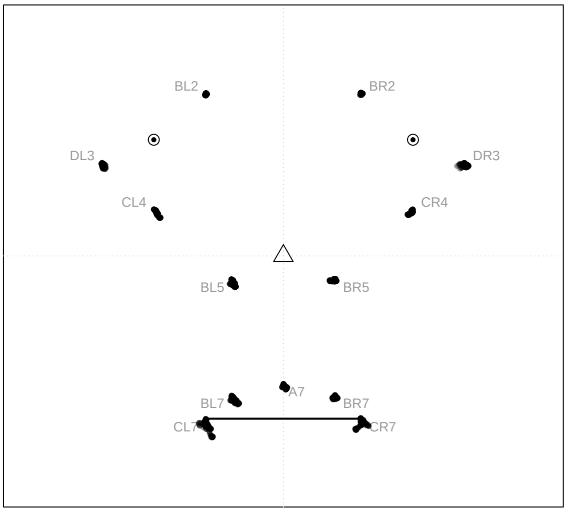

Participant 15 posing anger

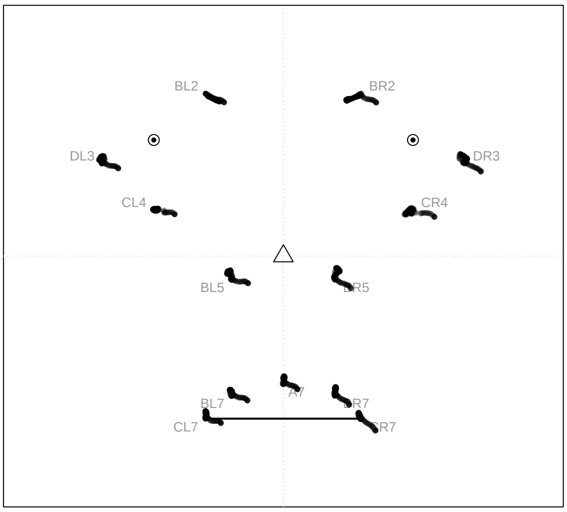

Participant 15 posing fear

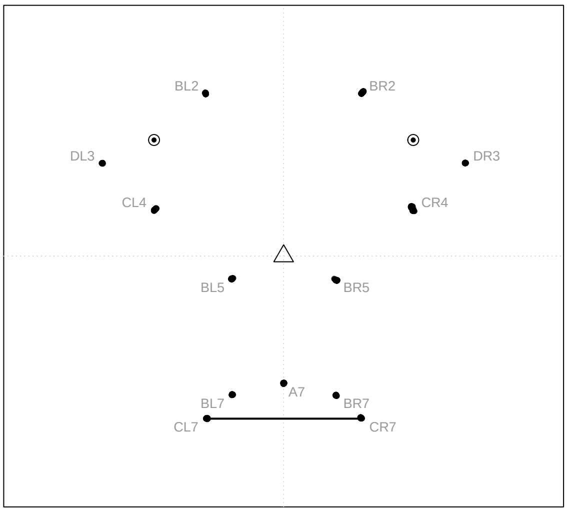

Participant 15 posing disgust

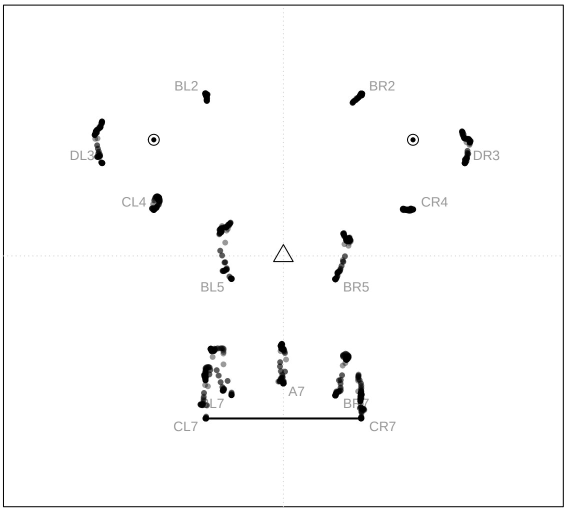

Participant 15 posing happiness

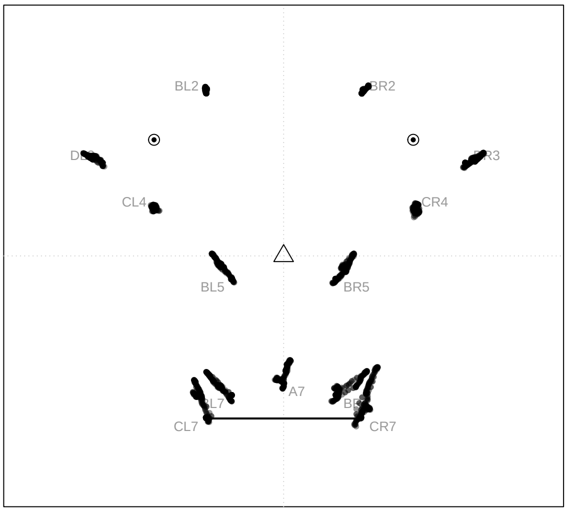

Participant 15 posing sadness

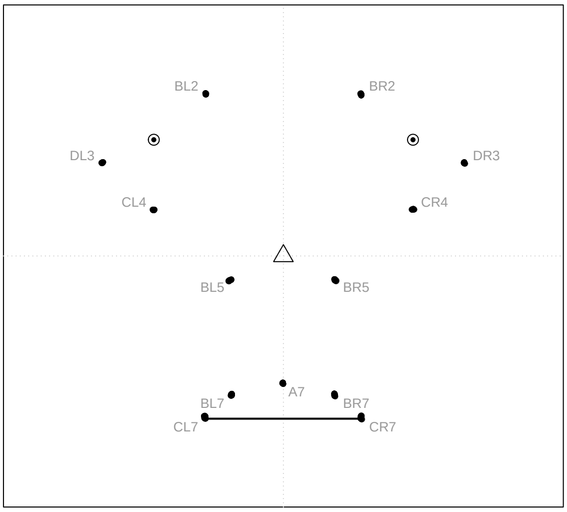

Participant 17 posing anger

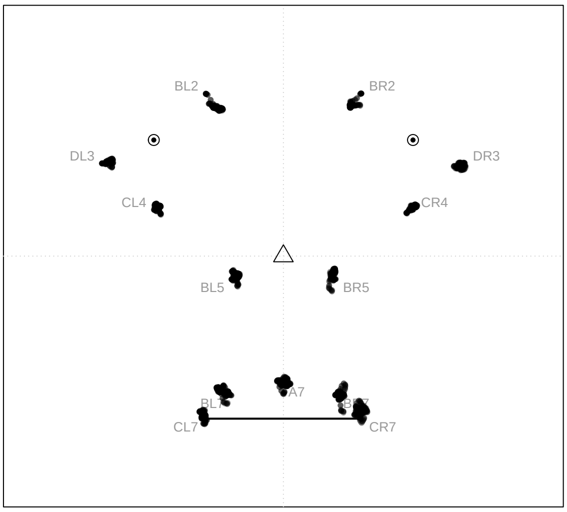

Participant 17 posing fear

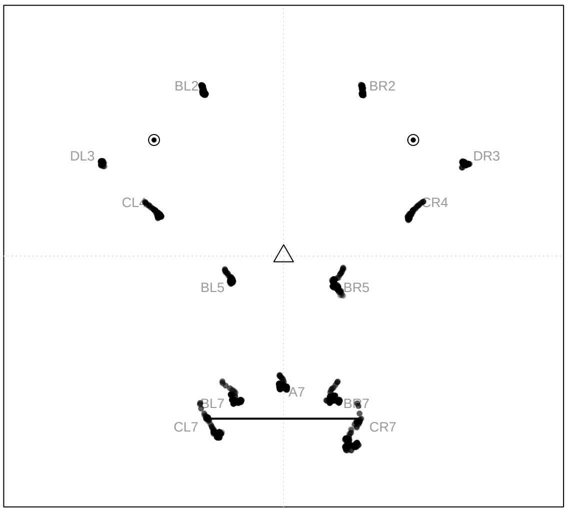

Participant 17 posing disgust

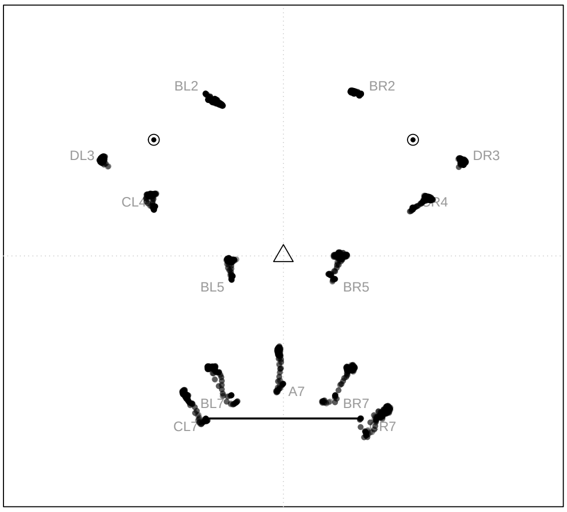

Participant 17 posing happiness

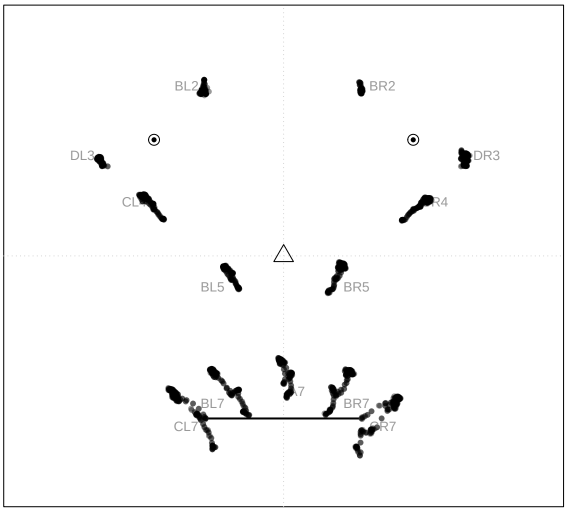

Participant 17 posing sadness

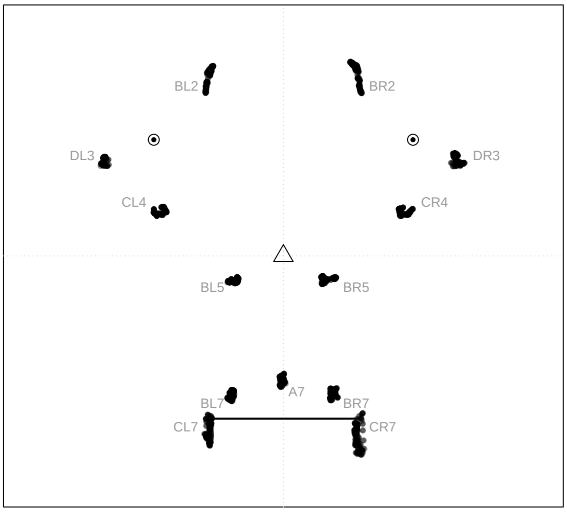

Participant 18 posing anger

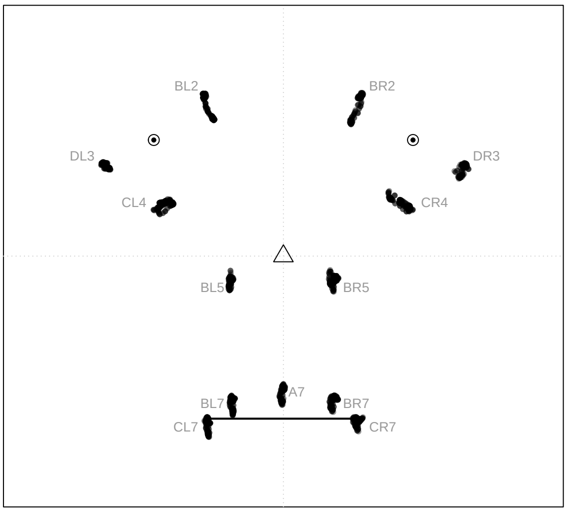

Participant 18 posing fear

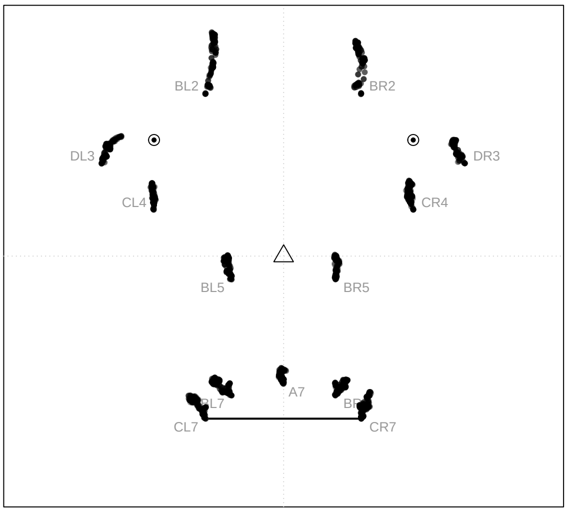

Participant 18 posing disgust

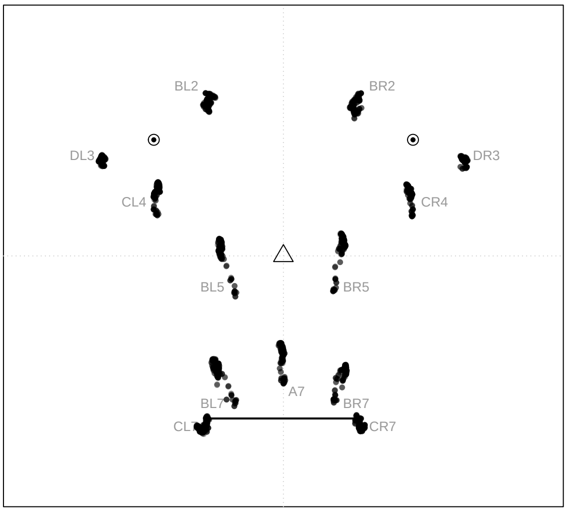

Participant 18 posing happiness

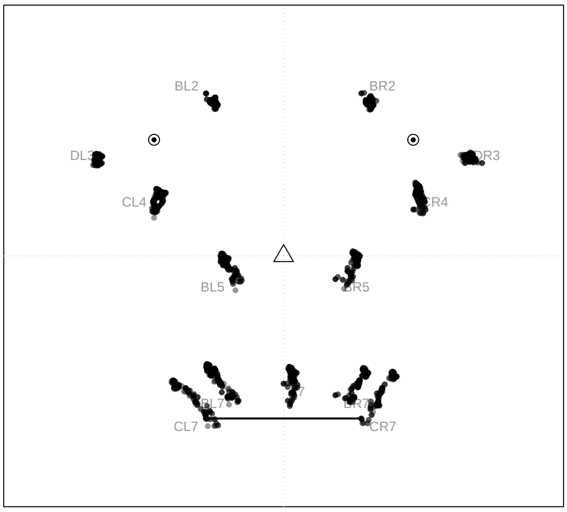

Participant 18 posing sadness

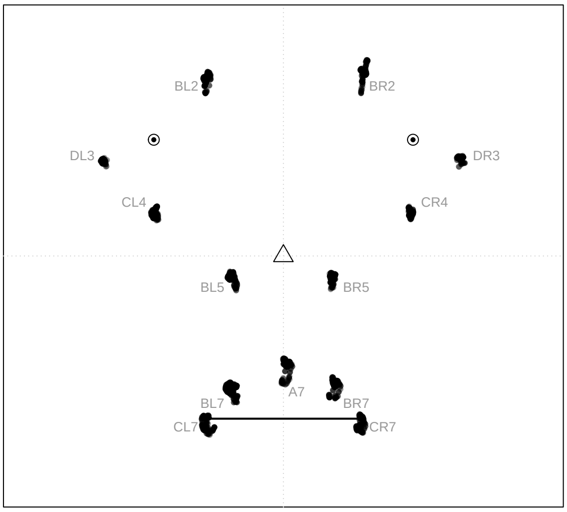

Participant 19 posing anger

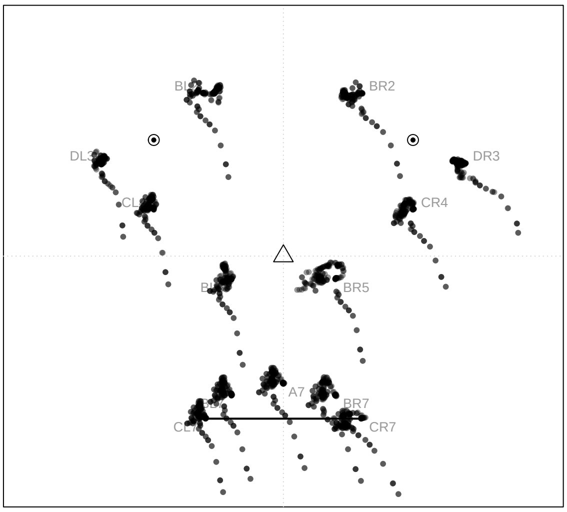

Participant 19 posing fear

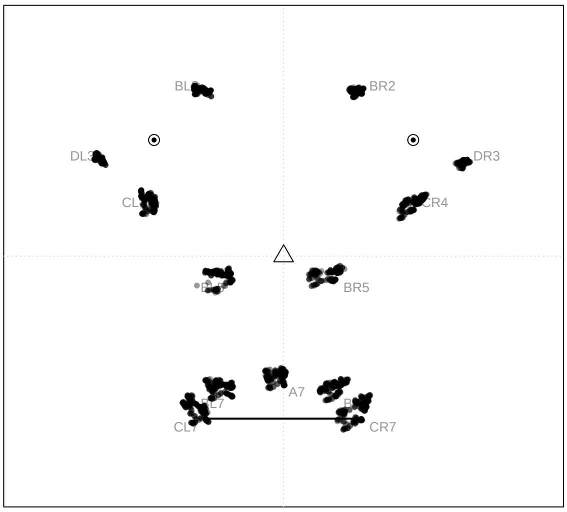

Participant 19 posing disgust

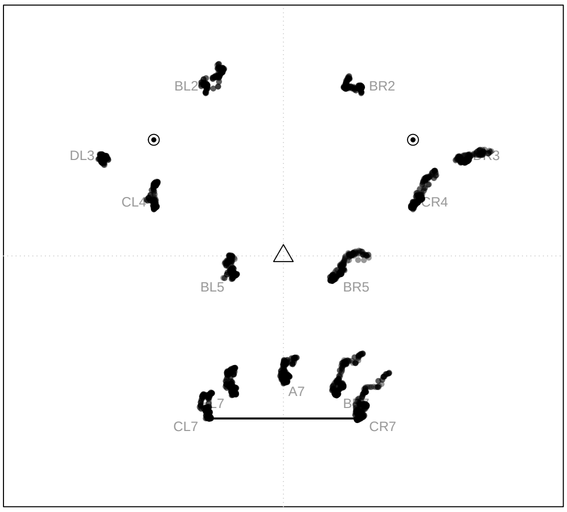

Participant 19 posing happiness

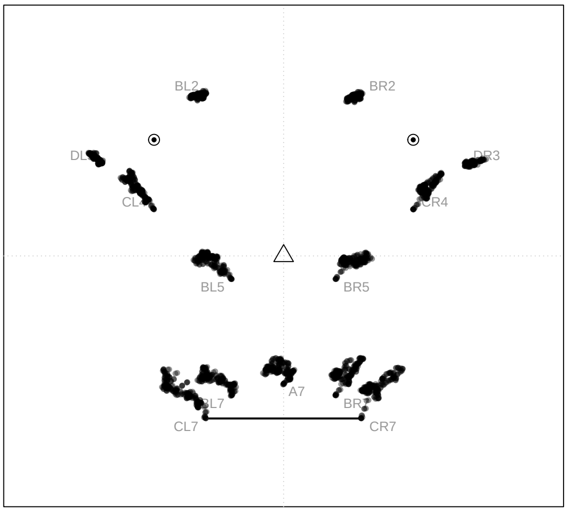

Participant 19 posing sadness

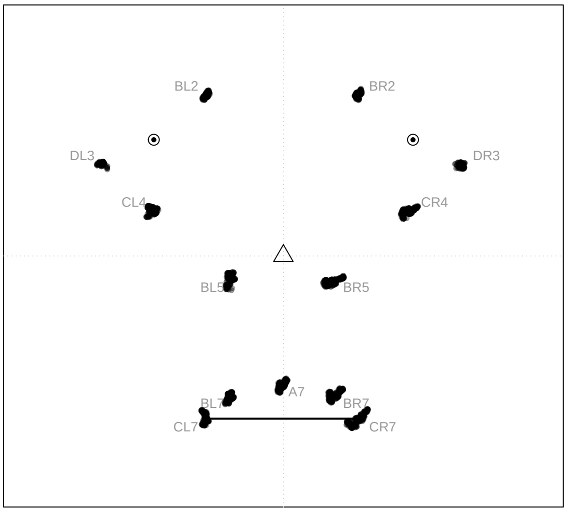

Participant 20 posing anger

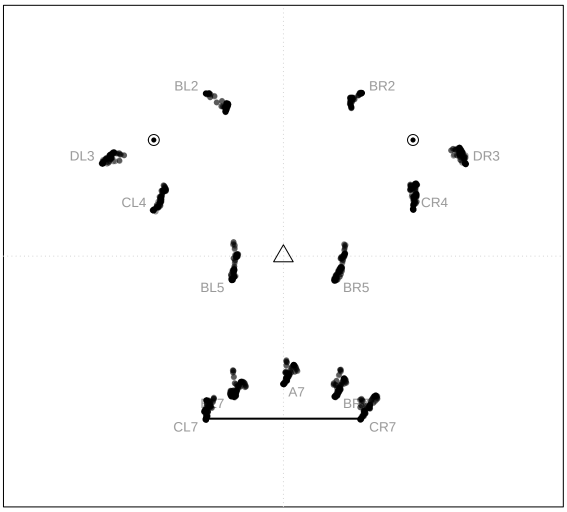

Participant 20 posing fear

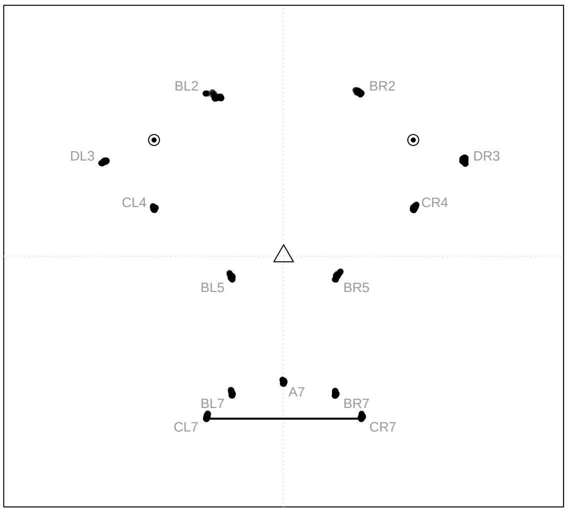

Participant 20 posing disgust

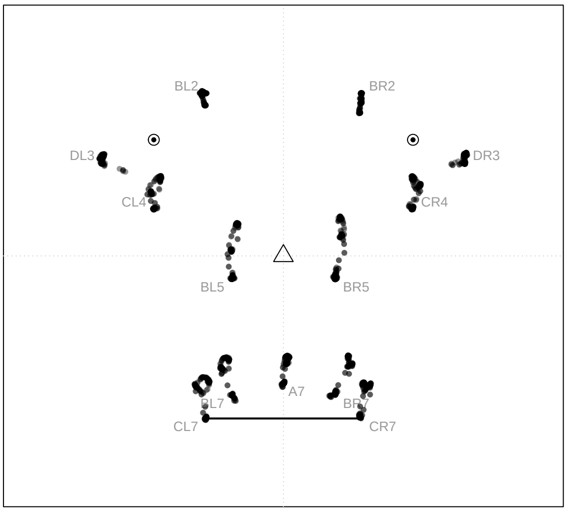

Participant 20 posing happiness

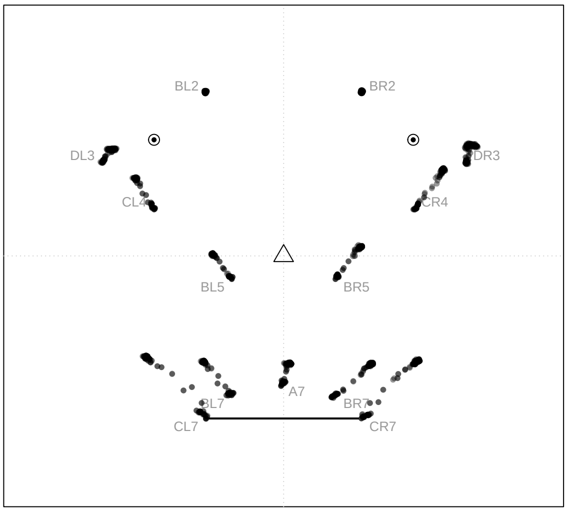

Participant 20 posing sadness

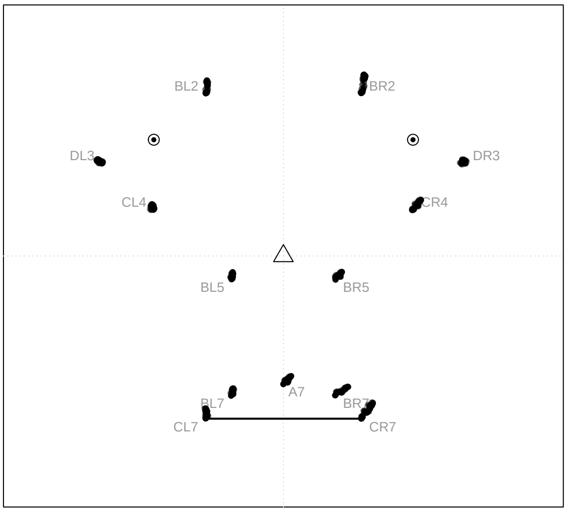

Participant 21 posing anger

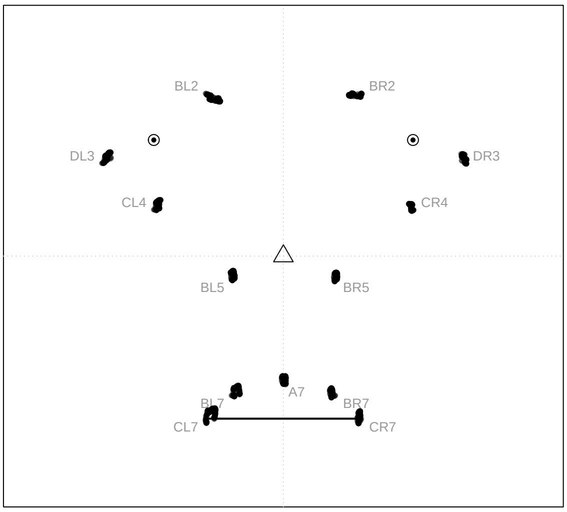

Participant 21 posing fear

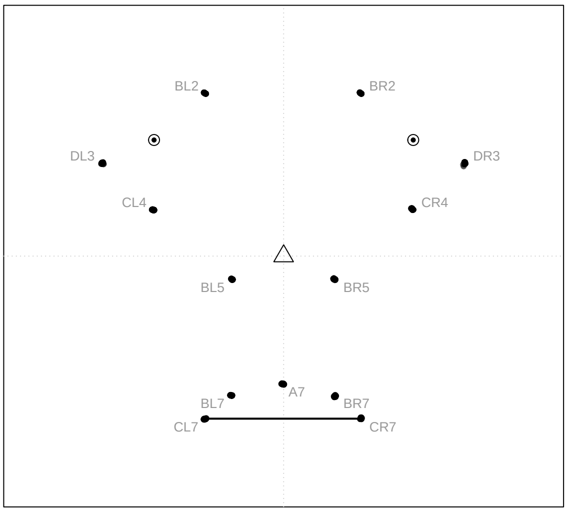

Participant 21 posing disgust

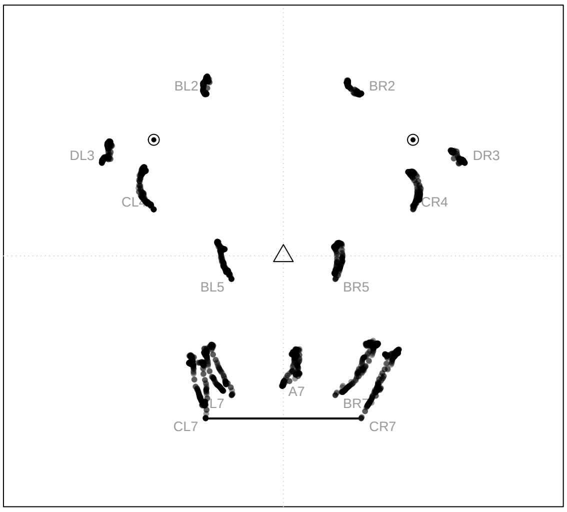

Participant 21 posing happiness

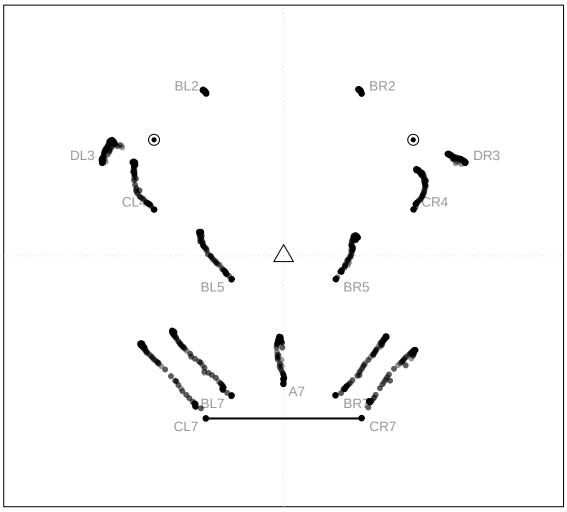

Participant 21 posing sadness

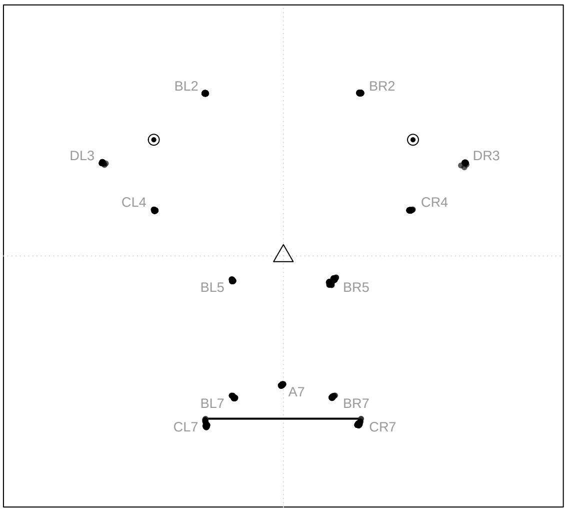

Participant 23 posing anger

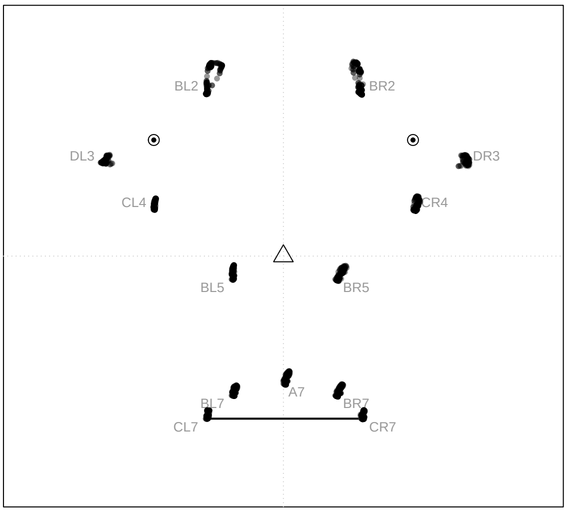

Participant 23 posing fear

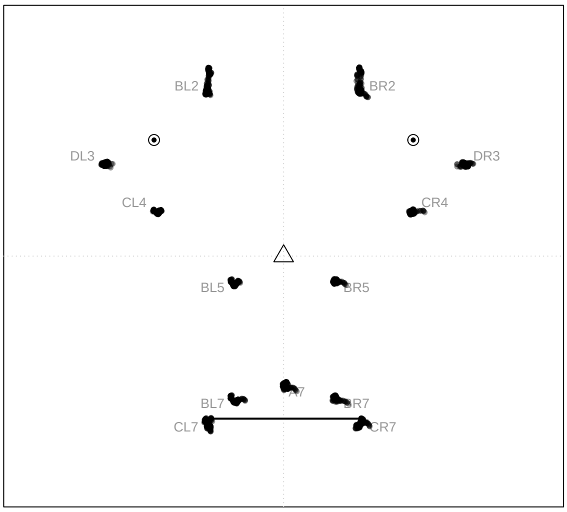

Participant 23 posing disgust

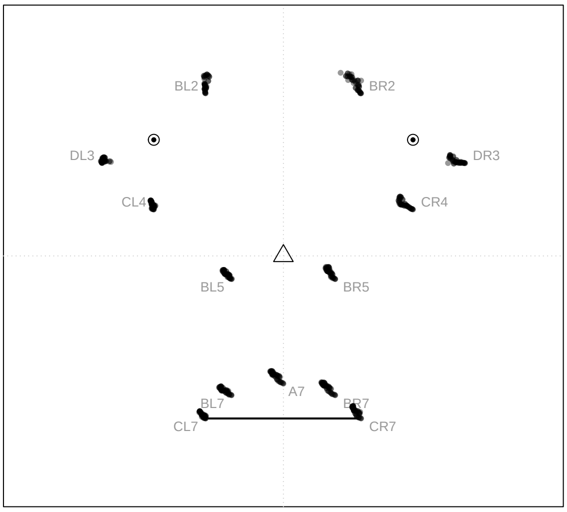

Participant 23 posing happiness

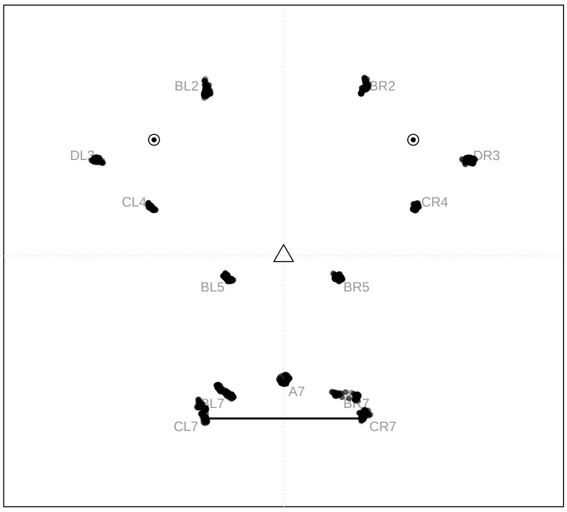

Participant 23 posing sadness

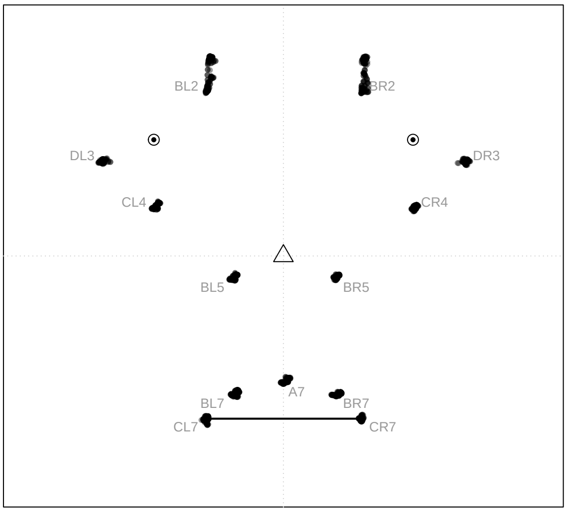

Participant 24 posing anger

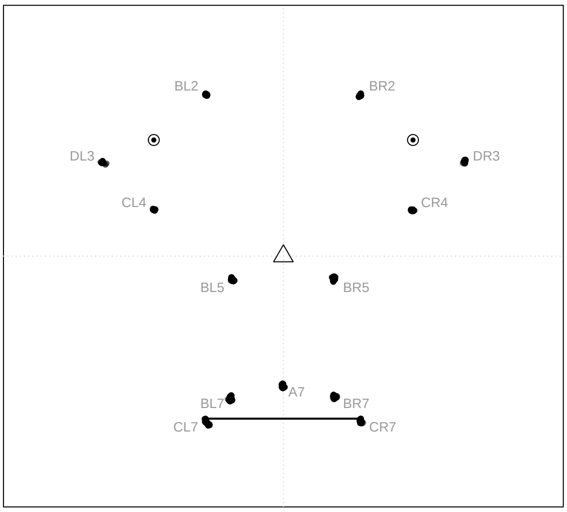

Participant 24 posing fear

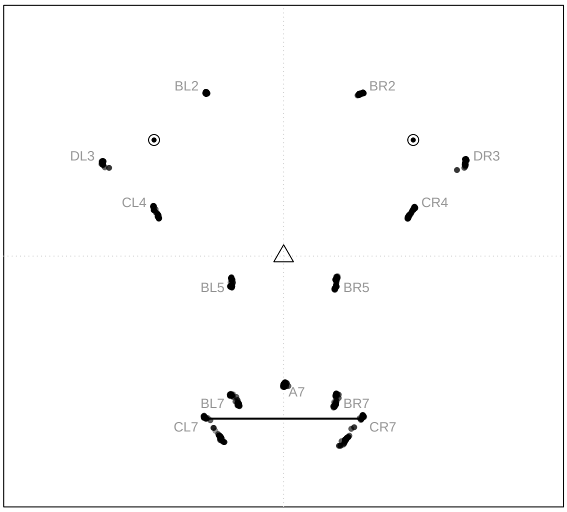

Participant 24 posing disgust

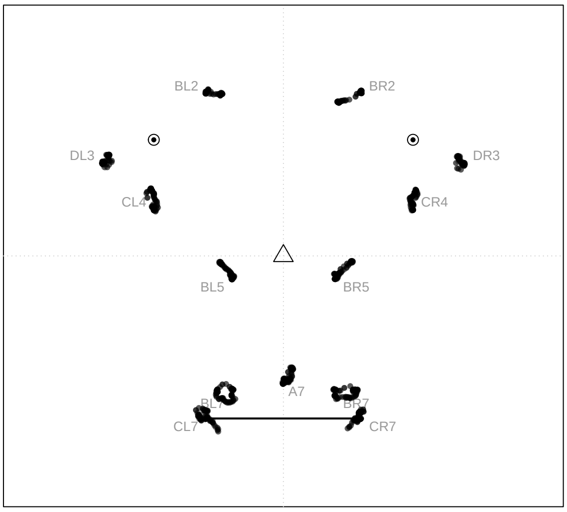

Participant 24 posing happiness

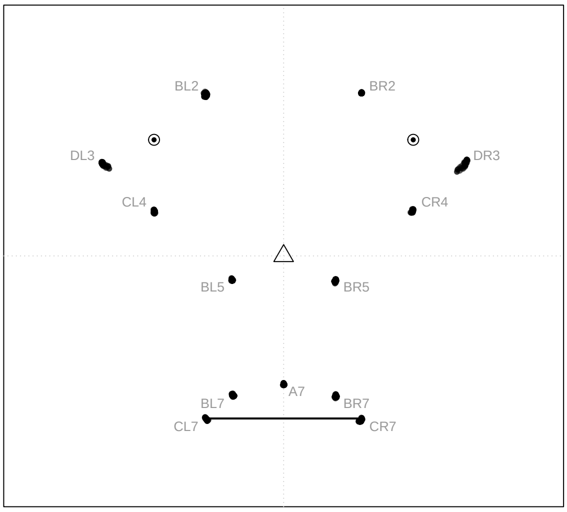

Participant 24 posing sadness

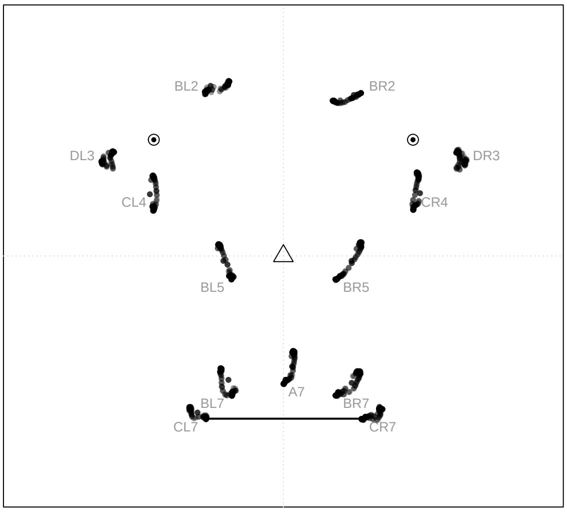

Participant 25 posing anger

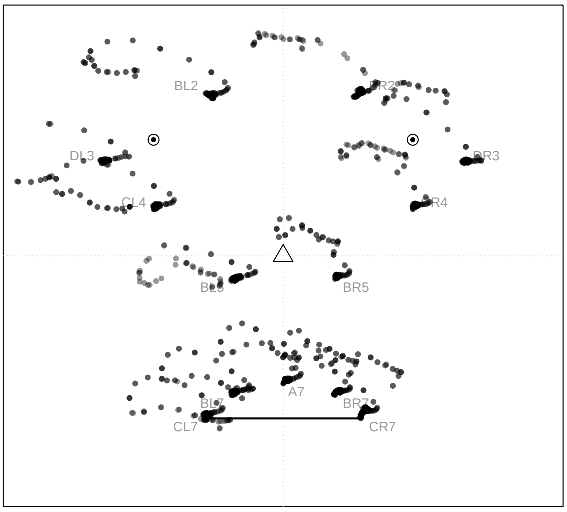

Participant 25 posing fear

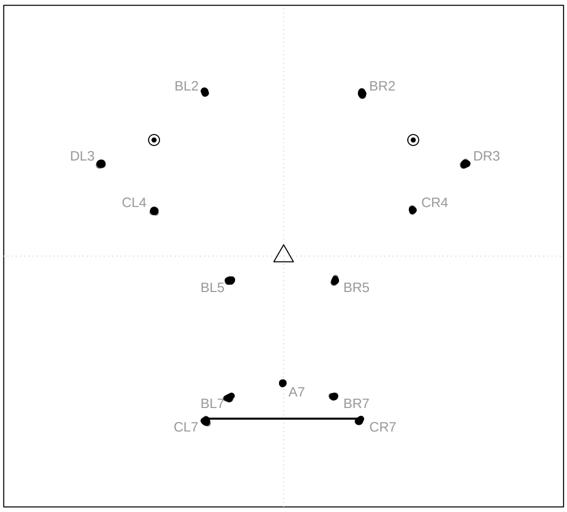

Participant 25 posing disgust

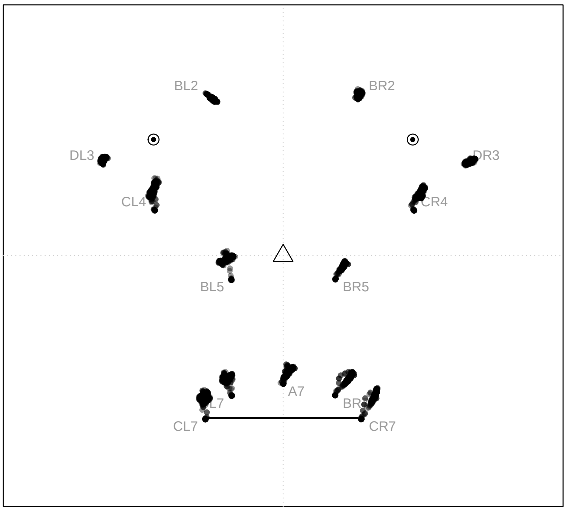

Participant 25 posing happiness

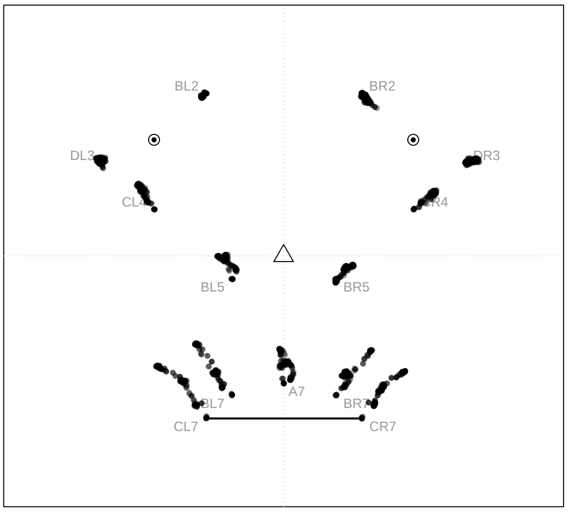

Participant 25 posing sadness

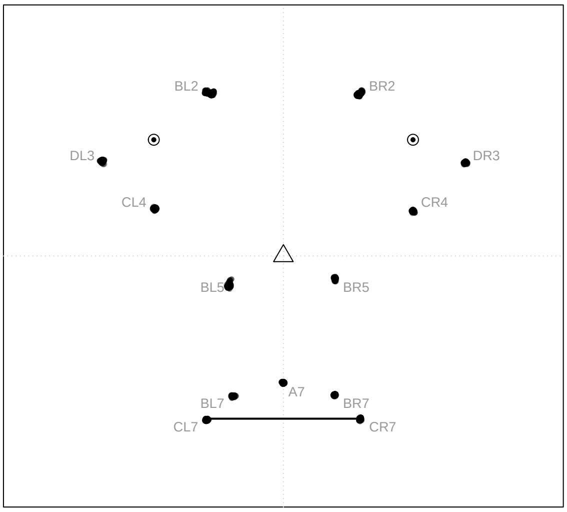

Participant 26 posing anger

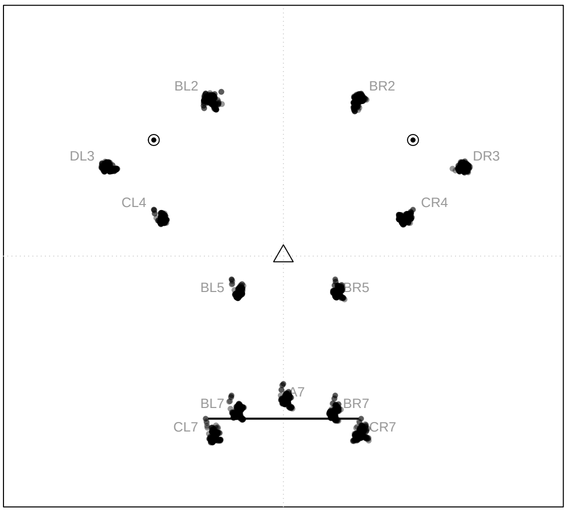

Participant 26 posing fear

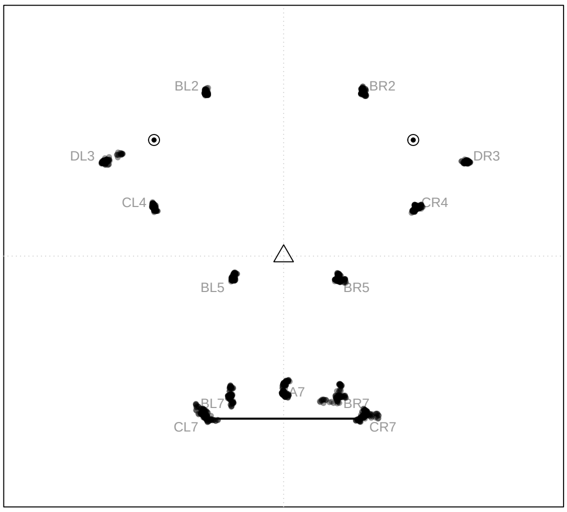

Participant 26 posing disgust

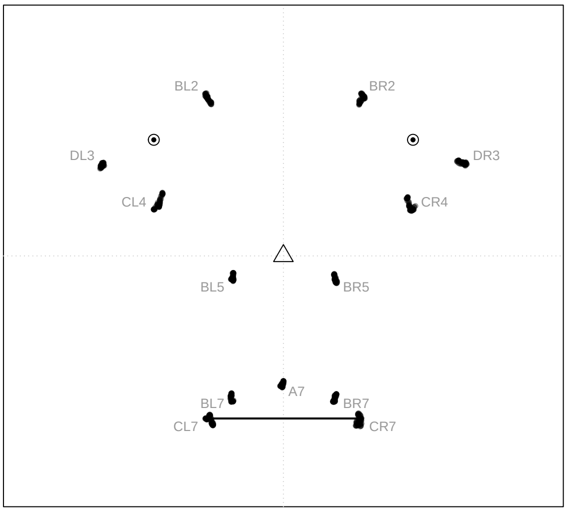

Participant 26 posing happiness

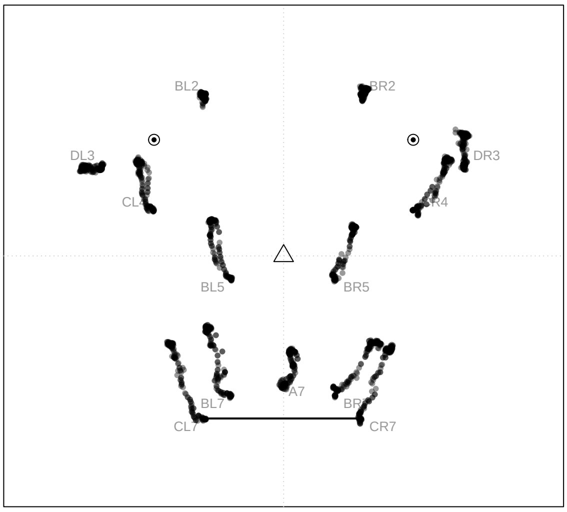

Participant 26 posing sadness

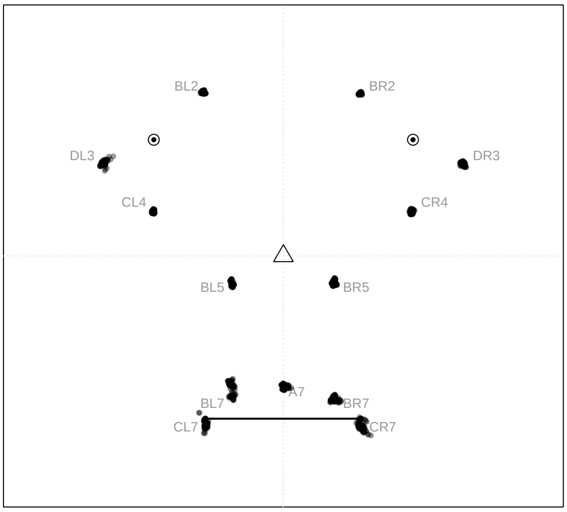

Participant 30 posing anger

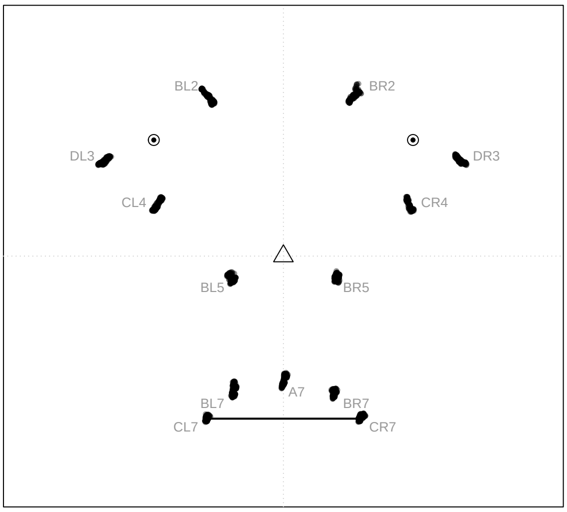

Participant 30 posing fear

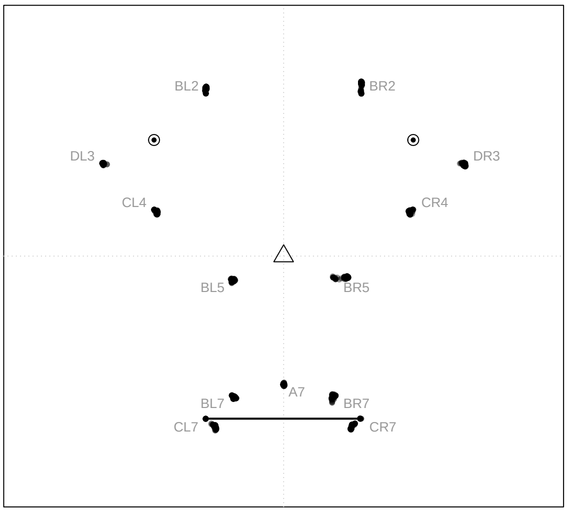

Participant 30 posing disgust

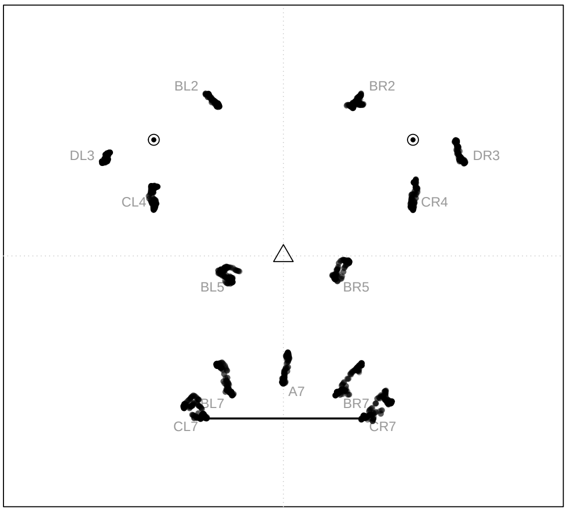

Participant 30 posing happiness

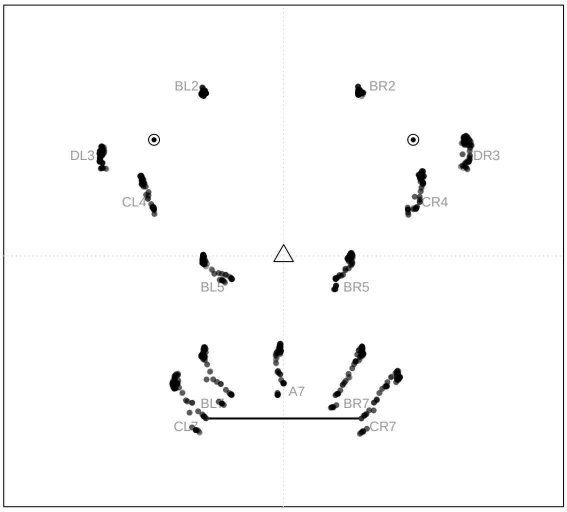

Participant 30 posing sadness

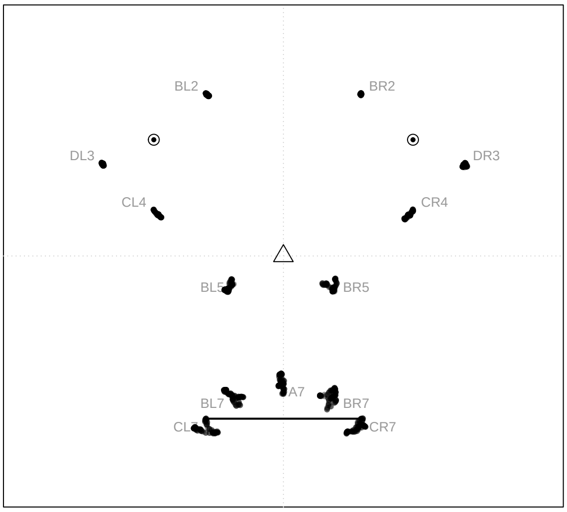

Participant 31 posing anger

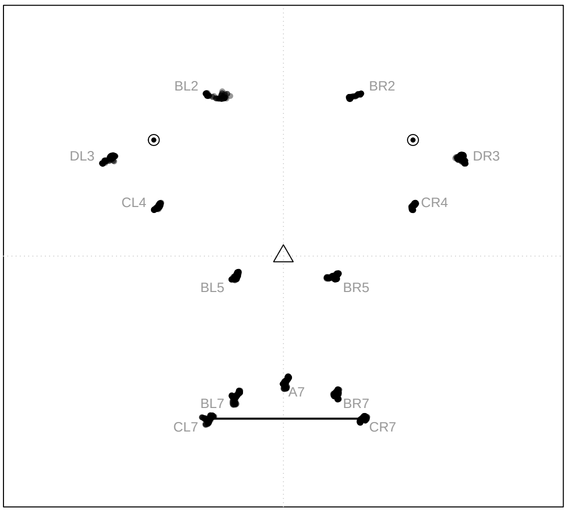

Participant 31 posing fear

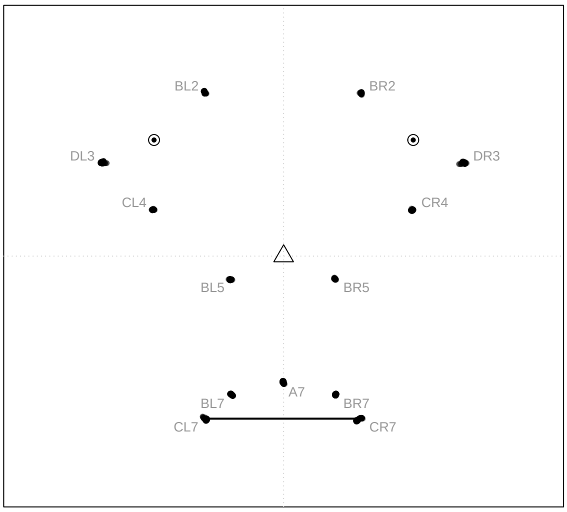

Participant 31 posing disgust

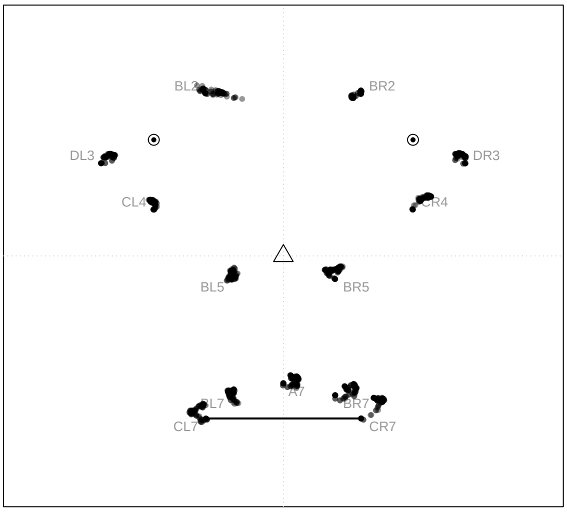

Participant 31 posing happiness

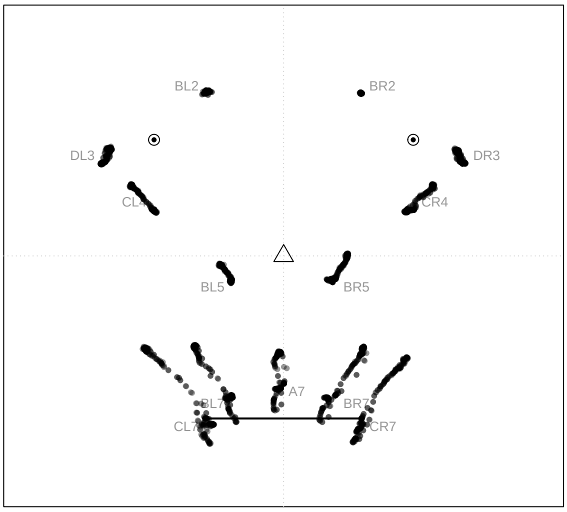

Participant 31 posing sadness

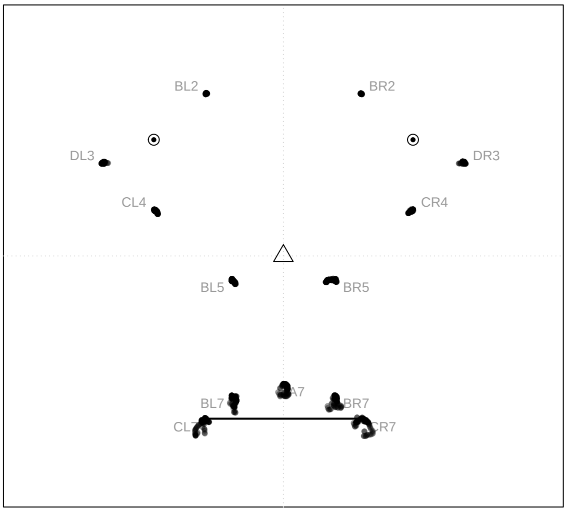

Supplement: Supplementary file 3 [file PSYP-63-e70394-s001.zip › Study 2/Plots/01_Descriptive_Plots/01_Xhead.pdf]
