# Supplementary material for: blenderFaceUV: Toward Marker‐Based Precision and Flexibility in Facial Tracking With “Invisible” Markers
Source: Psychophysiology. 2026 Sep 21;63(9):e70394. doi: 10.1111/psyp.70394 (PMC13594373; doi:10.1111/psyp.70394)
Supplement: Supplementary file 3 [file PSYP-63-e70394-s001.zip › Study 2/Plots/01_Descriptive_Plots/02_IndMM.pdf]

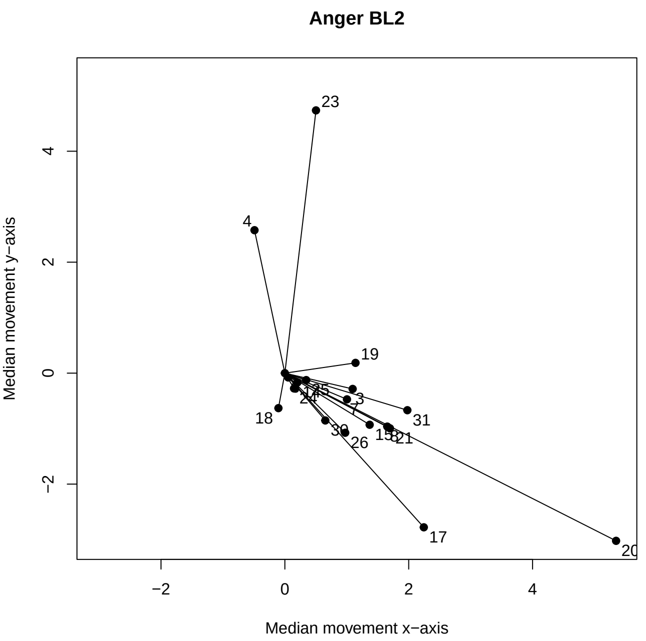
Anger BL2
Median movement y−axis
Median movement x−axis

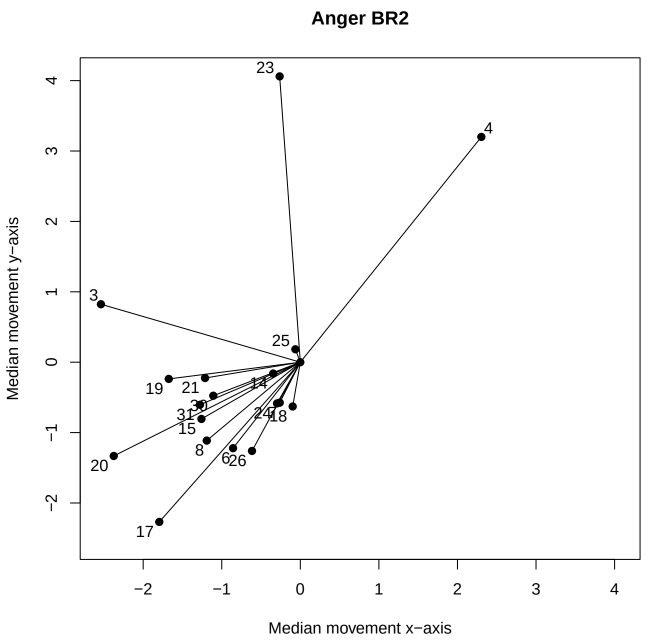
Anger BR2
Median movement y−axis
Median movement x−axis

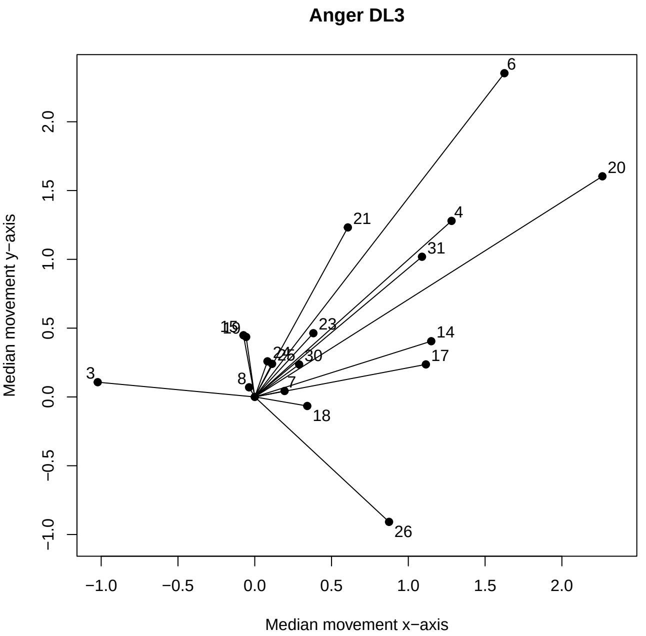
Anger DL3
Median movement y−axis
Median movement x−axis

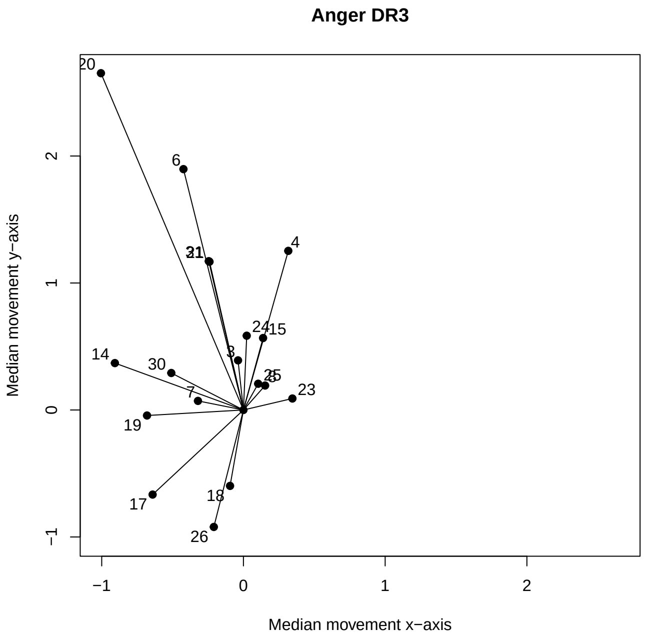
Anger DR3
Median movement y−axis
Median movement x−axis

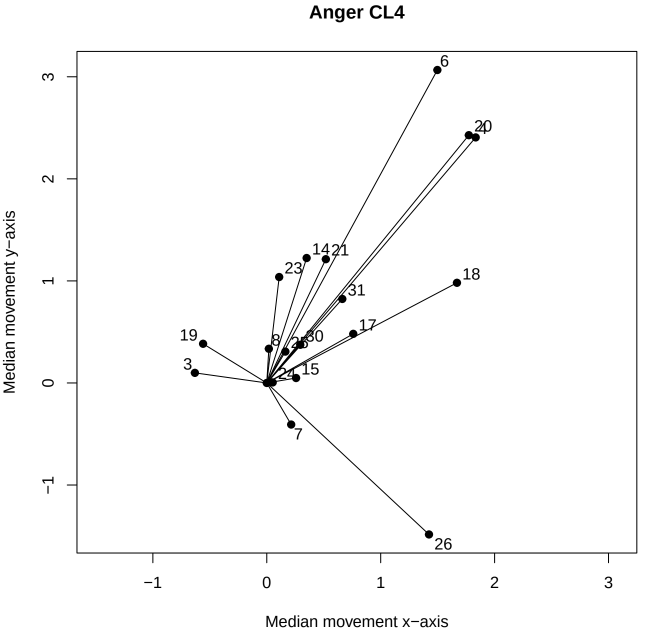
Anger CL4
Median movement y−axis
Median movement x−axis

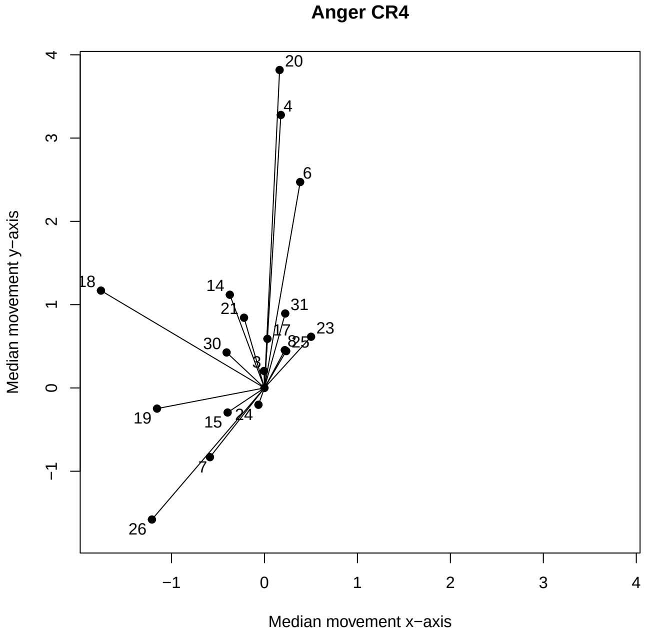
Anger CR4
Median movement y−axis
Median movement x−axis

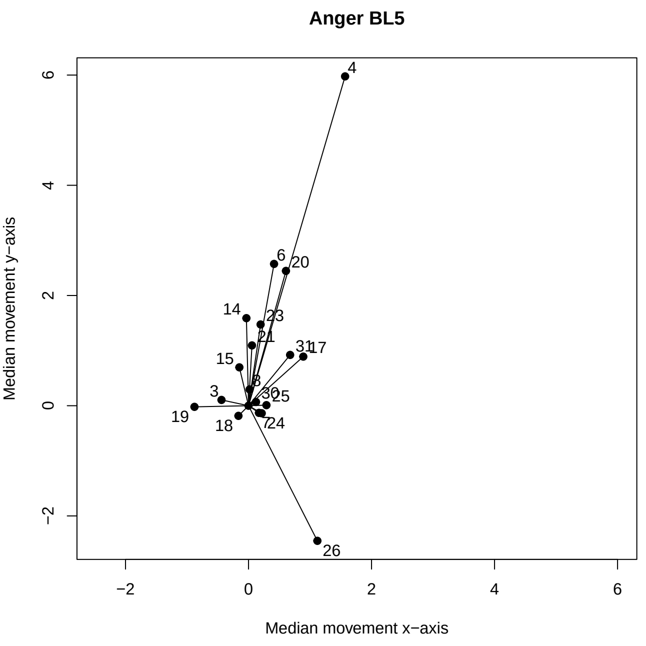
Anger BL5
Median movement y−axis
Median movement x−axis

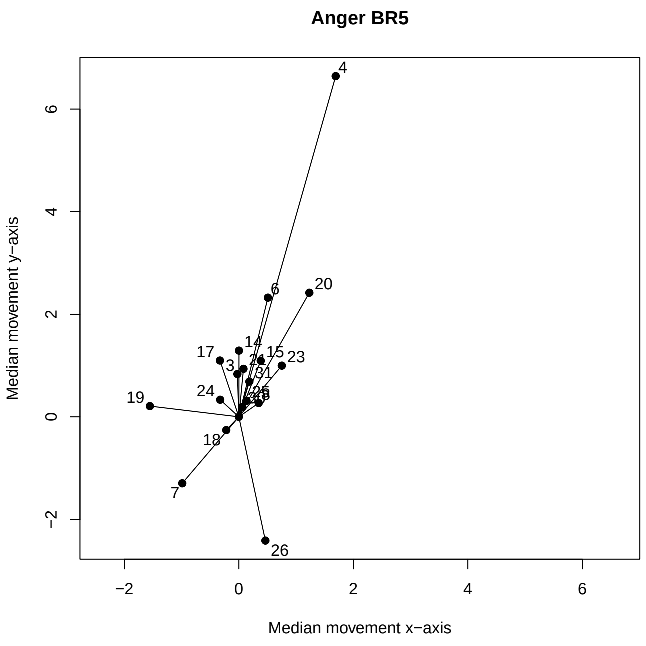
Anger BR5
Median movement y−axis
Median movement x−axis

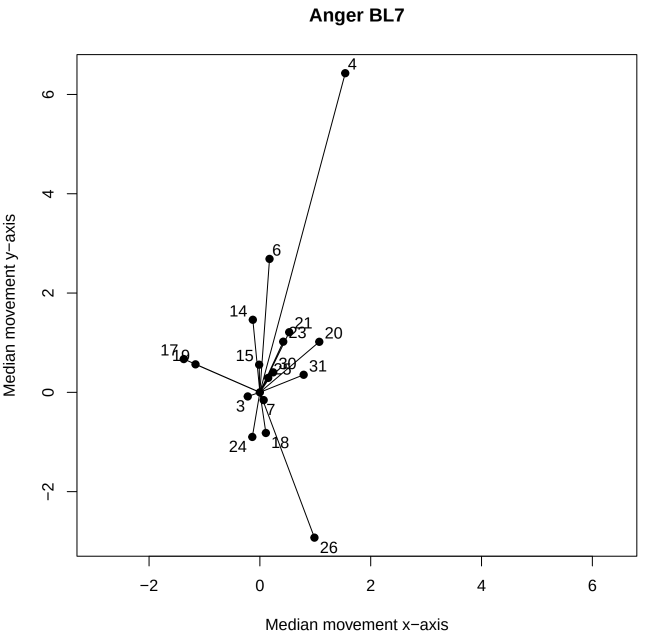
Anger BL7
Median movement y−axis
Median movement x−axis

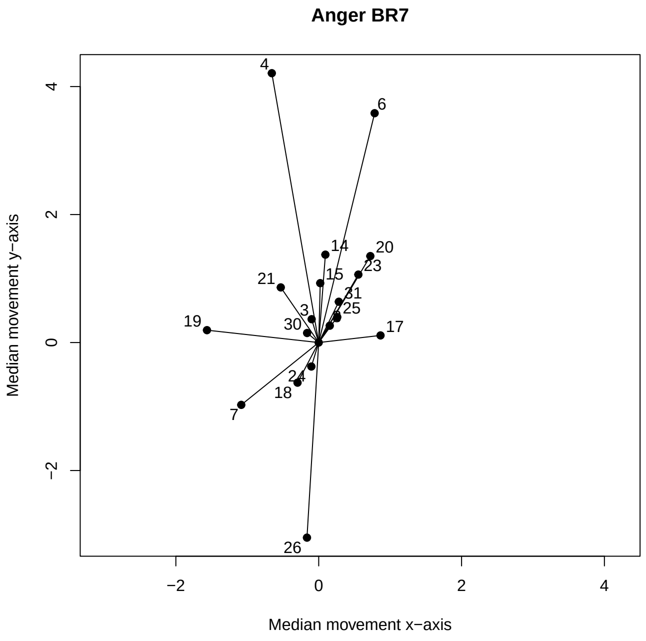
Anger BR7
Median movement y−axis
Median movement x−axis

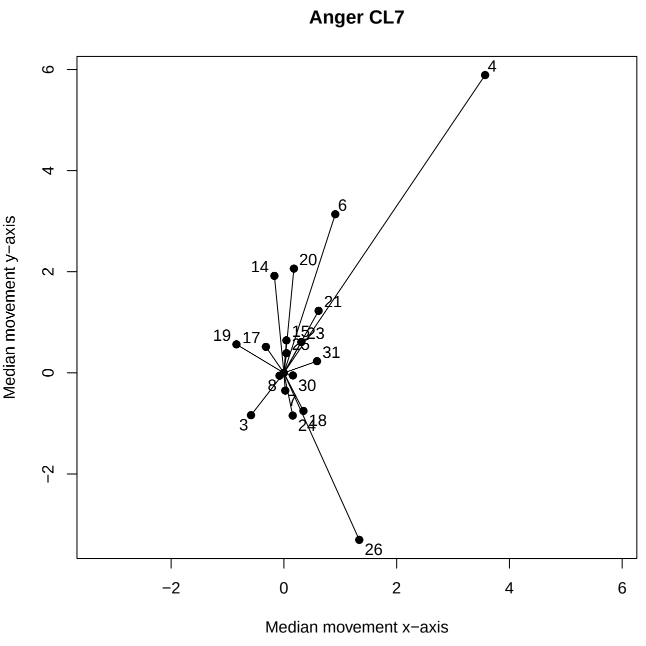
Anger CL7
Median movement y−axis
Median movement x−axis

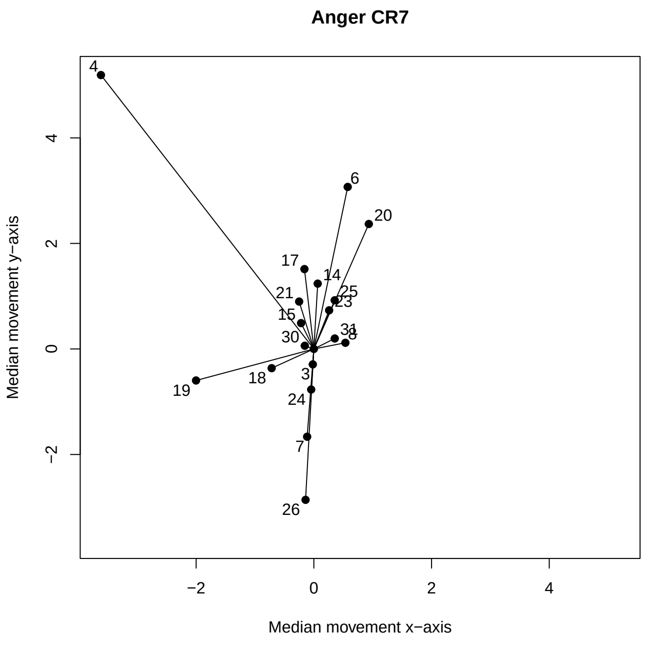
Anger CR7
Median movement y−axis
Median movement x−axis

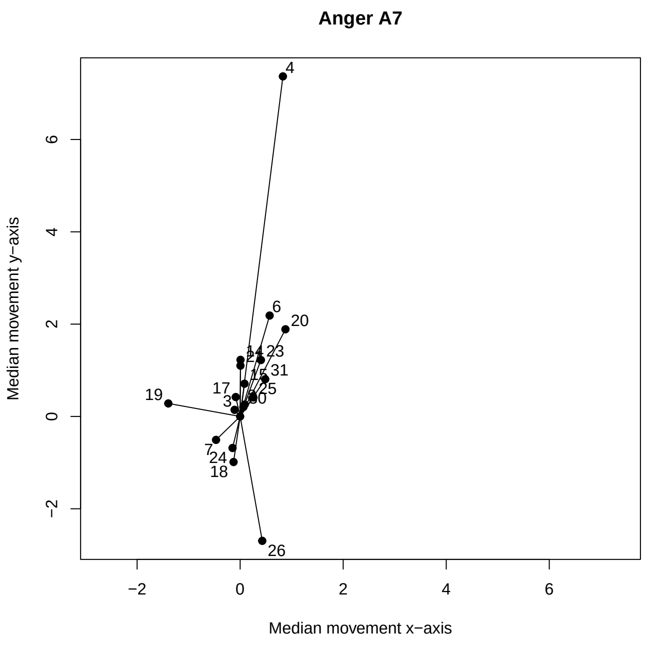

Anger A7
Median movement y−axis
Median movement x−axis
−2
0
2
4
6
4
6
20
14
23
31
17
25
19
3
7
24
18
26

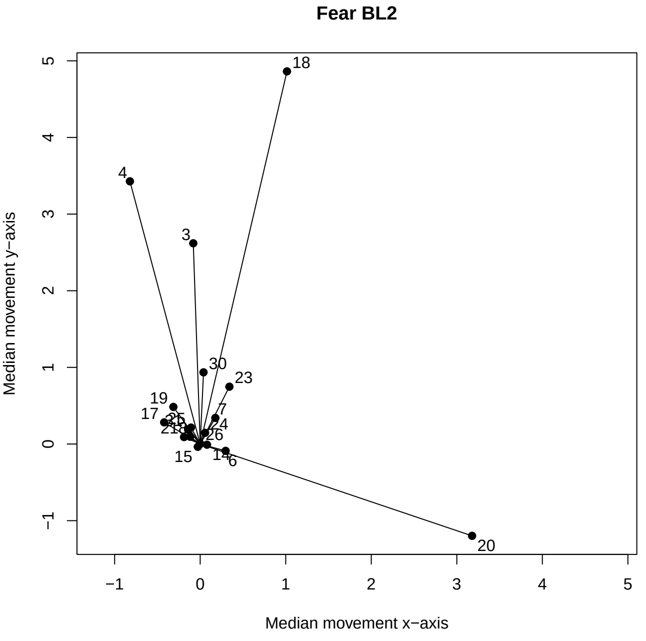
Fear BL2
Median movement x−axis
Median movement y−axis

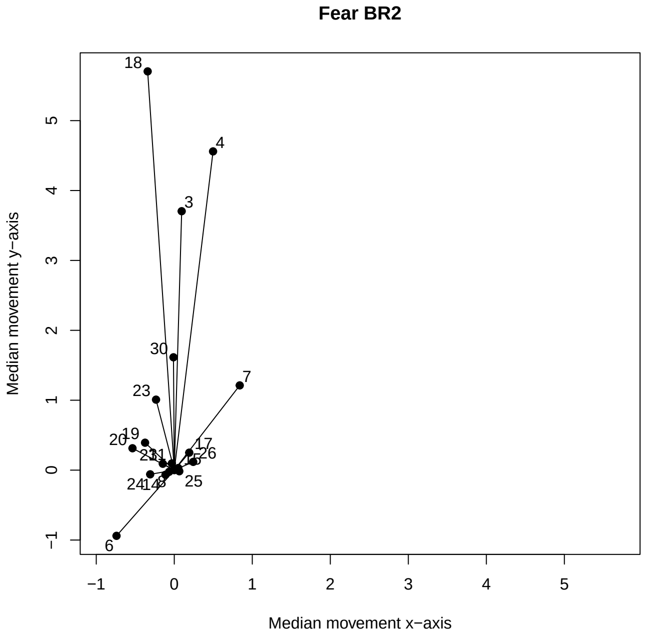
Fear BR2
Median movement x−axis
Median movement y−axis

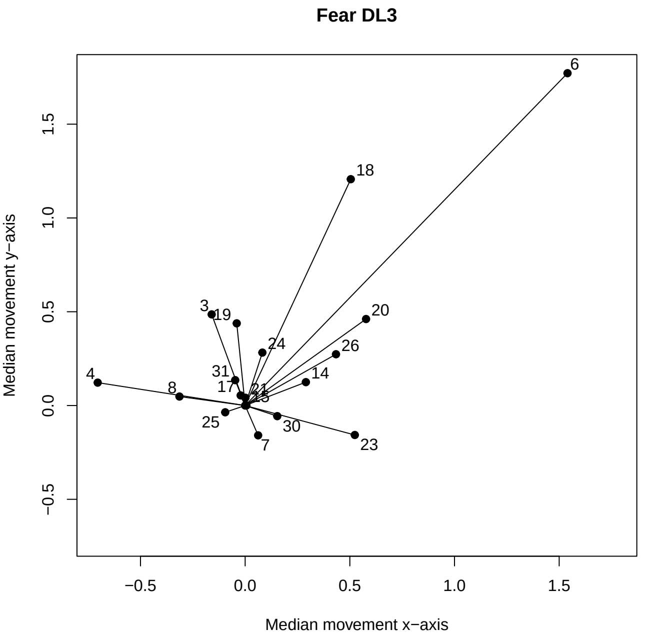
Fear DL3
Median movement x−axis
Median movement y−axis

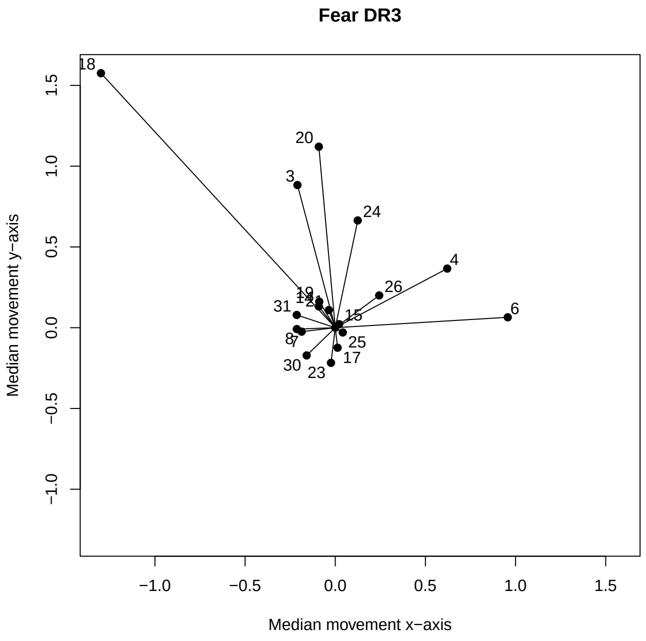
Fear DR3
Median movement x−axis
Median movement y−axis

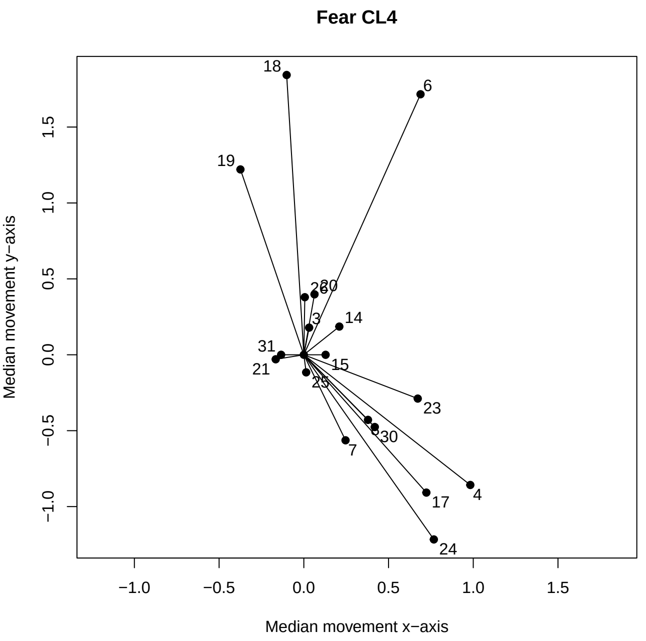
Fear CL4
Median movement x−axis
Median movement y−axis

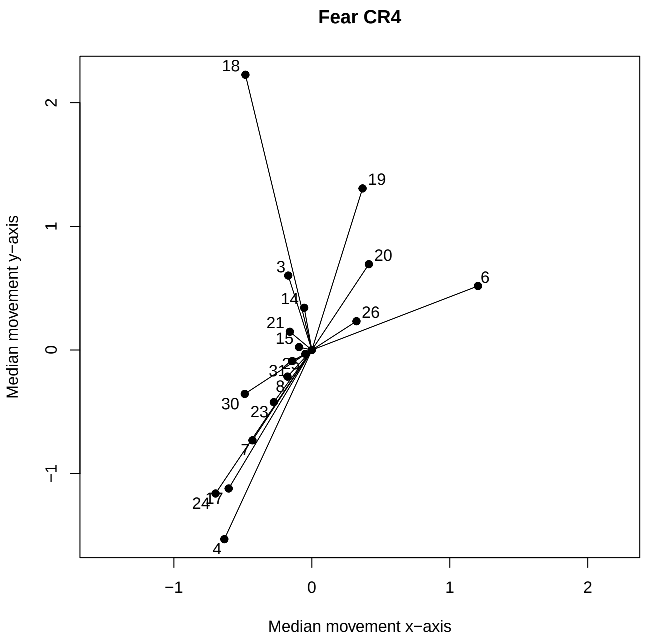
Fear CR4
Median movement x−axis
Median movement y−axis

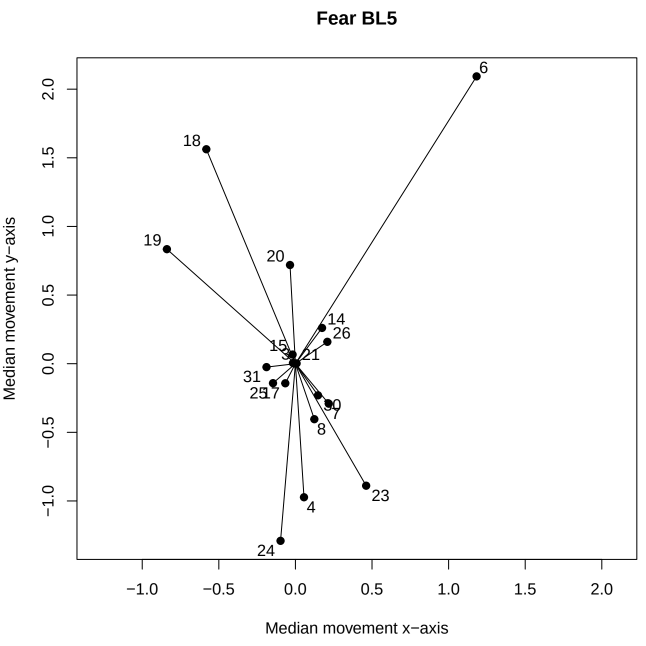
Fear BL5
Median movement x−axis
Median movement y−axis

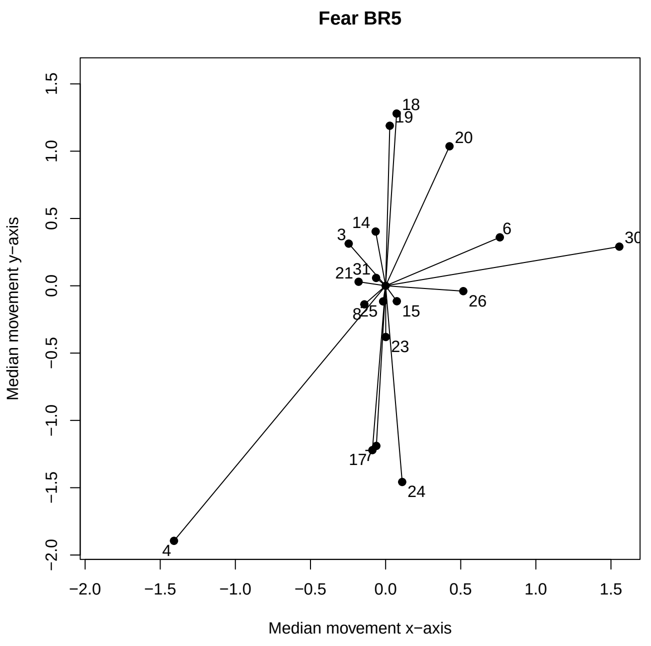
Fear BR5
Median movement x−axis
Median movement y−axis

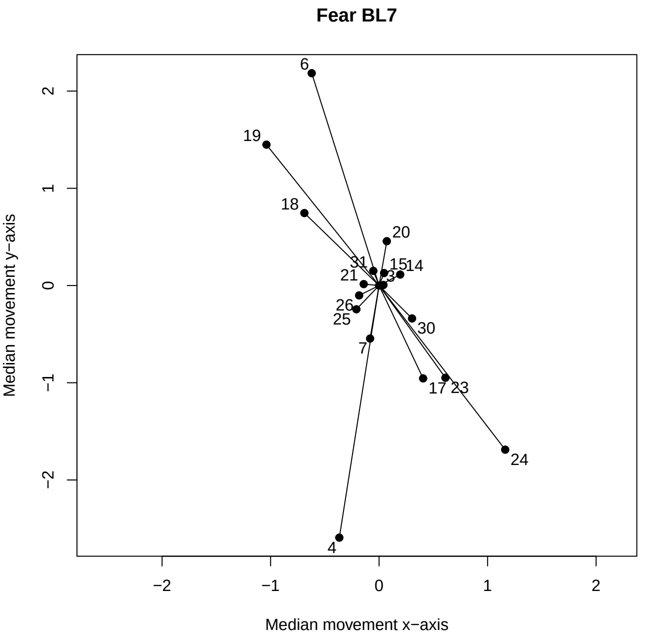
Fear BL7
Median movement x−axis
Median movement y−axis

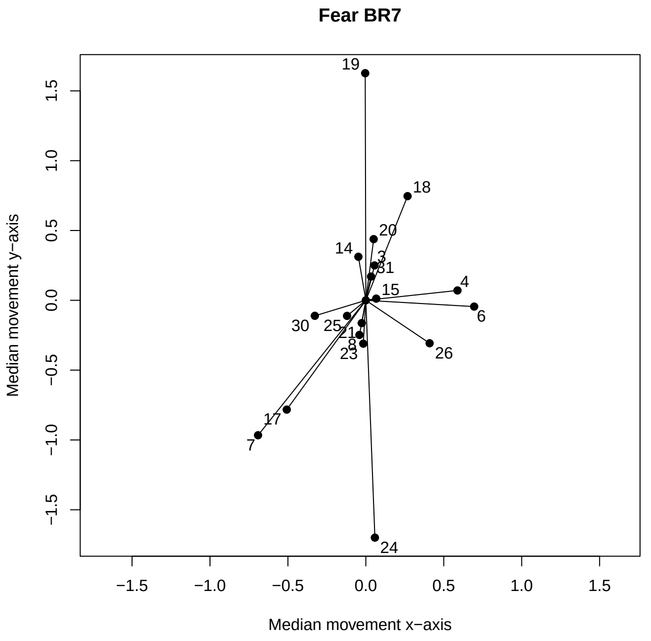
Fear BR7
Median movement x−axis
Median movement y−axis

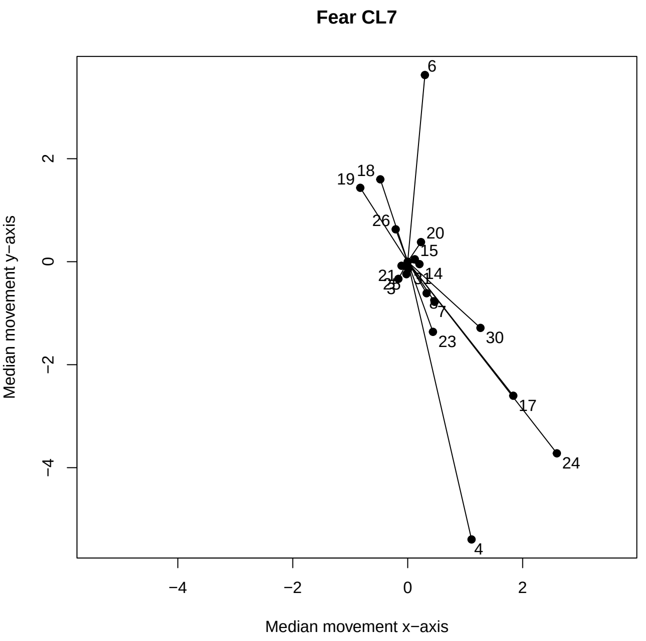
Fear CL7
Median movement x−axis
Median movement y−axis

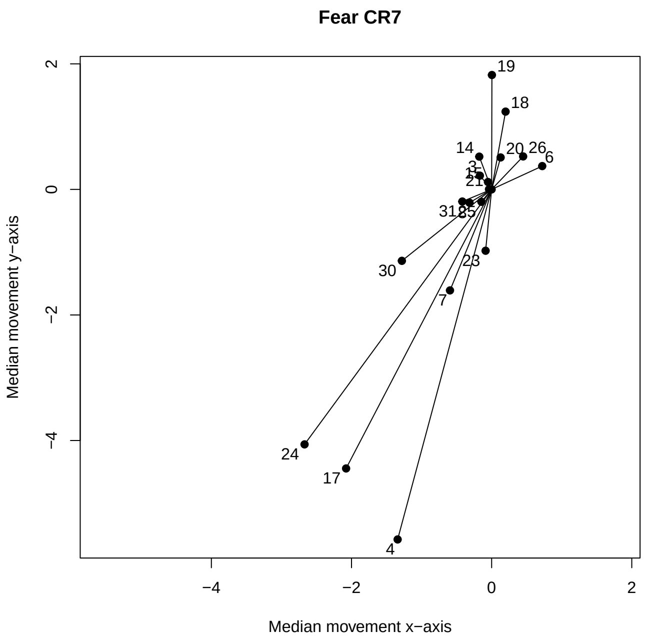
Fear CR7
Median movement x−axis
Median movement y−axis

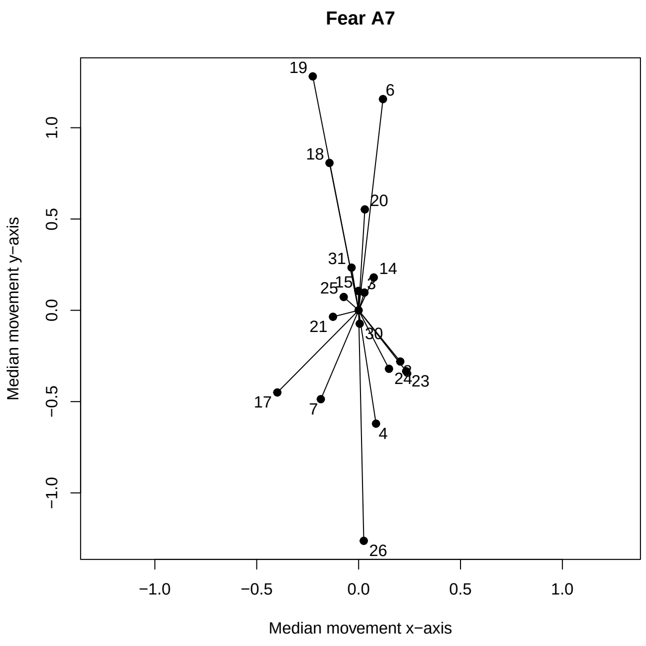

Fear A7
Median movement y−axis
Median movement x−axis
−1.0
−0.5
0.0
0.5
1.0
19
6
18
20
31
14
15
25
3
21
30
24
23
17
7
4
26

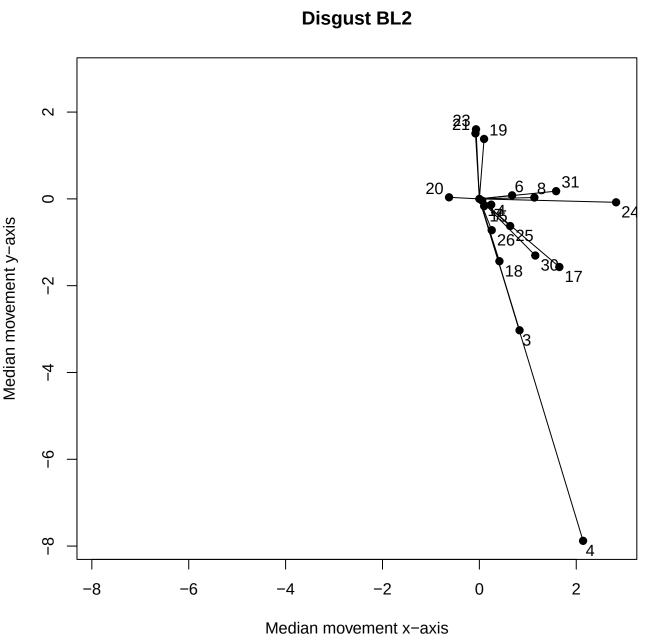
Disgust BL2
Median movement y−axis
Median movement x−axis

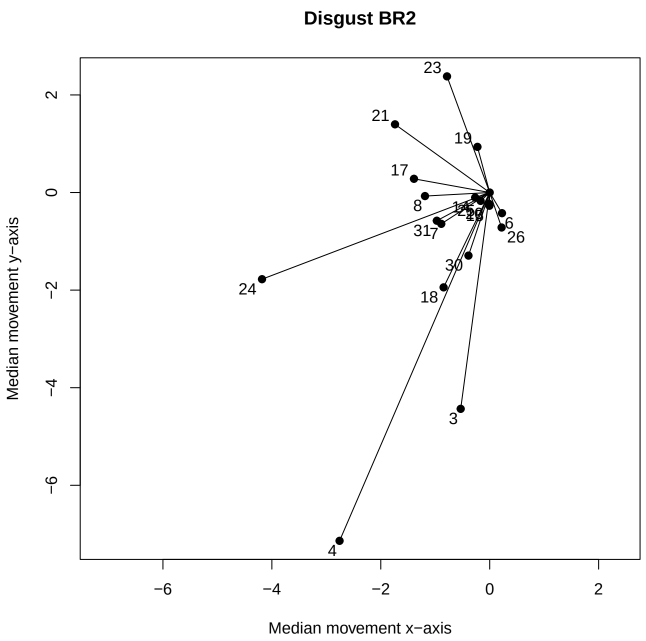
Disgust BR2
Median movement y−axis
Median movement x−axis

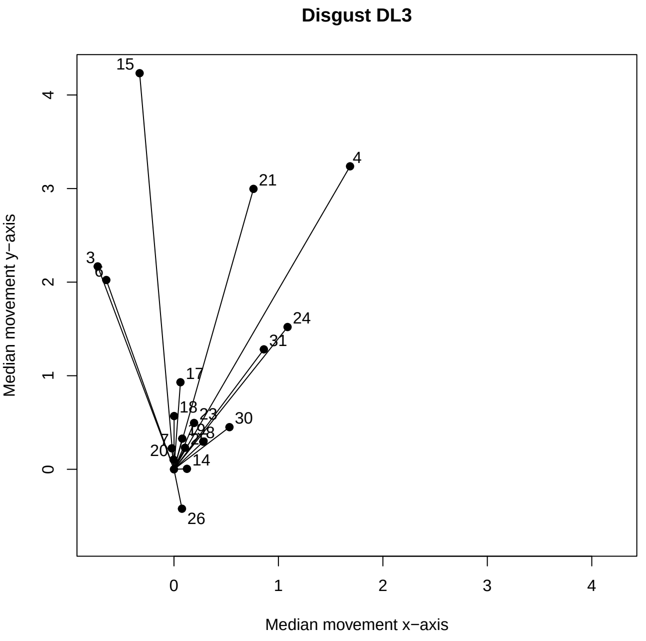
Disgust DL3
Median movement y−axis
Median movement x−axis

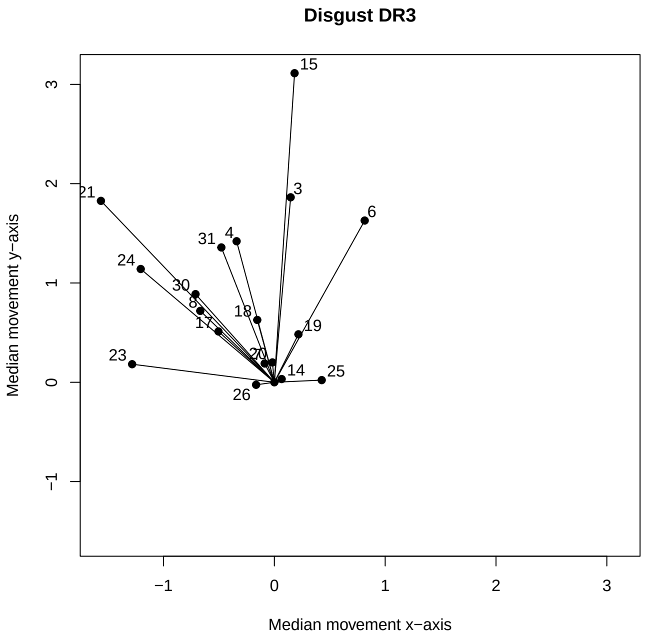
Disgust DR3
Median movement y−axis
Median movement x−axis

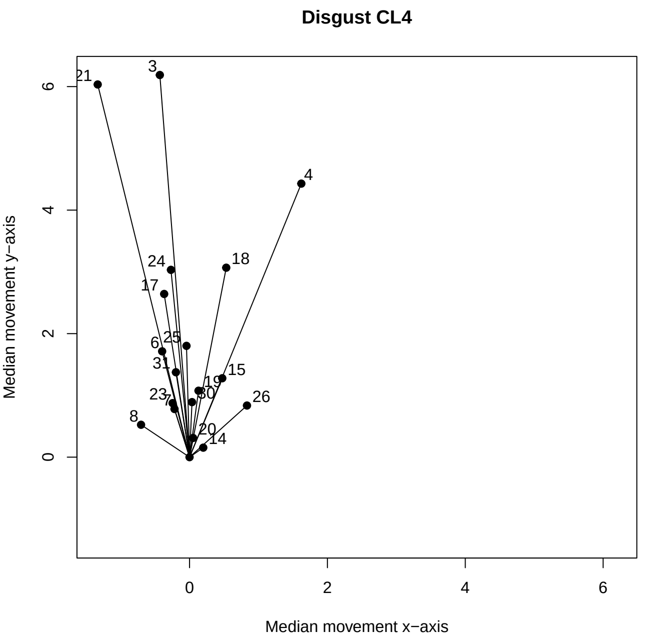
Disgust CL4
Median movement y−axis
Median movement x−axis

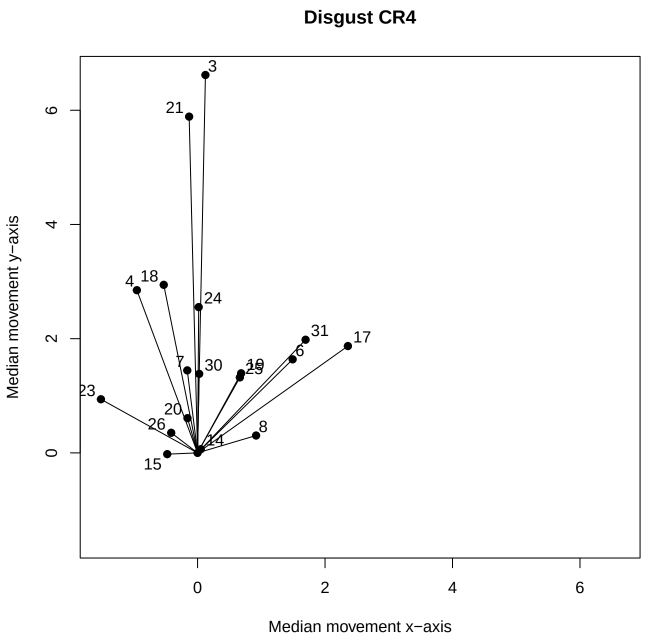
Disgust CR4
Median movement y−axis
Median movement x−axis

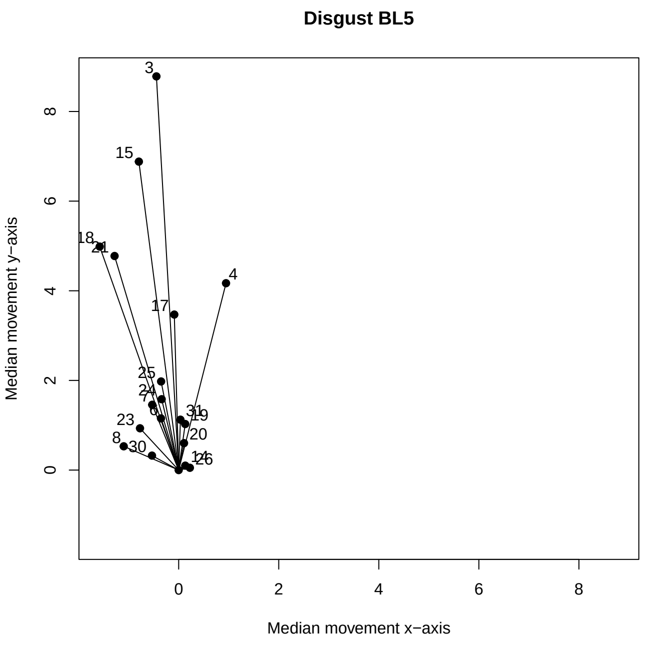
Disgust BL5
Median movement y−axis
Median movement x−axis

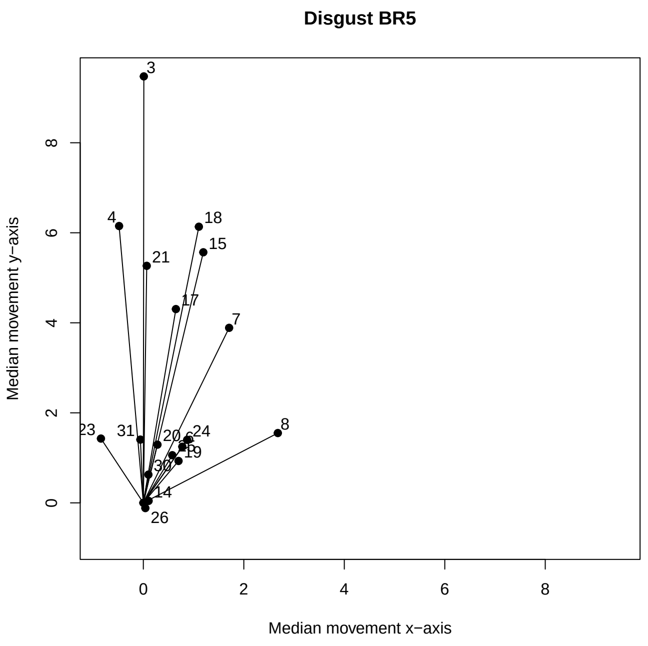
Disgust BR5
Median movement y−axis
Median movement x−axis

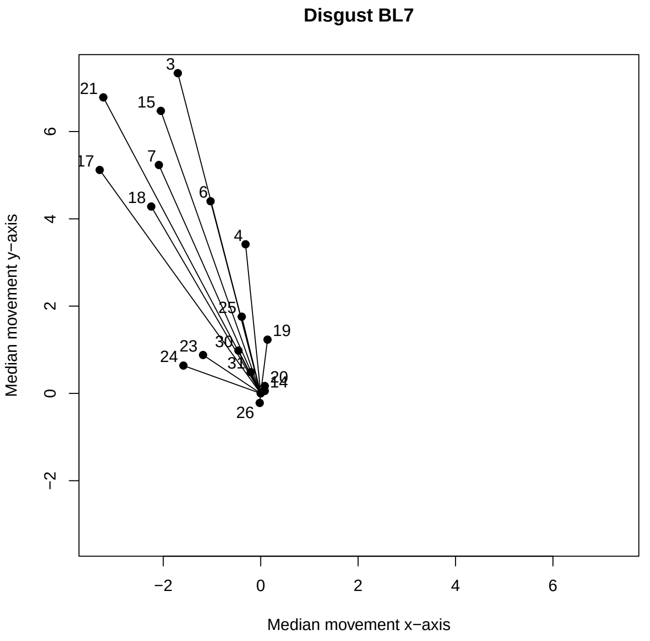
Disgust BL7
Median movement y−axis
Median movement x−axis

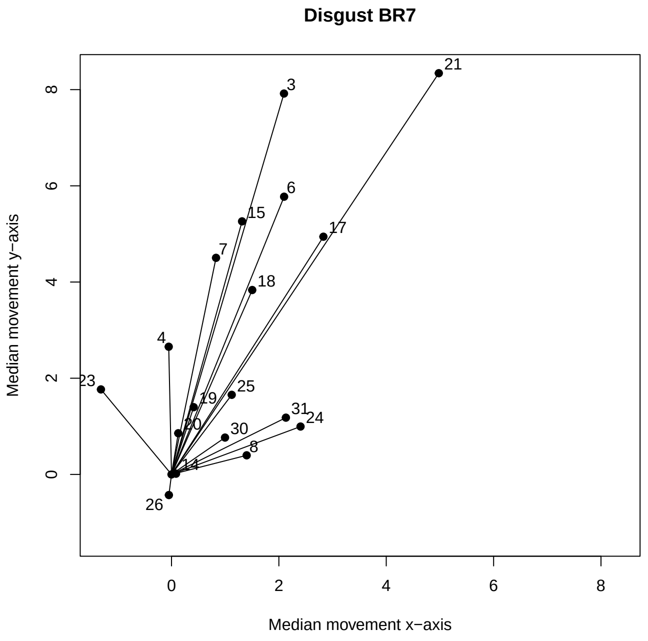
Disgust BR7
Median movement y−axis
Median movement x−axis

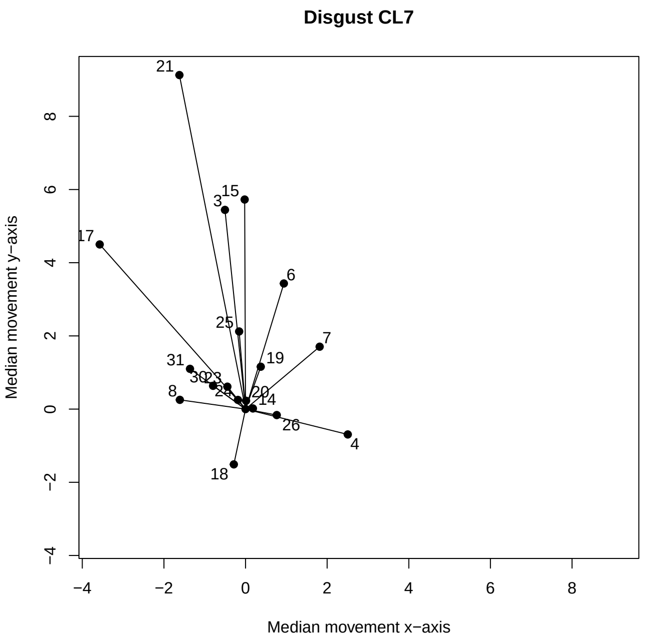
Disgust CL7
Median movement y−axis
Median movement x−axis

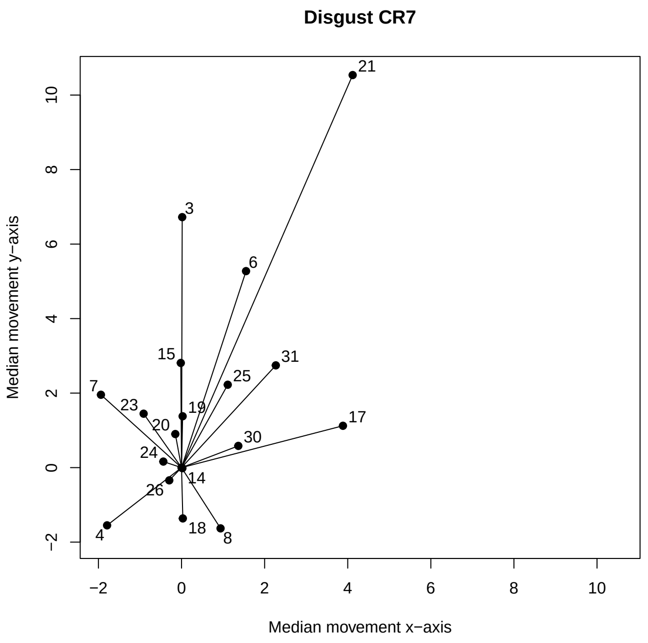
Disgust CR7
Median movement y−axis
Median movement x−axis

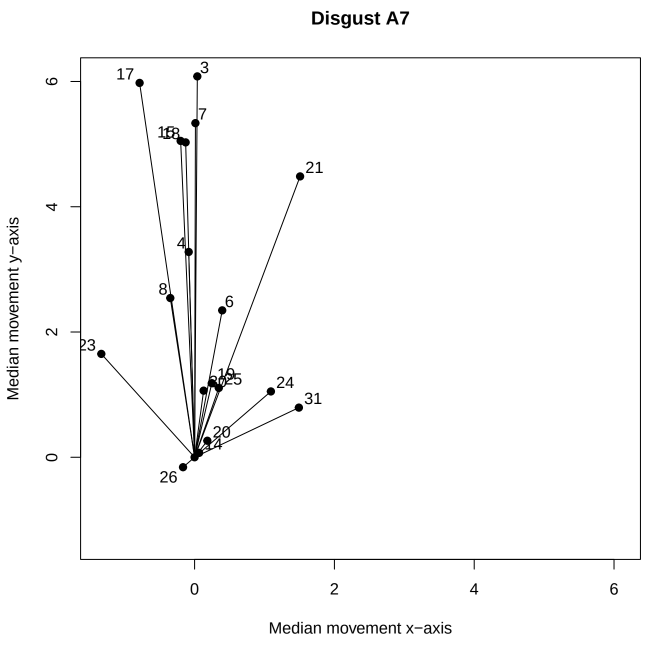

Disgust A7
Median movement y−axis
Median movement x−axis
0
2
4
6
17
3
7
18
4
21
8
6
23
10
24
31
20
14
26

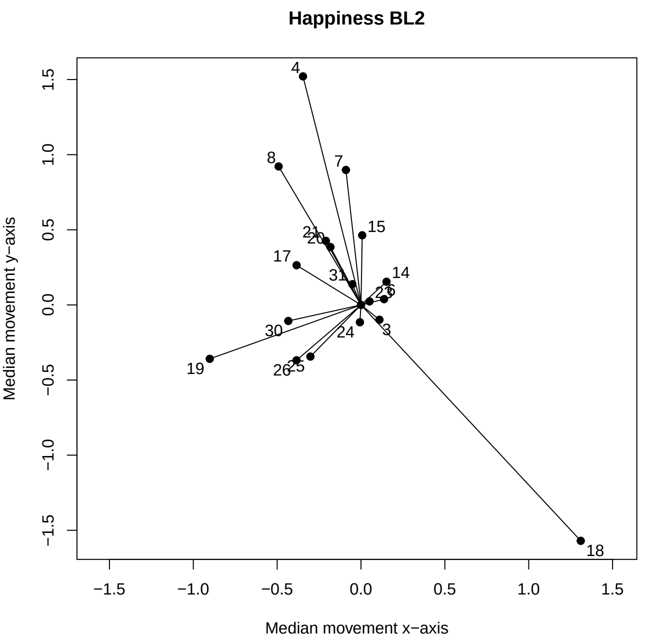
Happiness BL2
Median movement y−axis
Median movement x−axis

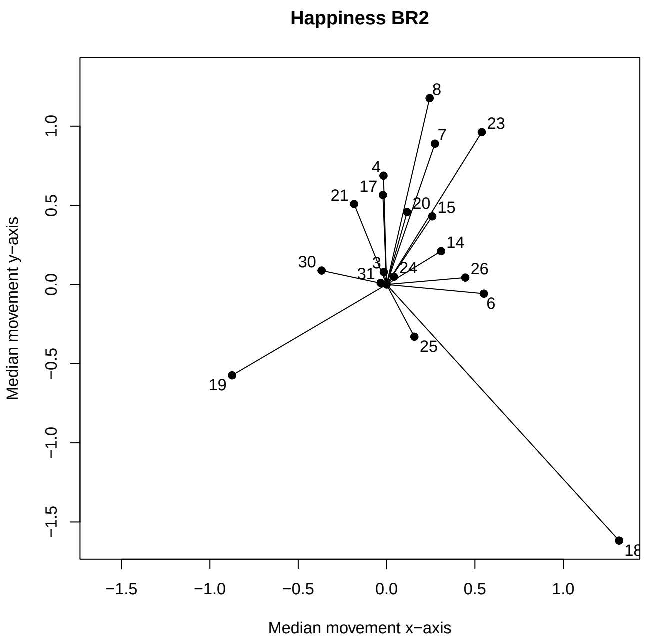
Happiness BR2
Median movement y−axis
Median movement x−axis

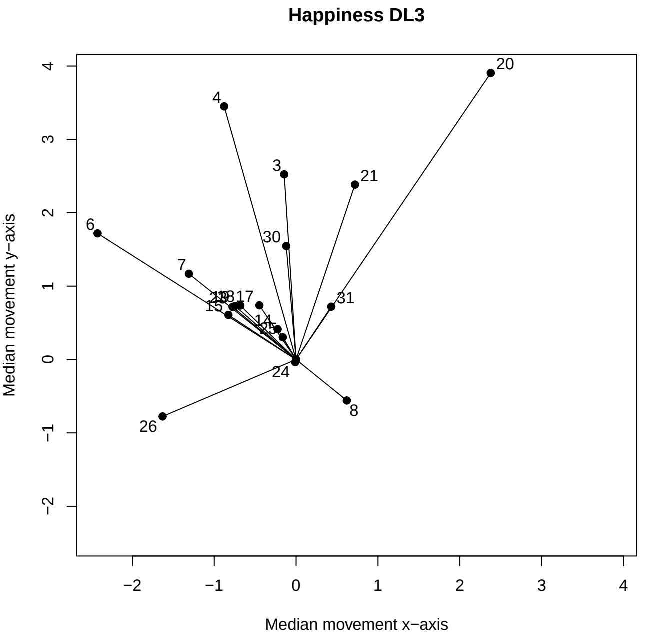
Happiness DL3
Median movement y−axis
Median movement x−axis

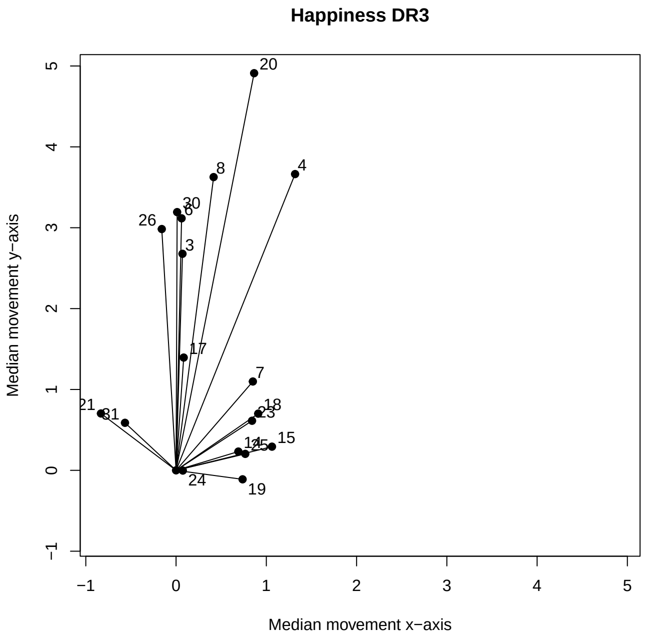
Happiness DR3
Median movement y−axis
Median movement x−axis

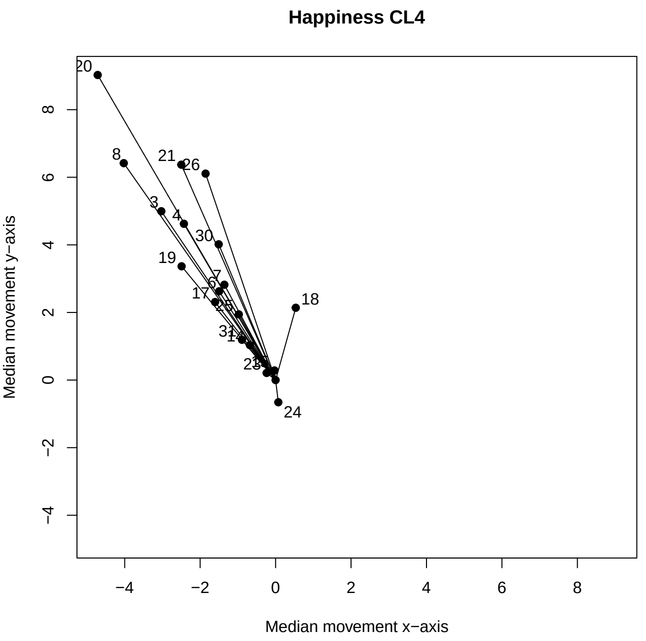
Happiness CL4
Median movement y−axis
Median movement x−axis

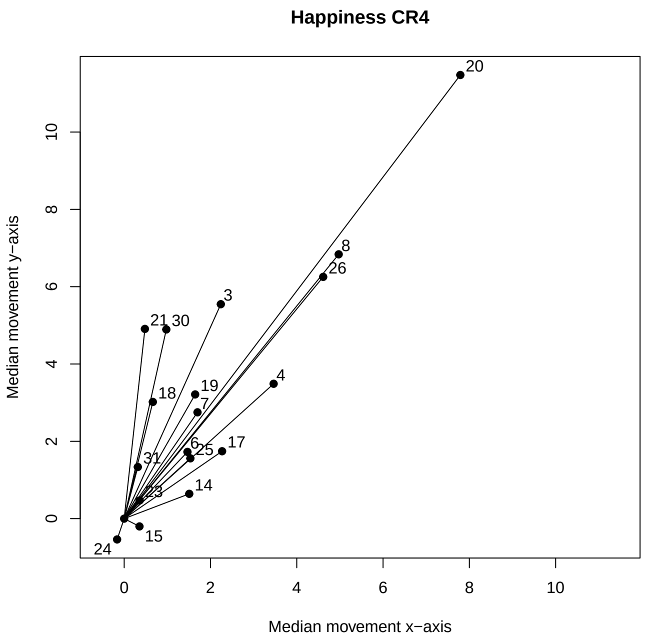
Happiness CR4
Median movement y−axis
Median movement x−axis

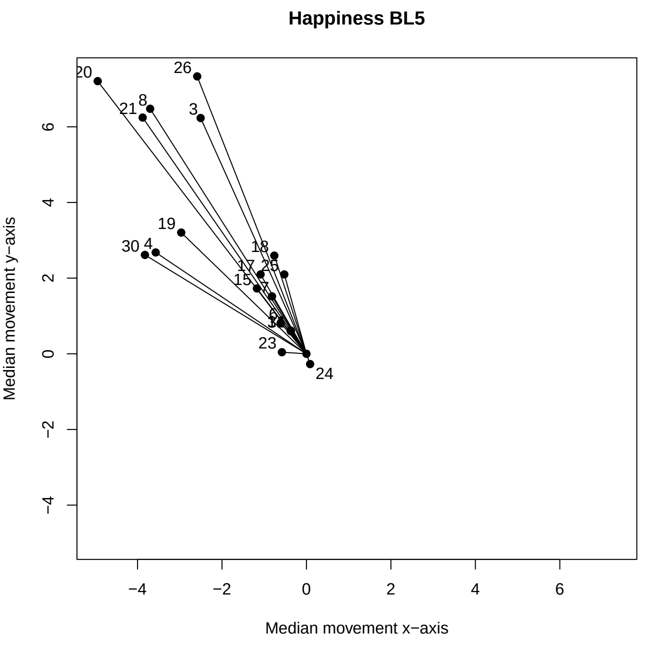
Happiness BL5
Median movement y−axis
Median movement x−axis

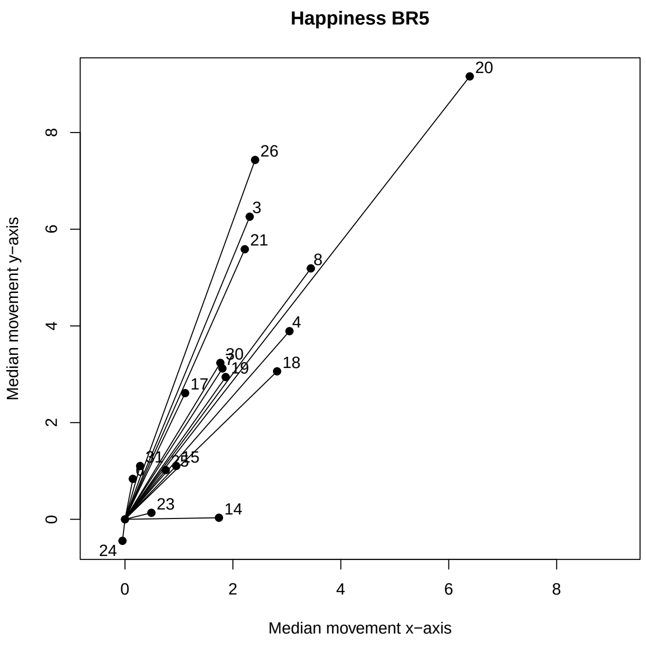
Happiness BR5
Median movement y−axis
Median movement x−axis

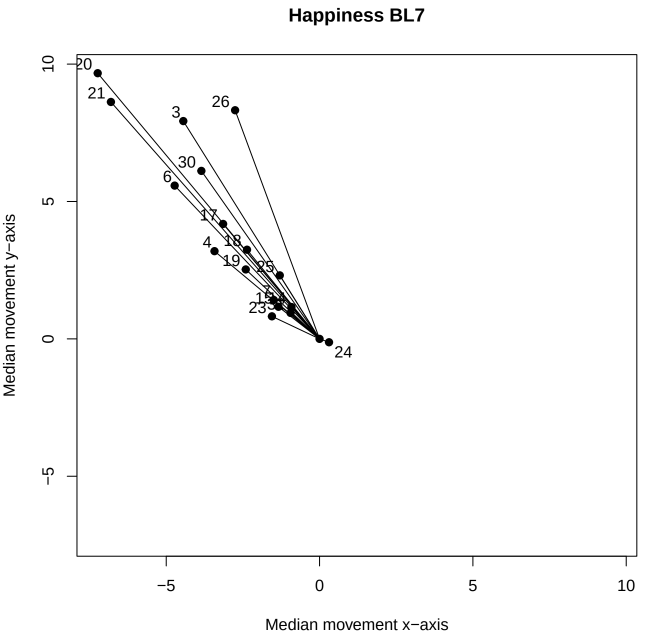
Happiness BL7
Median movement y−axis
Median movement x−axis

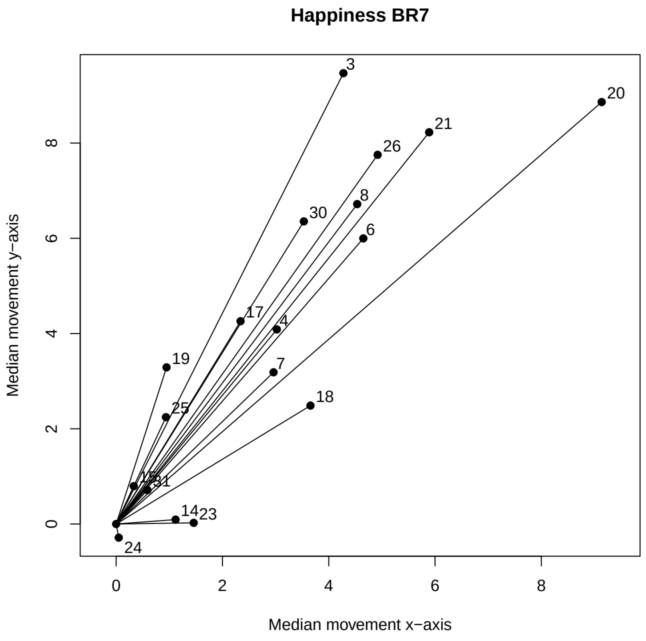
Happiness BR7
Median movement y−axis
Median movement x−axis

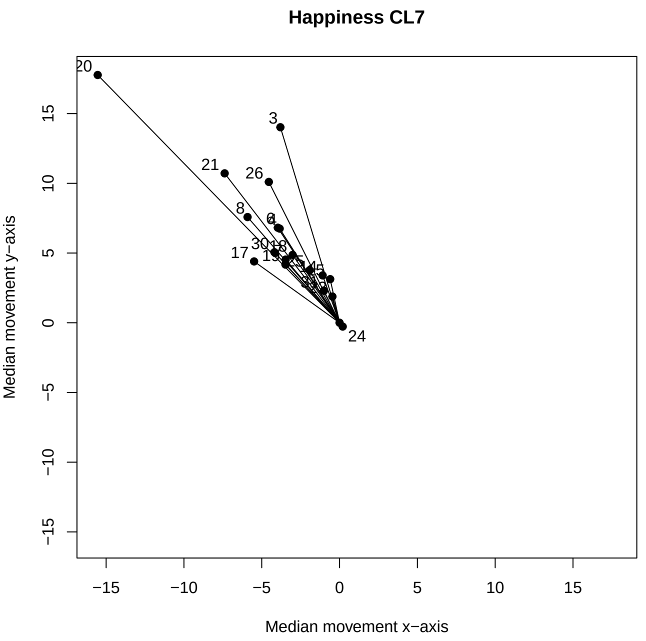
Happiness CL7
Median movement y−axis
Median movement x−axis

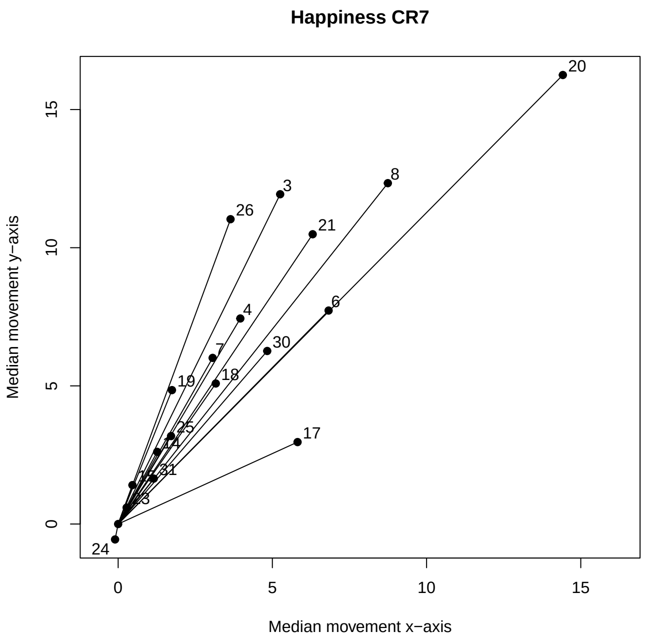
Happiness CR7
Median movement y−axis
Median movement x−axis

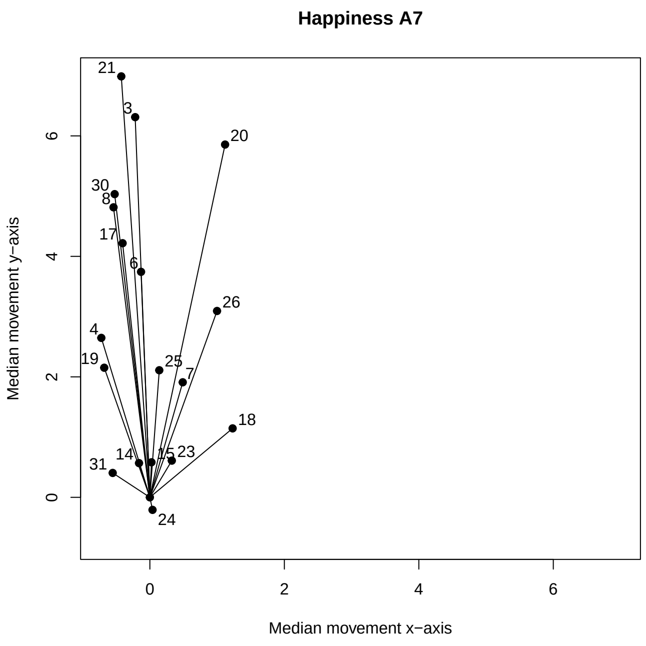

Happiness A7
Median movement y−axis
Median movement x−axis
0
2
4
6
21
3
20
30
8
17
6
26
4
19
25
7
18
14
15
23
31
24

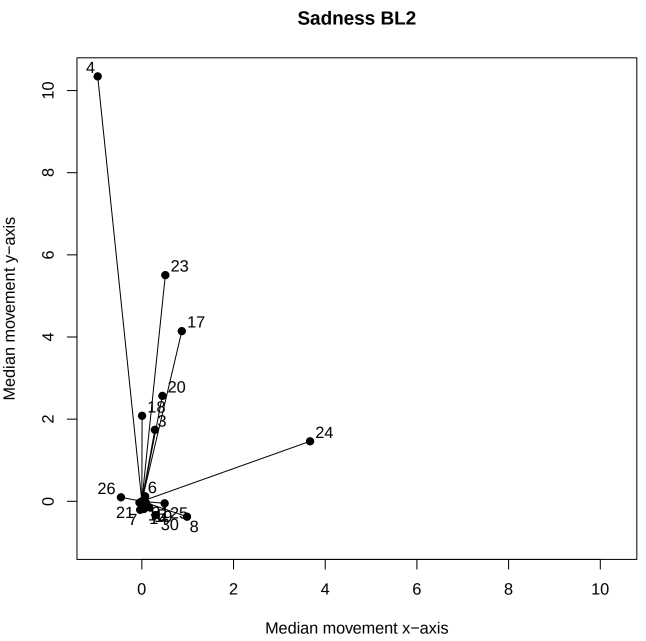
Sadness BL2
Median movement y−axis
Median movement x−axis
4
23
17
20
18
3
24
26
6
21
7
25
30
8

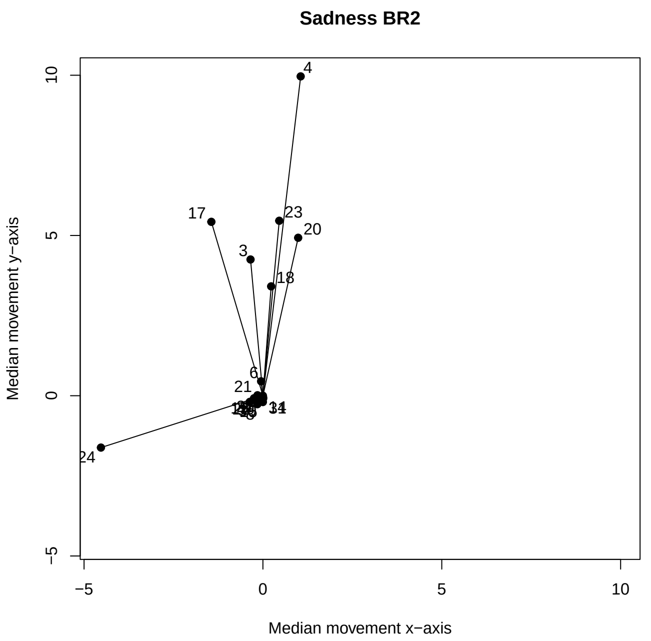
Sadness BR2
Median movement y−axis
Median movement x−axis
4
17
23
20
3
18
6
21
24

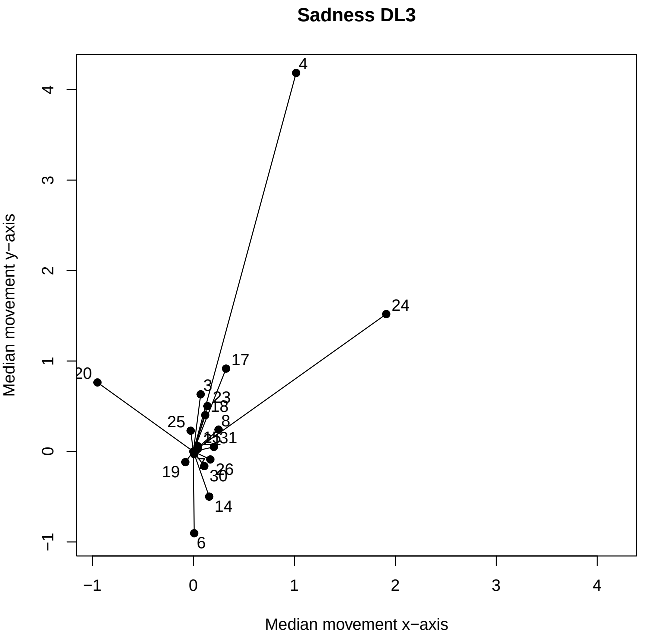
Sadness DL3
Median movement y−axis
Median movement x−axis
4
24
17
20
3
23
18
25
8
31
19
26
30
14
6

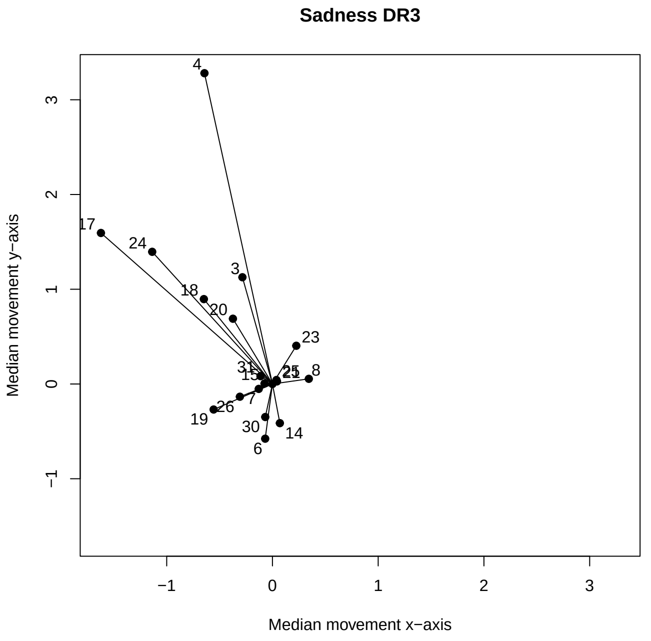
Sadness DR3
Median movement y−axis
Median movement x−axis
4
17
24
3
18
20
23
31
15
8
26
7
19
30
14
6

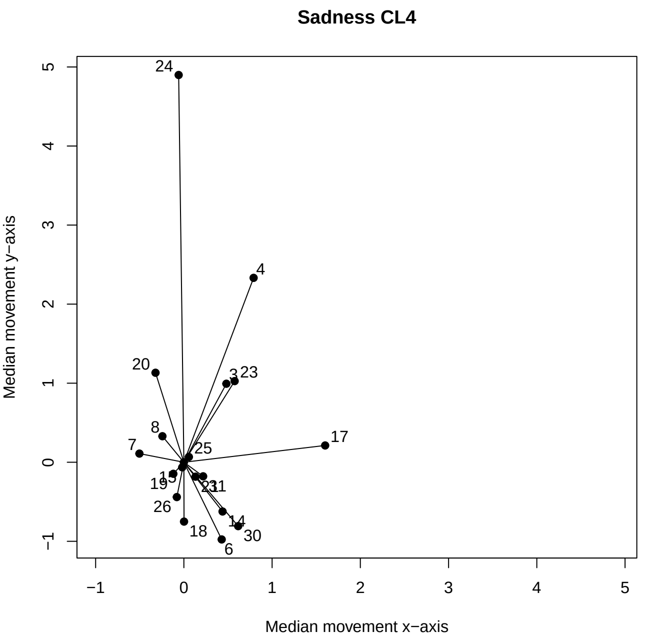
Sadness CL4
Median movement y−axis
Median movement x−axis
24
4
20
3
23
8
7
25
17
19
26
18
14
30
6

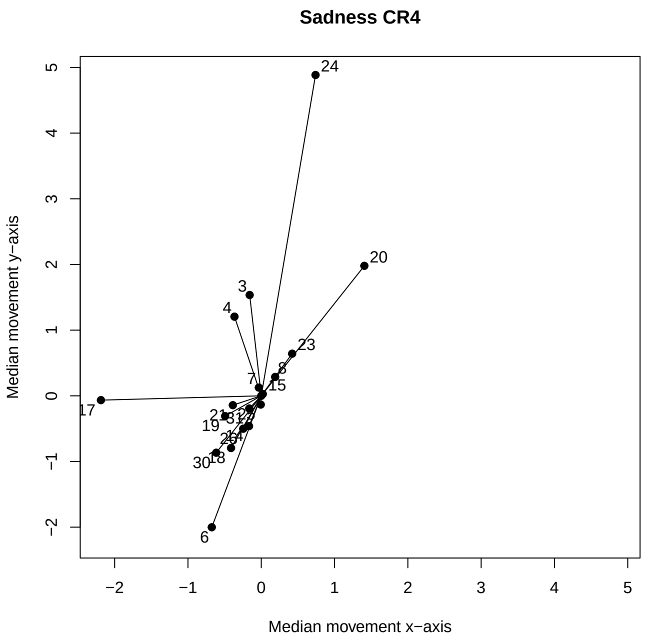
Sadness CR4
Median movement y−axis
Median movement x−axis
24
20
3
4
23
8
7
15
17
19
30
18
6

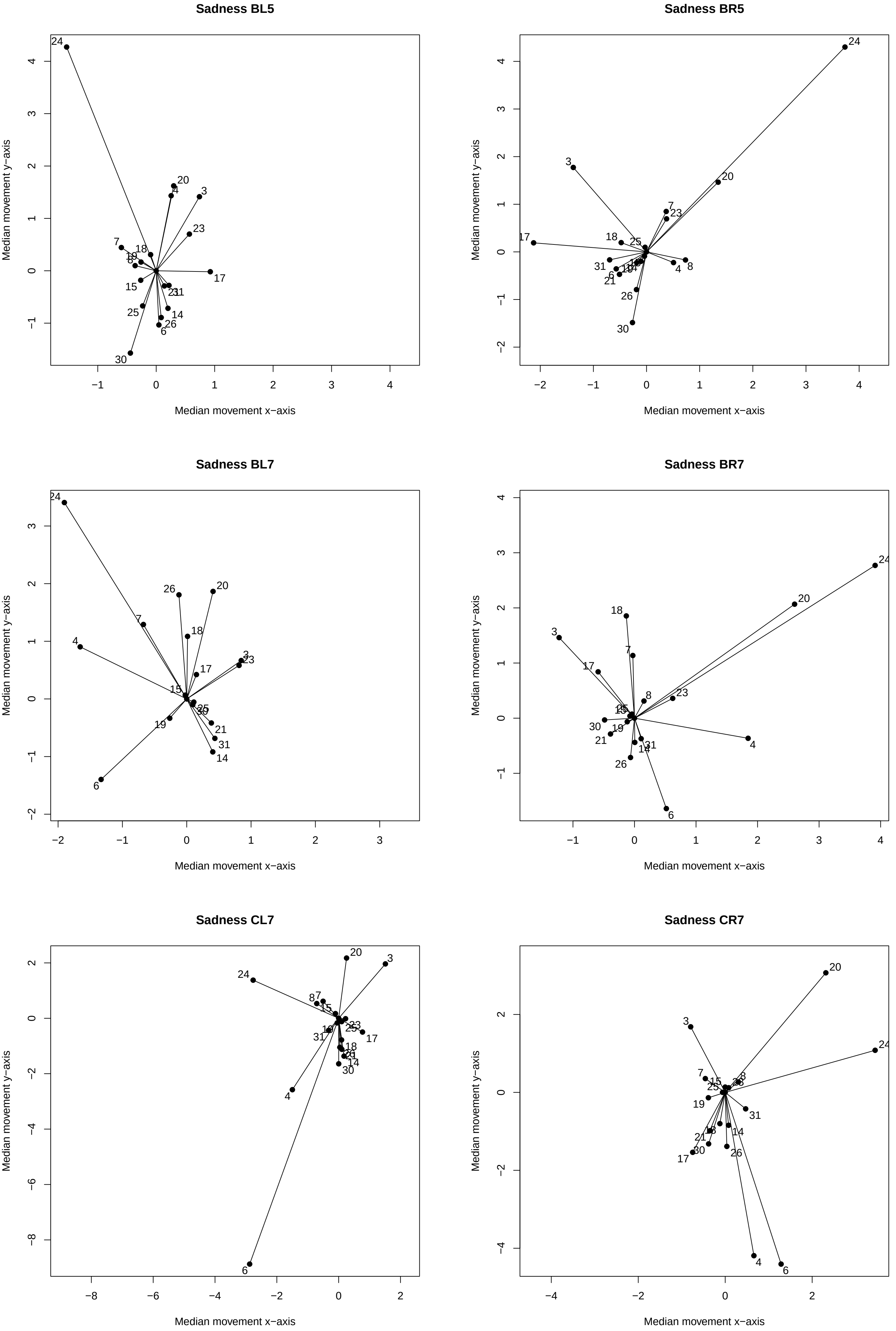
Sadness BL5
Sadness BR5
Sadness BL7
Sadness BR7
Sadness CL7
Sadness CR7
Median movement x−axis
Median movement y−axis

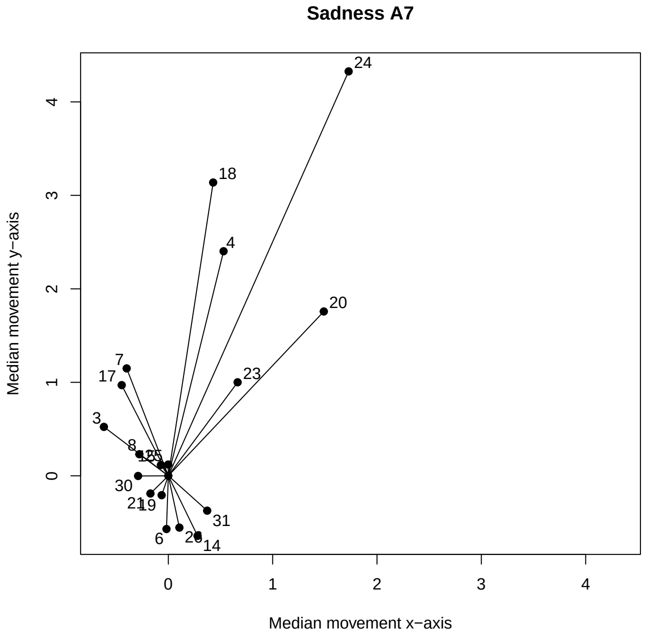

Sadness A7
Median movement y−axis
Median movement x−axis
0
1
2
3
4
24
18
4
20
23
7
17
3
8
30
21
19
31
6
26
14
