# Supplementary material for: blenderFaceUV: Toward Marker‐Based Precision and Flexibility in Facial Tracking With “Invisible” Markers
Source: Psychophysiology. 2026 Sep 21;63(9):e70394. doi: 10.1111/psyp.70394 (PMC13594373; doi:10.1111/psyp.70394)
Supplement: Supplementary file 3 [file PSYP-63-e70394-s001.zip › Study 2/Plots/01_Descriptive_Plots/03_XYmmpf.pdf]

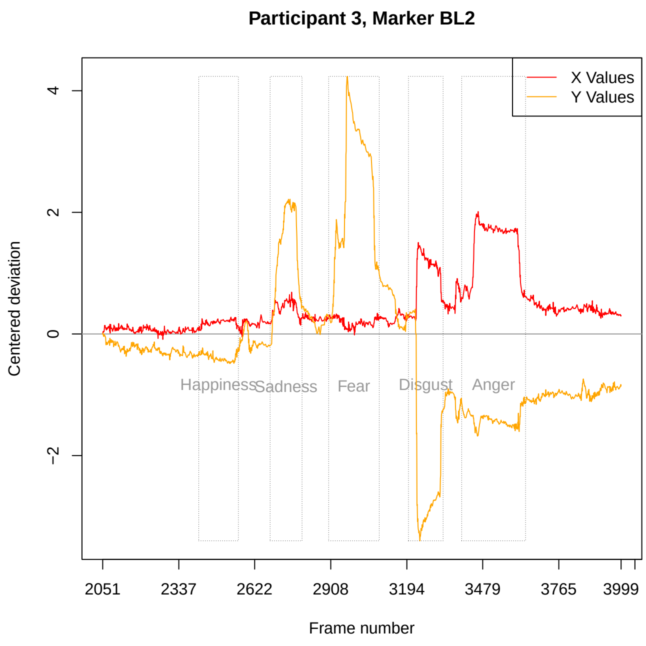

Participant 3, Marker BL2
X Values
Y Values
Happiness
Sadness
Fear
Disgust
Anger
Centered deviation
Frame number
2051 2337 2622 2908 3194 3479 3765 3999

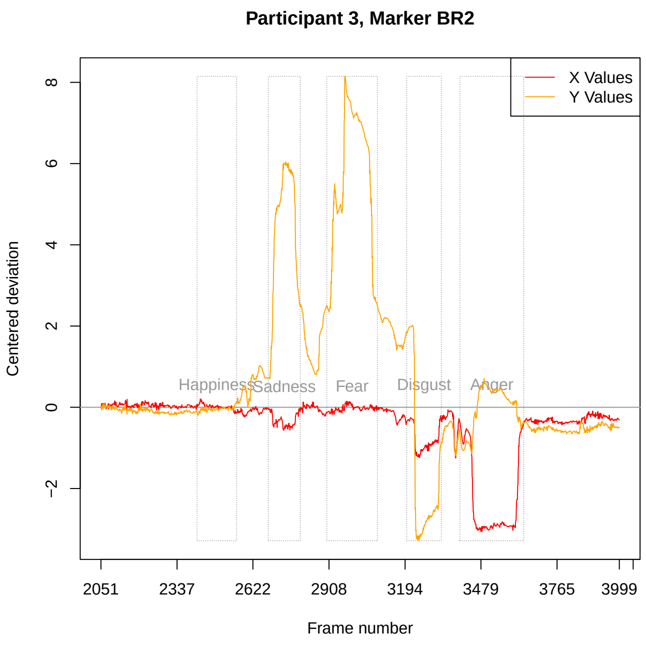

Participant 3, Marker BR2
X Values
Y Values
Happiness
Sadness
Fear
Disgust
Anger
Centered deviation
Frame number
2051 2337 2622 2908 3194 3479 3765 3999

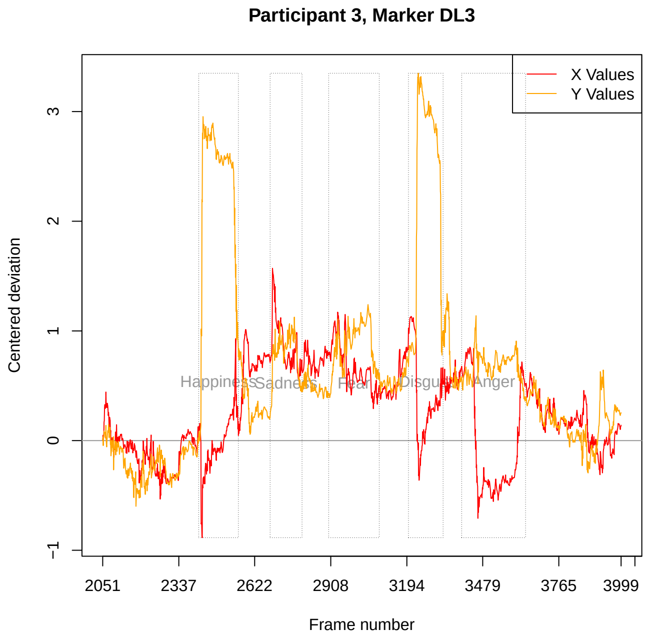

Participant 3, Marker DL3
X Values
Y Values
Happiness
Sadness
Fear
Disgust
Anger
Centered deviation
Frame number
2051 2337 2622 2908 3194 3479 3765 3999

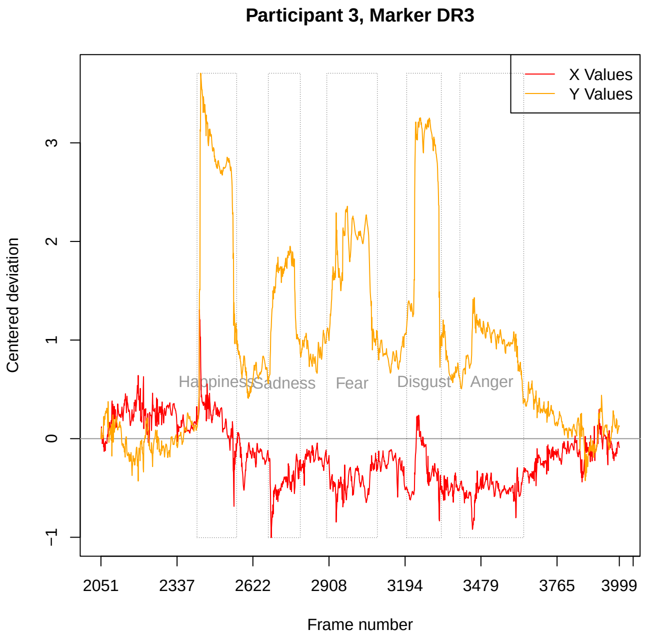

Participant 3, Marker DR3
X Values
Y Values
Happiness
Sadness
Fear
Disgust
Anger
Centered deviation
Frame number
2051 2337 2622 2908 3194 3479 3765 3999

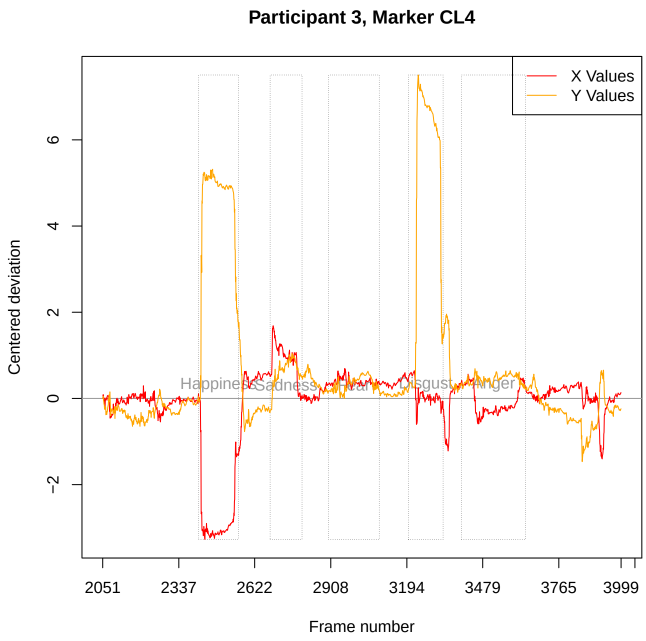

Participant 3, Marker CL4
X Values
Y Values
Happiness
Sadness
Fear
Disgust
Anger
Centered deviation
Frame number
2051 2337 2622 2908 3194 3479 3765 3999

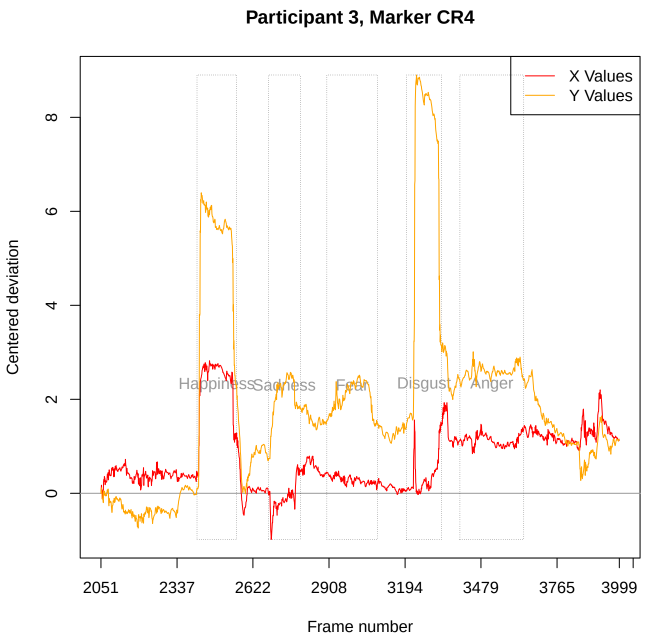

Participant 3, Marker CR4
X Values
Y Values
Happiness
Sadness
Fear
Disgust
Anger
Centered deviation
Frame number
2051 2337 2622 2908 3194 3479 3765 3999

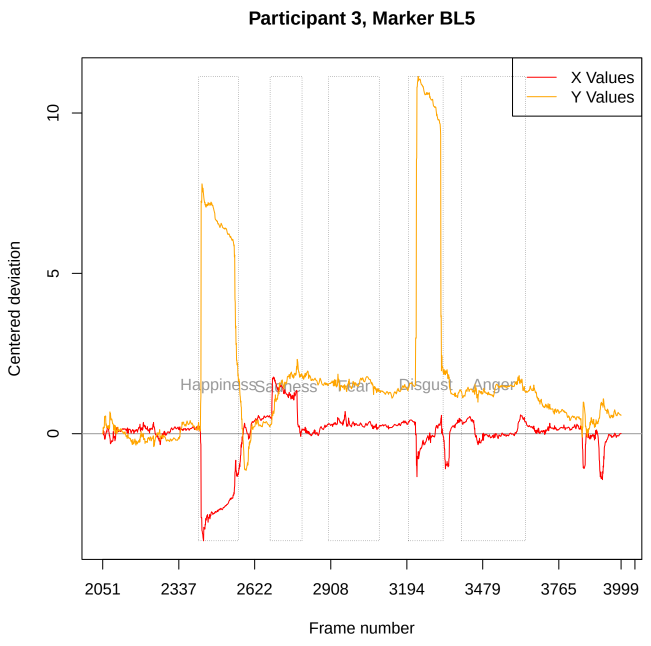
Participant 3, Marker BL5
X Values
Y Values
Happiness
Sadness
Fear
Disgust
Anger
0
5
10
2051
2337
2622
2908
3194
3479
3765
3999
Frame number
Centered deviation

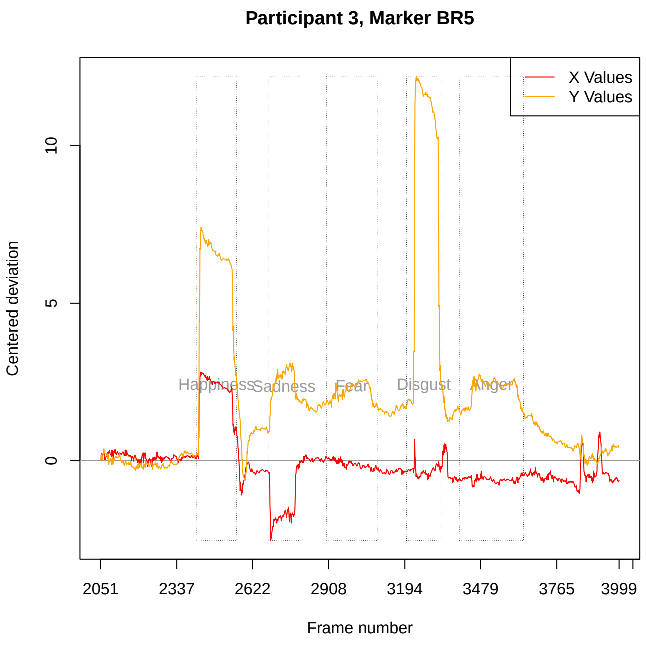
Participant 3, Marker BR5
X Values
Y Values
Happiness
Sadness
Fear
Disgust
Anger
0
5
10
2051
2337
2622
2908
3194
3479
3765
3999
Frame number
Centered deviation

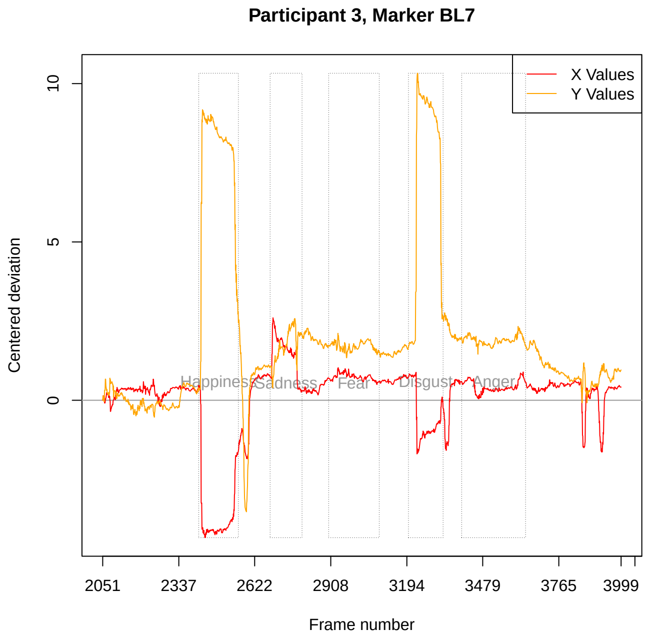
Participant 3, Marker BL7
X Values
Y Values
Happiness
Sadness
Fear
Disgust
Anger
0
5
10
2051
2337
2622
2908
3194
3479
3765
3999
Frame number
Centered deviation

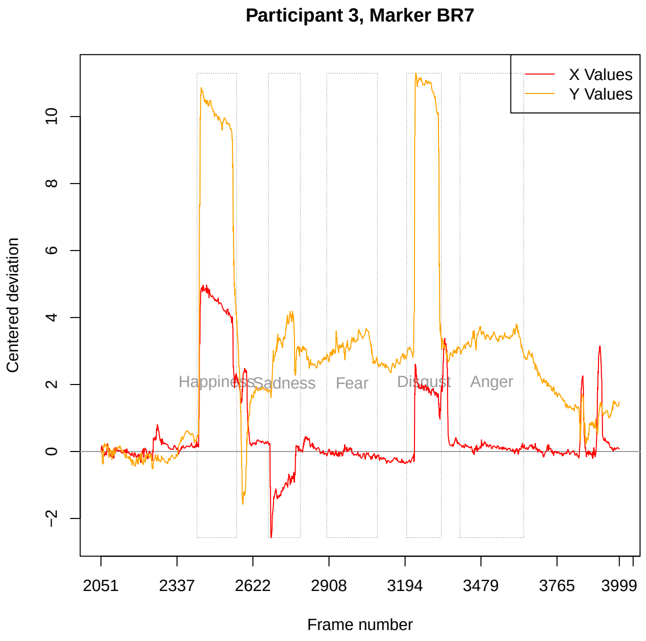
Participant 3, Marker BR7
X Values
Y Values
Happiness
Sadness
Fear
Disgust
Anger
−2
0
2
4
6
8
10
2051
2337
2622
2908
3194
3479
3765
3999
Frame number
Centered deviation

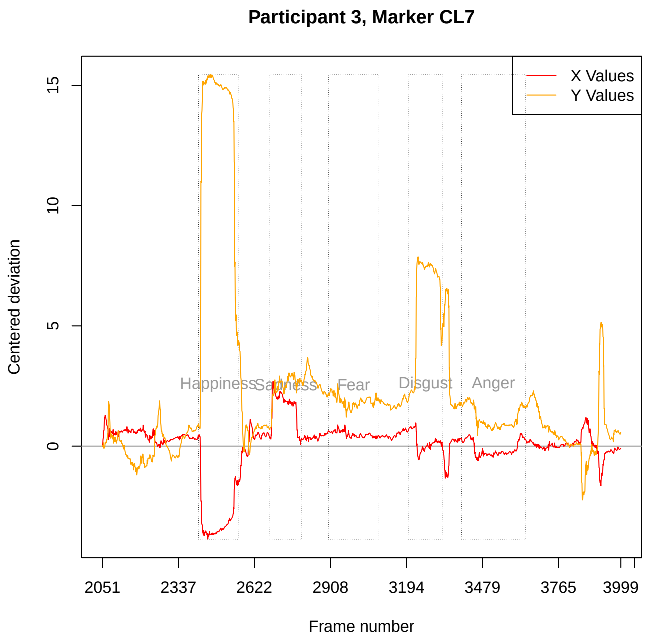
Participant 3, Marker CL7
X Values
Y Values
Happiness
Sadness
Fear
Disgust
Anger
0
5
10
15
2051
2337
2622
2908
3194
3479
3765
3999
Frame number
Centered deviation

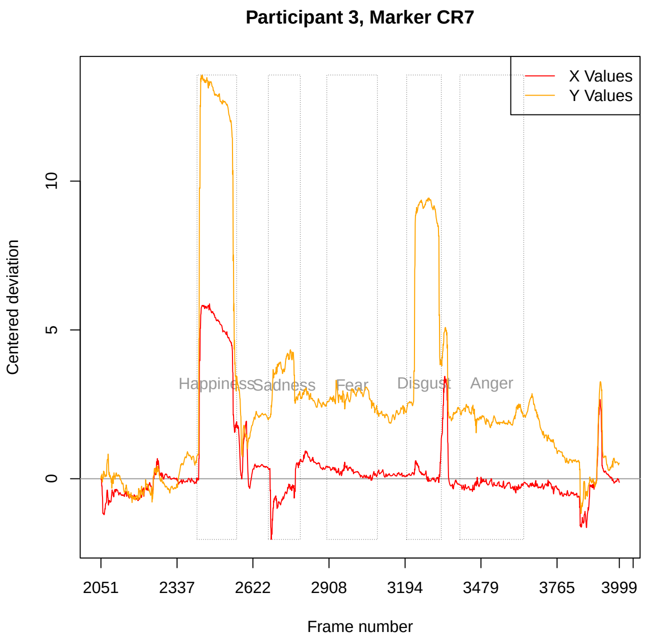
Participant 3, Marker CR7
X Values
Y Values
Happiness
Sadness
Fear
Disgust
Anger
0
5
10
2051
2337
2622
2908
3194
3479
3765
3999
Frame number
Centered deviation

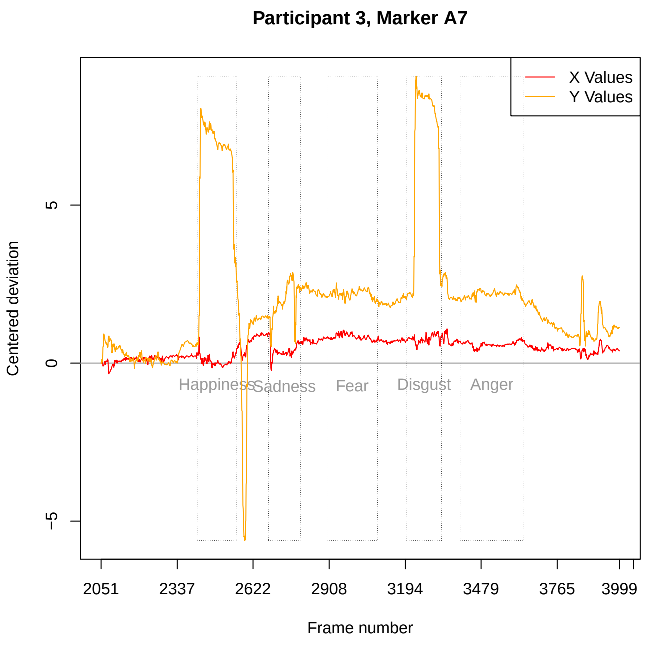

Participant 3, Marker A7
X Values
Y Values
Centered deviation
5
0
−5
Happiness
Sadness
Fear
Disgust
Anger
2051
2337
2622
2908
3194
3479
3765
3999
Frame number

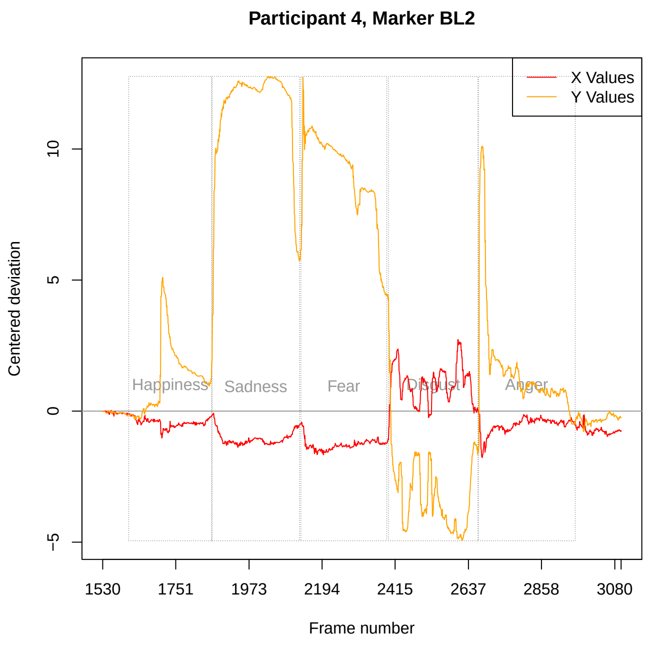
Participant 4, Marker BL2
X Values
Y Values
Centered deviation
Frame number
Happiness
Sadness
Fear
Disgust
Anger
-5
0
5
10
1530
1751
1973
2194
2415
2637
2858
3080

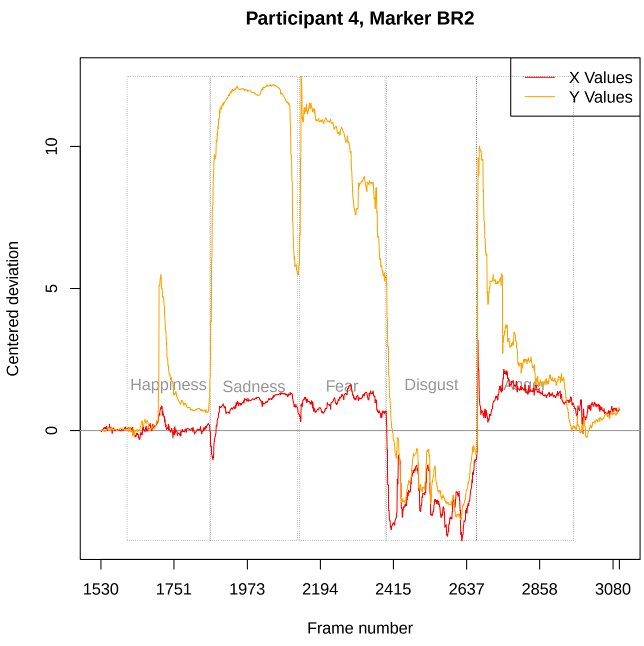
Participant 4, Marker BR2
X Values
Y Values
Centered deviation
Frame number
Happiness
Sadness
Fear
Disgust
Anger
0
5
10
1530
1751
1973
2194
2415
2637
2858
3080

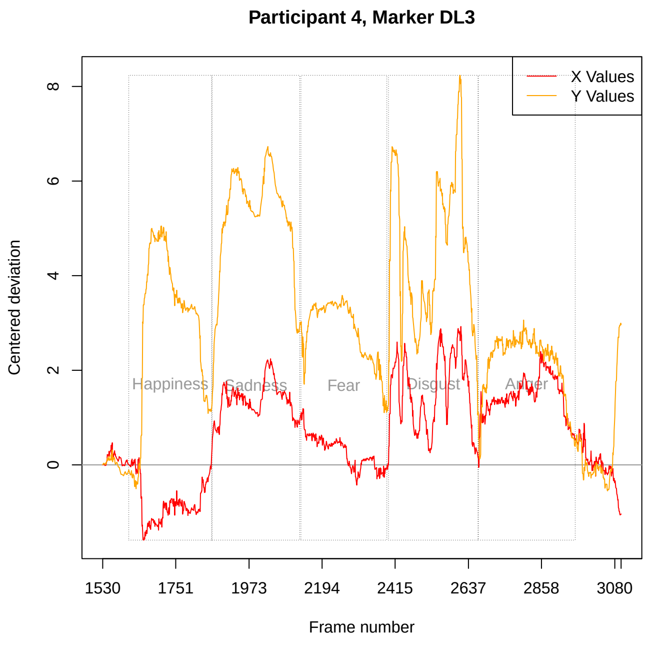
Participant 4, Marker DL3
X Values
Y Values
Centered deviation
Frame number
Happiness
Sadness
Fear
Disgust
Anger
0
2
4
6
8
1530
1751
1973
2194
2415
2637
2858
3080

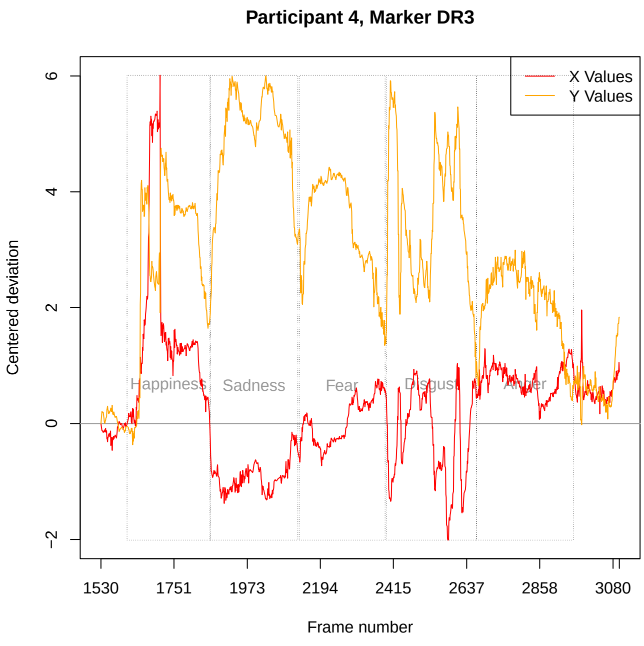
Participant 4, Marker DR3
X Values
Y Values
Centered deviation
Frame number
Happiness
Sadness
Fear
Disgust
Anger
-2
0
2
4
6
1530
1751
1973
2194
2415
2637
2858
3080

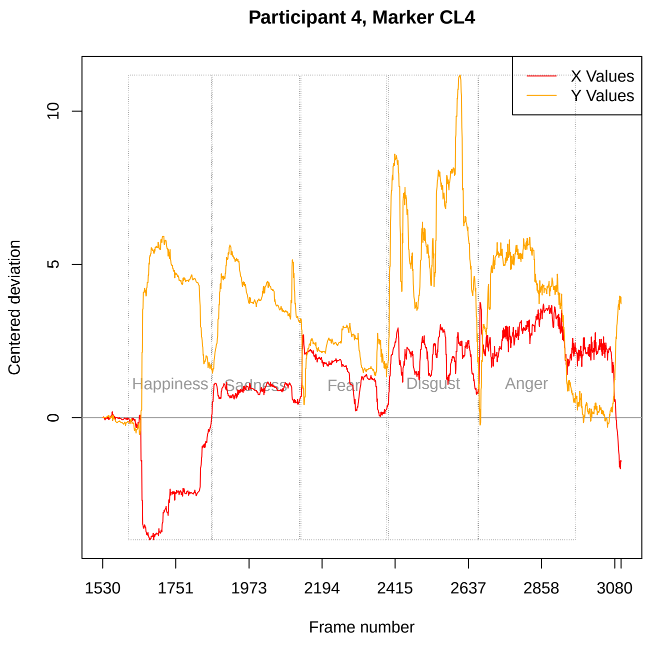
Participant 4, Marker CL4
X Values
Y Values
Centered deviation
Frame number
Happiness
Sadness
Fear
Disgust
Anger
0
5
10
1530
1751
1973
2194
2415
2637
2858
3080

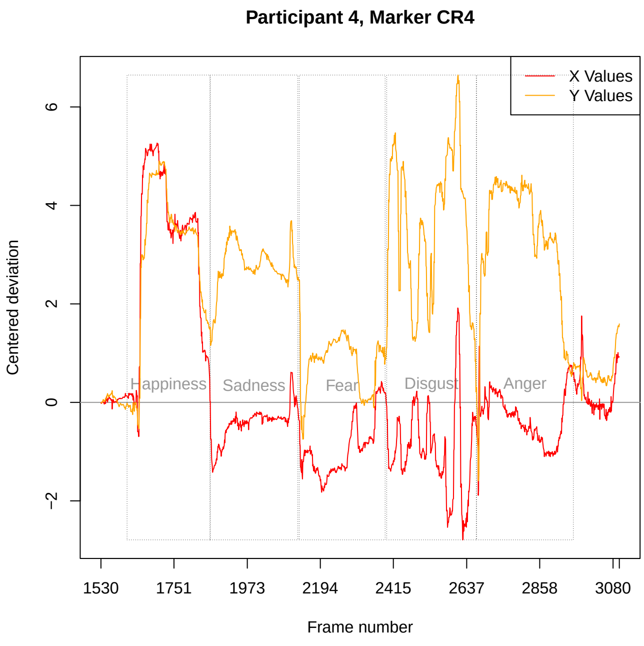
Participant 4, Marker CR4
X Values
Y Values
Centered deviation
Frame number
Happiness
Sadness
Fear
Disgust
Anger
-2
0
2
4
6
1530
1751
1973
2194
2415
2637
2858
3080

Participant 4, Marker BL5

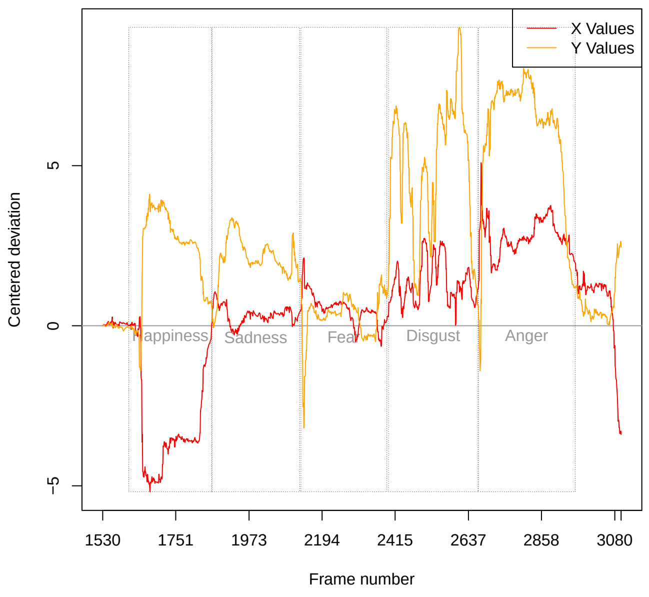

Participant 4, Marker BR5

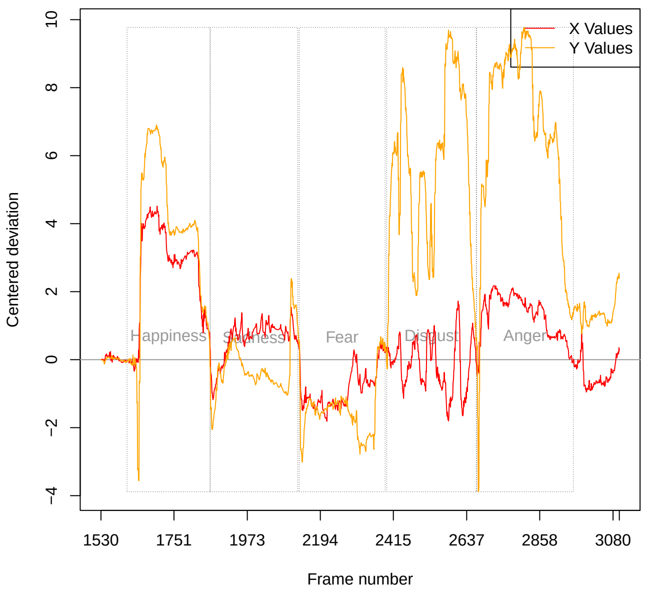

Participant 4, Marker BL7

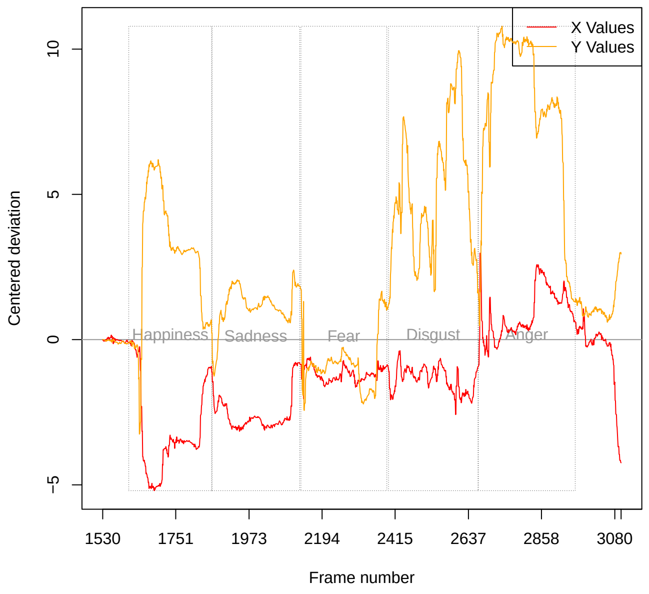

Participant 4, Marker BR7

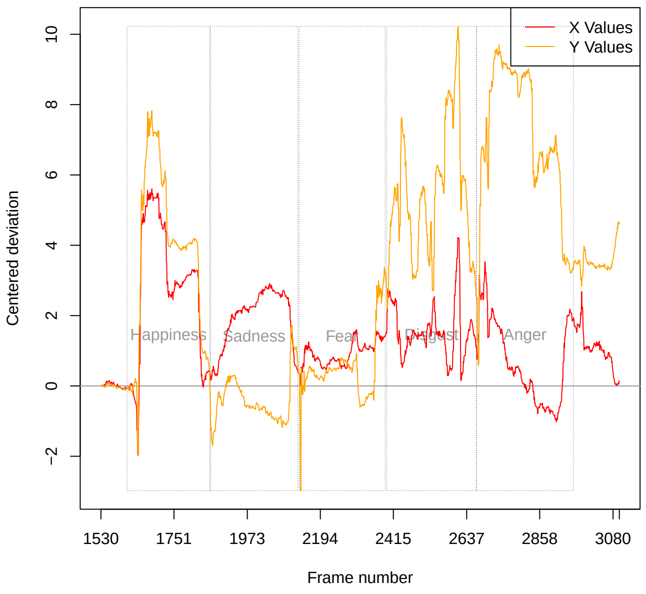

Participant 4, Marker CL7

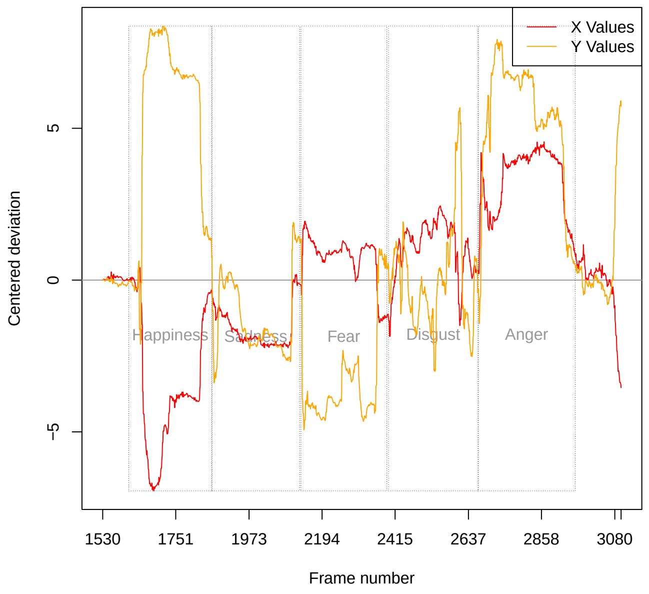

Participant 4, Marker CR7

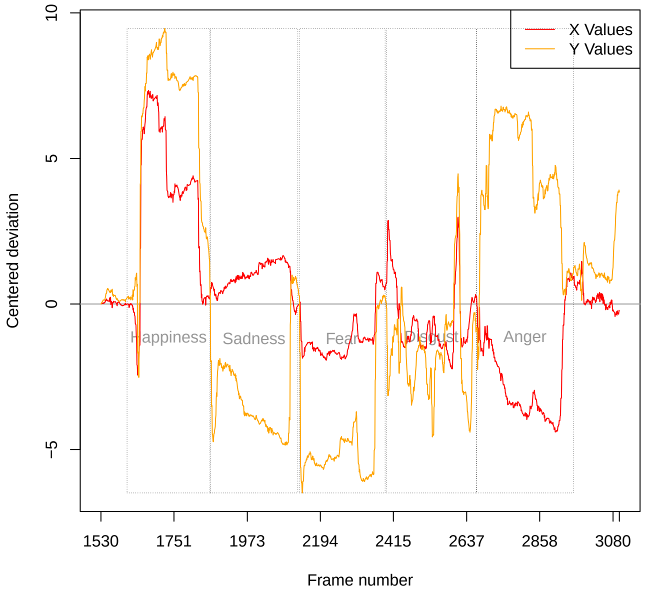

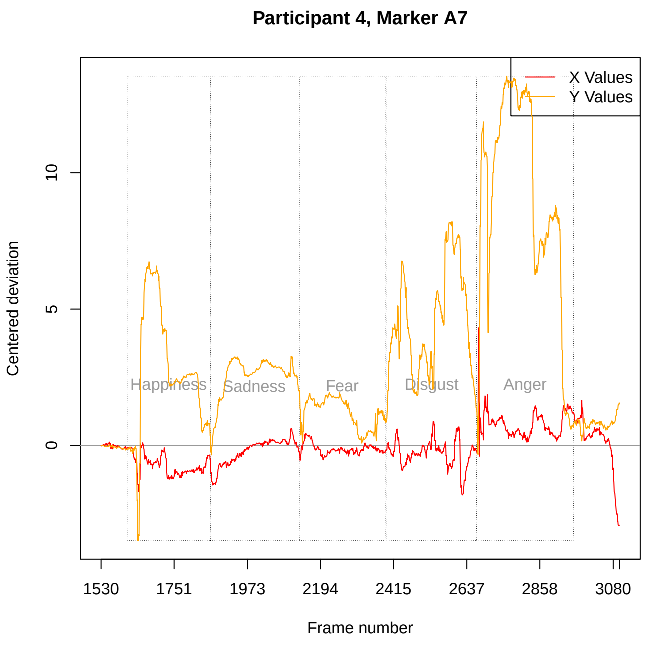

Participant 4, Marker A7
X Values
Y Values
Happiness
Sadness
Fear
Disgust
Anger
Centered deviation
0
5
10
1530
1751
1973
2194
2415
2637
2858
3080
Frame number

Participant 6, Marker BL2

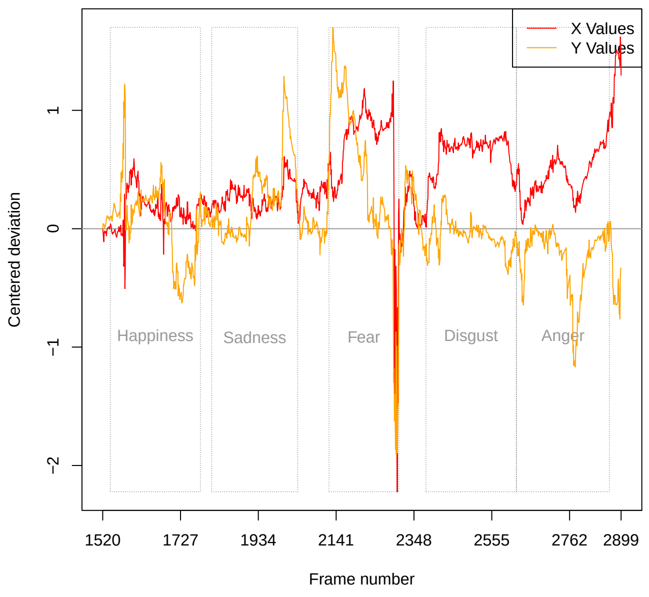

Participant 6, Marker BR2

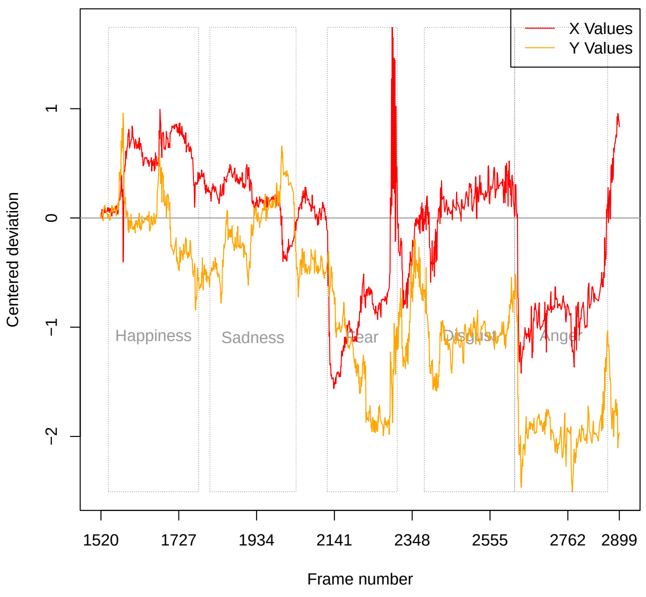

Participant 6, Marker DL3

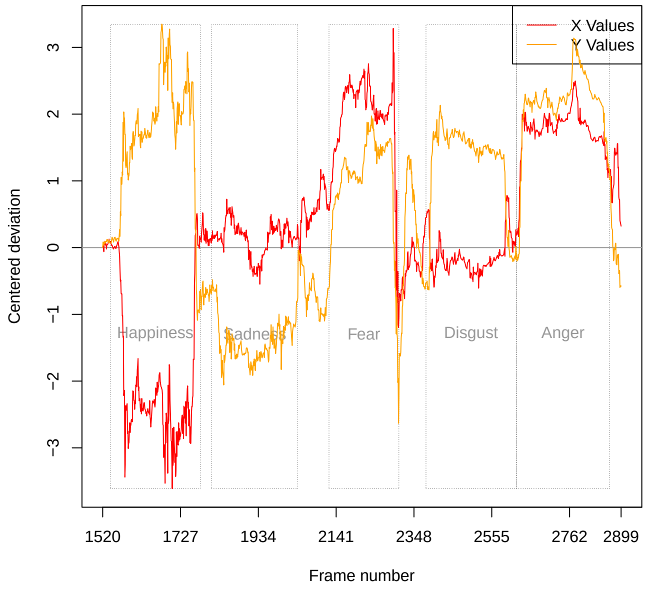

Participant 6, Marker DR3

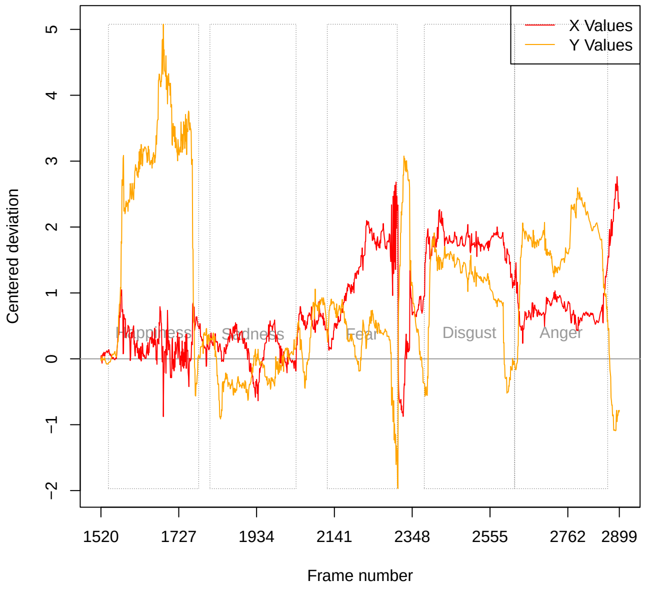

Participant 6, Marker CL4

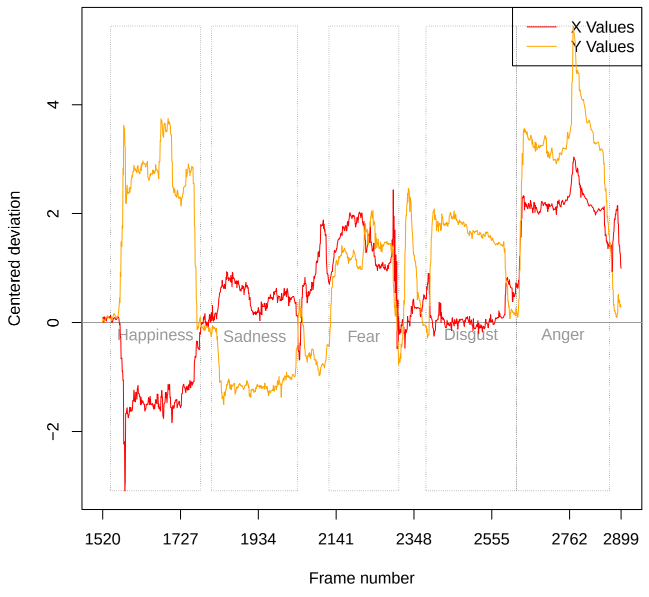

Participant 6, Marker CR4

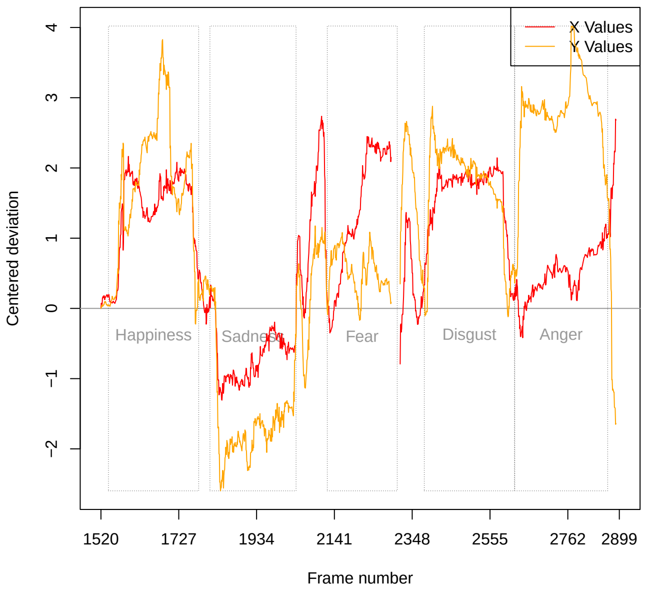

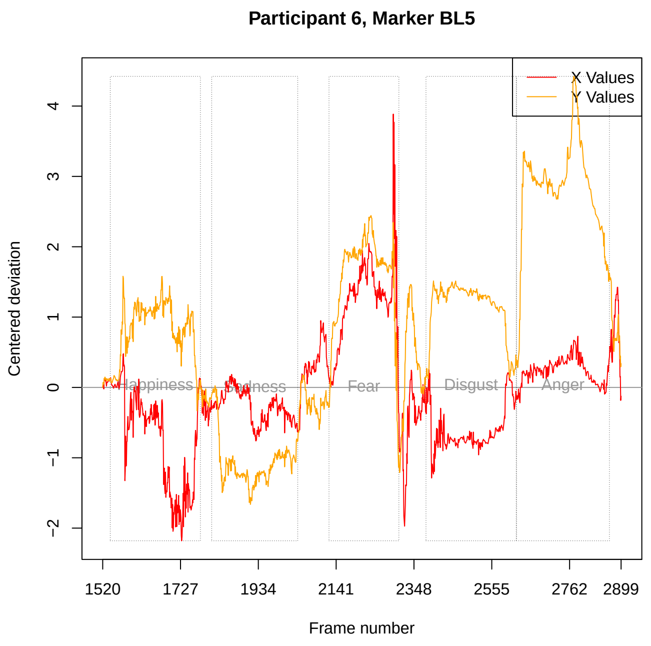
Participant 6, Marker BL5
X Values
Y Values
Happiness
Sadness
Fear
Disgust
Anger
Centered deviation
−2
−1
0
1
2
3
4
1520
1727
1934
2141
2348
2555
2762
2899
Frame number

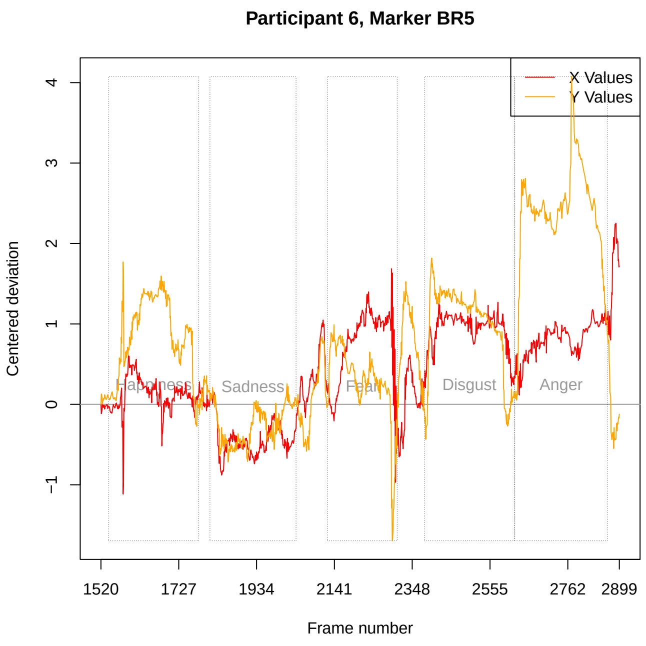
Participant 6, Marker BR5
X Values
Y Values
Happiness
Sadness
Fear
Disgust
Anger
Centered deviation
−1
0
1
2
3
4
1520
1727
1934
2141
2348
2555
2762
2899
Frame number

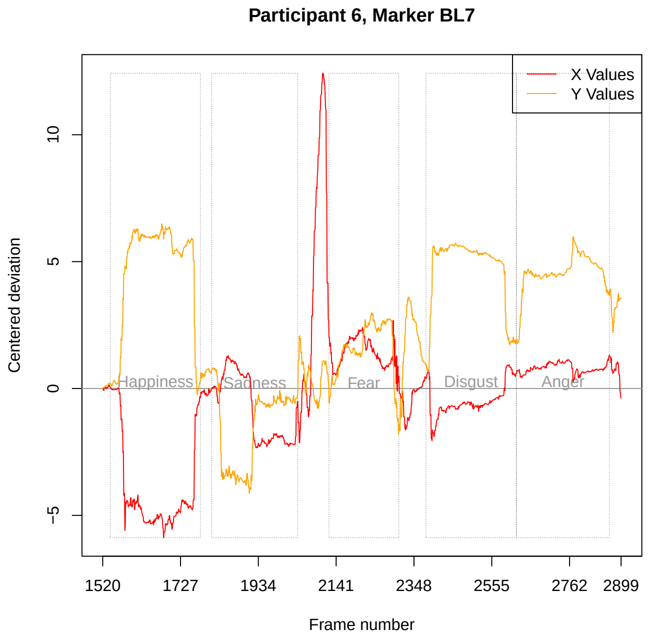
Participant 6, Marker BL7
X Values
Y Values
Happiness
Sadness
Fear
Disgust
Anger
Centered deviation
−5
0
5
10
1520
1727
1934
2141
2348
2555
2762
2899
Frame number

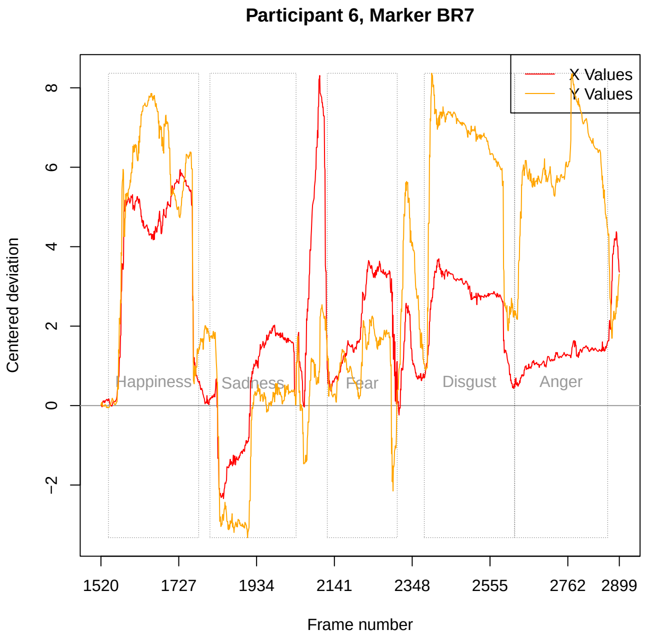
Participant 6, Marker BR7
X Values
Y Values
Happiness
Sadness
Fear
Disgust
Anger
Centered deviation
−2
0
2
4
6
8
1520
1727
1934
2141
2348
2555
2762
2899
Frame number

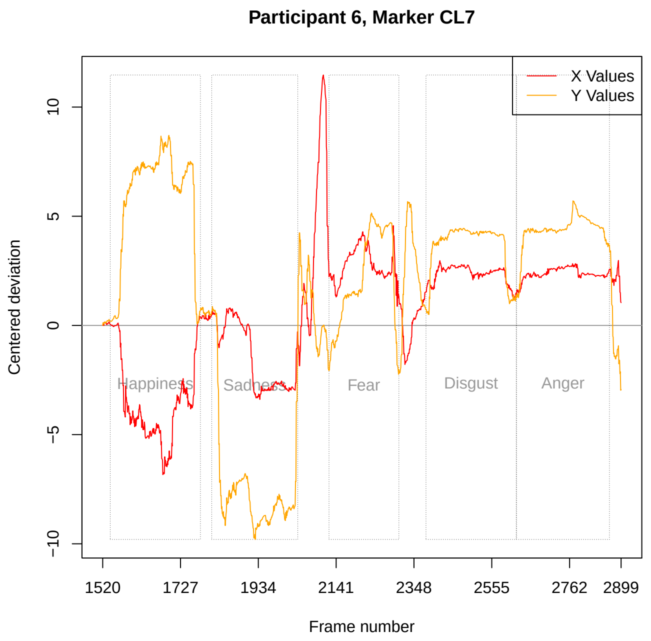
Participant 6, Marker CL7
X Values
Y Values
Happiness
Sadness
Fear
Disgust
Anger
Centered deviation
−10
−5
0
5
10
1520
1727
1934
2141
2348
2555
2762
2899
Frame number

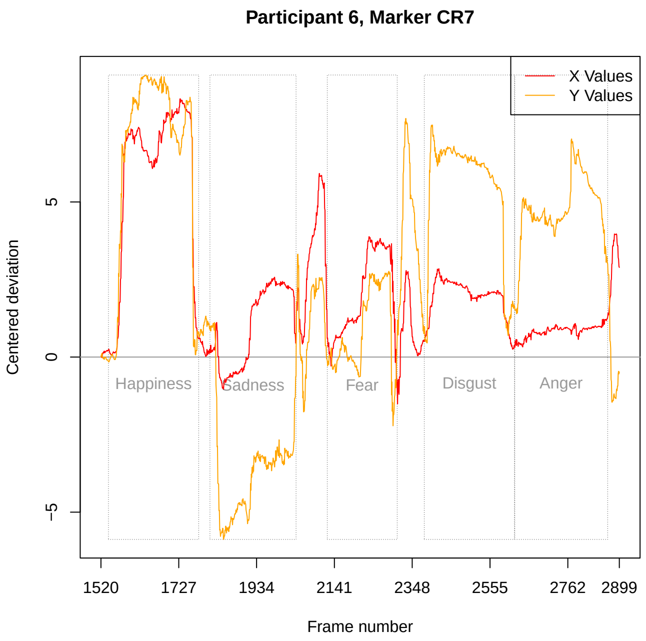
Participant 6, Marker CR7
X Values
Y Values
Happiness
Sadness
Fear
Disgust
Anger
Centered deviation
−5
0
5
1520
1727
1934
2141
2348
2555
2762
2899
Frame number

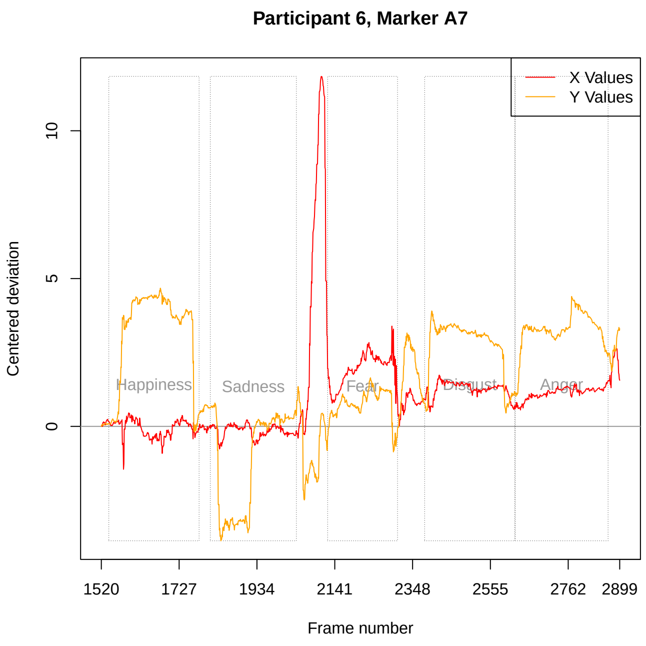

Participant 6, Marker A7
X Values
Y Values
Happiness
Sadness
Fear
Disgust
Anger
Centered deviation
0
5
10
1520
1727
1934
2141
2348
2555
2762
2899
Frame number

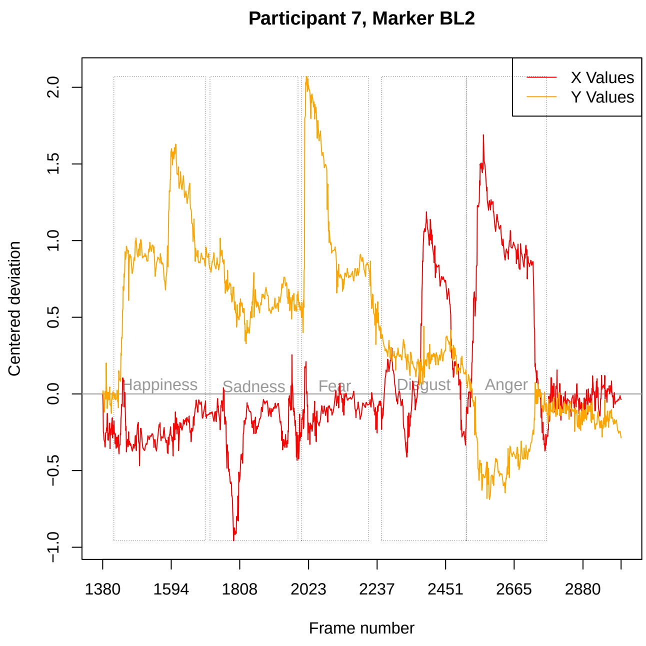

Participant 7, Marker BL2
X Values
Y Values
Happiness
Sadness
Fear
Disgust
Anger
Centered deviation
Frame number

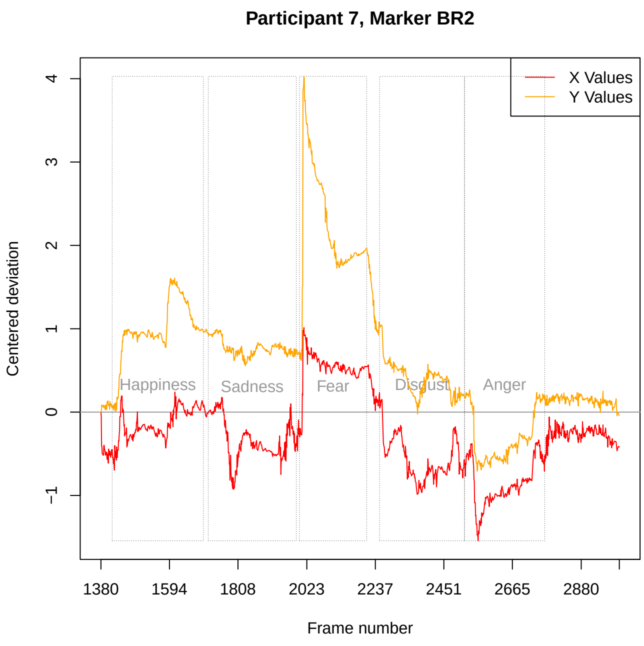

Participant 7, Marker BR2
X Values
Y Values
Happiness
Sadness
Fear
Disgust
Anger
Centered deviation
Frame number

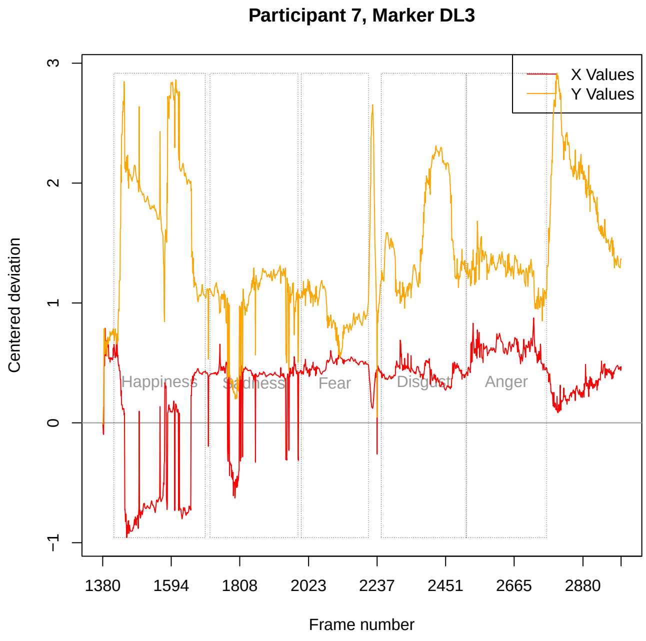

Participant 7, Marker DL3
X Values
Y Values
Happiness
Sadness
Fear
Disgust
Anger
Centered deviation
Frame number

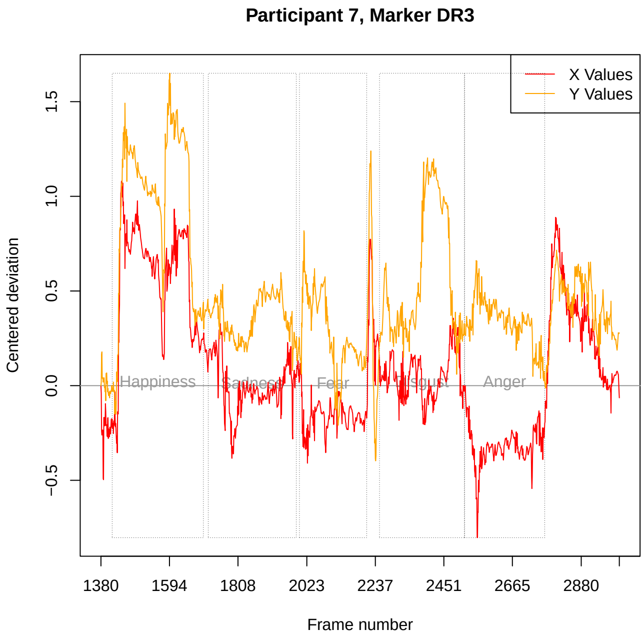

Participant 7, Marker DR3
X Values
Y Values
Happiness
Sadness
Fear
Disgust
Anger
Centered deviation
Frame number

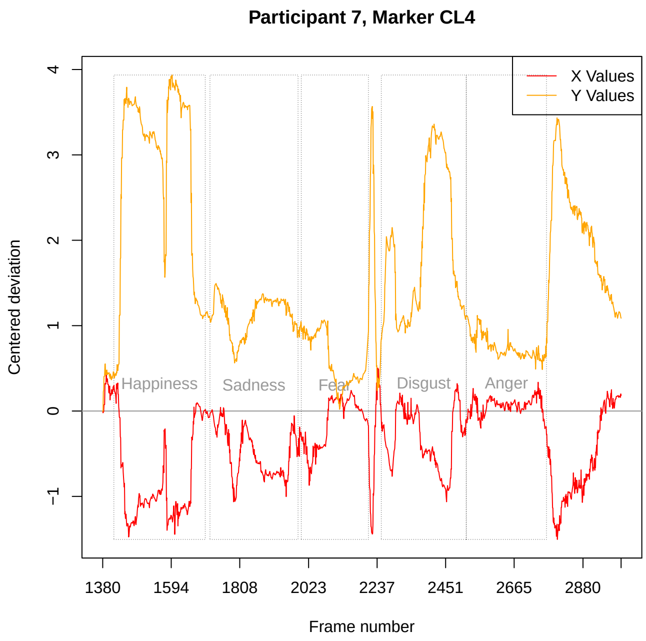

Participant 7, Marker CL4
X Values
Y Values
Happiness
Sadness
Fear
Disgust
Anger
Centered deviation
Frame number

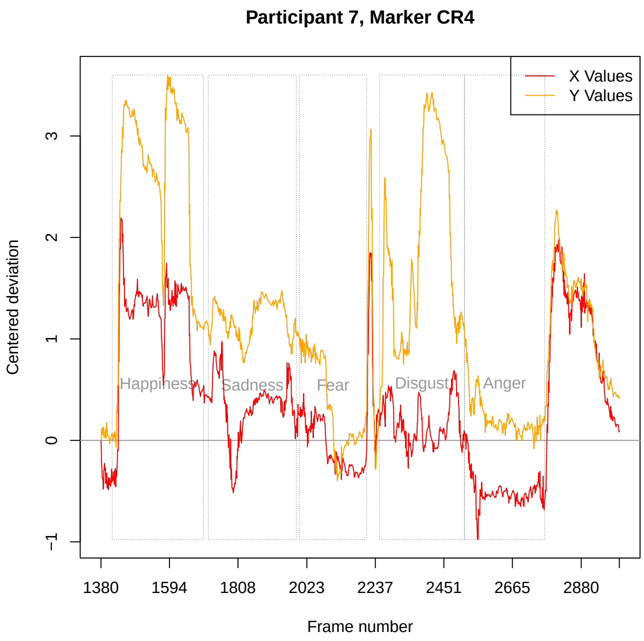

Participant 7, Marker CR4
X Values
Y Values
Happiness
Sadness
Fear
Disgust
Anger
Centered deviation
Frame number

Participant 7, Marker BL5

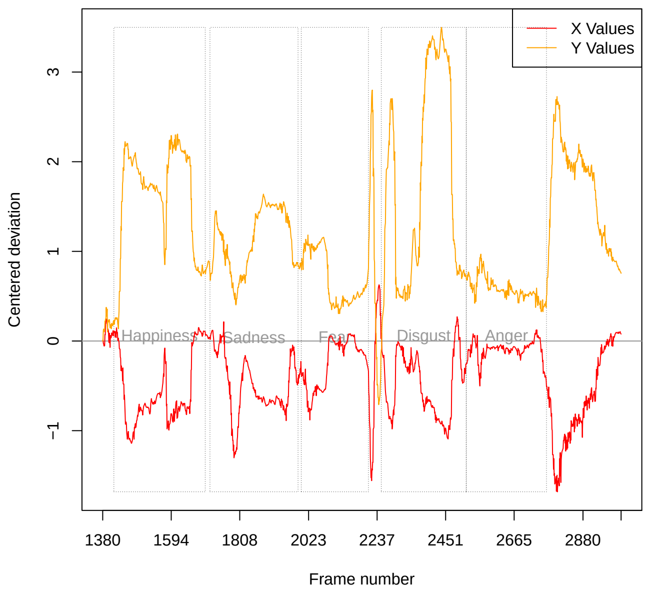

Participant 7, Marker BR5

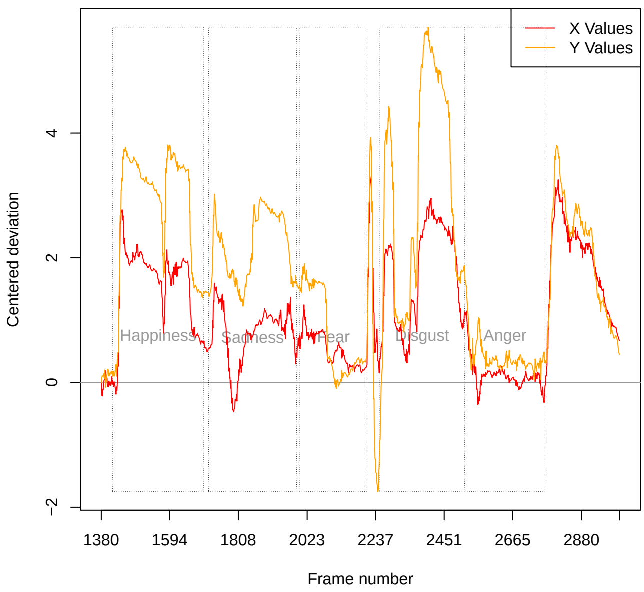

Participant 7, Marker BL7

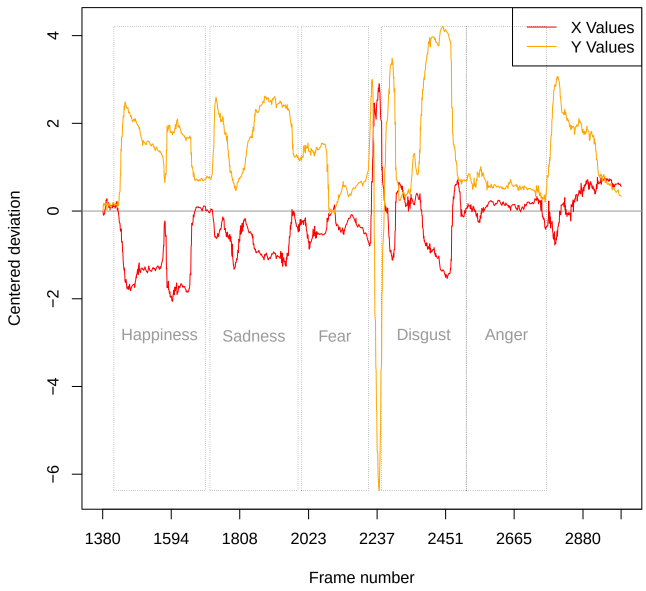

Participant 7, Marker BR7

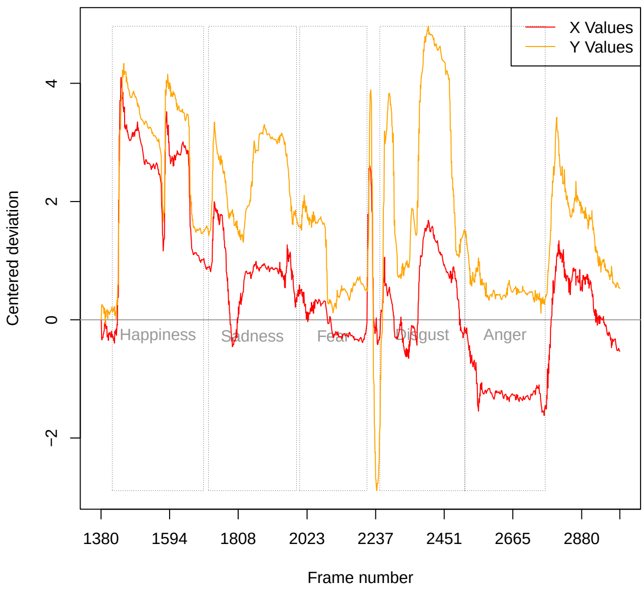

Participant 7, Marker CL7

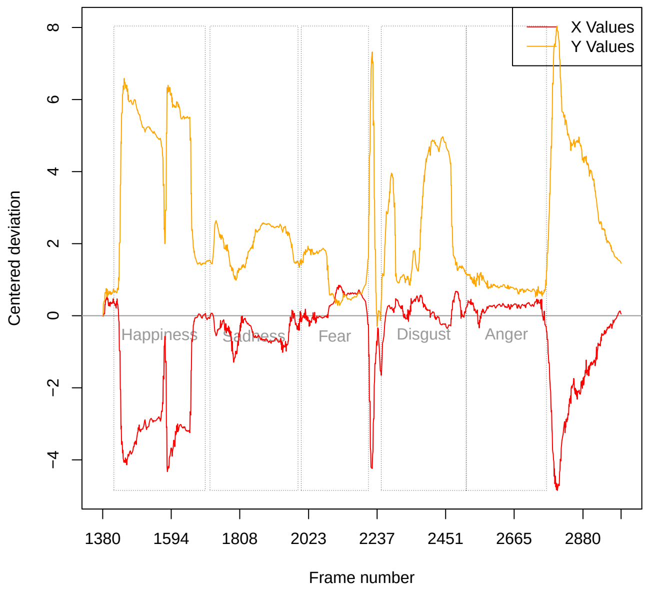

Participant 7, Marker CR7

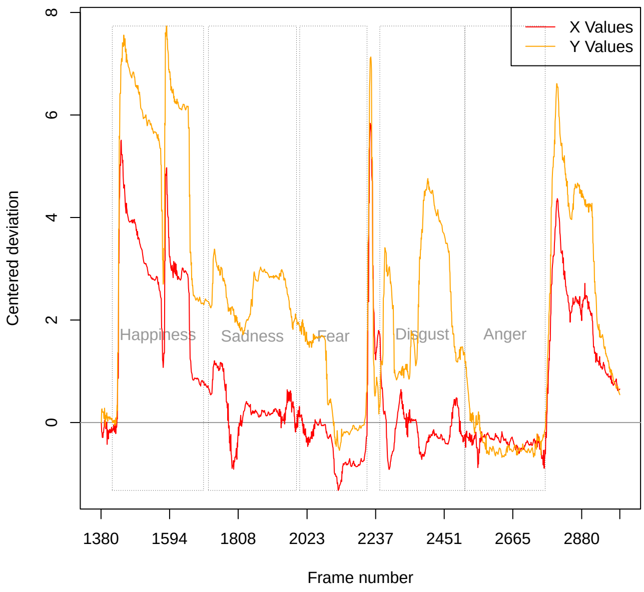

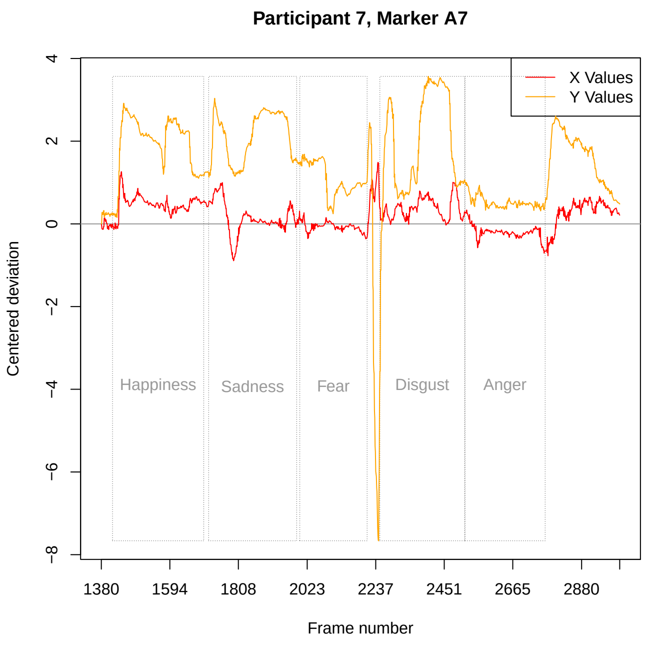

Participant 7, Marker A7
X Values
Y Values
Happiness
Sadness
Fear
Disgust
Anger
Centered deviation
Frame number
4
2
0
−2
−4
−6
−8
1380
1594
1808
2023
2237
2451
2665
2880

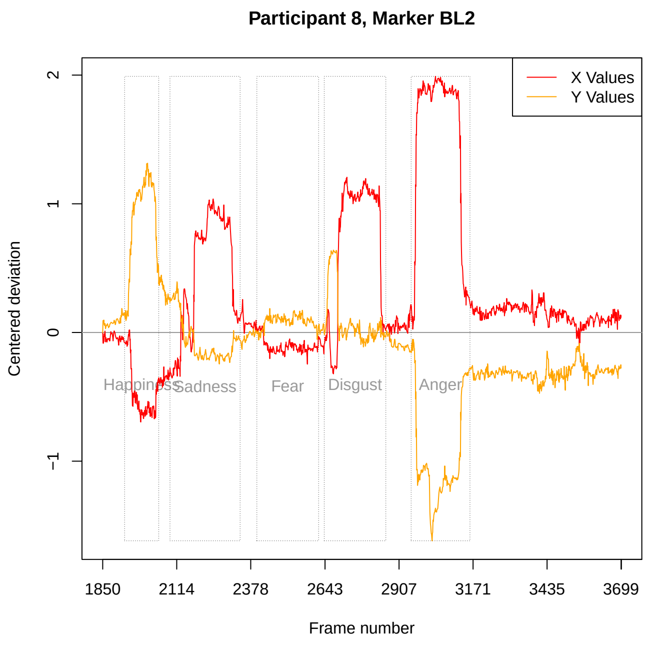
Participant 8, Marker BL2
X Values
Y Values
Centered deviation
Happiness
Sadness
Fear
Disgust
Anger
1850
2114
2378
2643
2907
3171
3435
3699
Frame number

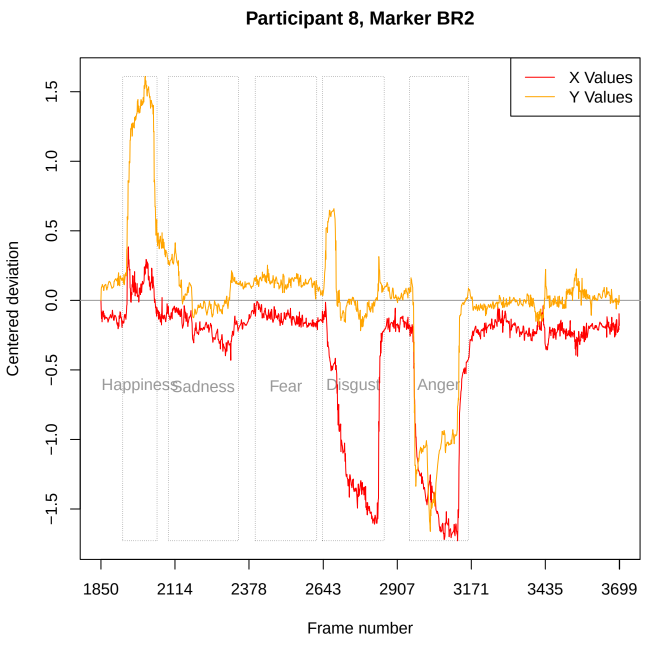
Participant 8, Marker BR2
X Values
Y Values
Centered deviation
Happiness
Sadness
Fear
Disgust
Anger
Frame number

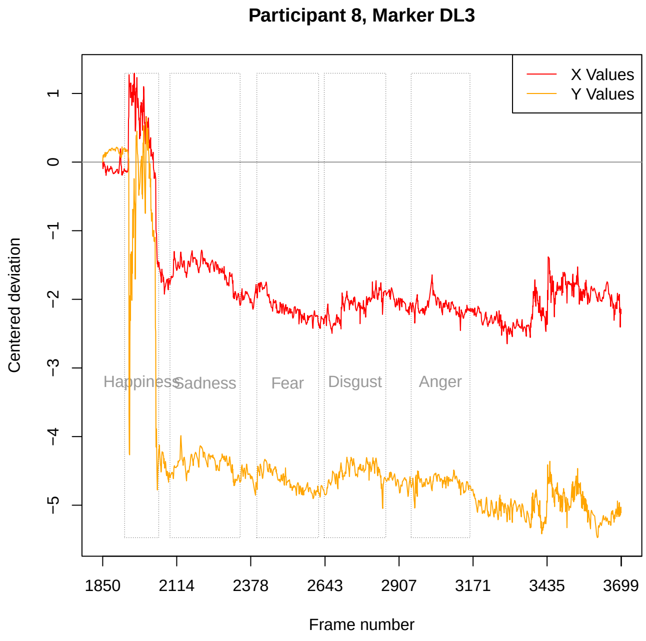
Participant 8, Marker DL3
X Values
Y Values
Centered deviation
Happiness
Sadness
Fear
Disgust
Anger
Frame number

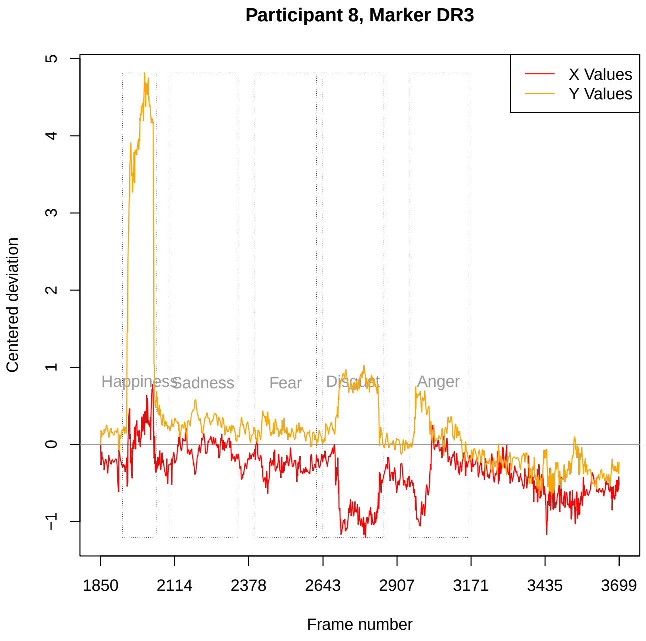
Participant 8, Marker DR3
X Values
Y Values
Centered deviation
Happiness
Sadness
Fear
Disgust
Anger
Frame number

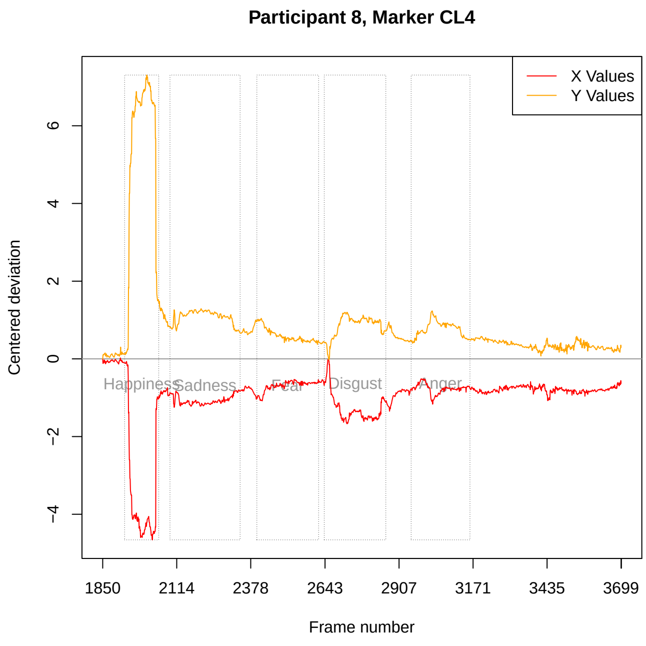
Participant 8, Marker CL4
X Values
Y Values
Centered deviation
Happiness
Sadness
Fear
Disgust
Anger
Frame number

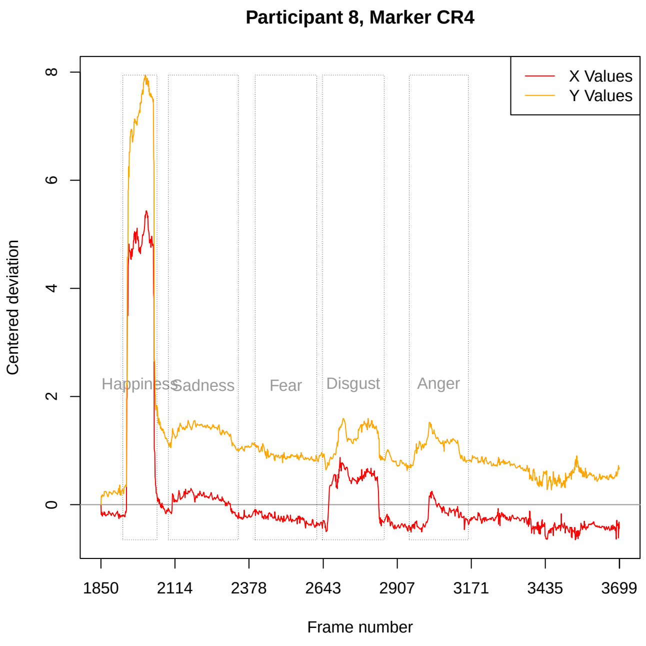
Participant 8, Marker CR4
X Values
Y Values
Centered deviation
Happiness
Sadness
Fear
Disgust
Anger
Frame number

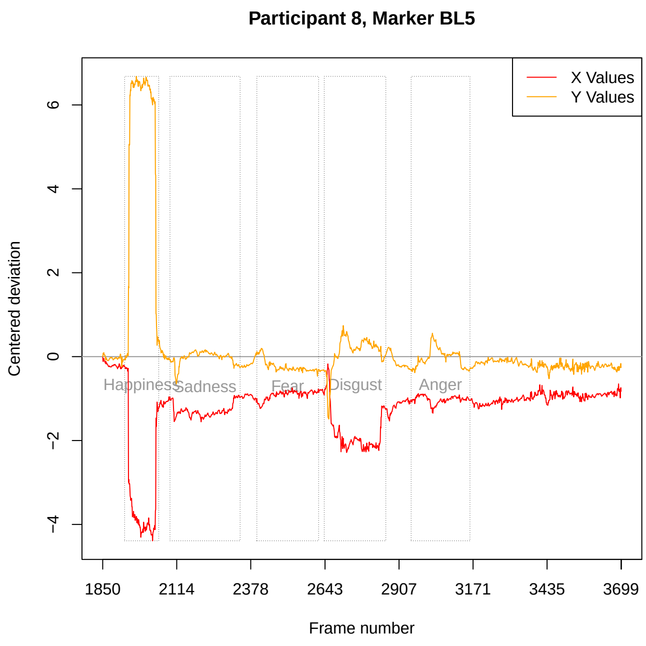

Participant 8, Marker BL5
X Values
Y Values
Centered deviation
Happiness
Sadness
Fear
Disgust
Anger
Frame number

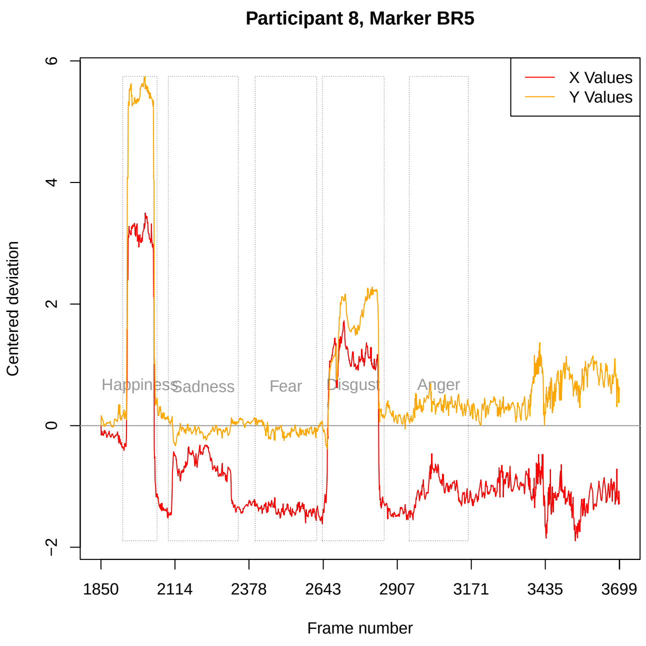

Participant 8, Marker BR5
X Values
Y Values
Centered deviation
Happiness
Sadness
Fear
Disgust
Anger
Frame number

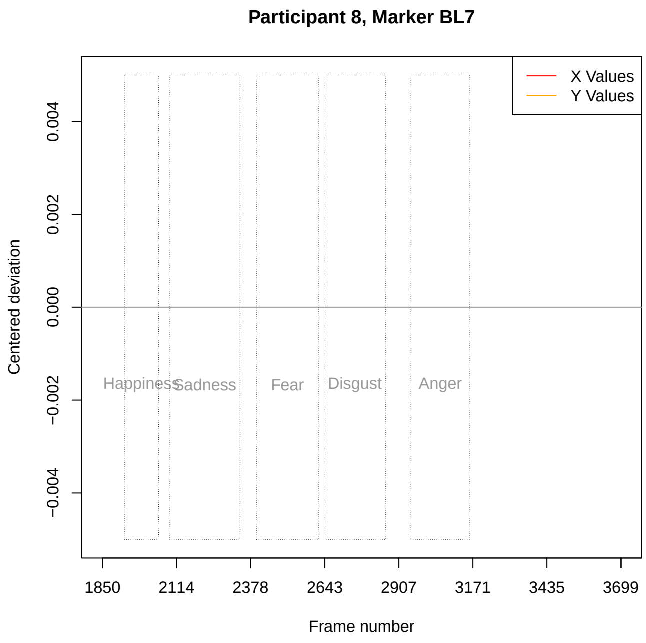

Participant 8, Marker BL7
X Values
Y Values
Centered deviation
Happiness
Sadness
Fear
Disgust
Anger
Frame number

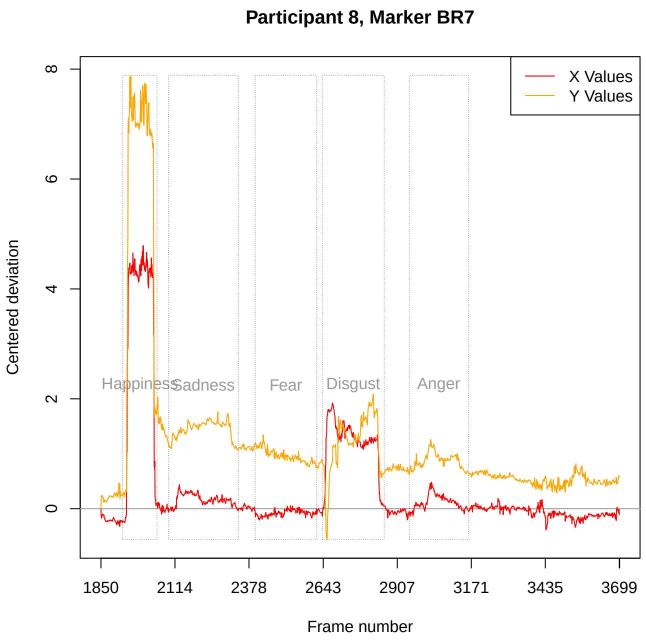

Participant 8, Marker BR7
X Values
Y Values
Centered deviation
Happiness
Sadness
Fear
Disgust
Anger
Frame number

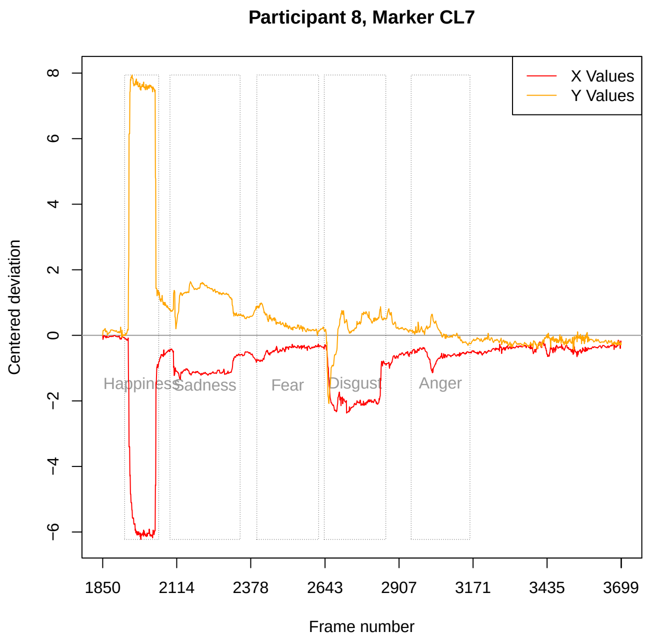

Participant 8, Marker CL7
X Values
Y Values
Centered deviation
Happiness
Sadness
Fear
Disgust
Anger
Frame number

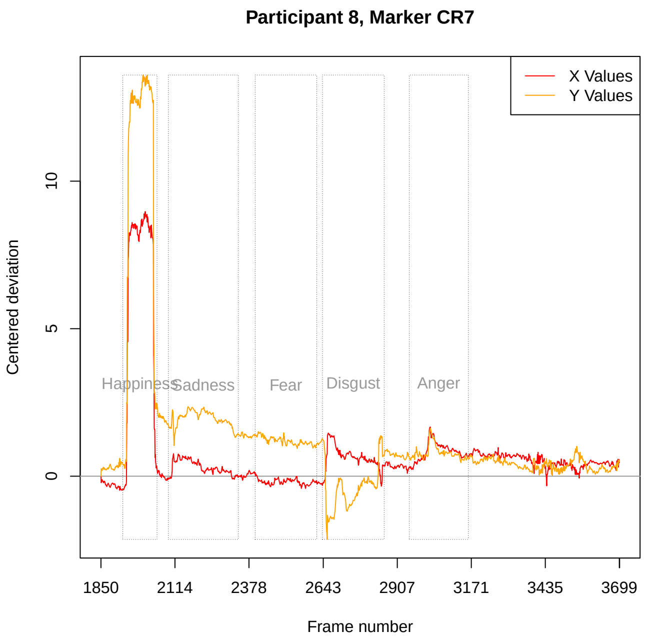

Participant 8, Marker CR7
X Values
Y Values
Centered deviation
Happiness
Sadness
Fear
Disgust
Anger
Frame number

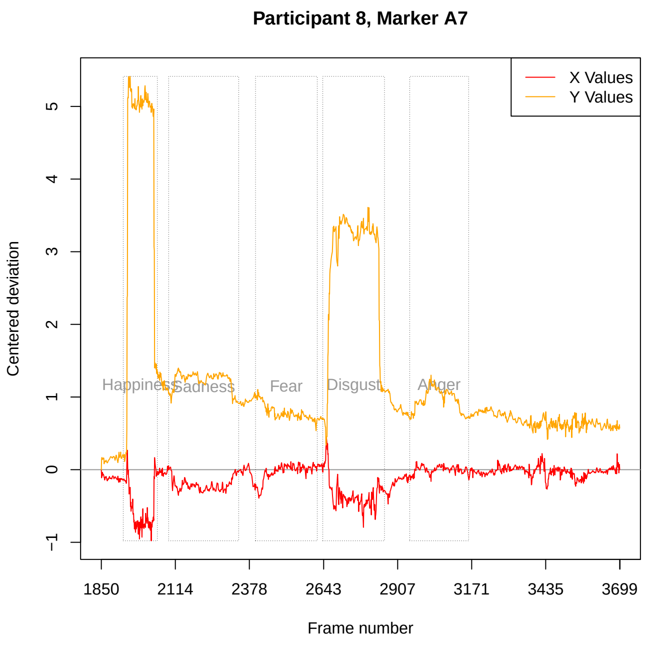

Participant 8, Marker A7
X Values
Y Values
Happiness
Sadness
Fear
Disgust
Anger
Centered deviation
−1
0
1
2
3
4
5
1850
2114
2378
2643
2907
3171
3435
3699
Frame number

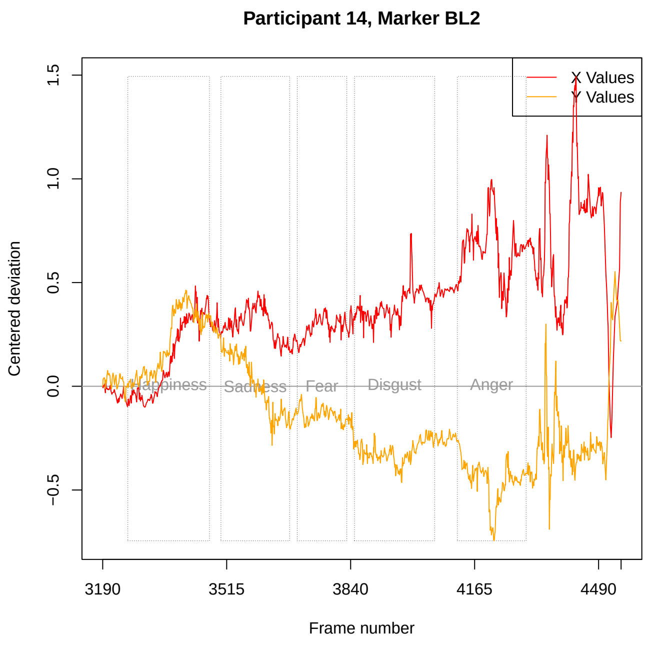
Participant 14, Marker BL2
X Values
Y Values
Happiness
Sadness
Fear
Disgust
Anger
Centered deviation
1.5
1.0
0.5
0.0
−0.5
3190
3515
3840
4165
4490
Frame number

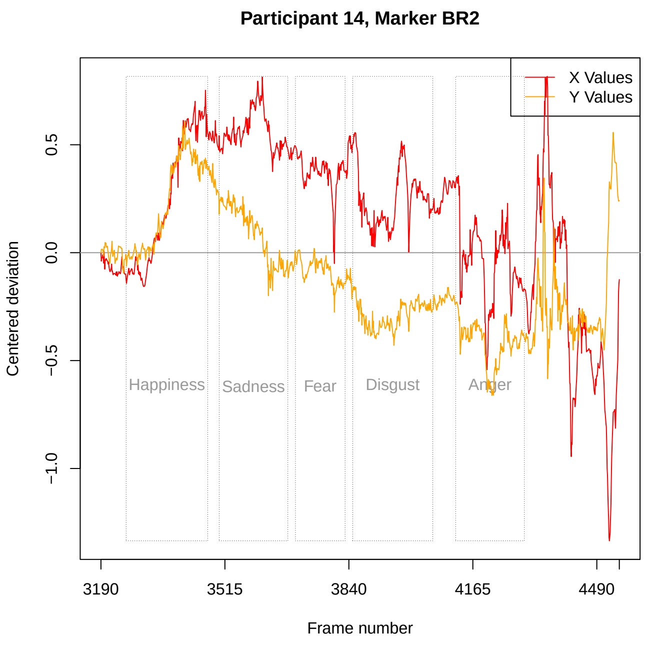
Participant 14, Marker BR2
X Values
Y Values
Happiness
Sadness
Fear
Disgust
Anger
Centered deviation
0.5
0.0
−0.5
−1.0
3190
3515
3840
4165
4490
Frame number

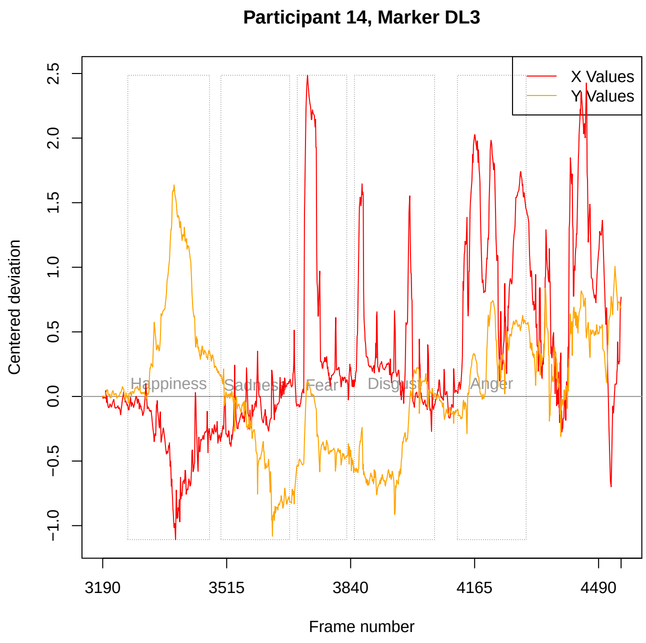
Participant 14, Marker DL3
X Values
Y Values
Happiness
Sadness
Fear
Disgust
Anger
Centered deviation
2.5
2.0
1.5
1.0
0.5
0.0
−0.5
−1.0
3190
3515
3840
4165
4490
Frame number

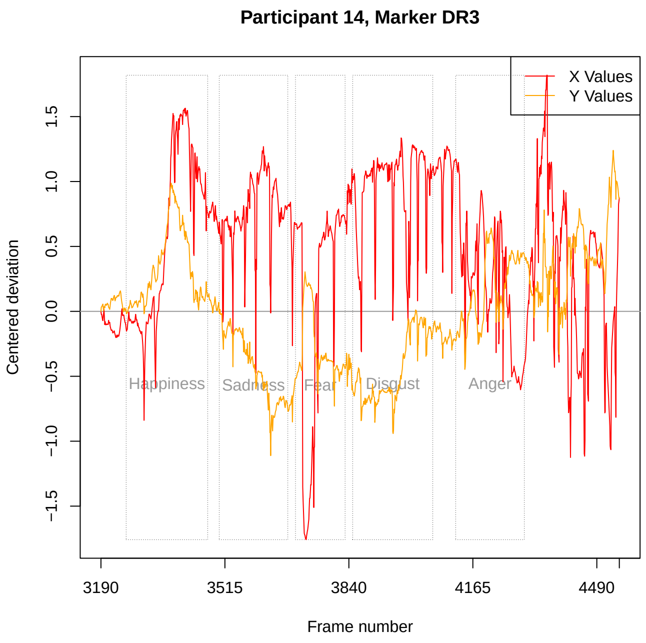
Participant 14, Marker DR3
X Values
Y Values
Happiness
Sadness
Fear
Disgust
Anger
Centered deviation
1.5
1.0
0.5
0.0
−0.5
−1.0
−1.5
3190
3515
3840
4165
4490
Frame number

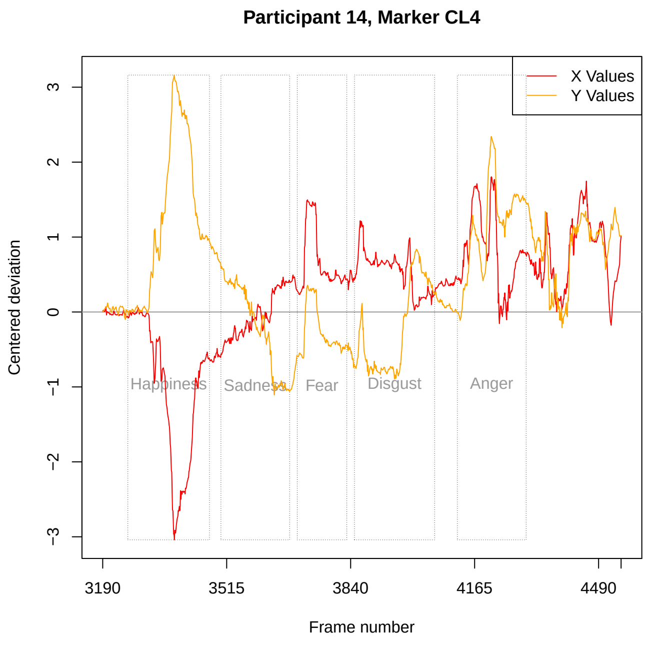
Participant 14, Marker CL4
X Values
Y Values
Happiness
Sadness
Fear
Disgust
Anger
Centered deviation
3
2
1
0
−1
−2
−3
3190
3515
3840
4165
4490
Frame number

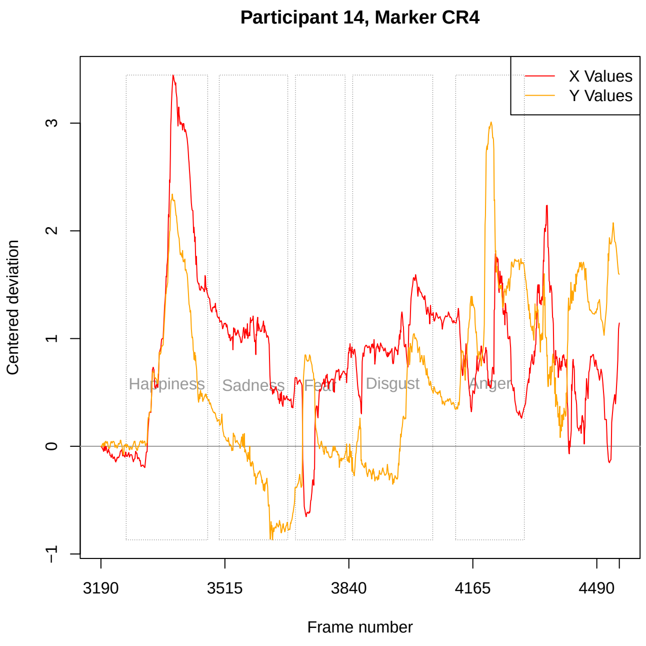
Participant 14, Marker CR4
X Values
Y Values
Happiness
Sadness
Fear
Disgust
Anger
Centered deviation
3
2
1
0
−1
3190
3515
3840
4165
4490
Frame number

Participant 14, Marker BL5

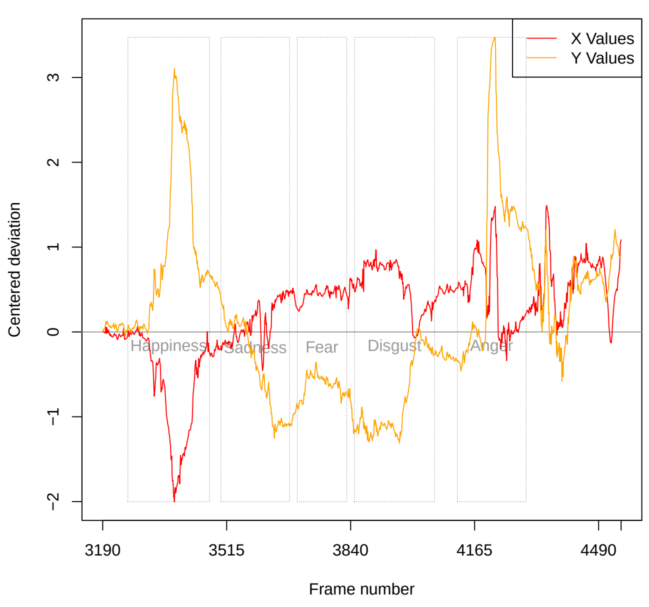

Participant 14, Marker BR5

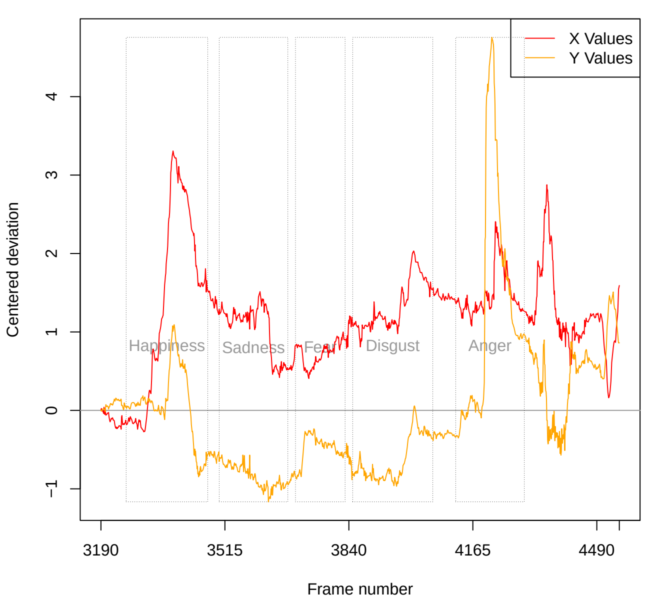

Participant 14, Marker BL7

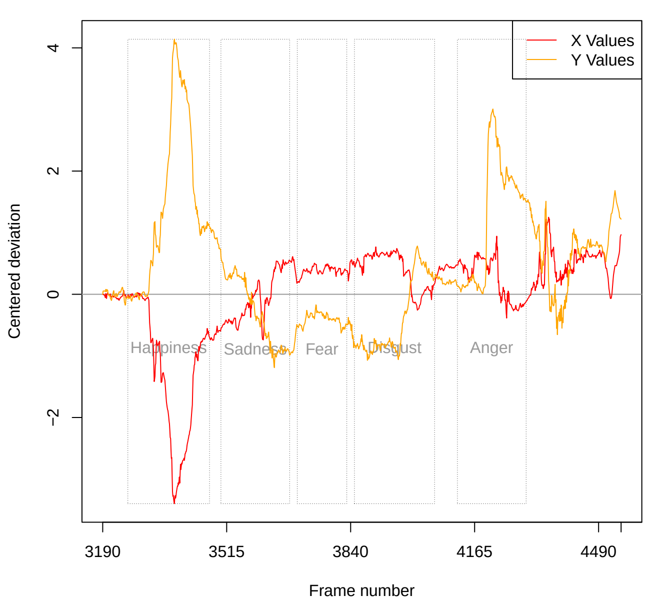

Participant 14, Marker BR7

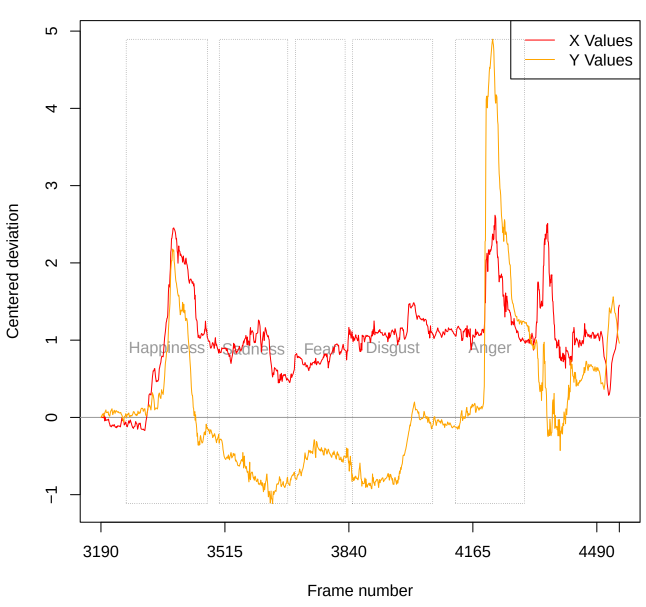

Participant 14, Marker CL7

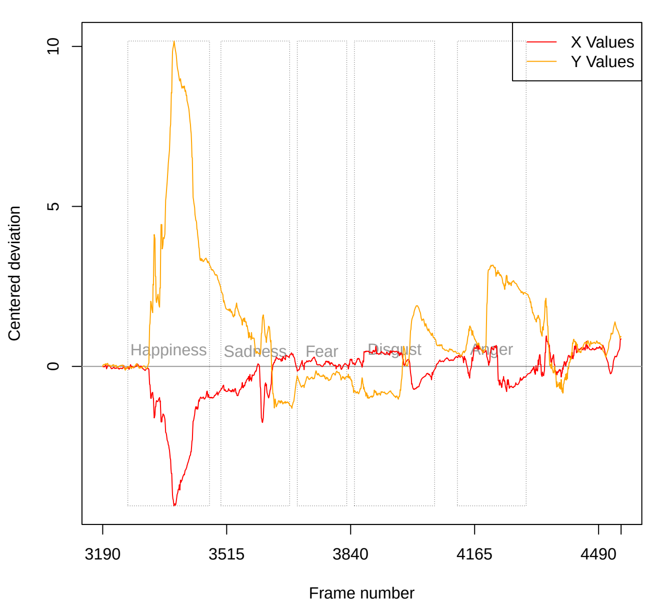

Participant 14, Marker CR7

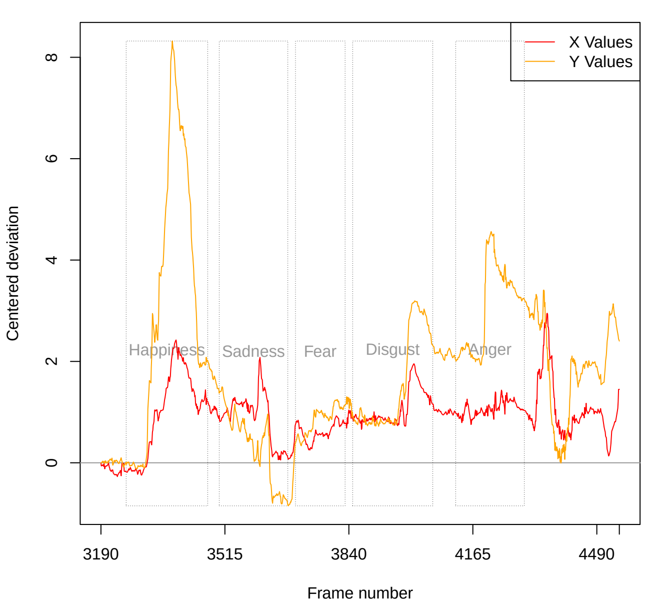

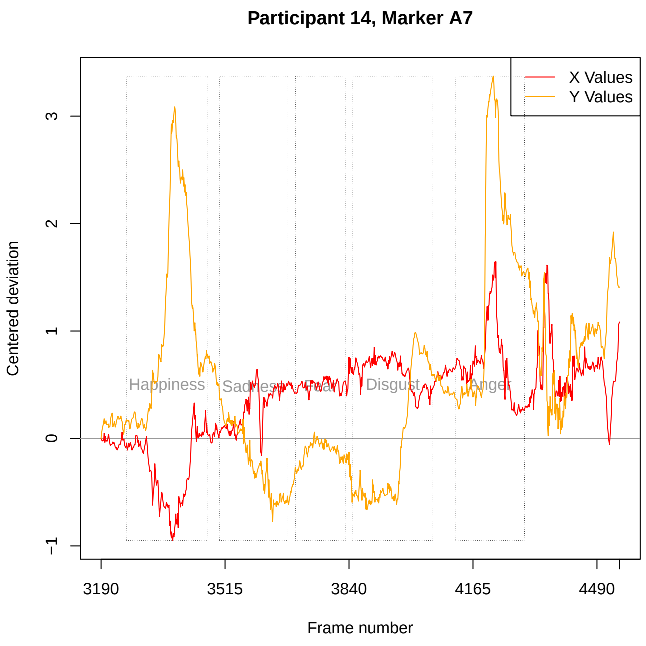
Participant 14, Marker A7
X Values
Y Values
Happiness
Sadness
Fear
Disgust
Anger
Centered deviation
−1
0
1
2
3
3190
3515
3840
4165
4490
Frame number

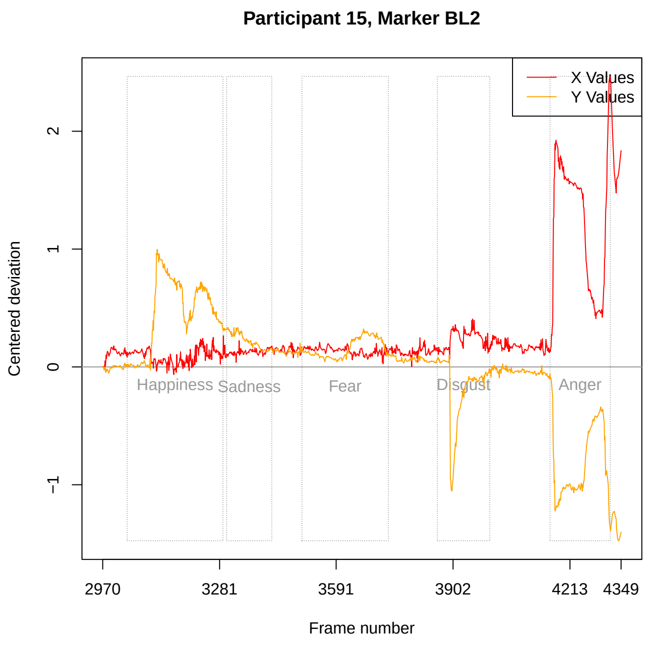
Participant 15, Marker BL2
X Values
Y Values
Centered deviation
Frame number
2970
3281
3591
3902
4213
4349
Happiness
Sadness
Fear
Disgust
Anger

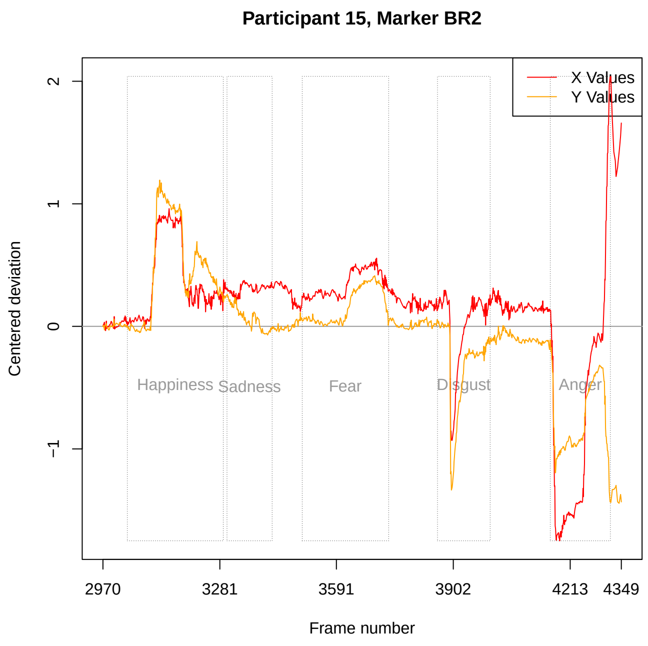
Participant 15, Marker BR2
X Values
Y Values
Centered deviation
Frame number
2970
3281
3591
3902
4213
4349
Happiness
Sadness
Fear
Disgust
Anger

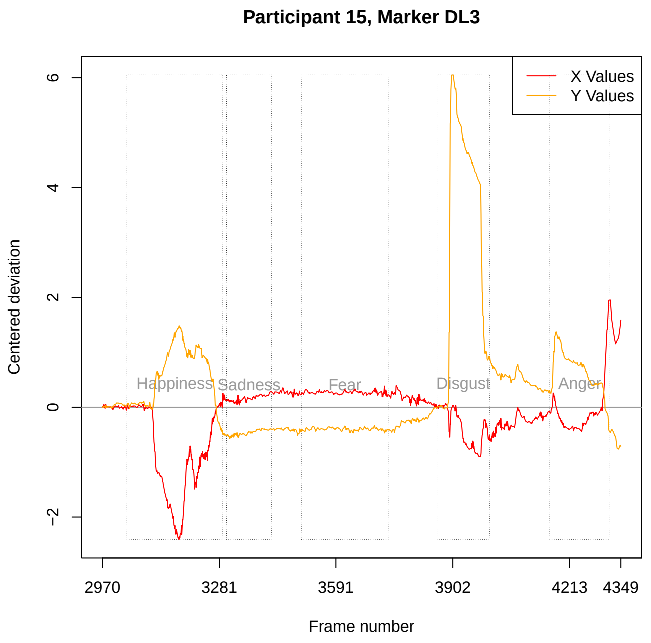
Participant 15, Marker DL3
X Values
Y Values
Centered deviation
Frame number
2970
3281
3591
3902
4213
4349
Happiness
Sadness
Fear
Disgust
Anger

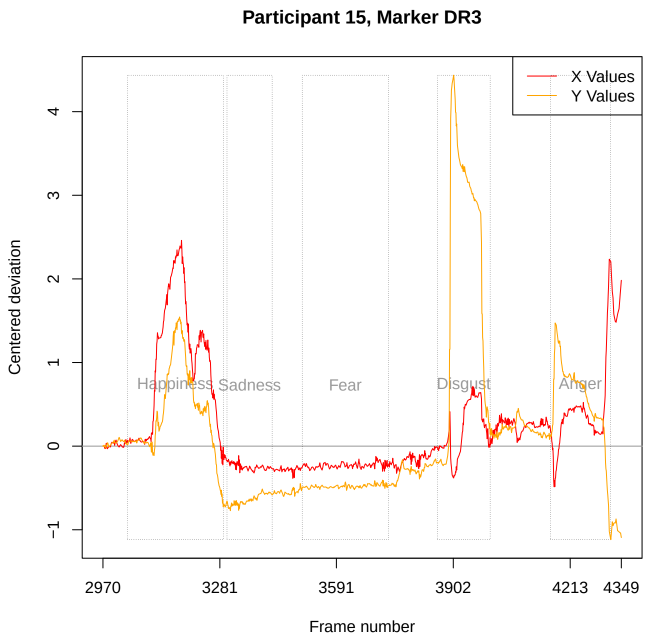
Participant 15, Marker DR3
X Values
Y Values
Centered deviation
Frame number
2970
3281
3591
3902
4213
4349
Happiness
Sadness
Fear
Disgust
Anger

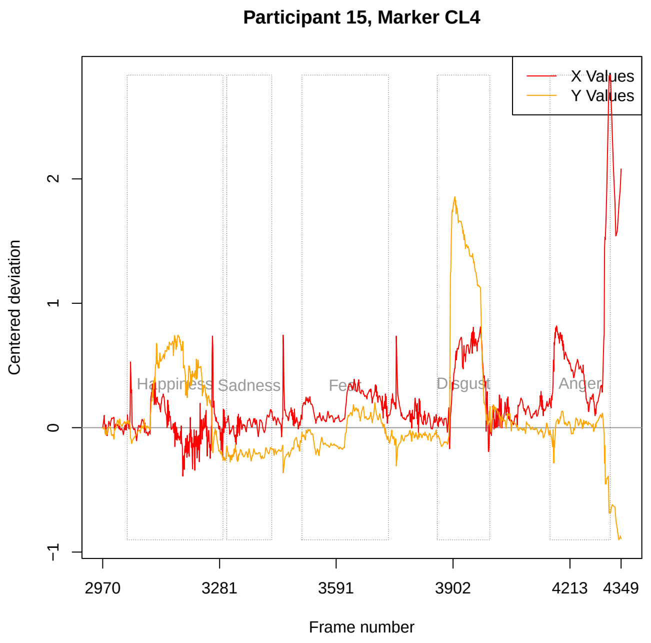
Participant 15, Marker CL4
X Values
Y Values
Centered deviation
Frame number
2970
3281
3591
3902
4213
4349
Happiness
Sadness
Fear
Disgust
Anger

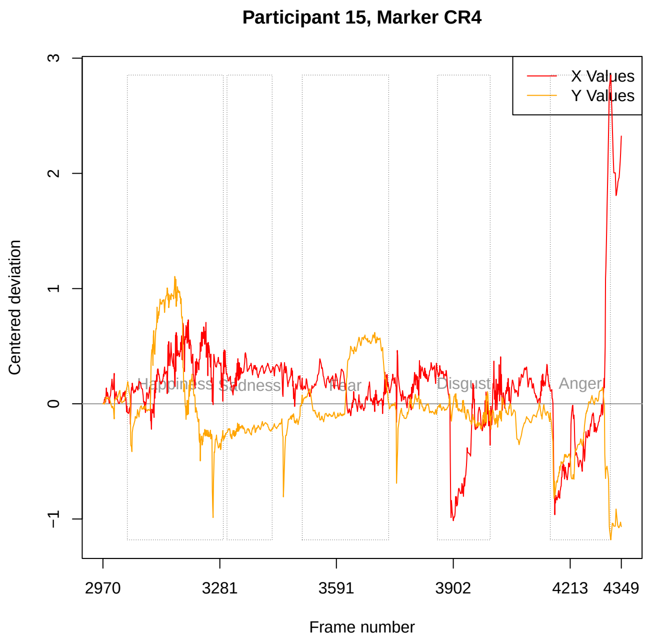
Participant 15, Marker CR4
X Values
Y Values
Centered deviation
Frame number
2970
3281
3591
3902
4213
4349
Happiness
Sadness
Fear
Disgust
Anger

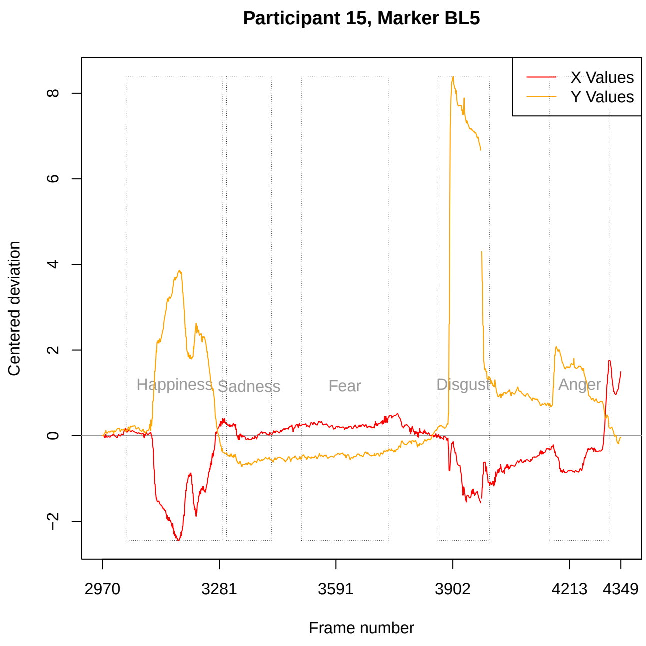
Participant 15, Marker BL5
X Values
Y Values
Centered deviation
Frame number
2970 3281 3591 3902 4213 4349
-2 0 2 4 6 8
Happiness
Sadness
Fear
Disgust
Anger

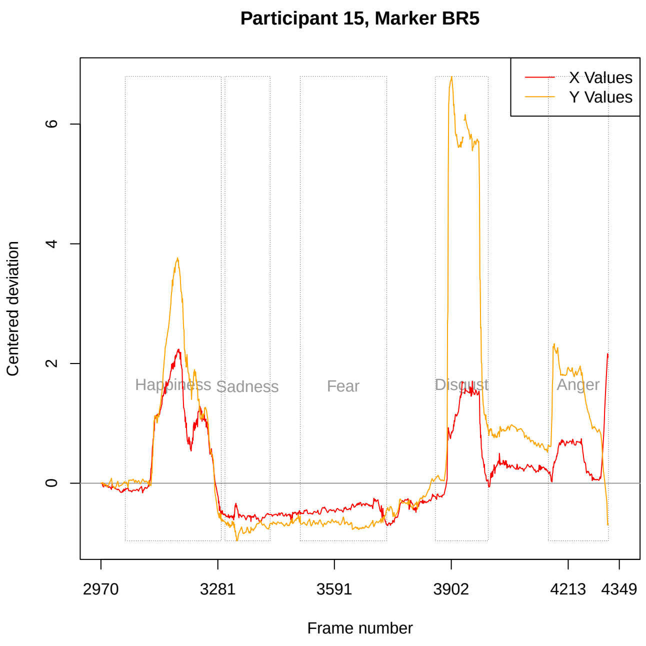
Participant 15, Marker BR5
X Values
Y Values
Centered deviation
Frame number
2970 3281 3591 3902 4213 4349
0 2 4 6
Happiness
Sadness
Fear
Disgust
Anger

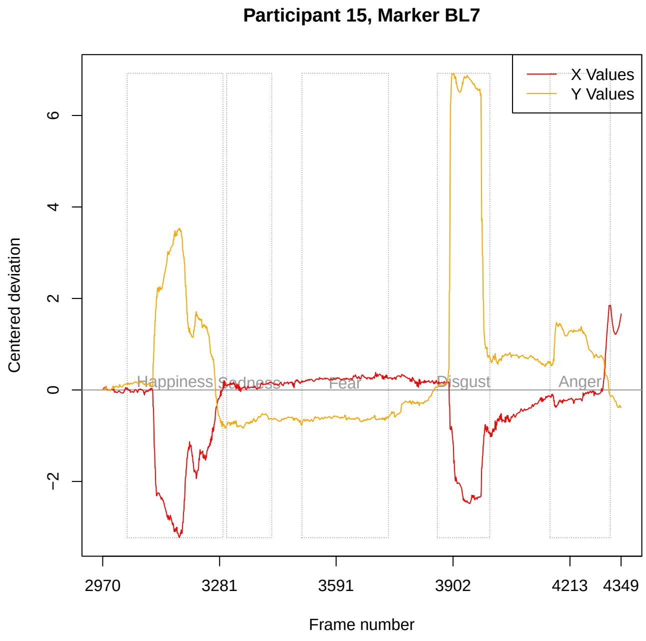
Participant 15, Marker BL7
X Values
Y Values
Centered deviation
Frame number
2970 3281 3591 3902 4213 4349
-2 0 2 4 6
Happiness
Sadness
Fear
Disgust
Anger

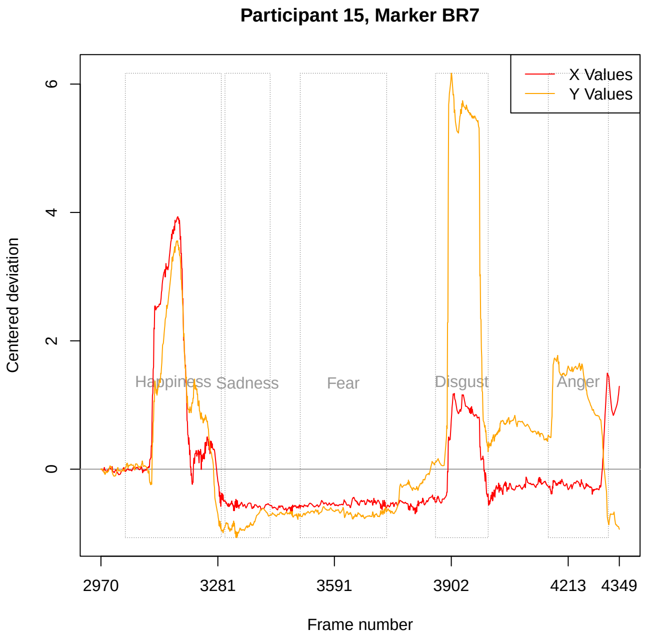
Participant 15, Marker BR7
X Values
Y Values
Centered deviation
Frame number
2970 3281 3591 3902 4213 4349
0 2 4 6
Happiness
Sadness
Fear
Disgust
Anger

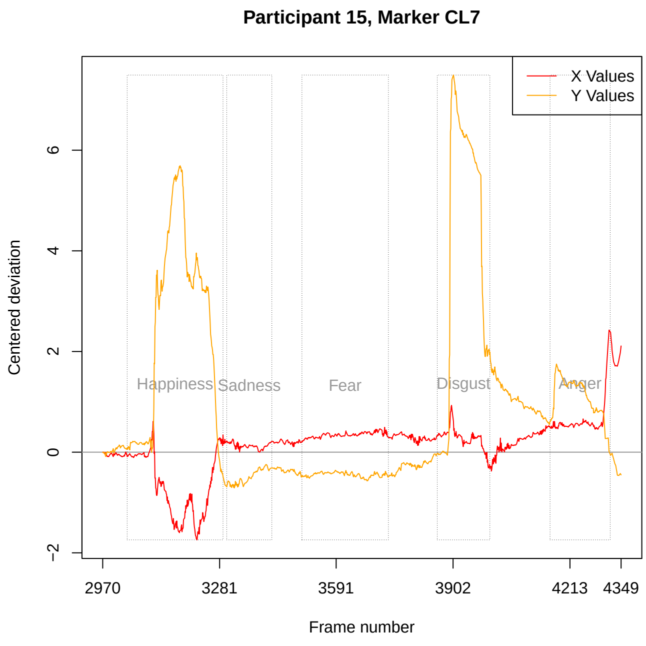
Participant 15, Marker CL7
X Values
Y Values
Centered deviation
Frame number
2970 3281 3591 3902 4213 4349
-2 0 2 4 6
Happiness
Sadness
Fear
Disgust
Anger

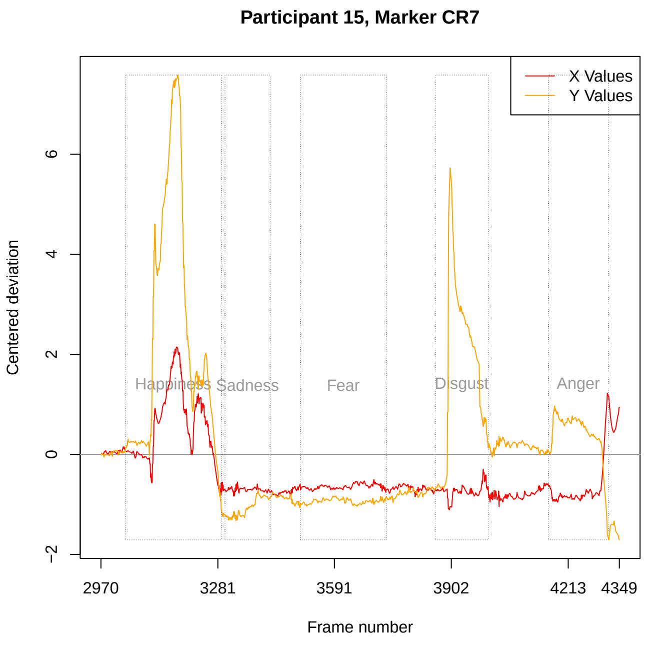
Participant 15, Marker CR7
X Values
Y Values
Centered deviation
Frame number
2970 3281 3591 3902 4213 4349
-2 0 2 4 6
Happiness
Sadness
Fear
Disgust
Anger

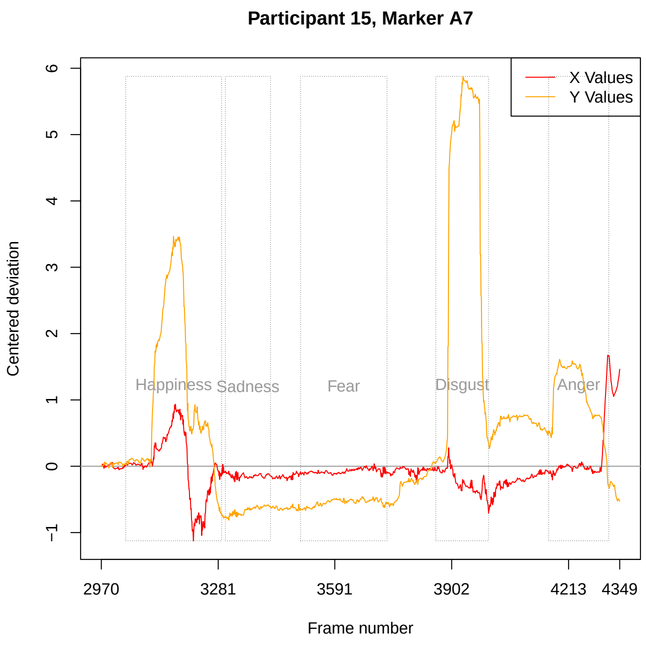

Participant 15, Marker A7
X Values
Y Values
Happiness
Sadness
Fear
Disgust
Anger
Centered deviation
Frame number
2970
3281
3591
3902
4213
4349
−1
0
1
2
3
4
5
6

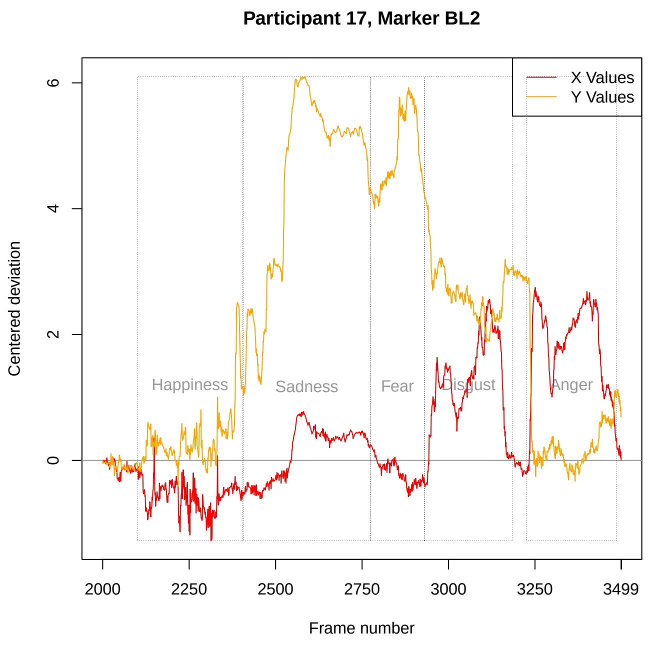
Participant 17, Marker BL2
X Values
Y Values
Centered deviation
Frame number
Happiness
Sadness
Fear
Disgust
Anger
0
2
4
6
2000
2250
2500
2750
3000
3250
3499

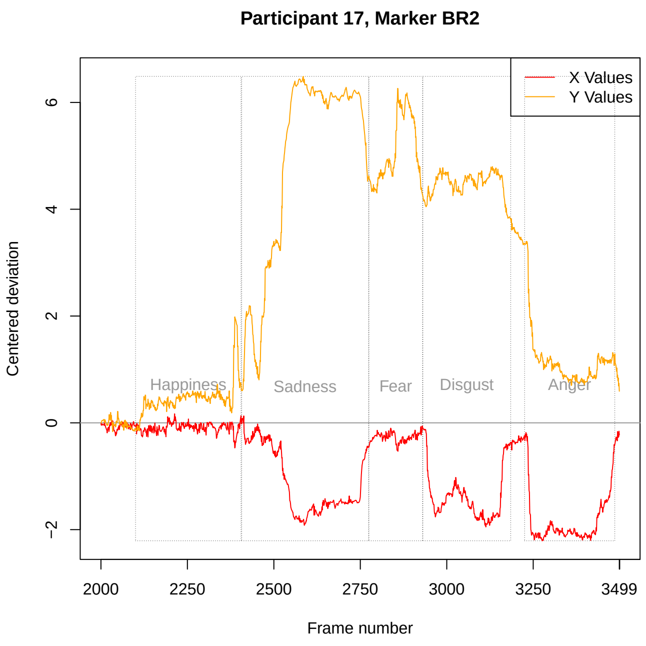
Participant 17, Marker BR2
X Values
Y Values
Centered deviation
Frame number
Happiness
Sadness
Fear
Disgust
Anger
−2
0
2
4
6
2000
2250
2500
2750
3000
3250
3499

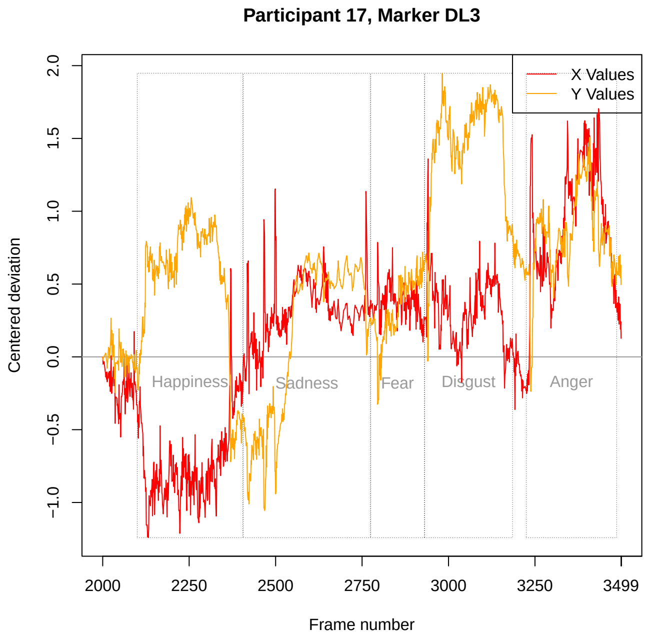
Participant 17, Marker DL3
X Values
Y Values
Centered deviation
Frame number
Happiness
Sadness
Fear
Disgust
Anger
−1.0
−0.5
0.0
0.5
1.0
1.5
2.0
2000
2250
2500
2750
3000
3250
3499

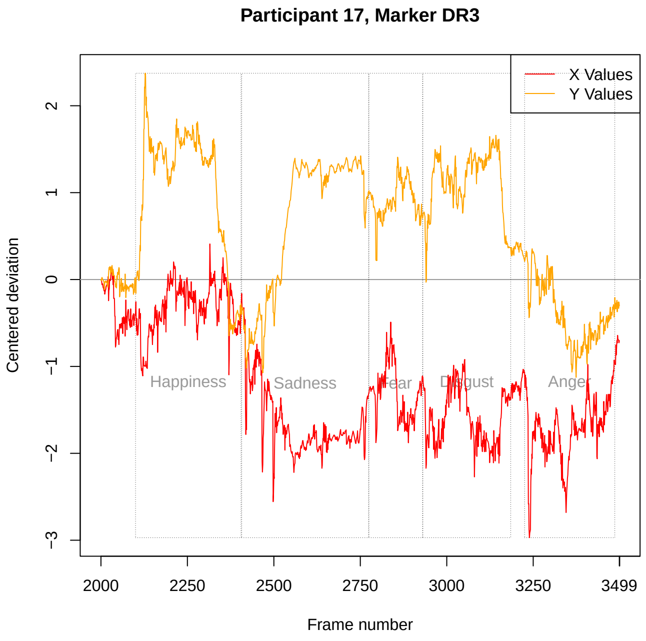
Participant 17, Marker DR3
X Values
Y Values
Centered deviation
Frame number
Happiness
Sadness
Fear
Disgust
Anger
−3
−2
−1
0
1
2
2000
2250
2500
2750
3000
3250
3499

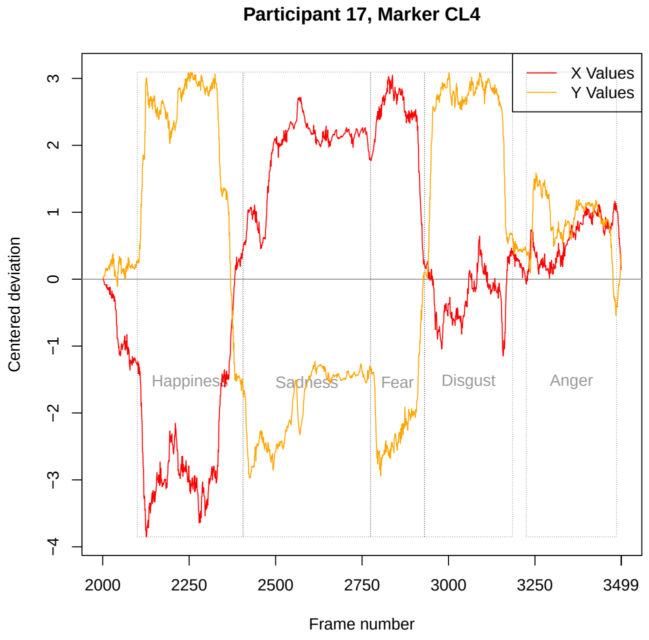
Participant 17, Marker CL4
X Values
Y Values
Centered deviation
Frame number
Happiness
Sadness
Fear
Disgust
Anger
−4
−3
−2
−1
0
1
2
3
2000
2250
2500
2750
3000
3250
3499

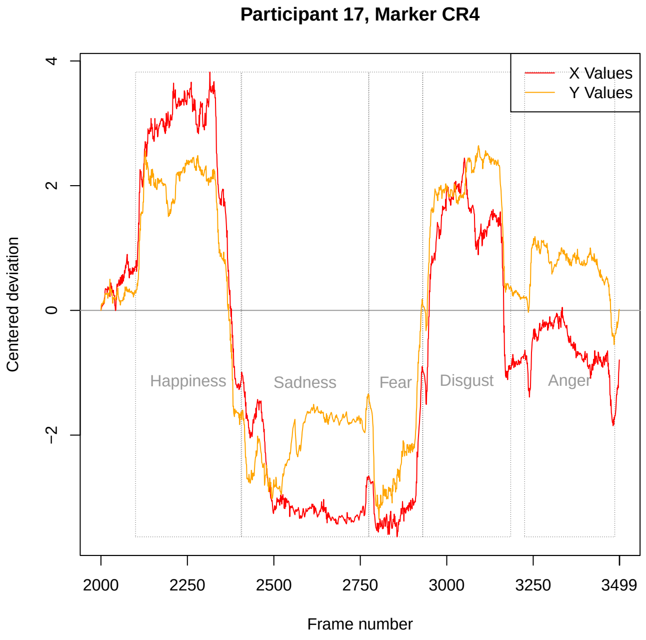
Participant 17, Marker CR4
X Values
Y Values
Centered deviation
Frame number
Happiness
Sadness
Fear
Disgust
Anger
−2
0
2
4
2000
2250
2500
2750
3000
3250
3499

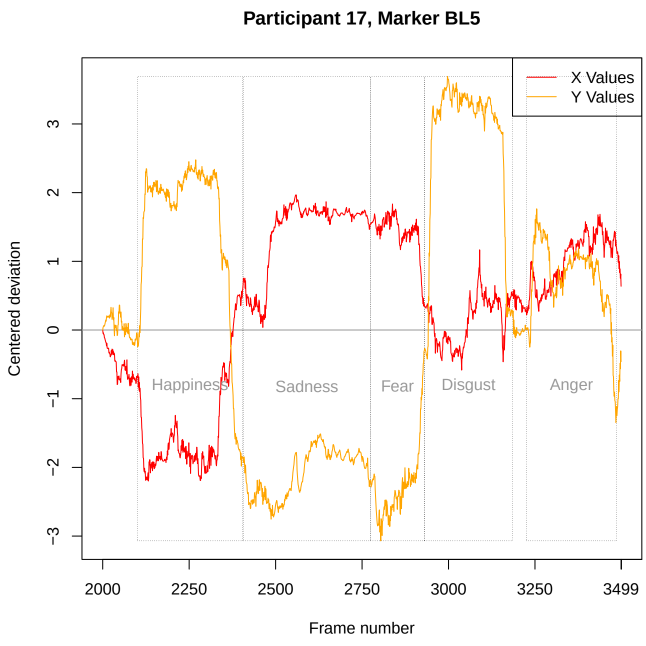
Participant 17, Marker BL5
X Values
Y Values
Happiness
Sadness
Fear
Disgust
Anger
Centered deviation
Frame number
2000
2250
2500
2750
3000
3250
3499
−3
−2
−1
0
1
2
3

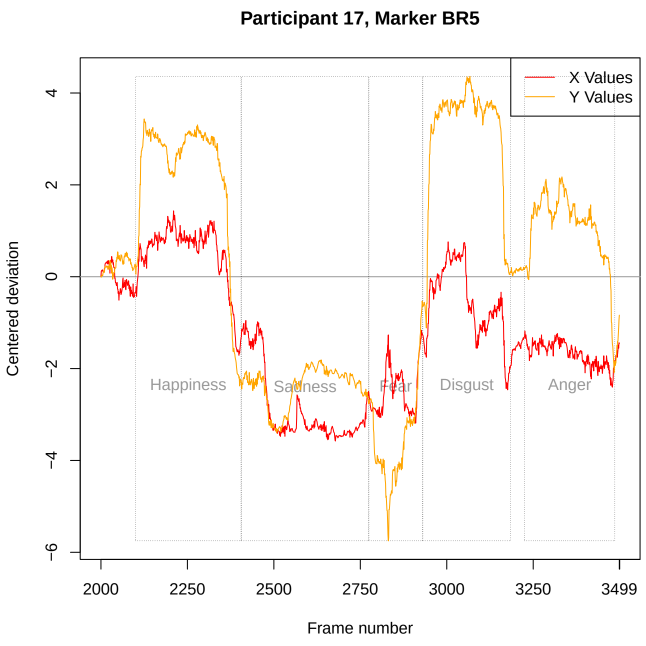
Participant 17, Marker BR5
X Values
Y Values
Happiness
Sadness
Fear
Disgust
Anger
Centered deviation
Frame number
2000
2250
2500
2750
3000
3250
3499
−6
−4
−2
0
2
4

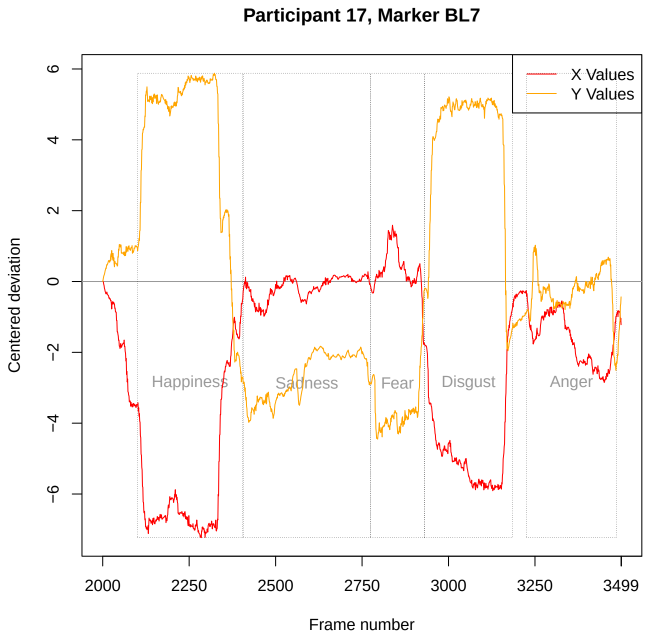
Participant 17, Marker BL7
X Values
Y Values
Happiness
Sadness
Fear
Disgust
Anger
Centered deviation
Frame number
2000
2250
2500
2750
3000
3250
3499
−6
−4
−2
0
2
4
6

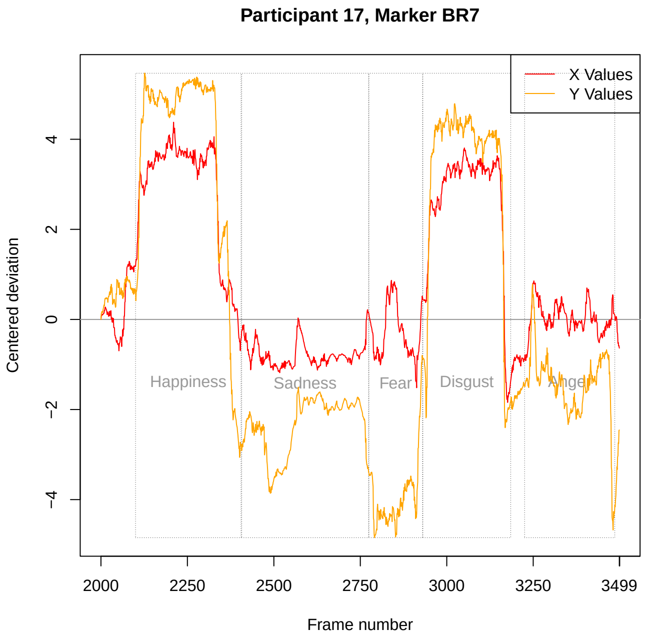
Participant 17, Marker BR7
X Values
Y Values
Happiness
Sadness
Fear
Disgust
Anger
Centered deviation
Frame number
2000
2250
2500
2750
3000
3250
3499
−4
−2
0
2
4

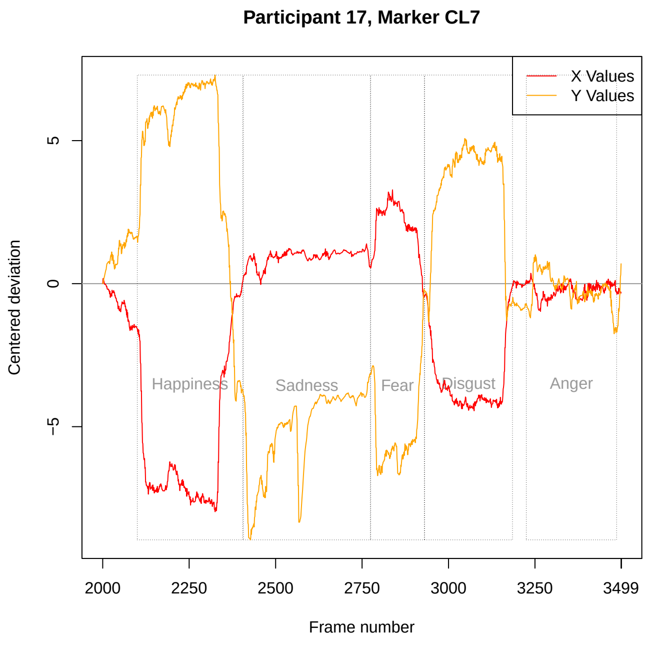
Participant 17, Marker CL7
X Values
Y Values
Happiness
Sadness
Fear
Disgust
Anger
Centered deviation
Frame number
2000
2250
2500
2750
3000
3250
3499
−5
0
5

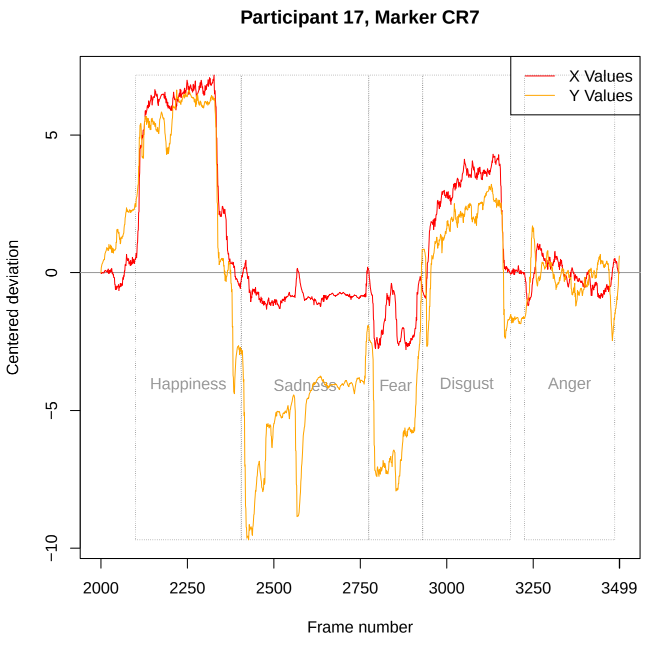
Participant 17, Marker CR7
X Values
Y Values
Happiness
Sadness
Fear
Disgust
Anger
Centered deviation
Frame number
2000
2250
2500
2750
3000
3250
3499
−10
−5
0
5

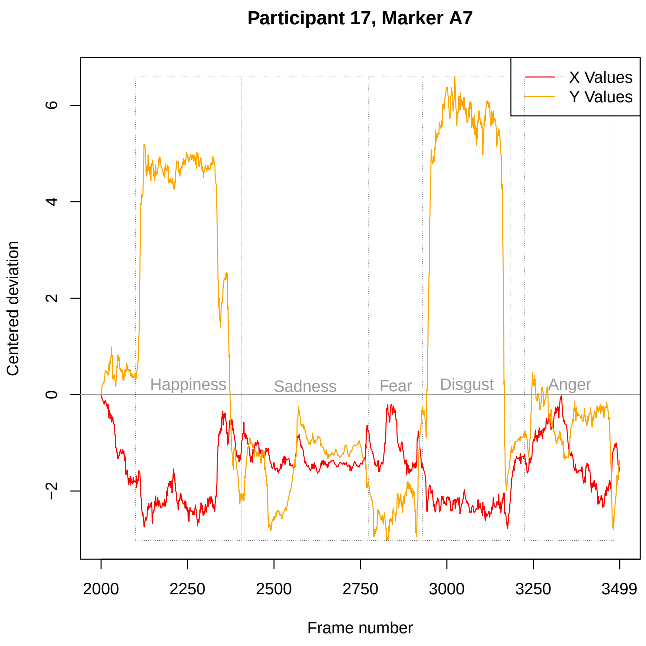
Participant 17, Marker A7
X Values
Y Values
Happiness
Sadness
Fear
Disgust
Anger
Centered deviation
−2
0
2
4
6
2000
2250
2500
2750
3000
3250
3499
Frame number

Participant 18, Marker BL2

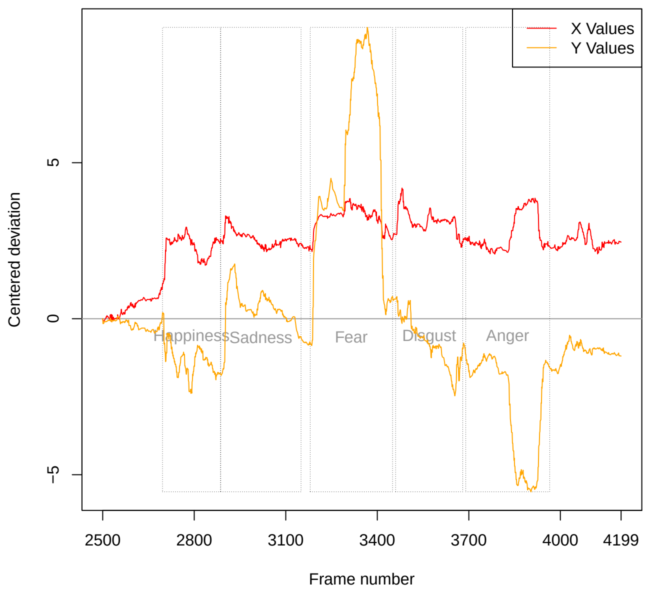

Participant 18, Marker BR2

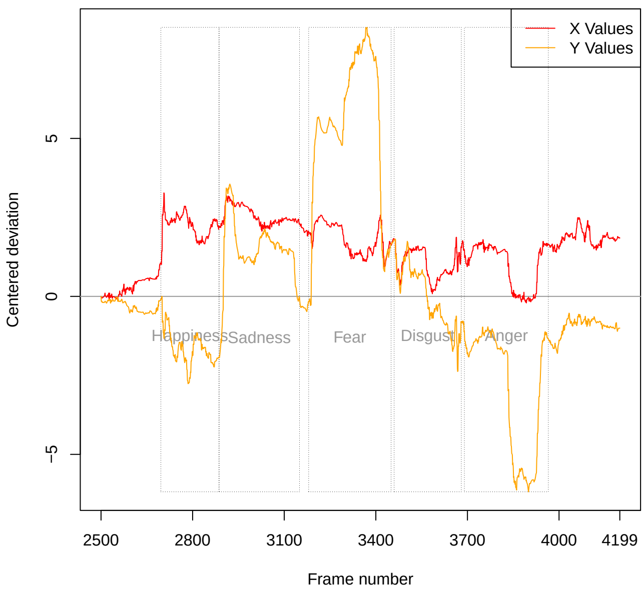

Participant 18, Marker DL3

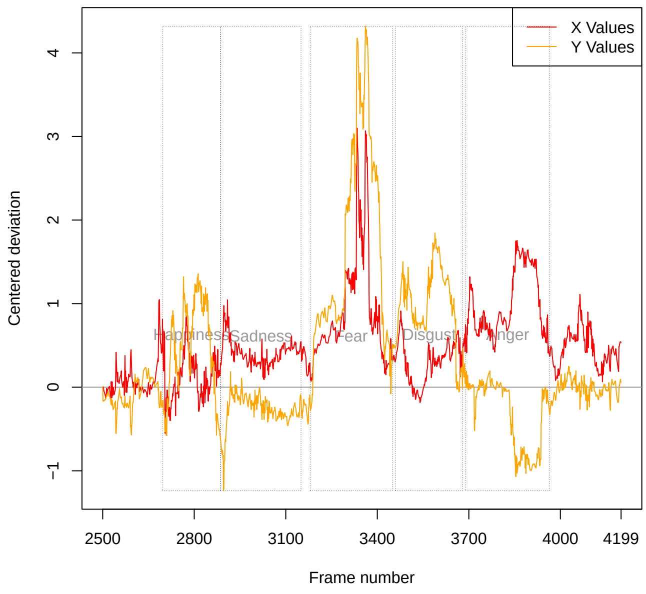

Participant 18, Marker DR3

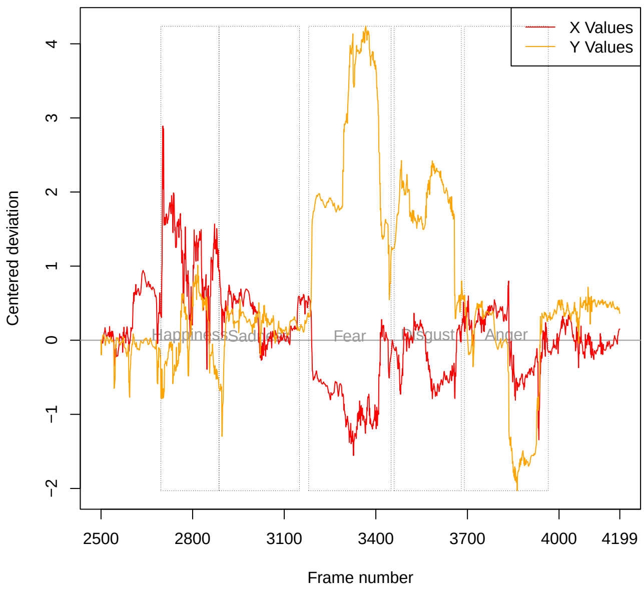

Participant 18, Marker CL4

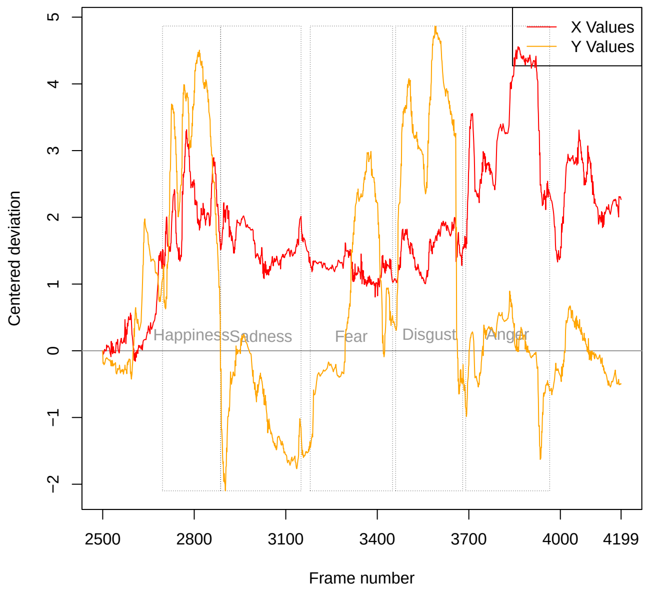

Participant 18, Marker CR4

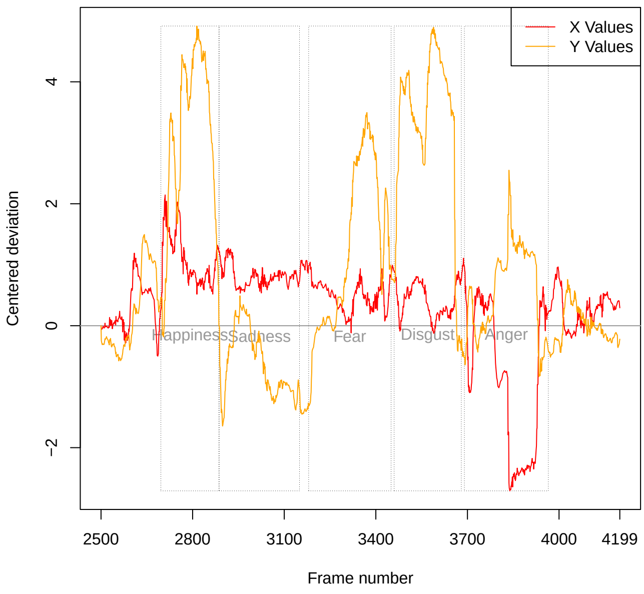

Participant 18, Marker BL5

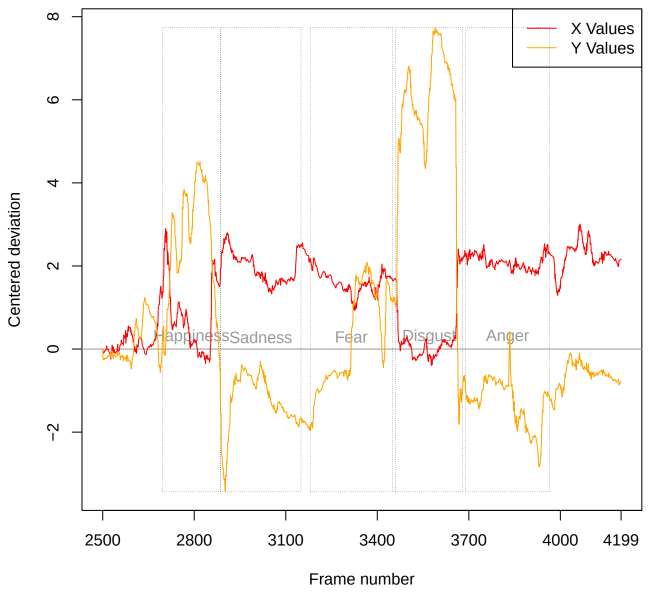

Participant 18, Marker BR5

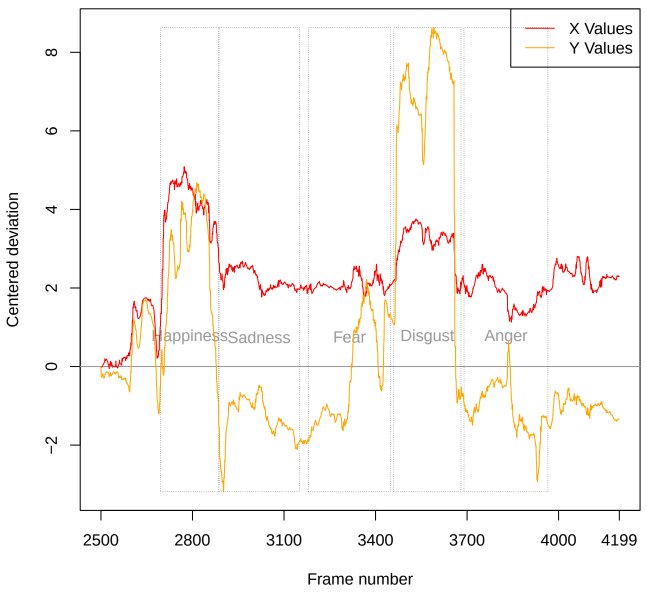

Participant 18, Marker BL7

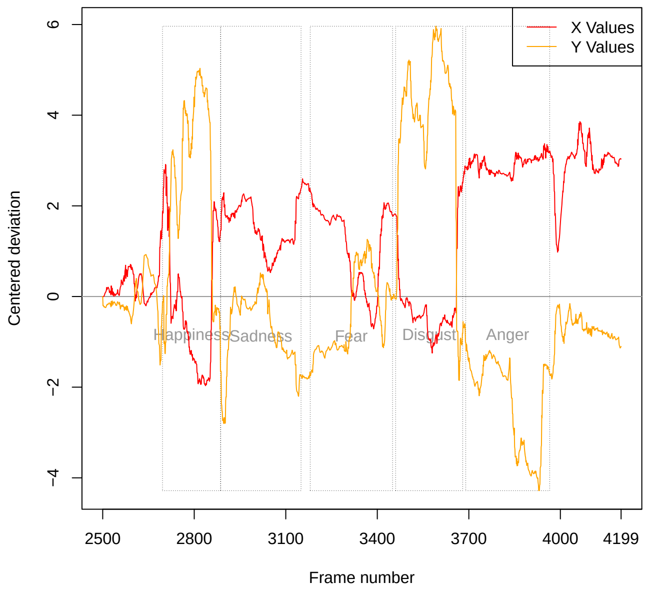

Participant 18, Marker BR7

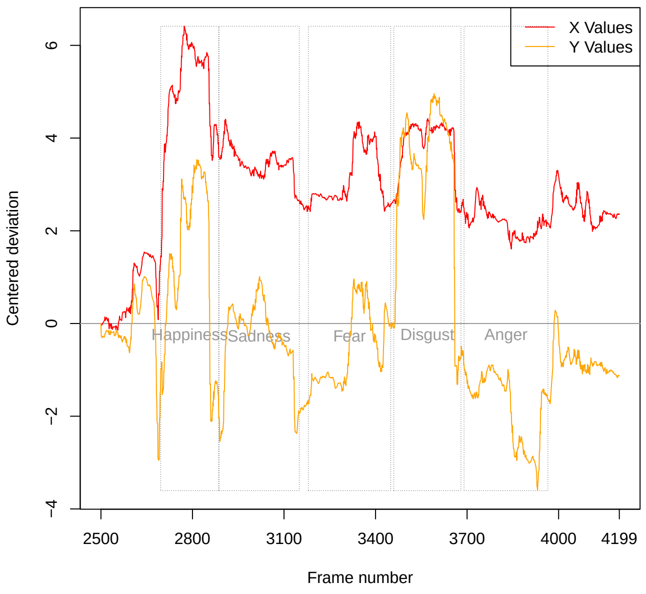

Participant 18, Marker CL7

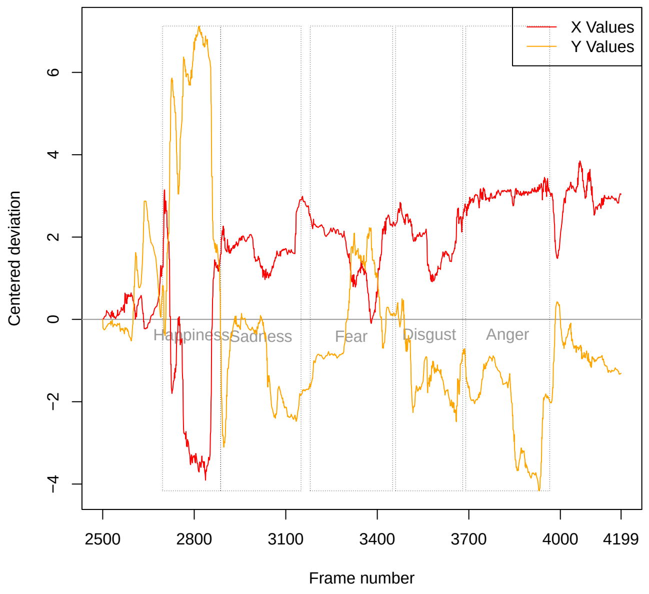

Participant 18, Marker CR7

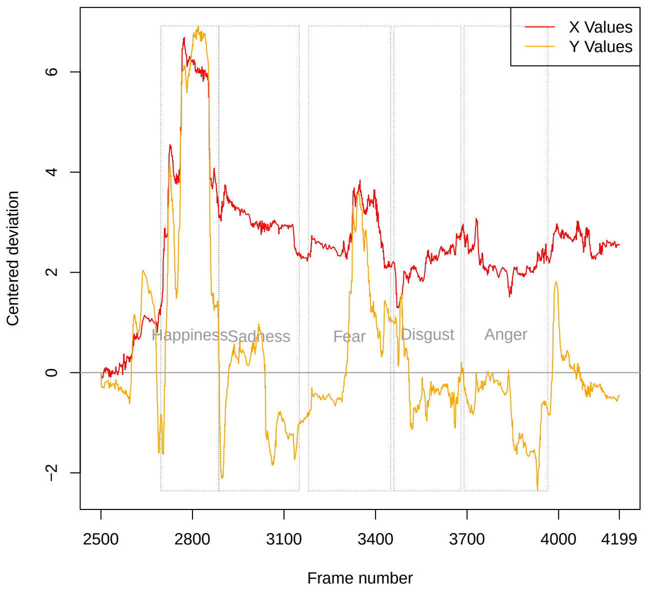

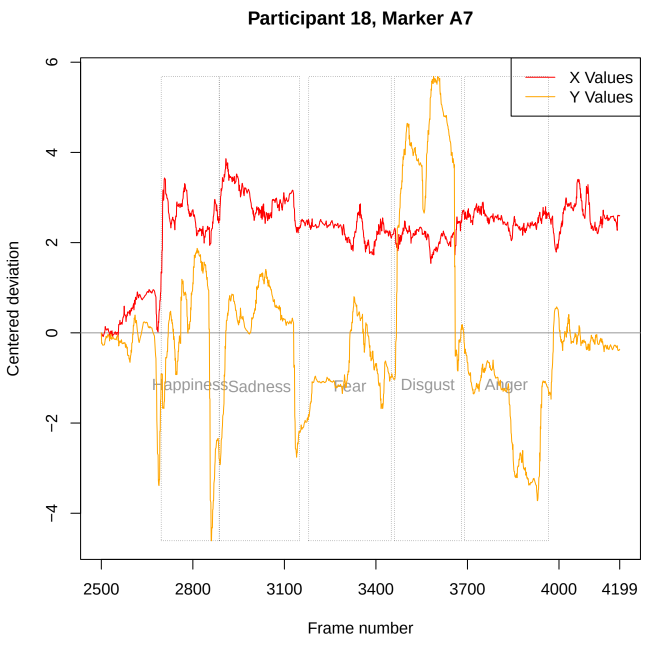

Participant 18, Marker A7
X Values
Y Values
Centered deviation
6
4
2
0
−2
−4
Happiness
Sadness
Fear
Disgust
Anger
2500
2800
3100
3400
3700
4000
4199
Frame number

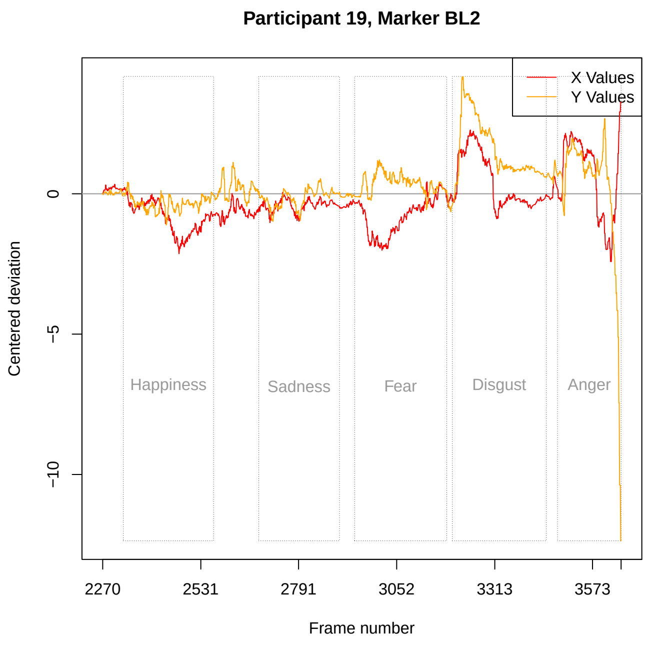
Participant 19, Marker BL2
X Values
Y Values
Happiness
Sadness
Fear
Disgust
Anger
Centered deviation
0
−5
−10
2270
2531
2791
3052
3313
3573
Frame number

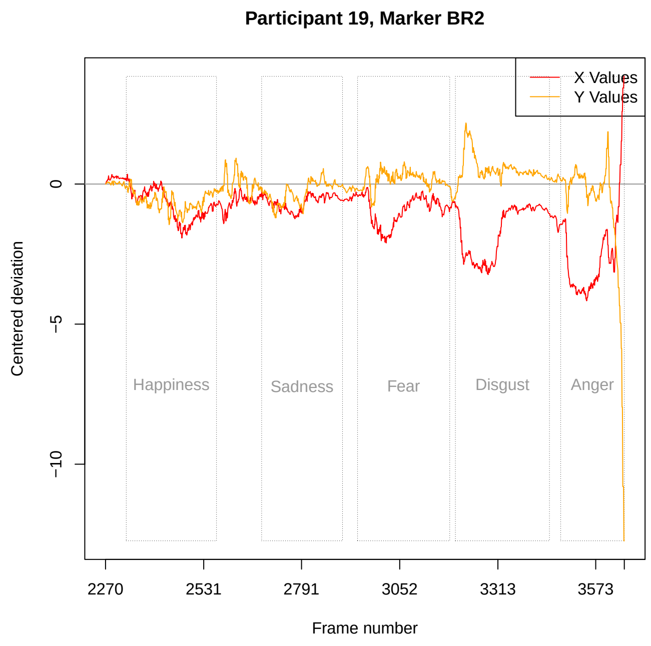
Participant 19, Marker BR2
X Values
Y Values
Happiness
Sadness
Fear
Disgust
Anger
Centered deviation
0
−5
−10
2270
2531
2791
3052
3313
3573
Frame number

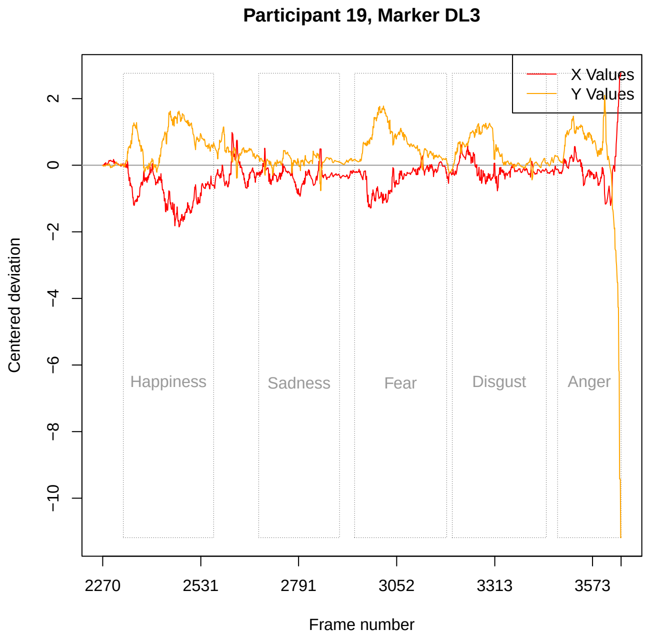
Participant 19, Marker DL3
X Values
Y Values
Happiness
Sadness
Fear
Disgust
Anger
Centered deviation
2
0
−2
−4
−6
−8
−10
2270
2531
2791
3052
3313
3573
Frame number

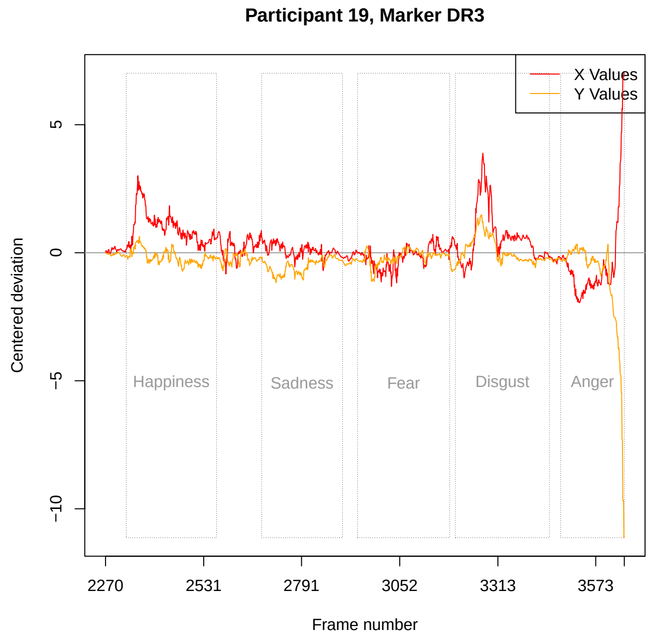
Participant 19, Marker DR3
X Values
Y Values
Happiness
Sadness
Fear
Disgust
Anger
Centered deviation
5
0
−5
−10
2270
2531
2791
3052
3313
3573
Frame number

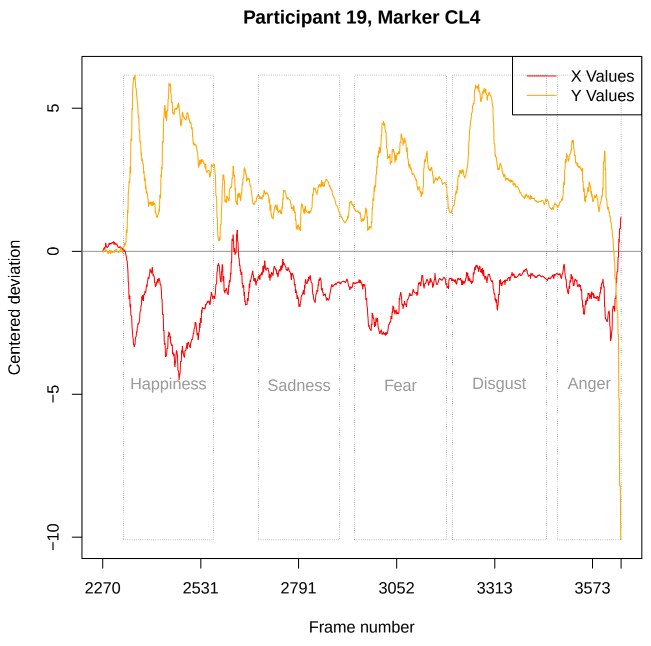
Participant 19, Marker CL4
X Values
Y Values
Happiness
Sadness
Fear
Disgust
Anger
Centered deviation
5
0
−5
−10
2270
2531
2791
3052
3313
3573
Frame number

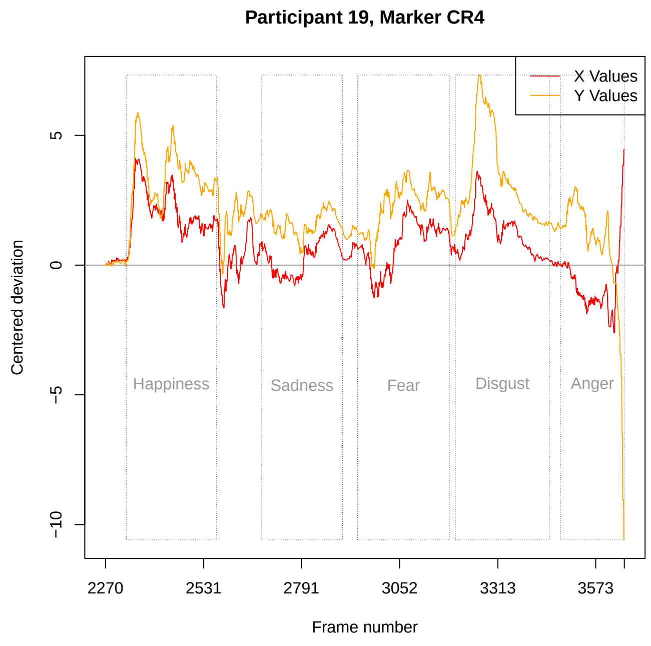
Participant 19, Marker CR4
X Values
Y Values
Happiness
Sadness
Fear
Disgust
Anger
Centered deviation
5
0
−5
−10
2270
2531
2791
3052
3313
3573
Frame number

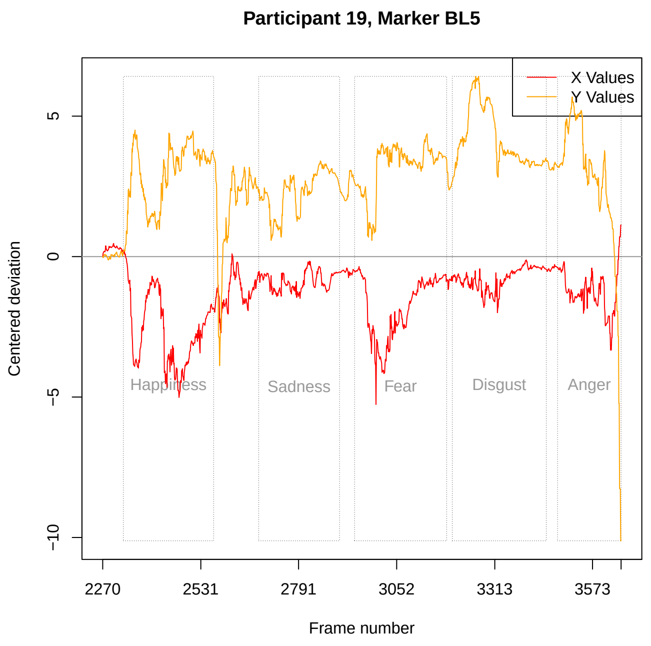
Participant 19, Marker BL5
X Values
Y Values
Centered deviation
Frame number
2270
2531
2791
3052
3313
3573
5
0
−5
−10
Happiness
Sadness
Fear
Disgust
Anger

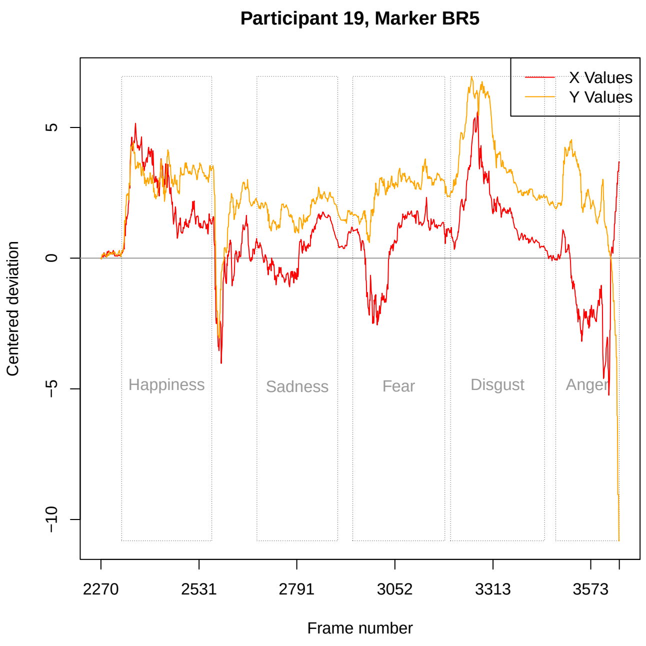
Participant 19, Marker BR5
X Values
Y Values
Centered deviation
Frame number
2270
2531
2791
3052
3313
3573
5
0
−5
−10
Happiness
Sadness
Fear
Disgust
Anger

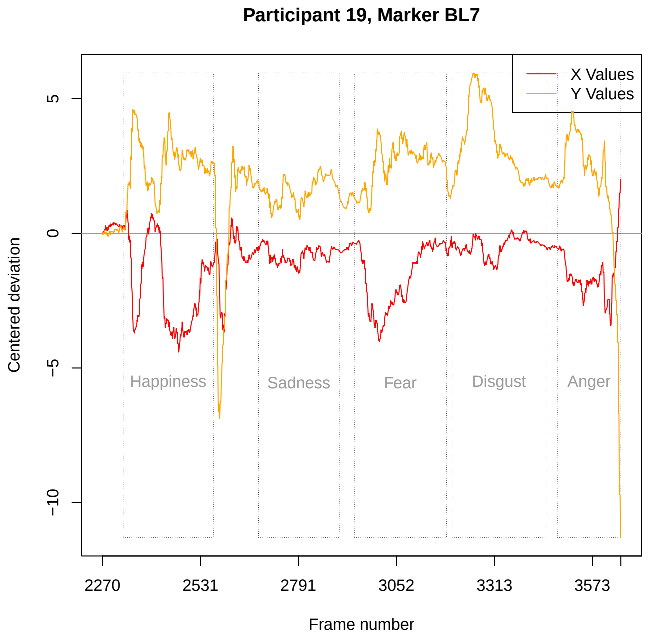
Participant 19, Marker BL7
X Values
Y Values
Centered deviation
Frame number
2270
2531
2791
3052
3313
3573
5
0
−5
−10
Happiness
Sadness
Fear
Disgust
Anger

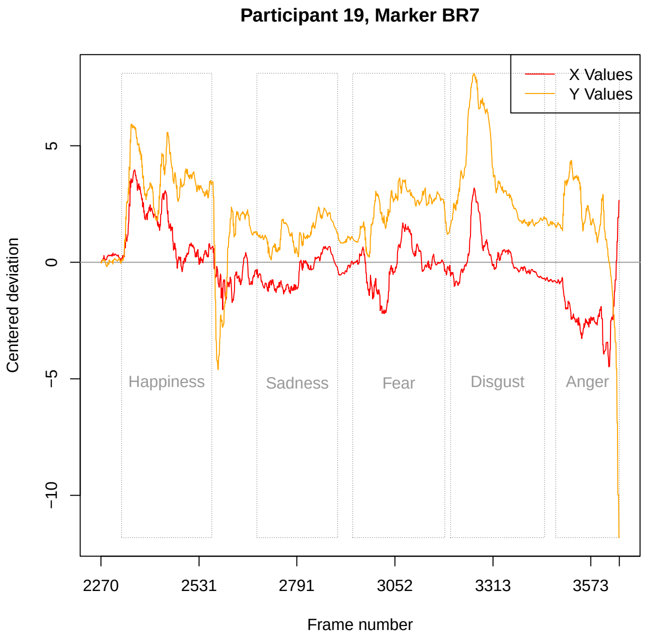
Participant 19, Marker BR7
X Values
Y Values
Centered deviation
Frame number
2270
2531
2791
3052
3313
3573
5
0
−5
−10
Happiness
Sadness
Fear
Disgust
Anger

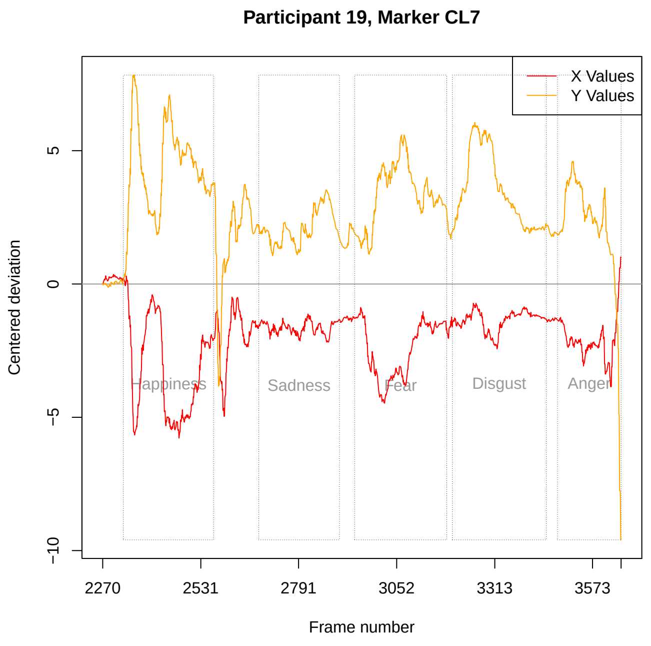
Participant 19, Marker CL7
X Values
Y Values
Centered deviation
Frame number
2270
2531
2791
3052
3313
3573
5
0
−5
−10
Happiness
Sadness
Fear
Disgust
Anger

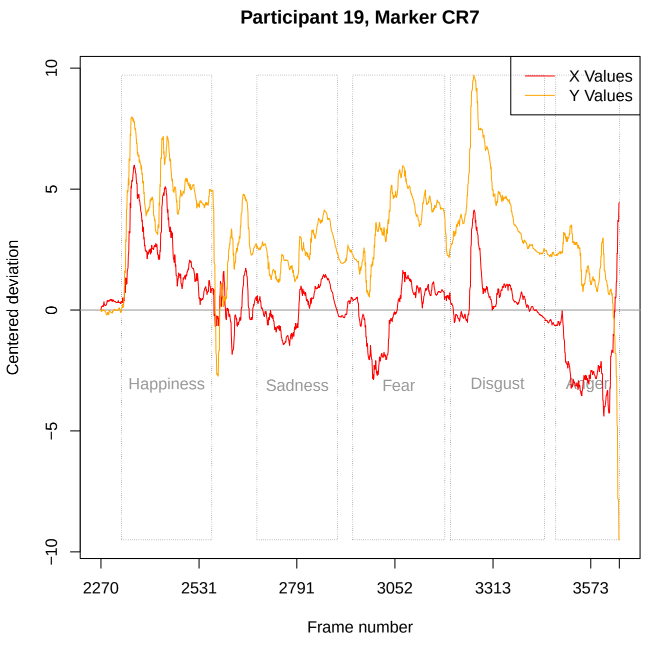
Participant 19, Marker CR7
X Values
Y Values
Centered deviation
Frame number
2270
2531
2791
3052
3313
3573
10
5
0
−5
−10
Happiness
Sadness
Fear
Disgust
Anger

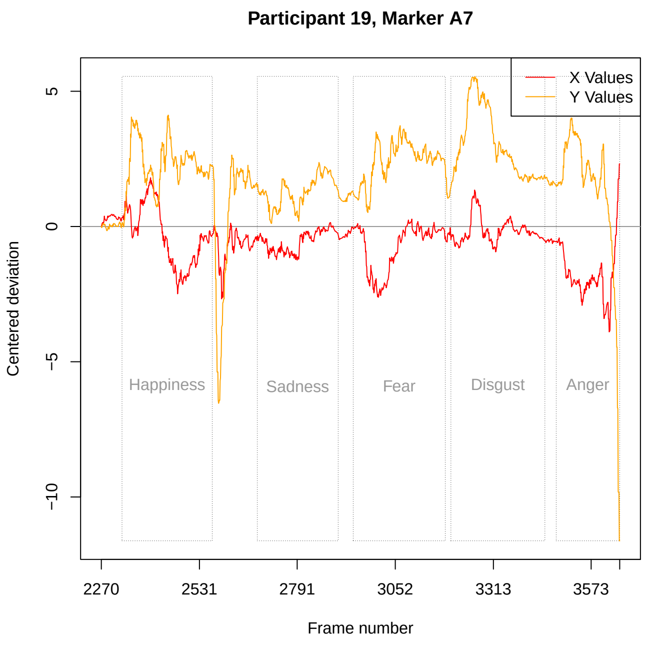
Participant 19, Marker A7
X Values
Y Values
5
0
−5
−10
Centered deviation
Happiness
Sadness
Fear
Disgust
Anger
2270
2531
2791
3052
3313
3573
Frame number

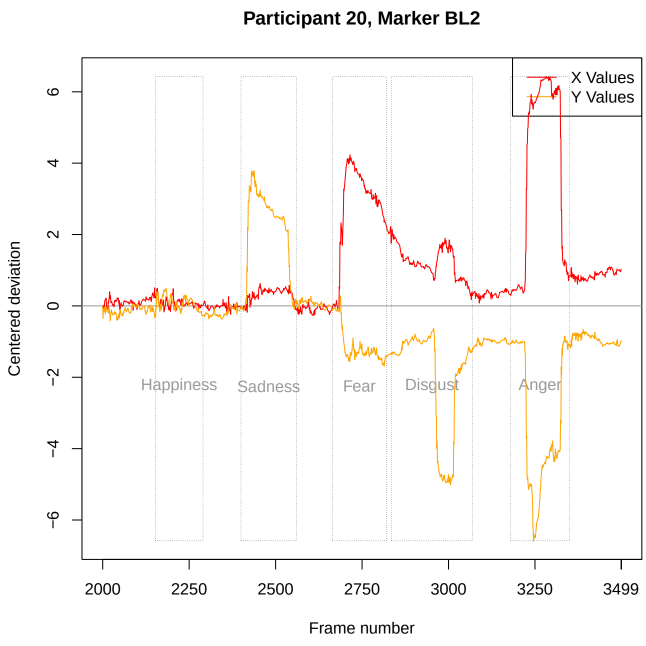
Participant 20, Marker BL2
X Values
Y Values
Happiness
Sadness
Fear
Disgust
Anger
Centered deviation
Frame number

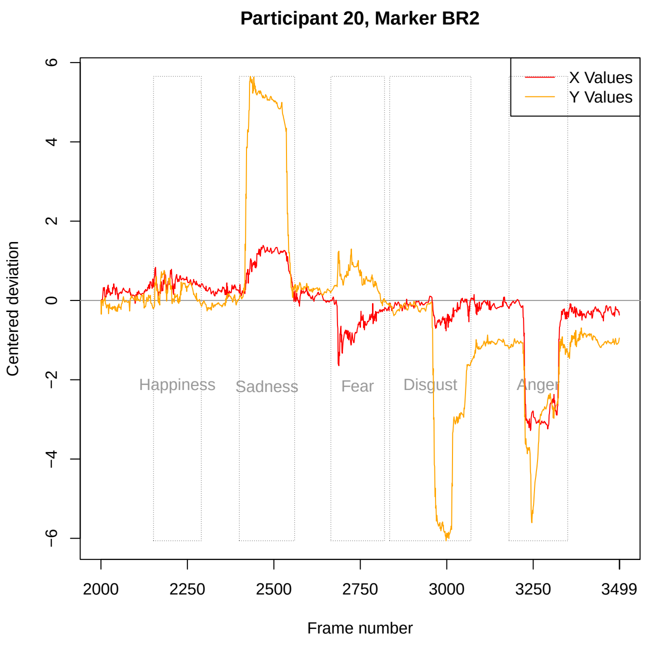
Participant 20, Marker BR2
X Values
Y Values
Happiness
Sadness
Fear
Disgust
Anger
Centered deviation
Frame number

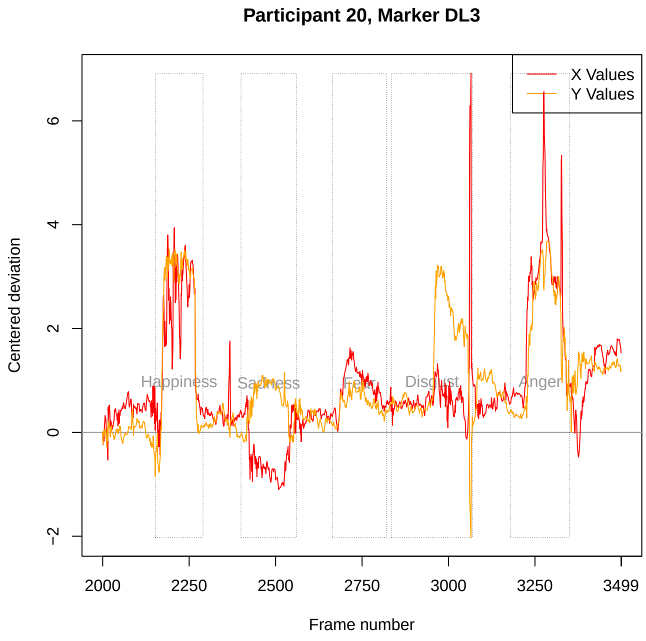
Participant 20, Marker DL3
X Values
Y Values
Happiness
Sadness
Fear
Disgust
Anger
Centered deviation
Frame number

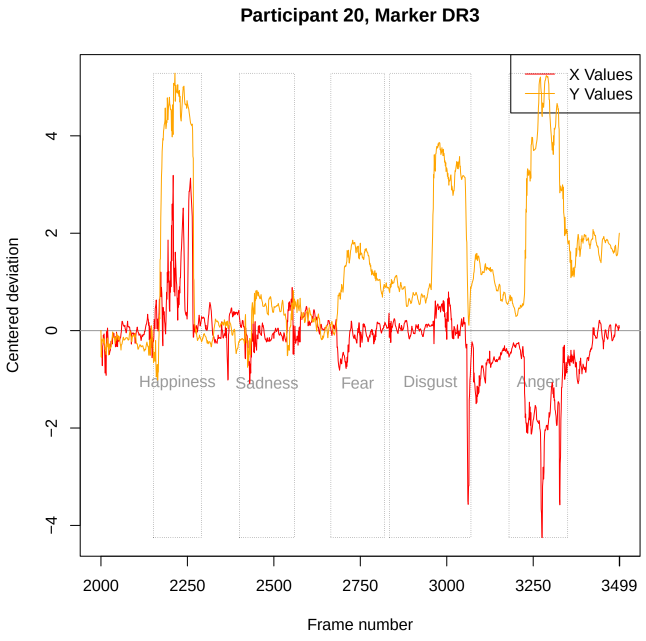
Participant 20, Marker DR3
X Values
Y Values
Happiness
Sadness
Fear
Disgust
Anger
Centered deviation
Frame number

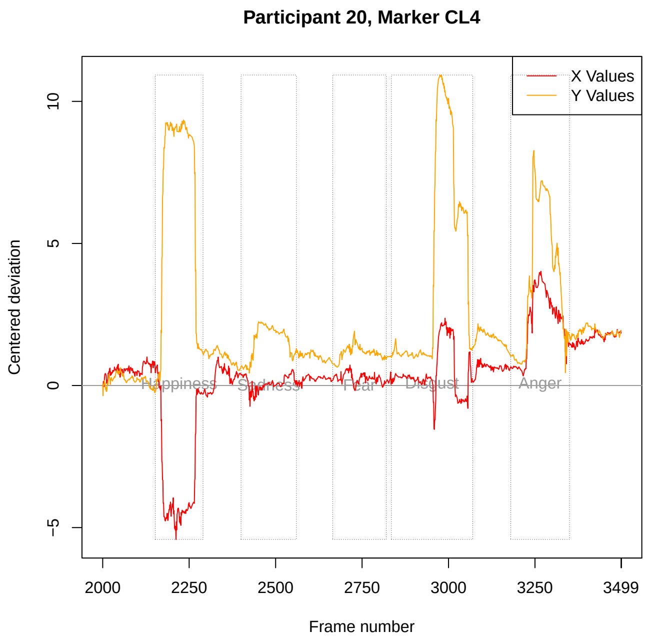
Participant 20, Marker CL4
X Values
Y Values
Happiness
Sadness
Fear
Disgust
Anger
Centered deviation
Frame number

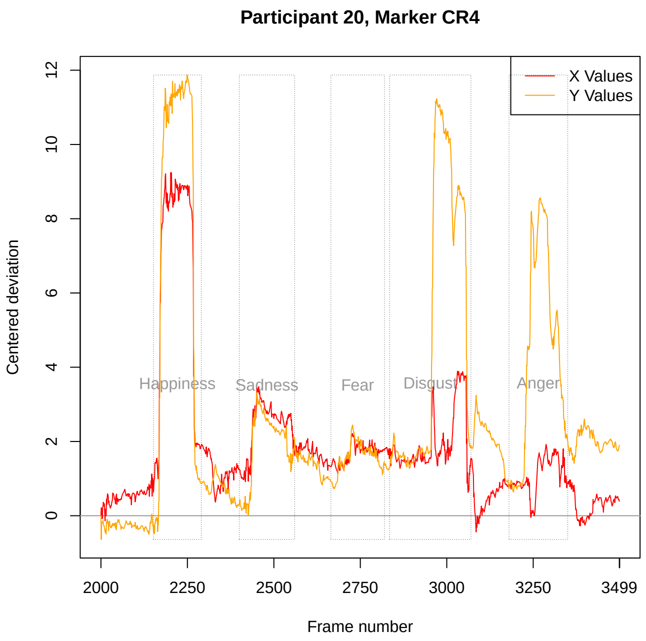
Participant 20, Marker CR4
X Values
Y Values
Happiness
Sadness
Fear
Disgust
Anger
Centered deviation
Frame number

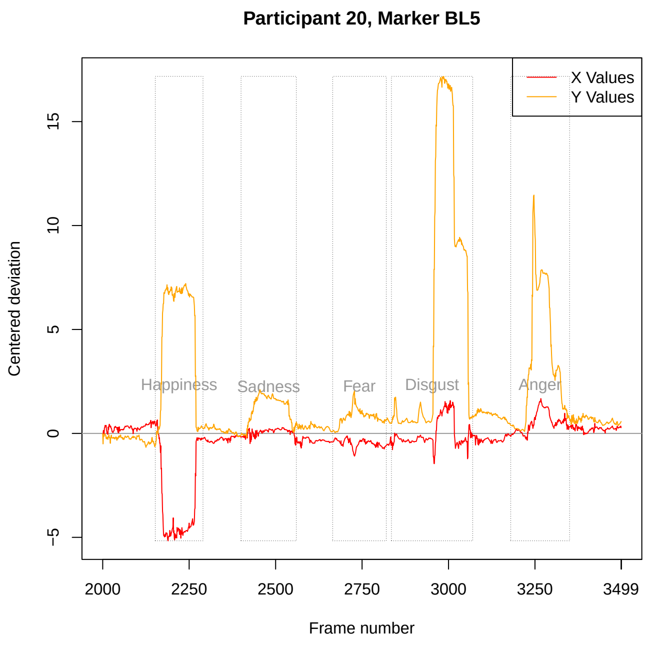
Participant 20, Marker BL5
X Values
Y Values
Happiness
Sadness
Fear
Disgust
Anger
Centered deviation
Frame number
2000
2250
2500
2750
3000
3250
3499

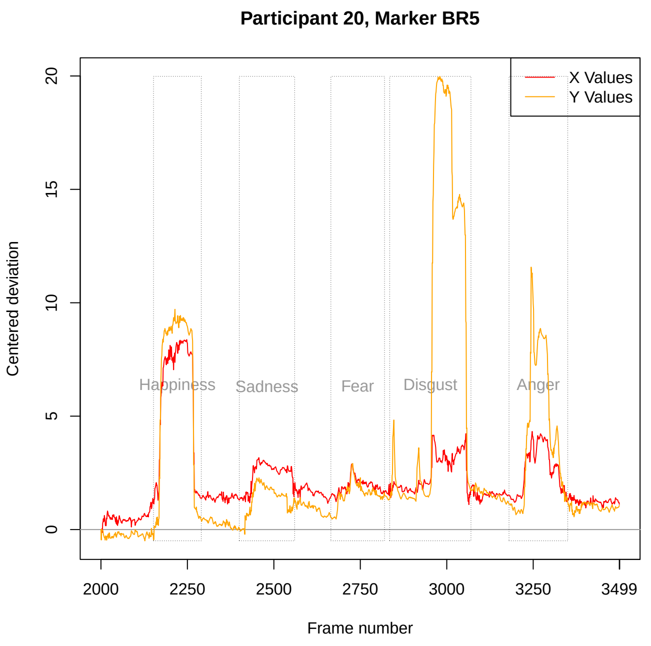
Participant 20, Marker BR5
X Values
Y Values
Happiness
Sadness
Fear
Disgust
Anger
Centered deviation
Frame number

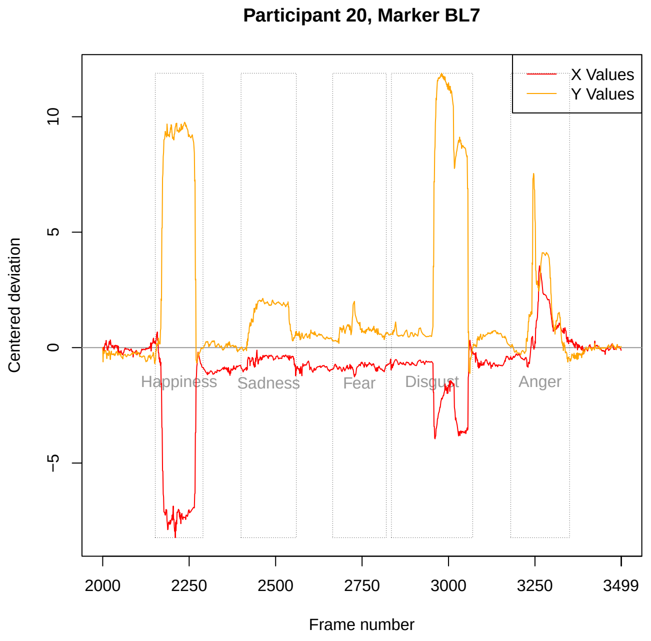
Participant 20, Marker BL7
X Values
Y Values
Happiness
Sadness
Fear
Disgust
Anger
Centered deviation
Frame number

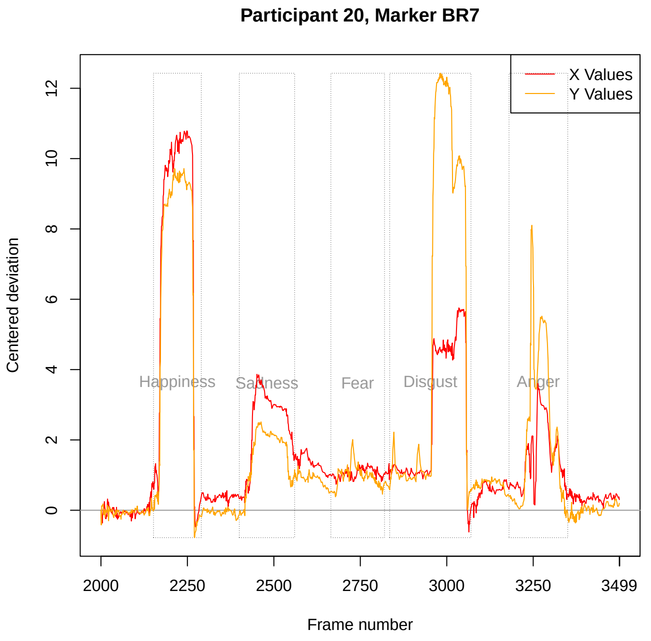
Participant 20, Marker BR7
X Values
Y Values
Happiness
Sadness
Fear
Disgust
Anger
Centered deviation
Frame number

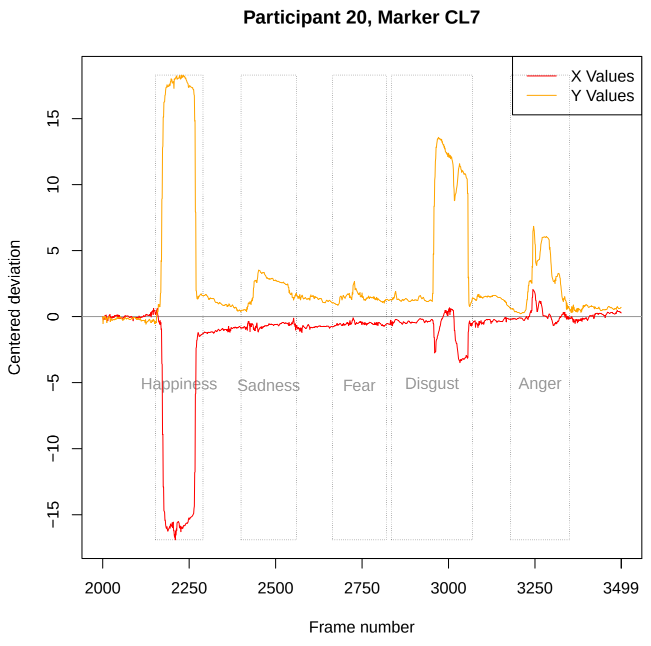
Participant 20, Marker CL7
X Values
Y Values
Happiness
Sadness
Fear
Disgust
Anger
Centered deviation
Frame number

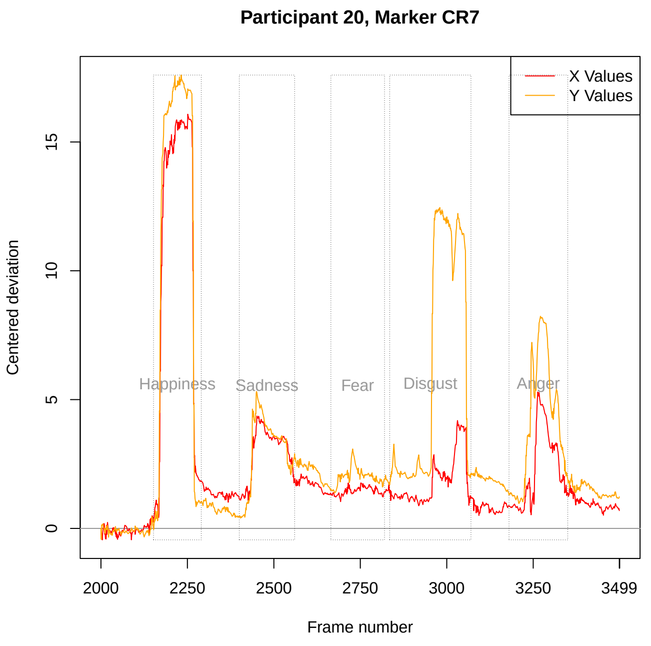
Participant 20, Marker CR7
X Values
Y Values
Happiness
Sadness
Fear
Disgust
Anger
Centered deviation
Frame number

Participant 20, Marker A7

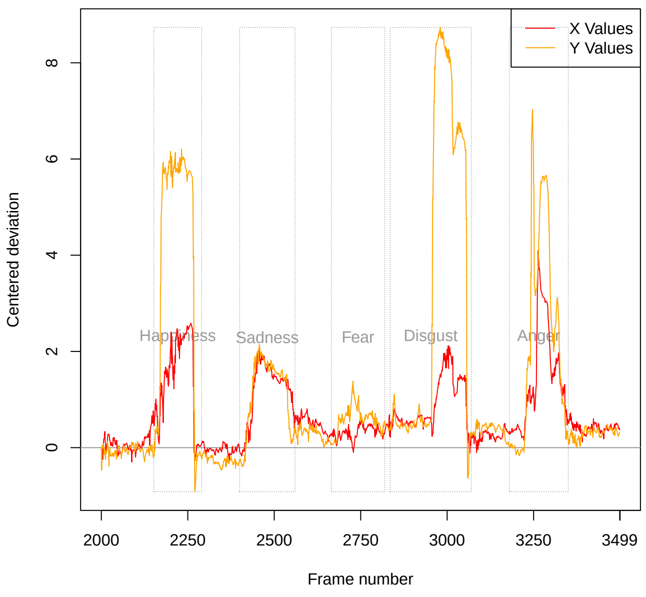

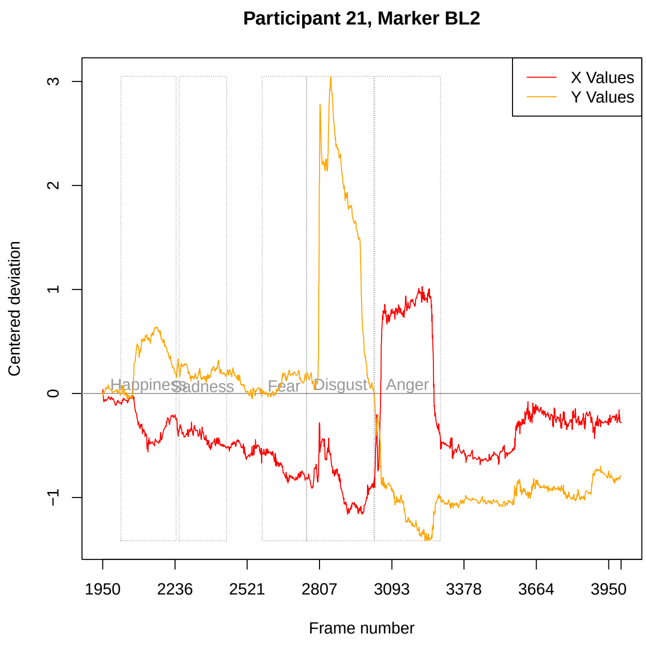
Participant 21, Marker BL2
X Values
Y Values
Happiness
Sadness
Fear
Disgust
Anger
Centered deviation
−1
0
1
2
3
1950
2236
2521
2807
3093
3378
3664
3950
Frame number

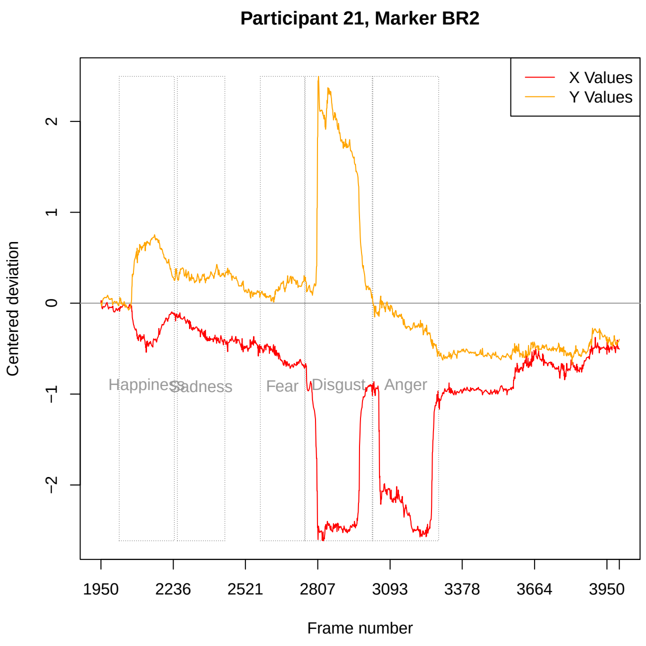
Participant 21, Marker BR2
X Values
Y Values
Happiness
Sadness
Fear
Disgust
Anger
Centered deviation
−2
−1
0
1
2
1950
2236
2521
2807
3093
3378
3664
3950
Frame number

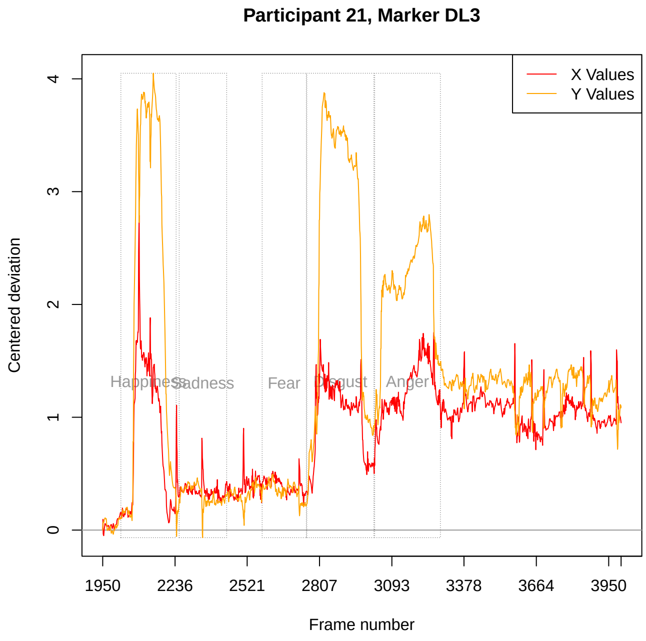
Participant 21, Marker DL3
X Values
Y Values
Happiness
Sadness
Fear
Disgust
Anger
Centered deviation
0
1
2
3
4
1950
2236
2521
2807
3093
3378
3664
3950
Frame number

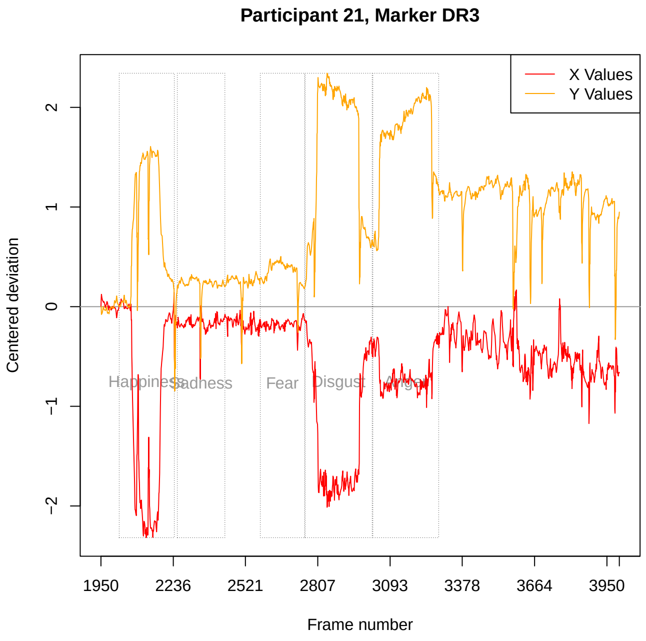
Participant 21, Marker DR3
X Values
Y Values
Happiness
Sadness
Fear
Disgust
Anger
Centered deviation
−2
−1
0
1
2
1950
2236
2521
2807
3093
3378
3664
3950
Frame number

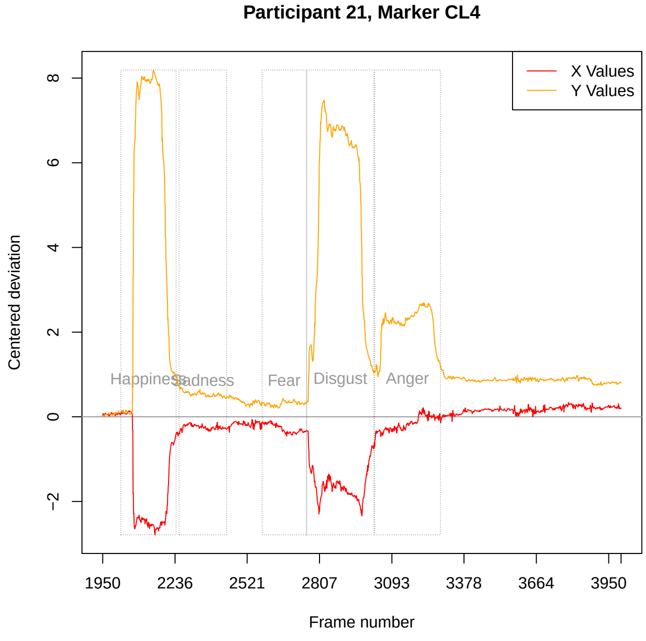
Participant 21, Marker CL4
X Values
Y Values
Happiness
Sadness
Fear
Disgust
Anger
Centered deviation
−2
0
2
4
6
8
1950
2236
2521
2807
3093
3378
3664
3950
Frame number

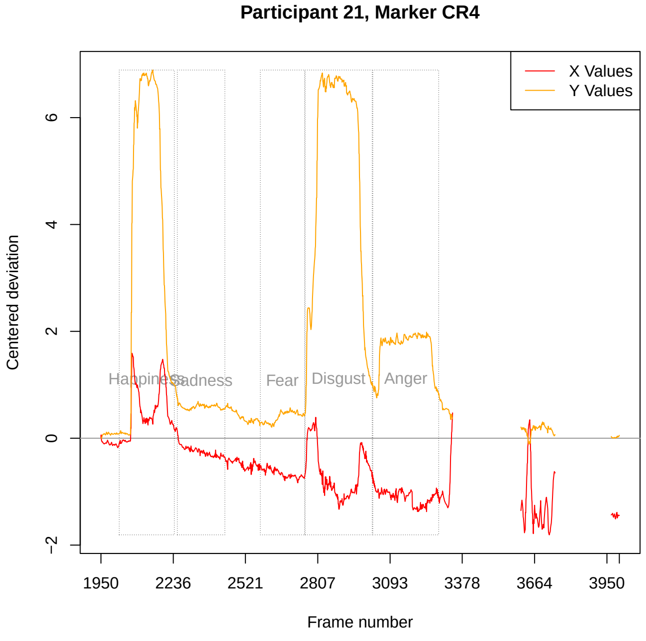
Participant 21, Marker CR4
X Values
Y Values
Happiness
Sadness
Fear
Disgust
Anger
Centered deviation
−2
0
2
4
6
1950
2236
2521
2807
3093
3378
3664
3950
Frame number

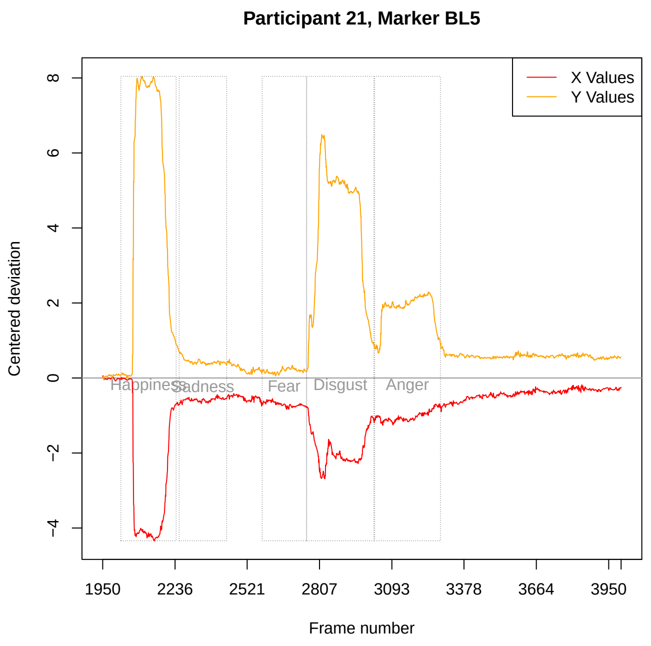
Participant 21, Marker BL5
Centered deviation
Frame number
X Values
Y Values
Happiness
Sadness
Fear
Disgust
Anger
1950 2236 2521 2807 3093 3378 3664 3950

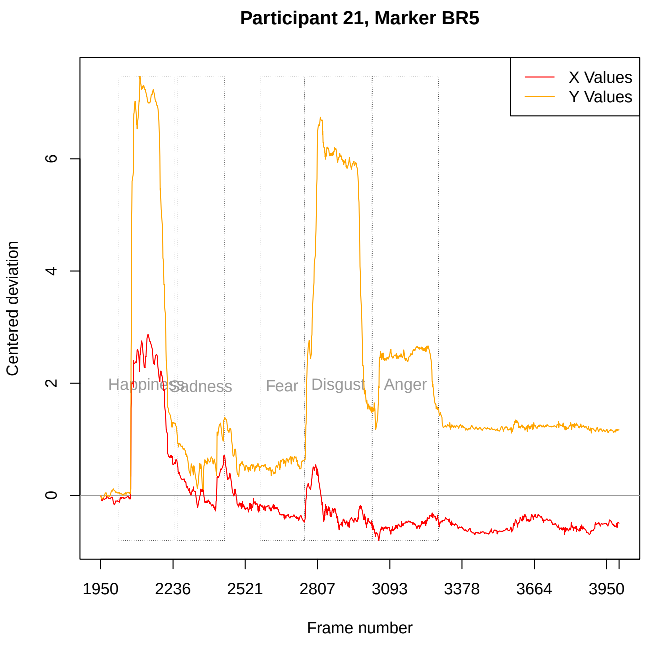
Participant 21, Marker BR5
Centered deviation
Frame number
X Values
Y Values
Happiness
Sadness
Fear
Disgust
Anger
1950 2236 2521 2807 3093 3378 3664 3950

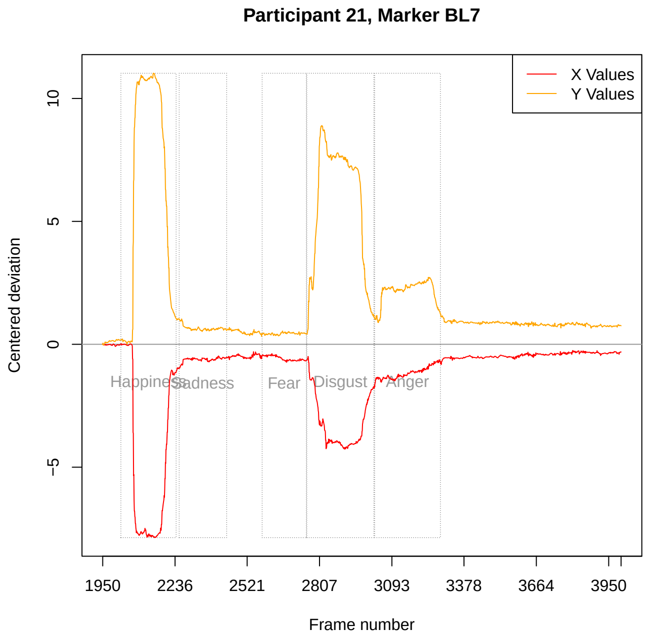
Participant 21, Marker BL7
Centered deviation
Frame number
X Values
Y Values
Happiness
Sadness
Fear
Disgust
Anger
1950 2236 2521 2807 3093 3378 3664 3950

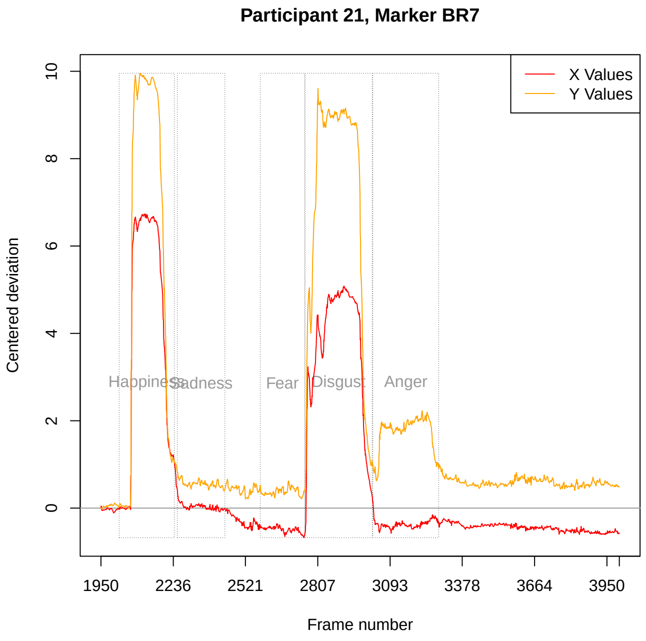
Participant 21, Marker BR7
Centered deviation
Frame number
X Values
Y Values
Happiness
Sadness
Fear
Disgust
Anger
1950 2236 2521 2807 3093 3378 3664 3950

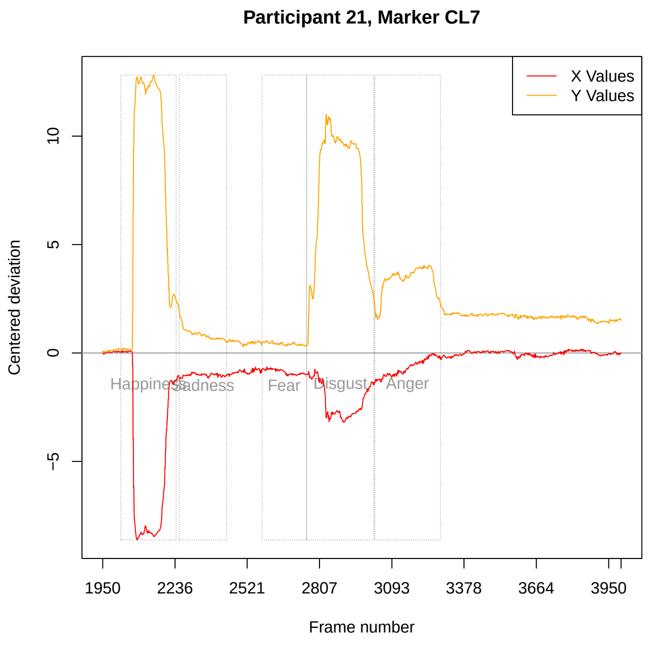
Participant 21, Marker CL7
Centered deviation
Frame number
X Values
Y Values
Happiness
Sadness
Fear
Disgust
Anger
1950 2236 2521 2807 3093 3378 3664 3950

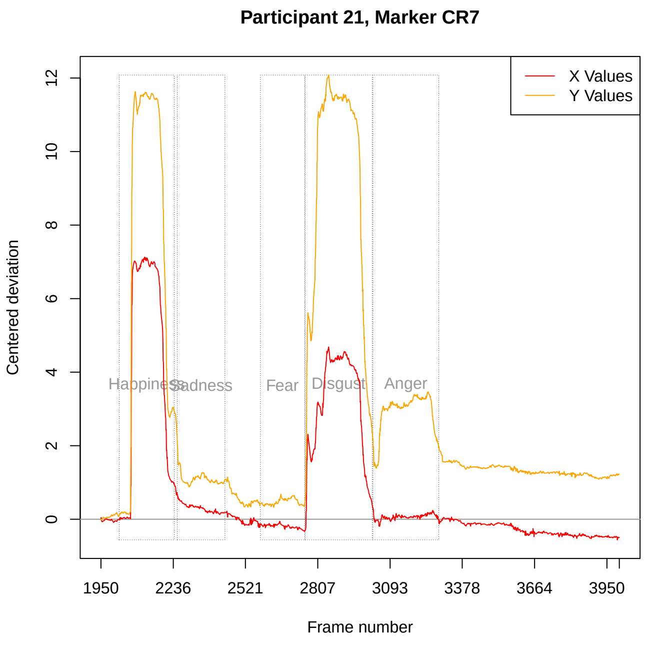
Participant 21, Marker CR7
Centered deviation
Frame number
X Values
Y Values
Happiness
Sadness
Fear
Disgust
Anger
1950 2236 2521 2807 3093 3378 3664 3950

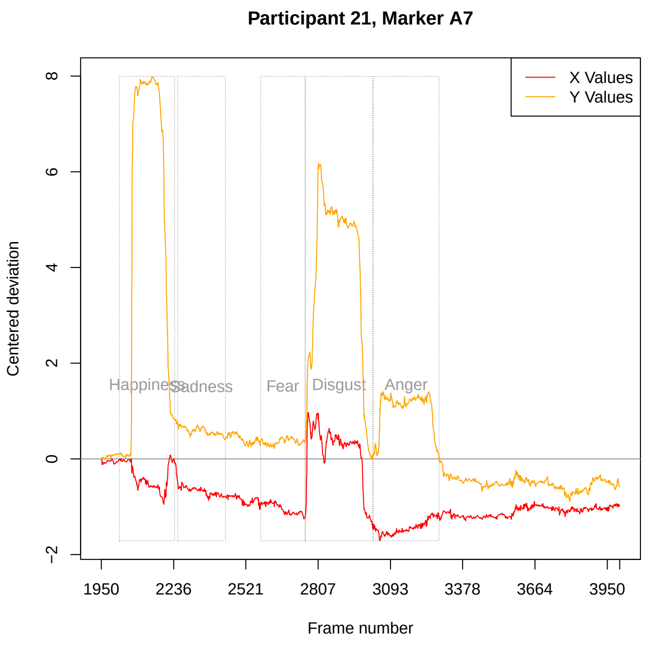

Participant 21, Marker A7
X Values
Y Values
Happiness
Sadness
Fear
Disgust
Anger
Centered deviation
−2
0
2
4
6
8
1950
2236
2521
2807
3093
3378
3664
3950
Frame number

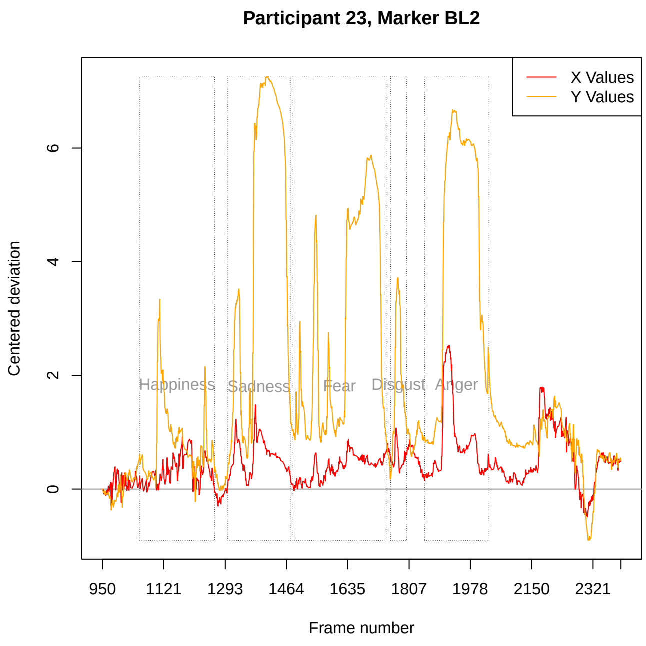
Participant 23, Marker BL2
X Values
Y Values
Happiness
Sadness
Fear
Disgust
Anger
Centered deviation
Frame number
950 1121 1293 1464 1635 1807 1978 2150 2321
0 2 4 6

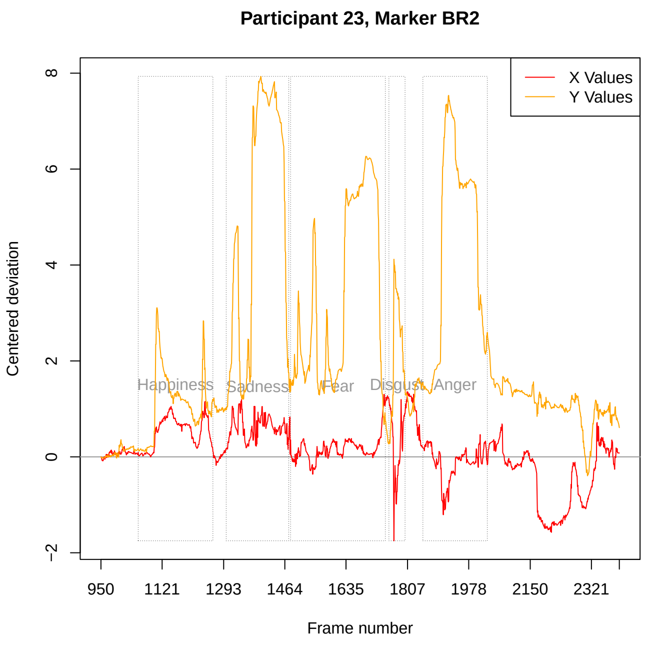
Participant 23, Marker BR2
X Values
Y Values
Happiness
Sadness
Fear
Disgust
Anger
Centered deviation
Frame number
950 1121 1293 1464 1635 1807 1978 2150 2321
−2 0 2 4 6 8

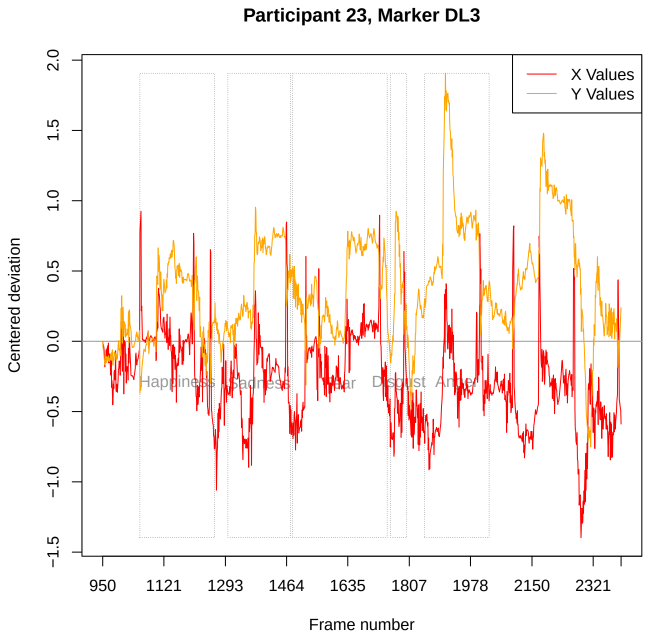
Participant 23, Marker DL3
X Values
Y Values
Happiness
Sadness
Fear
Disgust
Anger
Centered deviation
Frame number
950 1121 1293 1464 1635 1807 1978 2150 2321
−1.5 −1.0 −0.5 0.0 0.5 1.0 1.5 2.0

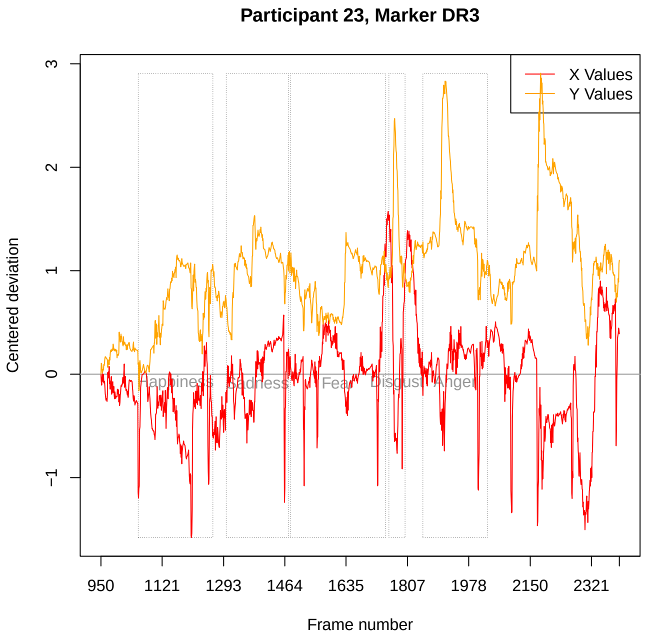
Participant 23, Marker DR3
X Values
Y Values
Happiness
Sadness
Fear
Disgust
Anger
Centered deviation
Frame number
950 1121 1293 1464 1635 1807 1978 2150 2321
−1 0 1 2 3

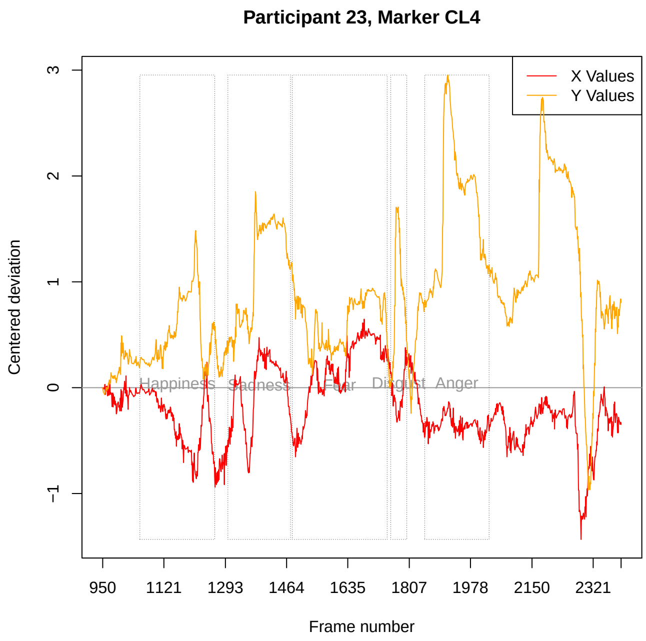
Participant 23, Marker CL4
X Values
Y Values
Happiness
Sadness
Fear
Disgust
Anger
Centered deviation
Frame number
950 1121 1293 1464 1635 1807 1978 2150 2321
−1 0 1 2 3

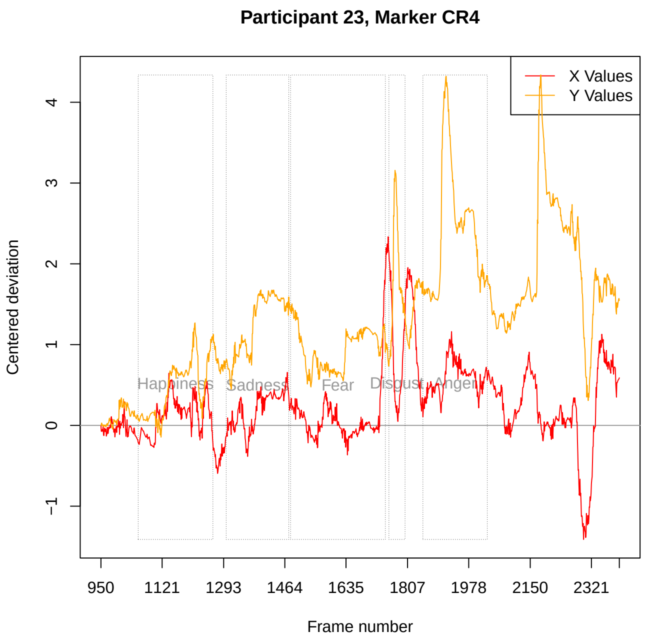
Participant 23, Marker CR4
X Values
Y Values
Happiness
Sadness
Fear
Disgust
Anger
Centered deviation
Frame number
950 1121 1293 1464 1635 1807 1978 2150 2321
−1 0 1 2 3 4

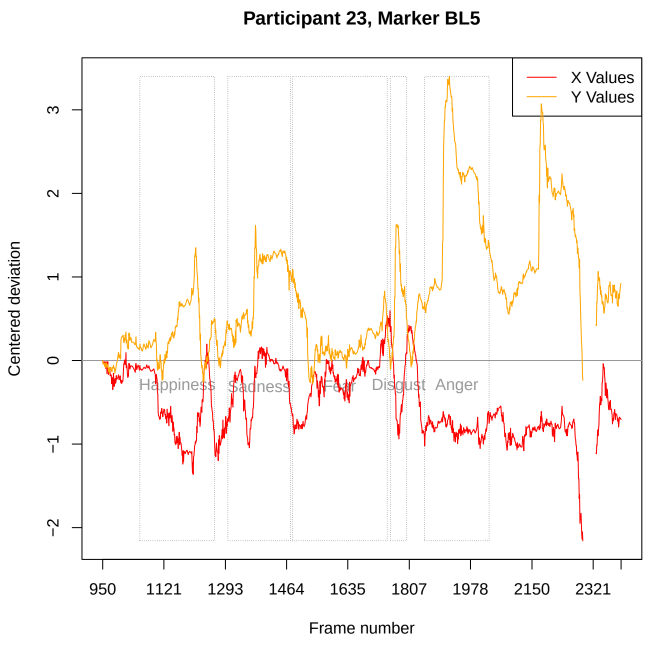
Participant 23, Marker BL5
X Values
Y Values
Happiness
Sadness
Fear
Disgust
Anger
Centered deviation
Frame number
950
1121
1293
1464
1635
1807
1978
2150
2321

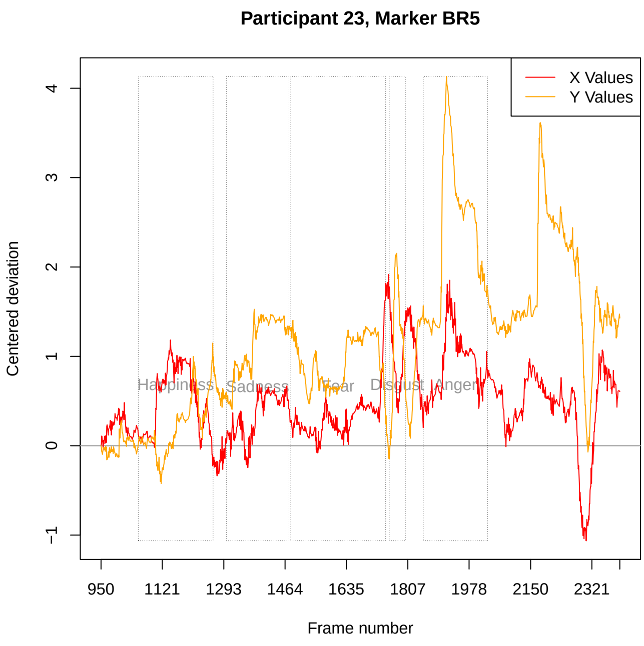
Participant 23, Marker BR5
X Values
Y Values
Happiness
Sadness
Fear
Disgust
Anger
Centered deviation
Frame number
950
1121
1293
1464
1635
1807
1978
2150
2321

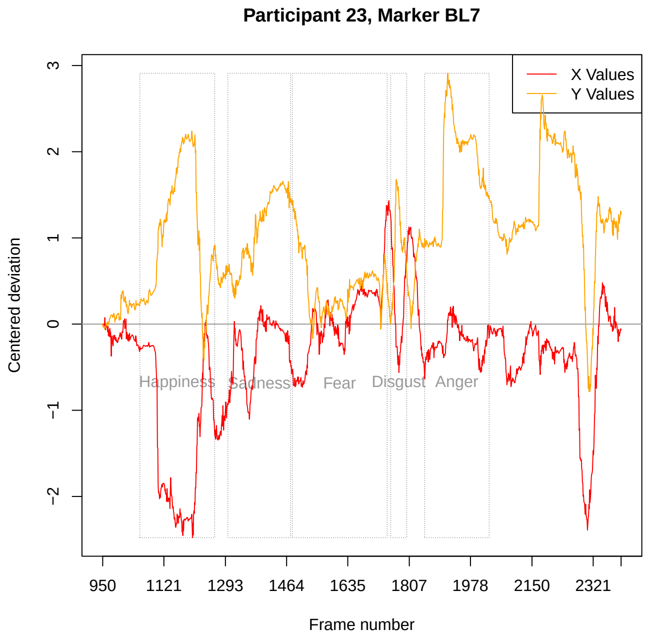
Participant 23, Marker BL7
X Values
Y Values
Happiness
Sadness
Fear
Disgust
Anger
Centered deviation
Frame number
950
1121
1293
1464
1635
1807
1978
2150
2321

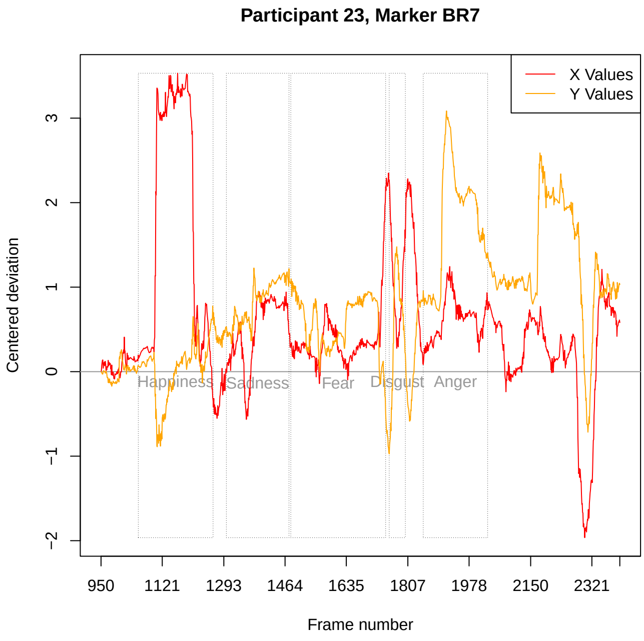
Participant 23, Marker BR7
X Values
Y Values
Happiness
Sadness
Fear
Disgust
Anger
Centered deviation
Frame number
950
1121
1293
1464
1635
1807
1978
2150
2321

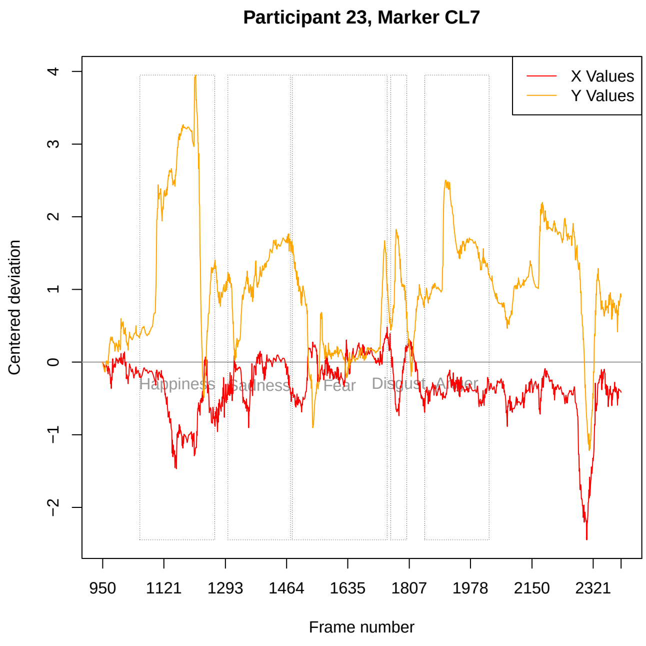
Participant 23, Marker CL7
X Values
Y Values
Happiness
Sadness
Fear
Disgust
Anger
Centered deviation
Frame number
950
1121
1293
1464
1635
1807
1978
2150
2321

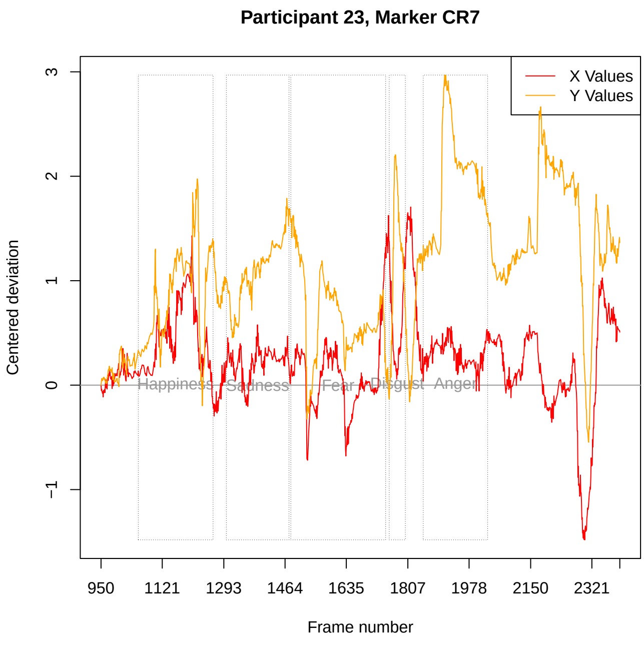
Participant 23, Marker CR7
X Values
Y Values
Happiness
Sadness
Fear
Disgust
Anger
Centered deviation
Frame number
950
1121
1293
1464
1635
1807
1978
2150
2321

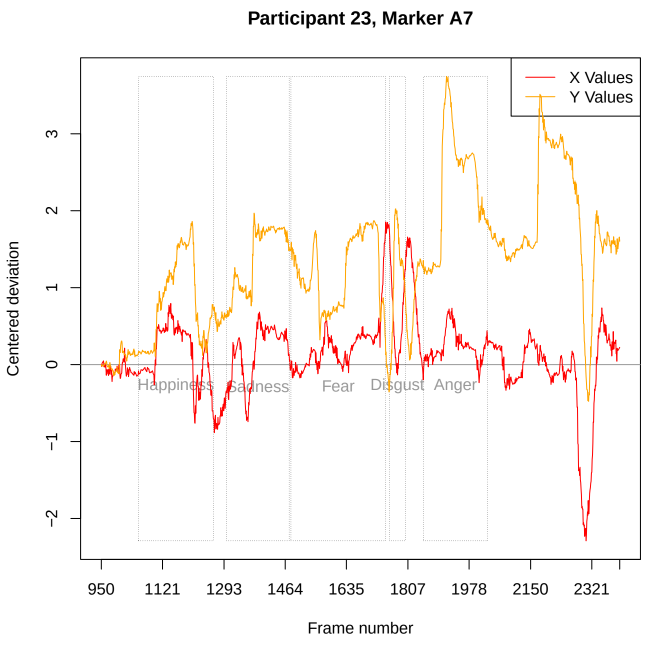

Participant 23, Marker A7
X Values
Y Values
Centered deviation
3
2
1
0
−1
−2
Happiness
Sadness
Fear
Disgust
Anger
950
1121
1293
1464
1635
1807
1978
2150
2321
Frame number

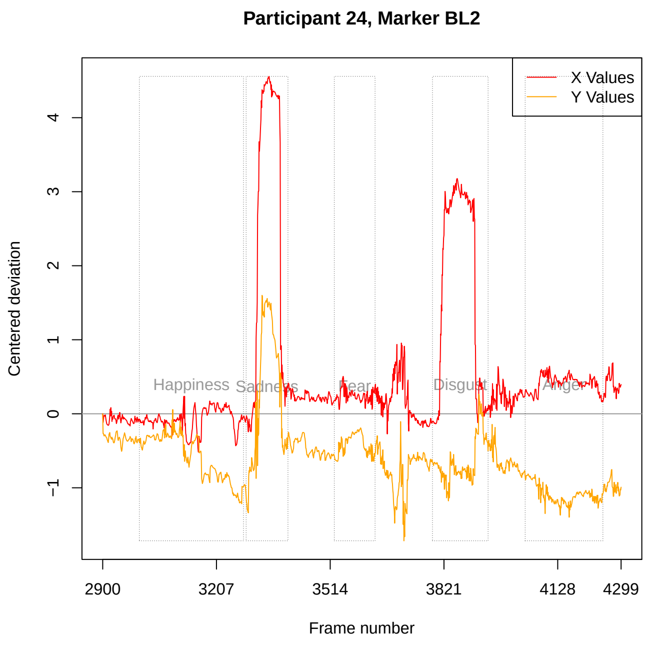
Participant 24, Marker BL2
X Values
Y Values
Happiness
Sadness
Fear
Disgust
Anger
Centered deviation
Frame number
2900
3207
3514
3821
4128
4299

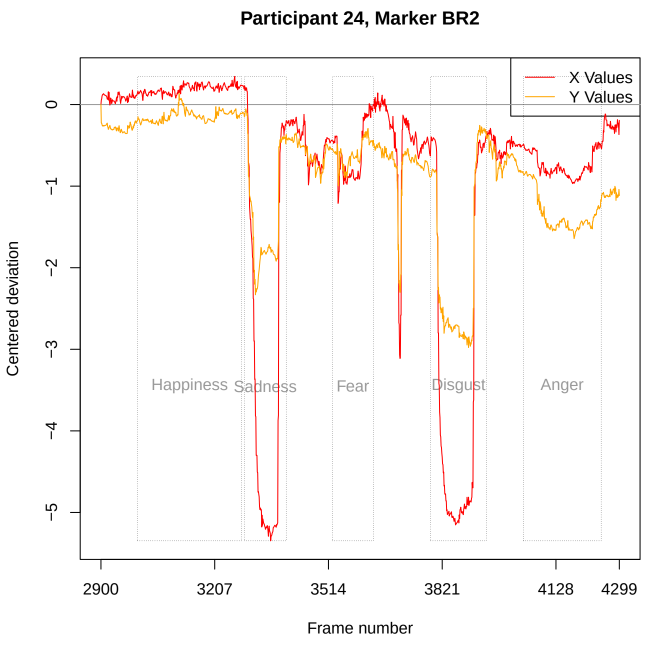
Participant 24, Marker BR2
X Values
Y Values
Happiness
Sadness
Fear
Disgust
Anger
Centered deviation
Frame number
2900
3207
3514
3821
4128
4299

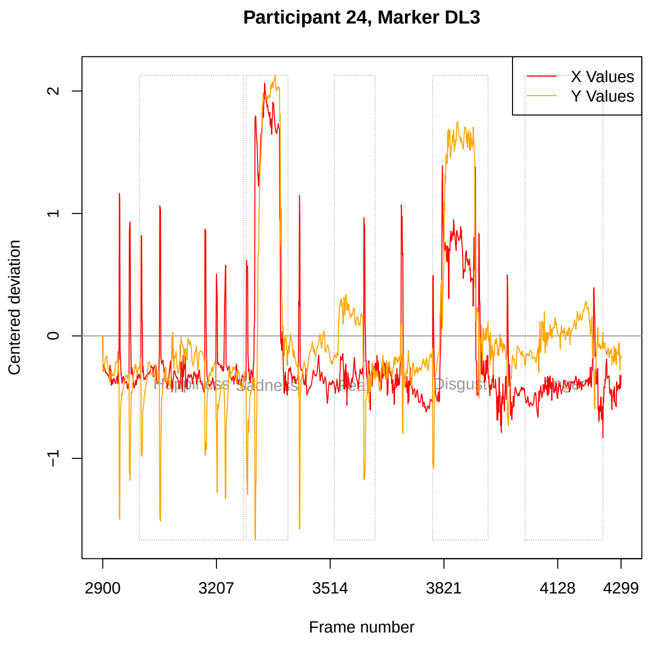
Participant 24, Marker DL3
X Values
Y Values
Happiness
Sadness
Fear
Disgust
Anger
Centered deviation
Frame number
2900
3207
3514
3821
4128
4299

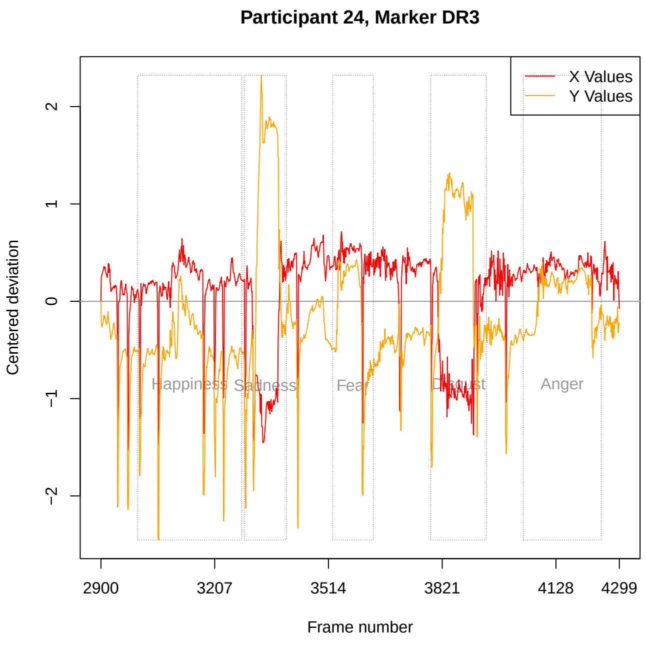
Participant 24, Marker DR3
X Values
Y Values
Happiness
Sadness
Fear
Disgust
Anger
Centered deviation
Frame number
2900
3207
3514
3821
4128
4299

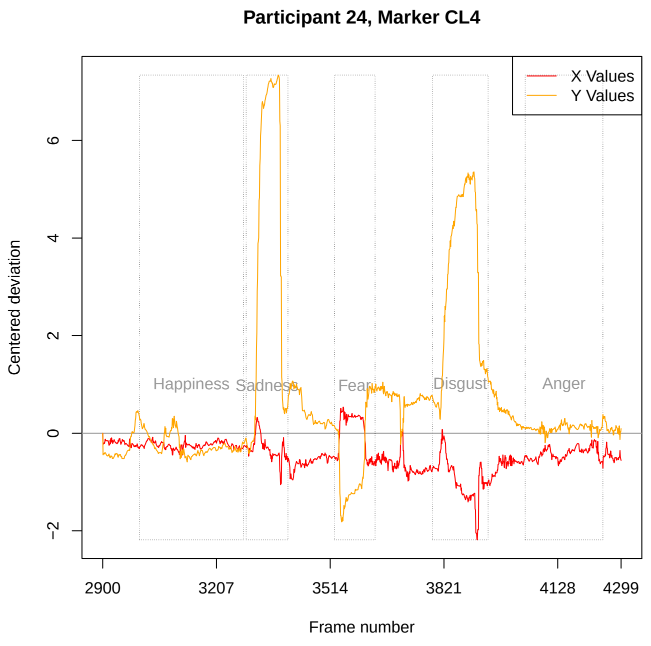
Participant 24, Marker CL4
X Values
Y Values
Happiness
Sadness
Fear
Disgust
Anger
Centered deviation
Frame number
2900
3207
3514
3821
4128
4299

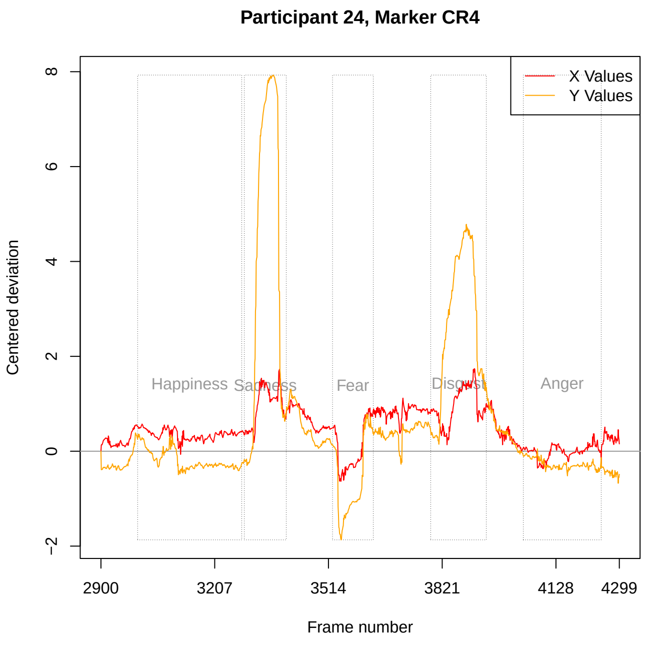
Participant 24, Marker CR4
X Values
Y Values
Happiness
Sadness
Fear
Disgust
Anger
Centered deviation
Frame number
2900
3207
3514
3821
4128
4299

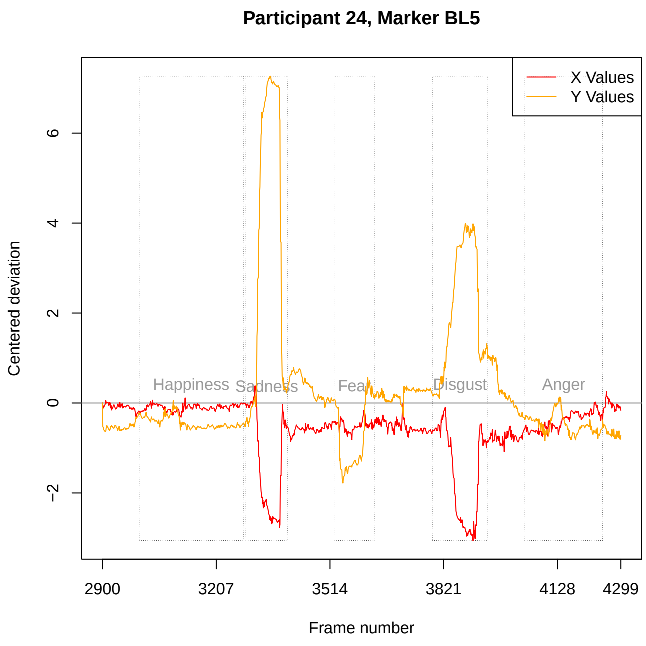
Participant 24, Marker BL5
X Values
Y Values
Happiness
Sadness
Fear
Disgust
Anger
Centered deviation
Frame number
2900
3207
3514
3821
4128
4299

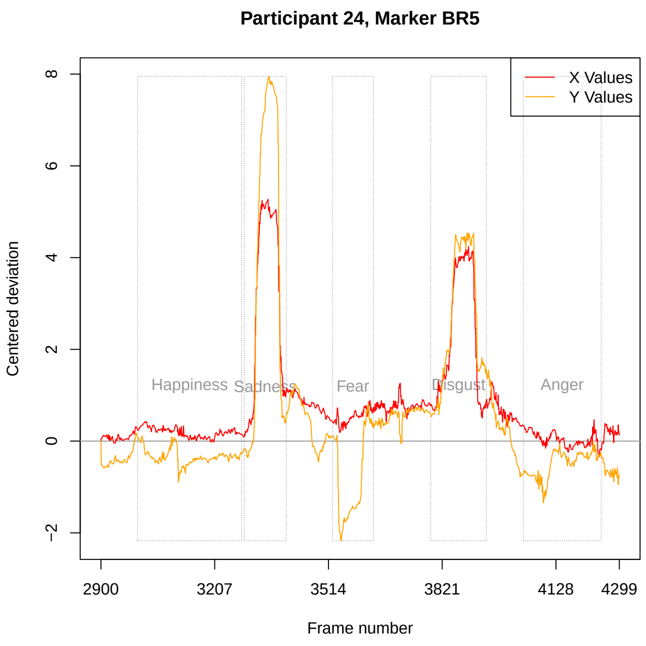
Participant 24, Marker BR5
X Values
Y Values
Happiness
Sadness
Fear
Disgust
Anger
Centered deviation
Frame number
2900
3207
3514
3821
4128
4299

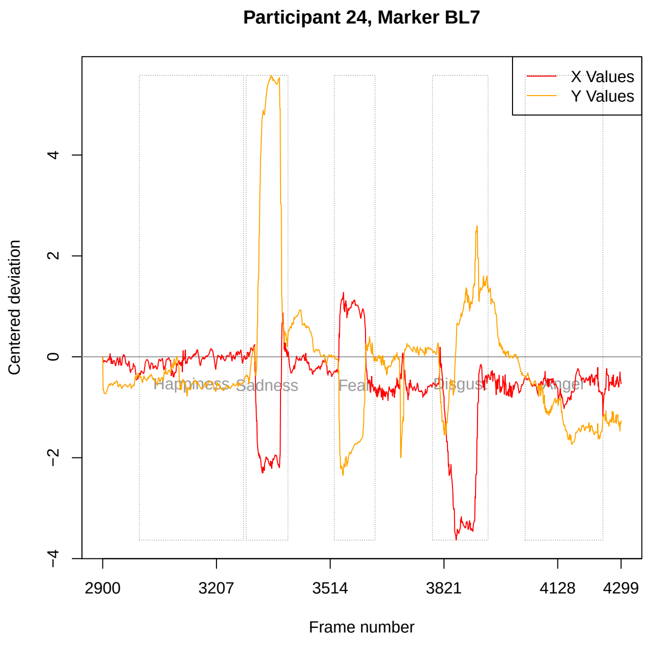
Participant 24, Marker BL7
X Values
Y Values
Happiness
Sadness
Fear
Disgust
Anger
Centered deviation
Frame number
2900
3207
3514
3821
4128
4299

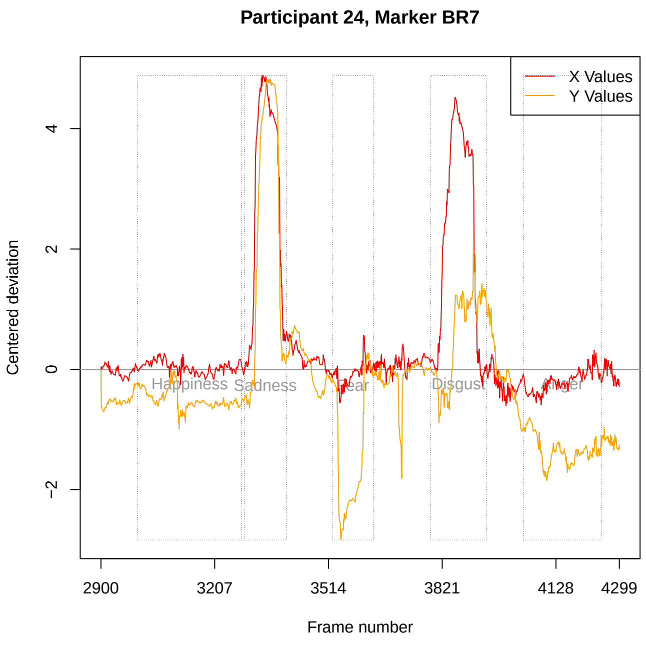
Participant 24, Marker BR7
X Values
Y Values
Happiness
Sadness
Fear
Disgust
Anger
Centered deviation
Frame number
2900
3207
3514
3821
4128
4299

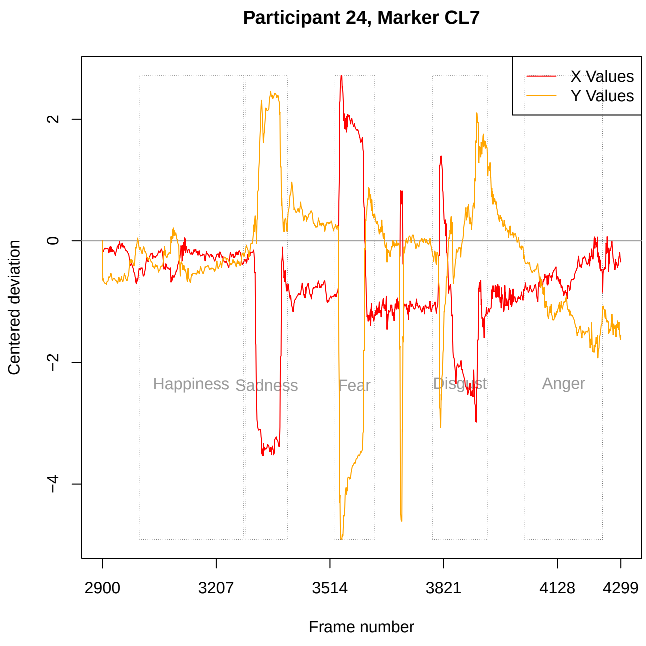
Participant 24, Marker CL7
X Values
Y Values
Happiness
Sadness
Fear
Disgust
Anger
Centered deviation
Frame number
2900
3207
3514
3821
4128
4299

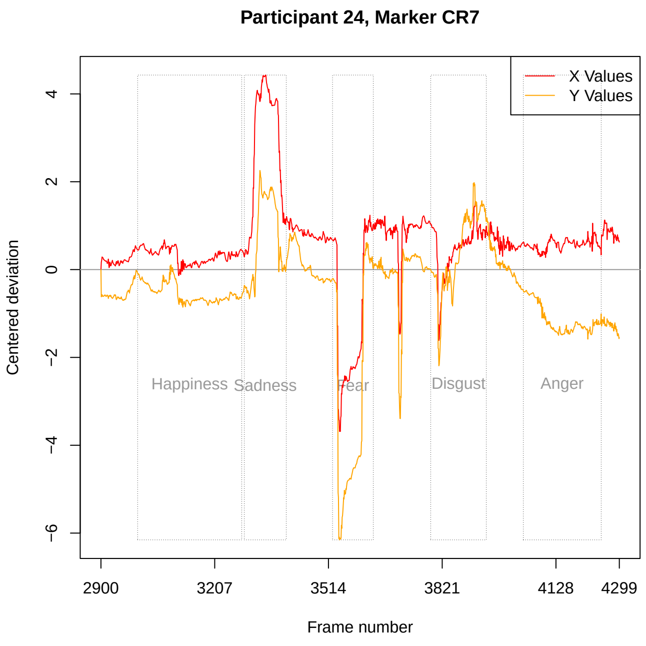
Participant 24, Marker CR7
X Values
Y Values
Happiness
Sadness
Fear
Disgust
Anger
Centered deviation
Frame number
2900
3207
3514
3821
4128
4299

Participant 24, Marker A7

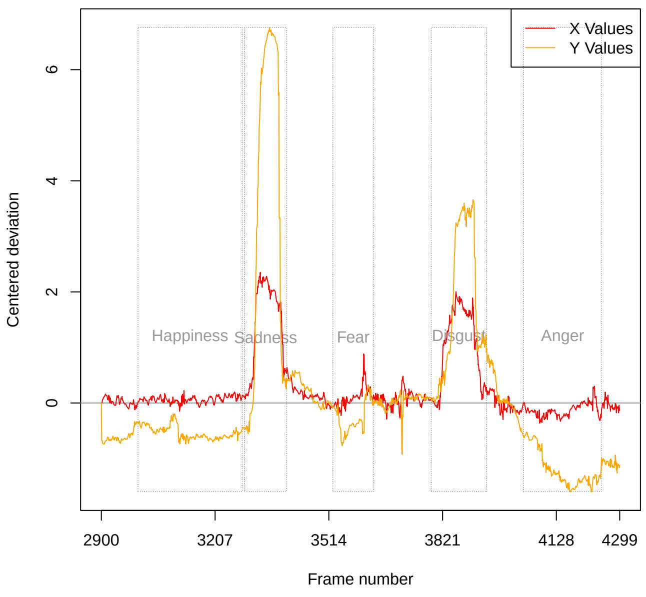

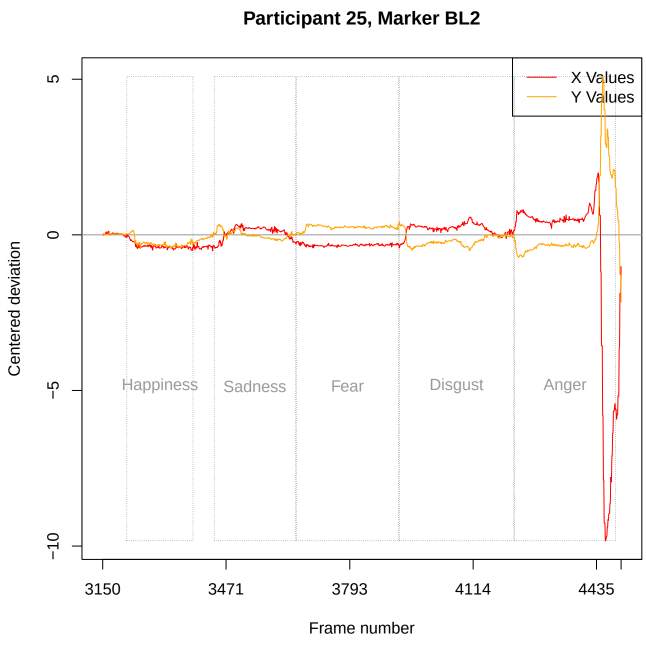
Participant 25, Marker BL2
X Values
Y Values
Centered deviation
5
0
−5
−10
Happiness
Sadness
Fear
Disgust
Anger
3150
3471
3793
4114
4435
Frame number

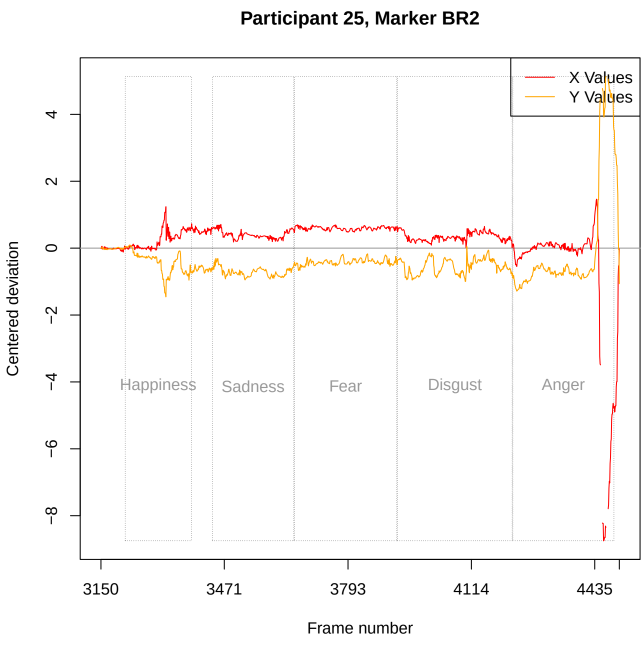
Participant 25, Marker BR2
X Values
Y Values
Centered deviation
4
2
0
−2
−4
−6
−8
Happiness
Sadness
Fear
Disgust
Anger
3150
3471
3793
4114
4435
Frame number

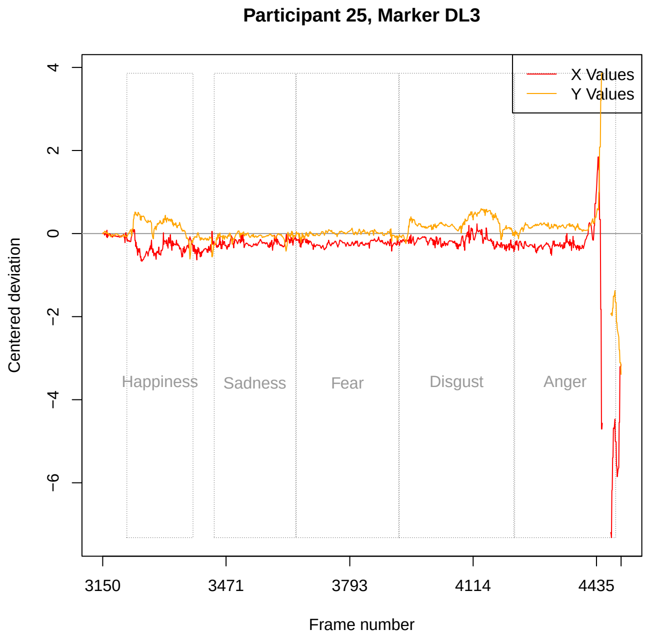
Participant 25, Marker DL3
X Values
Y Values
Centered deviation
4
2
0
−2
−4
−6
Happiness
Sadness
Fear
Disgust
Anger
3150
3471
3793
4114
4435
Frame number

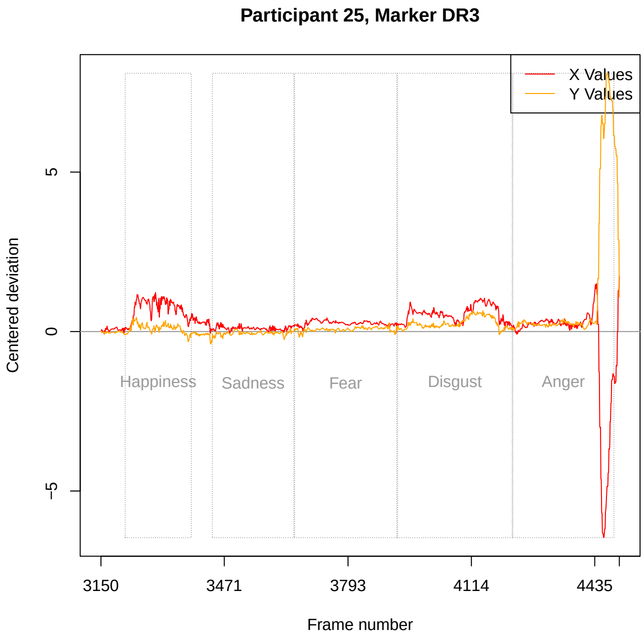
Participant 25, Marker DR3
X Values
Y Values
Centered deviation
5
0
−5
Happiness
Sadness
Fear
Disgust
Anger
3150
3471
3793
4114
4435
Frame number

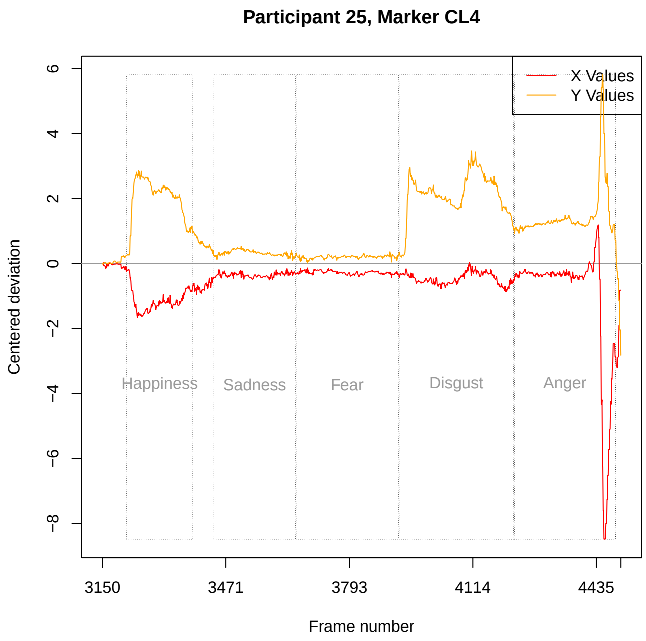
Participant 25, Marker CL4
X Values
Y Values
Centered deviation
6
4
2
0
−2
−4
−6
−8
Happiness
Sadness
Fear
Disgust
Anger
3150
3471
3793
4114
4435
Frame number

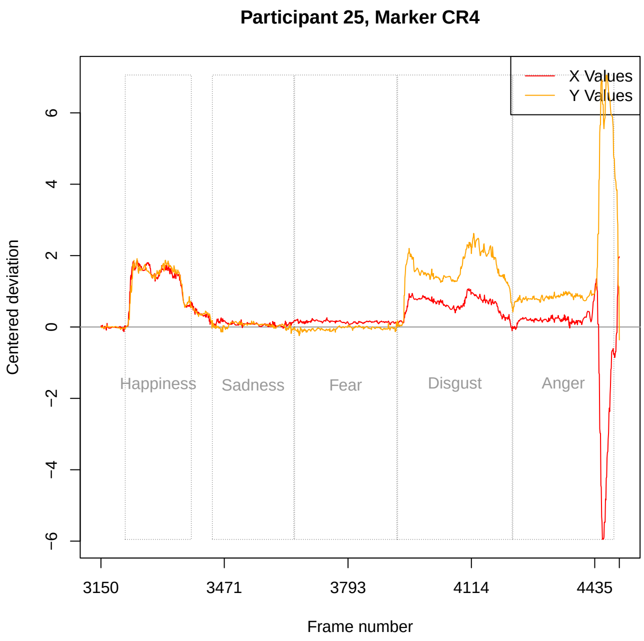
Participant 25, Marker CR4
X Values
Y Values
Centered deviation
6
4
2
0
−2
−4
−6
Happiness
Sadness
Fear
Disgust
Anger
3150
3471
3793
4114
4435
Frame number

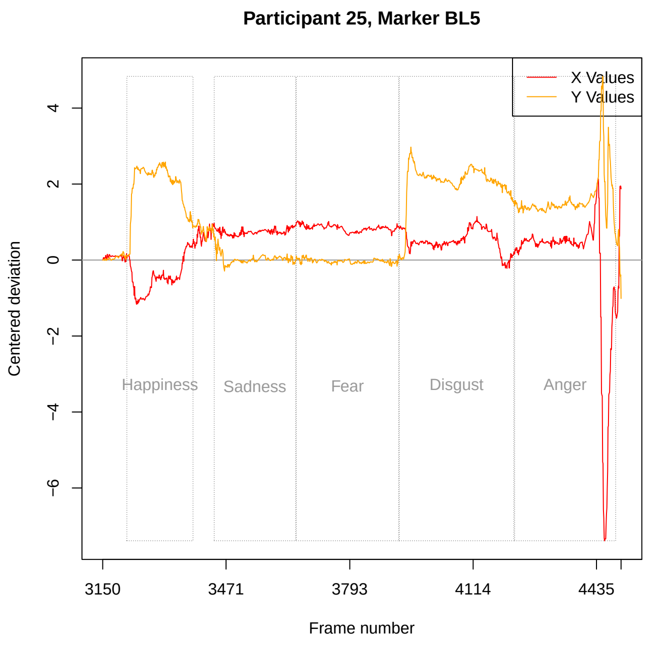
Participant 25, Marker BL5
X Values
Y Values
Centered deviation
Frame number
Happiness
Sadness
Fear
Disgust
Anger
3150
3471
3793
4114
4435
4
2
0
−2
−4
−6

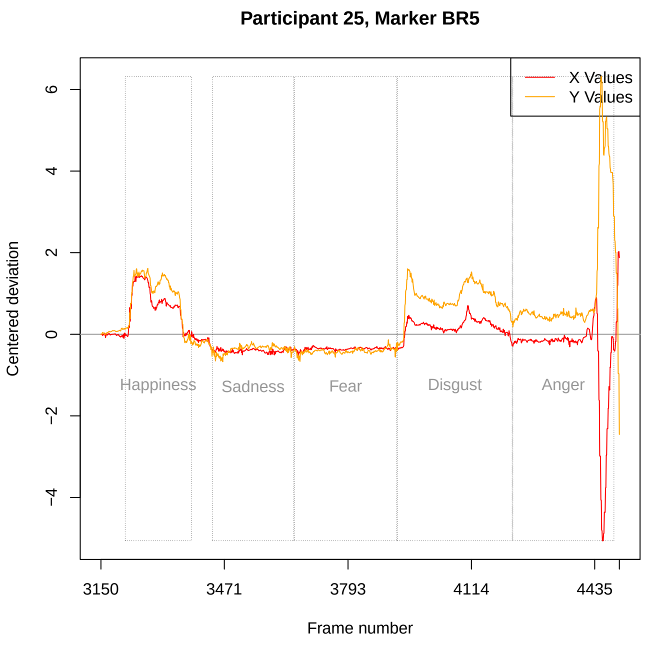
Participant 25, Marker BR5
X Values
Y Values
Centered deviation
Frame number
Happiness
Sadness
Fear
Disgust
Anger
3150
3471
3793
4114
4435
6
4
2
0
−2
−4

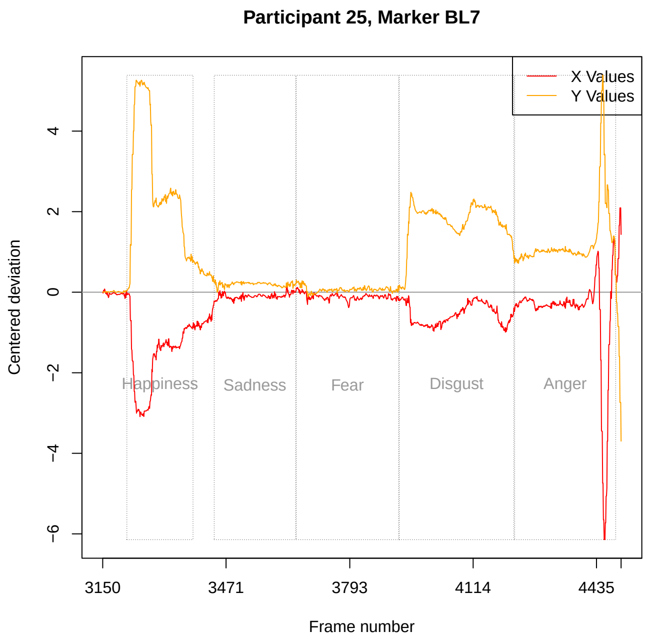
Participant 25, Marker BL7
X Values
Y Values
Centered deviation
Frame number
Happiness
Sadness
Fear
Disgust
Anger
3150
3471
3793
4114
4435
4
2
0
−2
−4
−6

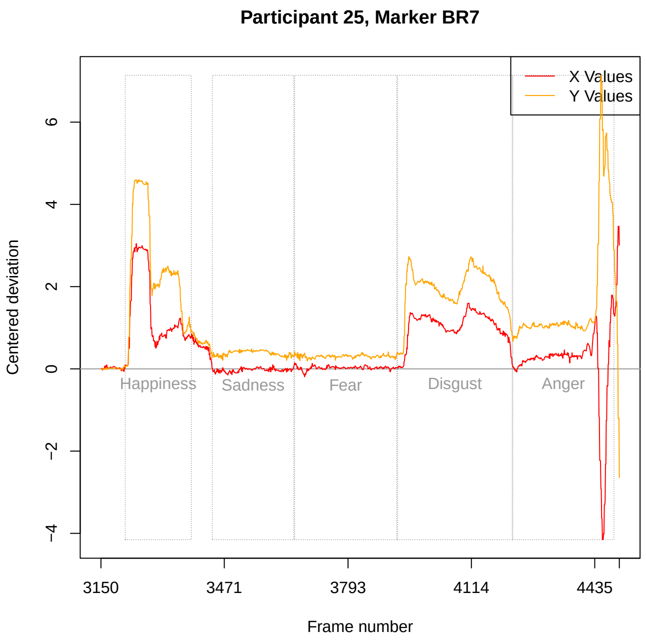
Participant 25, Marker BR7
X Values
Y Values
Centered deviation
Frame number
Happiness
Sadness
Fear
Disgust
Anger
3150
3471
3793
4114
4435
6
4
2
0
−2
−4

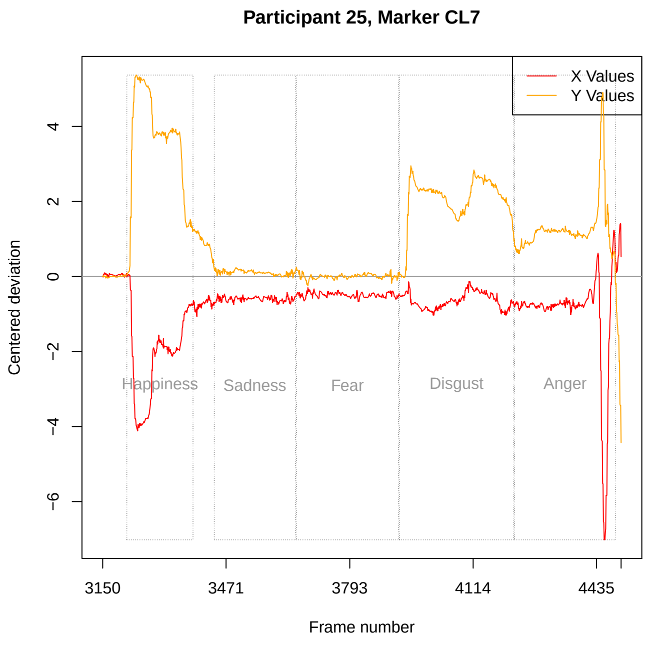
Participant 25, Marker CL7
X Values
Y Values
Centered deviation
Frame number
Happiness
Sadness
Fear
Disgust
Anger
3150
3471
3793
4114
4435
4
2
0
−2
−4
−6

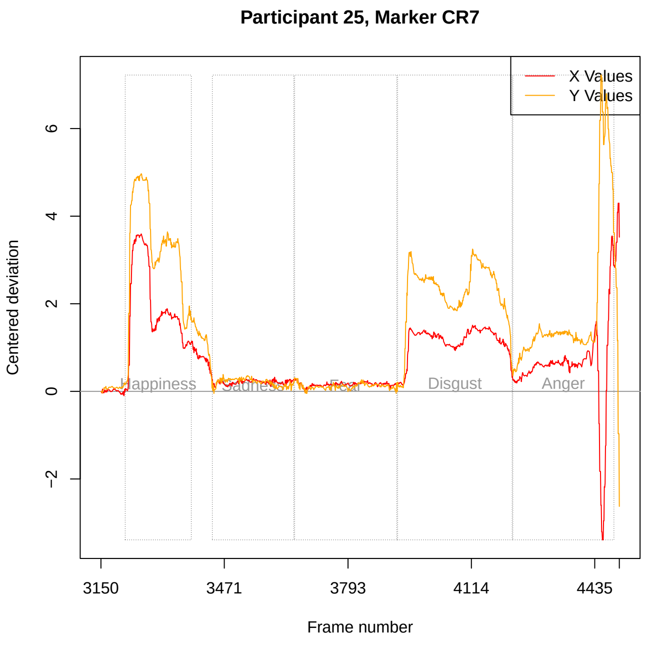
Participant 25, Marker CR7
X Values
Y Values
Centered deviation
Frame number
Happiness
Disgust
Anger
3150
3471
3793
4114
4435
6
4
2
0
−2

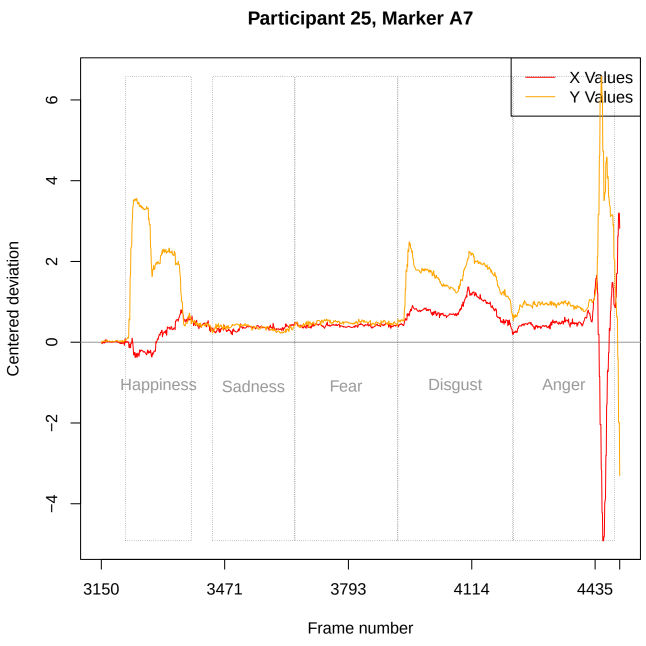

Participant 25, Marker A7
X Values
Y Values
Centered deviation
6
4
2
0
−2
−4
Happiness
Sadness
Fear
Disgust
Anger
3150
3471
3793
4114
4435
Frame number

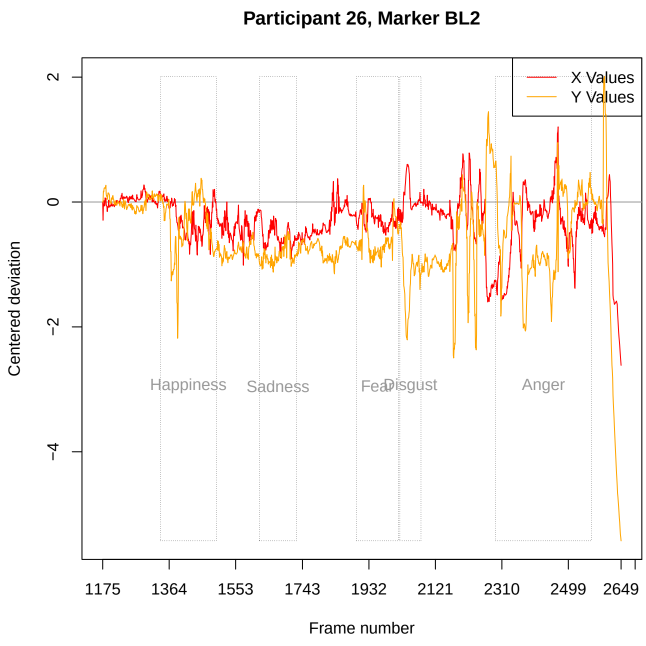

Participant 26, Marker BL2
X Values
Y Values
Happiness
Sadness
Fear
Disgust
Anger
Centered deviation
Frame number
1175
1364
1553
1743
1932
2121
2310
2499
2649

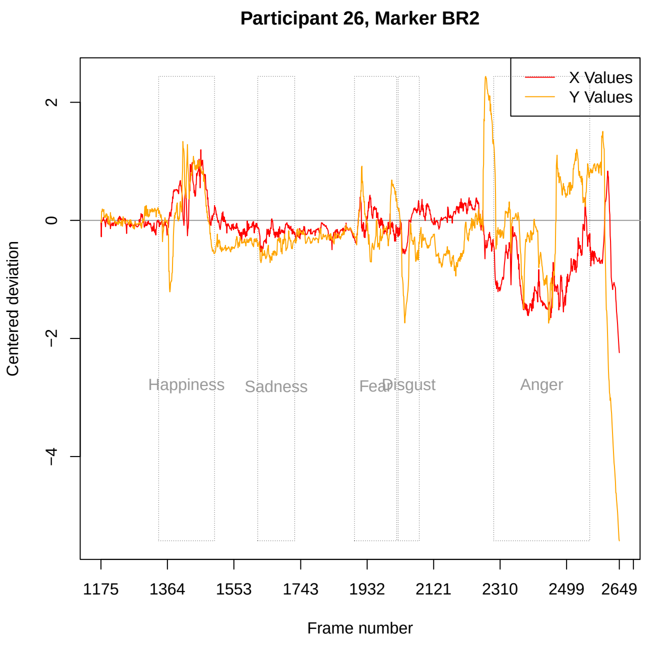

Participant 26, Marker BR2
X Values
Y Values
Happiness
Sadness
Fear
Disgust
Anger
Centered deviation
Frame number
1175
1364
1553
1743
1932
2121
2310
2499
2649

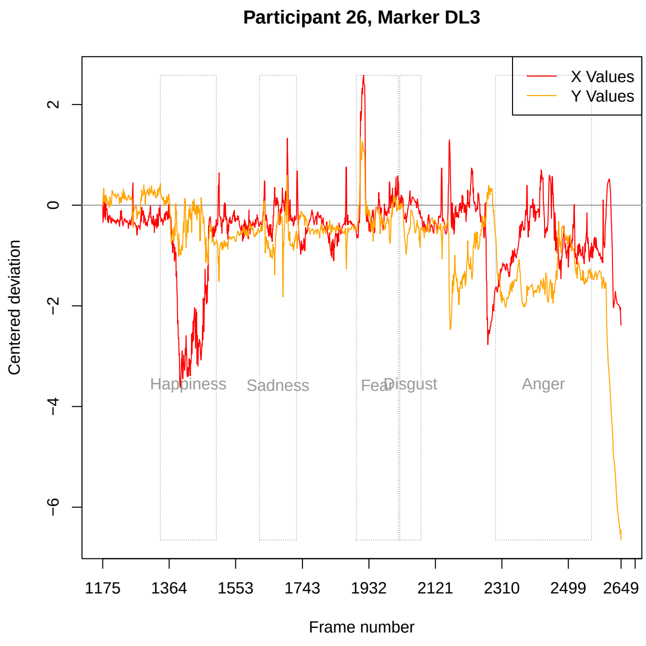

Participant 26, Marker DL3
X Values
Y Values
Happiness
Sadness
Fear
Disgust
Anger
Centered deviation
Frame number
1175
1364
1553
1743
1932
2121
2310
2499
2649

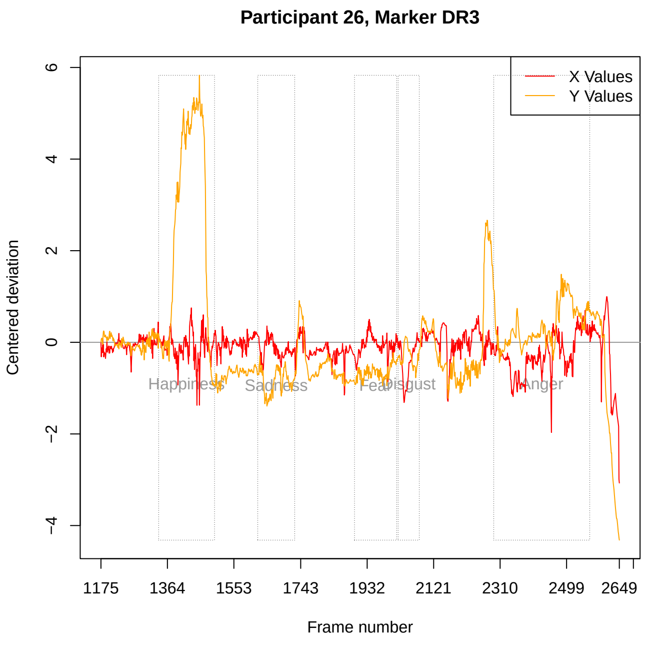

Participant 26, Marker DR3
X Values
Y Values
Happiness
Sadness
Fear
Disgust
Anger
Centered deviation
Frame number
1175
1364
1553
1743
1932
2121
2310
2499
2649

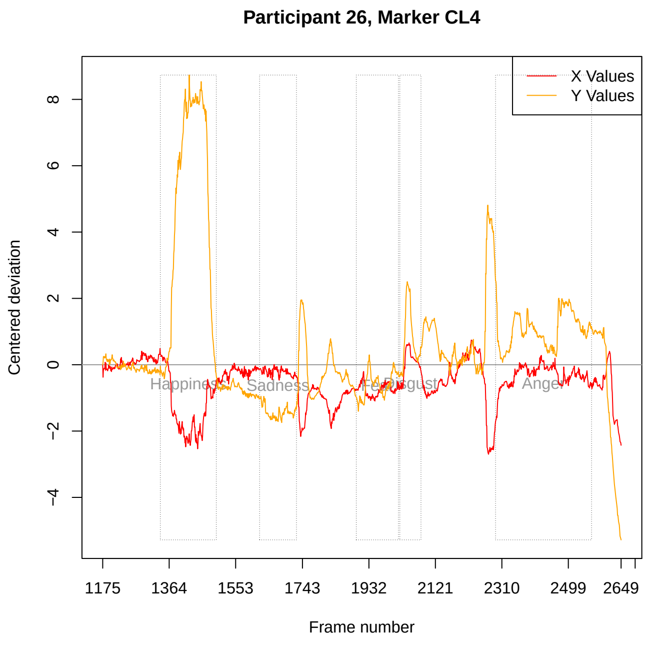

Participant 26, Marker CL4
X Values
Y Values
Happiness
Sadness
Fear
Disgust
Anger
Centered deviation
Frame number
1175
1364
1553
1743
1932
2121
2310
2499
2649

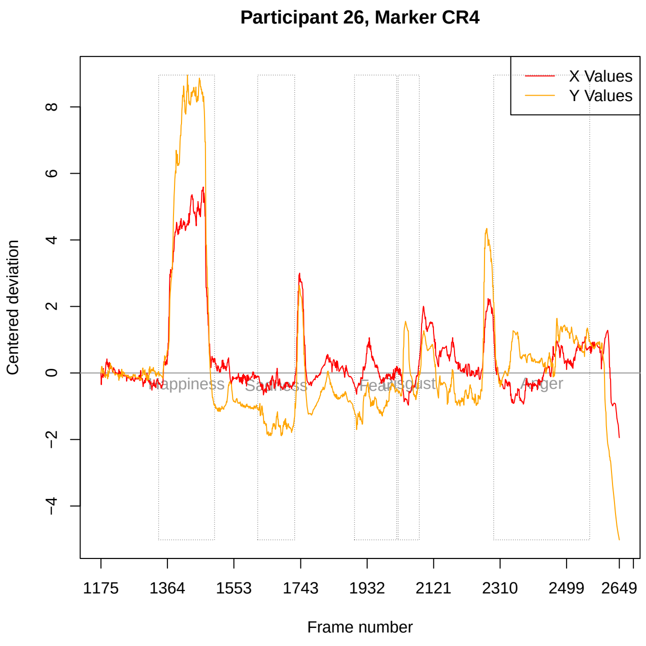

Participant 26, Marker CR4
X Values
Y Values
Happiness
Sadness
Fear
Disgust
Anger
Centered deviation
Frame number
1175
1364
1553
1743
1932
2121
2310
2499
2649

Participant 26, Marker BL5

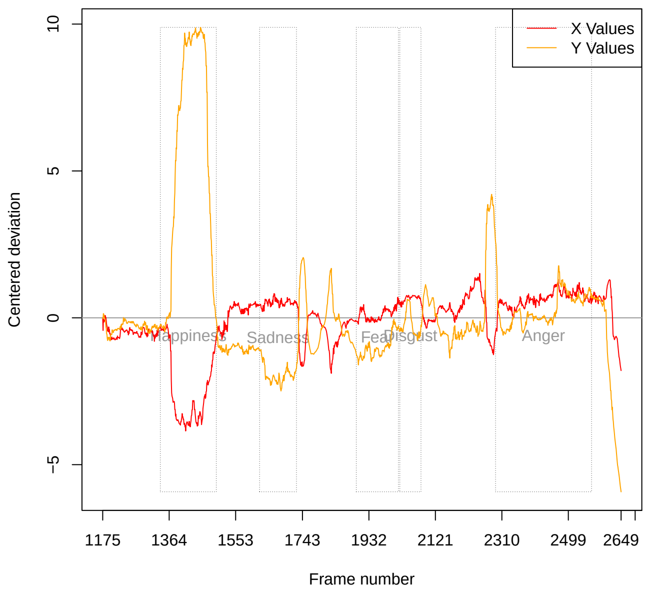

Participant 26, Marker BR5

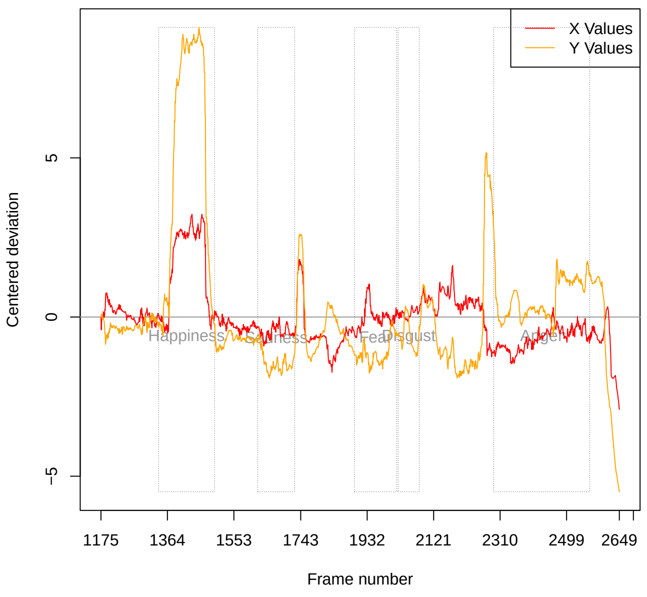

Participant 26, Marker BL7

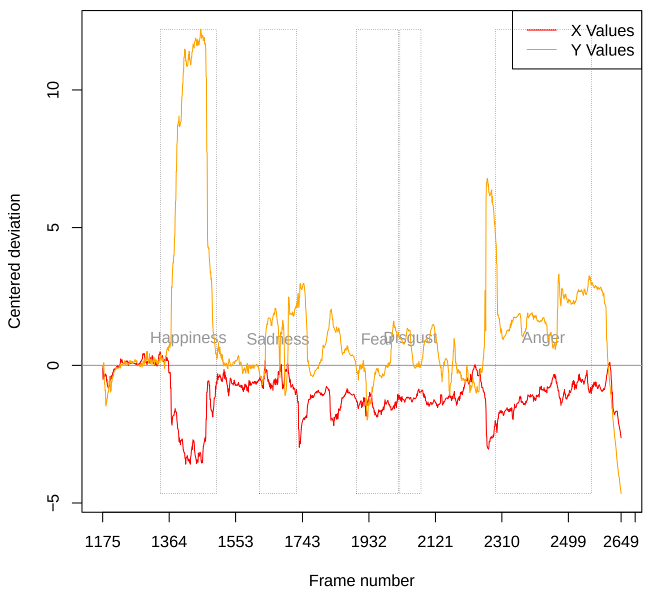

Participant 26, Marker BR7

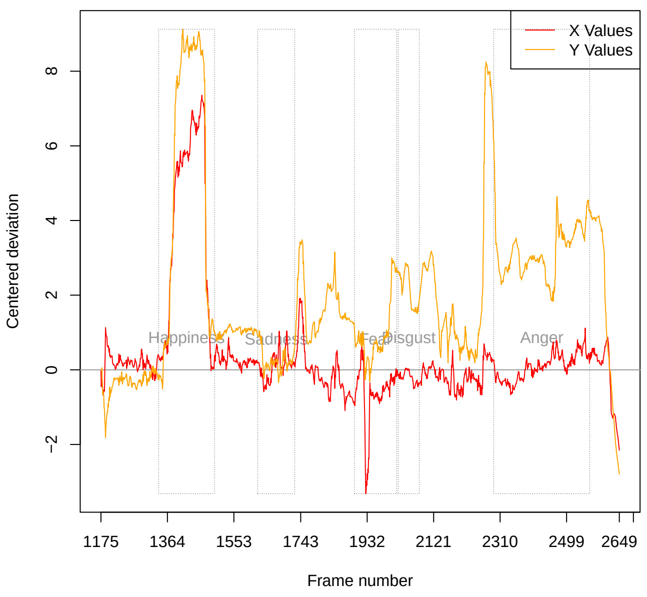

Participant 26, Marker CL7

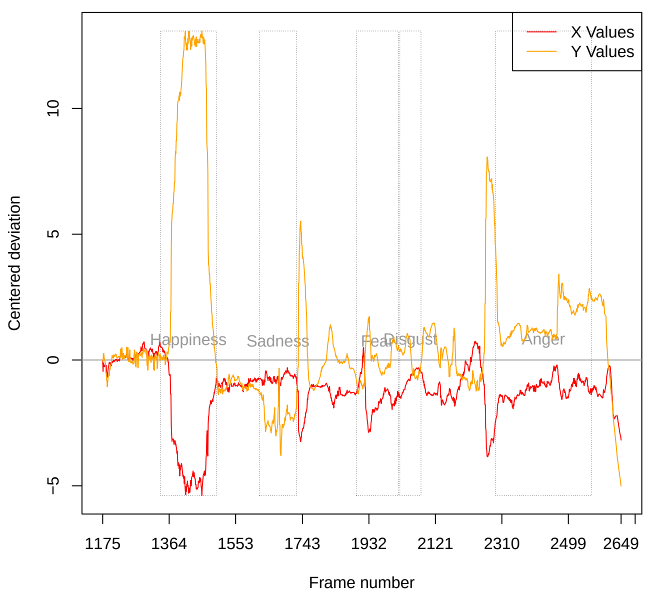

Participant 26, Marker CR7

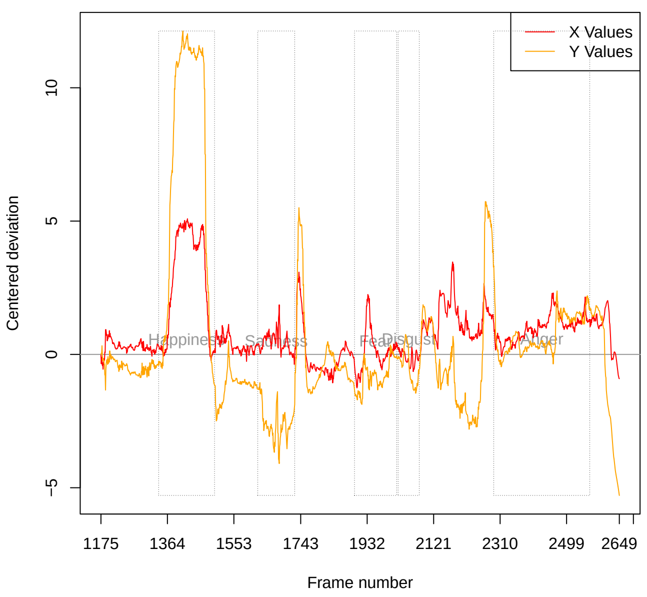

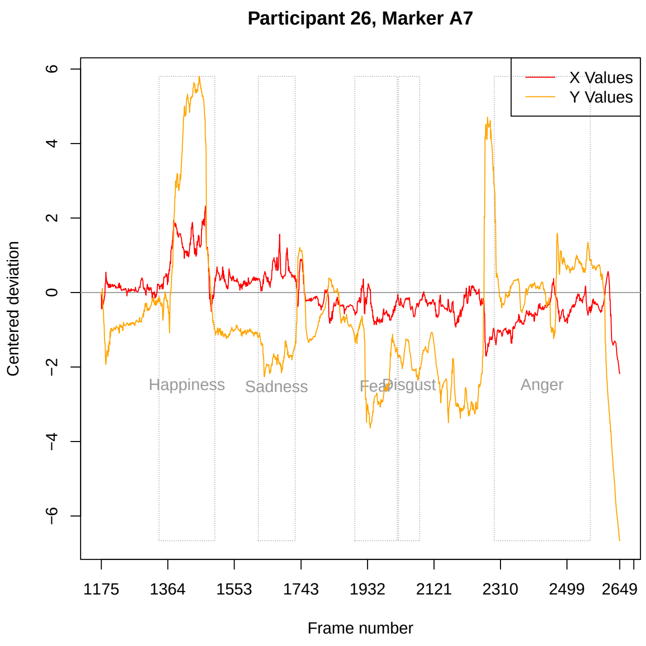

Participant 26, Marker A7
X Values
Y Values
Centered deviation
6
4
2
0
−2
−4
−6
Happiness
Sadness
Fear
Disgust
Anger
1175
1364
1553
1743
1932
2121
2310
2499
2649
Frame number

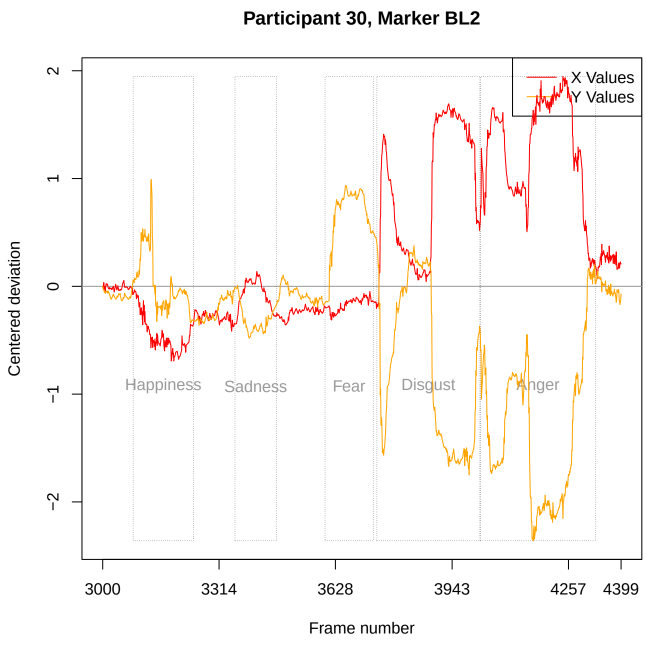
Participant 30, Marker BL2
X Values
Y Values
Happiness
Sadness
Fear
Disgust
Anger
Centered deviation
Frame number
3000
3314
3628
3943
4257
4399

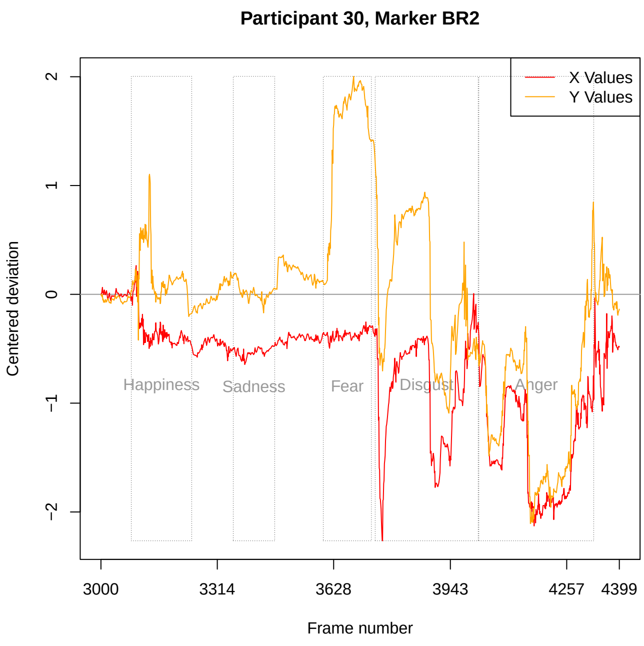
Participant 30, Marker BR2
X Values
Y Values
Happiness
Sadness
Fear
Disgust
Anger
Centered deviation
Frame number
3000
3314
3628
3943
4257
4399

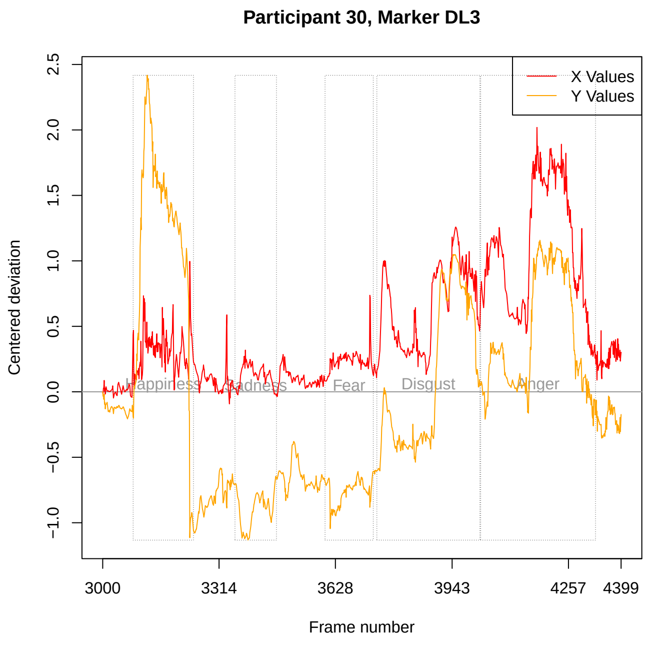
Participant 30, Marker DL3
X Values
Y Values
Happiness
Sadness
Fear
Disgust
Anger
Centered deviation
Frame number
3000
3314
3628
3943
4257
4399

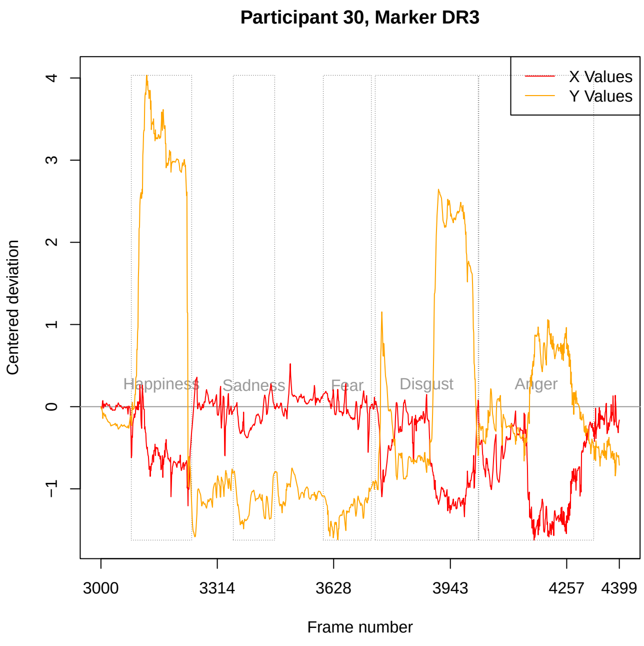
Participant 30, Marker DR3
X Values
Y Values
Happiness
Sadness
Fear
Disgust
Anger
Centered deviation
Frame number
3000
3314
3628
3943
4257
4399

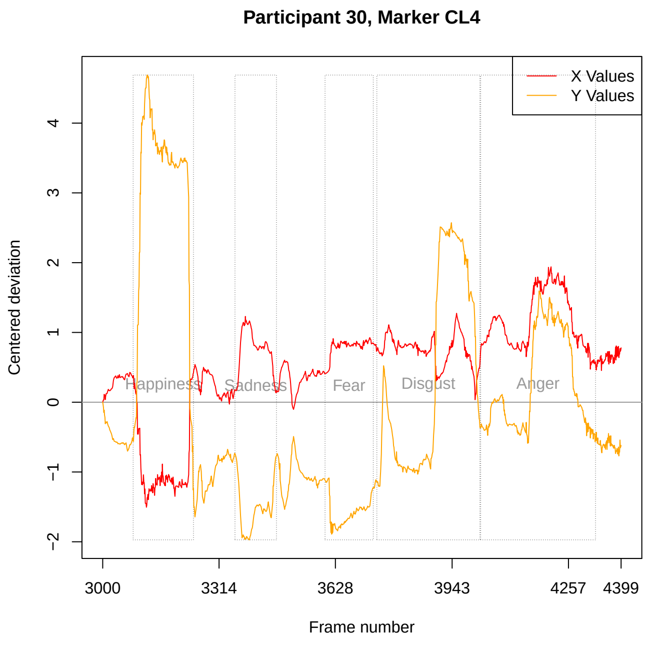
Participant 30, Marker CL4
X Values
Y Values
Happiness
Sadness
Fear
Disgust
Anger
Centered deviation
Frame number
3000
3314
3628
3943
4257
4399

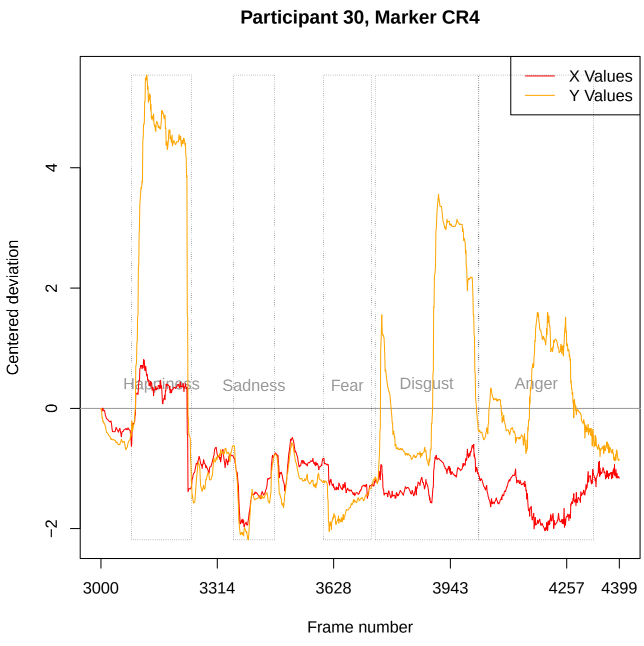
Participant 30, Marker CR4
X Values
Y Values
Happiness
Sadness
Fear
Disgust
Anger
Centered deviation
Frame number
3000
3314
3628
3943
4257
4399

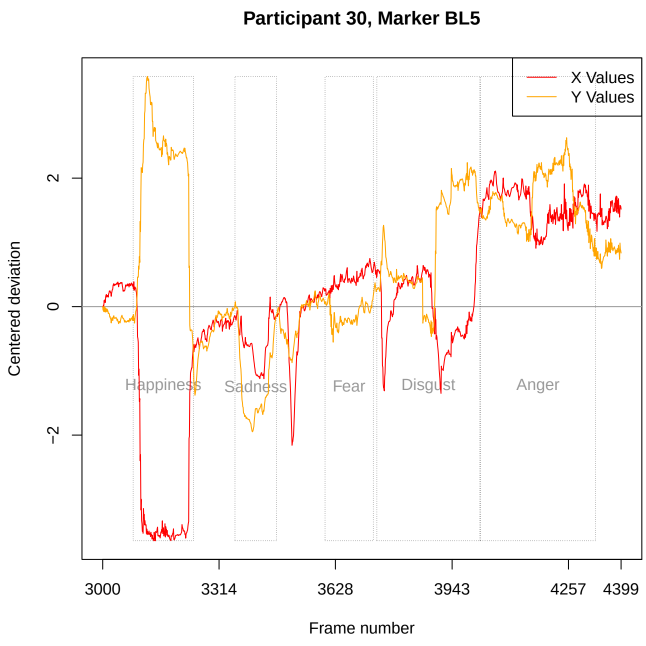
Participant 30, Marker BL5
X Values
Y Values
Happiness
Sadness
Fear
Disgust
Anger
Centered deviation
Frame number
3000
3314
3628
3943
4257
4399

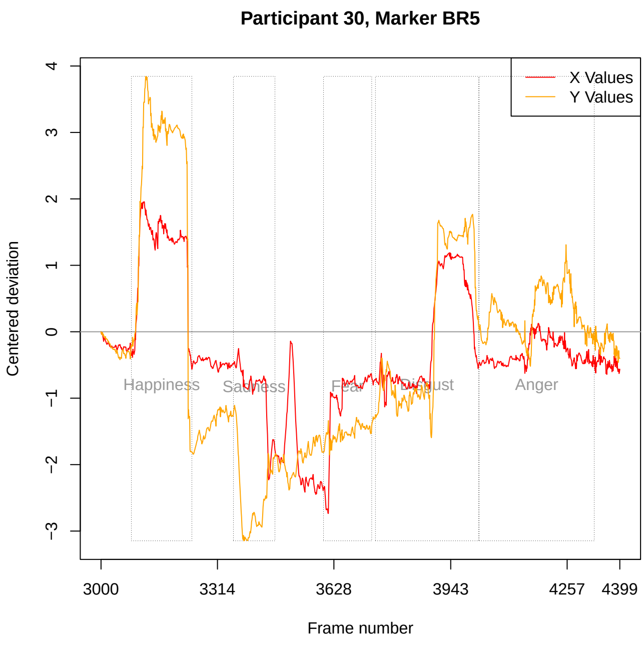
Participant 30, Marker BR5
X Values
Y Values
Happiness
Sadness
Fear
Disgust
Anger
Centered deviation
Frame number
3000
3314
3628
3943
4257
4399

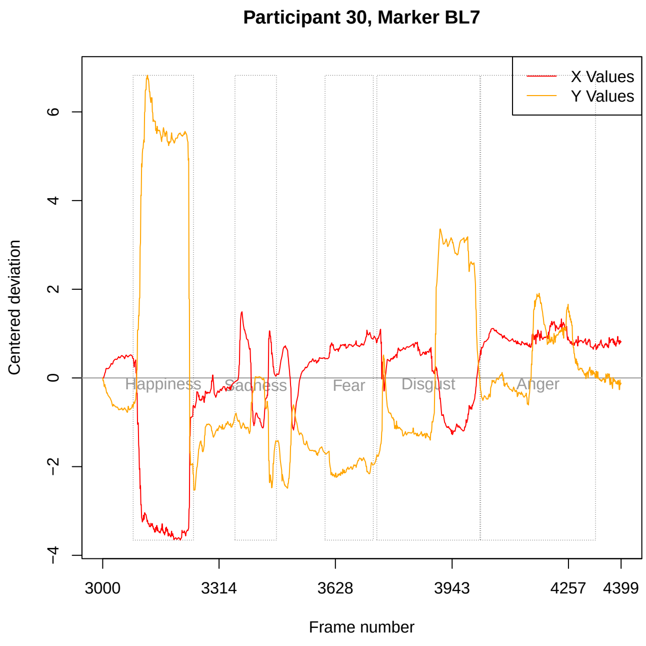
Participant 30, Marker BL7
X Values
Y Values
Happiness
Sadness
Fear
Disgust
Anger
Centered deviation
Frame number
3000
3314
3628
3943
4257
4399

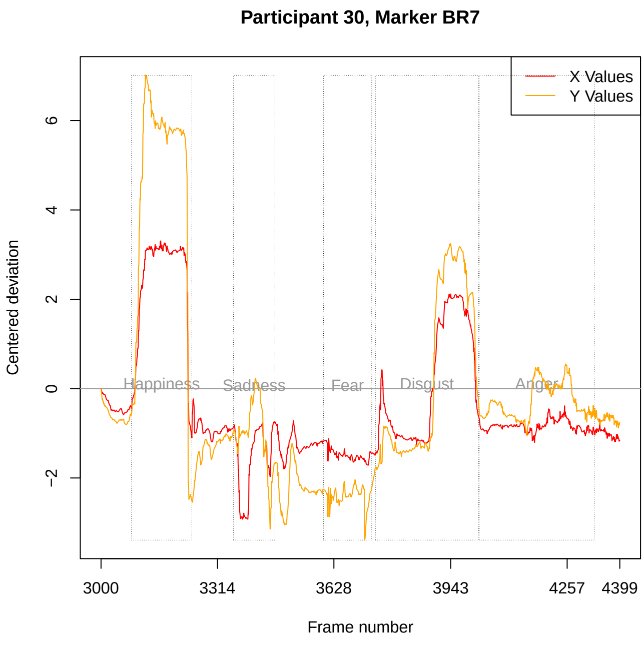
Participant 30, Marker BR7
X Values
Y Values
Happiness
Sadness
Fear
Disgust
Anger
Centered deviation
Frame number
3000
3314
3628
3943
4257
4399

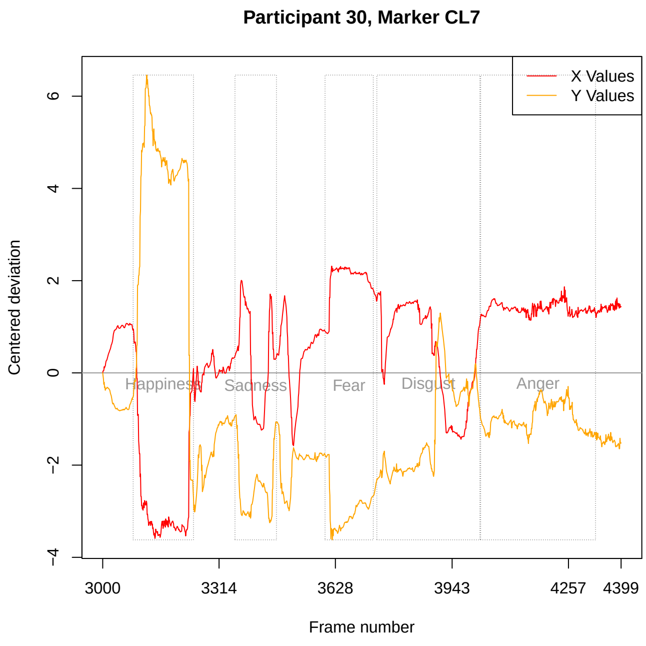
Participant 30, Marker CL7
X Values
Y Values
Happiness
Sadness
Fear
Disgust
Anger
Centered deviation
Frame number
3000
3314
3628
3943
4257
4399

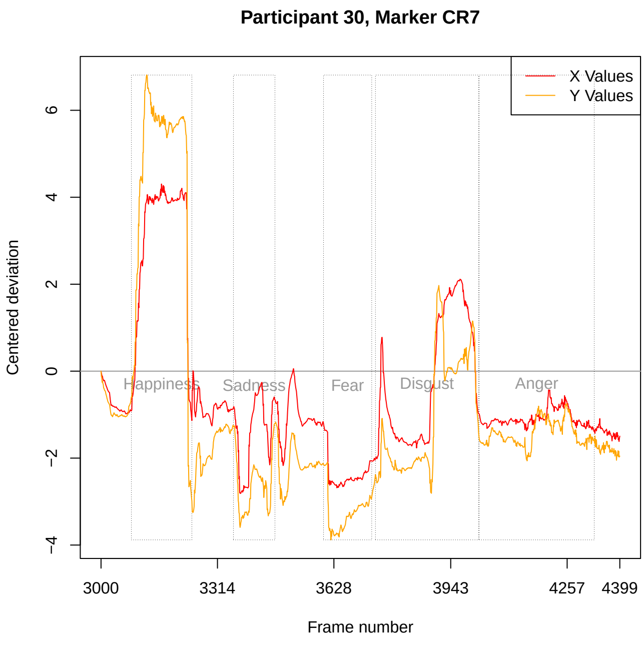
Participant 30, Marker CR7
X Values
Y Values
Happiness
Sadness
Fear
Disgust
Anger
Centered deviation
Frame number
3000
3314
3628
3943
4257
4399

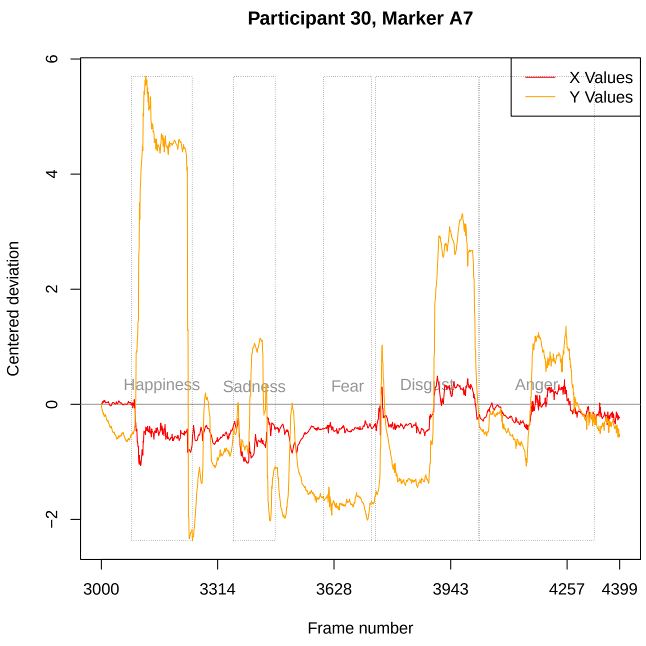

Participant 30, Marker A7
X Values
Y Values
Happiness
Sadness
Fear
Disgust
Anger
Centered deviation
−2
0
2
4
6
3000
3314
3628
3943
4257
4399
Frame number

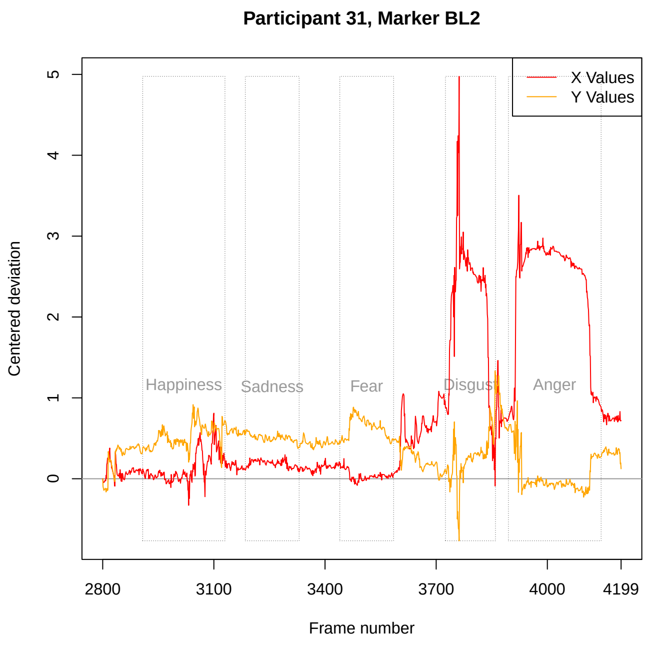
Participant 31, Marker BL2
Centered deviation
Frame number
X Values
Y Values
Happiness
Sadness
Fear
Disgust
Anger
0
1
2
3
4
5
2800
3100
3400
3700
4000
4199

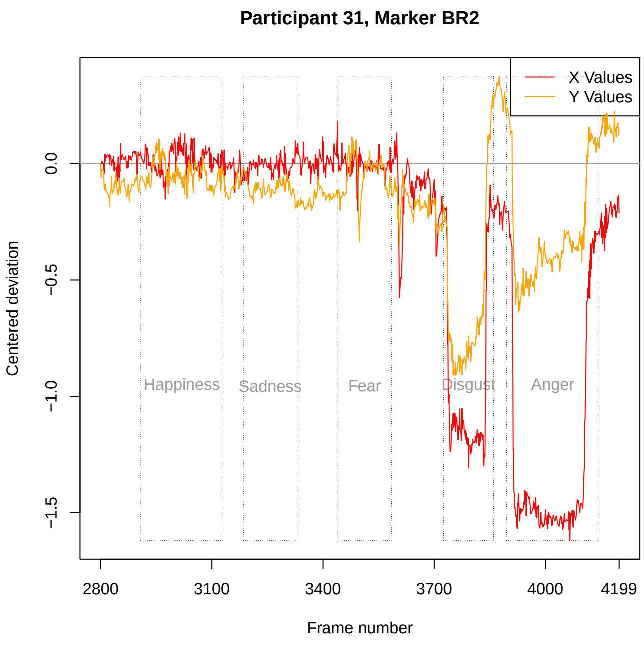
Participant 31, Marker BR2
Centered deviation
Frame number
X Values
Y Values
Happiness
Sadness
Fear
Disgust
Anger
0.0
−0.5
−1.0
−1.5
2800
3100
3400
3700
4000
4199

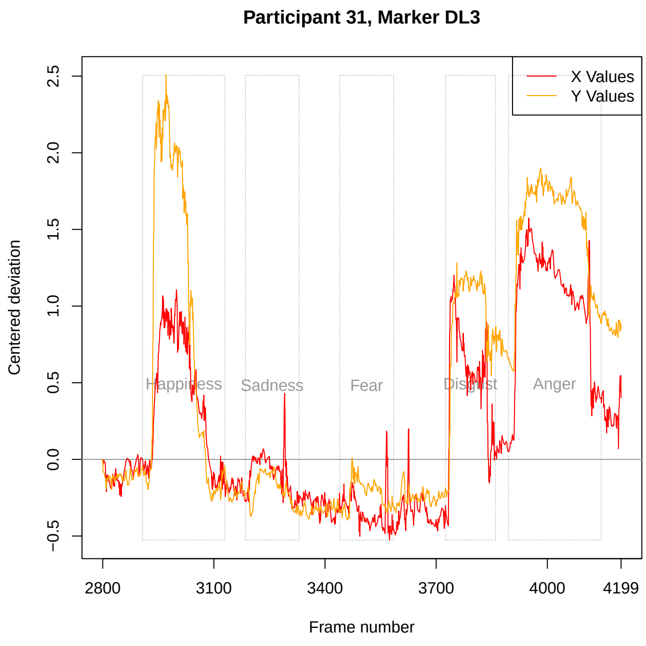
Participant 31, Marker DL3
Centered deviation
Frame number
X Values
Y Values
Happiness
Sadness
Fear
Disgust
Anger
−0.5
0.0
0.5
1.0
1.5
2.0
2.5
2800
3100
3400
3700
4000
4199

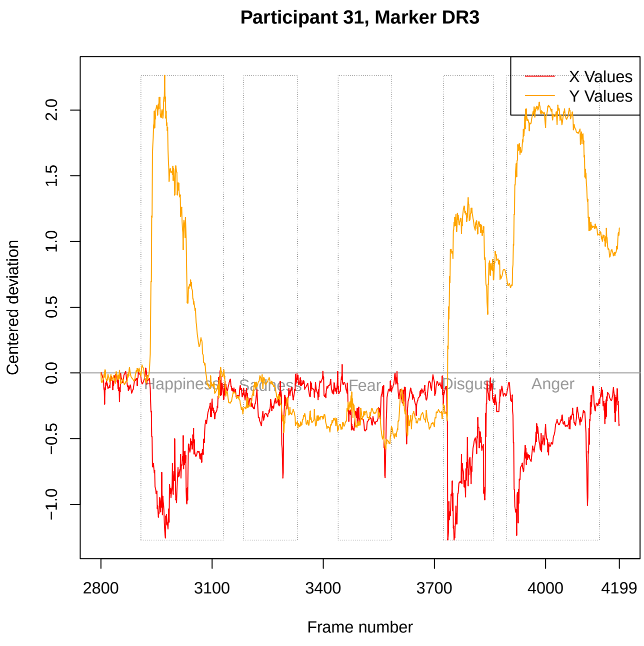
Participant 31, Marker DR3
Centered deviation
Frame number
X Values
Y Values
Happiness
Sadness
Fear
Disgust
Anger
−1.0
−0.5
0.0
0.5
1.0
1.5
2.0
2800
3100
3400
3700
4000
4199

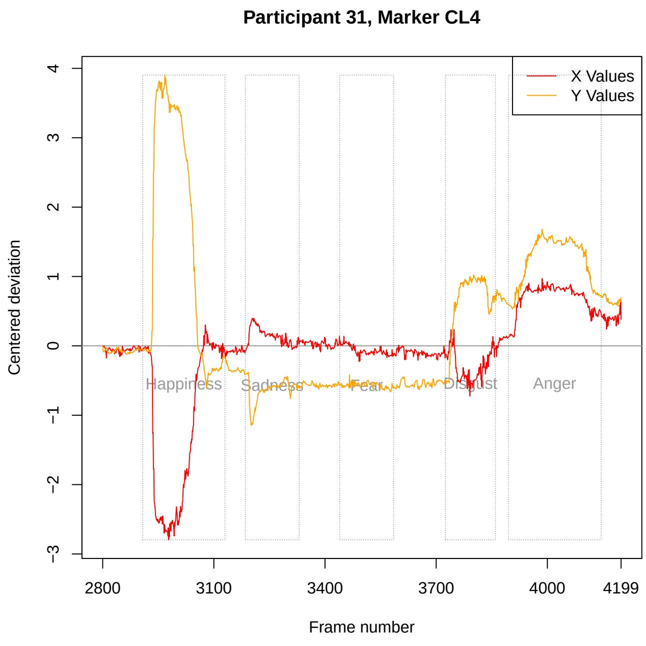
Participant 31, Marker CL4
Centered deviation
Frame number
X Values
Y Values
Happiness
Sadness
Fear
Disgust
Anger
−3
−2
−1
0
1
2
3
4
2800
3100
3400
3700
4000
4199

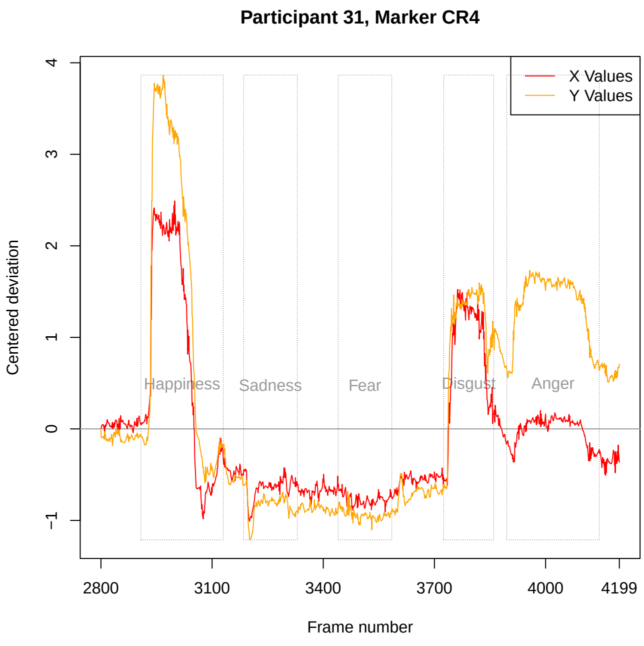
Participant 31, Marker CR4
Centered deviation
Frame number
X Values
Y Values
Happiness
Sadness
Fear
Disgust
Anger
−1
0
1
2
3
4
2800
3100
3400
3700
4000
4199

Participant 31, Marker BL5

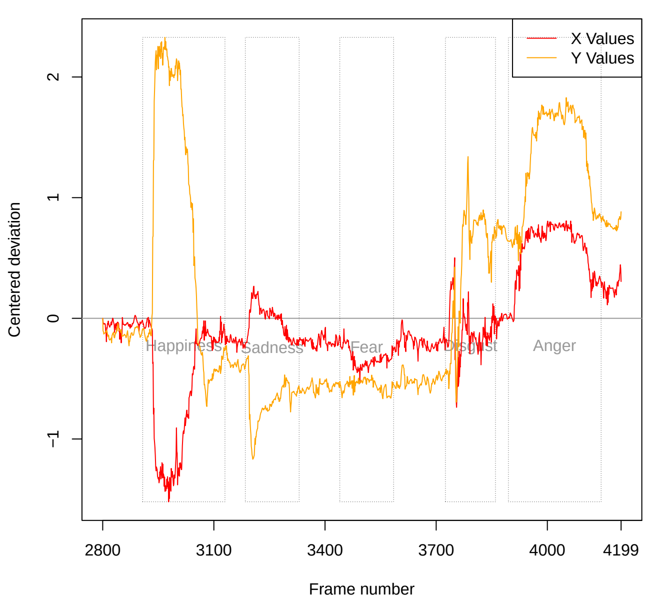

Participant 31, Marker BR5

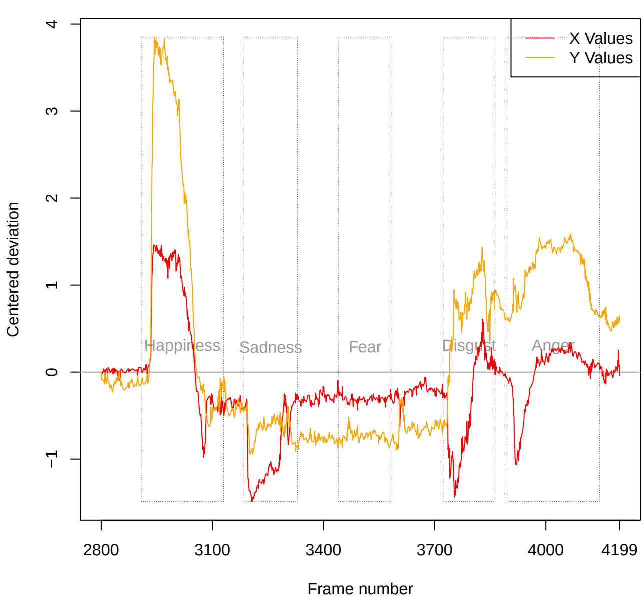

Participant 31, Marker BL7

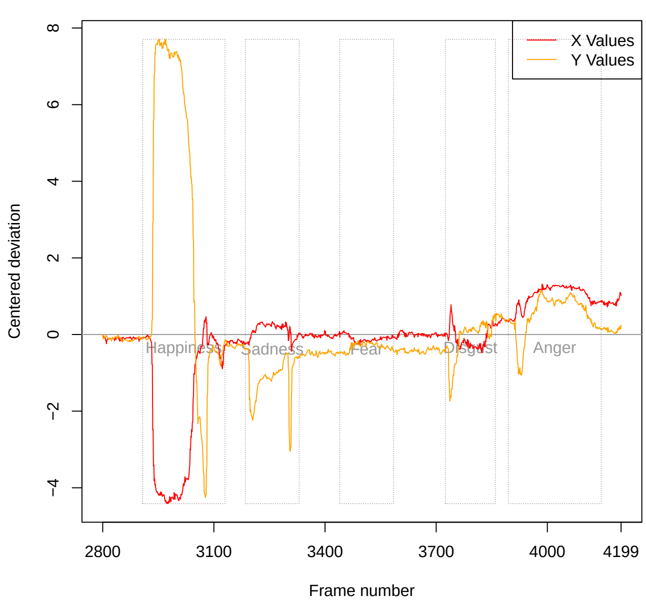

Participant 31, Marker BR7

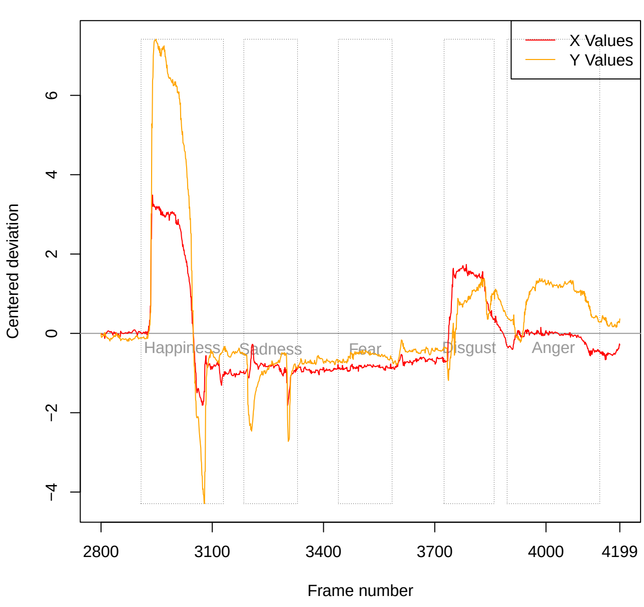

Participant 31, Marker CL7

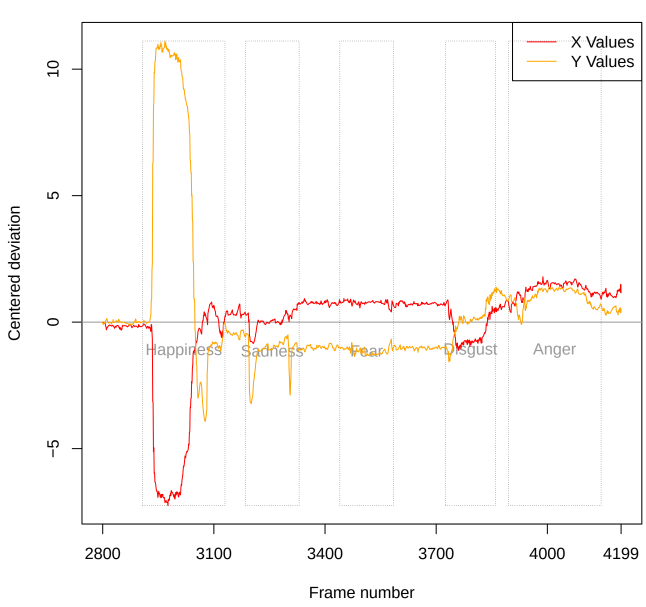

Participant 31, Marker CR7

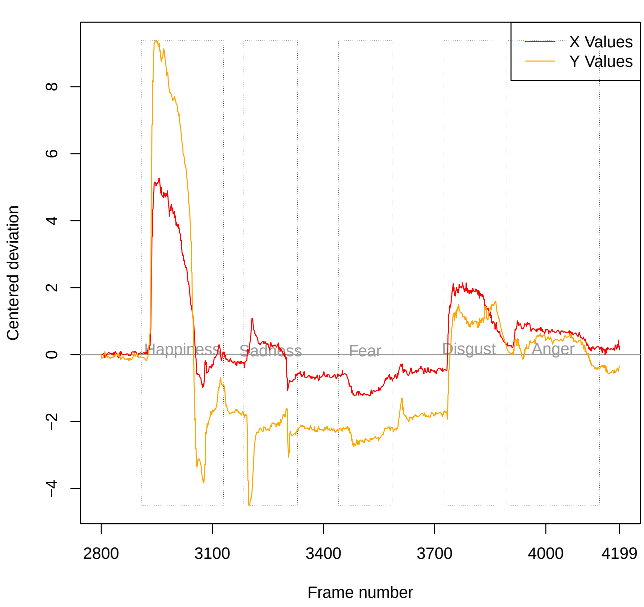

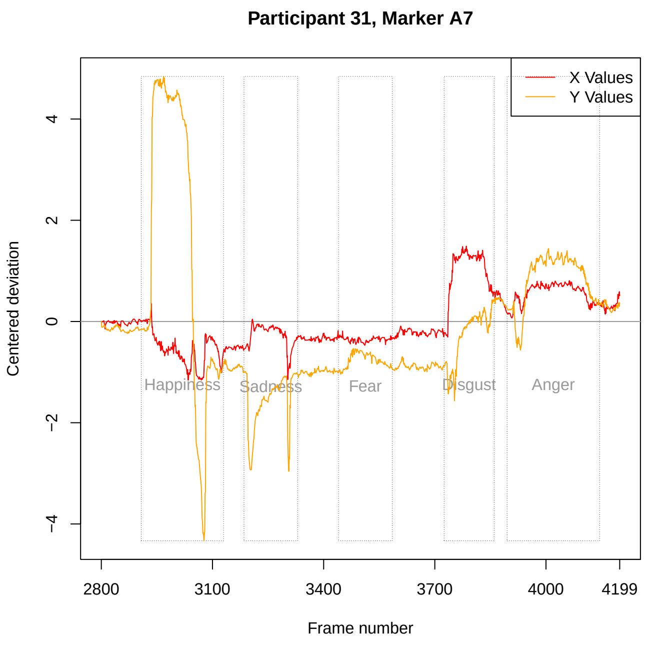

Participant 31, Marker A7
X Values
Y Values
Centered deviation
4
2
0
−2
−4
Happiness
Sadness
Fear
Disgust
Anger
2800
3100
3400
3700
4000
4199
Frame number
