# Supplementary material for: blenderFaceUV: Toward Marker‐Based Precision and Flexibility in Facial Tracking With “Invisible” Markers
Source: Psychophysiology. 2026 Sep 21;63(9):e70394. doi: 10.1111/psyp.70394 (PMC13594373; doi:10.1111/psyp.70394)

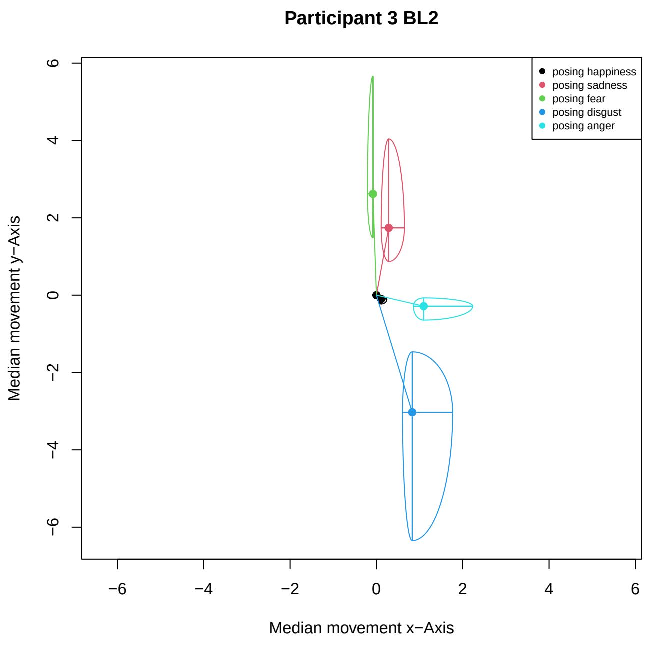
Participant 3 BL2
posing happiness
posing sadness
posing fear
posing disgust
posing anger
Median movement y−Axis
Median movement x−Axis
−6 −4 −2 0 2 4 6
6 4 2 0 −2 −4 −6

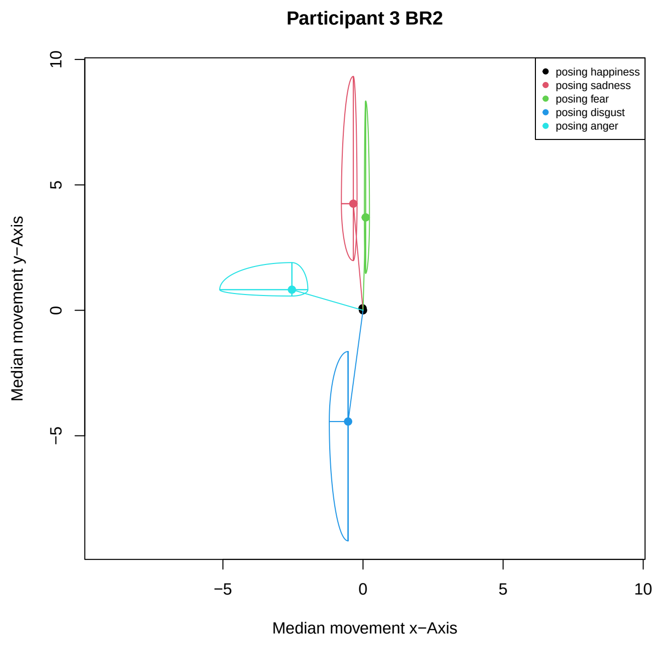
Participant 3 BR2
posing happiness
posing sadness
posing fear
posing disgust
posing anger
Median movement y−Axis
Median movement x−Axis
−5 0 5 10
10 5 0 −5

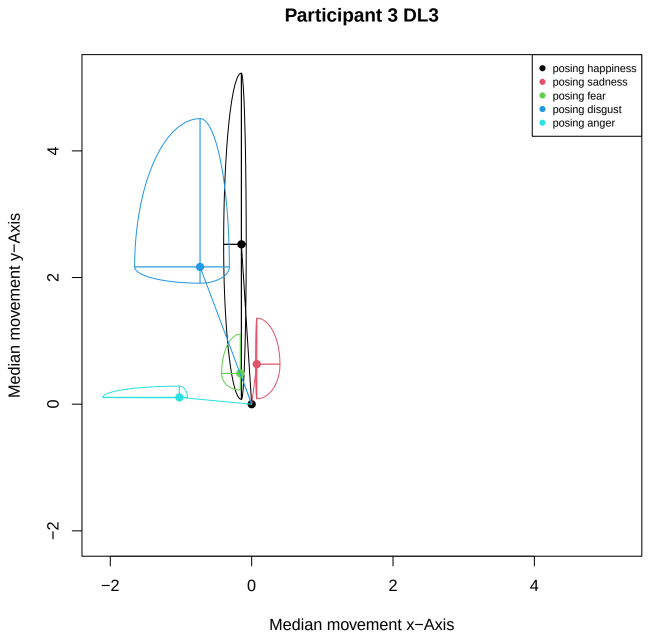
Participant 3 DL3
posing happiness
posing sadness
posing fear
posing disgust
posing anger
Median movement y−Axis
Median movement x−Axis
−2 0 2 4
4 2 0 −2

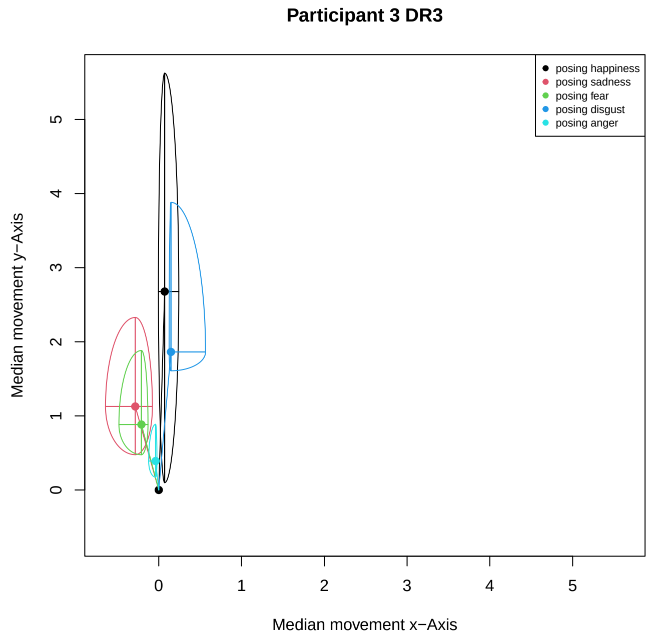
Participant 3 DR3
posing happiness
posing sadness
posing fear
posing disgust
posing anger
Median movement y−Axis
Median movement x−Axis
0 1 2 3 4 5
5 4 3 2 1 0

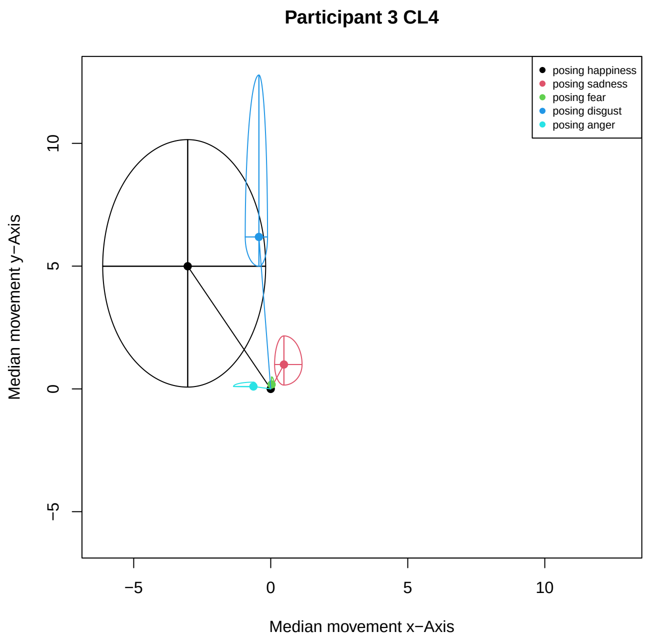
Participant 3 CL4
posing happiness
posing sadness
posing fear
posing disgust
posing anger
Median movement y−Axis
Median movement x−Axis
−5 0 5 10
10 5 0 −5

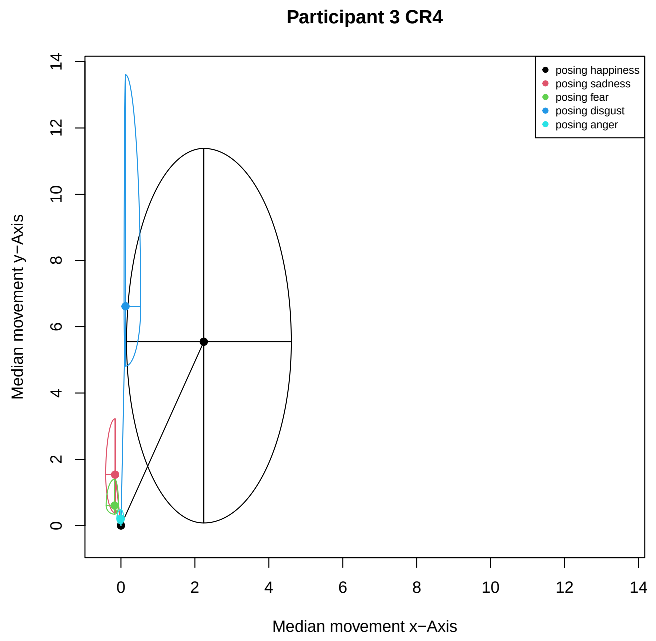
Participant 3 CR4
posing happiness
posing sadness
posing fear
posing disgust
posing anger
Median movement y−Axis
Median movement x−Axis
0 2 4 6 8 10 12 14
14 12 10 8 6 4 2 0

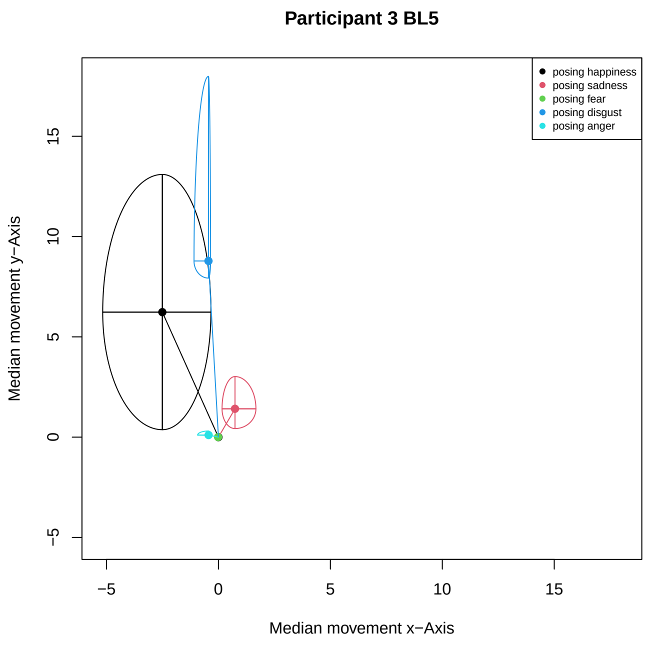
Participant 3 BL5
posing happiness
posing sadness
posing fear
posing disgust
posing anger
Median movement y−Axis
Median movement x−Axis
−5
0
5
10
15

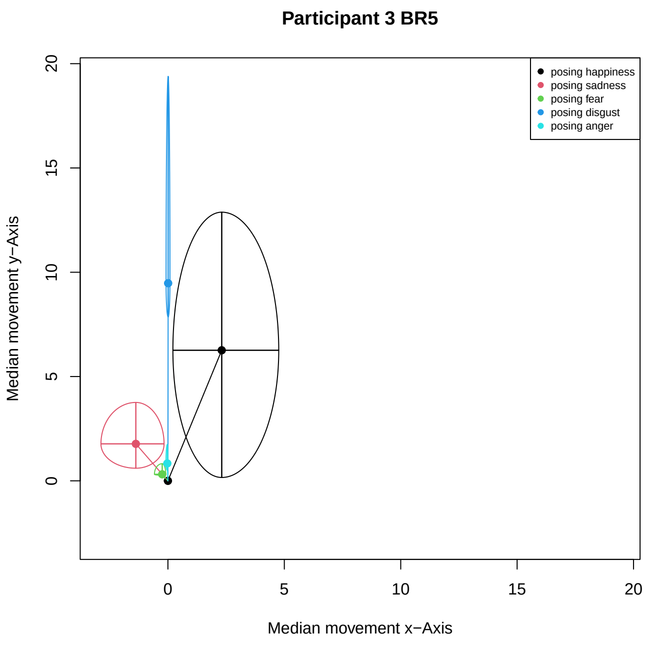
Participant 3 BR5
posing happiness
posing sadness
posing fear
posing disgust
posing anger
Median movement y−Axis
Median movement x−Axis
0
5
10
15
20

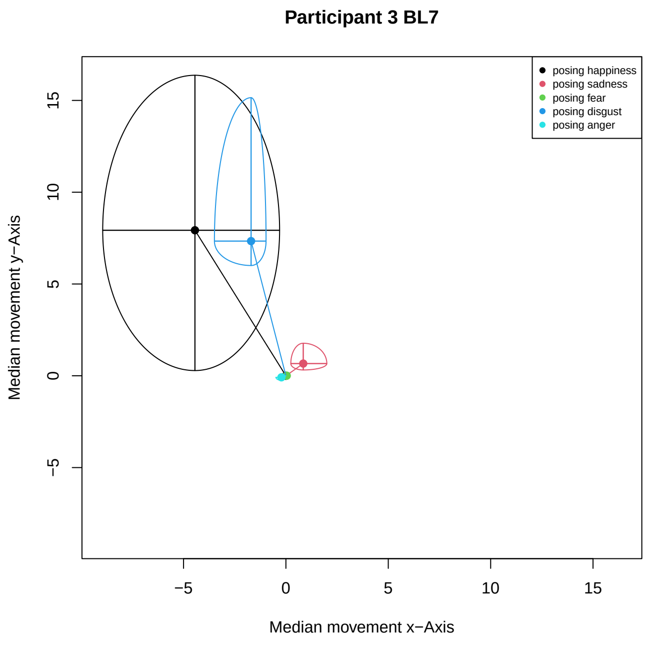
Participant 3 BL7
posing happiness
posing sadness
posing fear
posing disgust
posing anger
Median movement y−Axis
Median movement x−Axis
−5
0
5
10
15

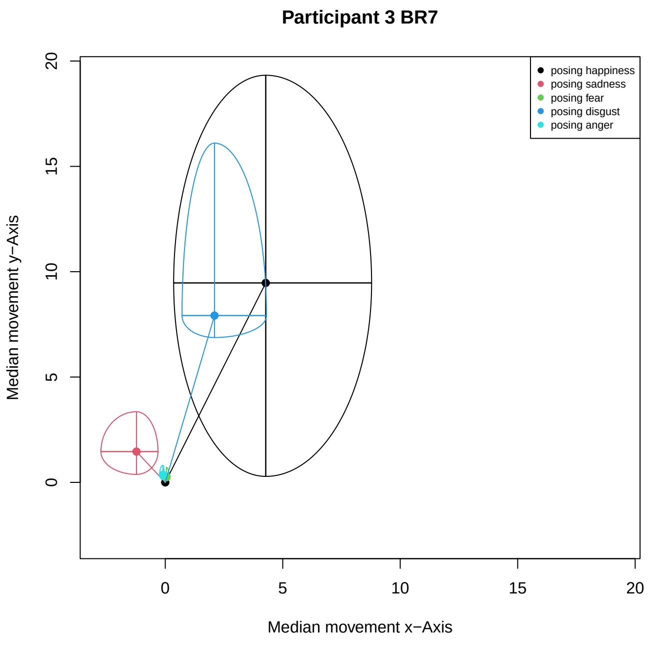
Participant 3 BR7
posing happiness
posing sadness
posing fear
posing disgust
posing anger
Median movement y−Axis
Median movement x−Axis
0
5
10
15
20

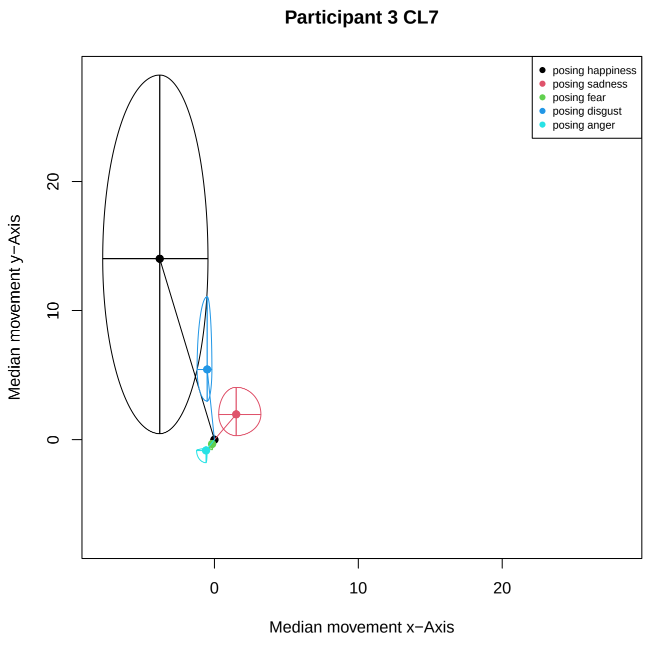
Participant 3 CL7
posing happiness
posing sadness
posing fear
posing disgust
posing anger
Median movement y−Axis
Median movement x−Axis
0
10
20

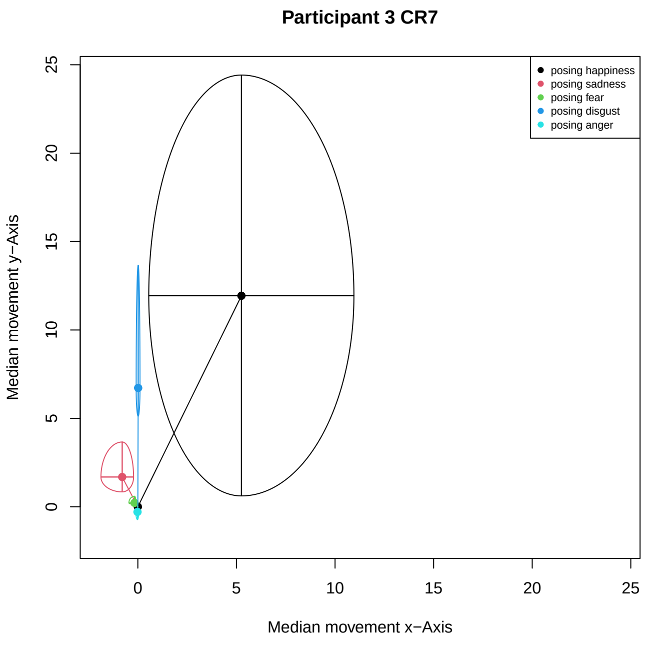
Participant 3 CR7
posing happiness
posing sadness
posing fear
posing disgust
posing anger
Median movement y−Axis
Median movement x−Axis
0
5
10
15
20
25

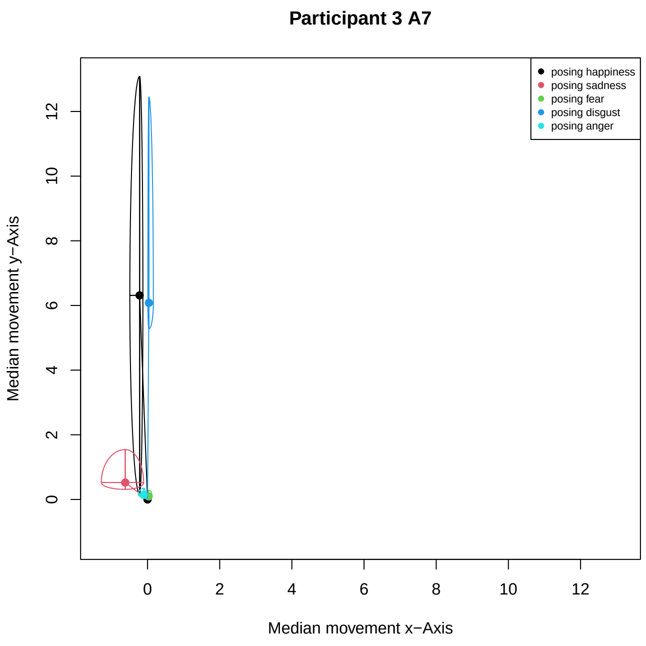

Participant 3 A7
posing happiness
posing sadness
posing fear
posing disgust
posing anger
Median movement y−Axis
Median movement x−Axis
0
2
4
6
8
10
12

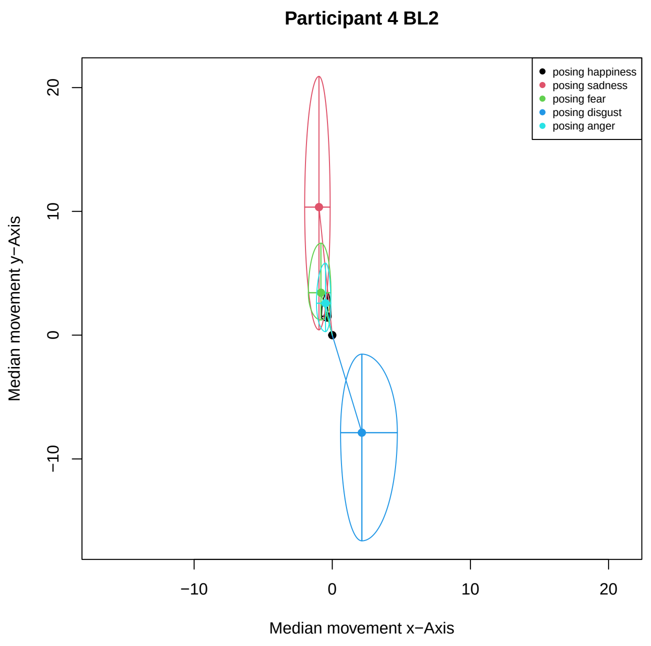
Participant 4 BL2
posing happiness
posing sadness
posing fear
posing disgust
posing anger
Median movement y−Axis
Median movement x−Axis
−10
0
10
20

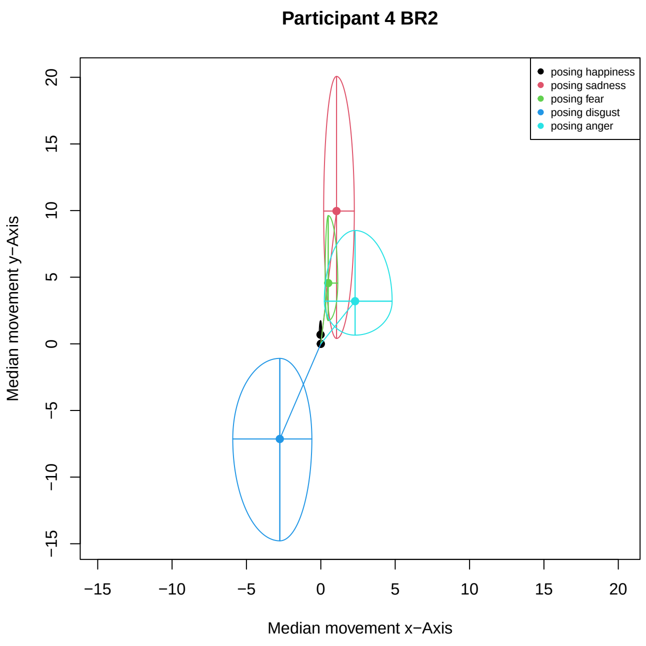
Participant 4 BR2
posing happiness
posing sadness
posing fear
posing disgust
posing anger
Median movement y−Axis
Median movement x−Axis
−15
−10
−5
0
5
10
15
20

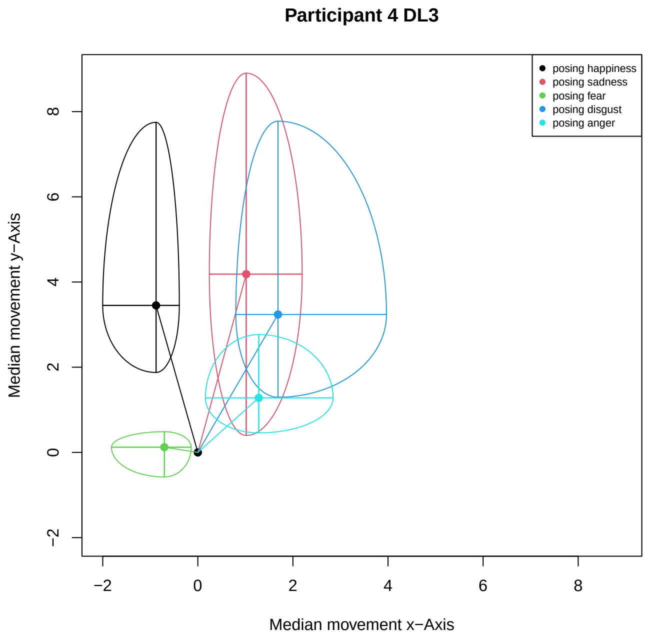
Participant 4 DL3
posing happiness
posing sadness
posing fear
posing disgust
posing anger
Median movement y−Axis
Median movement x−Axis
−2
0
2
4
6
8

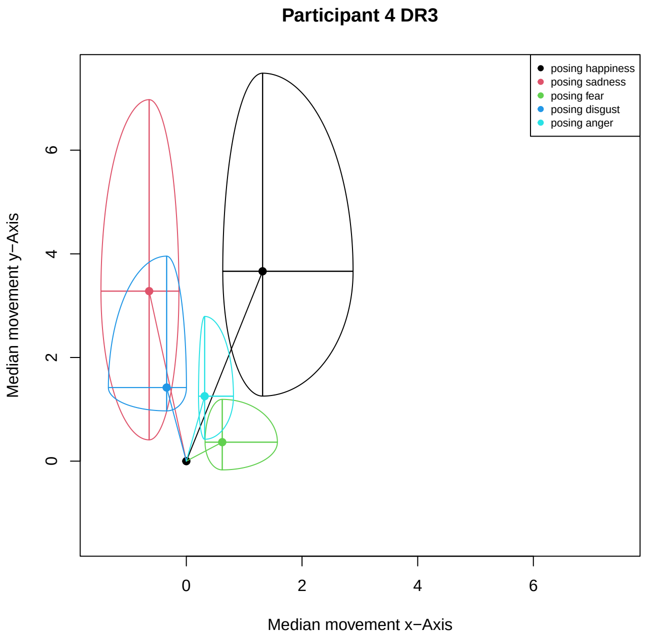
Participant 4 DR3
posing happiness
posing sadness
posing fear
posing disgust
posing anger
Median movement y−Axis
Median movement x−Axis
0
2
4
6

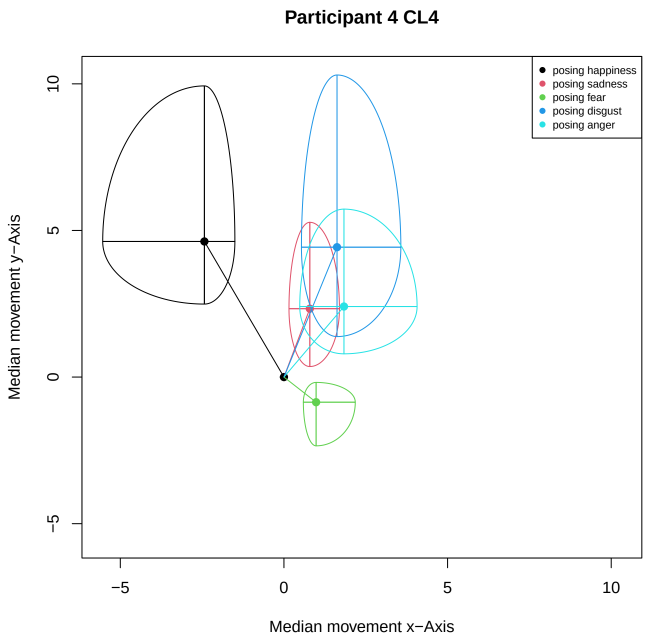
Participant 4 CL4
posing happiness
posing sadness
posing fear
posing disgust
posing anger
Median movement y−Axis
Median movement x−Axis
−5
0
5
10

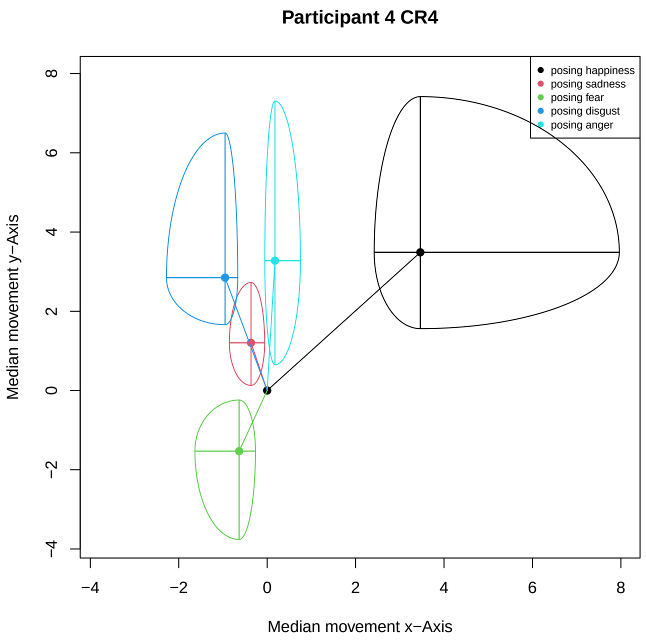
Participant 4 CR4
posing happiness
posing sadness
posing fear
posing disgust
posing anger
Median movement y−Axis
Median movement x−Axis
−4
−2
0
2
4
6
8

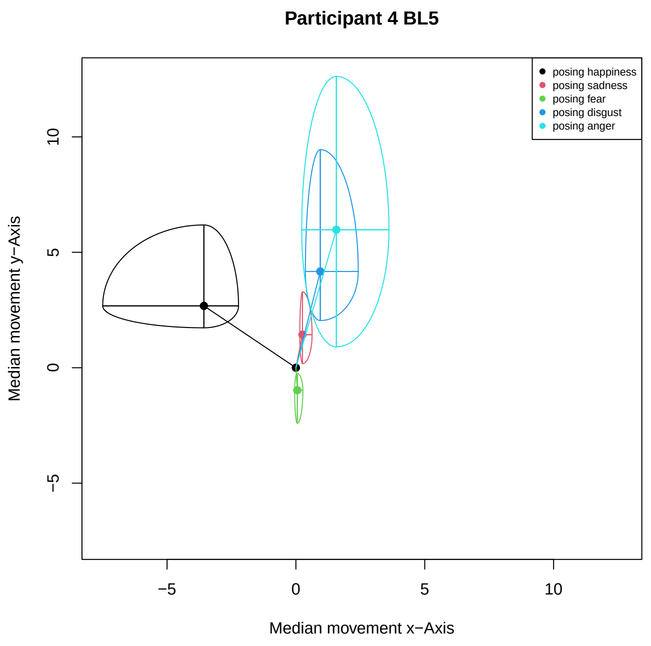
Participant 4 BL5
posing happiness
posing sadness
posing fear
posing disgust
posing anger
Median movement y−Axis
−5
0
5
10
−5
0
5
10
Median movement x−Axis

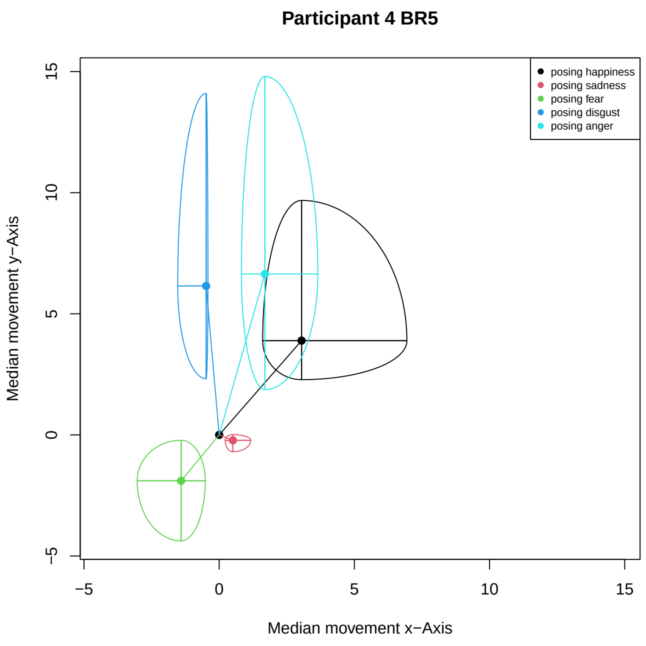
Participant 4 BR5
posing happiness
posing sadness
posing fear
posing disgust
posing anger
Median movement y−Axis
−5
0
5
10
15
−5
0
5
10
15
Median movement x−Axis

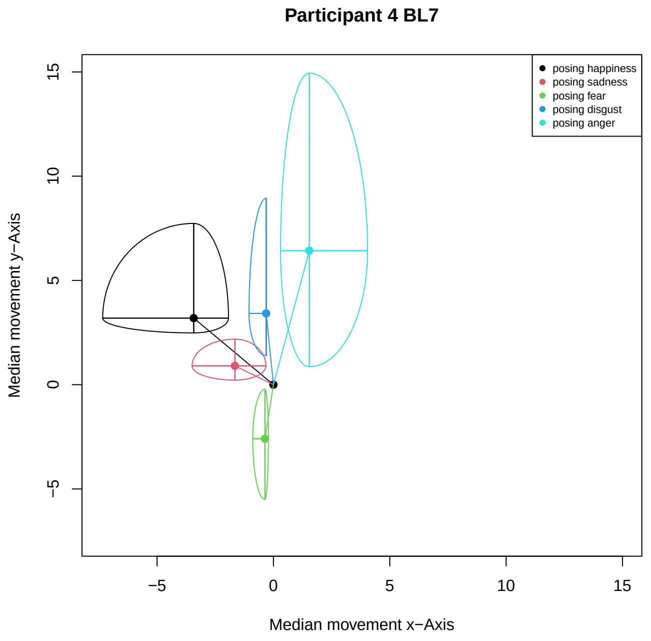
Participant 4 BL7
posing happiness
posing sadness
posing fear
posing disgust
posing anger
Median movement y−Axis
−5
0
5
10
15
−5
0
5
10
15
Median movement x−Axis

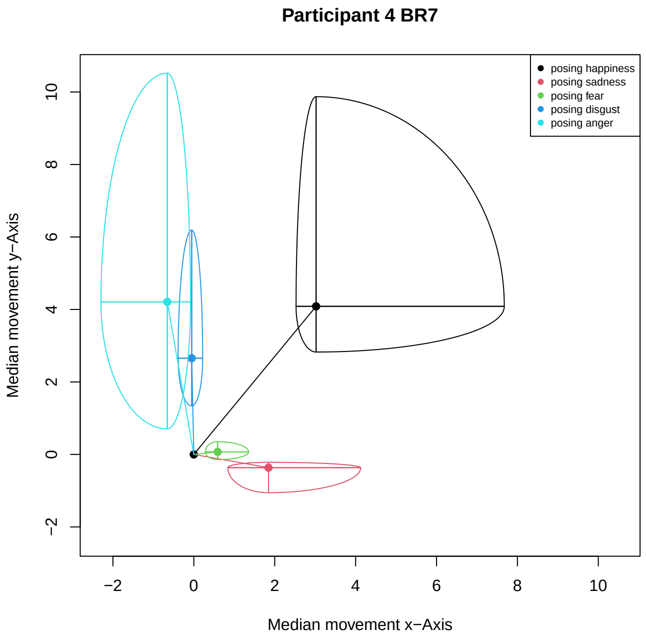
Participant 4 BR7
posing happiness
posing sadness
posing fear
posing disgust
posing anger
Median movement y−Axis
−2
0
2
4
6
8
10
−2
0
2
4
6
8
10
Median movement x−Axis

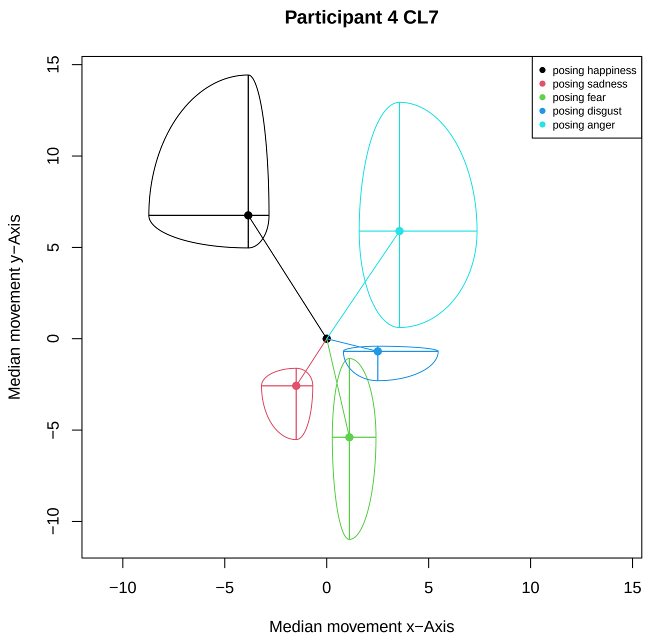
Participant 4 CL7
posing happiness
posing sadness
posing fear
posing disgust
posing anger
Median movement y−Axis
−10
−5
0
5
10
15
−10
−5
0
5
10
15
Median movement x−Axis

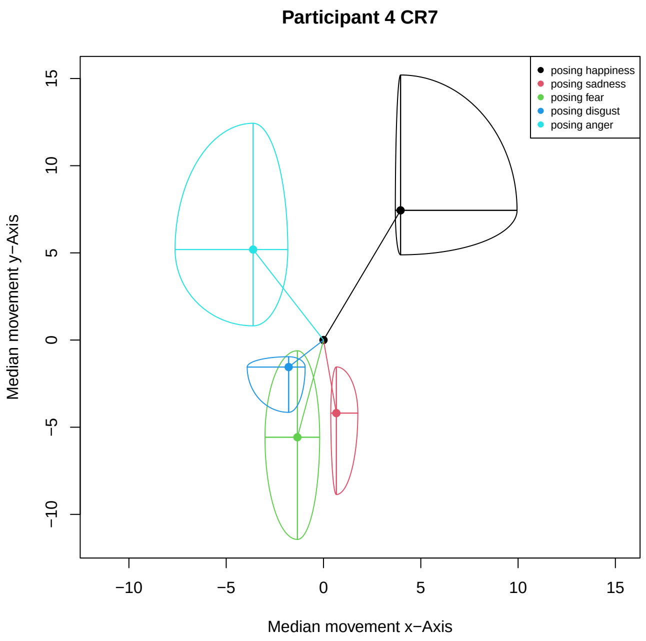
Participant 4 CR7
posing happiness
posing sadness
posing fear
posing disgust
posing anger
Median movement y−Axis
−10
−5
0
5
10
15
−10
−5
0
5
10
15
Median movement x−Axis

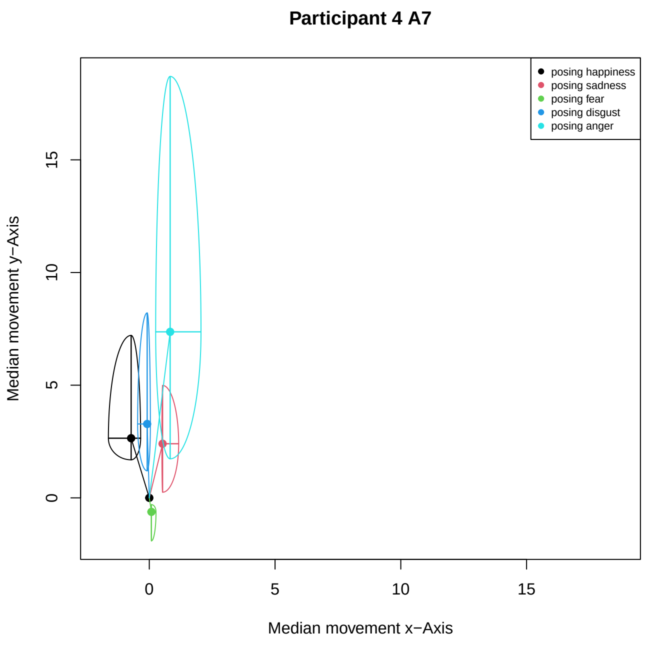

Participant 4 A7
posing happiness
posing sadness
posing fear
posing disgust
posing anger
Median movement y−Axis
Median movement x−Axis
0
5
10
15

Participant 6 BL2

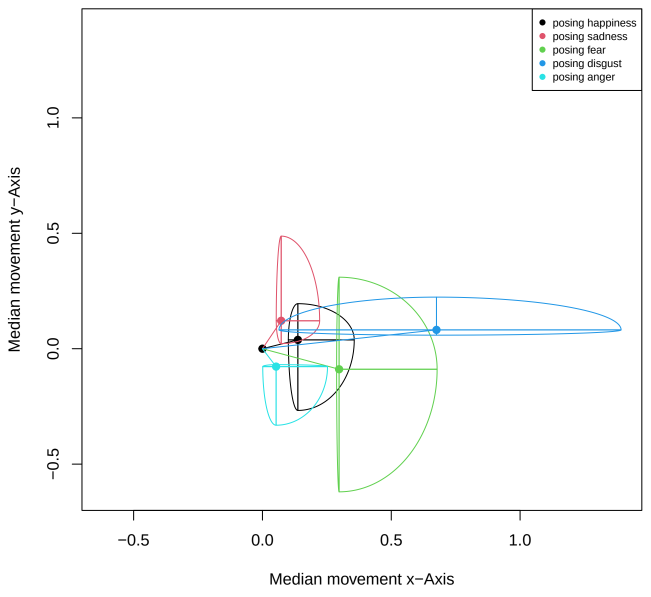

Participant 6 BR2

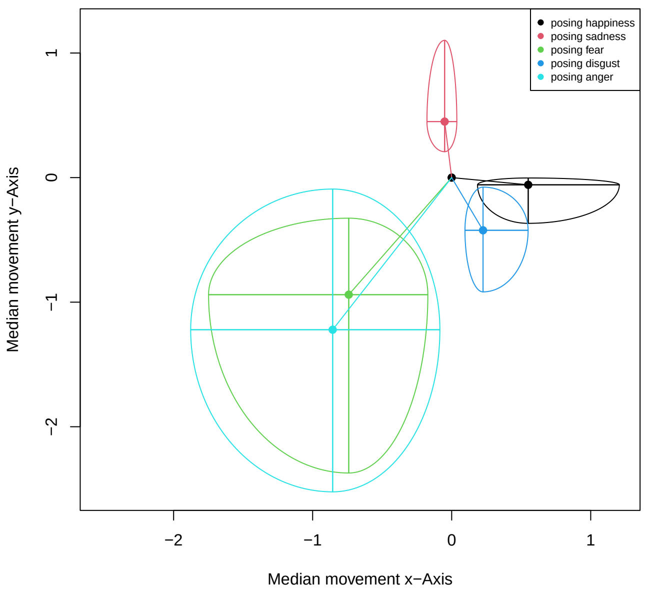

Participant 6 DL3

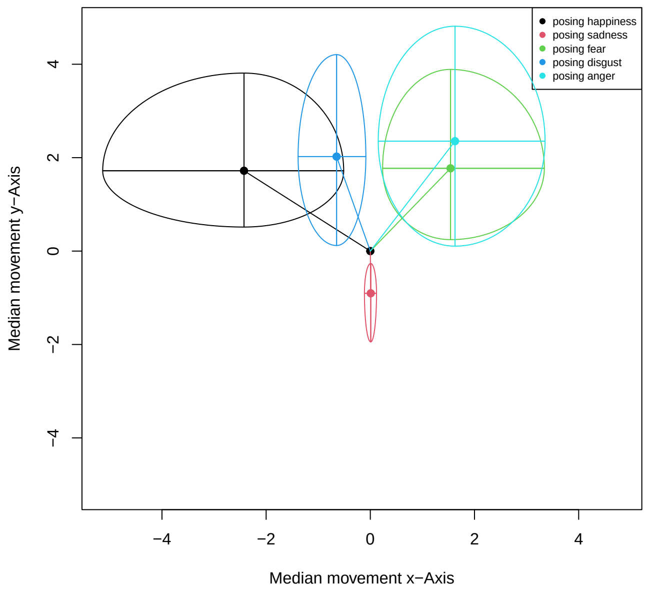

Participant 6 DR3

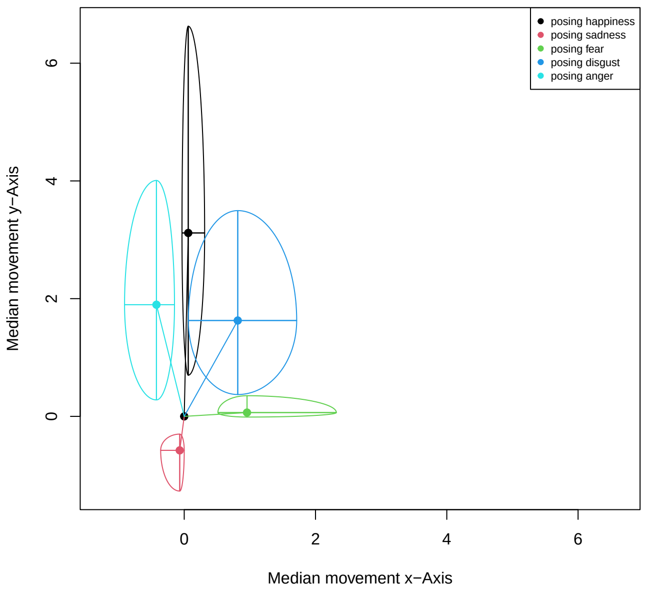

Participant 6 CL4

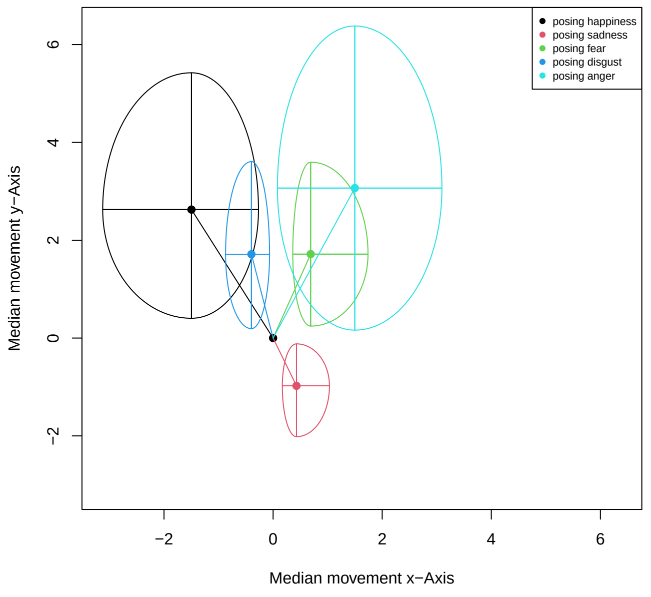

Participant 6 CR4

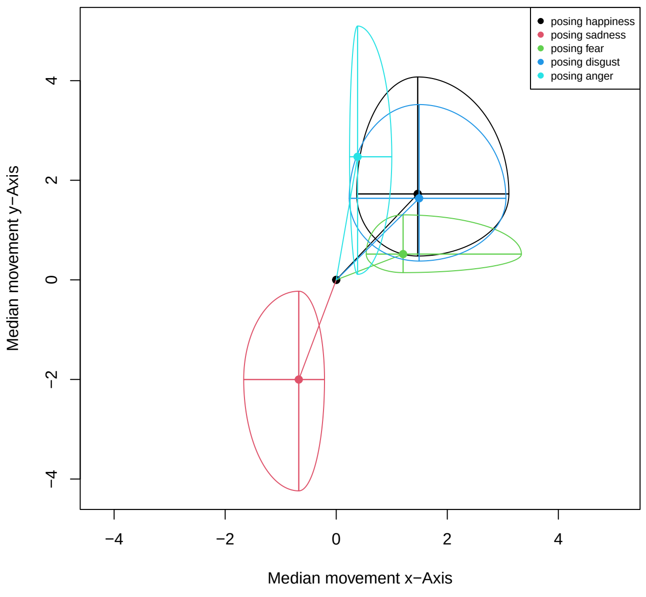

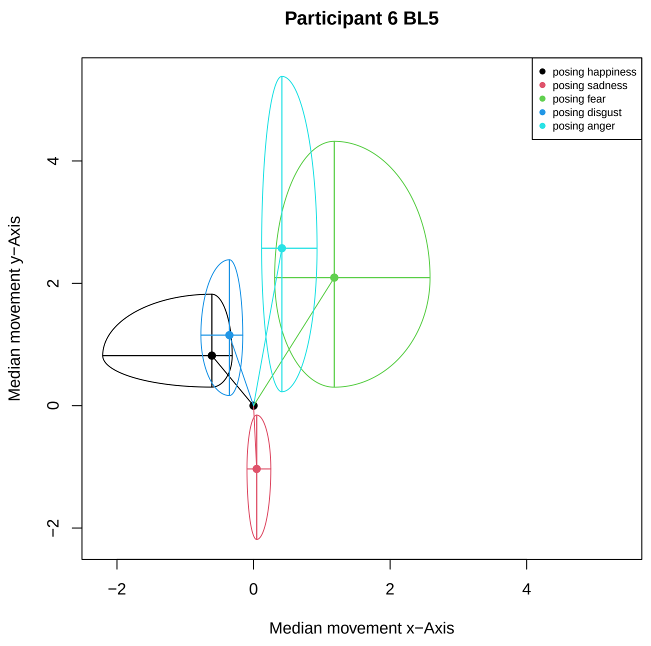
Participant 6 BL5
posing happiness
posing sadness
posing fear
posing disgust
posing anger
Median movement x−Axis
Median movement y−Axis

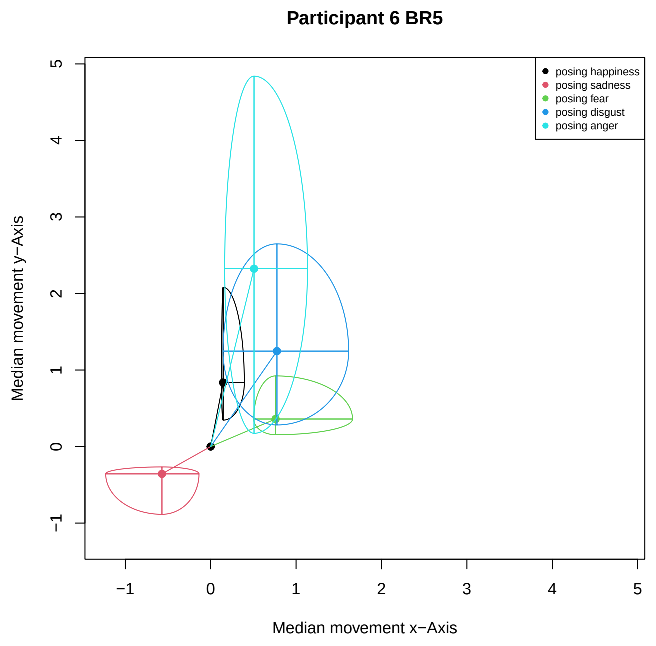
Participant 6 BR5
posing happiness
posing sadness
posing fear
posing disgust
posing anger
Median movement x−Axis
Median movement y−Axis

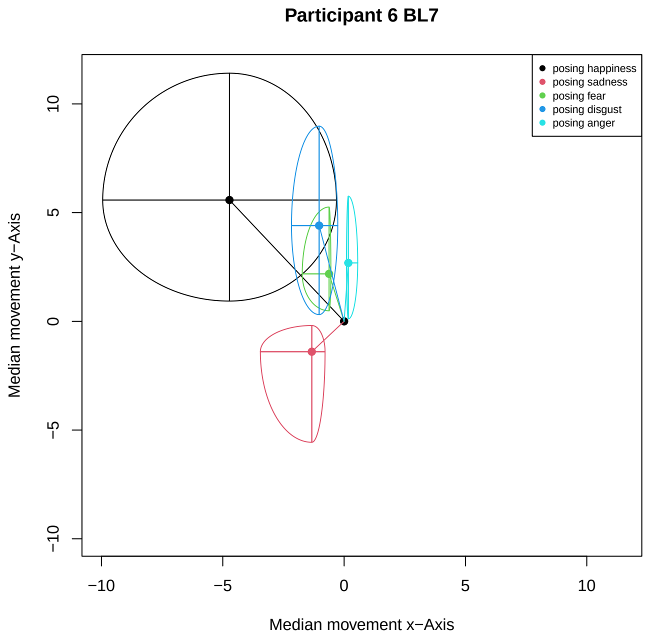
Participant 6 BL7
posing happiness
posing sadness
posing fear
posing disgust
posing anger
Median movement x−Axis
Median movement y−Axis

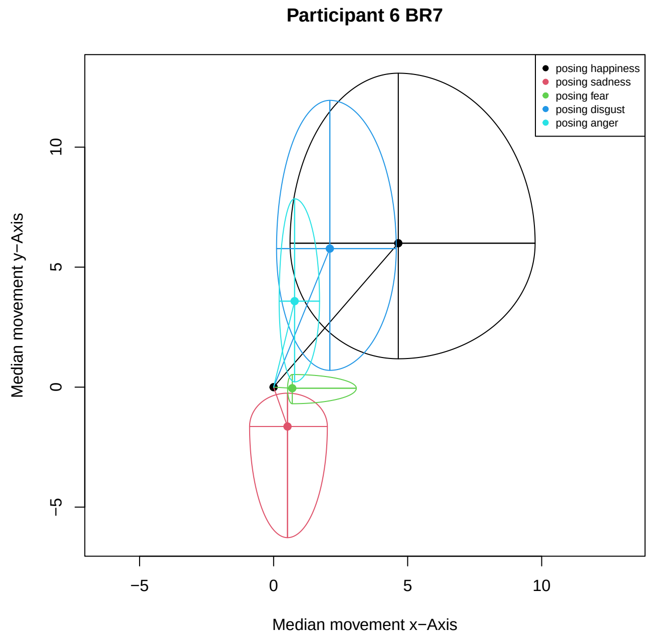
Participant 6 BR7
posing happiness
posing sadness
posing fear
posing disgust
posing anger
Median movement x−Axis
Median movement y−Axis

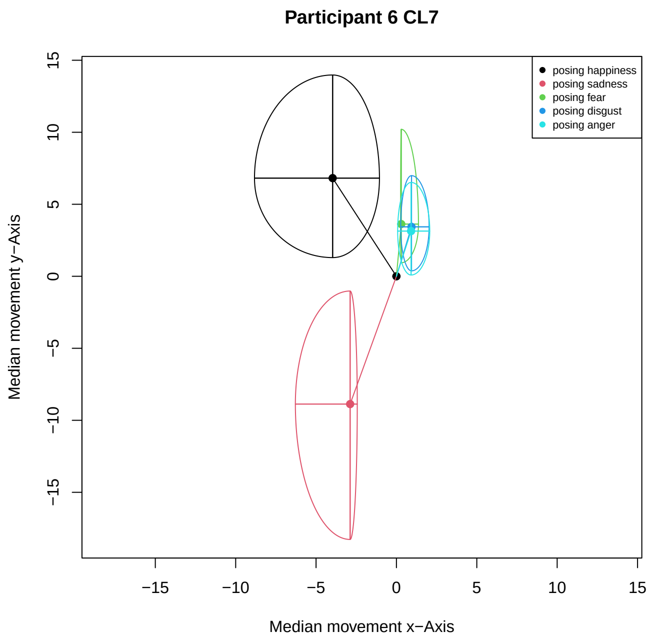
Participant 6 CL7
posing happiness
posing sadness
posing fear
posing disgust
posing anger
Median movement x−Axis
Median movement y−Axis

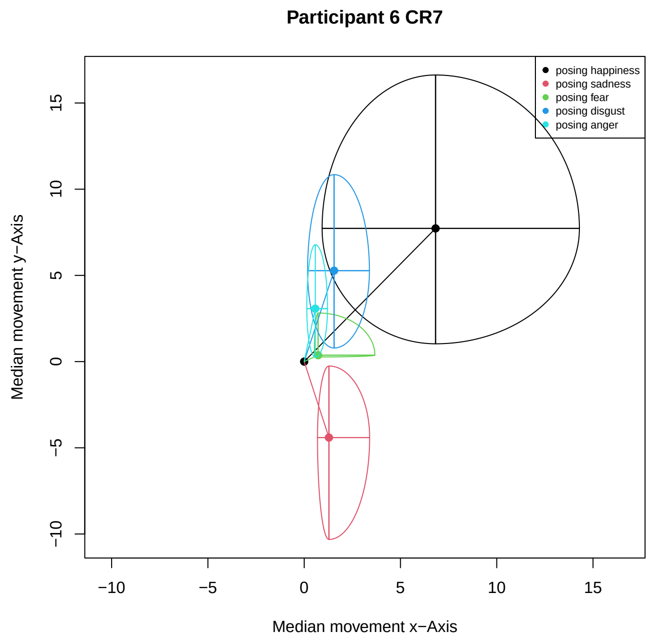
Participant 6 CR7
posing happiness
posing sadness
posing fear
posing disgust
posing anger
Median movement x−Axis
Median movement y−Axis

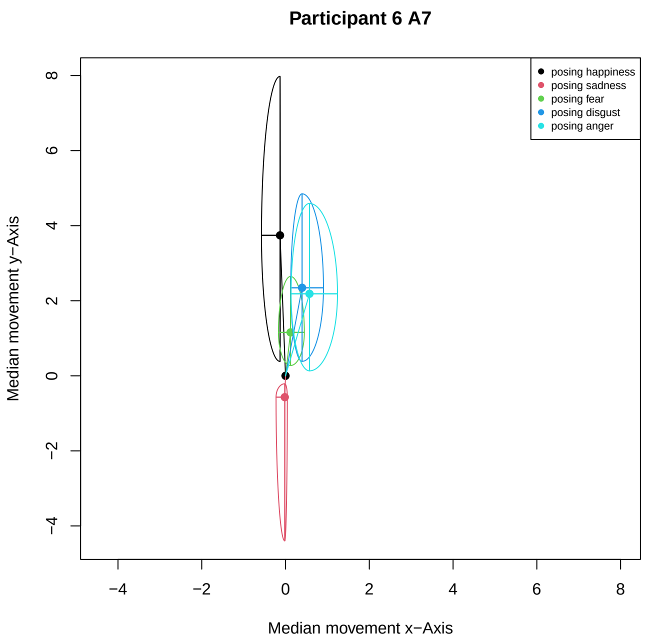
Participant 6 A7
posing happiness
posing sadness
posing fear
posing disgust
posing anger
Median movement y−Axis
Median movement x−Axis
8
6
4
2
0
−2
−4
−4
−2
0
2
4
6
8

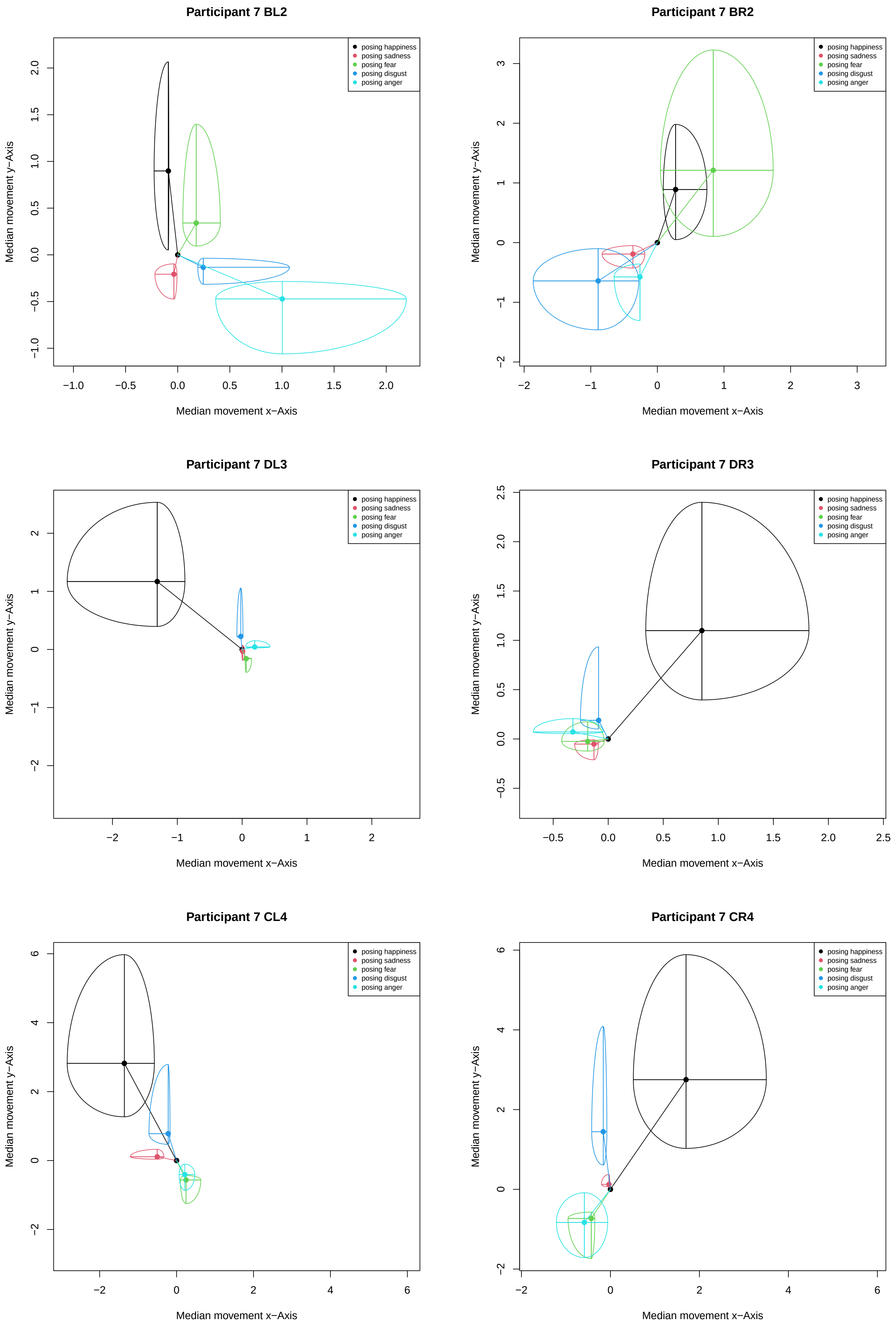
Participant 7 BL2
Participant 7 BR2
Participant 7 DL3
Participant 7 DR3
Participant 7 CL4
Participant 7 CR4
posing happiness
posing sadness
posing fear
posing disgust
posing anger
Median movement x−Axis
Median movement y−Axis

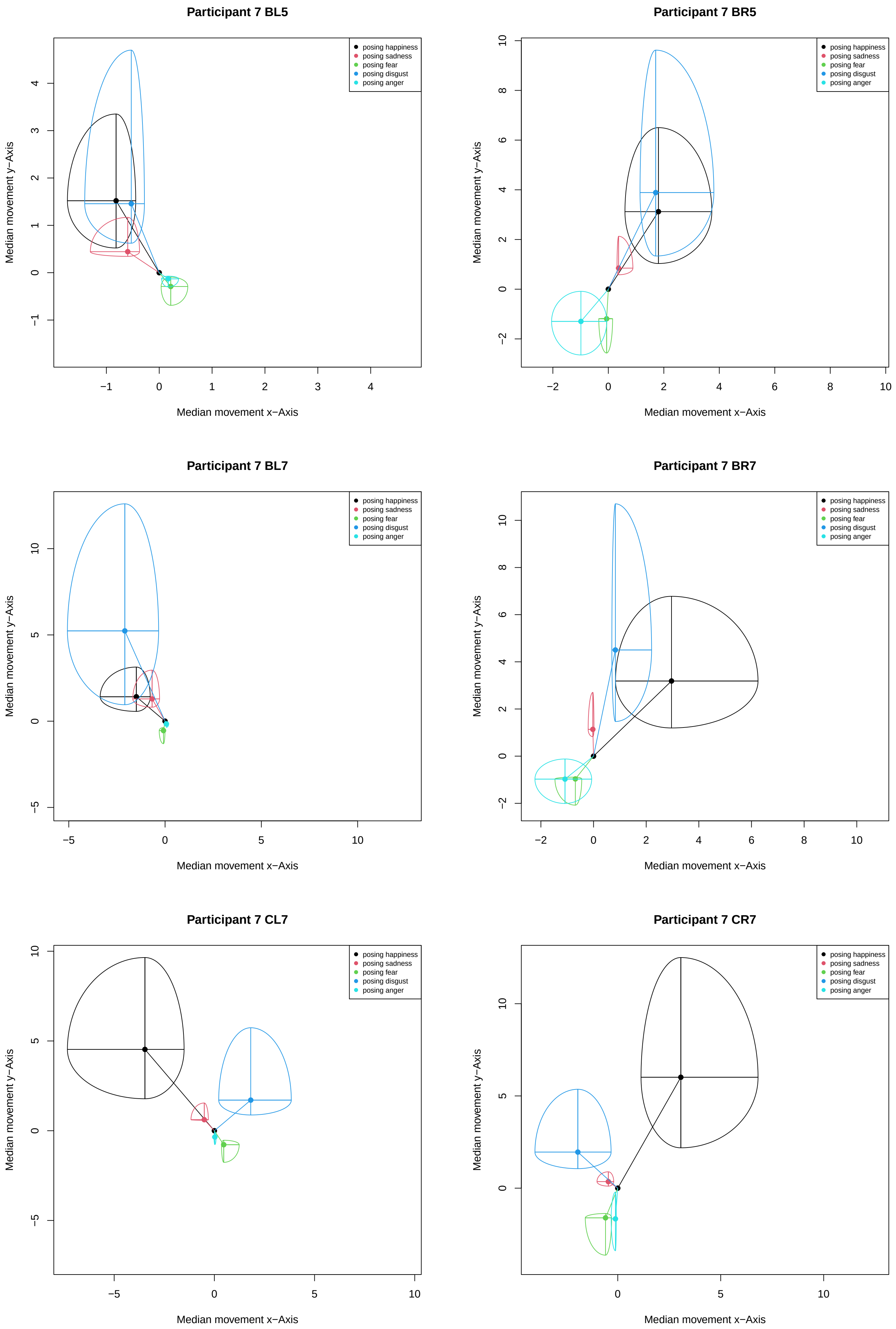

Participant 7 BL5
Participant 7 BR5
Participant 7 BL7
Participant 7 BR7
Participant 7 CL7
Participant 7 CR7
posing happiness
posing sadness
posing fear
posing disgust
posing anger
Median movement x−Axis
Median movement y−Axis

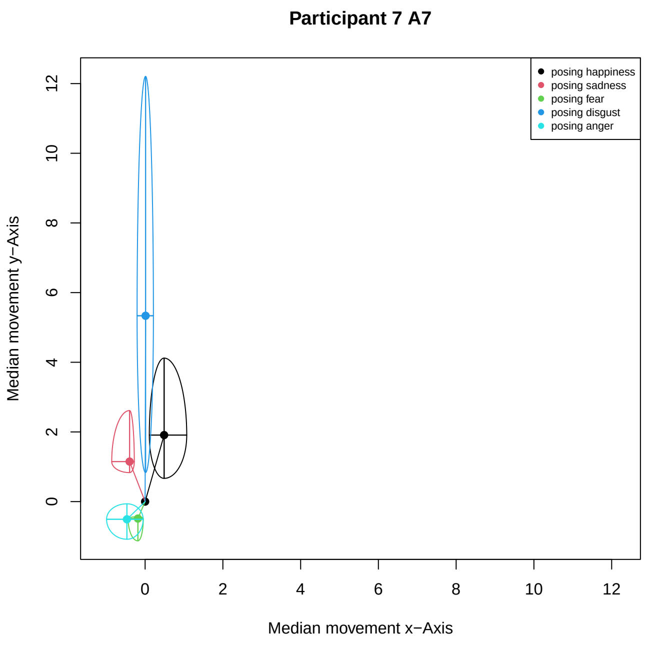

Participant 7 A7
posing happiness
posing sadness
posing fear
posing disgust
posing anger
Median movement y−Axis
Median movement x−Axis
0
2
4
6
8
10
12

Participant 8 BL2

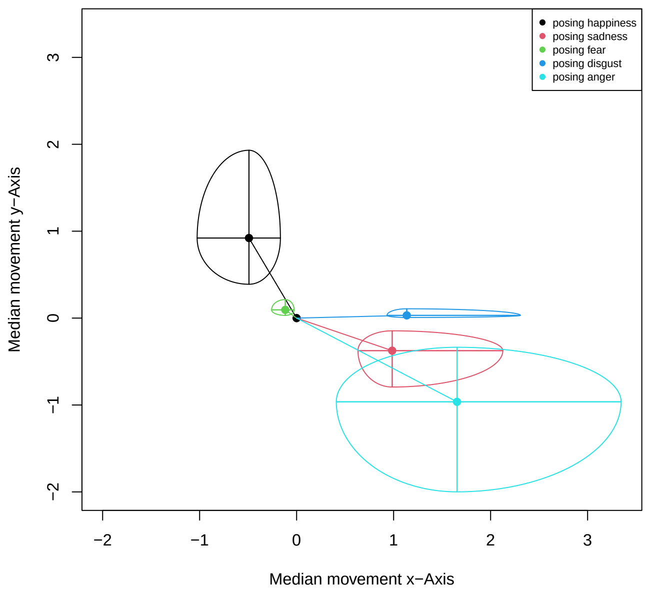

Participant 8 BR2

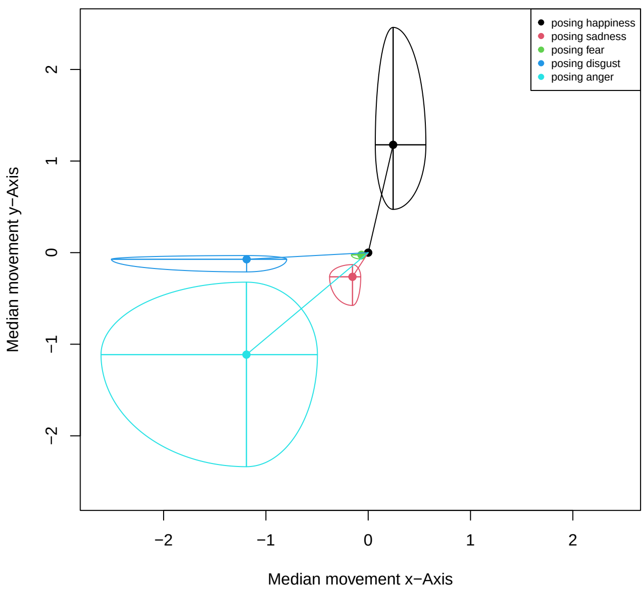

Participant 8 DL3

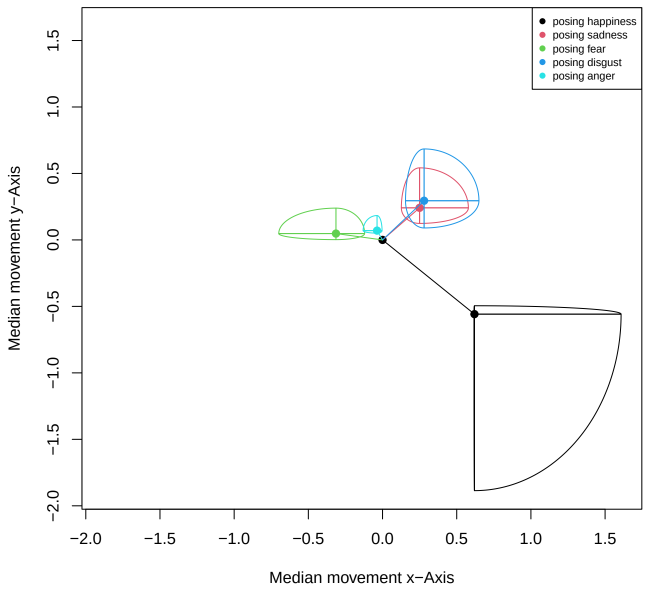

Participant 8 DR3

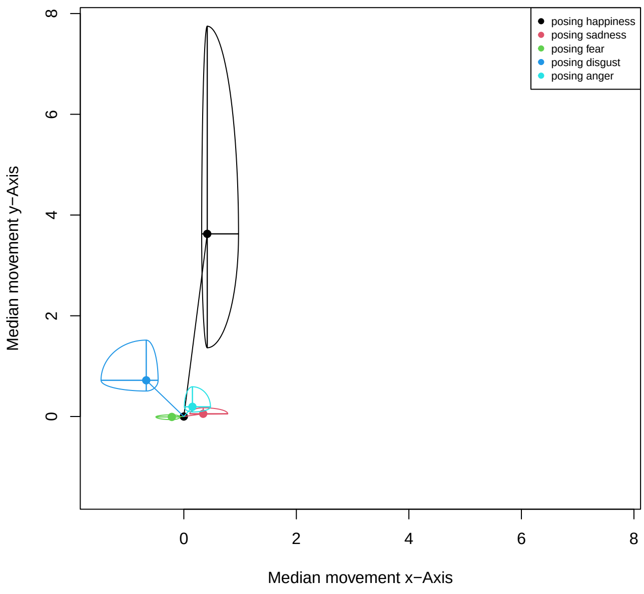

Participant 8 CL4

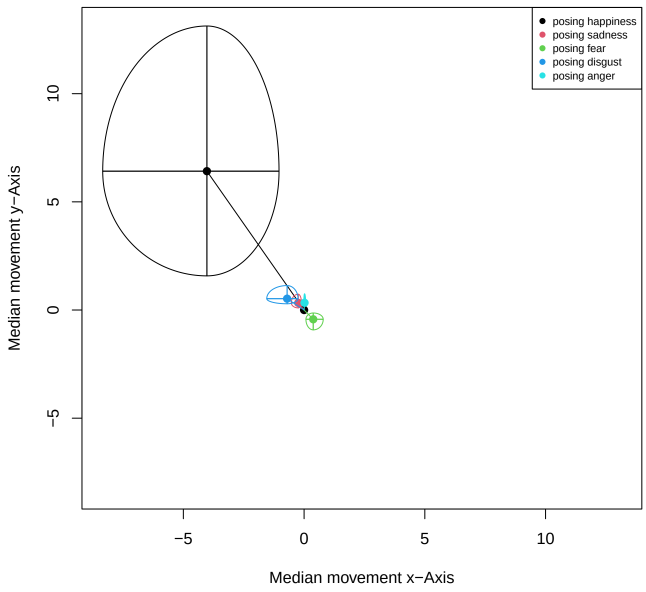

Participant 8 CR4

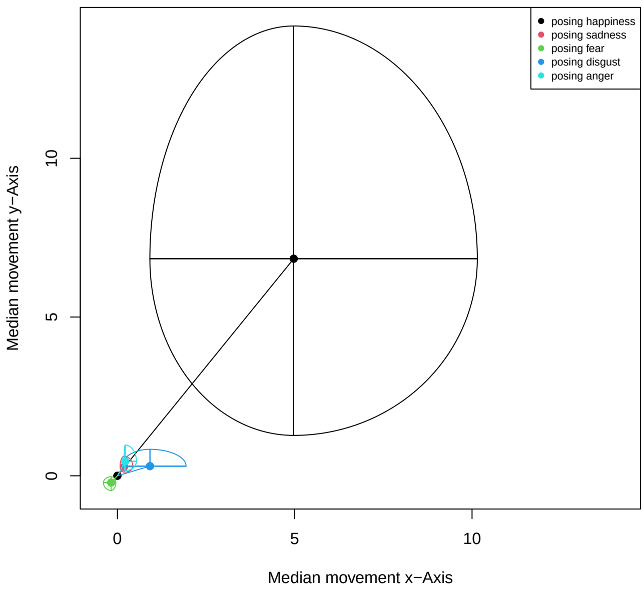

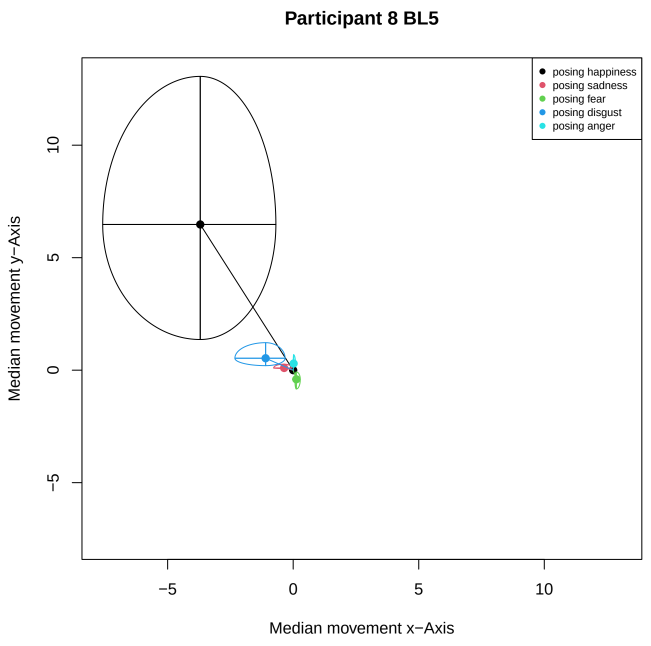
Participant 8 BL5
posing happiness
posing sadness
posing fear
posing disgust
posing anger
Median movement x−Axis
Median movement y−Axis

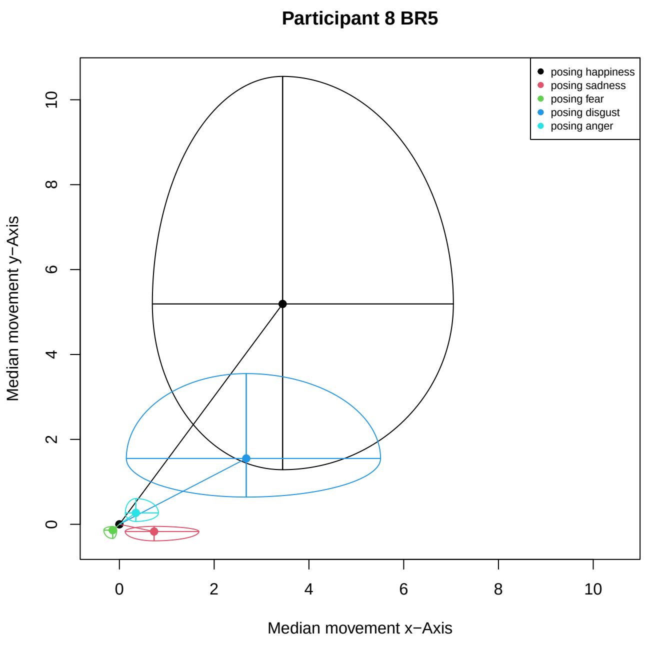
Participant 8 BR5
posing happiness
posing sadness
posing fear
posing disgust
posing anger
Median movement x−Axis
Median movement y−Axis

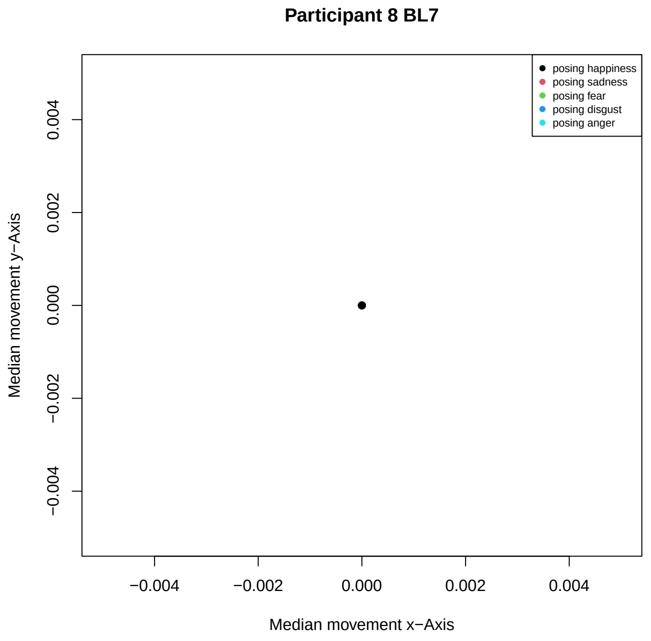
Participant 8 BL7
posing happiness
posing sadness
posing fear
posing disgust
posing anger
Median movement x−Axis
Median movement y−Axis

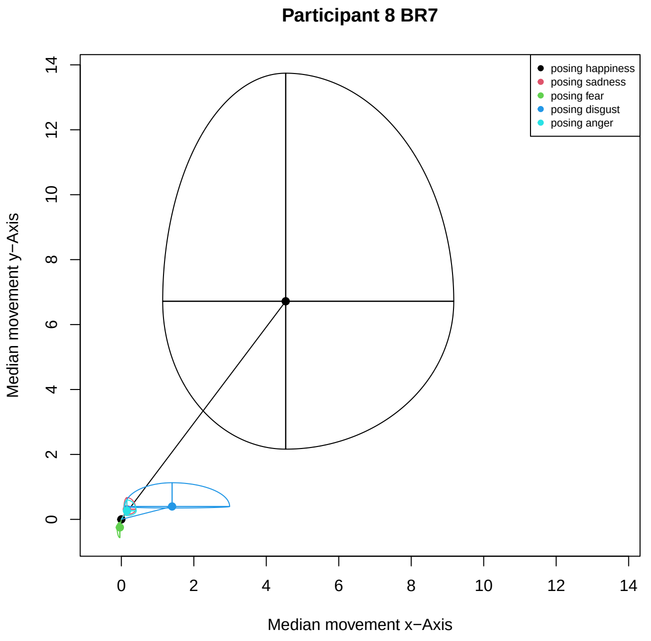
Participant 8 BR7
posing happiness
posing sadness
posing fear
posing disgust
posing anger
Median movement x−Axis
Median movement y−Axis

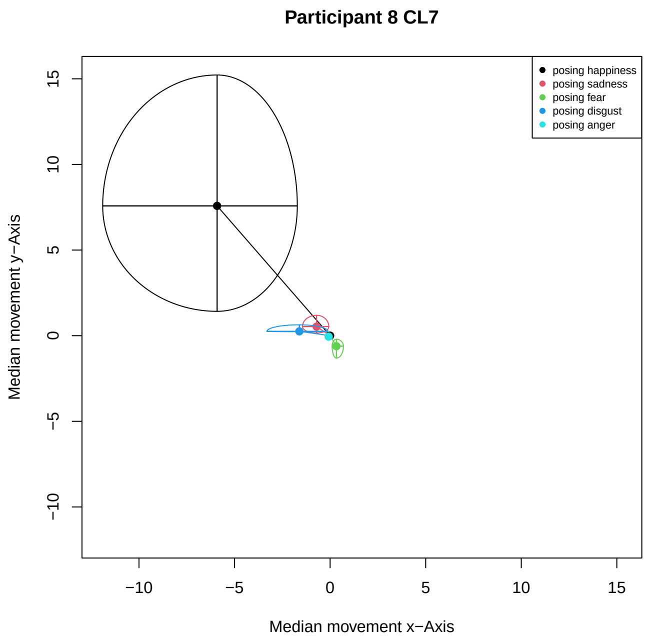
Participant 8 CL7
posing happiness
posing sadness
posing fear
posing disgust
posing anger
Median movement x−Axis
Median movement y−Axis

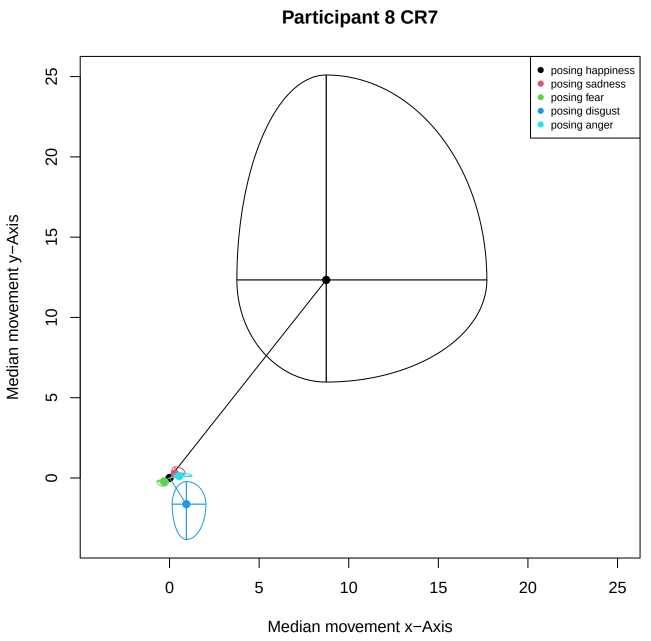
Participant 8 CR7
posing happiness
posing sadness
posing fear
posing disgust
posing anger
Median movement x−Axis
Median movement y−Axis

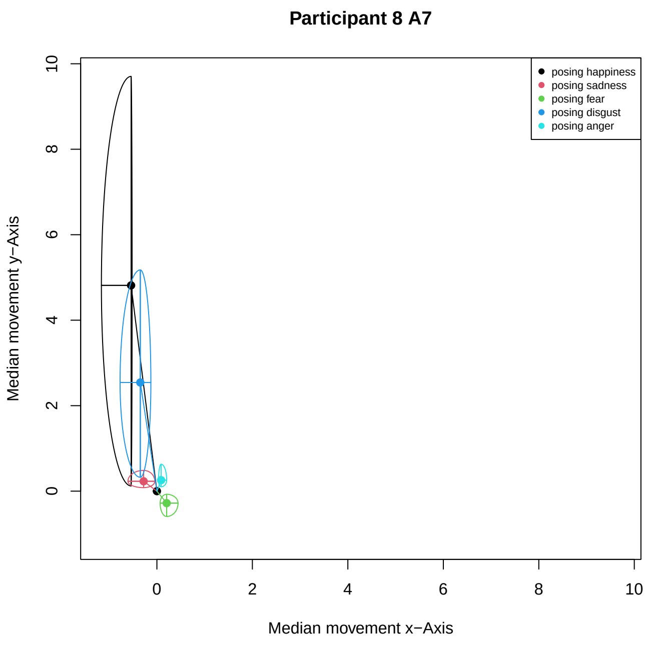

Participant 8 A7
posing happiness
posing sadness
posing fear
posing disgust
posing anger
Median movement y−Axis
Median movement x−Axis
0
2
4
6
8
10

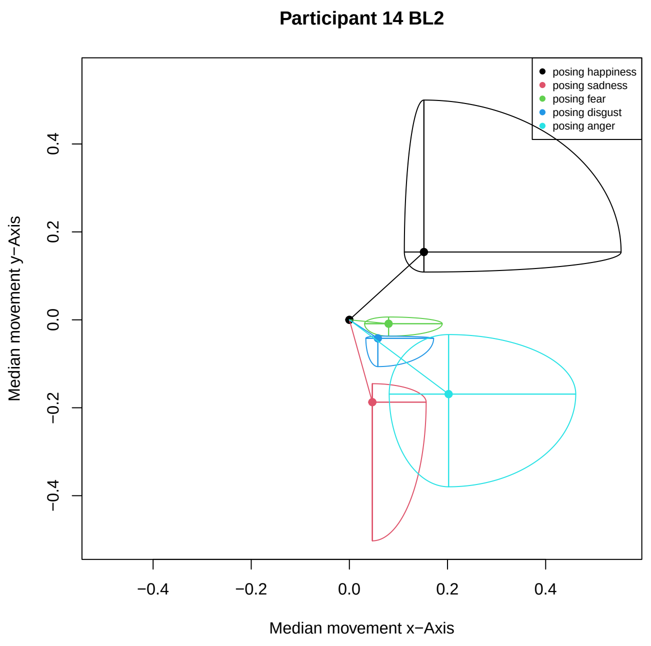
Participant 14 BL2
posing happiness
posing sadness
posing fear
posing disgust
posing anger
Median movement y−Axis
Median movement x−Axis
0.4
0.2
0.0
−0.2
−0.4
−0.4
−0.2
0.0
0.2
0.4

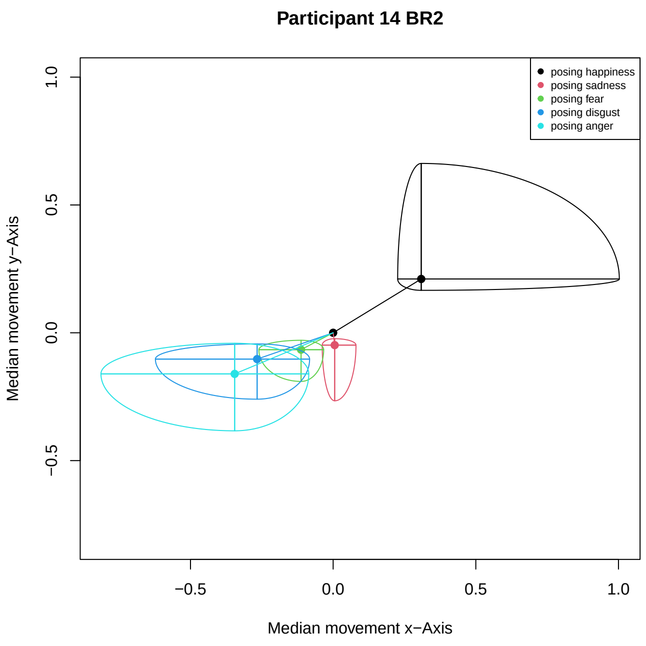
Participant 14 BR2
posing happiness
posing sadness
posing fear
posing disgust
posing anger
Median movement y−Axis
Median movement x−Axis
1.0
0.5
0.0
−0.5
−0.5
0.0
0.5
1.0

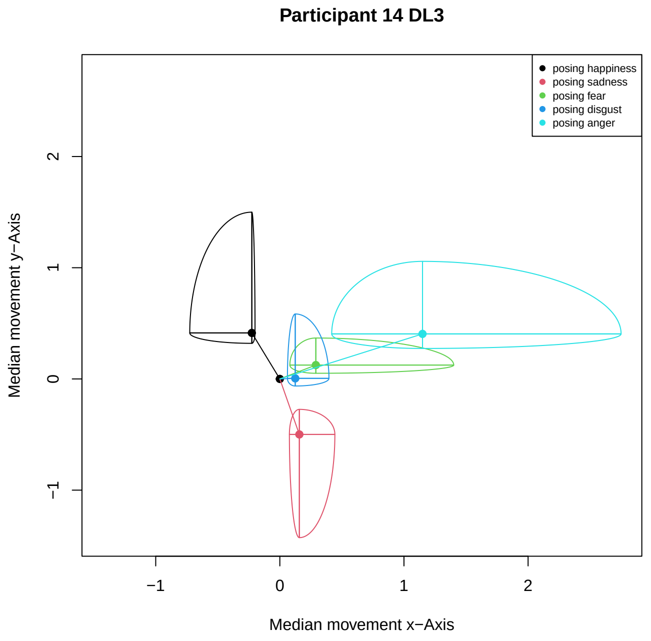
Participant 14 DL3
posing happiness
posing sadness
posing fear
posing disgust
posing anger
Median movement y−Axis
Median movement x−Axis
2
1
0
−1
−1
0
1
2

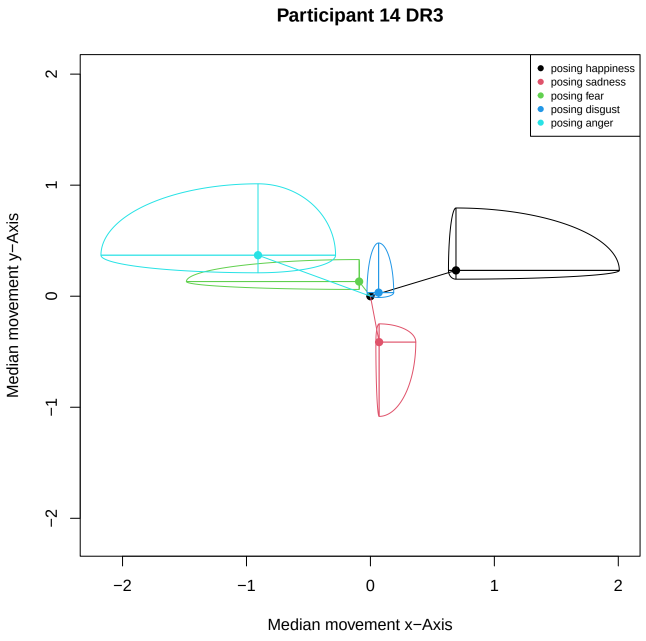
Participant 14 DR3
posing happiness
posing sadness
posing fear
posing disgust
posing anger
Median movement y−Axis
Median movement x−Axis
2
1
0
−1
−2
−2
−1
0
1
2

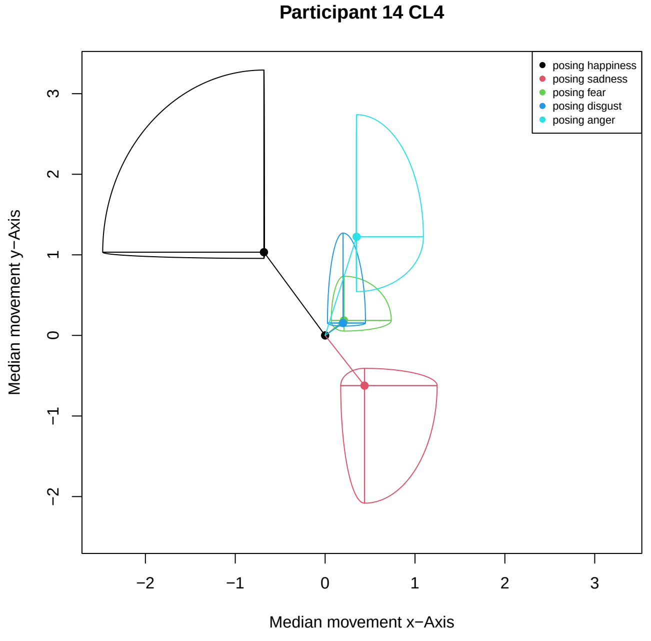
Participant 14 CL4
posing happiness
posing sadness
posing fear
posing disgust
posing anger
Median movement y−Axis
Median movement x−Axis
3
2
1
0
−1
−2
−2
−1
0
1
2
3

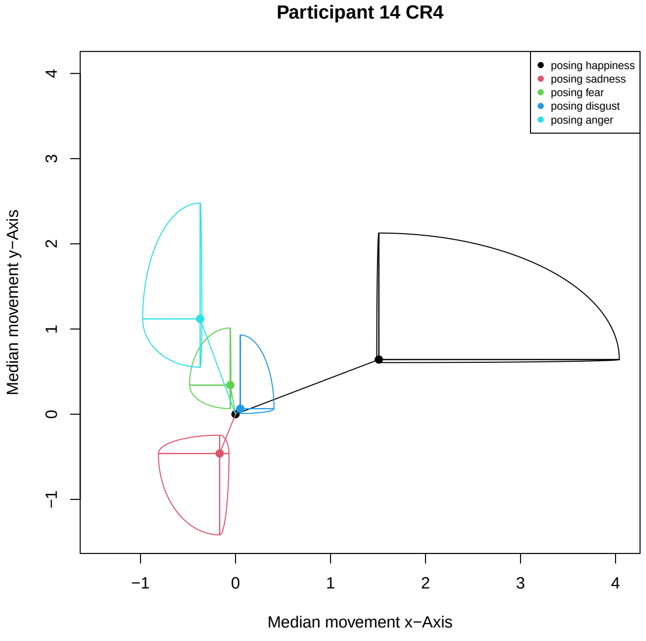
Participant 14 CR4
posing happiness
posing sadness
posing fear
posing disgust
posing anger
Median movement y−Axis
Median movement x−Axis
4
3
2
1
0
−1
−1
0
1
2
3
4

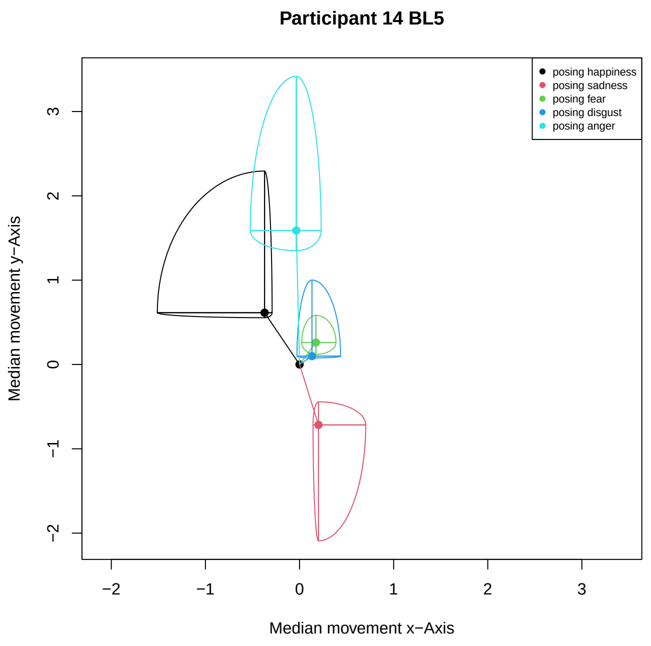
Participant 14 BL5
posing happiness
posing sadness
posing fear
posing disgust
posing anger
Median movement y−Axis
Median movement x−Axis
3
2
1
0
−1
−2
−2
−1
0
1
2
3

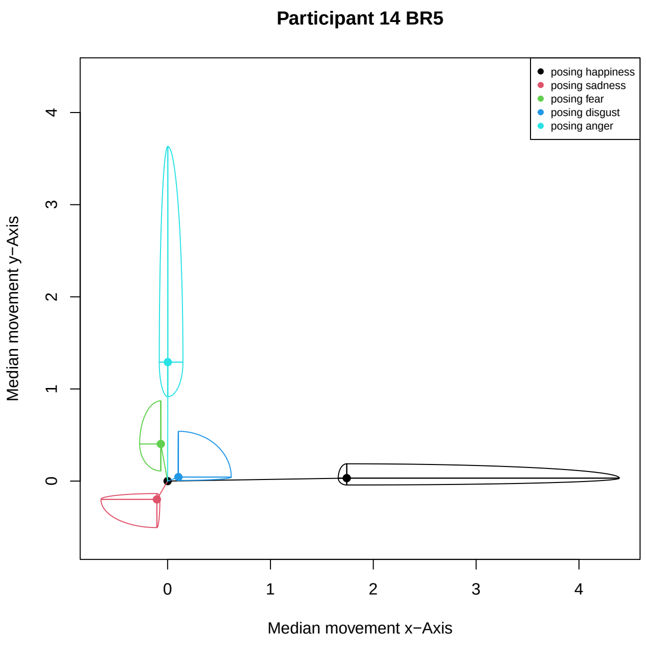
Participant 14 BR5
posing happiness
posing sadness
posing fear
posing disgust
posing anger
Median movement y−Axis
Median movement x−Axis
4
3
2
1
0
0
1
2
3
4

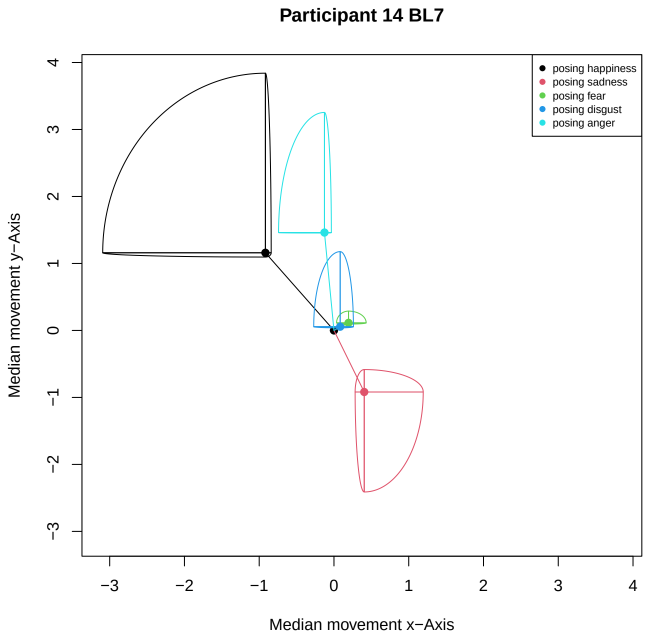
Participant 14 BL7
posing happiness
posing sadness
posing fear
posing disgust
posing anger
Median movement y−Axis
Median movement x−Axis
4
3
2
1
0
−1
−2
−3
−3
−2
−1
0
1
2
3
4

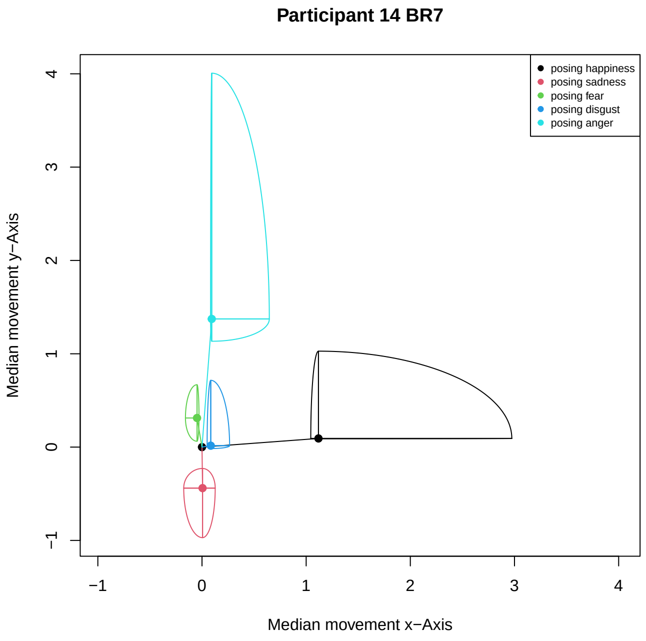
Participant 14 BR7
posing happiness
posing sadness
posing fear
posing disgust
posing anger
Median movement y−Axis
Median movement x−Axis
4
3
2
1
0
−1
−1
0
1
2
3
4

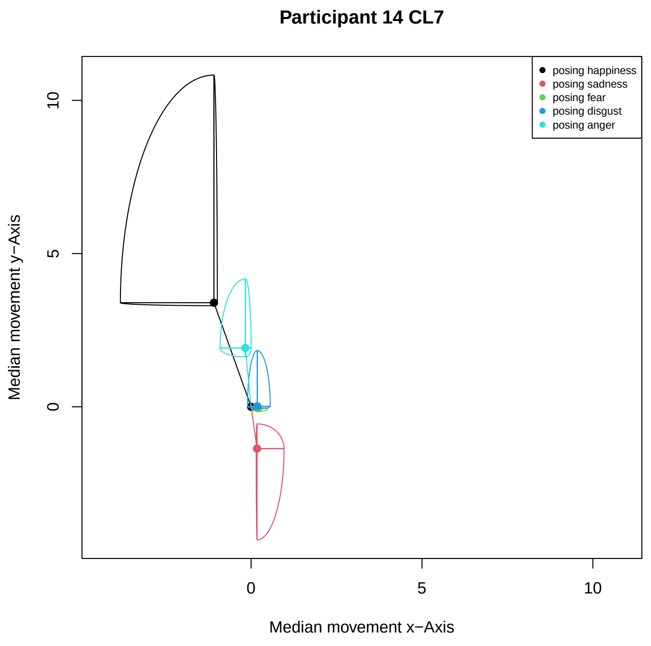
Participant 14 CL7
posing happiness
posing sadness
posing fear
posing disgust
posing anger
Median movement y−Axis
Median movement x−Axis
10
5
0
0
5
10

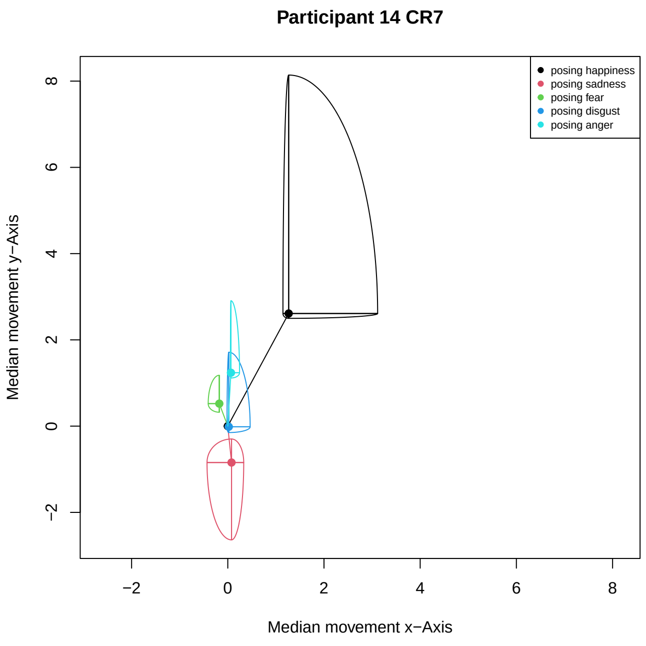
Participant 14 CR7
posing happiness
posing sadness
posing fear
posing disgust
posing anger
Median movement y−Axis
Median movement x−Axis
8
6
4
2
0
−2
−2
0
2
4
6
8

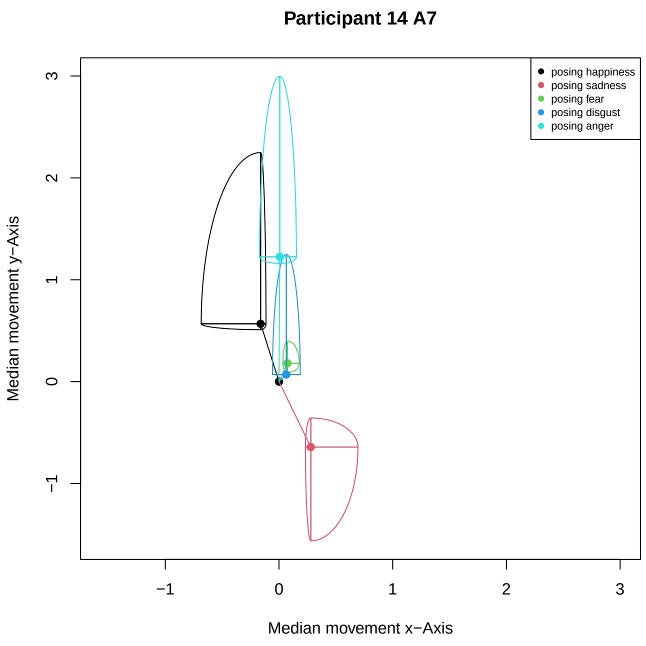

Participant 14 A7
posing happiness
posing sadness
posing fear
posing disgust
posing anger
Median movement x−Axis
Median movement y−Axis
−1
0
1
2
3

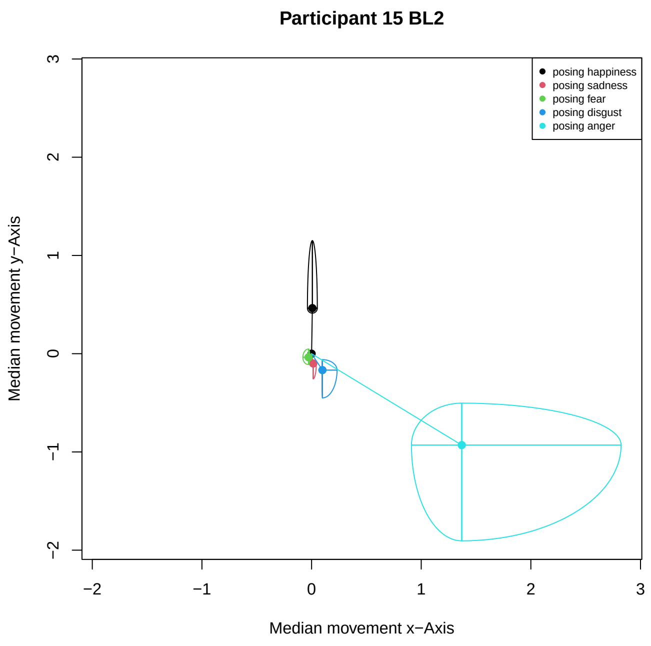
Participant 15 BL2
posing happiness
posing sadness
posing fear
posing disgust
posing anger
Median movement y−Axis
Median movement x−Axis

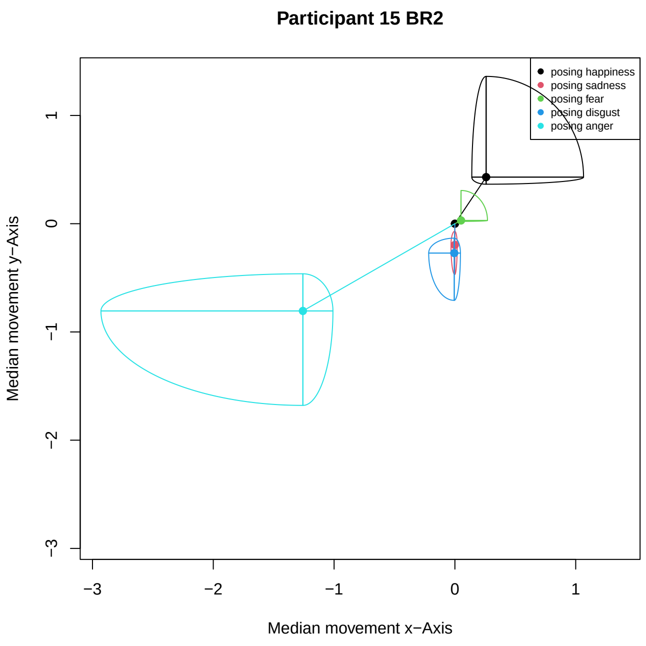
Participant 15 BR2
posing happiness
posing sadness
posing fear
posing disgust
posing anger
Median movement y−Axis
Median movement x−Axis

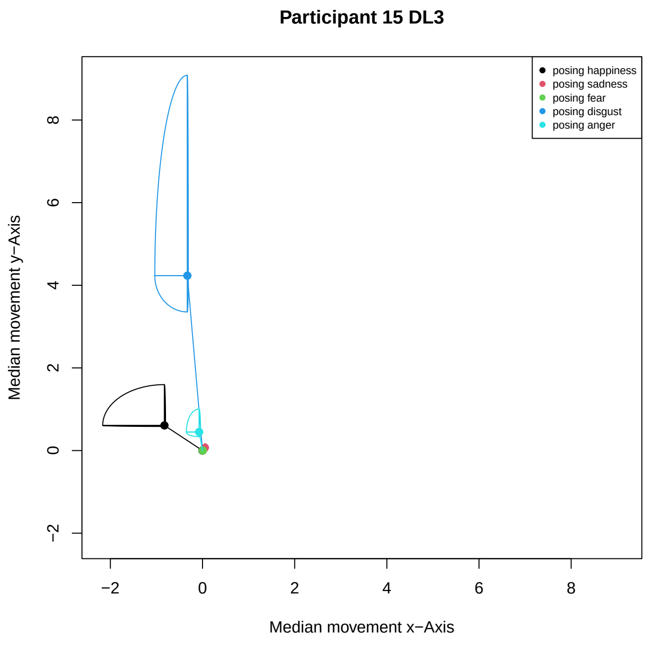
Participant 15 DL3
posing happiness
posing sadness
posing fear
posing disgust
posing anger
Median movement y−Axis
Median movement x−Axis

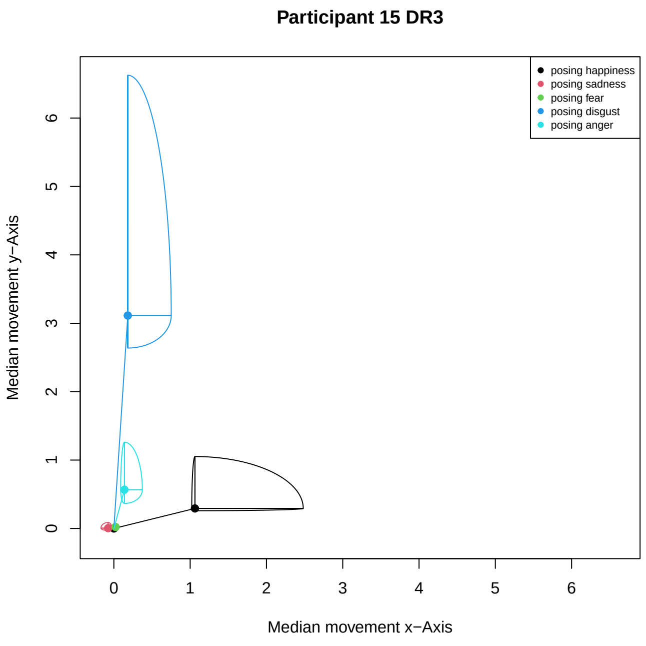
Participant 15 DR3
posing happiness
posing sadness
posing fear
posing disgust
posing anger
Median movement y−Axis
Median movement x−Axis

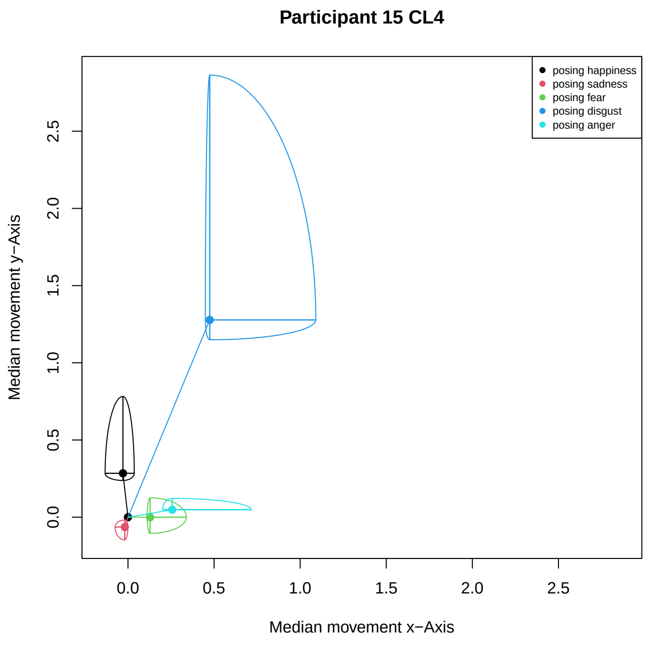
Participant 15 CL4
posing happiness
posing sadness
posing fear
posing disgust
posing anger
Median movement y−Axis
Median movement x−Axis

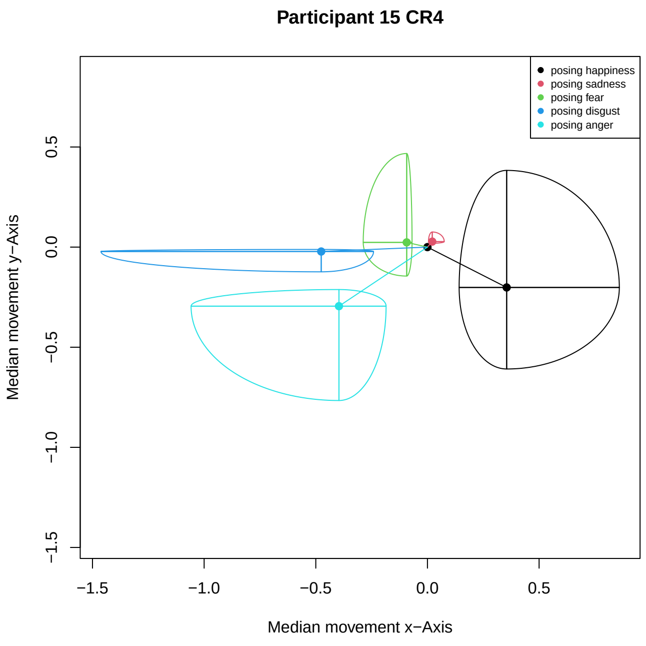
Participant 15 CR4
posing happiness
posing sadness
posing fear
posing disgust
posing anger
Median movement y−Axis
Median movement x−Axis

Participant 15 BL5

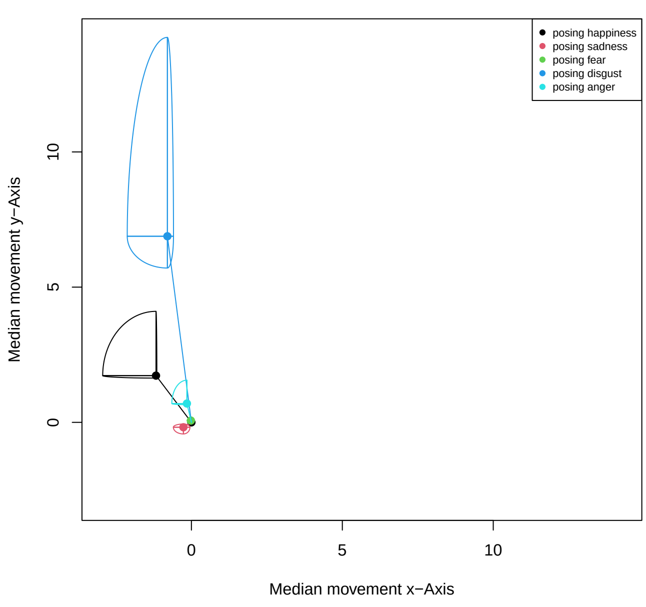

Participant 15 BR5

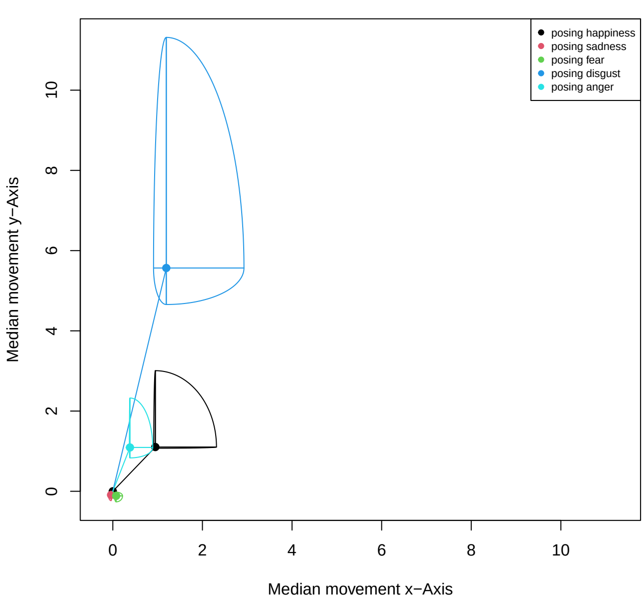

Participant 15 BL7

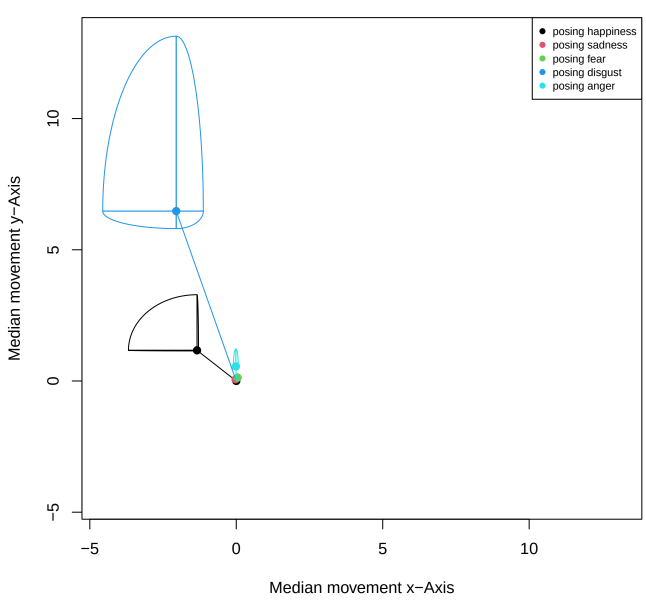

Participant 15 BR7

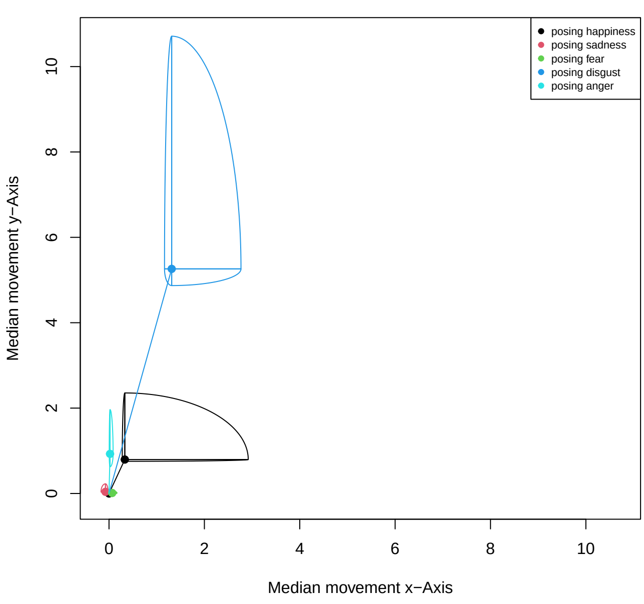

Participant 15 CL7

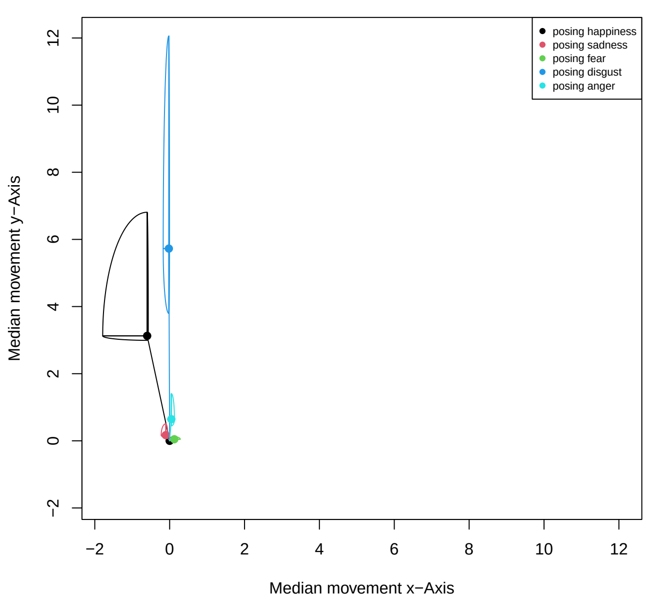

Participant 15 CR7

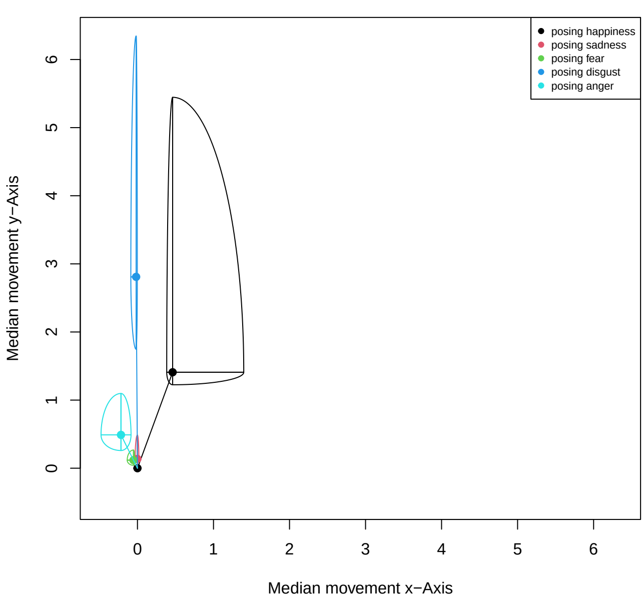

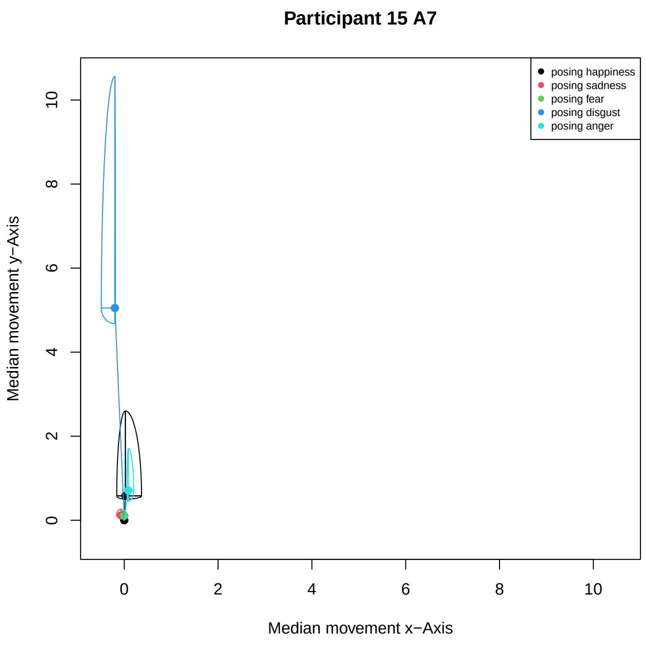

Participant 15 A7
posing happiness
posing sadness
posing fear
posing disgust
posing anger
Median movement y−Axis
Median movement x−Axis
0
2
4
6
8
10

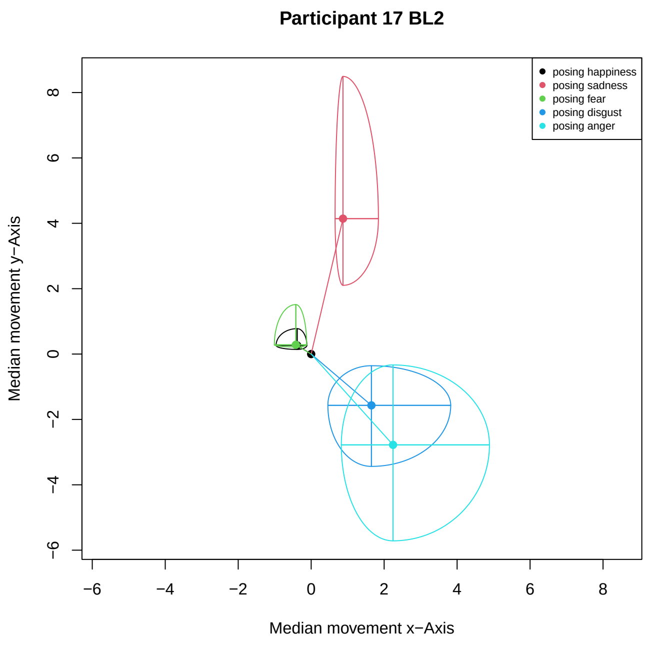
Participant 17 BL2
posing happiness
posing sadness
posing fear
posing disgust
posing anger
Median movement y−Axis
Median movement x−Axis
−6 −4 −2 0 2 4 6 8
−6 −4 −2 0 2 4 6 8

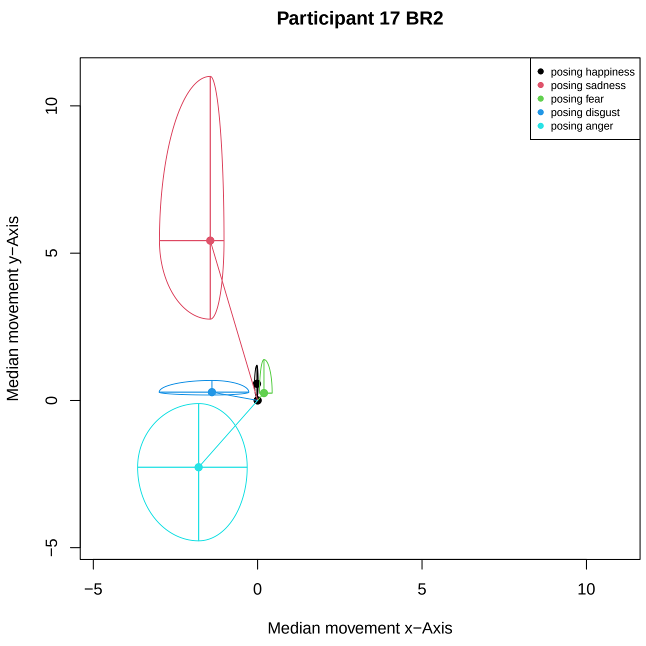
Participant 17 BR2
posing happiness
posing sadness
posing fear
posing disgust
posing anger
Median movement y−Axis
Median movement x−Axis
−5 0 5 10
−5 0 5 10

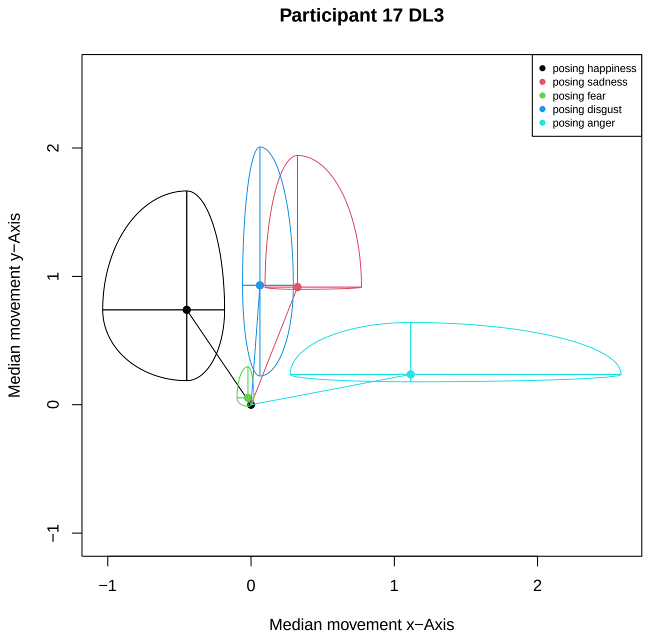
Participant 17 DL3
posing happiness
posing sadness
posing fear
posing disgust
posing anger
Median movement y−Axis
Median movement x−Axis
−1 0 1 2
−1 0 1 2

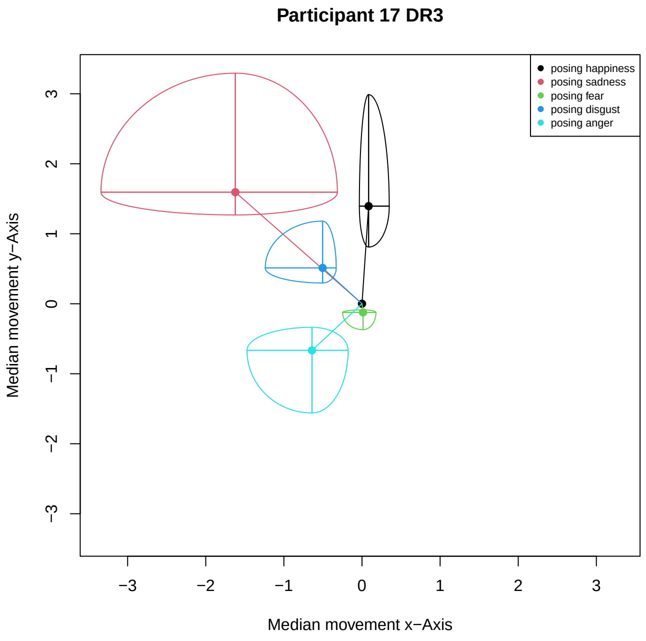
Participant 17 DR3
posing happiness
posing sadness
posing fear
posing disgust
posing anger
Median movement y−Axis
Median movement x−Axis
−3 −2 −1 0 1 2 3
−3 −2 −1 0 1 2 3

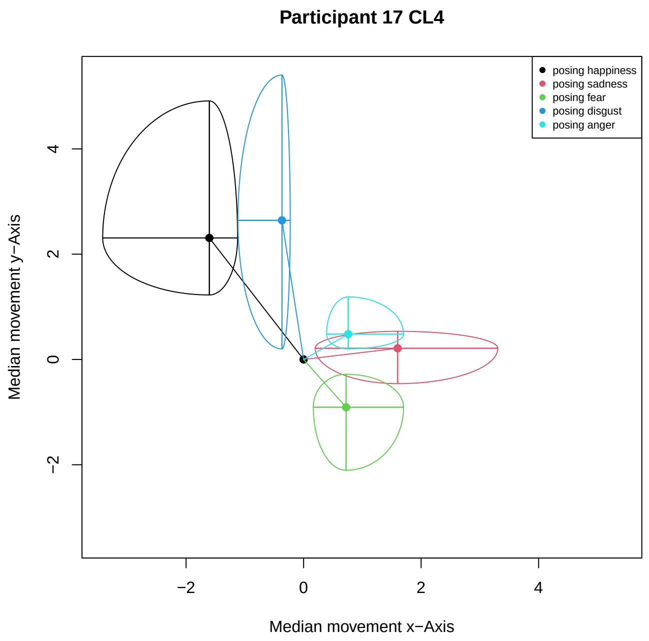
Participant 17 CL4
posing happiness
posing sadness
posing fear
posing disgust
posing anger
Median movement y−Axis
Median movement x−Axis
−2 0 2 4
−2 0 2 4

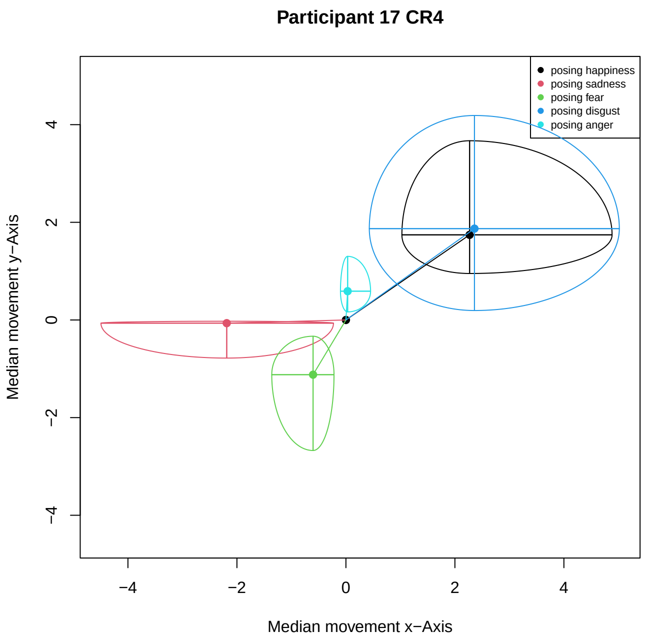
Participant 17 CR4
posing happiness
posing sadness
posing fear
posing disgust
posing anger
Median movement y−Axis
Median movement x−Axis
−4 −2 0 2 4
−4 −2 0 2 4

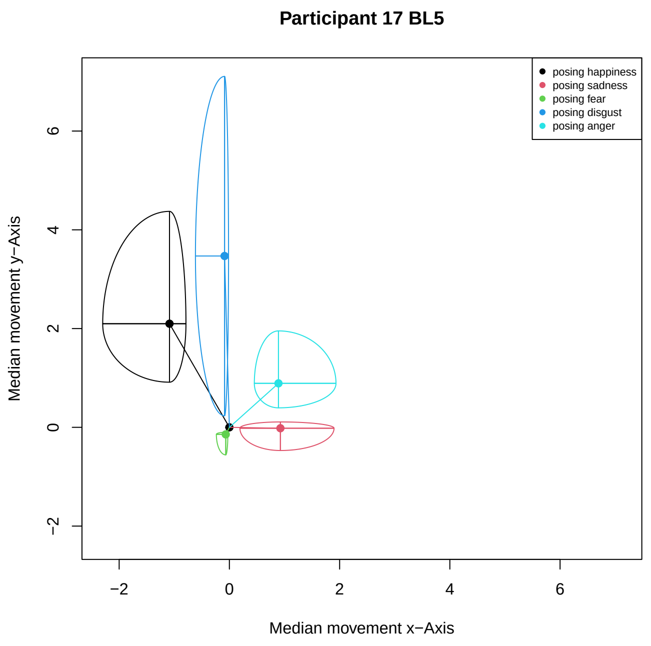
Participant 17 BL5
posing happiness
posing sadness
posing fear
posing disgust
posing anger
Median movement y−Axis
Median movement x−Axis

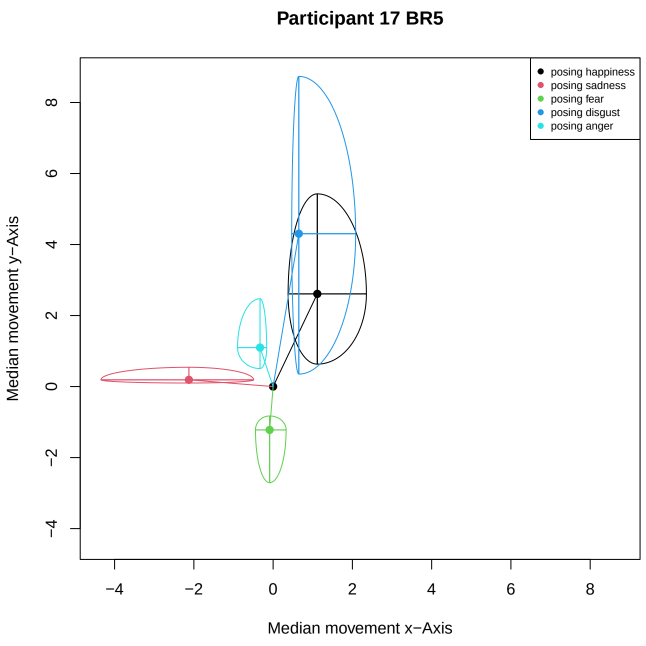
Participant 17 BR5
posing happiness
posing sadness
posing fear
posing disgust
posing anger
Median movement y−Axis
Median movement x−Axis

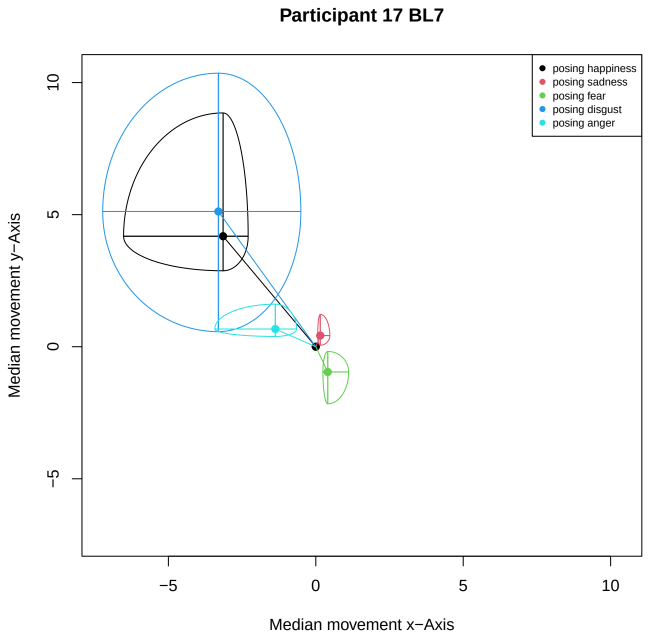
Participant 17 BL7
posing happiness
posing sadness
posing fear
posing disgust
posing anger
Median movement y−Axis
Median movement x−Axis

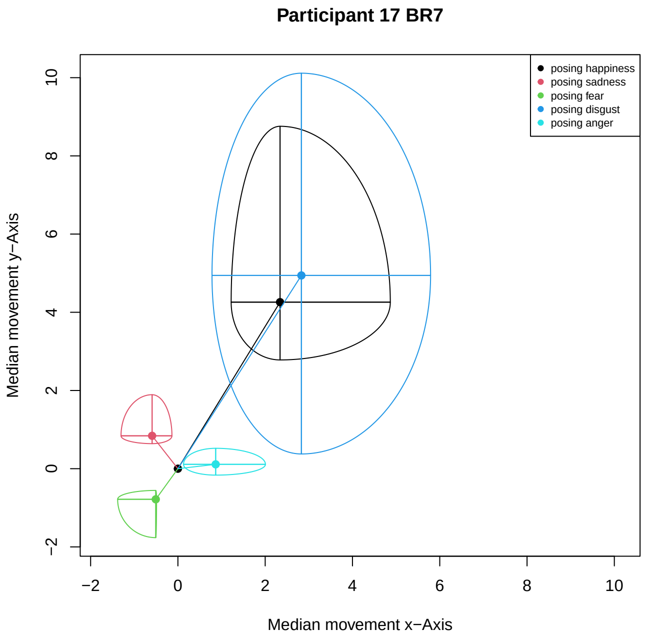
Participant 17 BR7
posing happiness
posing sadness
posing fear
posing disgust
posing anger
Median movement y−Axis
Median movement x−Axis

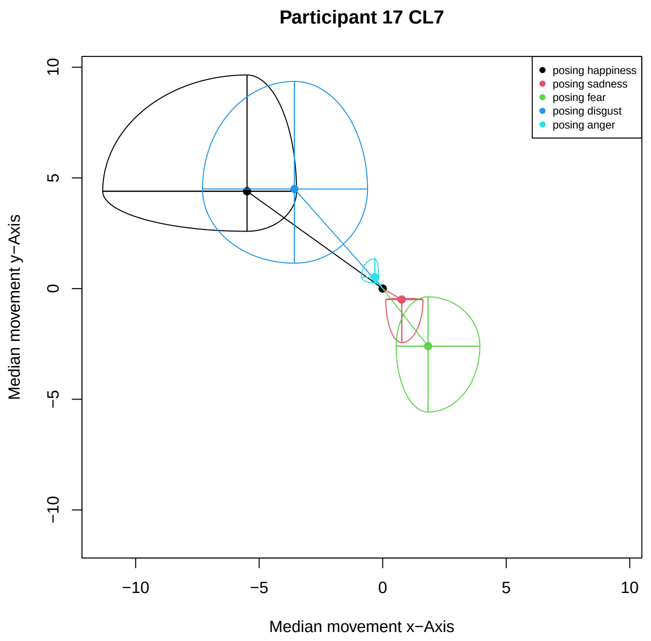
Participant 17 CL7
posing happiness
posing sadness
posing fear
posing disgust
posing anger
Median movement y−Axis
Median movement x−Axis

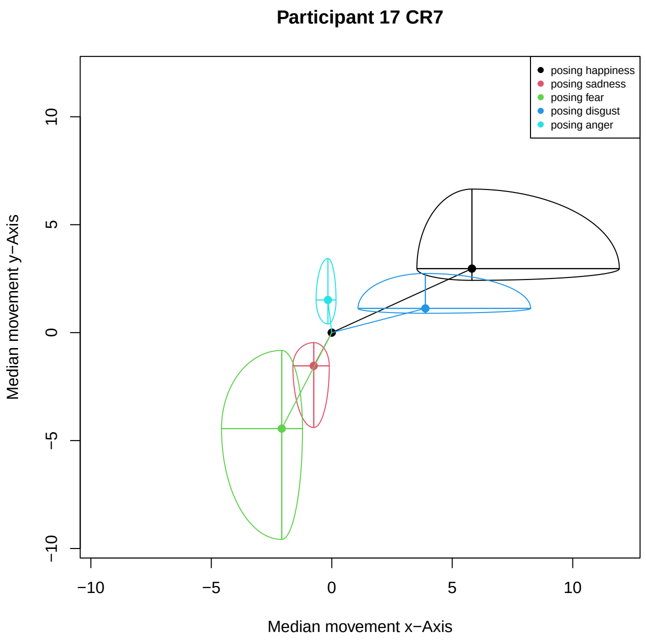
Participant 17 CR7
posing happiness
posing sadness
posing fear
posing disgust
posing anger
Median movement y−Axis
Median movement x−Axis

Participant 17 A7

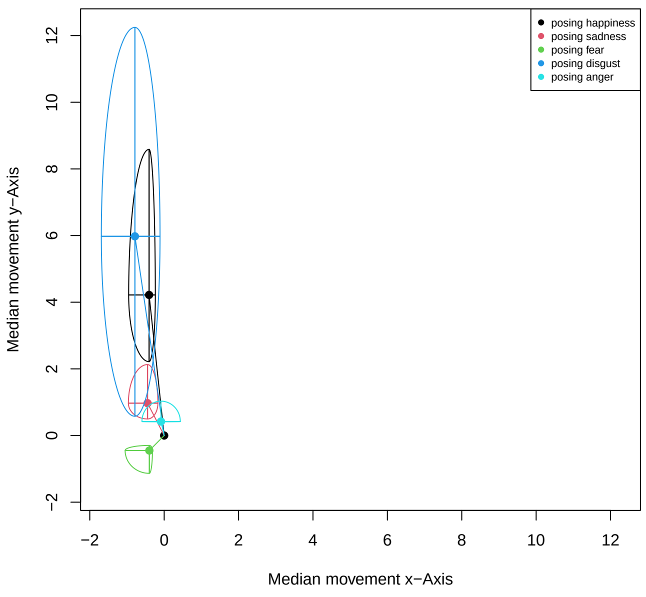

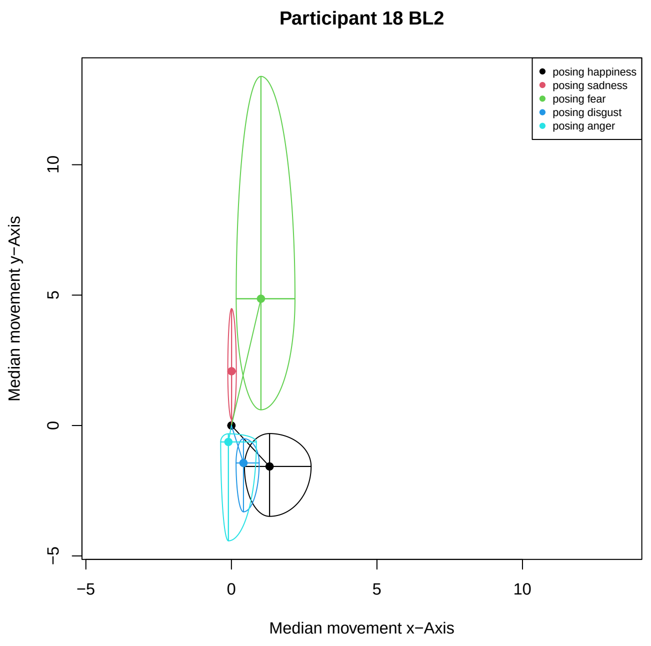
Participant 18 BL2
posing happiness
posing sadness
posing fear
posing disgust
posing anger
Median movement y−Axis
Median movement x−Axis

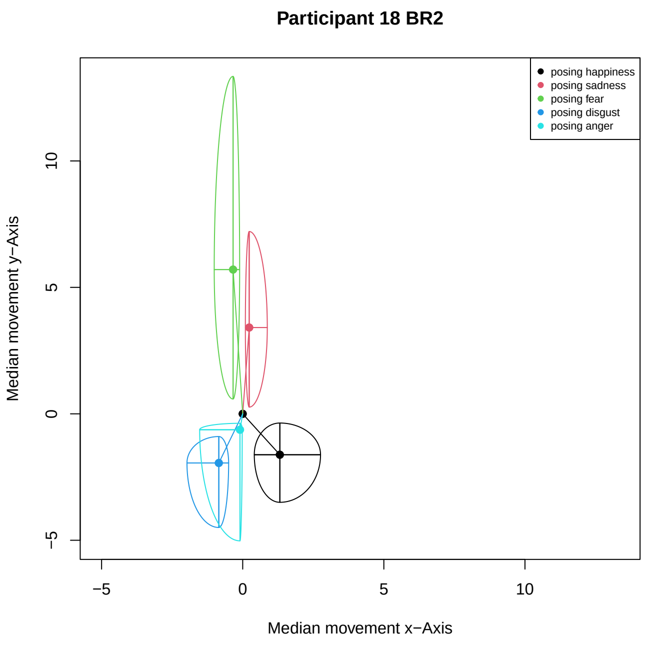
Participant 18 BR2
posing happiness
posing sadness
posing fear
posing disgust
posing anger
Median movement y−Axis
Median movement x−Axis

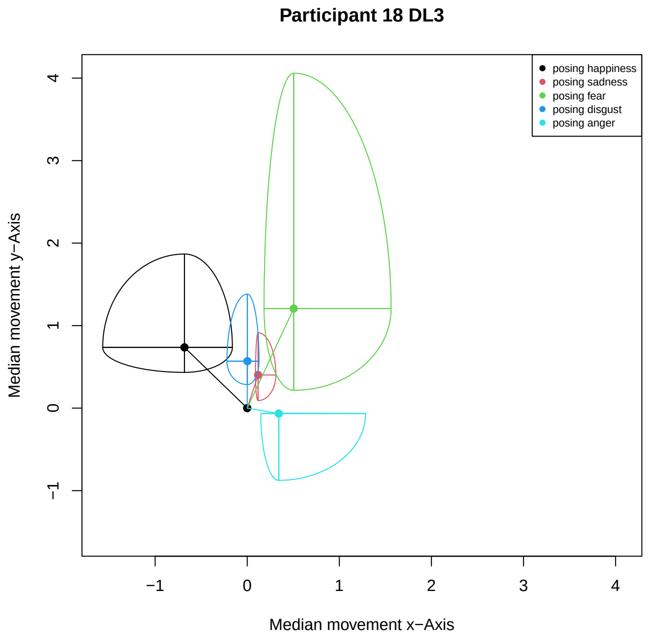
Participant 18 DL3
posing happiness
posing sadness
posing fear
posing disgust
posing anger
Median movement y−Axis
Median movement x−Axis

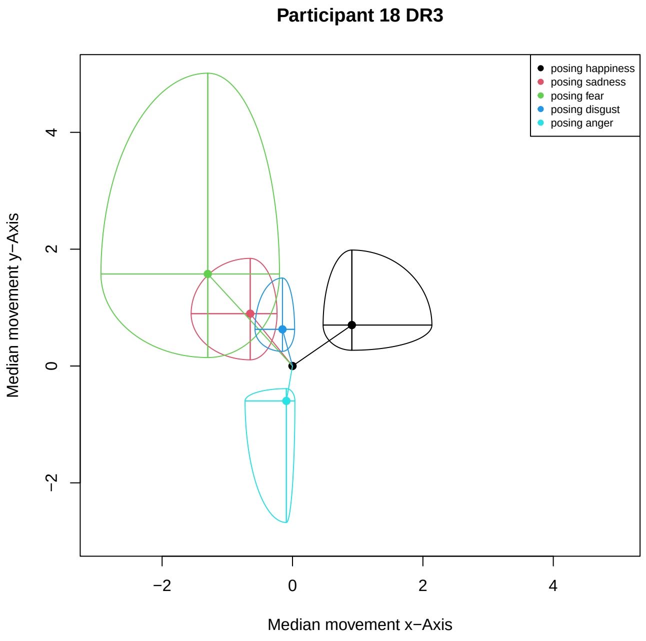
Participant 18 DR3
posing happiness
posing sadness
posing fear
posing disgust
posing anger
Median movement y−Axis
Median movement x−Axis

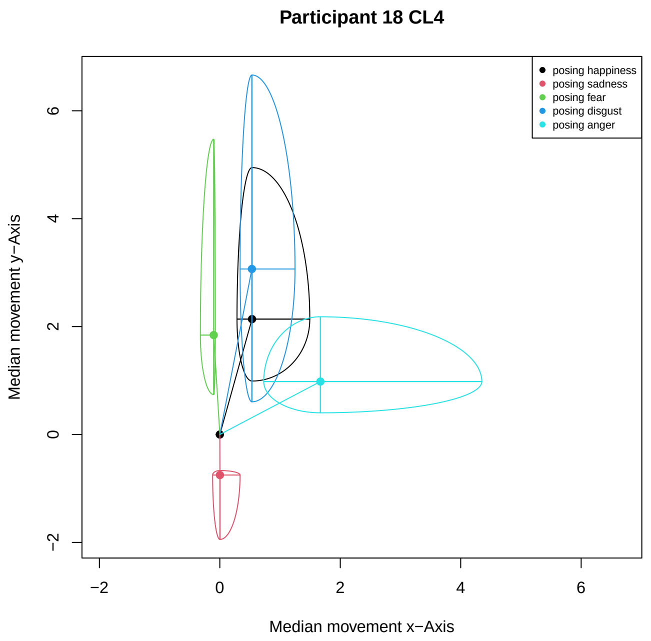
Participant 18 CL4
posing happiness
posing sadness
posing fear
posing disgust
posing anger
Median movement y−Axis
Median movement x−Axis

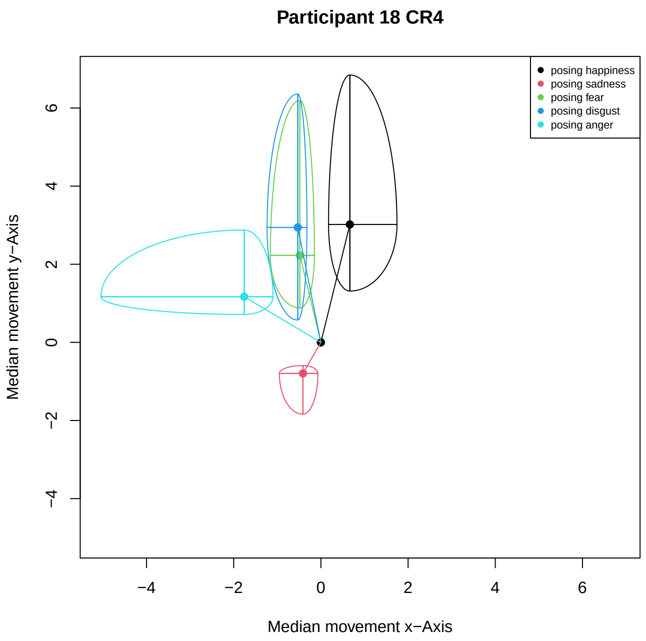
Participant 18 CR4
posing happiness
posing sadness
posing fear
posing disgust
posing anger
Median movement y−Axis
Median movement x−Axis

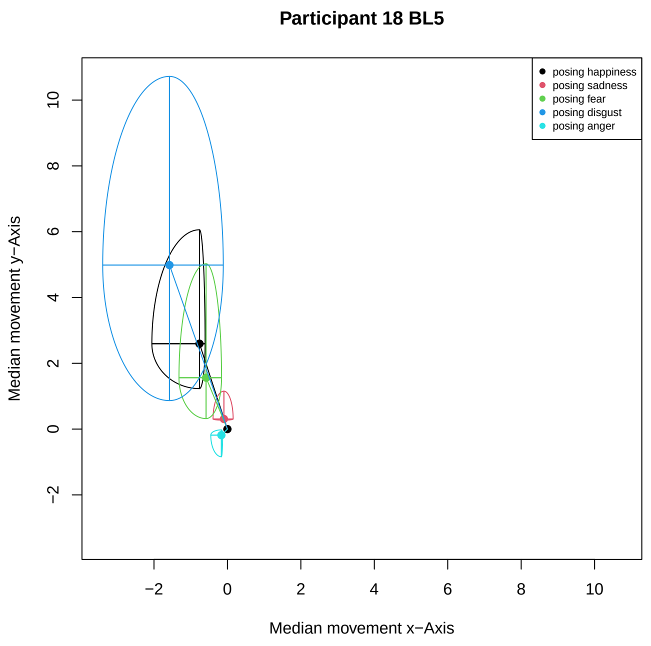
Participant 18 BL5
posing happiness
posing sadness
posing fear
posing disgust
posing anger
Median movement y−Axis
Median movement x−Axis

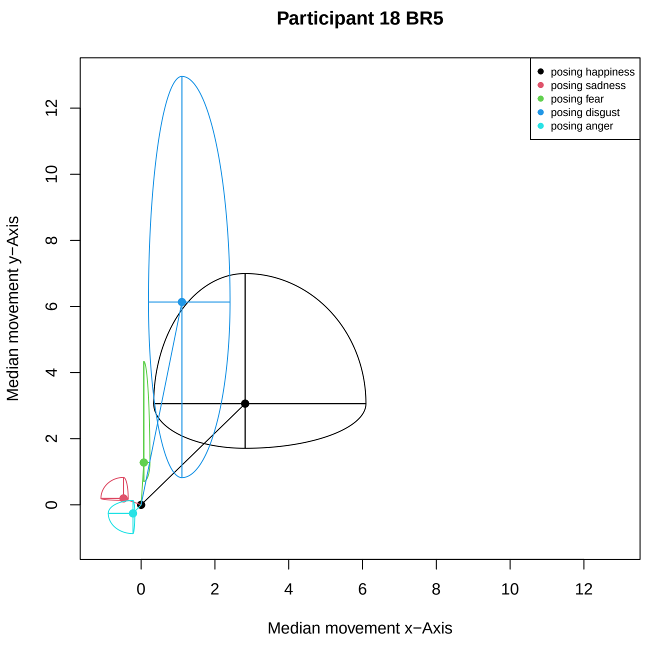
Participant 18 BR5
posing happiness
posing sadness
posing fear
posing disgust
posing anger
Median movement y−Axis
Median movement x−Axis

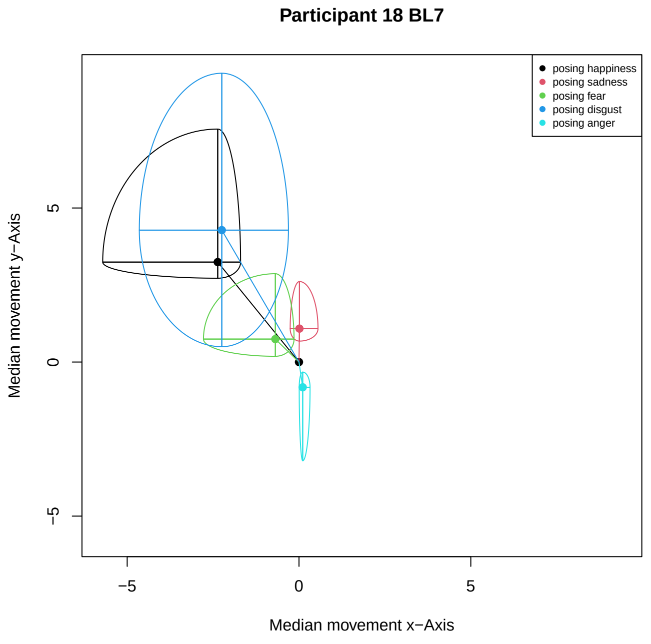
Participant 18 BL7
posing happiness
posing sadness
posing fear
posing disgust
posing anger
Median movement y−Axis
Median movement x−Axis

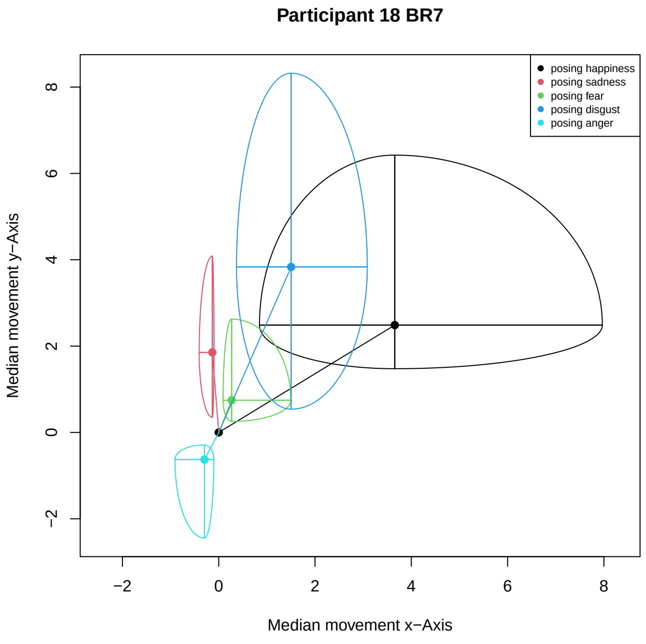
Participant 18 BR7
posing happiness
posing sadness
posing fear
posing disgust
posing anger
Median movement y−Axis
Median movement x−Axis

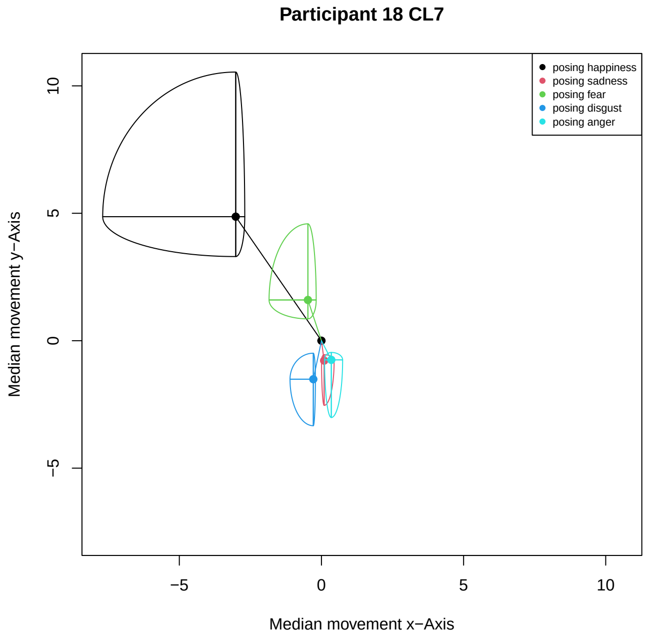
Participant 18 CL7
posing happiness
posing sadness
posing fear
posing disgust
posing anger
Median movement y−Axis
Median movement x−Axis

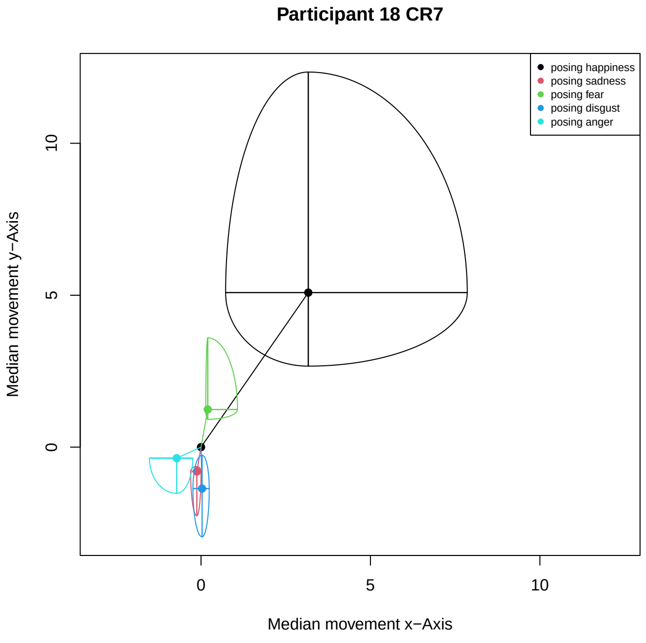
Participant 18 CR7
posing happiness
posing sadness
posing fear
posing disgust
posing anger
Median movement y−Axis
Median movement x−Axis

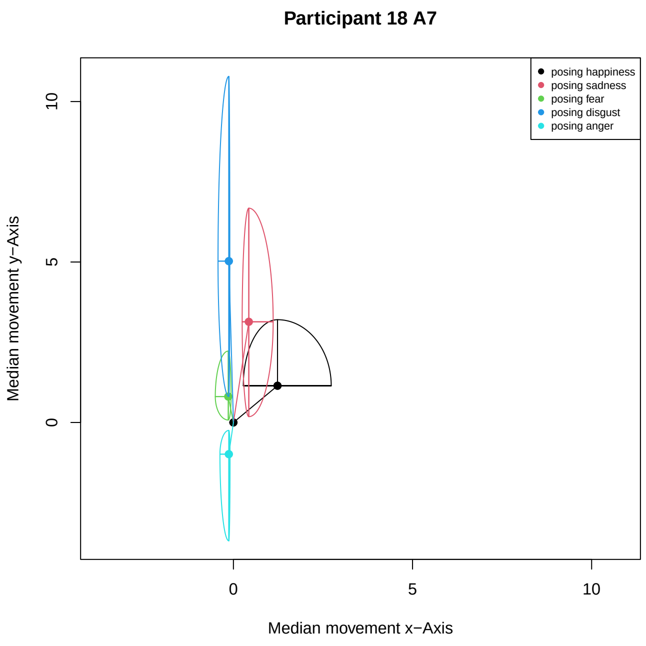

Participant 18 A7
posing happiness
posing sadness
posing fear
posing disgust
posing anger
Median movement y−Axis
0
5
10
0
5
10
Median movement x−Axis

Participant 19 BL2

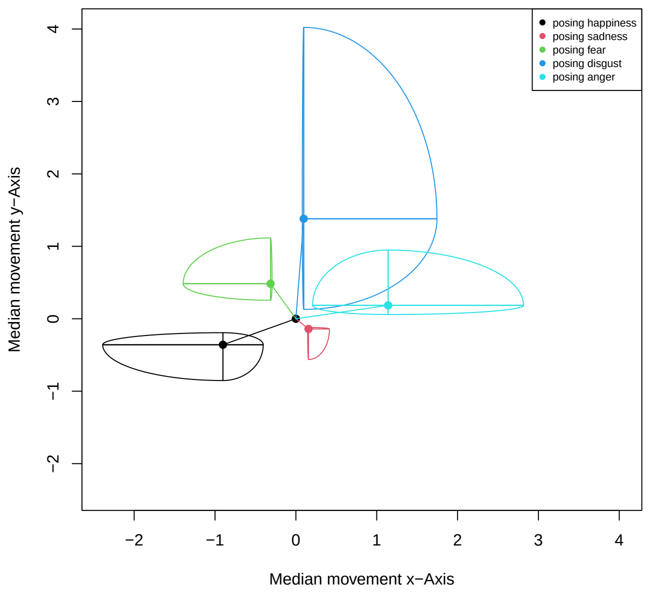

Participant 19 BR2

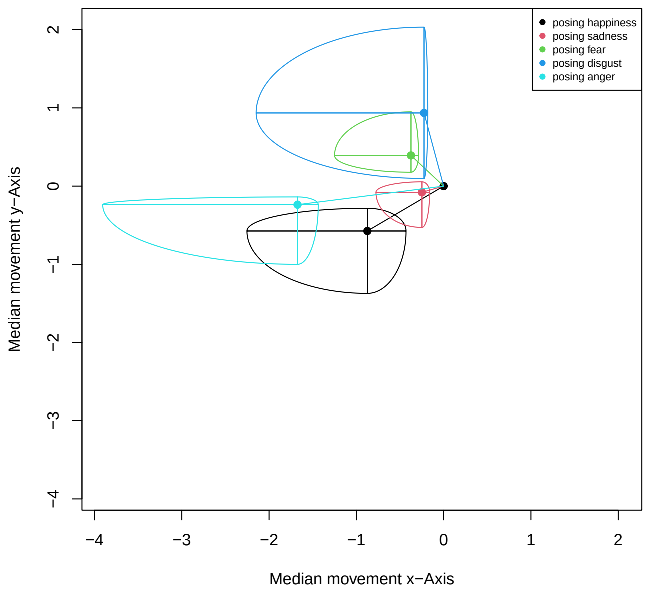

Participant 19 DL3

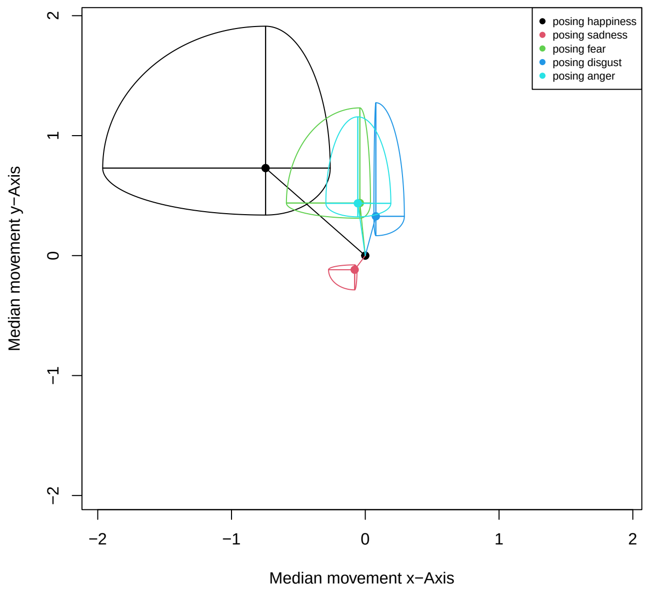

Participant 19 DR3

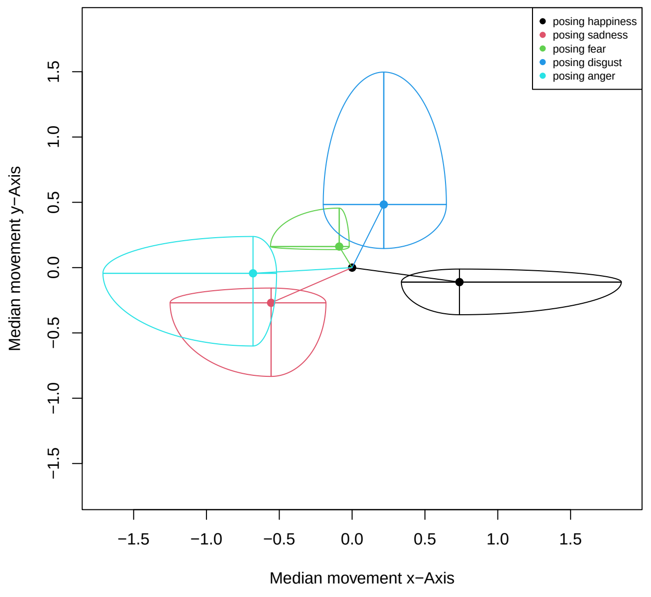

Participant 19 CL4

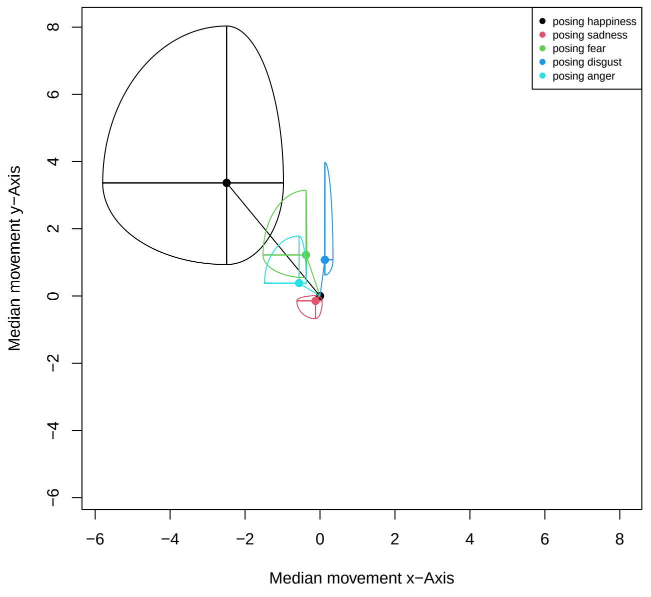

Participant 19 CR4

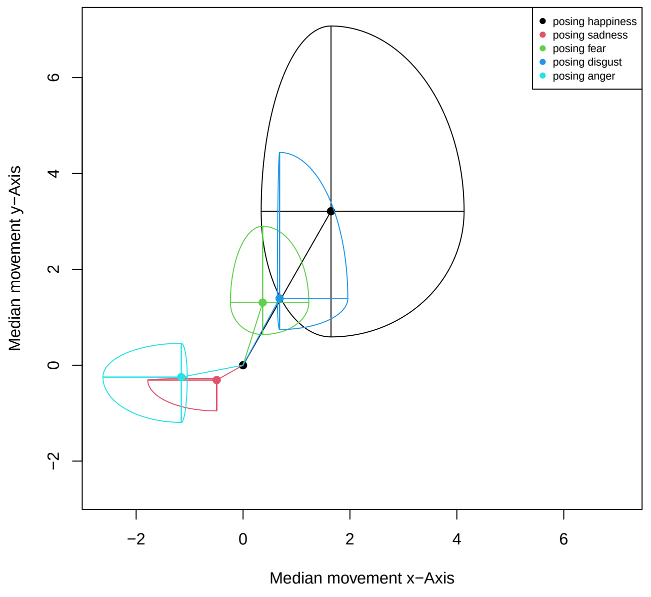

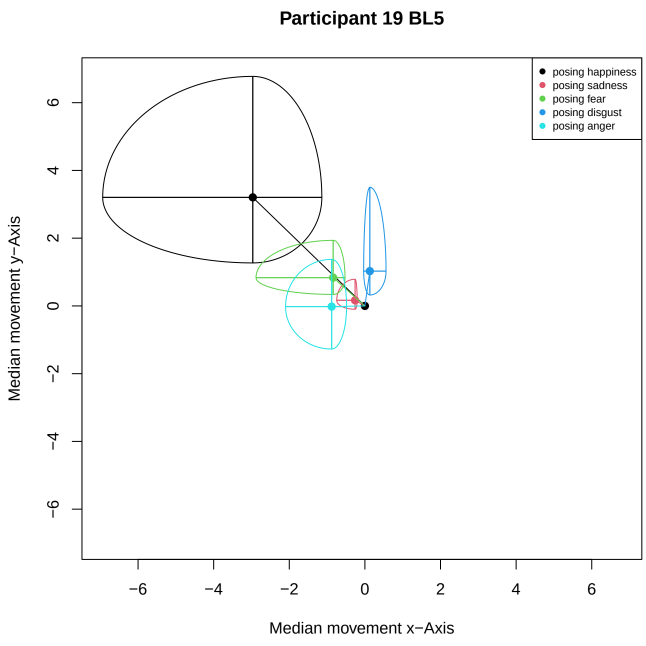
Participant 19 BL5
posing happiness
posing sadness
posing fear
posing disgust
posing anger
Median movement x−Axis
Median movement y−Axis

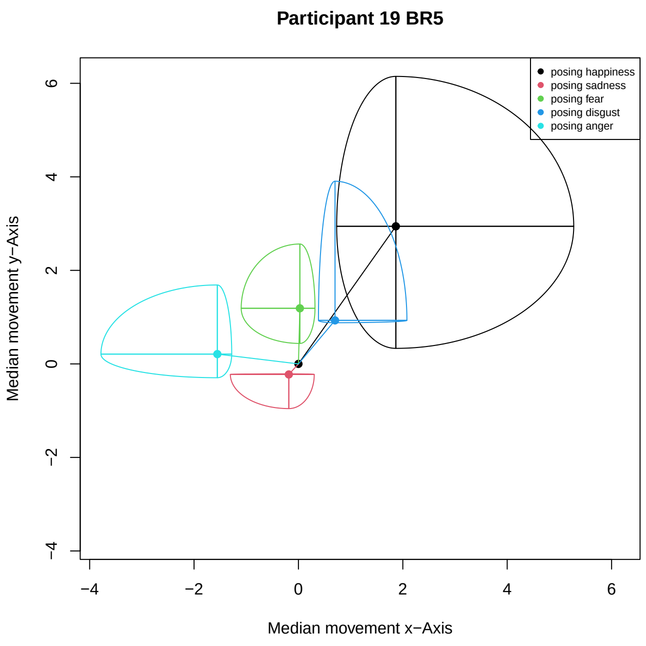
Participant 19 BR5
posing happiness
posing sadness
posing fear
posing disgust
posing anger
Median movement x−Axis
Median movement y−Axis

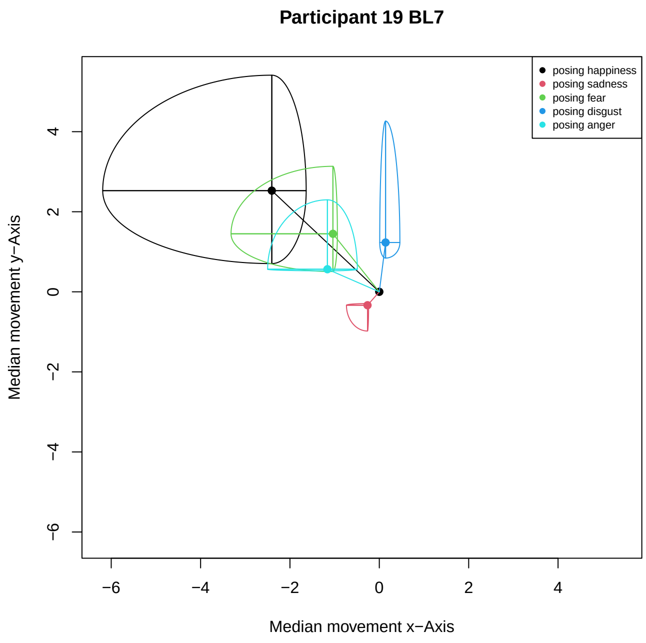
Participant 19 BL7
posing happiness
posing sadness
posing fear
posing disgust
posing anger
Median movement x−Axis
Median movement y−Axis

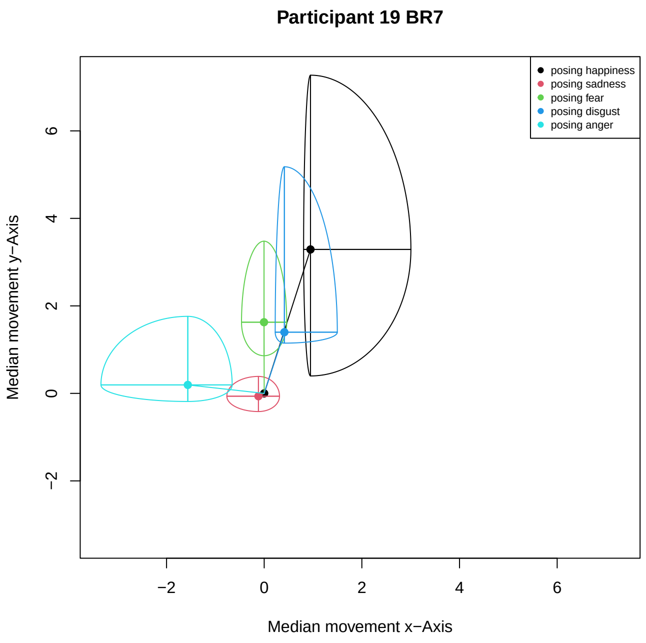
Participant 19 BR7
posing happiness
posing sadness
posing fear
posing disgust
posing anger
Median movement x−Axis
Median movement y−Axis

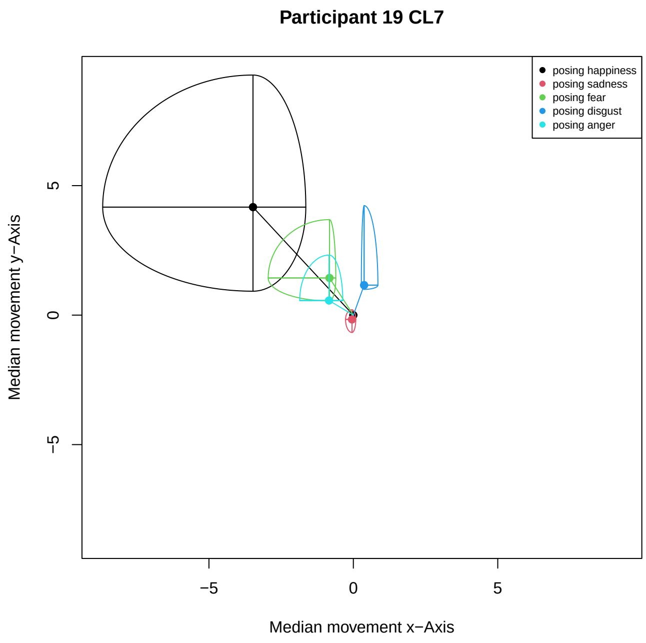
Participant 19 CL7
posing happiness
posing sadness
posing fear
posing disgust
posing anger
Median movement x−Axis
Median movement y−Axis

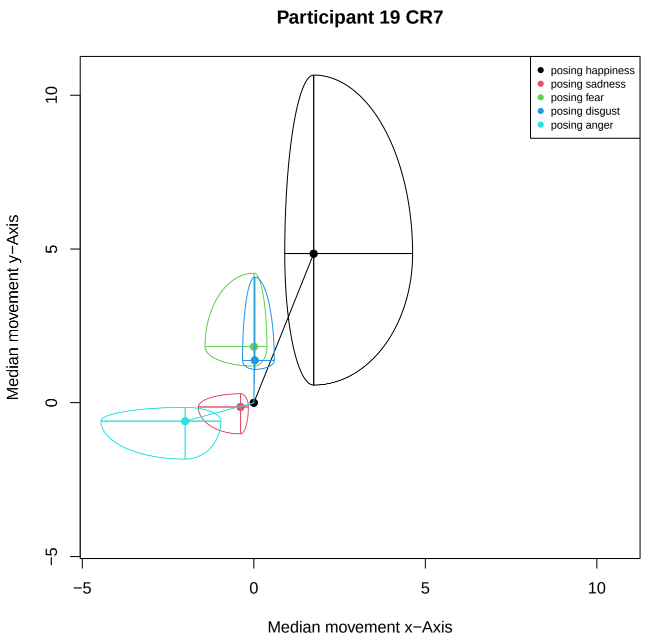
Participant 19 CR7
posing happiness
posing sadness
posing fear
posing disgust
posing anger
Median movement x−Axis
Median movement y−Axis

Participant 19 A7

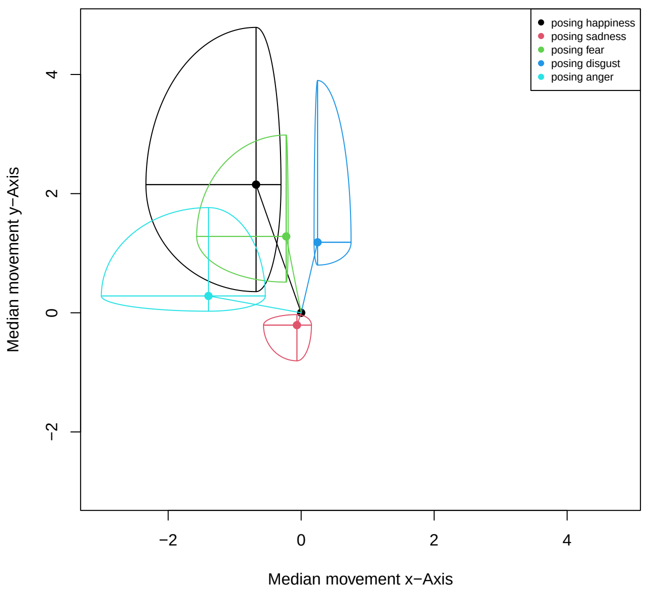

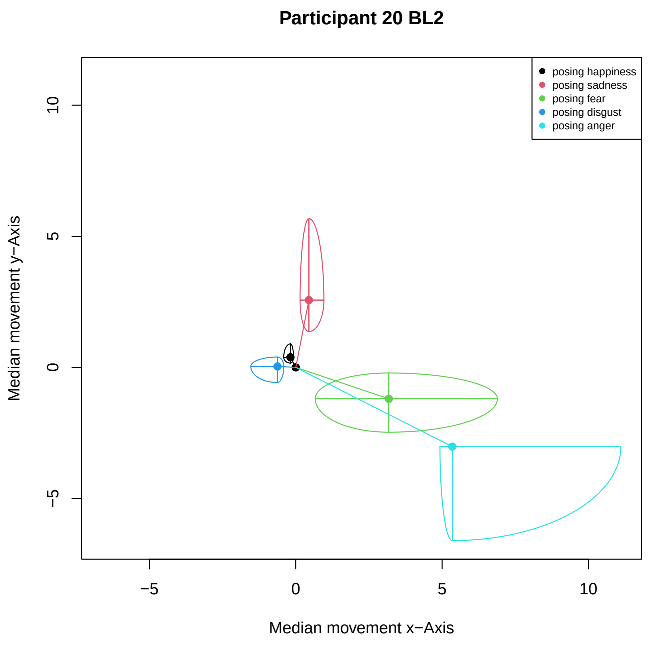
Participant 20 BL2
posing happiness
posing sadness
posing fear
posing disgust
posing anger
Median movement x−Axis
Median movement y−Axis

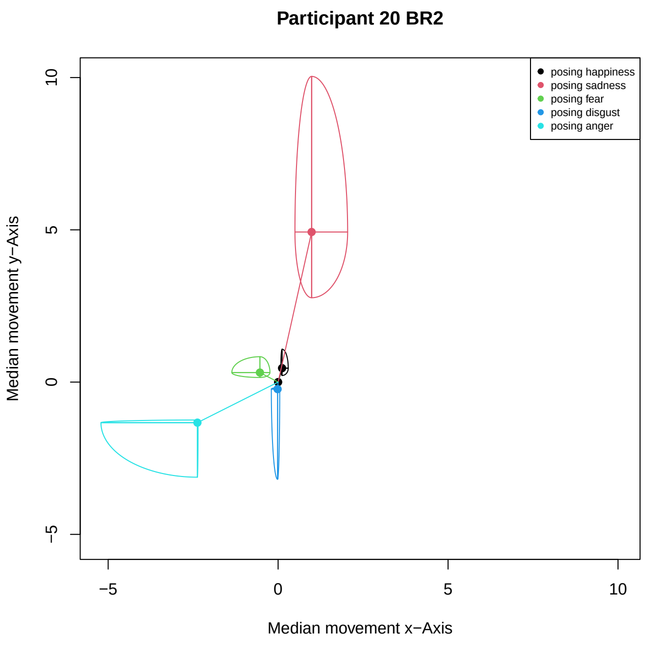
Participant 20 BR2
posing happiness
posing sadness
posing fear
posing disgust
posing anger
Median movement x−Axis
Median movement y−Axis

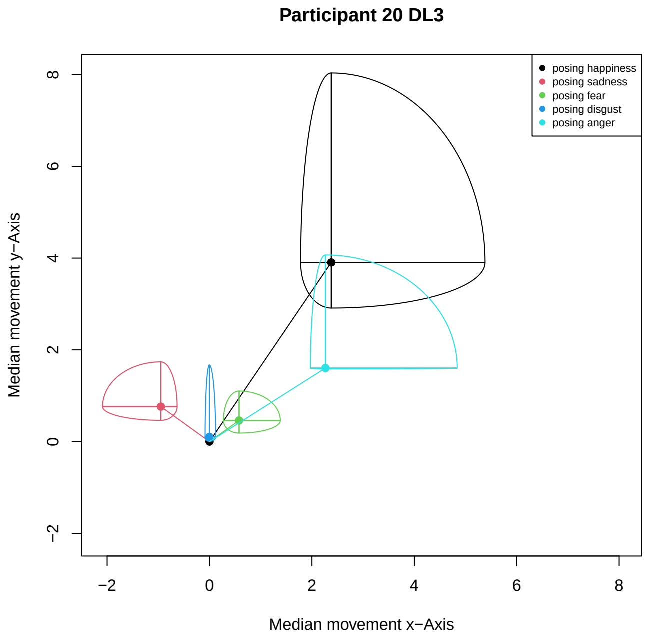
Participant 20 DL3
posing happiness
posing sadness
posing fear
posing disgust
posing anger
Median movement x−Axis
Median movement y−Axis

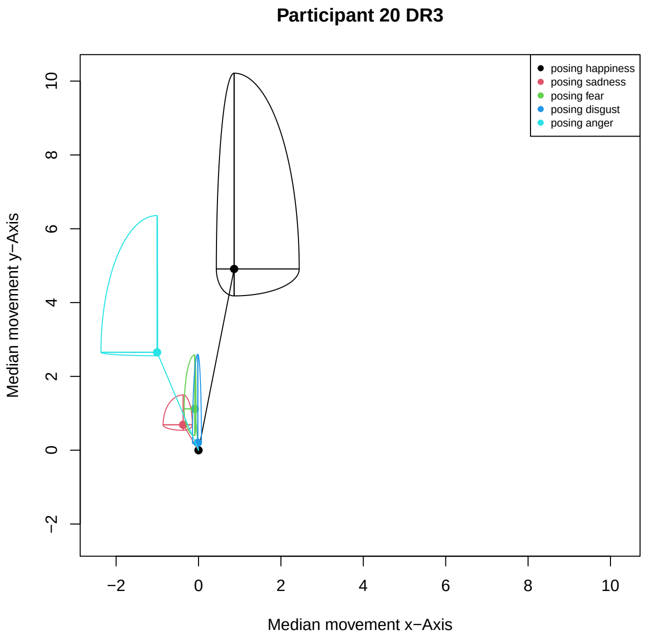
Participant 20 DR3
posing happiness
posing sadness
posing fear
posing disgust
posing anger
Median movement x−Axis
Median movement y−Axis

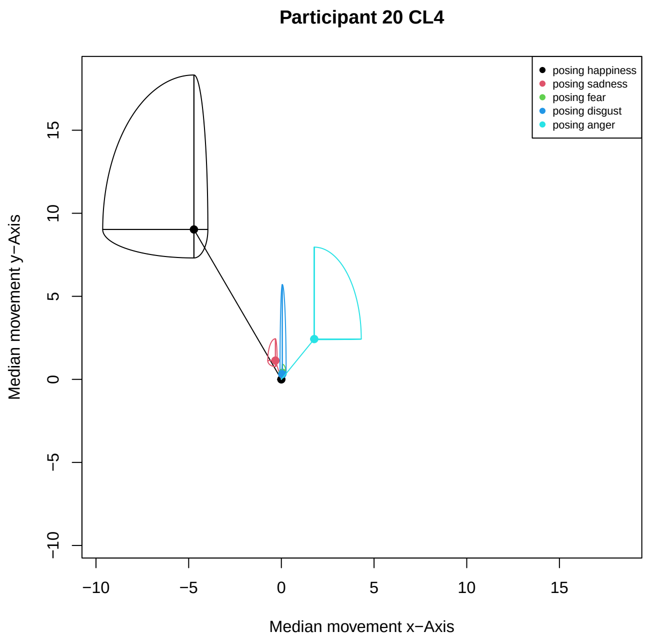
Participant 20 CL4
posing happiness
posing sadness
posing fear
posing disgust
posing anger
Median movement x−Axis
Median movement y−Axis

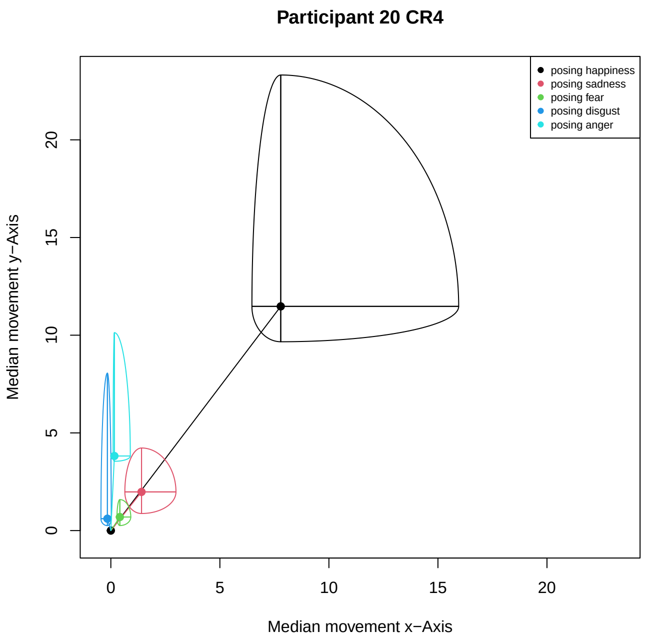
Participant 20 CR4
posing happiness
posing sadness
posing fear
posing disgust
posing anger
Median movement x−Axis
Median movement y−Axis

Participant 20 BL5

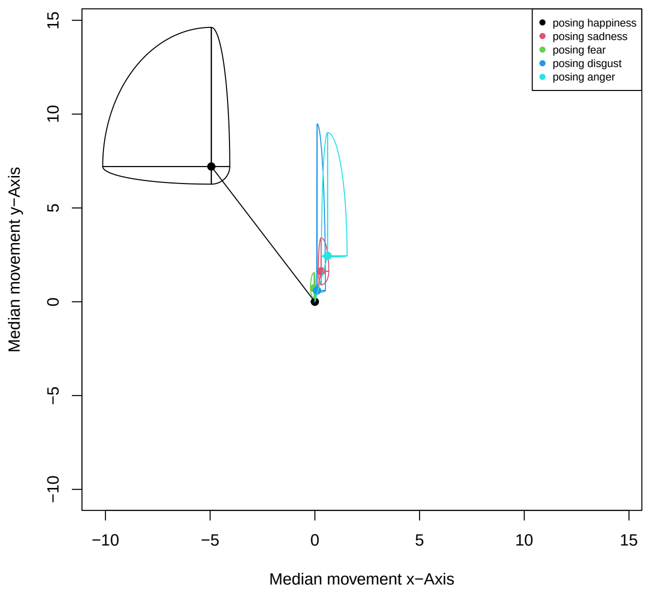

Participant 20 BR5

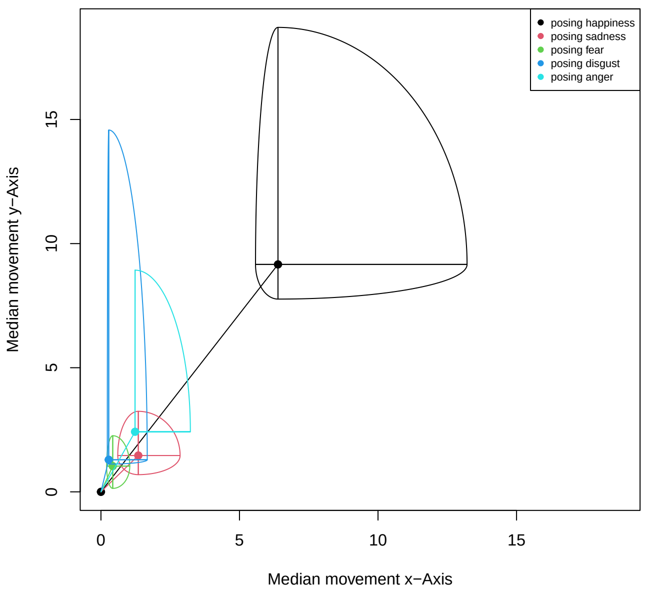

Participant 20 BL7

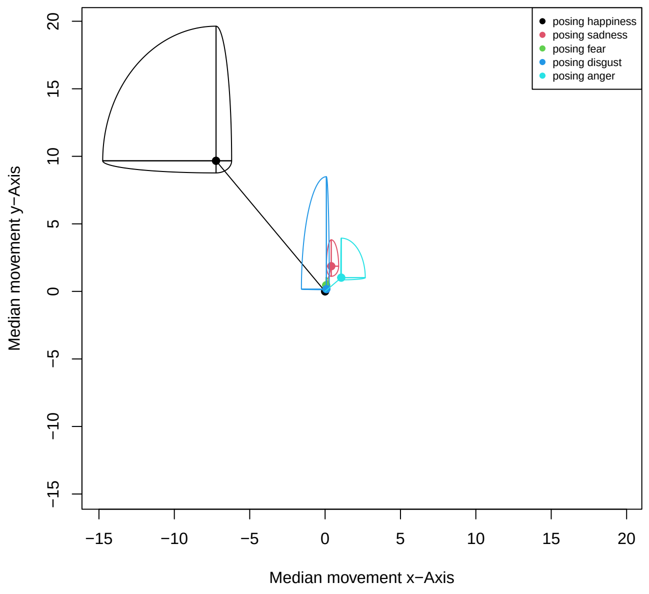

Participant 20 BR7

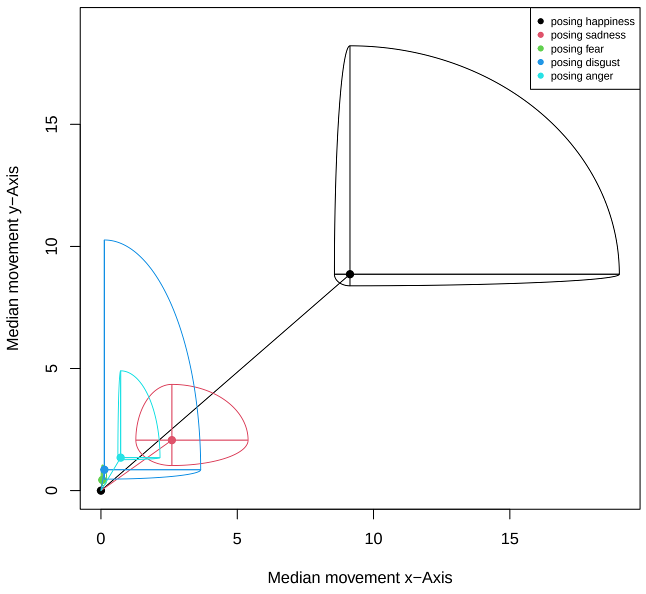

Participant 20 CL7

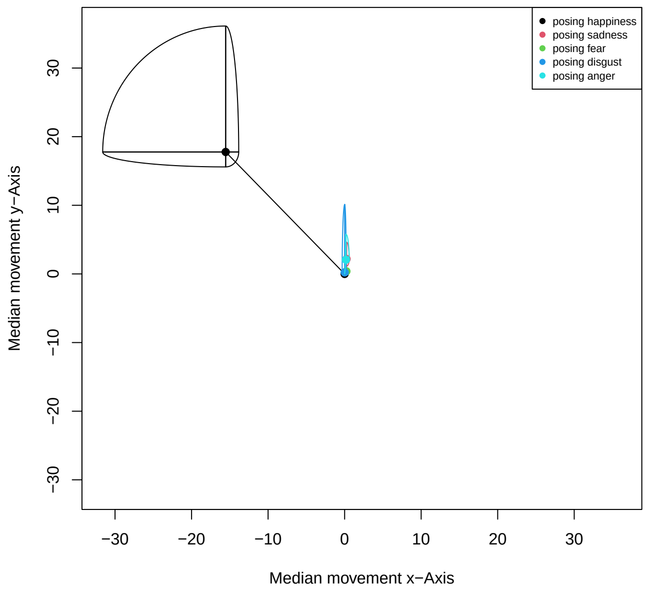

Participant 20 CR7

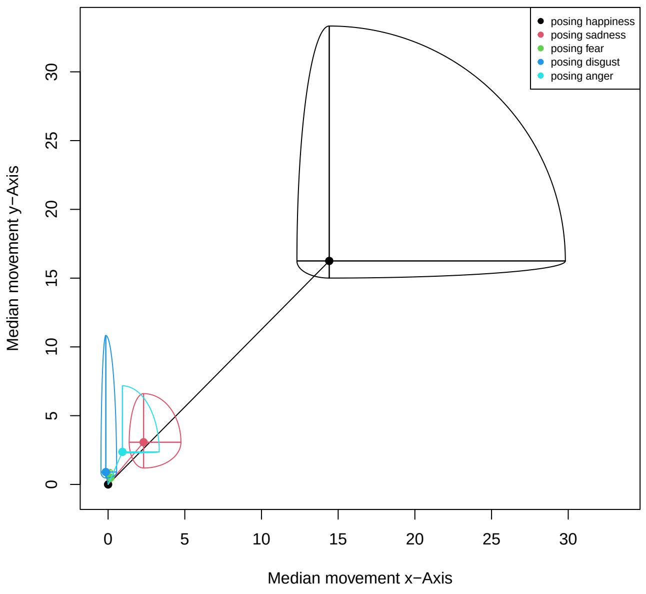

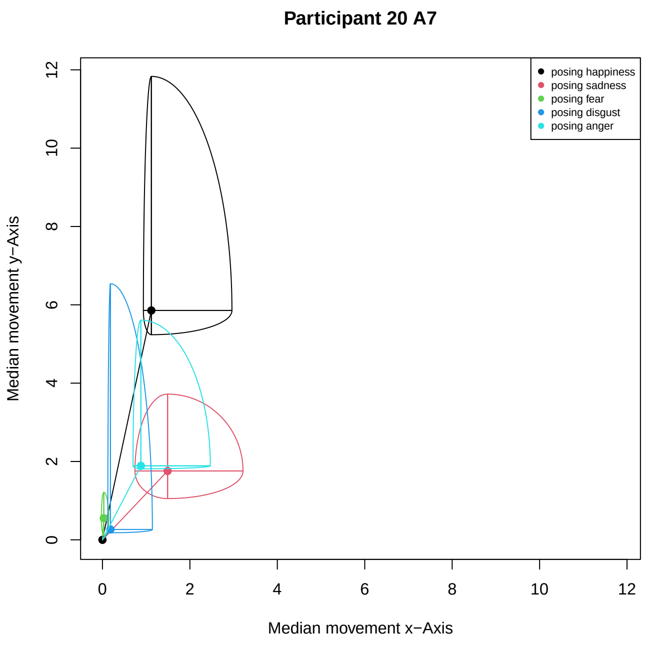

Participant 20 A7
posing happiness
posing sadness
posing fear
posing disgust
posing anger
Median movement y−Axis
Median movement x−Axis
0
2
4
6
8
10
12

Participant 21 BL2

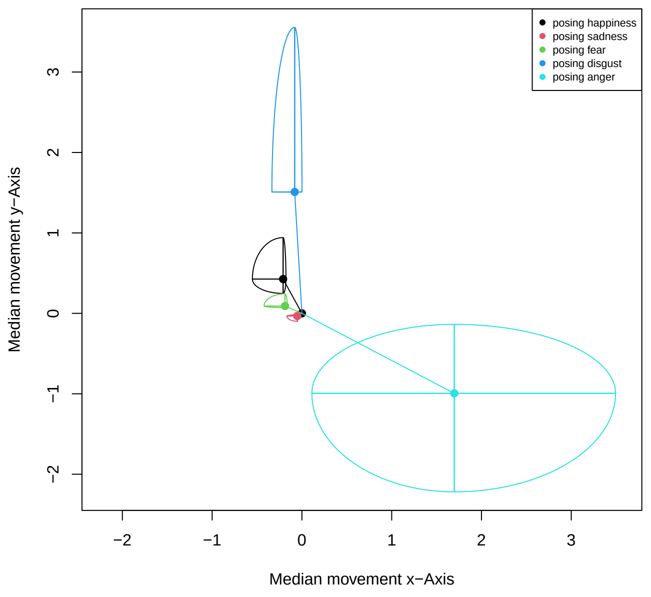

Participant 21 BR2

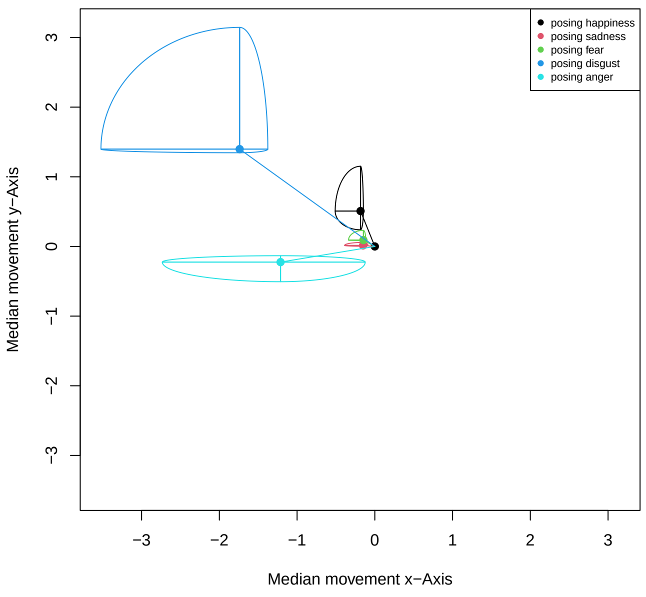

Participant 21 DL3

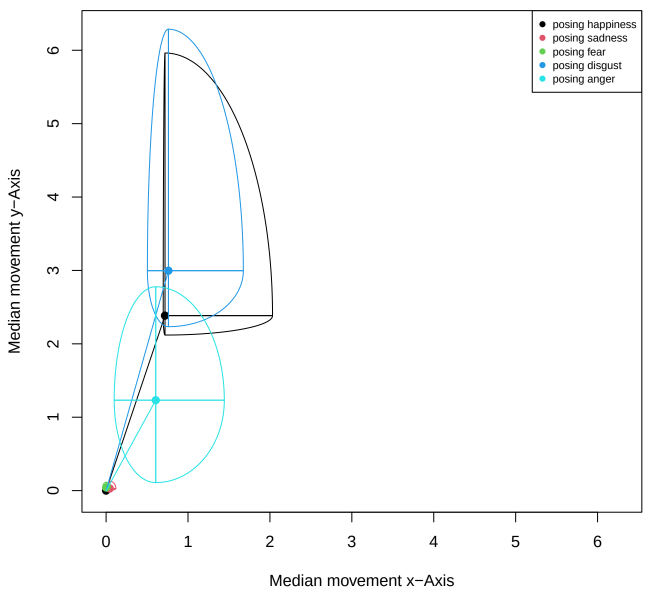

Participant 21 DR3

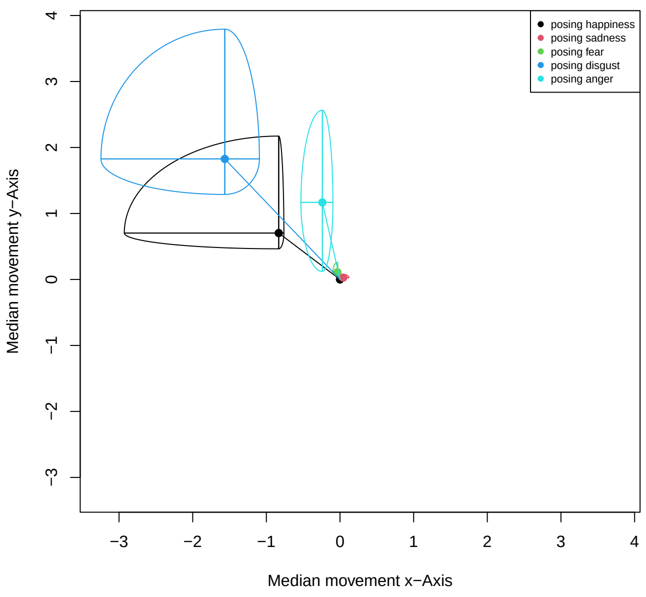

Participant 21 CL4

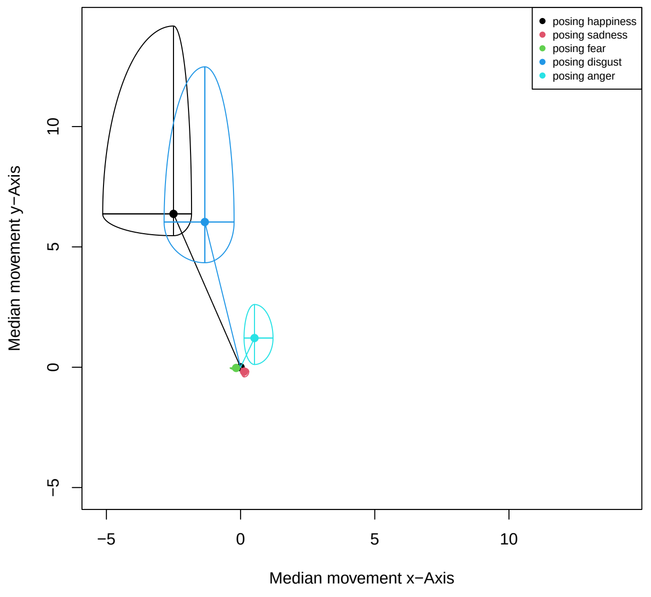

Participant 21 CR4

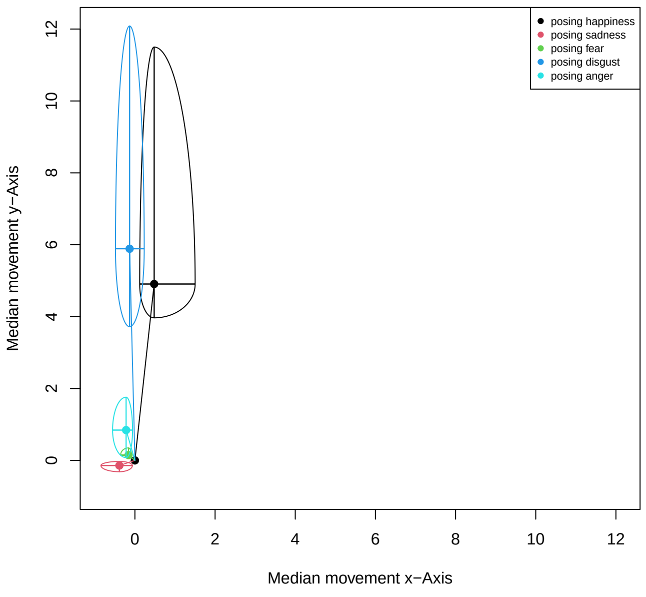

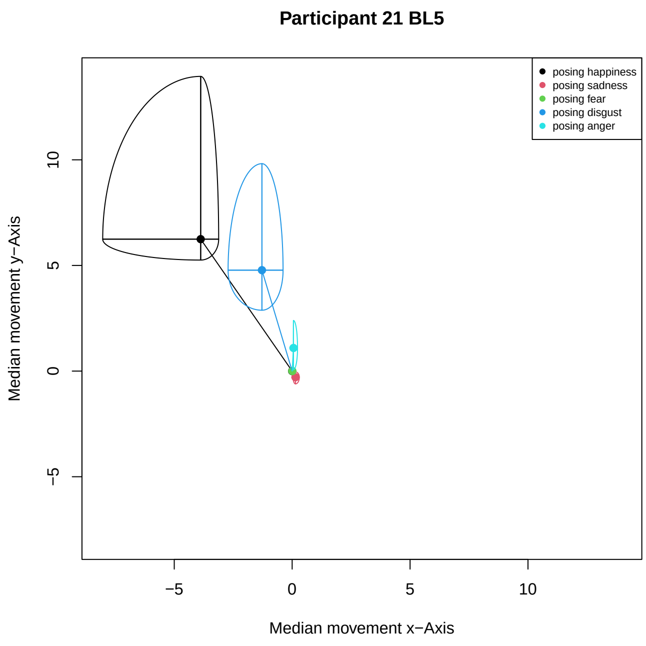
Participant 21 BL5
posing happiness
posing sadness
posing fear
posing disgust
posing anger
Median movement y−Axis
Median movement x−Axis

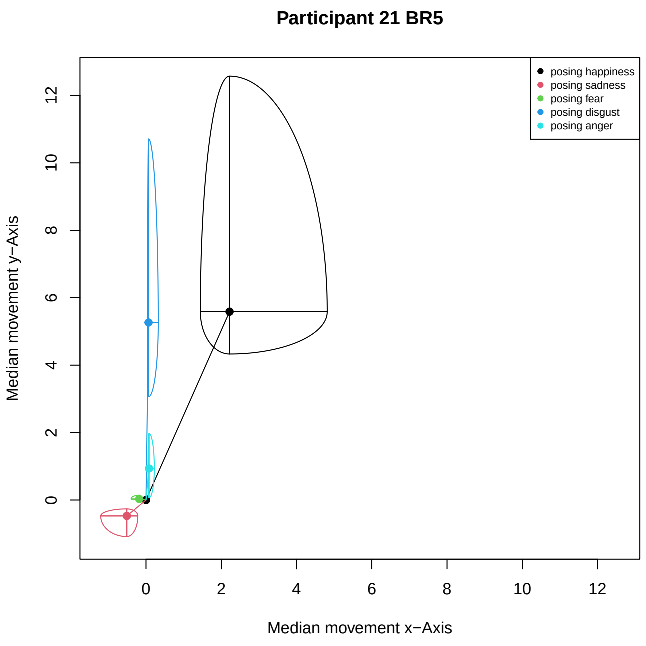
Participant 21 BR5
posing happiness
posing sadness
posing fear
posing disgust
posing anger
Median movement y−Axis
Median movement x−Axis

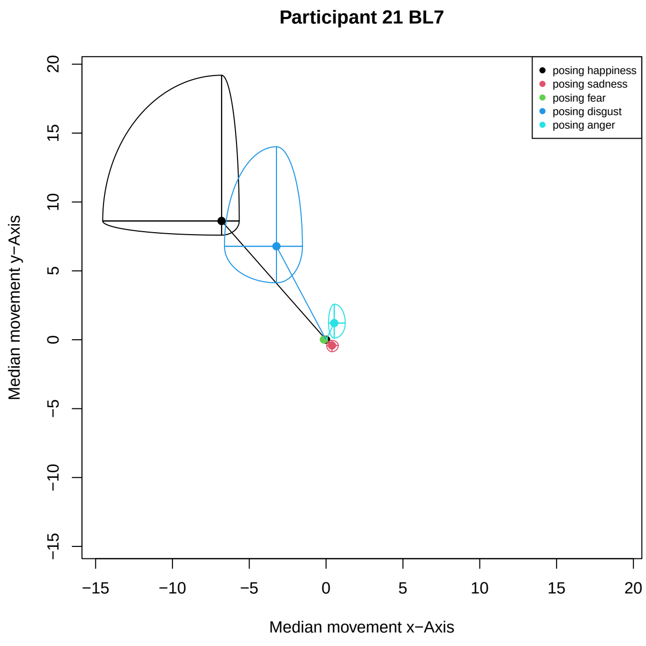
Participant 21 BL7
posing happiness
posing sadness
posing fear
posing disgust
posing anger
Median movement y−Axis
Median movement x−Axis

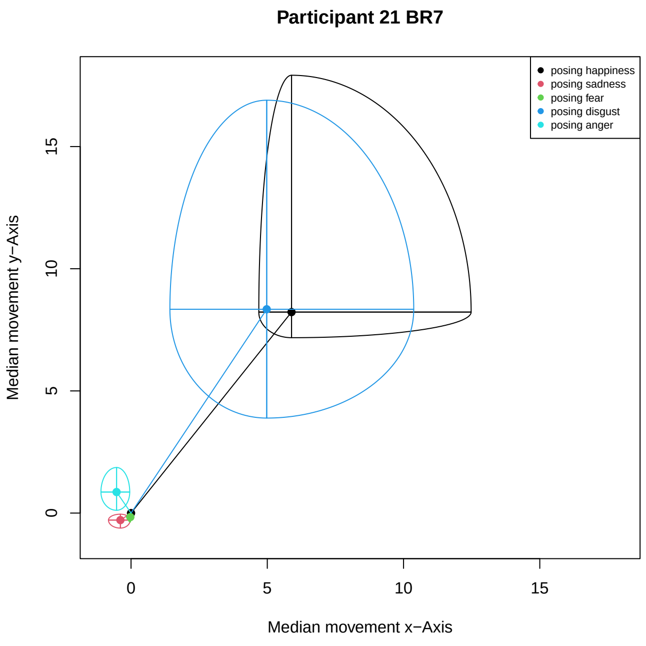
Participant 21 BR7
posing happiness
posing sadness
posing fear
posing disgust
posing anger
Median movement y−Axis
Median movement x−Axis

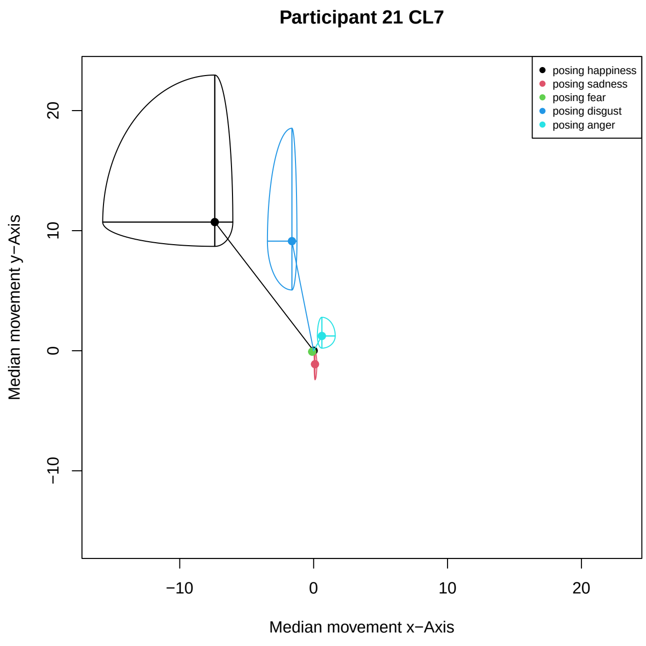
Participant 21 CL7
posing happiness
posing sadness
posing fear
posing disgust
posing anger
Median movement y−Axis
Median movement x−Axis

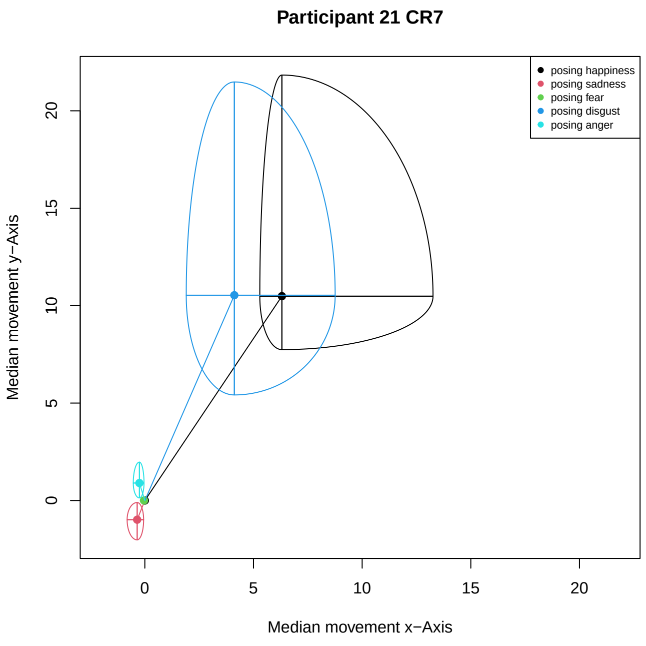
Participant 21 CR7
posing happiness
posing sadness
posing fear
posing disgust
posing anger
Median movement y−Axis
Median movement x−Axis

Participant 21 A7

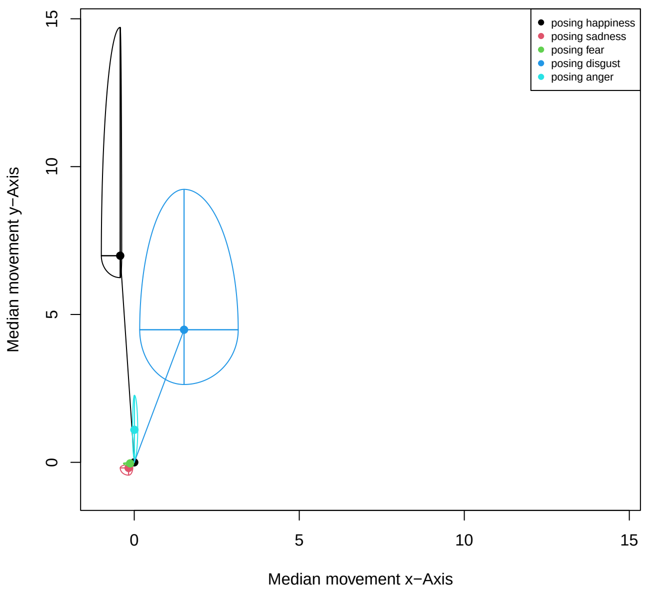

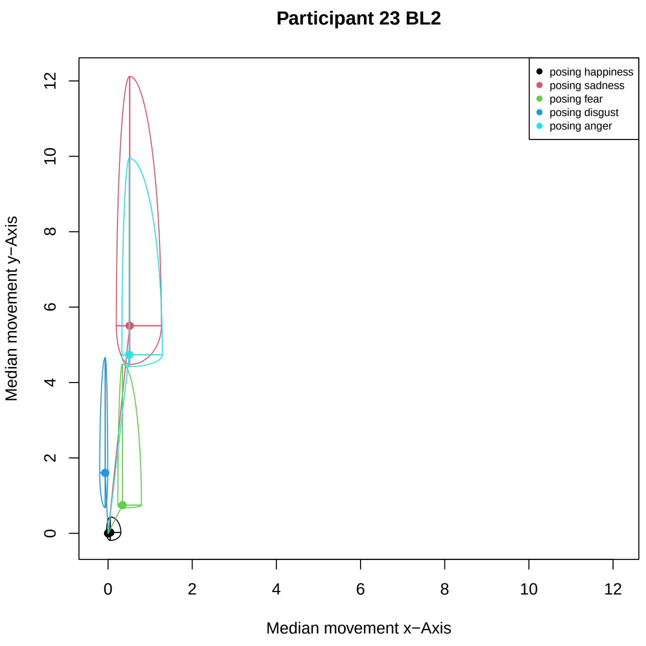
Participant 23 BL2
posing happiness
posing sadness
posing fear
posing disgust
posing anger
Median movement y−Axis
Median movement x−Axis

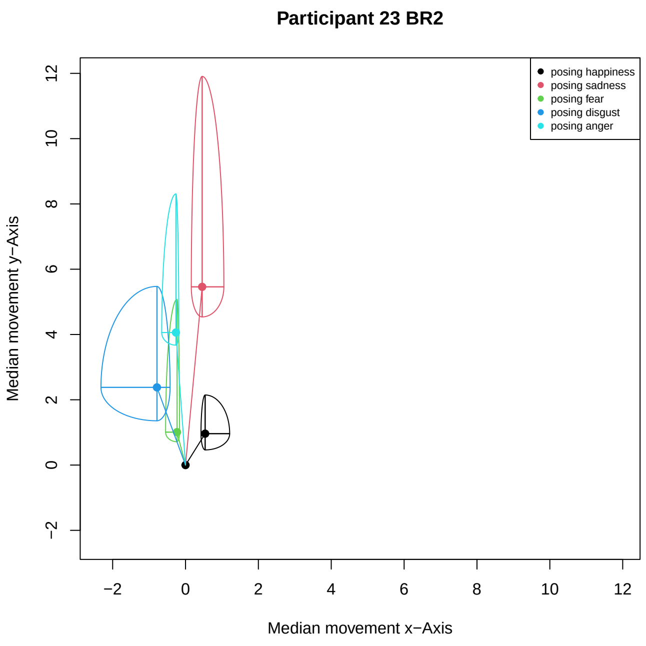
Participant 23 BR2
posing happiness
posing sadness
posing fear
posing disgust
posing anger
Median movement y−Axis
Median movement x−Axis

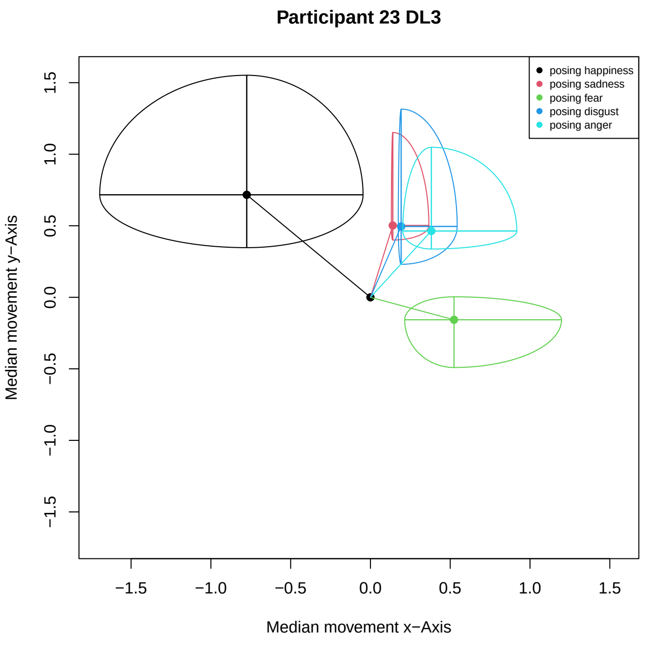
Participant 23 DL3
posing happiness
posing sadness
posing fear
posing disgust
posing anger
Median movement y−Axis
Median movement x−Axis

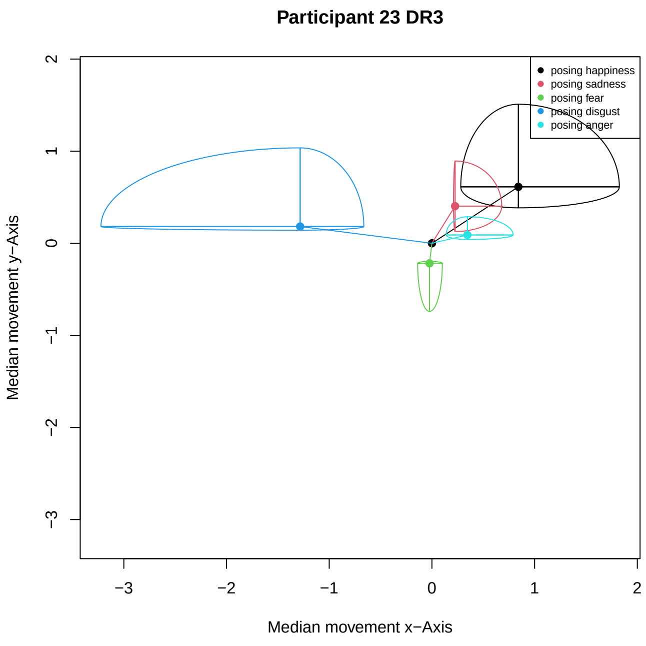
Participant 23 DR3
posing happiness
posing sadness
posing fear
posing disgust
posing anger
Median movement y−Axis
Median movement x−Axis

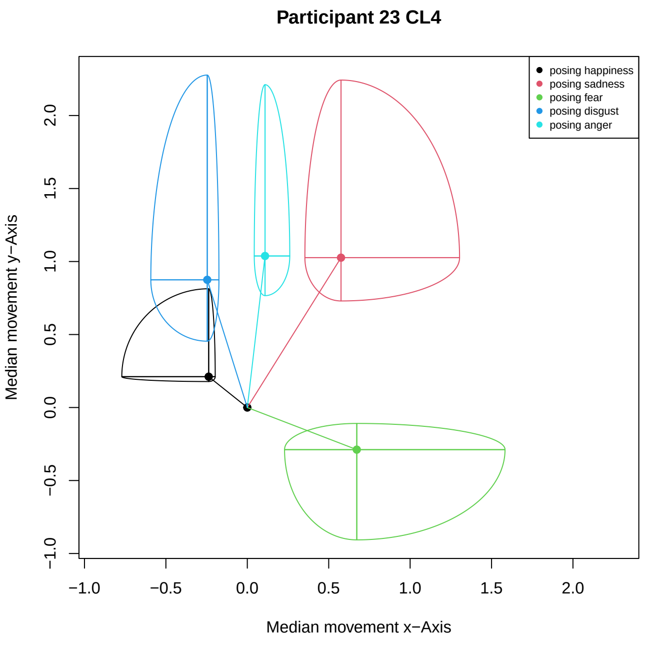
Participant 23 CL4
posing happiness
posing sadness
posing fear
posing disgust
posing anger
Median movement y−Axis
Median movement x−Axis

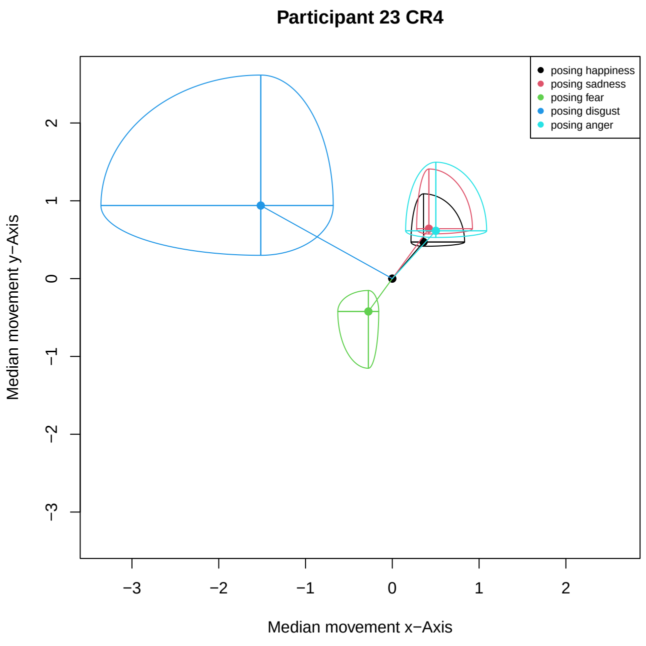
Participant 23 CR4
posing happiness
posing sadness
posing fear
posing disgust
posing anger
Median movement y−Axis
Median movement x−Axis

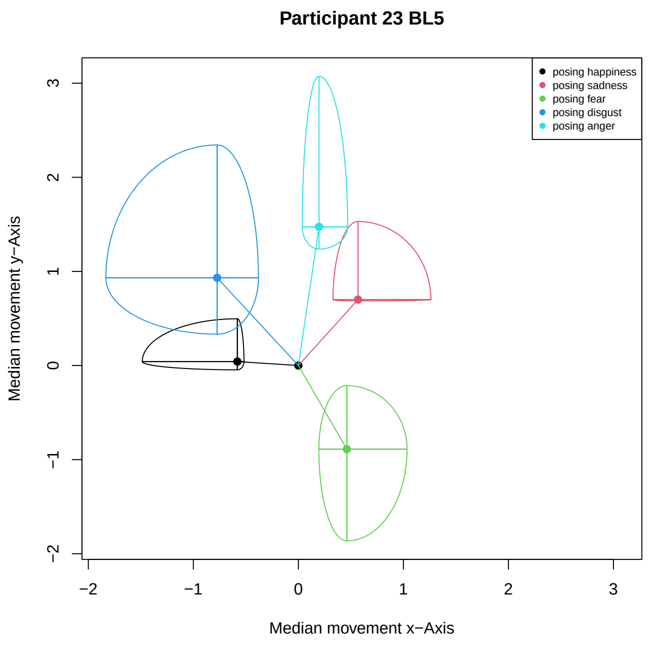
Participant 23 BL5
posing happiness
posing sadness
posing fear
posing disgust
posing anger
Median movement y−Axis
−2
−1
0
1
2
3
Median movement x−Axis
−2
−1
0
1
2
3

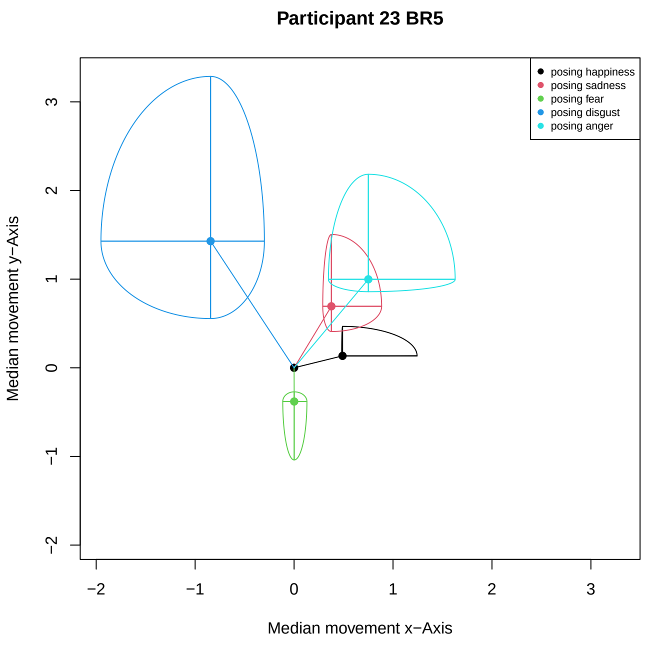
Participant 23 BR5
posing happiness
posing sadness
posing fear
posing disgust
posing anger
Median movement y−Axis
−2
−1
0
1
2
3
Median movement x−Axis
−2
−1
0
1
2
3

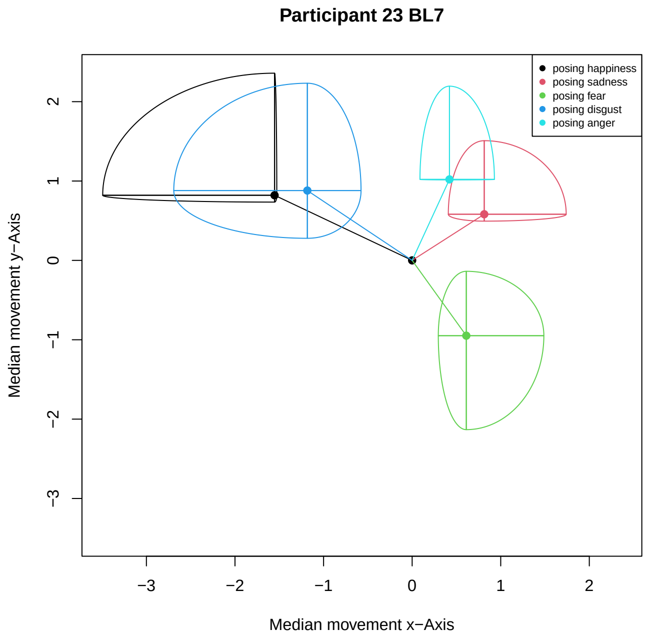
Participant 23 BL7
posing happiness
posing sadness
posing fear
posing disgust
posing anger
Median movement y−Axis
−3
−2
−1
0
1
2
Median movement x−Axis
−3
−2
−1
0
1
2

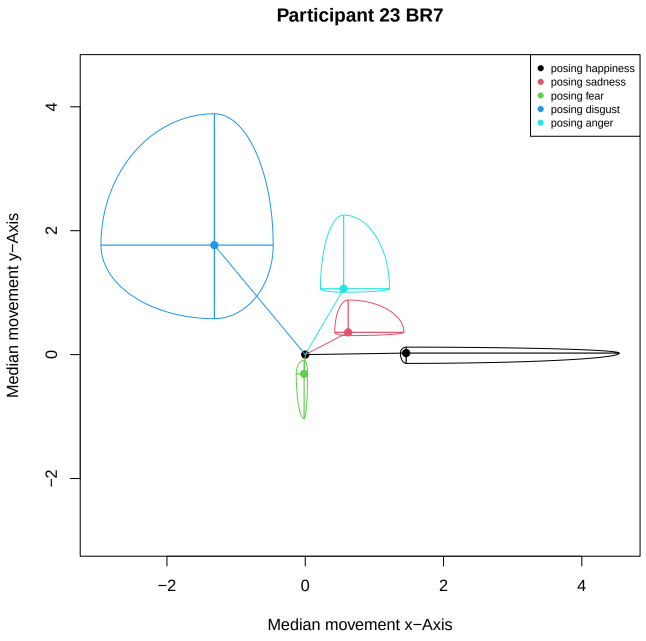
Participant 23 BR7
posing happiness
posing sadness
posing fear
posing disgust
posing anger
Median movement y−Axis
−2
0
2
4
Median movement x−Axis
−2
0
2
4

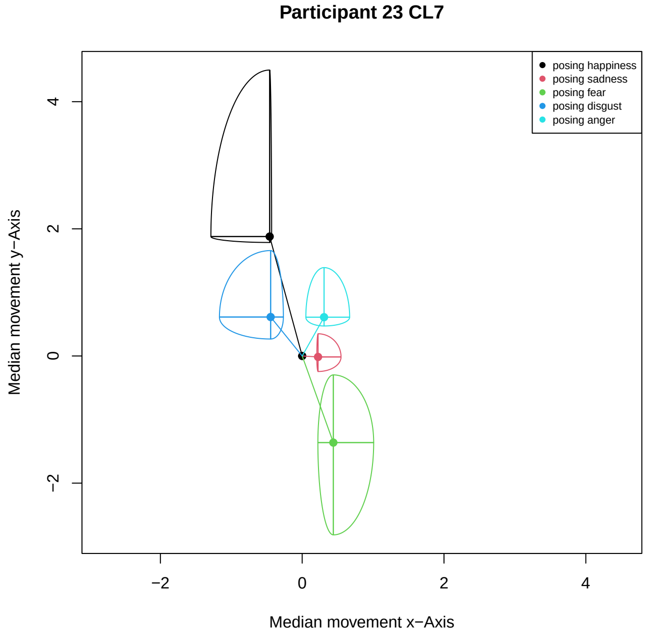
Participant 23 CL7
posing happiness
posing sadness
posing fear
posing disgust
posing anger
Median movement y−Axis
−2
0
2
4
Median movement x−Axis
−2
0
2
4

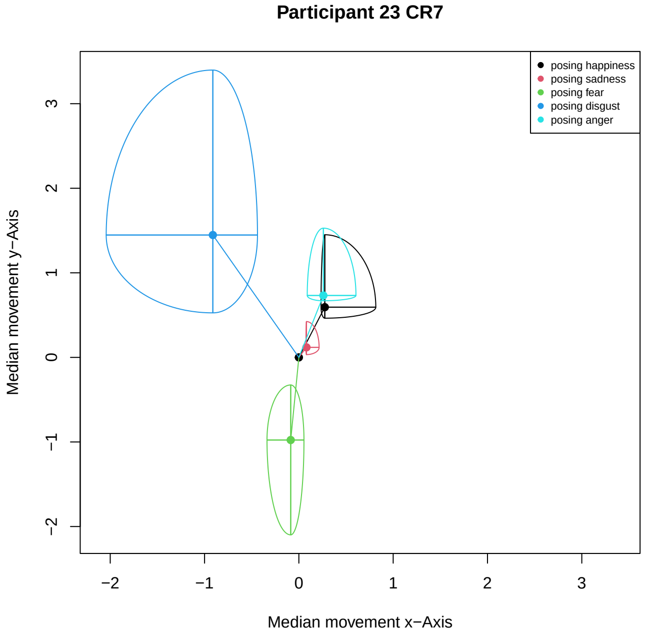
Participant 23 CR7
posing happiness
posing sadness
posing fear
posing disgust
posing anger
Median movement y−Axis
−2
−1
0
1
2
3
Median movement x−Axis
−2
−1
0
1
2
3

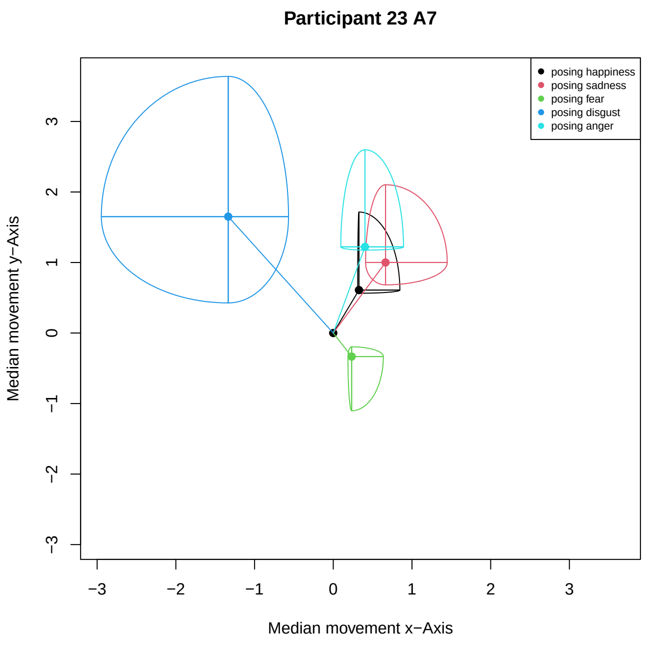
Participant 23 A7
posing happiness
posing sadness
posing fear
posing disgust
posing anger
Median movement y−Axis
Median movement x−Axis
−3
−2
−1
0
1
2
3

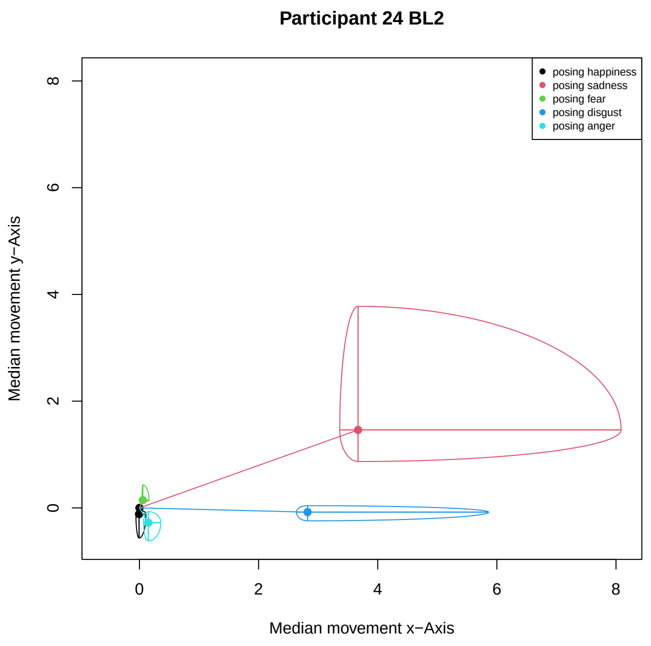
Participant 24 BL2
posing happiness
posing sadness
posing fear
posing disgust
posing anger
Median movement y−Axis
0 2 4 6 8
Median movement x−Axis
0 2 4 6 8

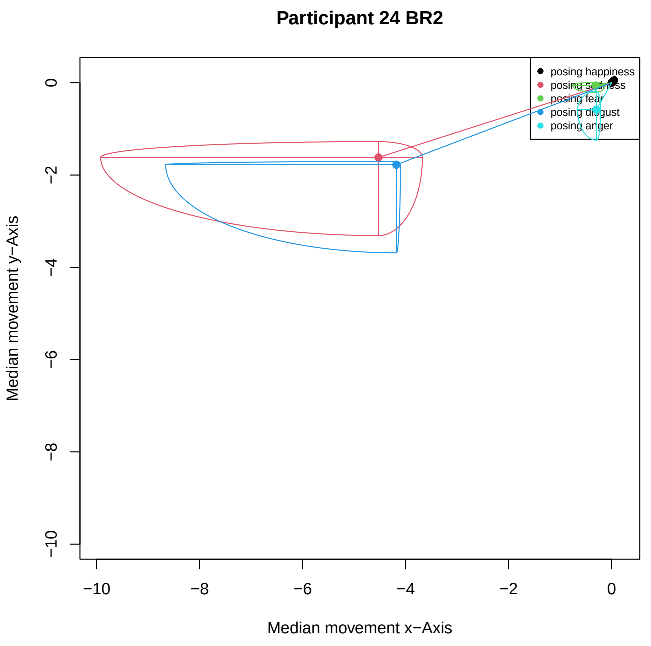
Participant 24 BR2
posing happiness
posing sadness
posing fear
posing disgust
posing anger
Median movement y−Axis
0 −2 −4 −6 −8 −10
Median movement x−Axis
−10 −8 −6 −4 −2 0

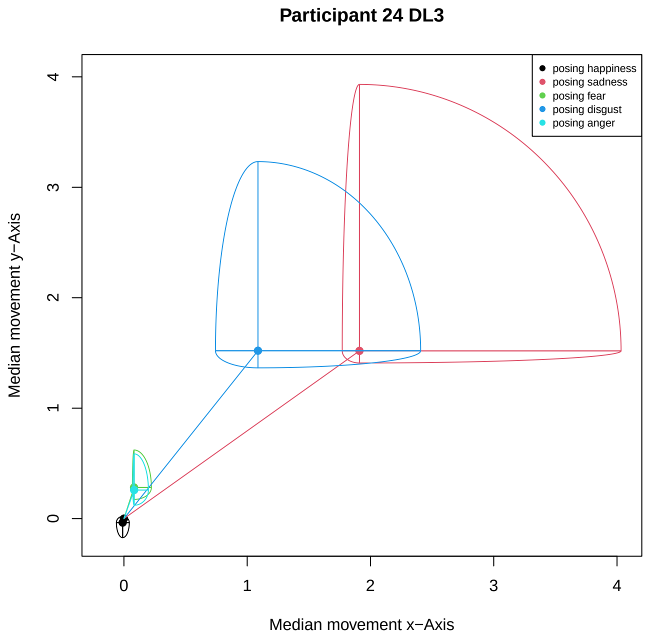
Participant 24 DL3
posing happiness
posing sadness
posing fear
posing disgust
posing anger
Median movement y−Axis
0 1 2 3 4
Median movement x−Axis
0 1 2 3 4

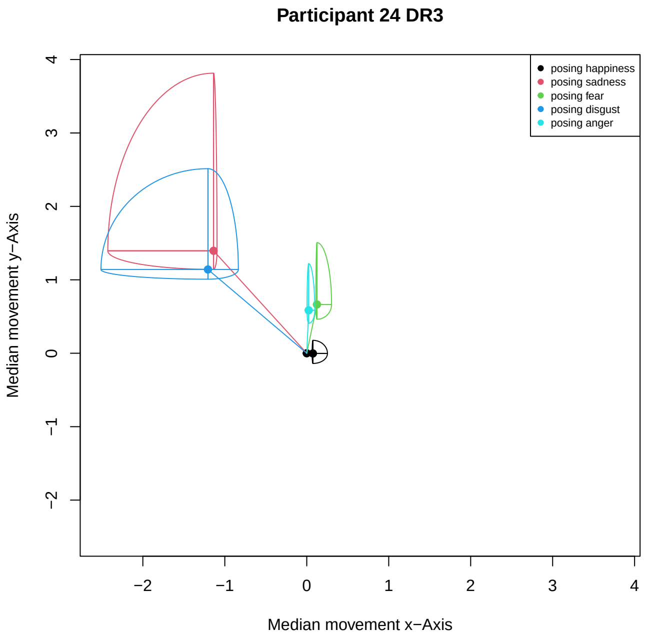
Participant 24 DR3
posing happiness
posing sadness
posing fear
posing disgust
posing anger
Median movement y−Axis
4 3 2 1 0 −1 −2
Median movement x−Axis
−2 −1 0 1 2 3 4

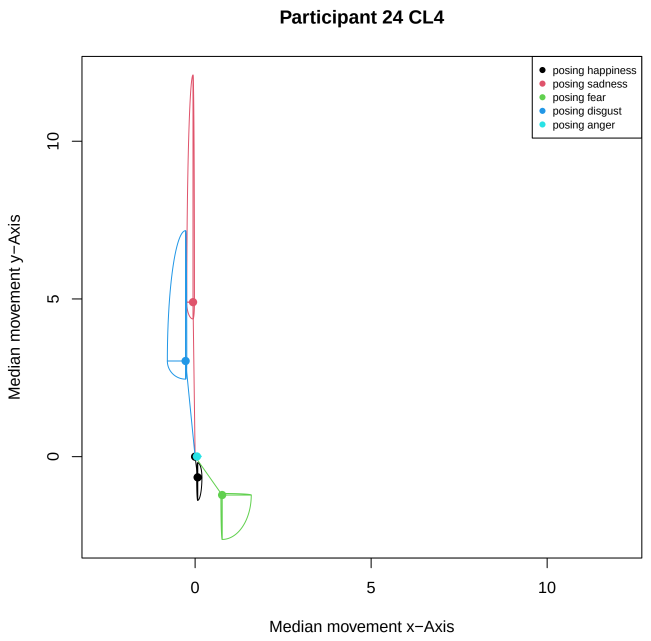
Participant 24 CL4
posing happiness
posing sadness
posing fear
posing disgust
posing anger
Median movement y−Axis
0 5 10
Median movement x−Axis
0 5 10

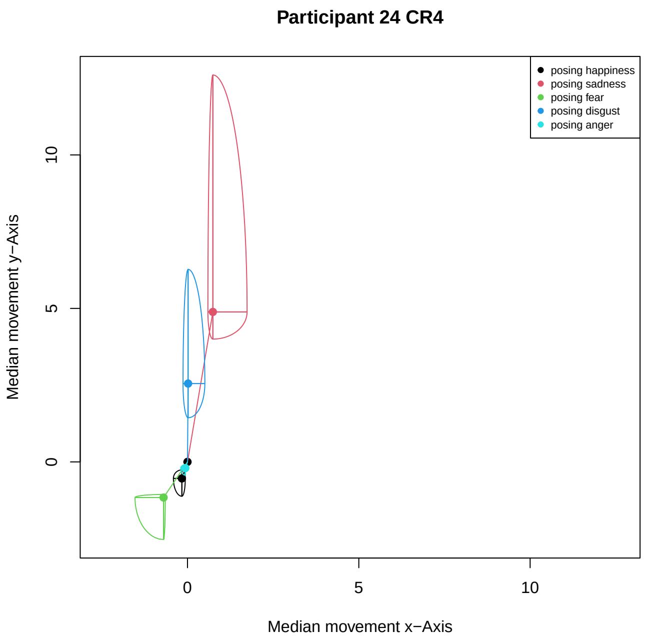
Participant 24 CR4
posing happiness
posing sadness
posing fear
posing disgust
posing anger
Median movement y−Axis
0 5 10
Median movement x−Axis
0 5 10

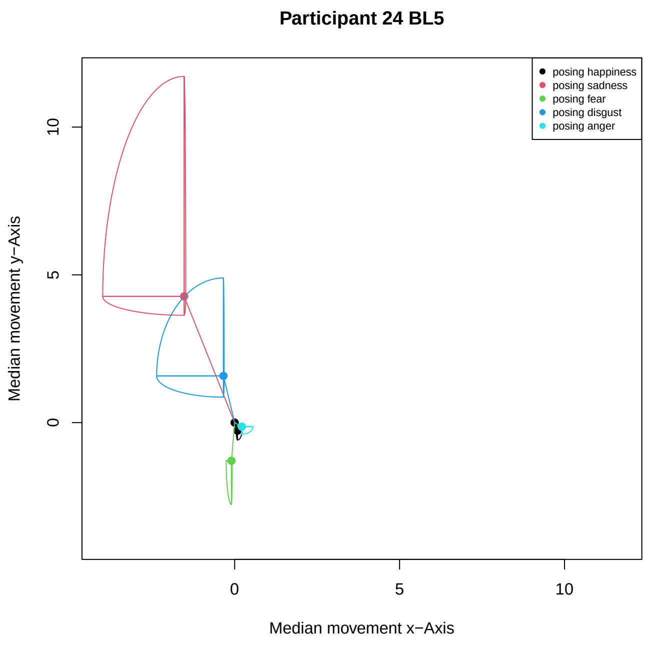
Participant 24 BL5
posing happiness
posing sadness
posing fear
posing disgust
posing anger
Median movement y−Axis
Median movement x−Axis

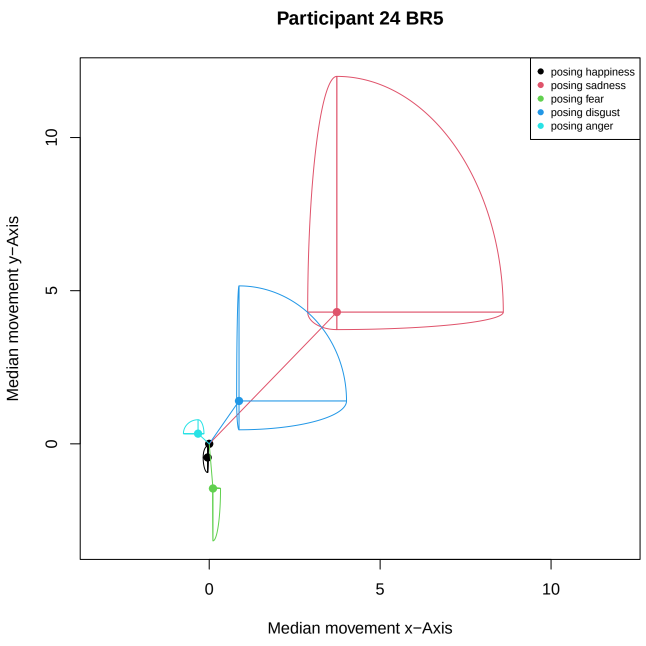
Participant 24 BR5
posing happiness
posing sadness
posing fear
posing disgust
posing anger
Median movement y−Axis
Median movement x−Axis

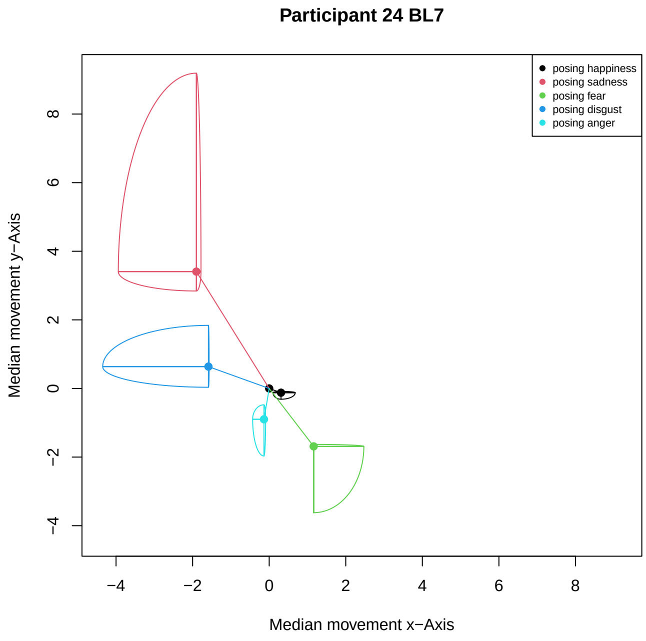
Participant 24 BL7
posing happiness
posing sadness
posing fear
posing disgust
posing anger
Median movement y−Axis
Median movement x−Axis

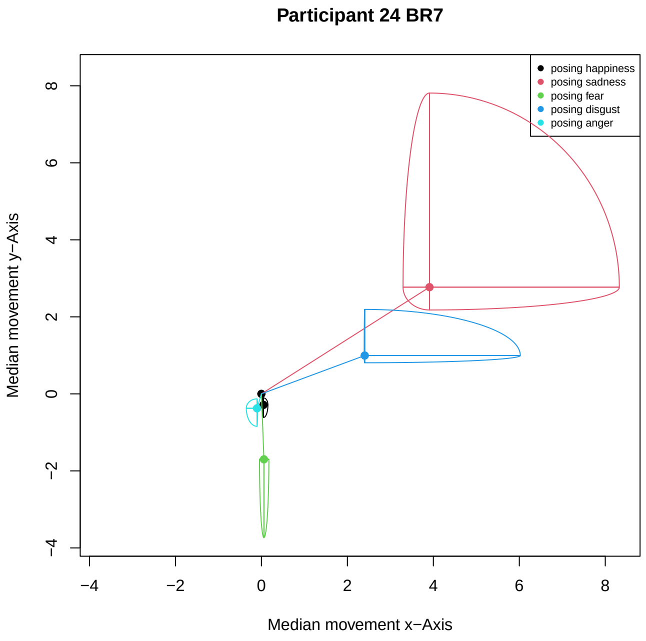
Participant 24 BR7
posing happiness
posing sadness
posing fear
posing disgust
posing anger
Median movement y−Axis
Median movement x−Axis

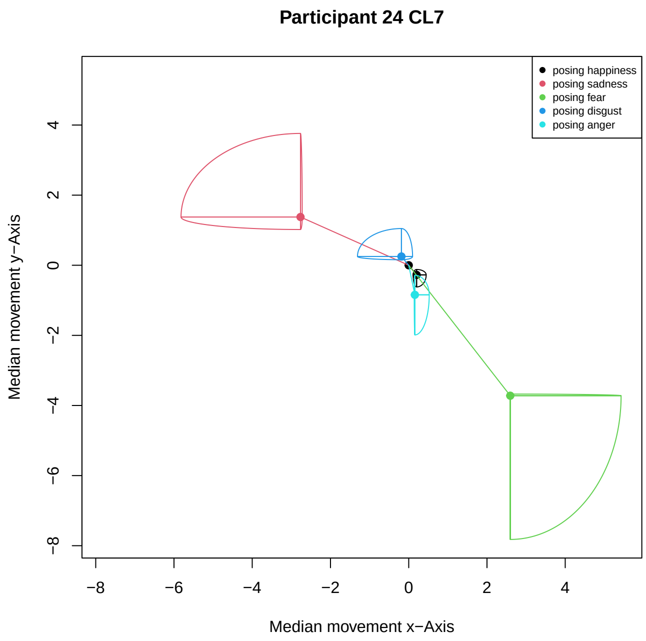
Participant 24 CL7
posing happiness
posing sadness
posing fear
posing disgust
posing anger
Median movement y−Axis
Median movement x−Axis

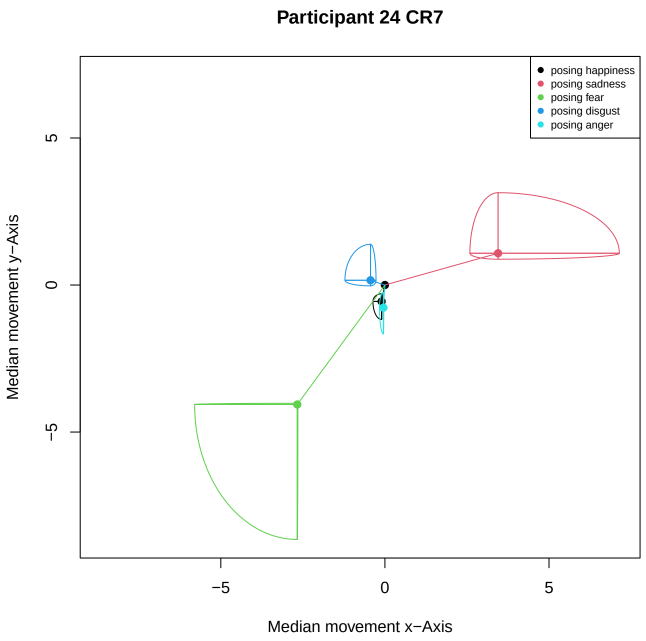
Participant 24 CR7
posing happiness
posing sadness
posing fear
posing disgust
posing anger
Median movement y−Axis
Median movement x−Axis

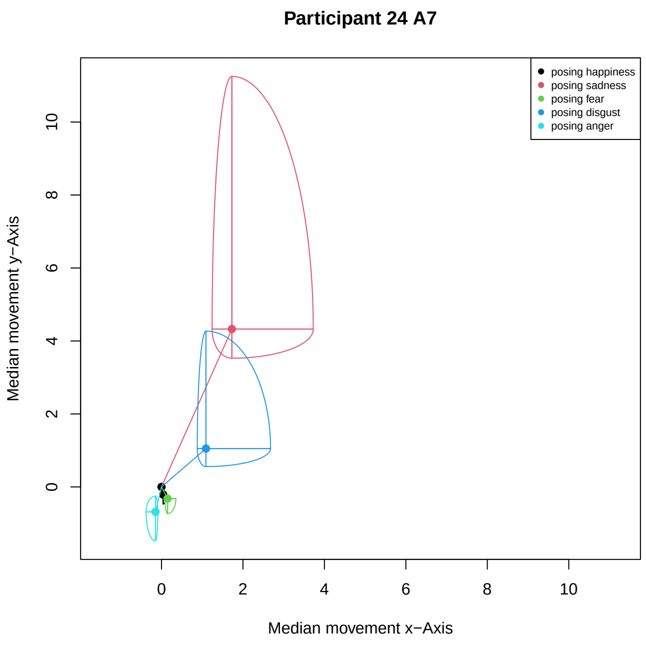

Participant 24 A7
posing happiness
posing sadness
posing fear
posing disgust
posing anger
Median movement y−Axis
Median movement x−Axis
0
2
4
6
8
10

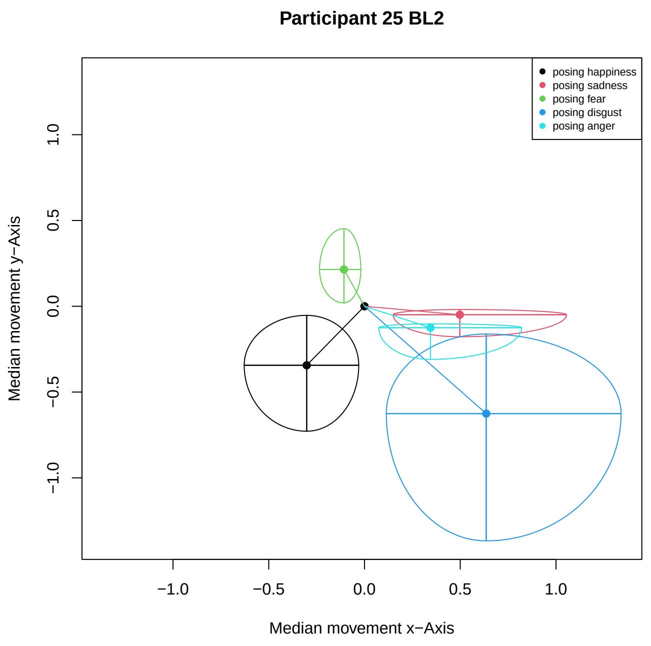
Participant 25 BL2
posing happiness
posing sadness
posing fear
posing disgust
posing anger
Median movement x−Axis
Median movement y−Axis

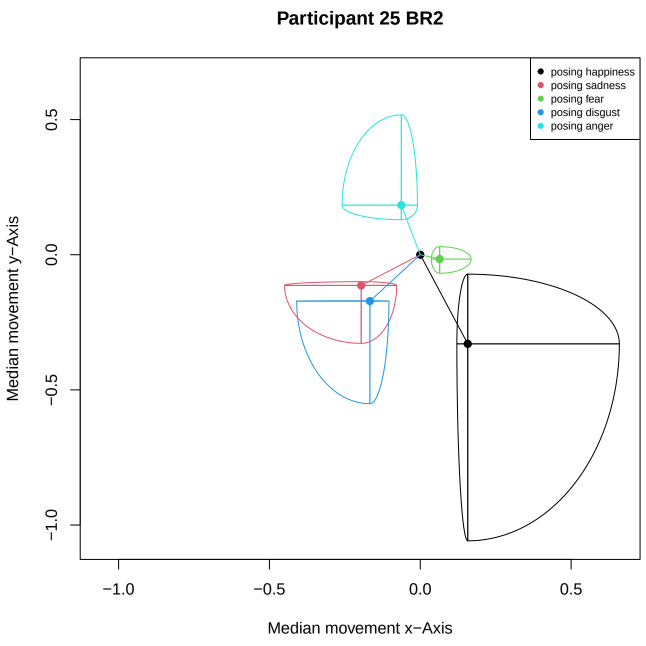
Participant 25 BR2
posing happiness
posing sadness
posing fear
posing disgust
posing anger
Median movement x−Axis
Median movement y−Axis

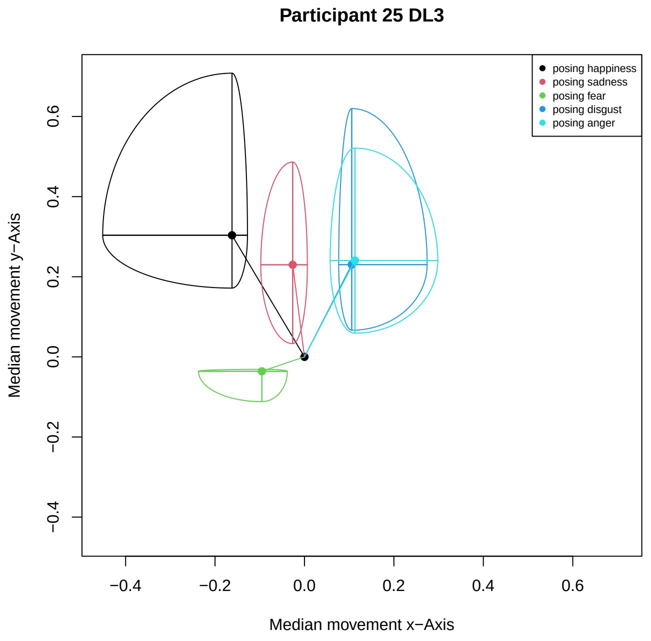
Participant 25 DL3
posing happiness
posing sadness
posing fear
posing disgust
posing anger
Median movement x−Axis
Median movement y−Axis

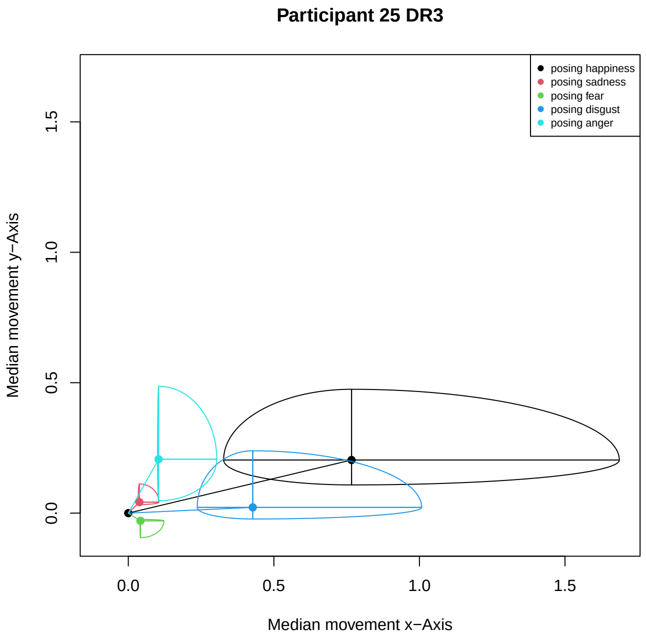
Participant 25 DR3
posing happiness
posing sadness
posing fear
posing disgust
posing anger
Median movement x−Axis
Median movement y−Axis

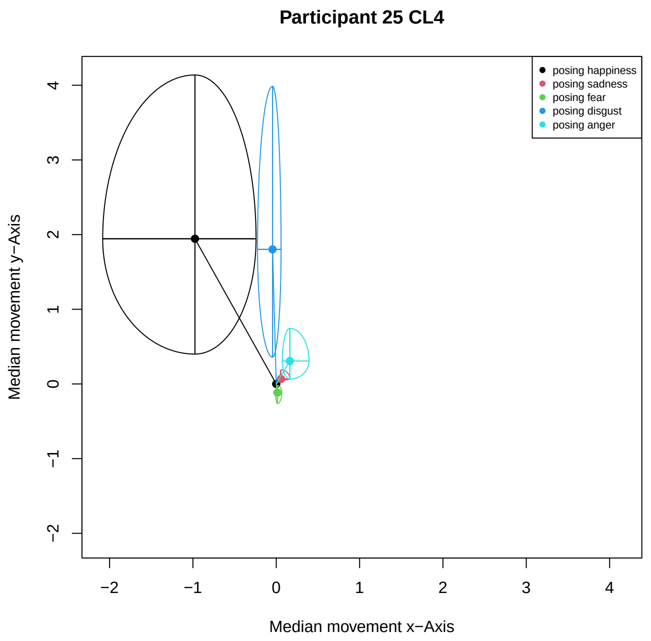
Participant 25 CL4
posing happiness
posing sadness
posing fear
posing disgust
posing anger
Median movement x−Axis
Median movement y−Axis

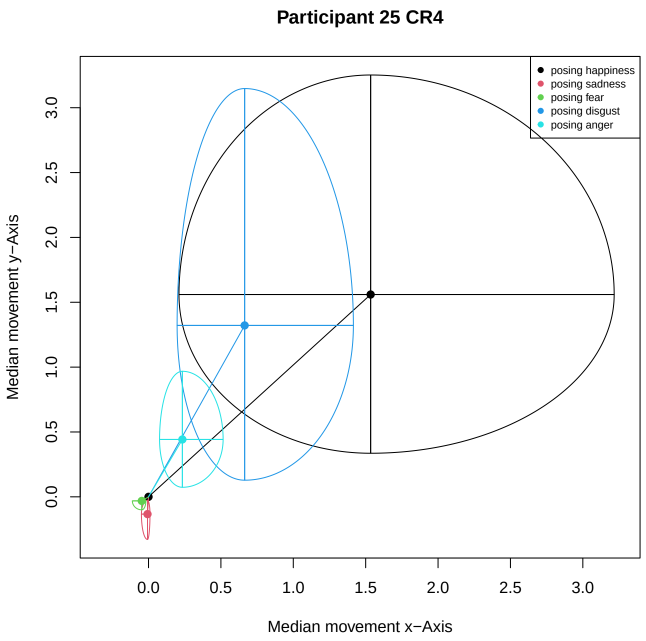
Participant 25 CR4
posing happiness
posing sadness
posing fear
posing disgust
posing anger
Median movement x−Axis
Median movement y−Axis

Participant 25 BL5

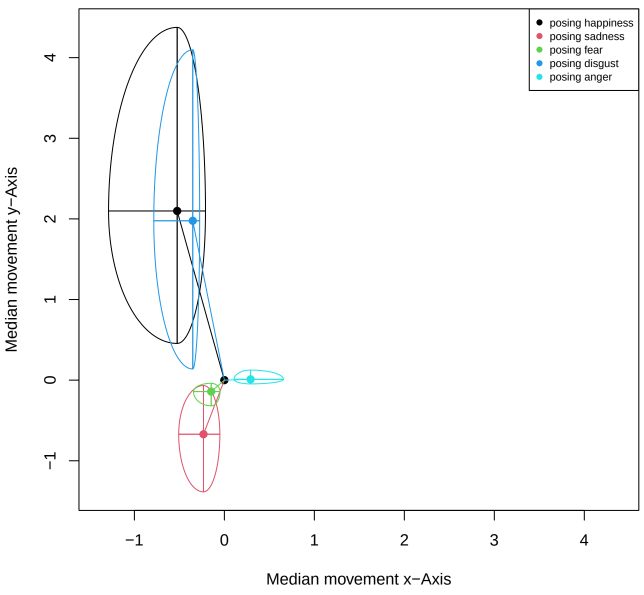

Participant 25 BR5

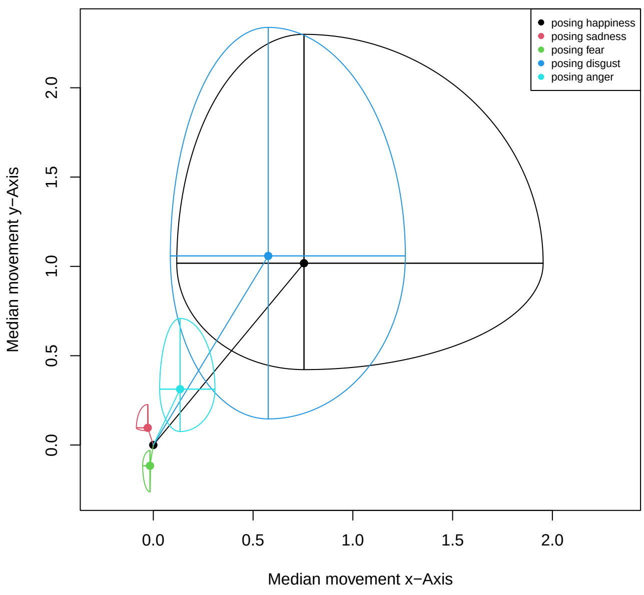

Participant 25 BL7

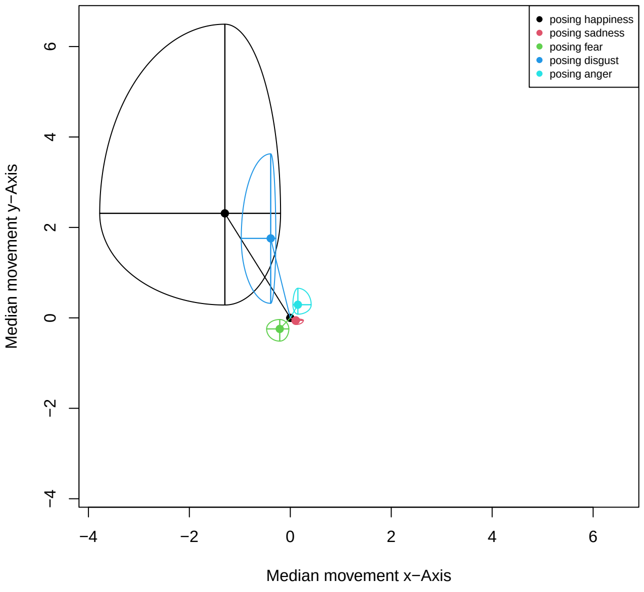

Participant 25 BR7

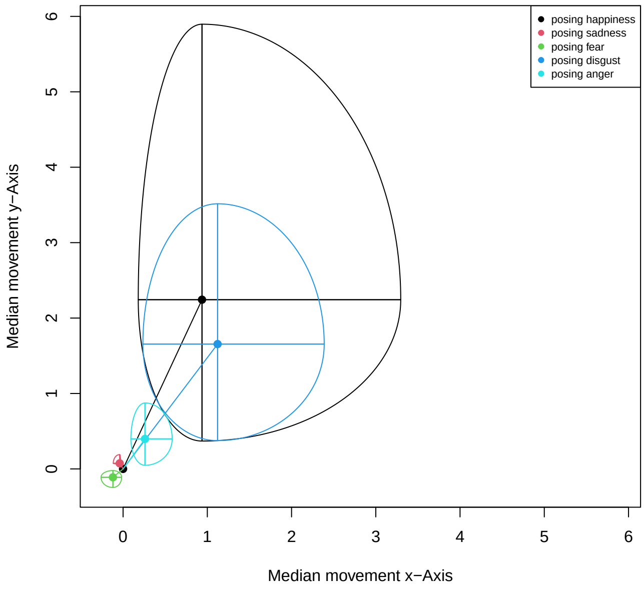

Participant 25 CL7

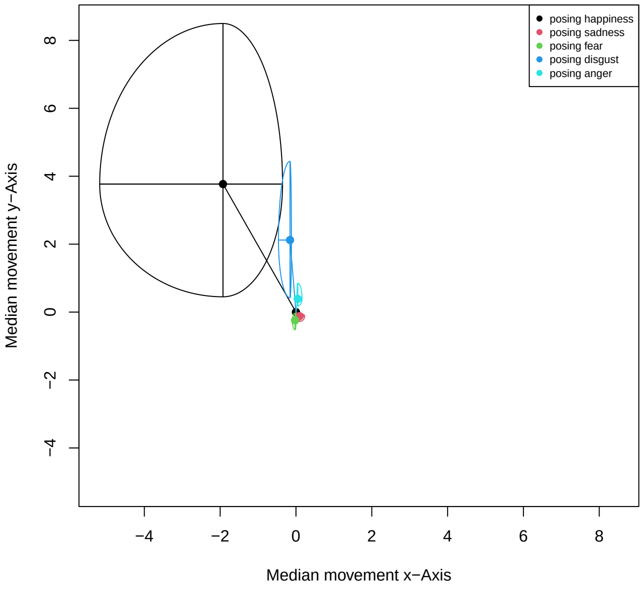

Participant 25 CR7

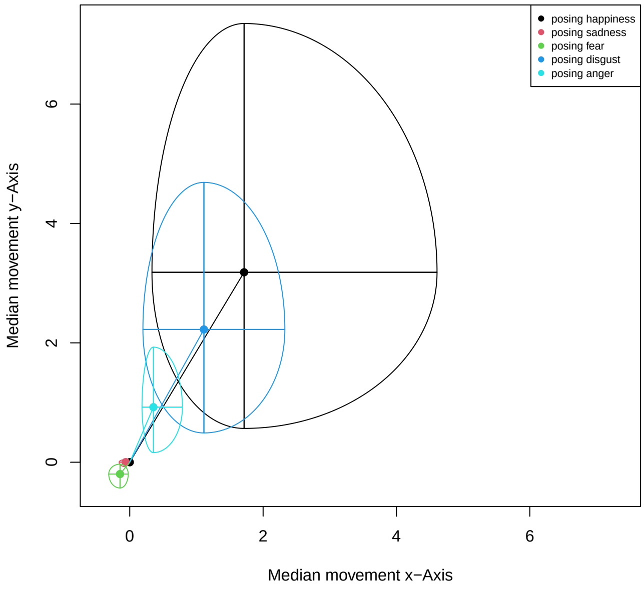

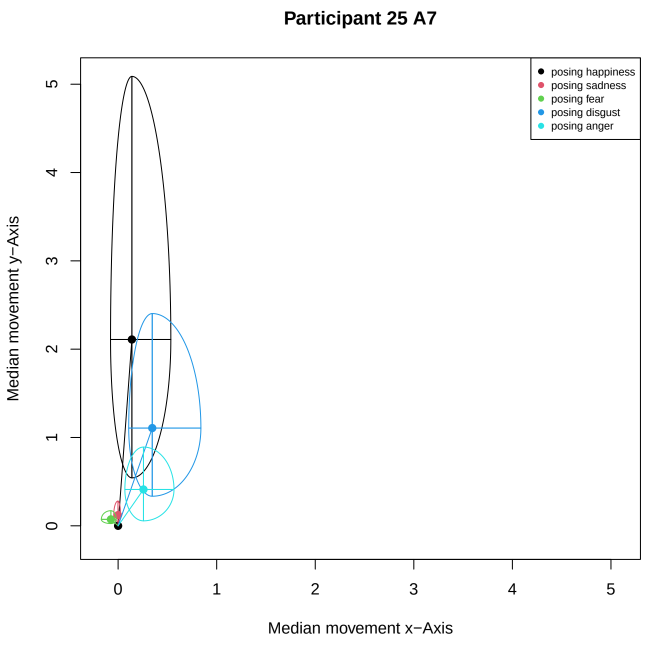

Participant 25 A7
posing happiness
posing sadness
posing fear
posing disgust
posing anger
Median movement y−Axis
Median movement x−Axis
0
1
2
3
4
5

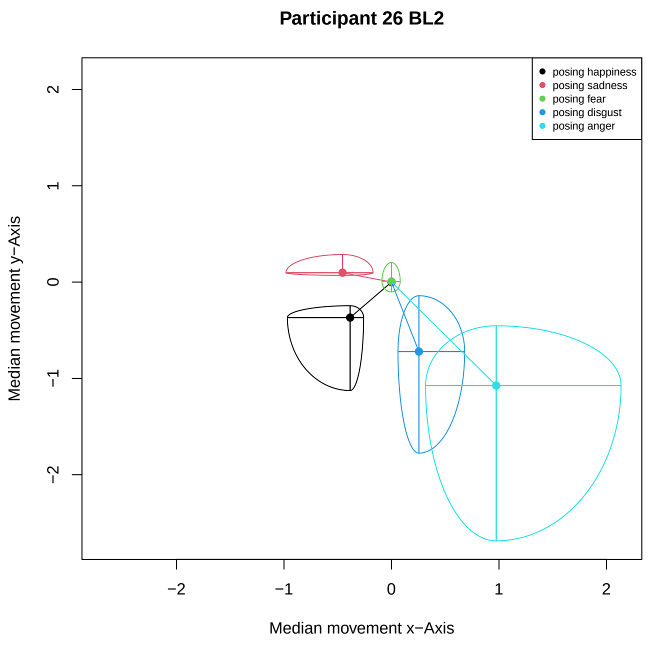
Participant 26 BL2
posing happiness
posing sadness
posing fear
posing disgust
posing anger
Median movement x−Axis
Median movement y−Axis

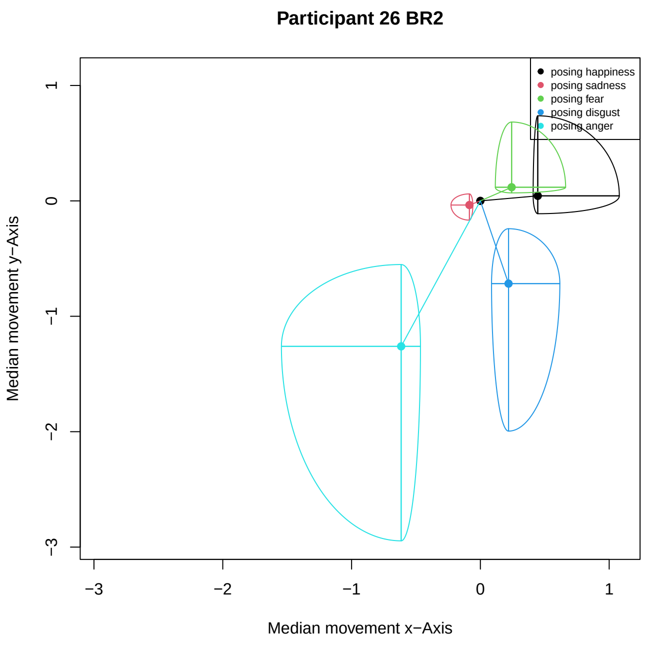
Participant 26 BR2
posing happiness
posing sadness
posing fear
posing disgust
posing anger
Median movement x−Axis
Median movement y−Axis

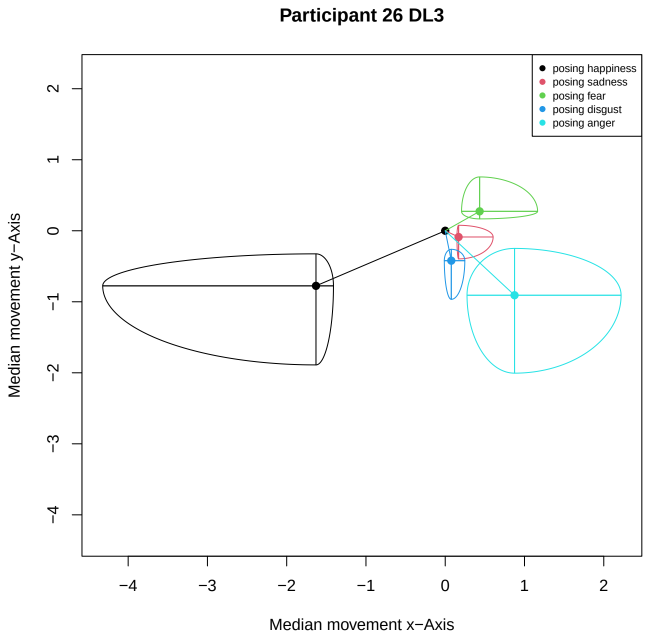
Participant 26 DL3
posing happiness
posing sadness
posing fear
posing disgust
posing anger
Median movement x−Axis
Median movement y−Axis

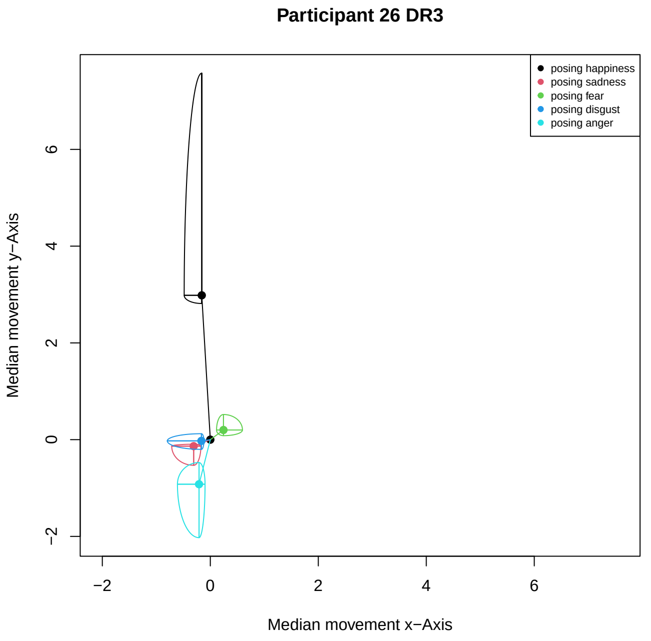
Participant 26 DR3
posing happiness
posing sadness
posing fear
posing disgust
posing anger
Median movement x−Axis
Median movement y−Axis

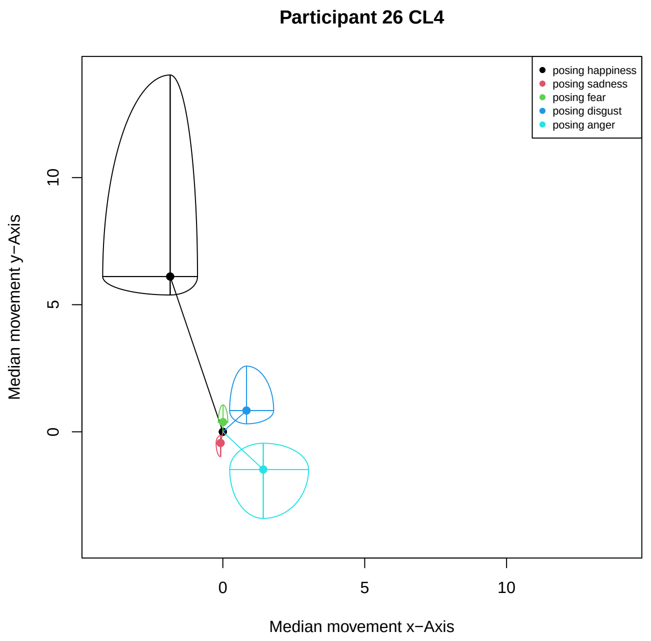
Participant 26 CL4
posing happiness
posing sadness
posing fear
posing disgust
posing anger
Median movement x−Axis
Median movement y−Axis

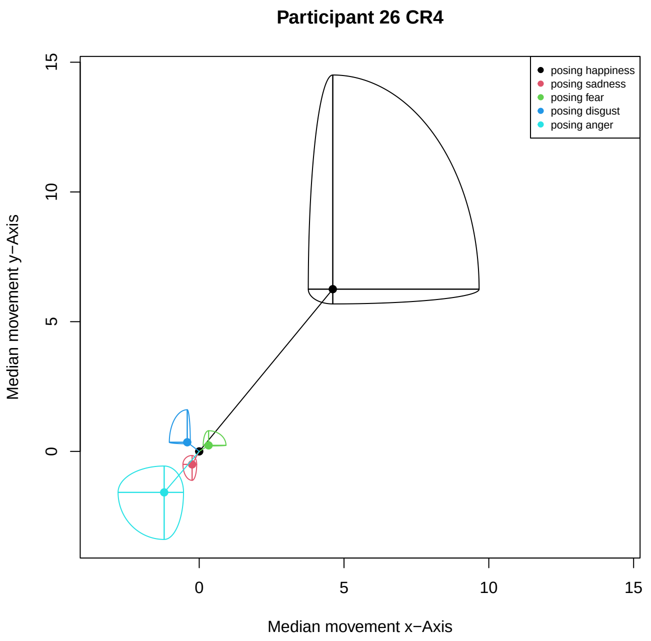
Participant 26 CR4
posing happiness
posing sadness
posing fear
posing disgust
posing anger
Median movement x−Axis
Median movement y−Axis

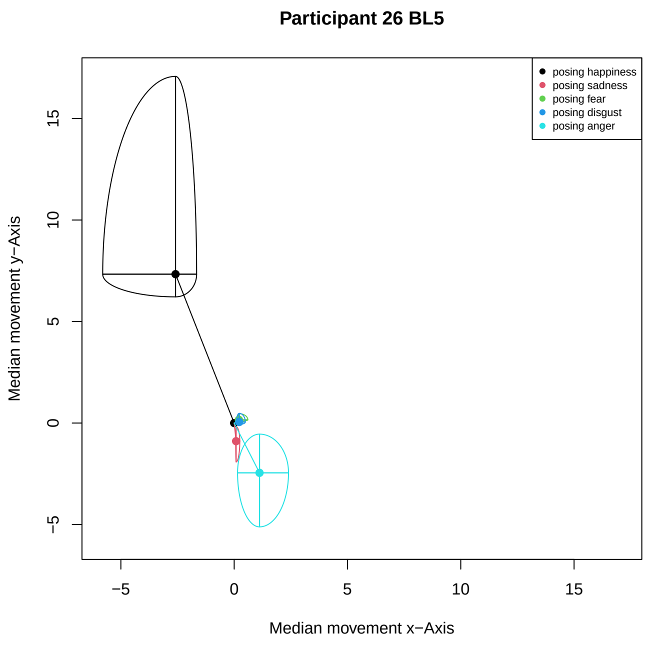
Participant 26 BL5
posing happiness
posing sadness
posing fear
posing disgust
posing anger
Median movement y−Axis
Median movement x−Axis

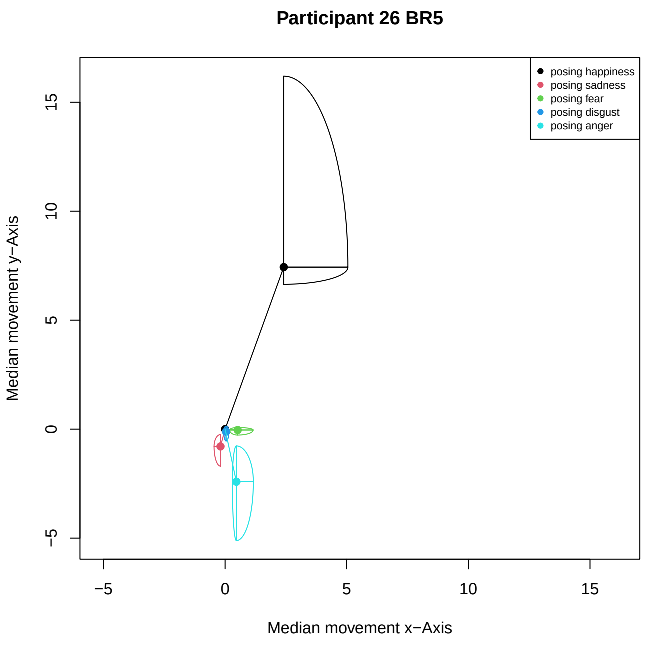
Participant 26 BR5
posing happiness
posing sadness
posing fear
posing disgust
posing anger
Median movement y−Axis
Median movement x−Axis

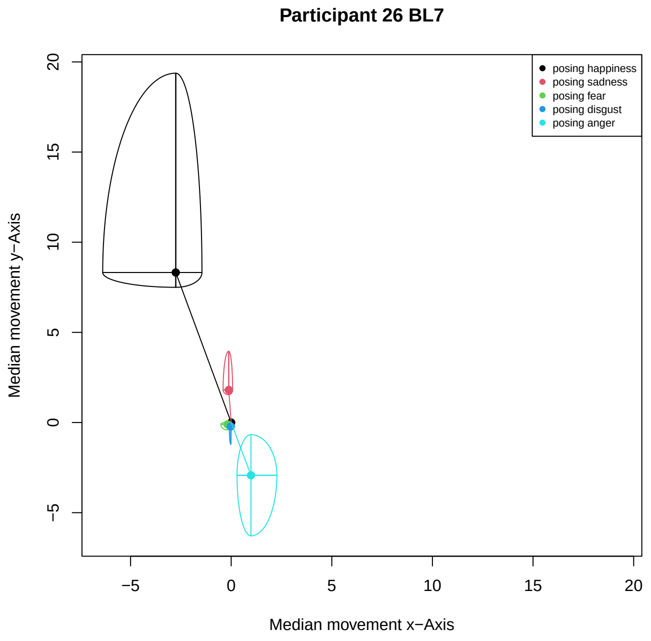
Participant 26 BL7
posing happiness
posing sadness
posing fear
posing disgust
posing anger
Median movement y−Axis
Median movement x−Axis

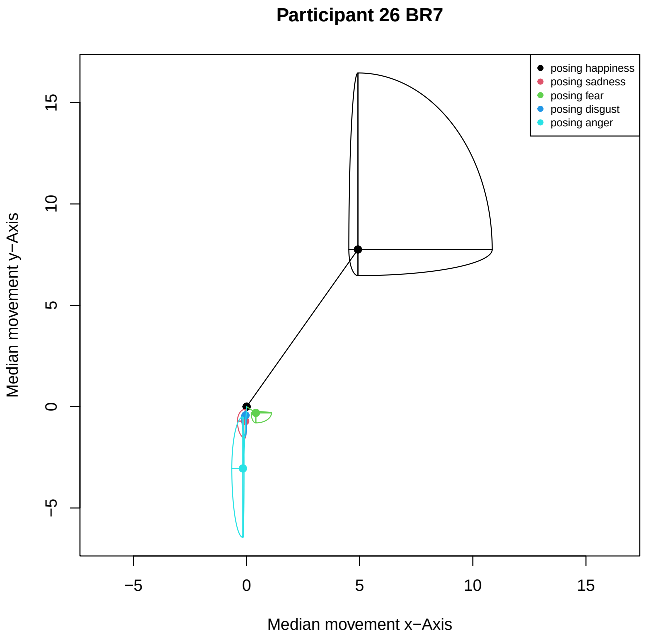
Participant 26 BR7
posing happiness
posing sadness
posing fear
posing disgust
posing anger
Median movement y−Axis
Median movement x−Axis

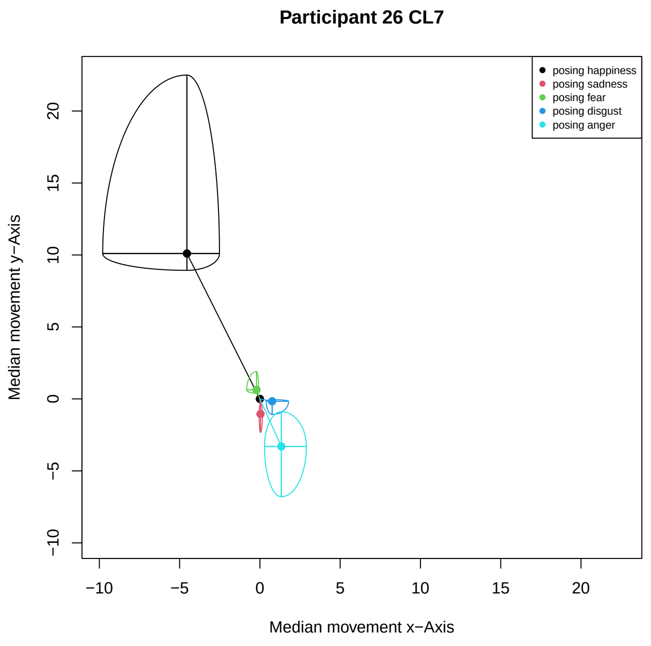
Participant 26 CL7
posing happiness
posing sadness
posing fear
posing disgust
posing anger
Median movement y−Axis
Median movement x−Axis

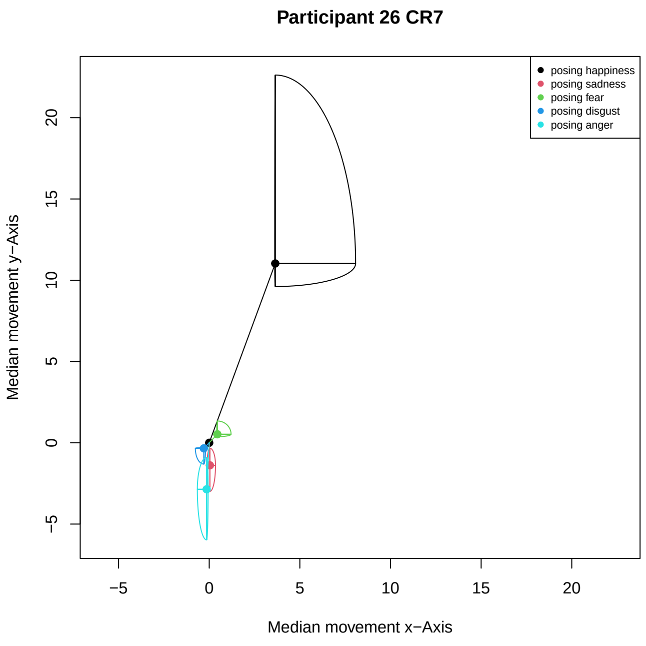
Participant 26 CR7
posing happiness
posing sadness
posing fear
posing disgust
posing anger
Median movement y−Axis
Median movement x−Axis

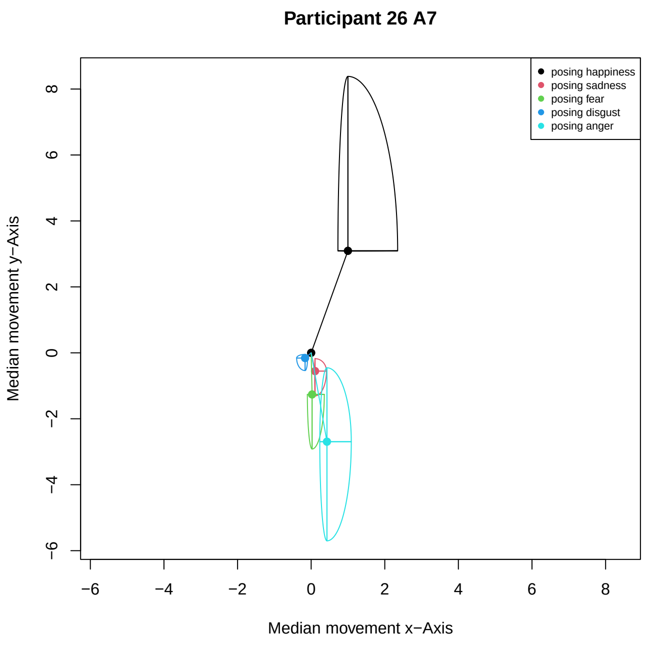

Participant 26 A7
posing happiness
posing sadness
posing fear
posing disgust
posing anger
Median movement y−Axis
Median movement x−Axis
−6
−4
−2
0
2
4
6
8

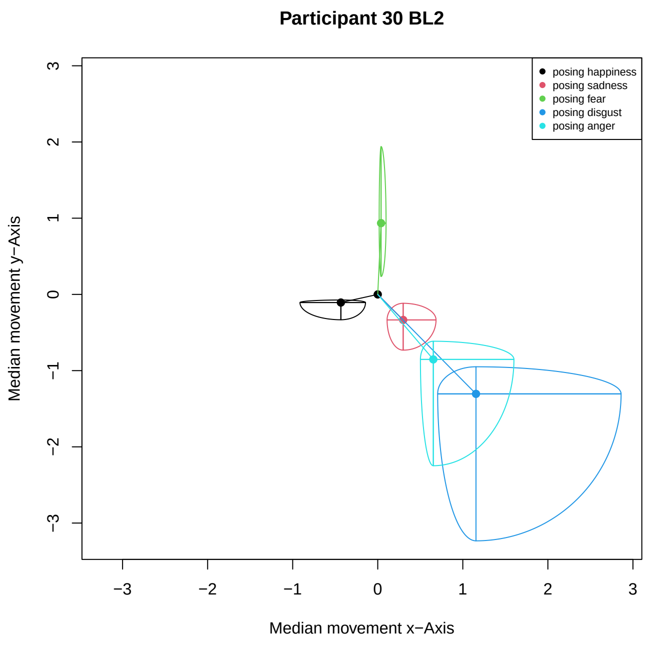
Participant 30 BL2
posing happiness
posing sadness
posing fear
posing disgust
posing anger
Median movement x−Axis
Median movement y−Axis

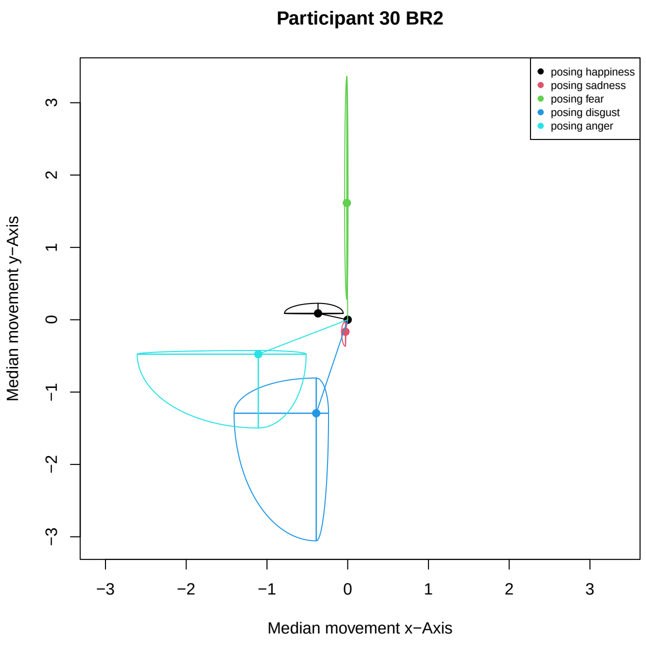
Participant 30 BR2
posing happiness
posing sadness
posing fear
posing disgust
posing anger
Median movement x−Axis
Median movement y−Axis

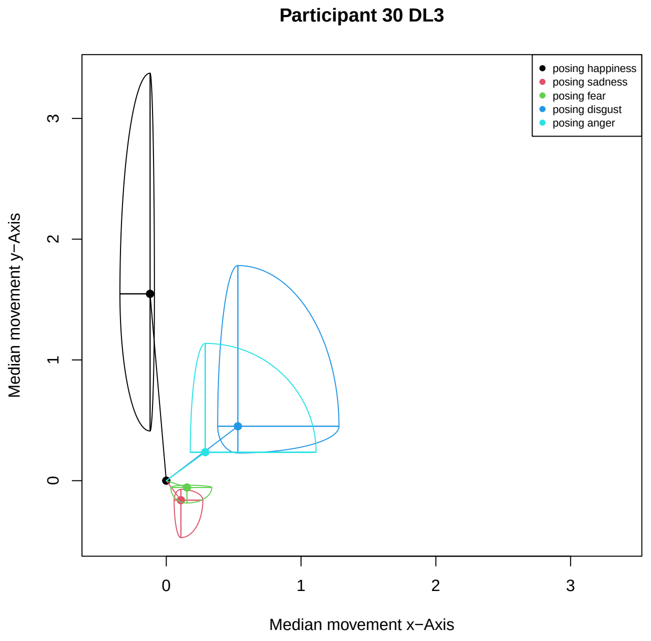
Participant 30 DL3
posing happiness
posing sadness
posing fear
posing disgust
posing anger
Median movement x−Axis
Median movement y−Axis

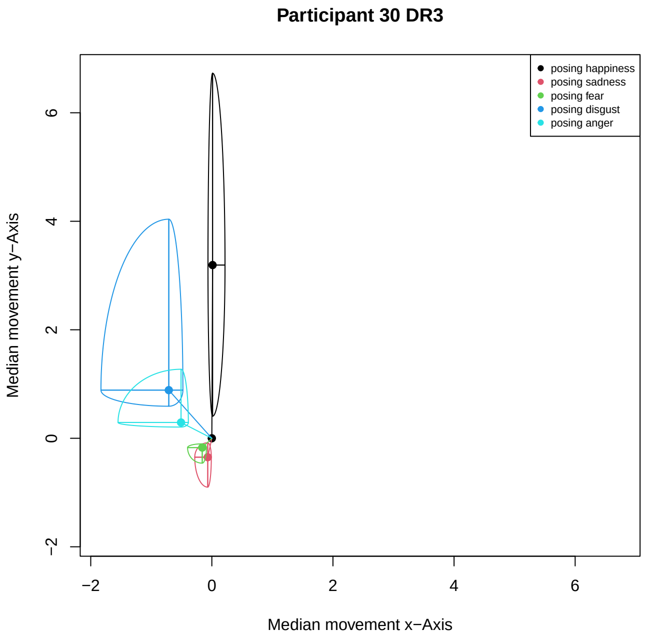
Participant 30 DR3
posing happiness
posing sadness
posing fear
posing disgust
posing anger
Median movement x−Axis
Median movement y−Axis

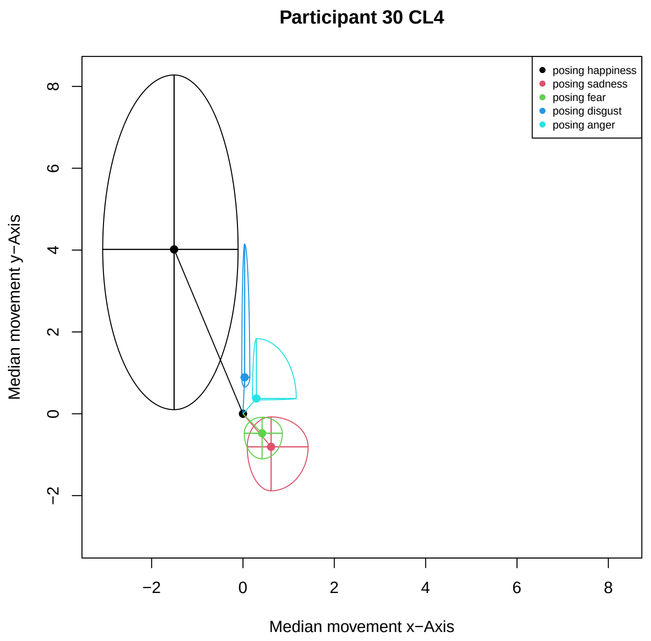
Participant 30 CL4
posing happiness
posing sadness
posing fear
posing disgust
posing anger
Median movement x−Axis
Median movement y−Axis

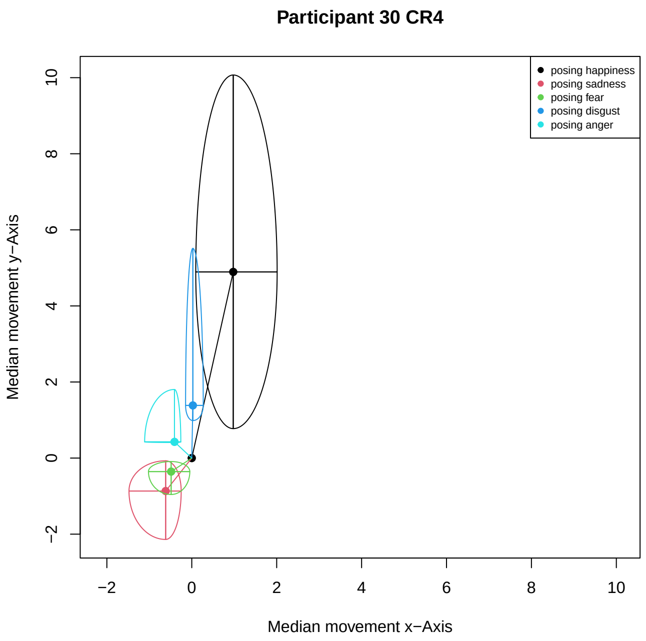
Participant 30 CR4
posing happiness
posing sadness
posing fear
posing disgust
posing anger
Median movement x−Axis
Median movement y−Axis

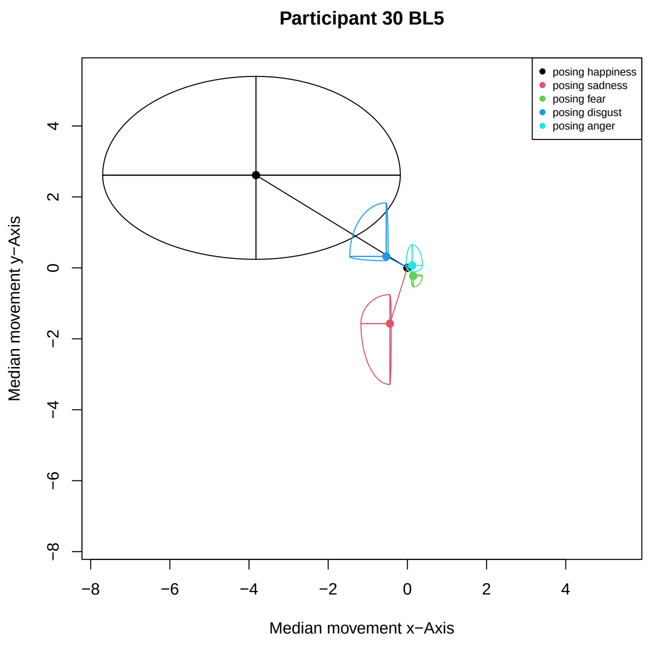
Participant 30 BL5
posing happiness
posing sadness
posing fear
posing disgust
posing anger
Median movement y−Axis
Median movement x−Axis

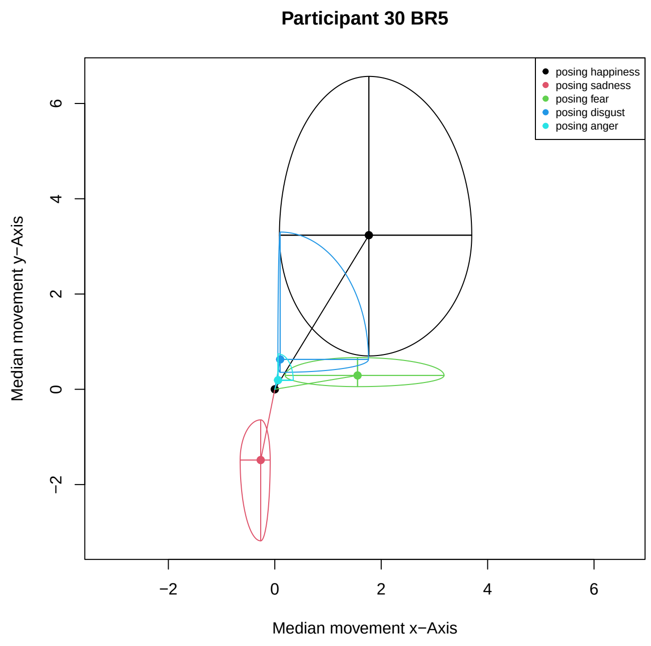
Participant 30 BR5
posing happiness
posing sadness
posing fear
posing disgust
posing anger
Median movement y−Axis
Median movement x−Axis

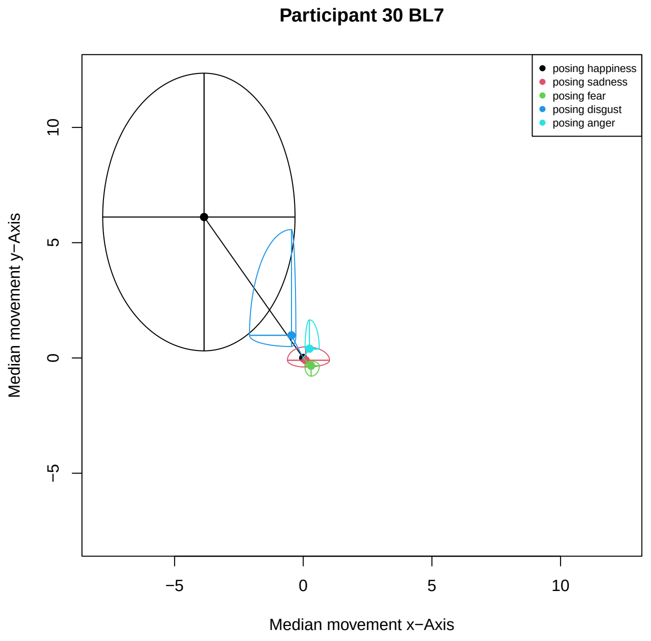
Participant 30 BL7
posing happiness
posing sadness
posing fear
posing disgust
posing anger
Median movement y−Axis
Median movement x−Axis

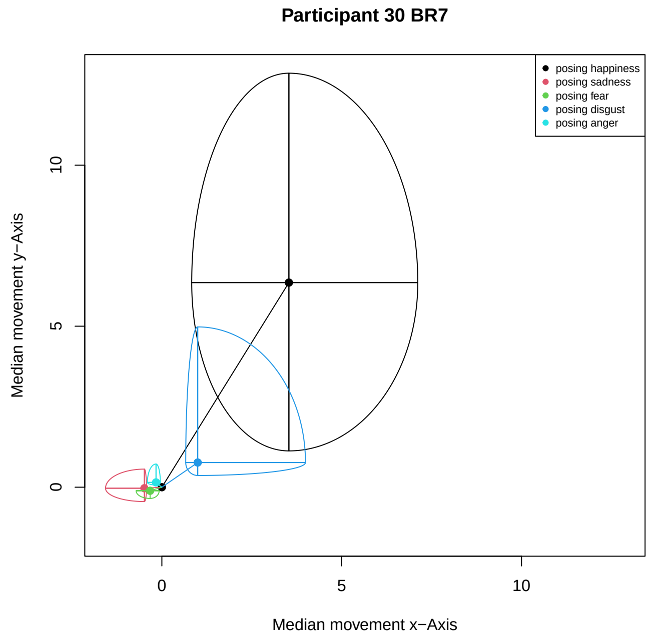
Participant 30 BR7
posing happiness
posing sadness
posing fear
posing disgust
posing anger
Median movement y−Axis
Median movement x−Axis

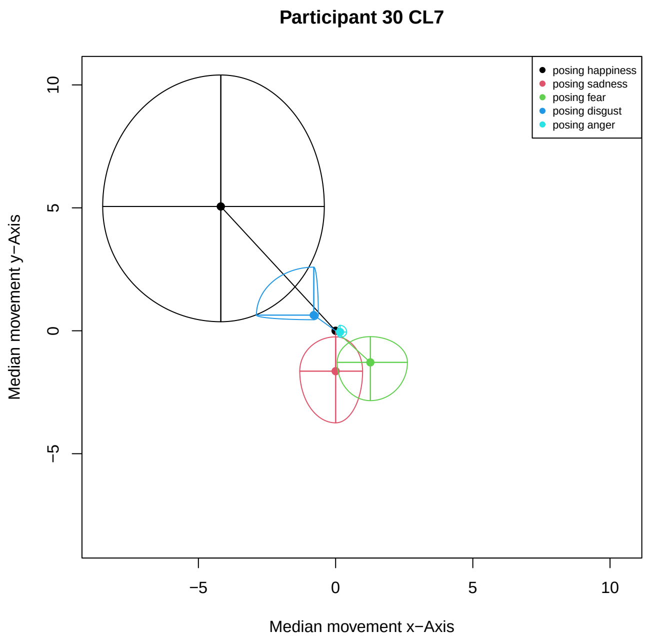
Participant 30 CL7
posing happiness
posing sadness
posing fear
posing disgust
posing anger
Median movement y−Axis
Median movement x−Axis

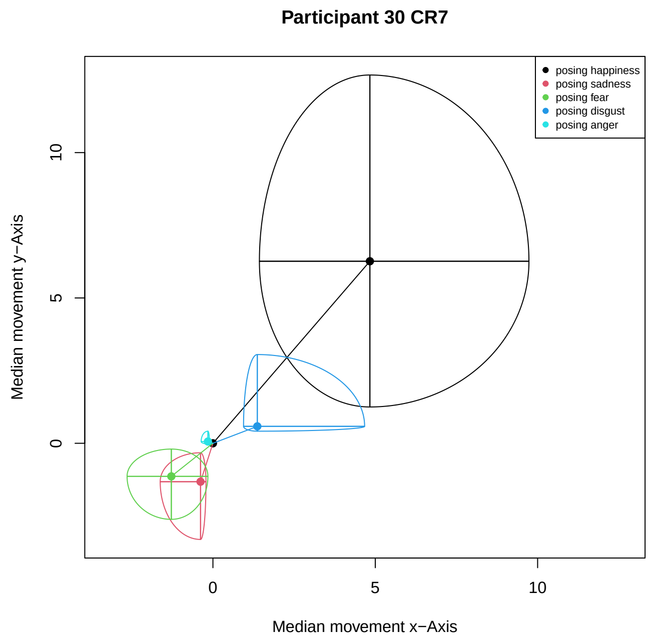
Participant 30 CR7
posing happiness
posing sadness
posing fear
posing disgust
posing anger
Median movement y−Axis
Median movement x−Axis

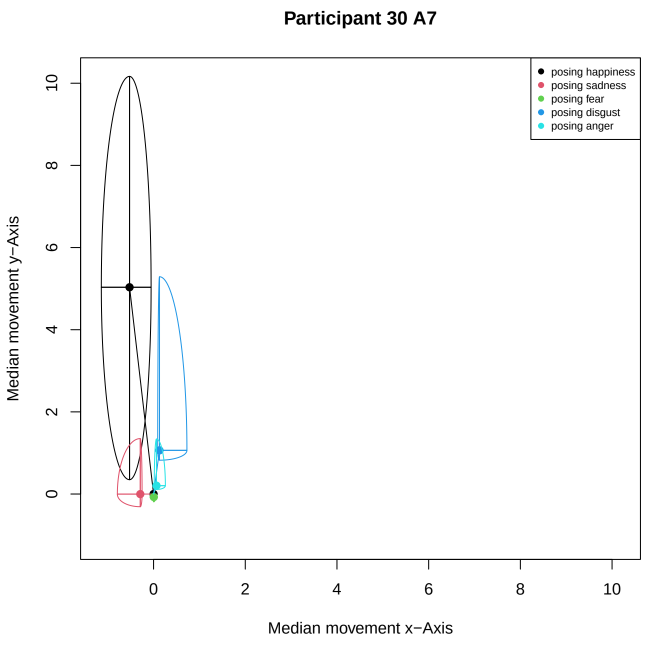
Participant 30 A7
posing happiness
posing sadness
posing fear
posing disgust
posing anger
Median movement y−Axis
Median movement x−Axis
0
2
4
6
8
10

Participant 31 BL2

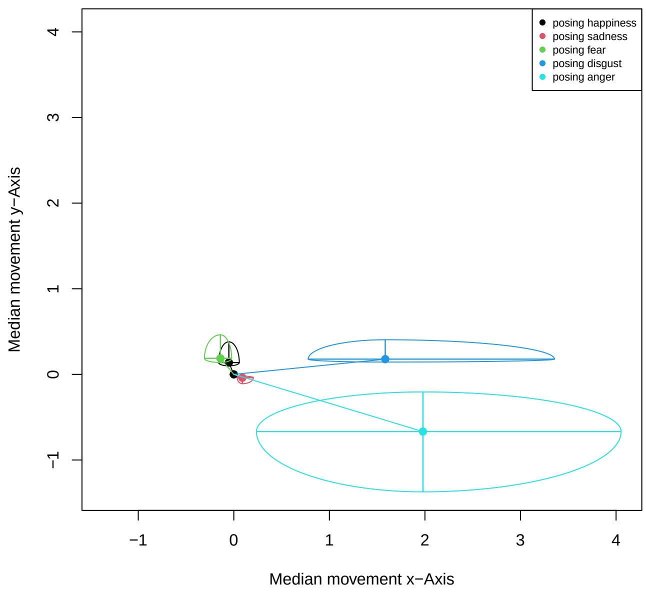

Participant 31 BR2

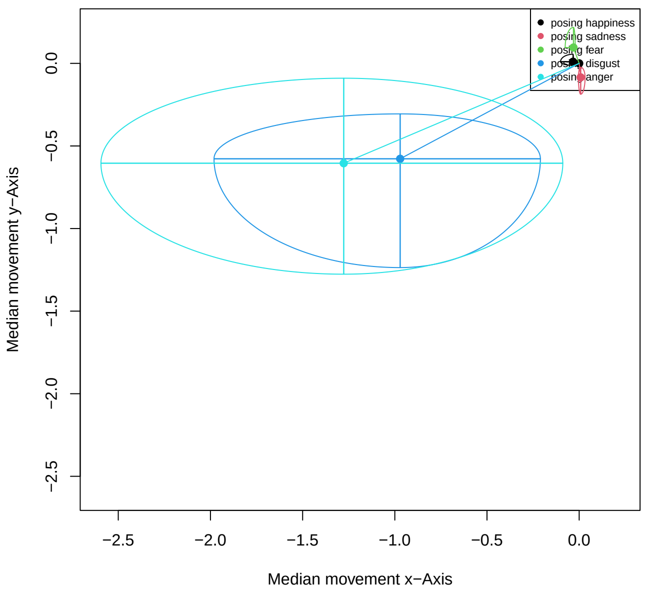

Participant 31 DL3

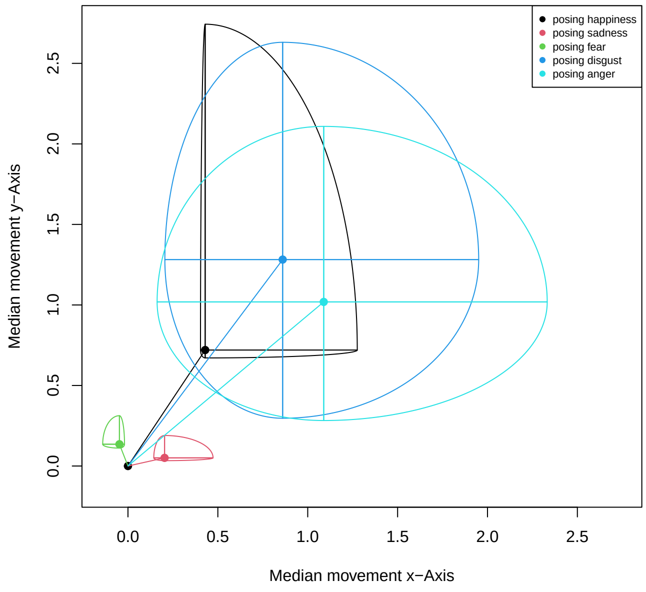

Participant 31 DR3

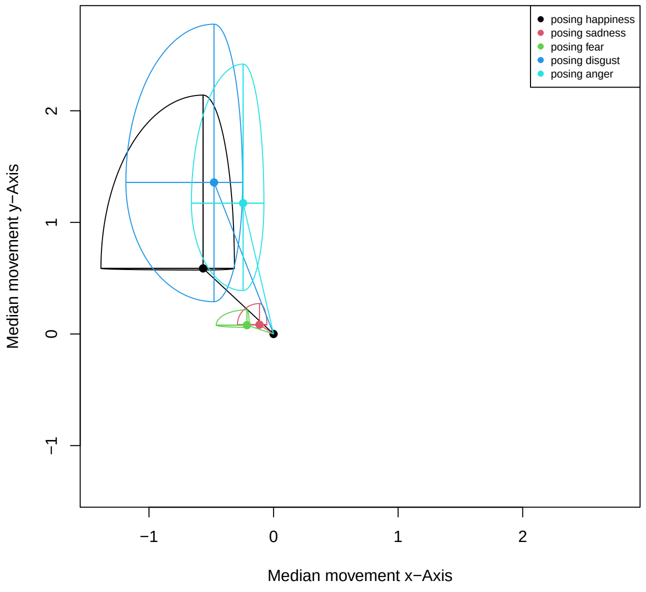

Participant 31 CL4

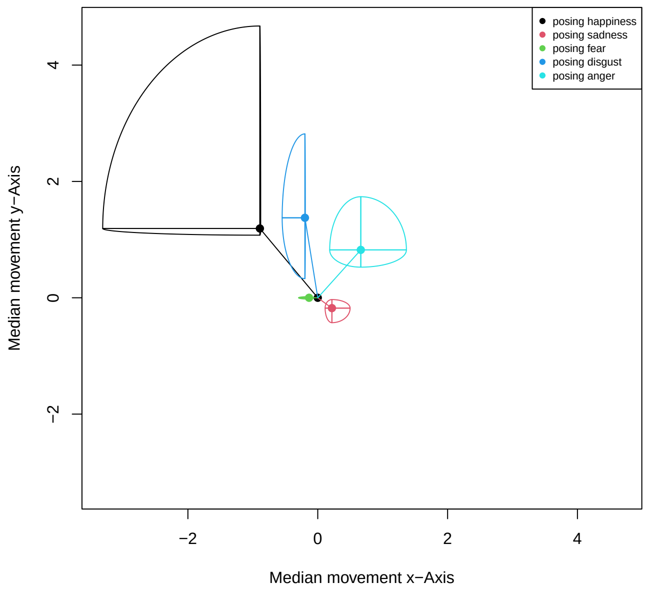

Participant 31 CR4

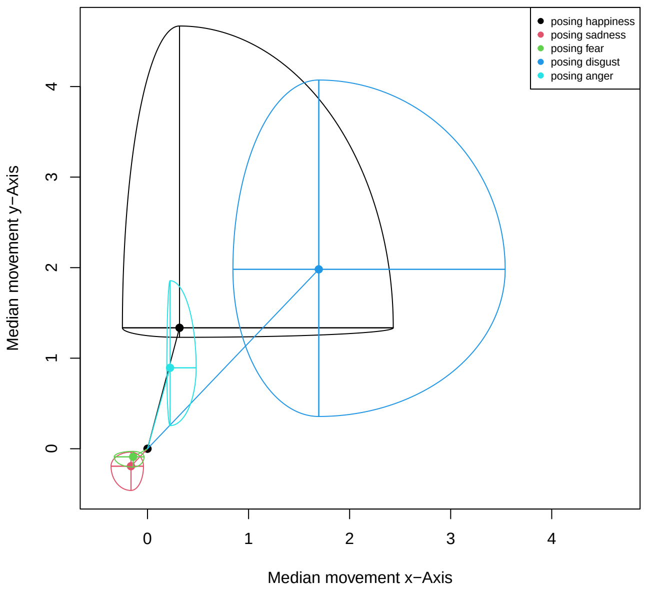

Participant 31 BL5

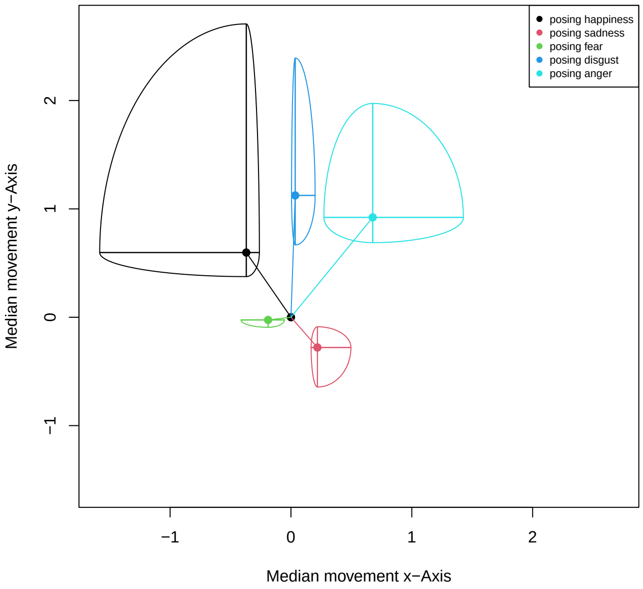

Participant 31 BR5

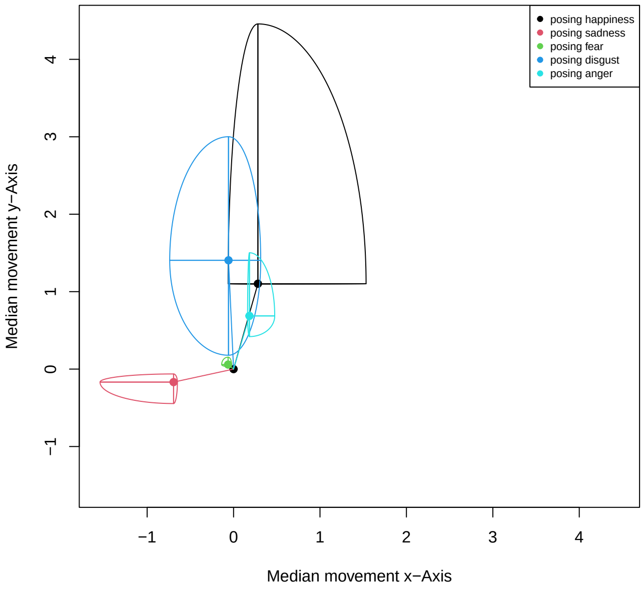

Participant 31 BL7

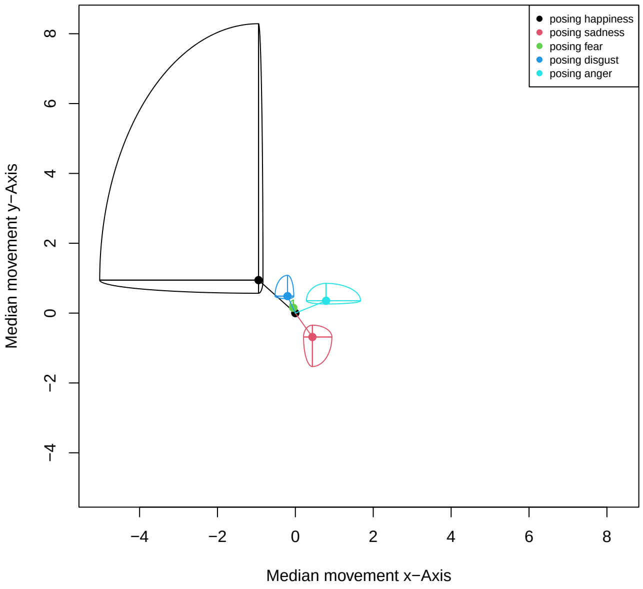

Participant 31 BR7

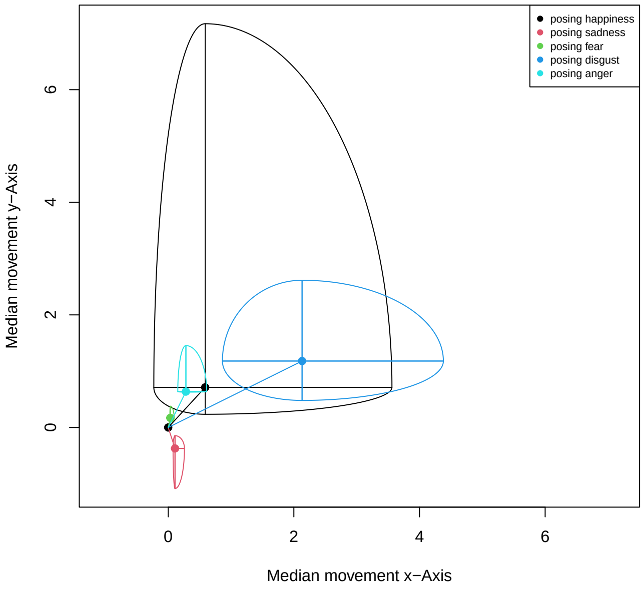

Participant 31 CL7

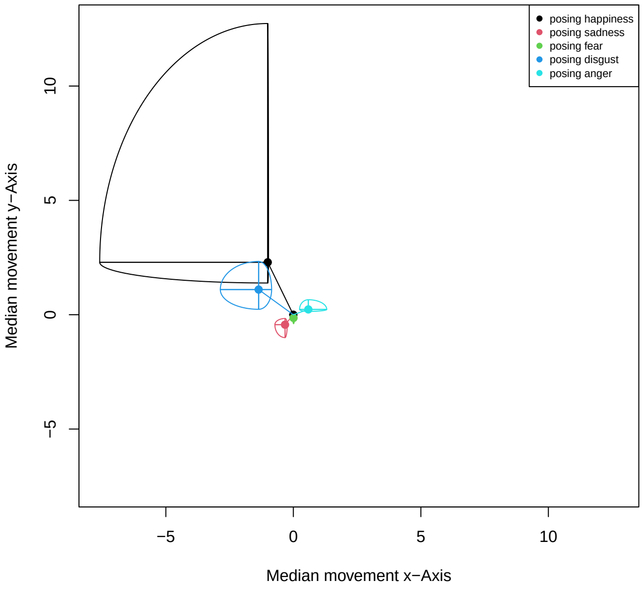

Participant 31 CR7

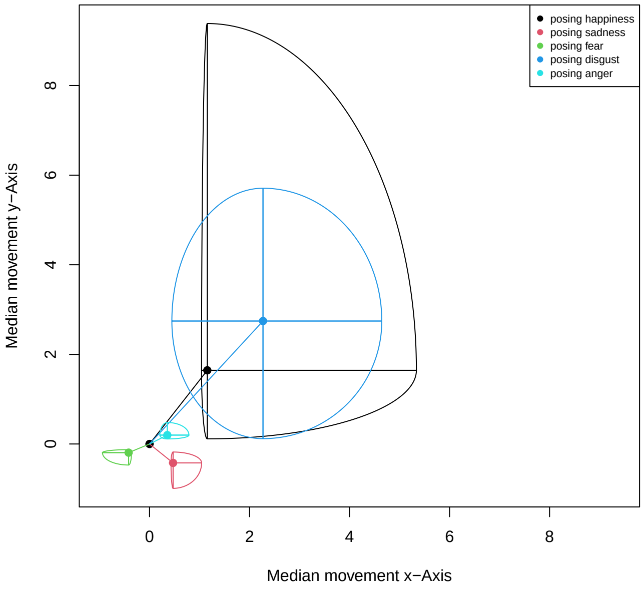

Participant 31 A7

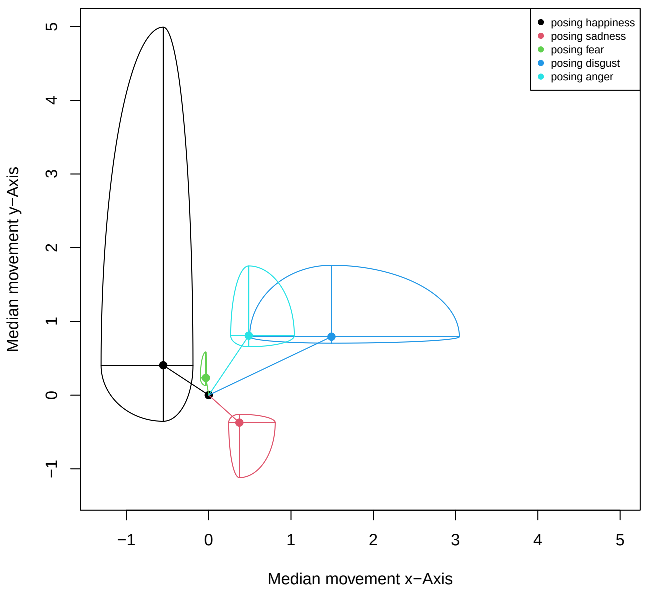

Supplement: Supplementary file 3 [file PSYP-63-e70394-s001.zip › Study 2/Plots/01_Descriptive_Plots/04_MmpCond.pdf]
