# Supplementary material for: blenderFaceUV: Toward Marker‐Based Precision and Flexibility in Facial Tracking With “Invisible” Markers
Source: Psychophysiology. 2026 Sep 21;63(9):e70394. doi: 10.1111/psyp.70394 (PMC13594373; doi:10.1111/psyp.70394)
Supplement: Supplementary file 3 [file PSYP-63-e70394-s001.zip › Study 2/Plots/01_Descriptive_Plots/05_MmpCond_AggregatedSubj.pdf]

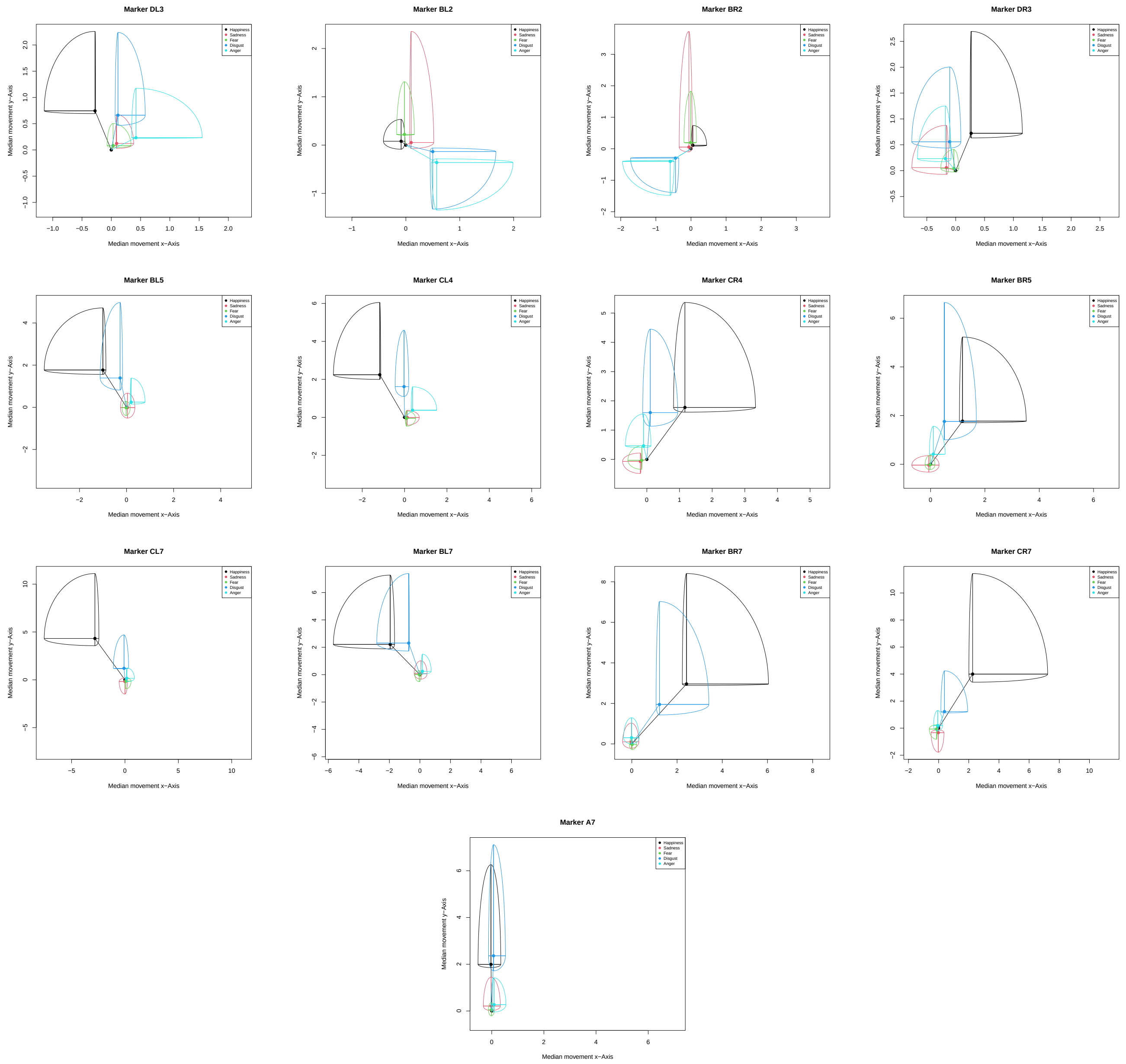

Marker DL3
Marker BL2
Marker BR2
Marker DR3
Marker BL5
Marker CL4
Marker CR4
Marker BR5
Marker CL7
Marker BL7
Marker BR7
Marker CR7
Marker A7
Median movement x−Axis
Median movement y−Axis
Happiness
Sadness
Fear
Disgust
Anger

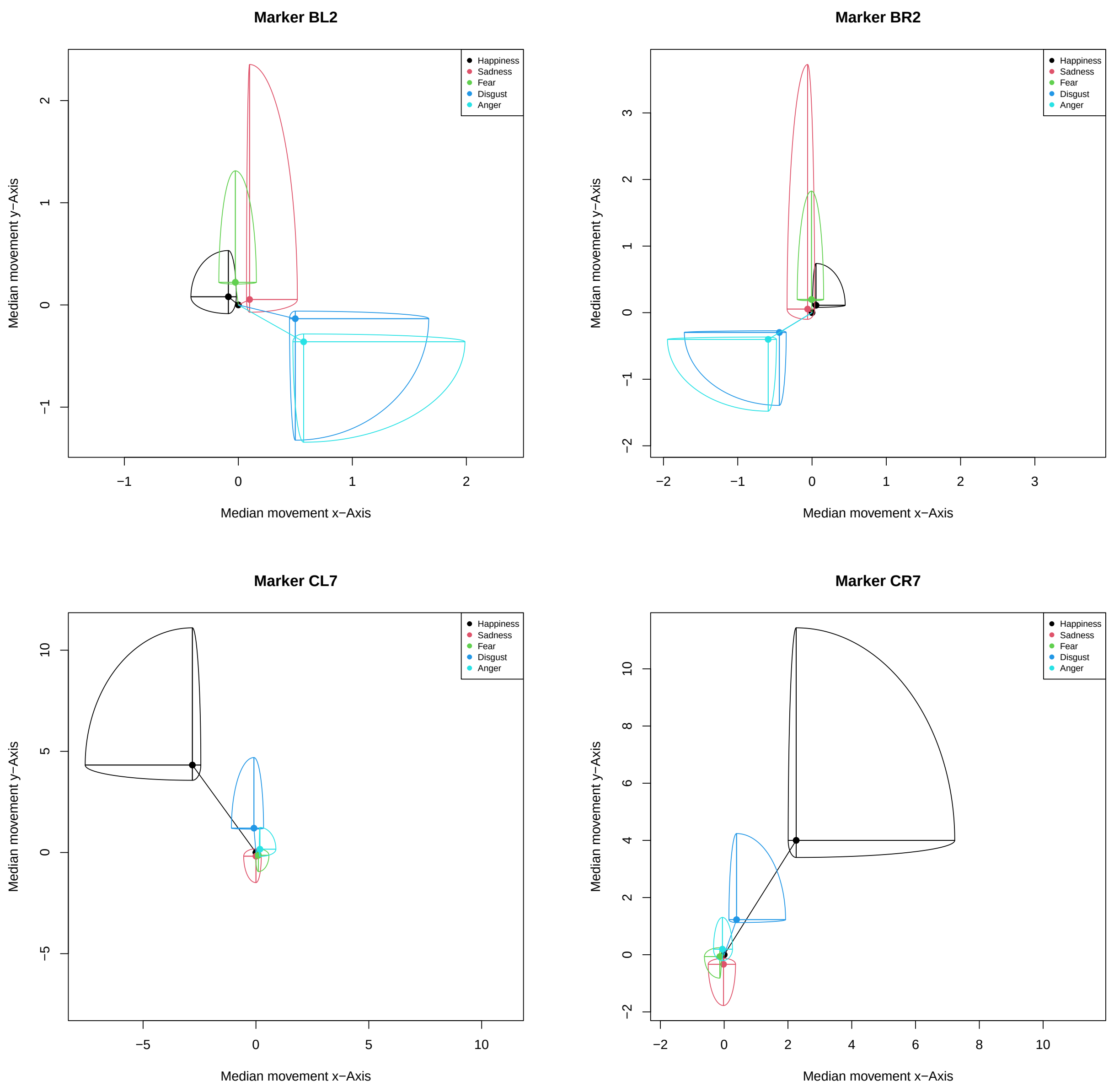
Marker BL2
Marker BR2
Marker CL7
Marker CR7
Happiness
Sadness
Fear
Disgust
Anger
Median movement x−Axis
Median movement y−Axis
