# Supplementary material for: blenderFaceUV: Toward Marker‐Based Precision and Flexibility in Facial Tracking With “Invisible” Markers
Source: Psychophysiology. 2026 Sep 21;63(9):e70394. doi: 10.1111/psyp.70394 (PMC13594373; doi:10.1111/psyp.70394)

Subject 3, Anger

Participant 3 Anger, X_21, BL2_x

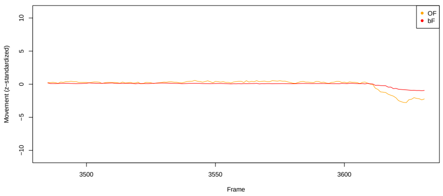

Participant 3 Anger, X_22, BR2_x

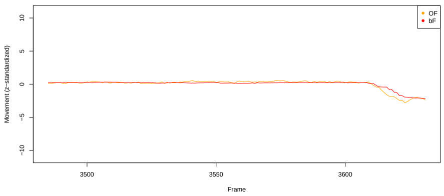

Participant 3 Anger, Y_21, BL2_y

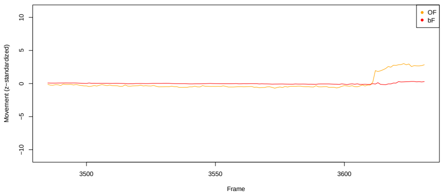

Participant 3 Anger, Y_22, BR2_y

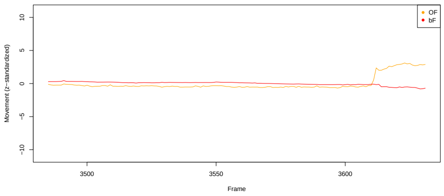

Participant 3 Anger, X_49, BL7_x

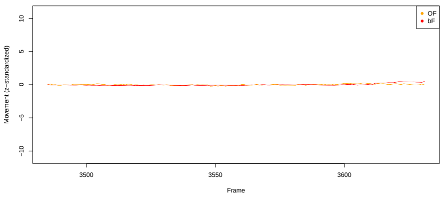

Participant 3 Anger, X_53, BR7_x

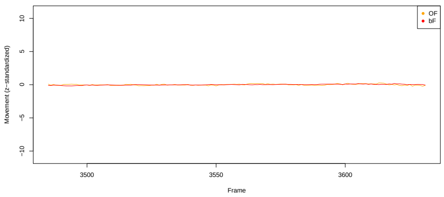

Participant 3 Anger, Y_49, BL7_y

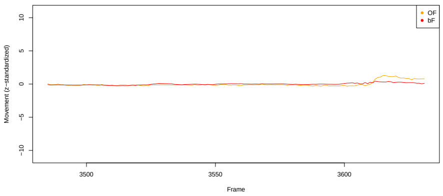

Participant 3 Anger, Y_53, BR7_y

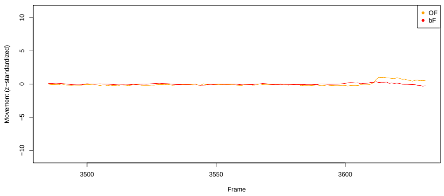

Participant 3 Anger, X_48, CL7_x

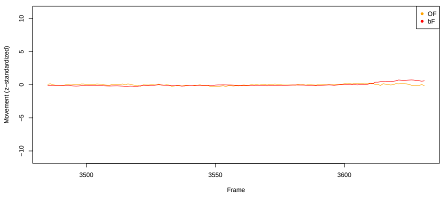

Participant 3 Anger, X_54, CR7_x

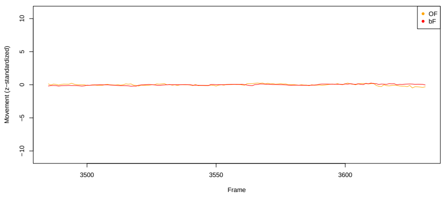

Participant 3 Anger, Y_48, CL7_y

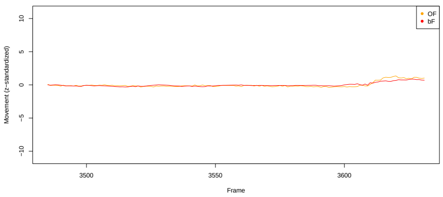

Participant 3 Anger, Y_54, CR7_y

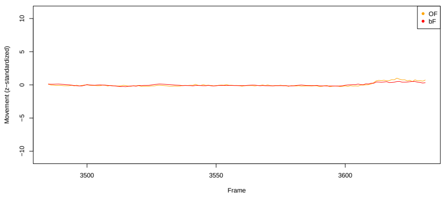

Participant 3 Anger, X_51, A7_x

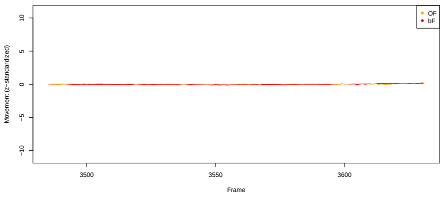

Participant 3 Anger, Y_51, A7_y

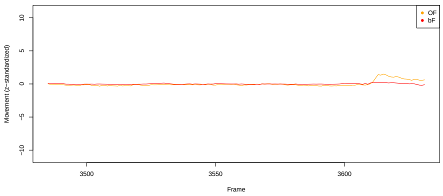

Subject 3, Anxiety

Participant 3 Anxiety, X_21, BL2_x

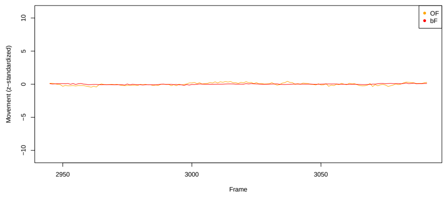

Participant 3 Anxiety, X_22, BR2_x

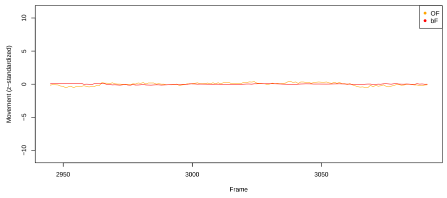

Participant 3 Anxiety, Y_21, BL2_y

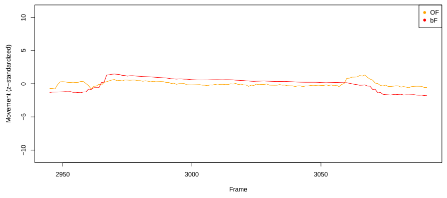

Participant 3 Anxiety, Y_22, BR2_y

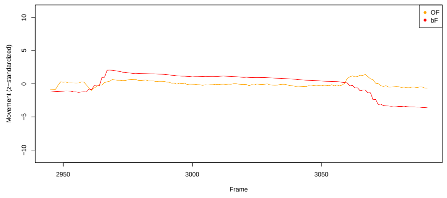

Participant 3 Anxiety, X_49, BL7_x

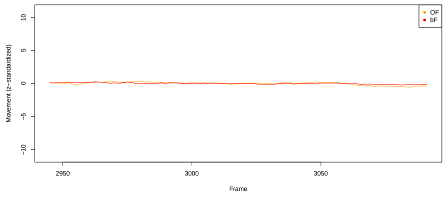

Participant 3 Anxiety, X_53, BR7_x

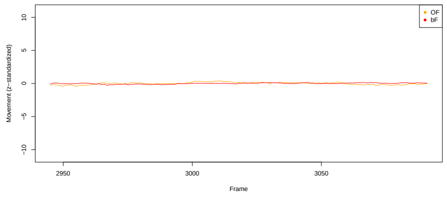

Participant 3 Anxiety, Y_49, BL7_y

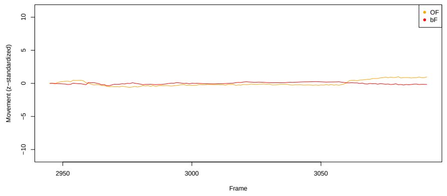

Participant 3 Anxiety, Y_53, BR7_y

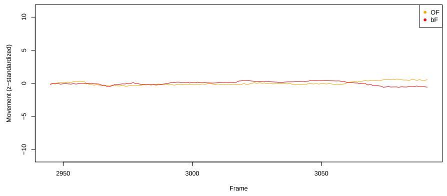

Participant 3 Anxiety, X_48, CL7_x

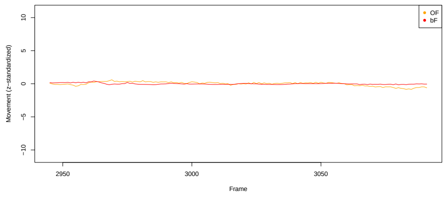

Participant 3 Anxiety, X_54, CR7_x

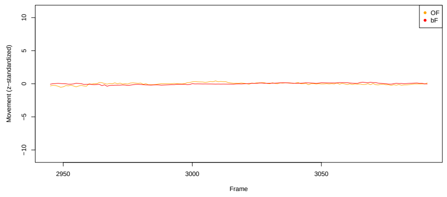

Participant 3 Anxiety, Y_48, CL7_y

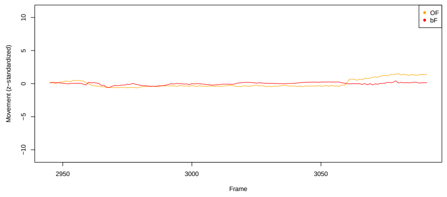

Participant 3 Anxiety, Y_54, CR7_y

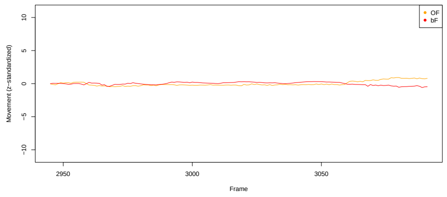

Participant 3 Anxiety, X_51, A7_x

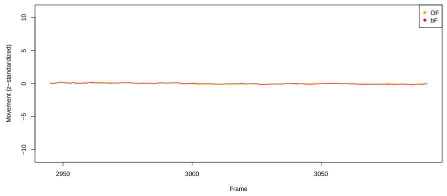

Participant 3 Anxiety, Y_51, A7_y

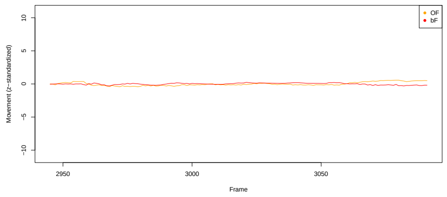

Subject 3, Disgust

Participant 3 Disgust, X_21, BL2_x

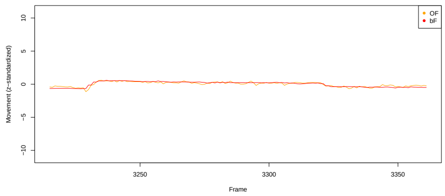

Participant 3 Disgust, X_22, BR2_x

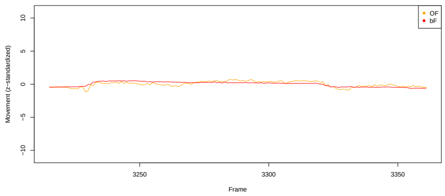

Participant 3 Disgust, Y_21, BL2_y

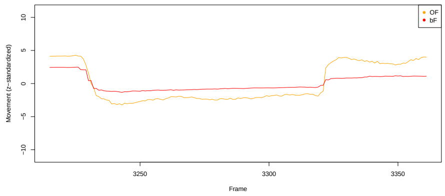

Participant 3 Disgust, Y_22, BR2_y

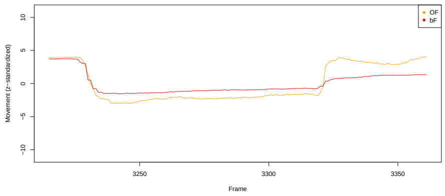

Participant 3 Disgust, X_49, BL7_x

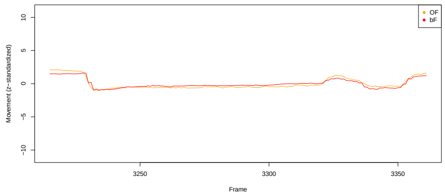

Participant 3 Disgust, X_53, BR7_x

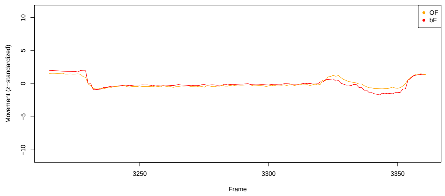

Participant 3 Disgust, Y_49, BL7_y

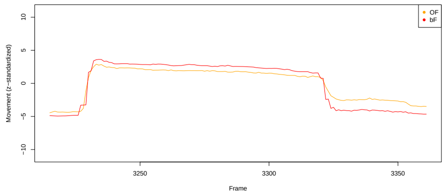

Participant 3 Disgust, Y_53, BR7_y

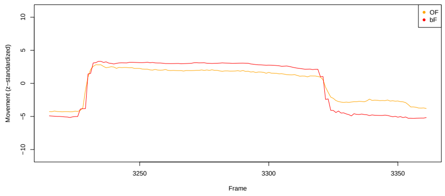

Participant 3 Disgust, X_48, CL7_x

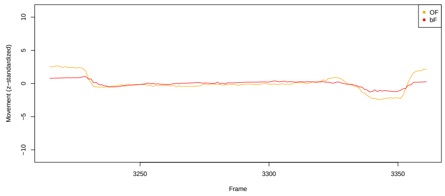

Participant 3 Disgust, X_54, CR7_x

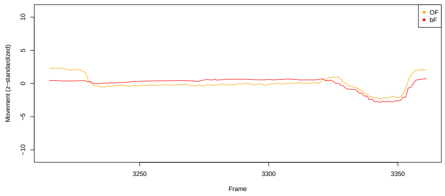

Participant 3 Disgust, Y_48, CL7_y

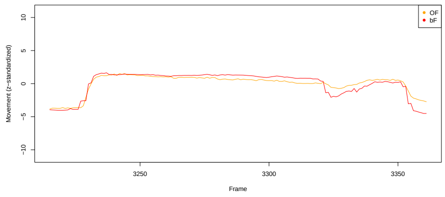

Participant 3 Disgust, Y_54, CR7_y

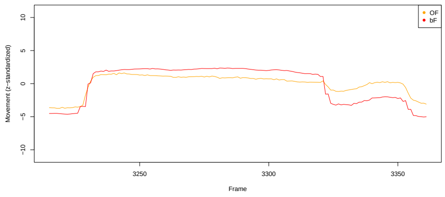

Participant 3 Disgust, X_51, A7_x

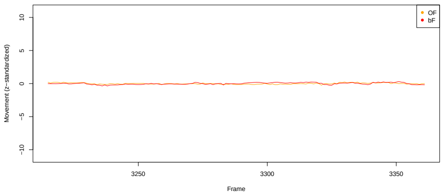

Participant 3 Disgust, Y_51, A7_y

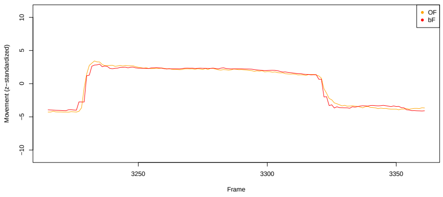

Subject 3, Happiness

Participant 3 Happiness, X_21, BL2_x

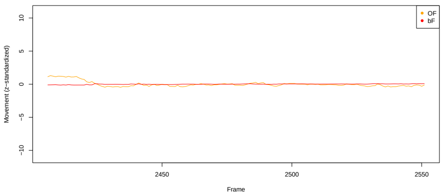

Participant 3 Happiness, X_22, BR2_x

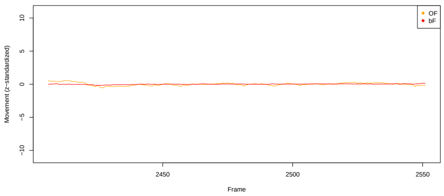

Participant 3 Happiness, Y_21, BL2_y

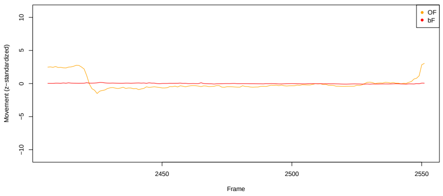

Participant 3 Happiness, Y_22, BR2_y

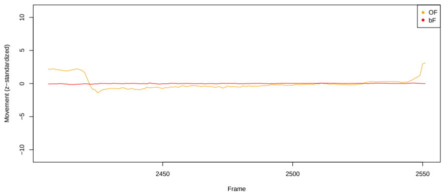

Participant 3 Happiness, X_49, BL7_x

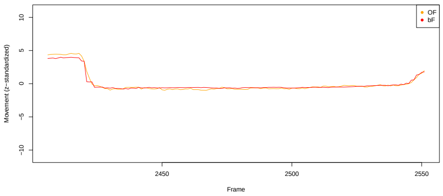

Participant 3 Happiness, X_53, BR7_x

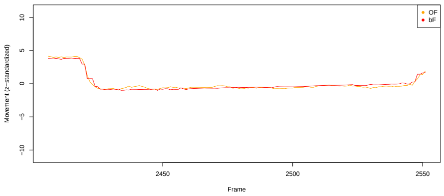

Participant 3 Happiness, Y_49, BL7_y

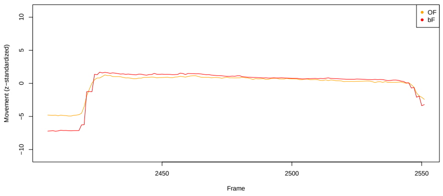

Participant 3 Happiness, Y_53, BR7_y

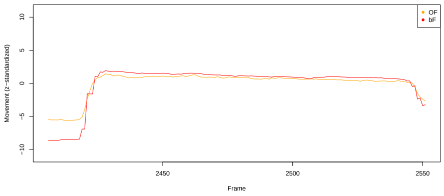

Participant 3 Happiness, X_48, CL7_x

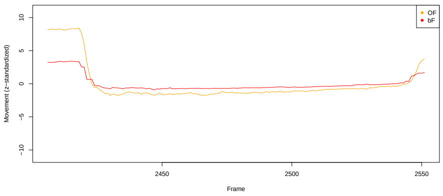

Participant 3 Happiness, X_54, CR7_x

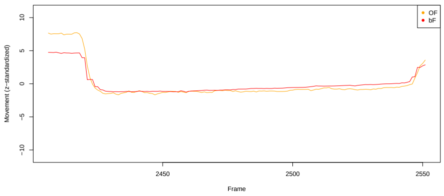

Participant 3 Happiness, Y_48, CL7_y

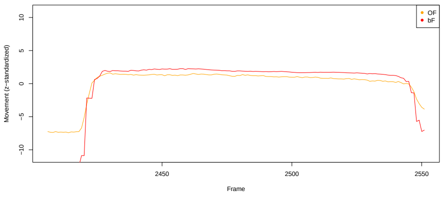

Participant 3 Happiness, Y_54, CR7_y

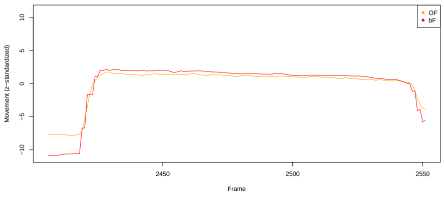

Participant 3 Happiness, X_51, A7_x

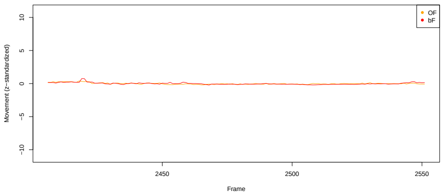

Participant 3 Happiness, Y_51, A7_y

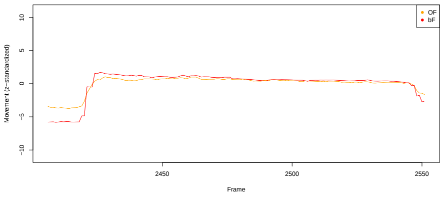

Subject 3, Sadness

Participant 3 Sadness, X_21, BL2_x

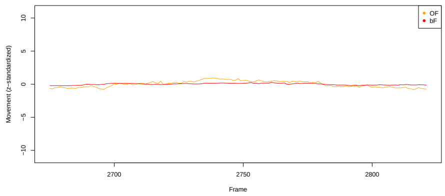

Participant 3 Sadness, X_22, BR2_x

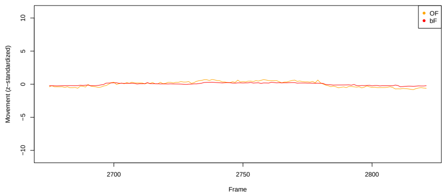

Participant 3 Sadness, Y_21, BL2_y

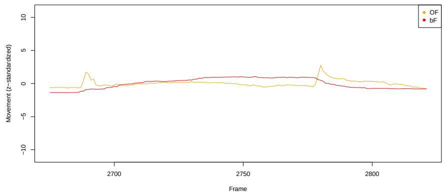

Participant 3 Sadness, Y_22, BR2_y

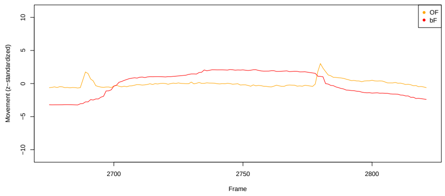

Participant 3 Sadness, X_49, BL7_x

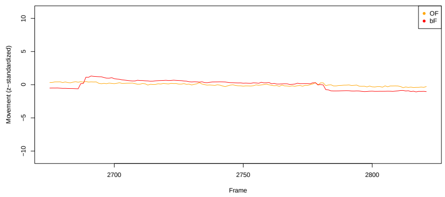

Participant 3 Sadness, X_53, BR7_x

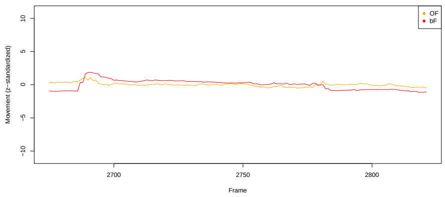

Participant 3 Sadness, Y_49, BL7_y

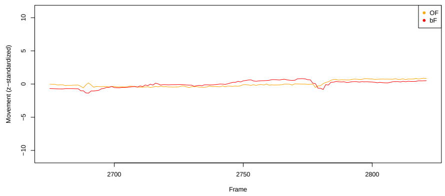

Participant 3 Sadness, Y_53, BR7_y

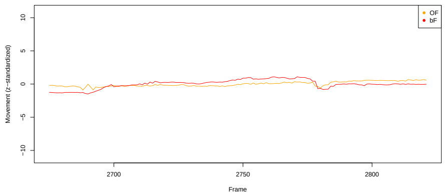

Participant 3 Sadness, X_48, CL7_x

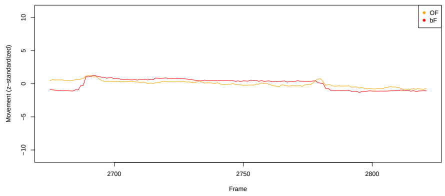

Participant 3 Sadness, X_54, CR7_x

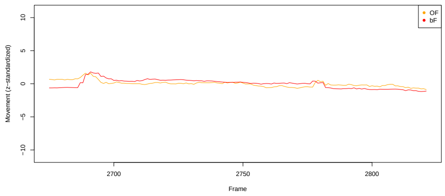

Participant 3 Sadness, Y_48, CL7_y

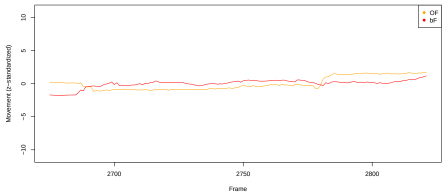

Participant 3 Sadness, Y_54, CR7_y

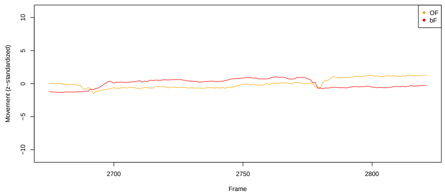

Participant 3 Sadness, X_51, A7_x

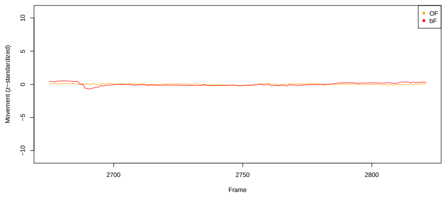

Participant 3 Sadness, Y_51, A7_y

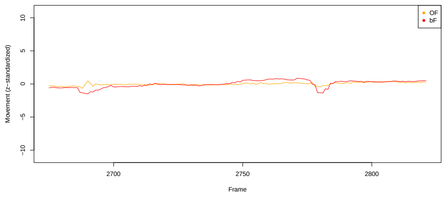

Subject 4, Anger

Participant 4 Anger, X_21, BL2_x

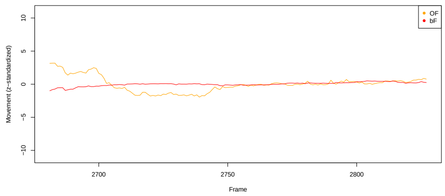

Participant 4 Anger, X_22, BR2_x

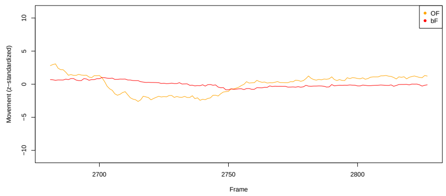

Participant 4 Anger, Y_21, BL2_y

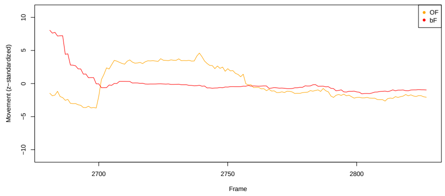

Participant 4 Anger, Y_22, BR2_y

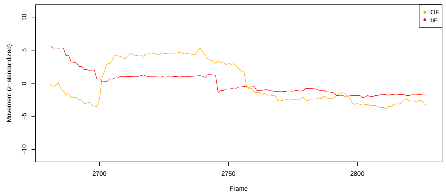

Participant 4 Anger, X_49, BL7_x

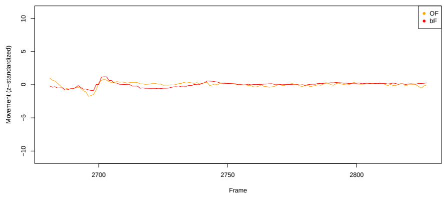

Participant 4 Anger, X_53, BR7_x

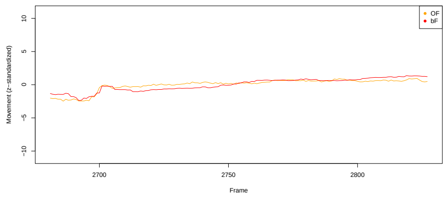

Participant 4 Anger, Y_49, BL7_y

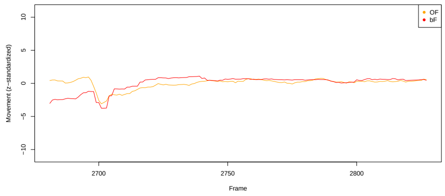

Participant 4 Anger, Y_53, BR7_y

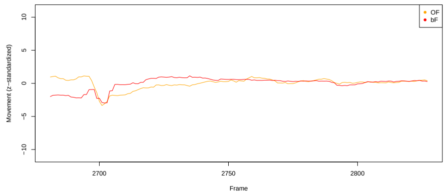

Participant 4 Anger, X_48, CL7_x

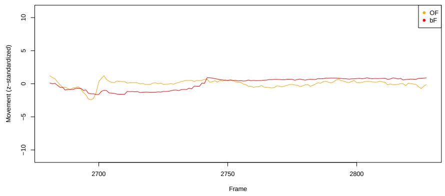

Participant 4 Anger, X_54, CR7_x

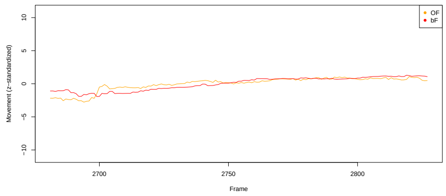

Participant 4 Anger, Y_48, CL7_y

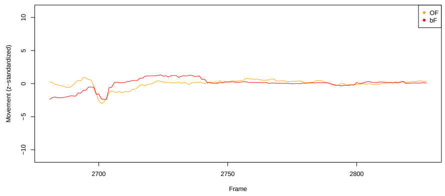

Participant 4 Anger, Y_54, CR7_y

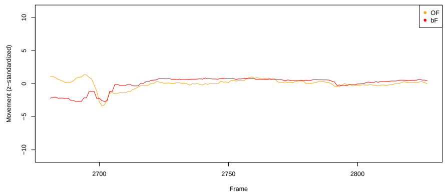

Participant 4 Anger, X_51, A7_x

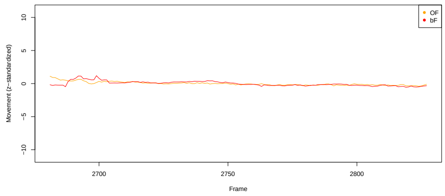

Participant 4 Anger, Y_51, A7_y

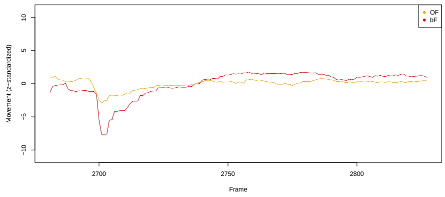

Subject 4, Anxiety

Participant 4 Anxiety, X_21, BL2_x

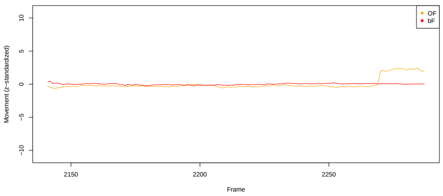

Participant 4 Anxiety, X_22, BR2_x

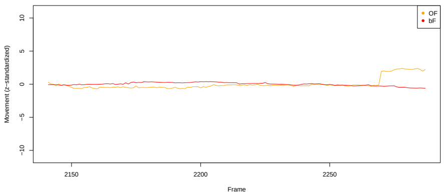

Participant 4 Anxiety, Y_21, BL2_y

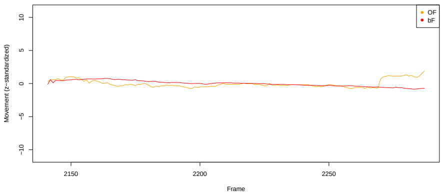

Participant 4 Anxiety, Y_22, BR2_y

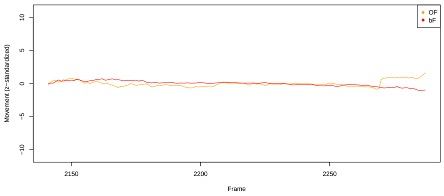

Participant 4 Anxiety, X_49, BL7_x

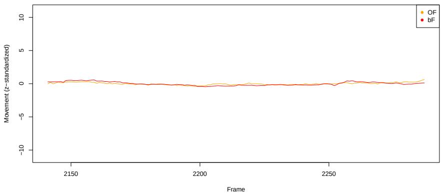

Participant 4 Anxiety, X_53, BR7_x

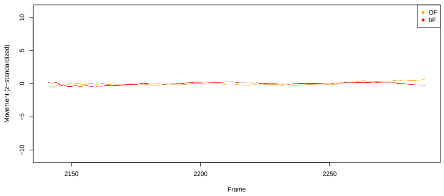

Participant 4 Anxiety, Y_49, BL7_y

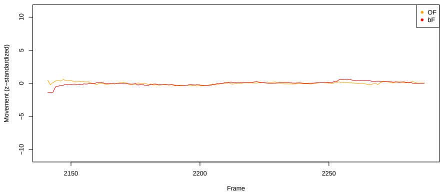

Participant 4 Anxiety, Y_53, BR7_y

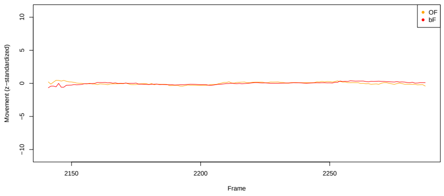

Participant 4 Anxiety, X_48, CL7_x

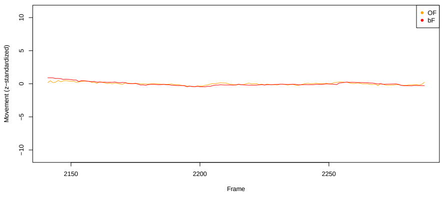

Participant 4 Anxiety, X_54, CR7_x

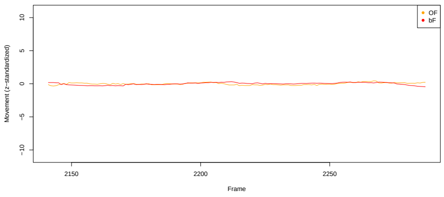

Participant 4 Anxiety, Y_48, CL7_y

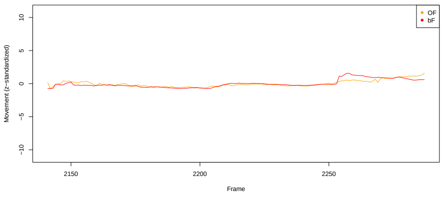

Participant 4 Anxiety, Y_54, CR7_y

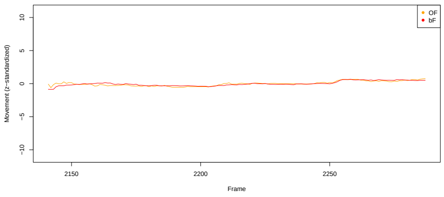

Participant 4 Anxiety, X_51, A7_x

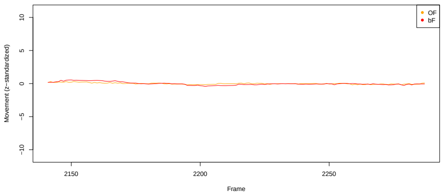

Participant 4 Anxiety, Y_51, A7_y

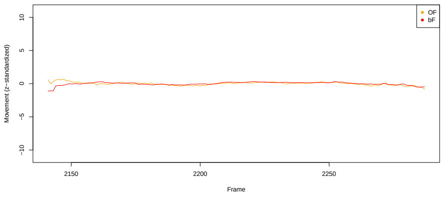

Subject 4, Disgust

Participant 4 Disgust, X_21, BL2_x

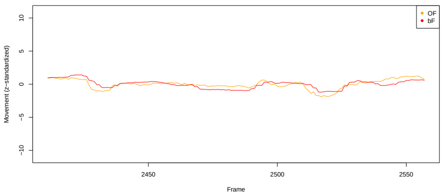

Participant 4 Disgust, X_22, BR2_x

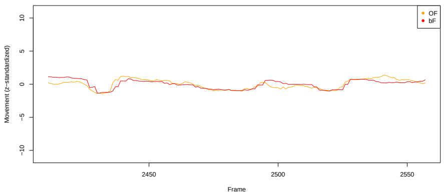

Participant 4 Disgust, Y_21, BL2_y

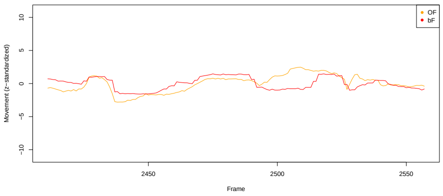

Participant 4 Disgust, Y_22, BR2_y

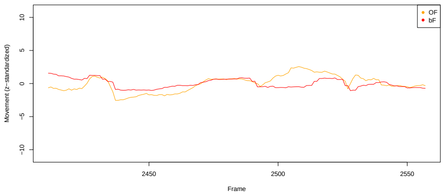

Participant 4 Disgust, X_49, BL7_x

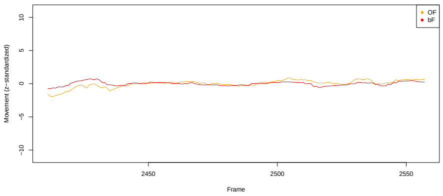

Participant 4 Disgust, X_53, BR7_x

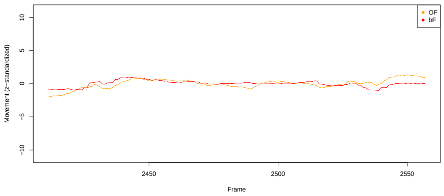

Participant 4 Disgust, Y_49, BL7_y

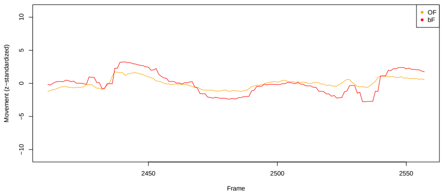

Participant 4 Disgust, Y_53, BR7_y

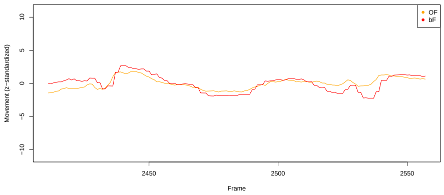

Participant 4 Disgust, X_48, CL7_x

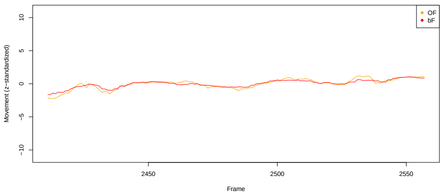

Participant 4 Disgust, X_54, CR7_x

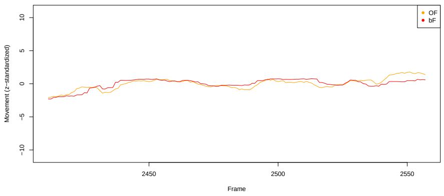

Participant 4 Disgust, Y_48, CL7_y

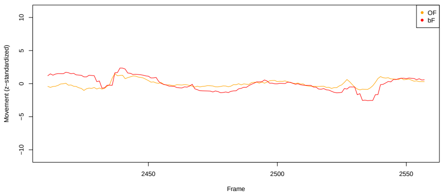

Participant 4 Disgust, Y_54, CR7_y

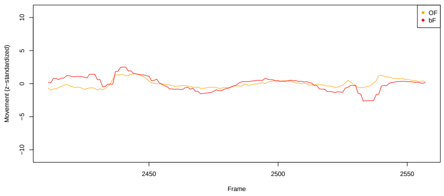

Participant 4 Disgust, X_51, A7_x

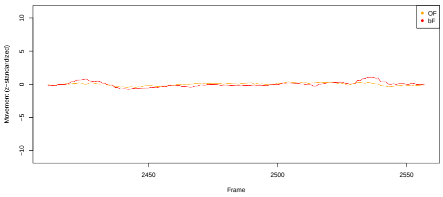

Participant 4 Disgust, Y_51, A7_y

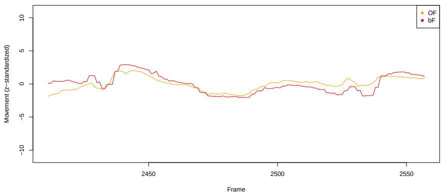

Subject 4, Happiness

Participant 4 Happiness, X_21, BL2_x

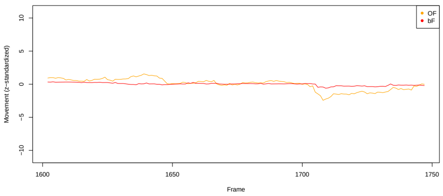

Participant 4 Happiness, X_22, BR2_x

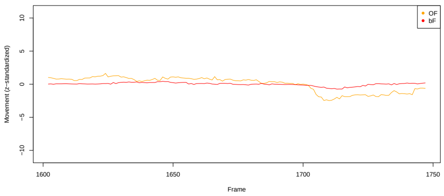

Participant 4 Happiness, Y_21, BL2_y

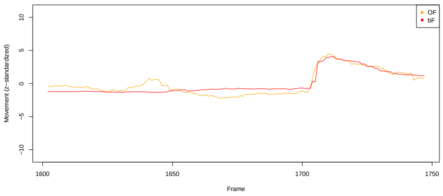

Participant 4 Happiness, Y_22, BR2_y

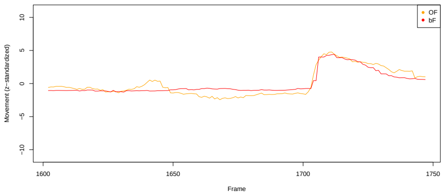

Participant 4 Happiness, X_49, BL7_x

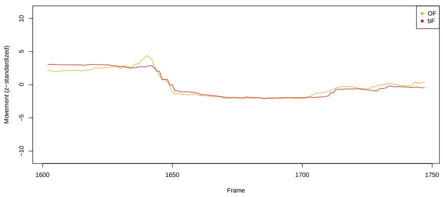

Participant 4 Happiness, X_53, BR7_x

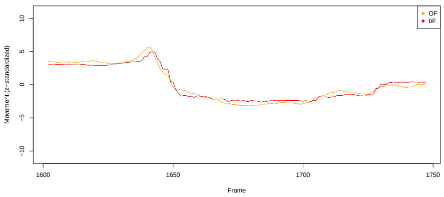

Participant 4 Happiness, Y_49, BL7_y

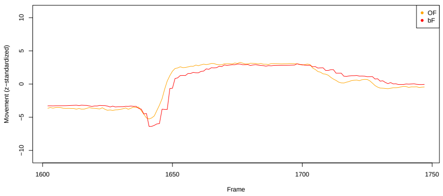

Participant 4 Happiness, Y_53, BR7_y

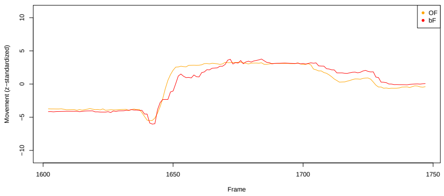

Participant 4 Happiness, X_48, CL7_x

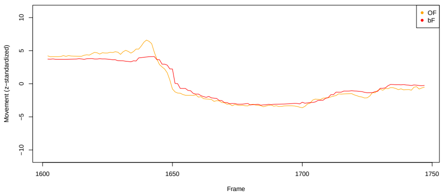

Participant 4 Happiness, X_54, CR7_x

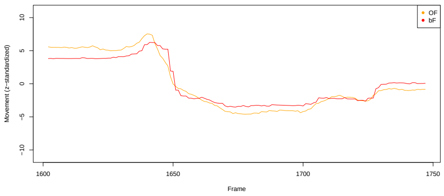

Participant 4 Happiness, Y_48, CL7_y

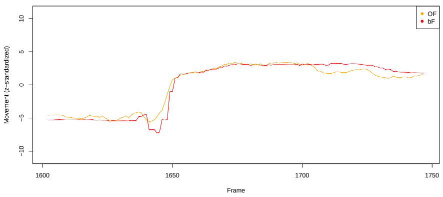

Participant 4 Happiness, Y_54, CR7_y

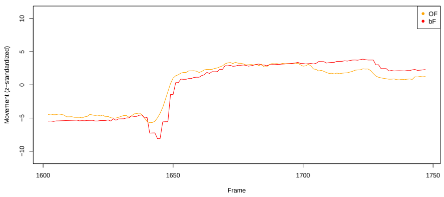

Participant 4 Happiness, X_51, A7_x

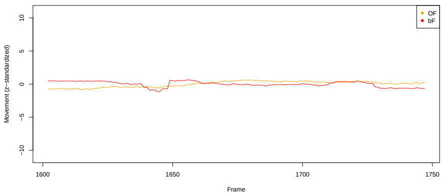

Participant 4 Happiness, Y_51, A7_y

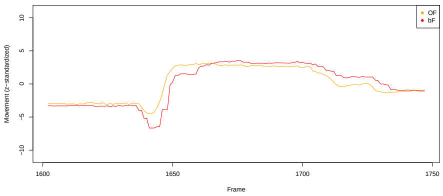

Subject 4, Sadness

Participant 4 Sadness, X_21, BL2_x

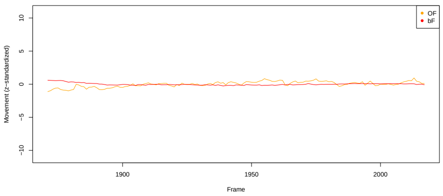

Participant 4 Sadness, X_22, BR2_x

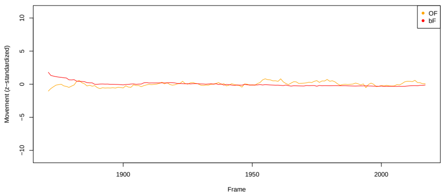

Participant 4 Sadness, Y_21, BL2_y

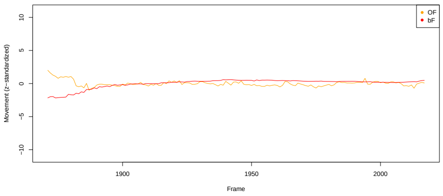

Participant 4 Sadness, Y_22, BR2_y

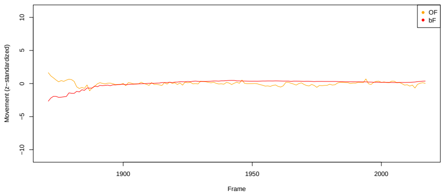

Participant 4 Sadness, X_49, BL7_x

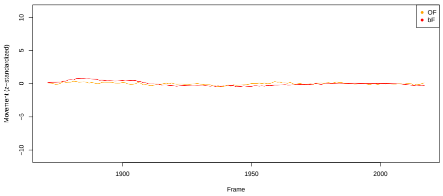

Participant 4 Sadness, X_53, BR7_x

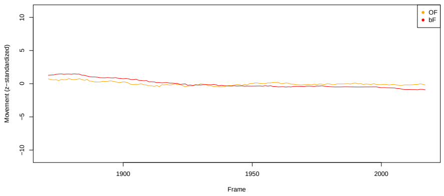

Participant 4 Sadness, Y_49, BL7_y

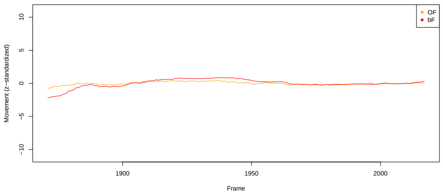

Participant 4 Sadness, Y_53, BR7_y

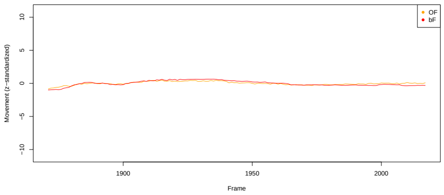

Participant 4 Sadness, X_48, CL7_x

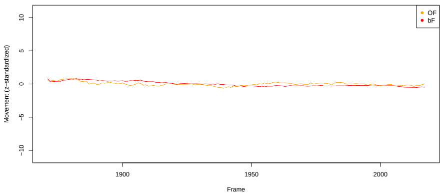

Participant 4 Sadness, X_54, CR7_x

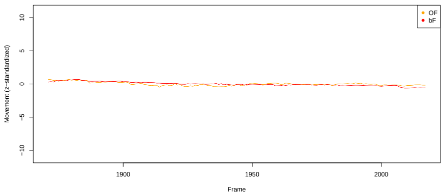

Participant 4 Sadness, Y_48, CL7_y

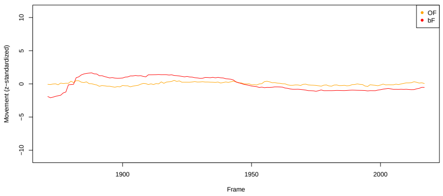

Participant 4 Sadness, Y_54, CR7_y

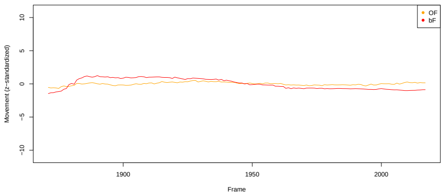

Participant 4 Sadness, X_51, A7_x

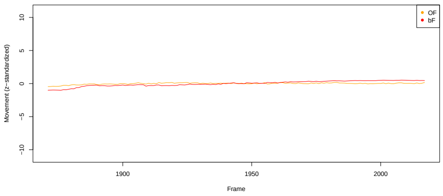

Participant 4 Sadness, Y_51, A7_y

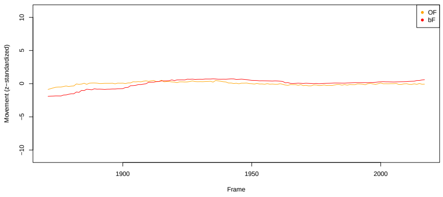

Subject 6, Anger

Participant 6 Anger, X_21, BL2_x

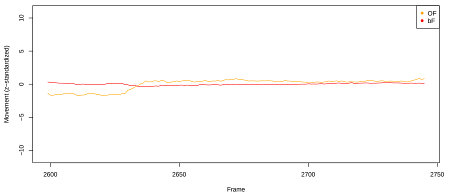

Participant 6 Anger, X_22, BR2_x

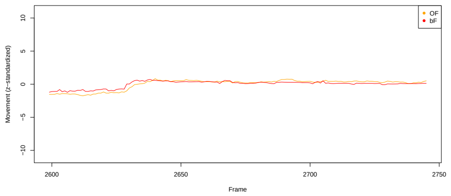

Participant 6 Anger, Y_21, BL2_y

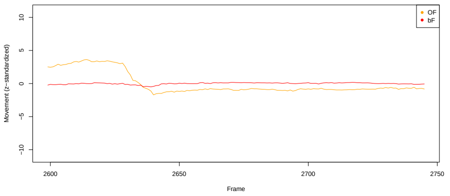

Participant 6 Anger, Y_22, BR2_y

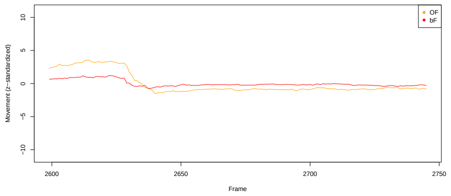

Participant 6 Anger, X_49, BL7_x

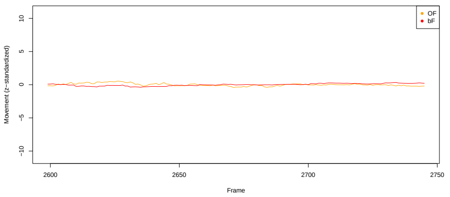

Participant 6 Anger, X_53, BR7_x

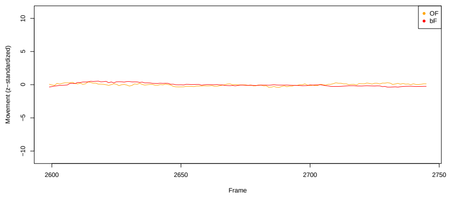

Participant 6 Anger, Y_49, BL7_y

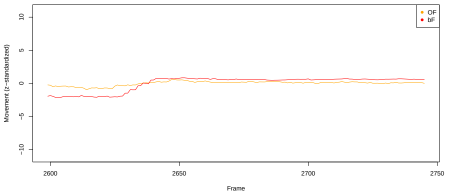

Participant 6 Anger, Y_53, BR7_y

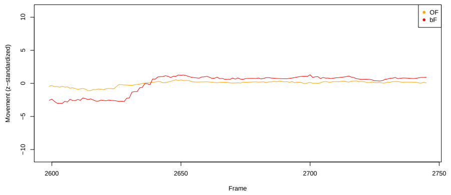

Participant 6 Anger, X_48, CL7_x

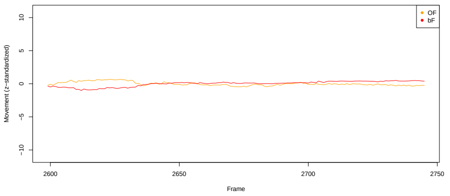

Participant 6 Anger, X_54, CR7_x

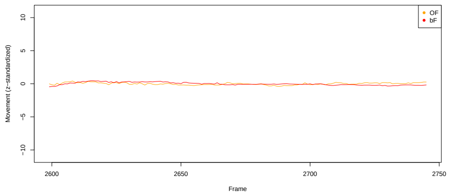

Participant 6 Anger, Y_48, CL7_y

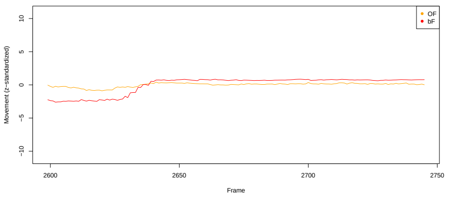

Participant 6 Anger, Y_54, CR7_y

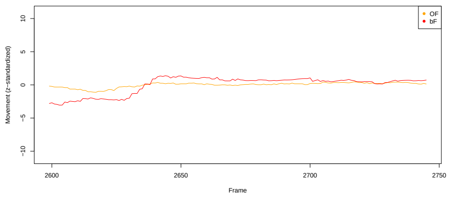

Participant 6 Anger, X_51, A7_x

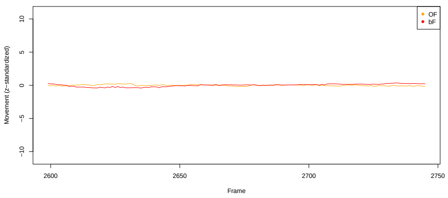

Participant 6 Anger, Y_51, A7_y

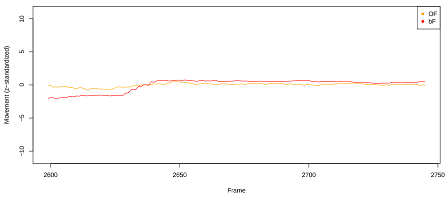

Subject 6, Anxiety

Participant 6 Anxiety, X_21, BL2_x

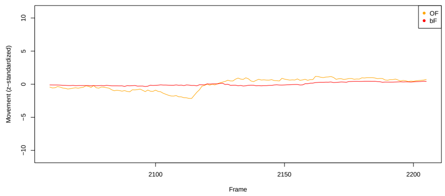

Participant 6 Anxiety, X_22, BR2_x

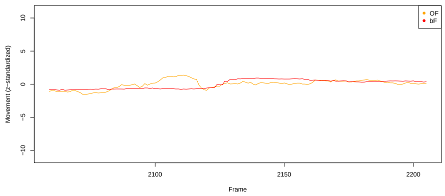

Participant 6 Anxiety, Y_21, BL2_y

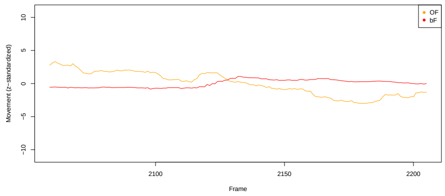

Participant 6 Anxiety, Y_22, BR2_y

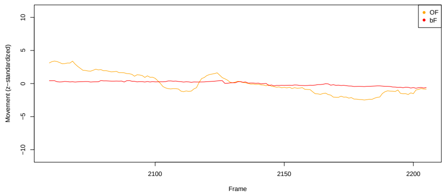

Participant 6 Anxiety, X_49, BL7_x

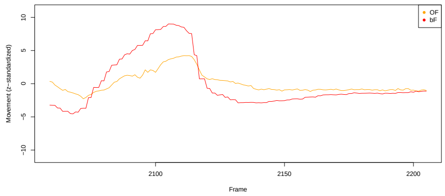

Participant 6 Anxiety, X_53, BR7_x

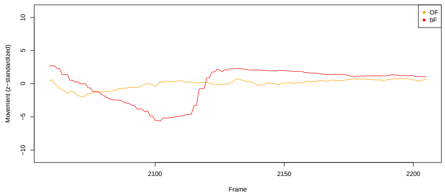

Participant 6 Anxiety, Y_49, BL7_y

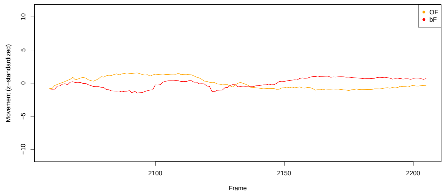

Participant 6 Anxiety, Y_53, BR7_y

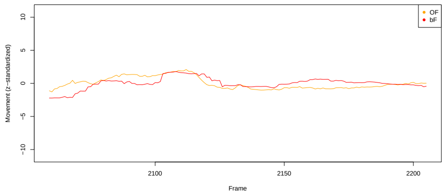

Participant 6 Anxiety, X_48, CL7_x

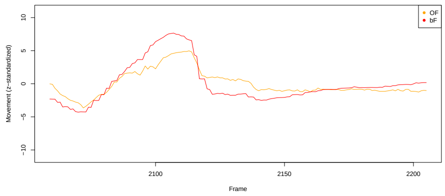

Participant 6 Anxiety, X_54, CR7_x

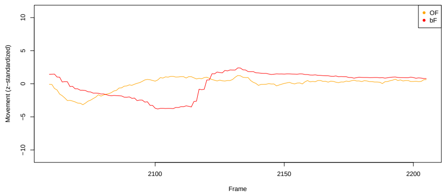

Participant 6 Anxiety, Y_48, CL7_y

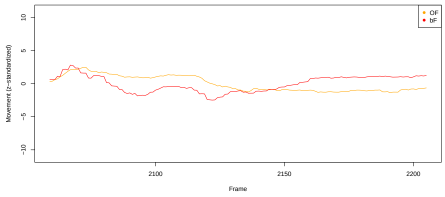

Participant 6 Anxiety, Y_54, CR7_y

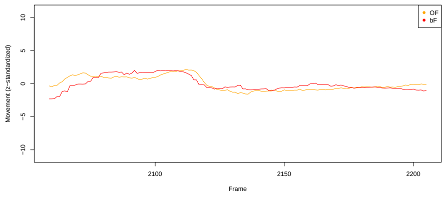

Participant 6 Anxiety, X_51, A7_x

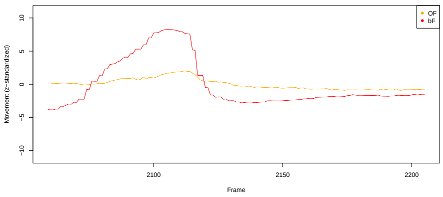

Participant 6 Anxiety, Y_51, A7_y

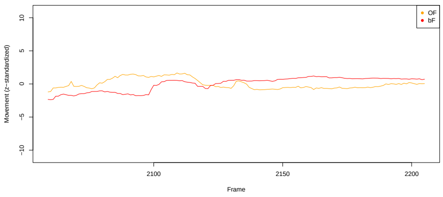

Subject 6, Disgust

Participant 6 Disgust, X_21, BL2_x

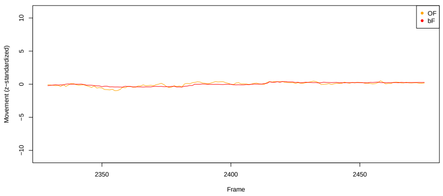

Participant 6 Disgust, X_22, BR2_x

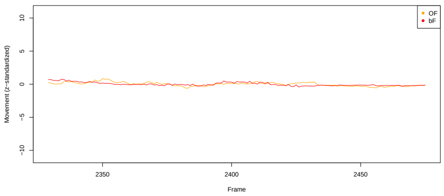

Participant 6 Disgust, Y_21, BL2_y

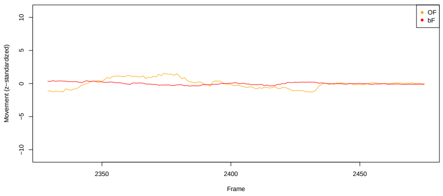

Participant 6 Disgust, Y_22, BR2_y

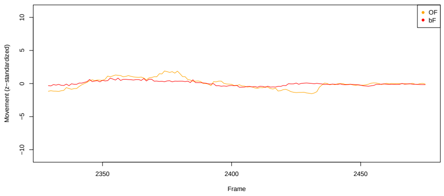

Participant 6 Disgust, X_49, BL7_x

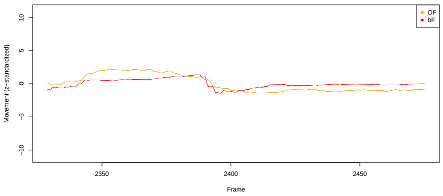

Participant 6 Disgust, X_53, BR7_x

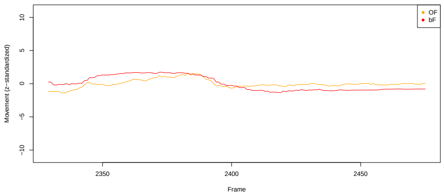

Participant 6 Disgust, Y_49, BL7_y

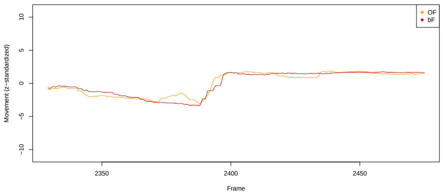

Participant 6 Disgust, Y_53, BR7_y

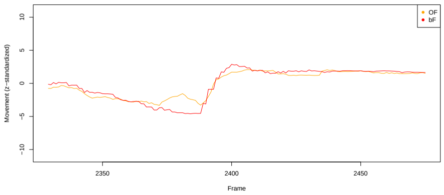

Participant 6 Disgust, X_48, CL7_x

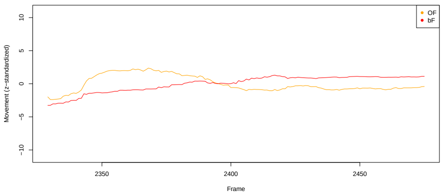

Participant 6 Disgust, X_54, CR7_x

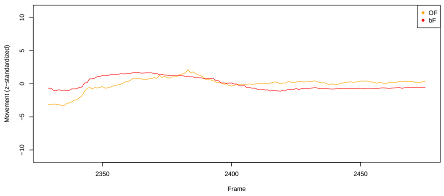

Participant 6 Disgust, Y_48, CL7_y

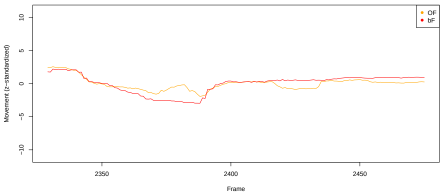

Participant 6 Disgust, Y_54, CR7_y

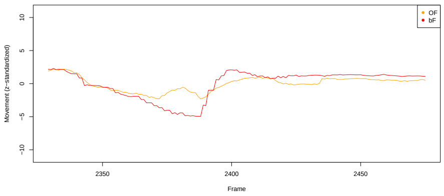

Participant 6 Disgust, X_51, A7_x

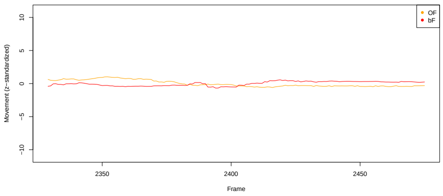

Participant 6 Disgust, Y_51, A7_y

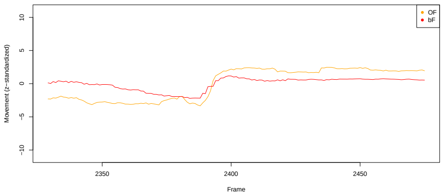

Subject 6, Happiness

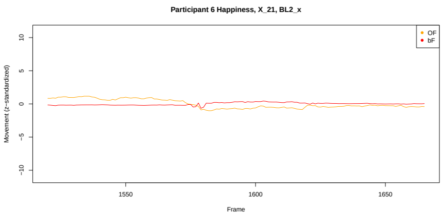

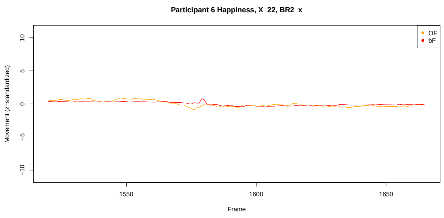

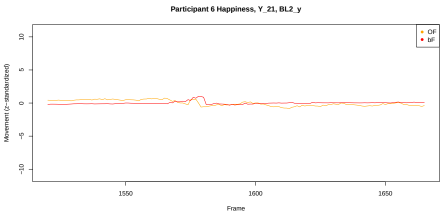

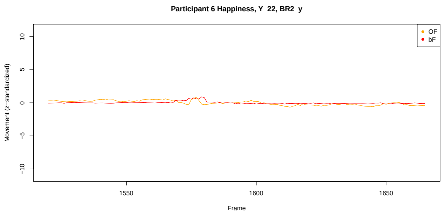

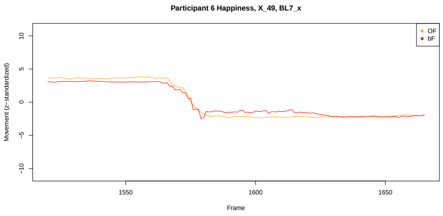

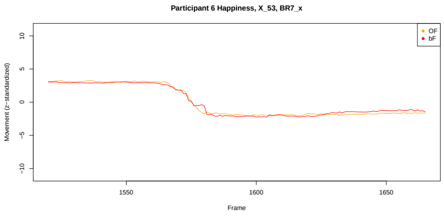

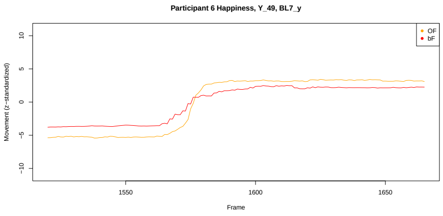

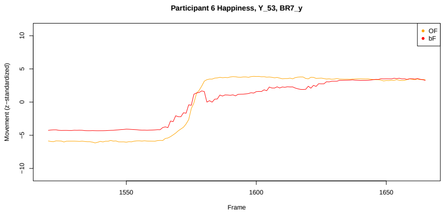

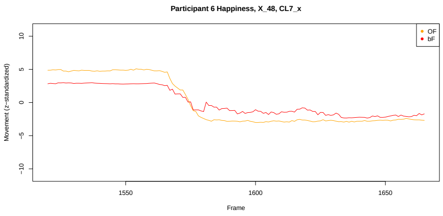

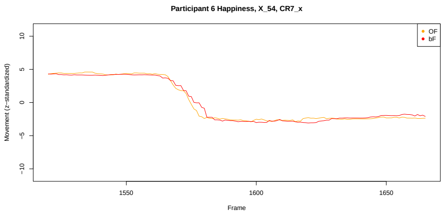

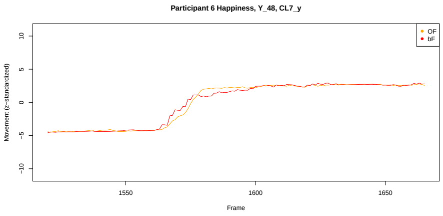

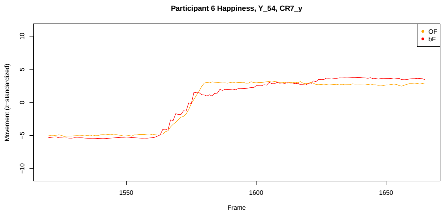

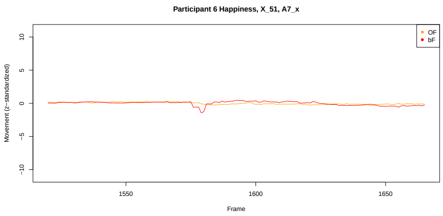

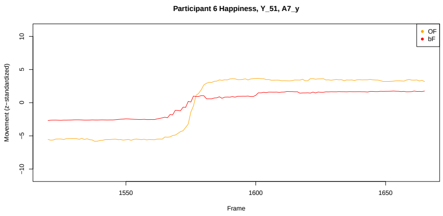

Subject 6, Sadness

Participant 6 Sadness, X_21, BL2_x

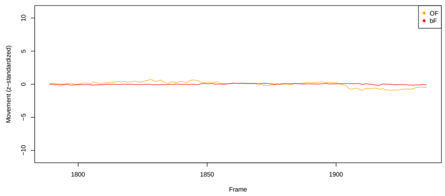

Participant 6 Sadness, X_22, BR2_x

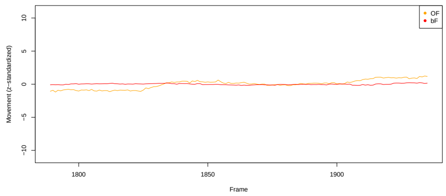

Participant 6 Sadness, Y_21, BL2_y

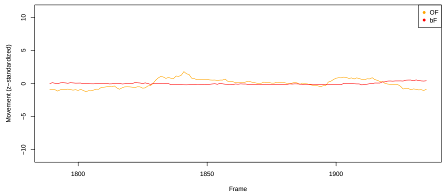

Participant 6 Sadness, Y_22, BR2_y

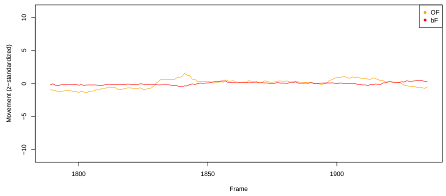

Participant 6 Sadness, X_49, BL7_x

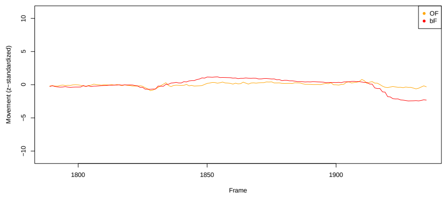

Participant 6 Sadness, X_53, BR7_x

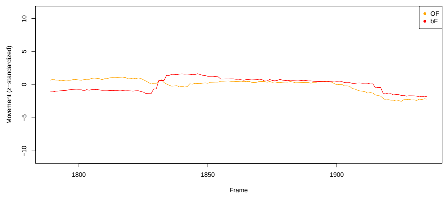

Participant 6 Sadness, Y_49, BL7_y

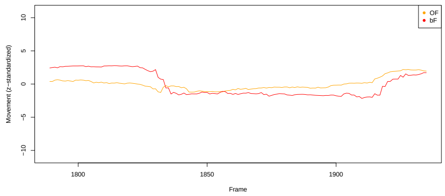

Participant 6 Sadness, Y_53, BR7_y

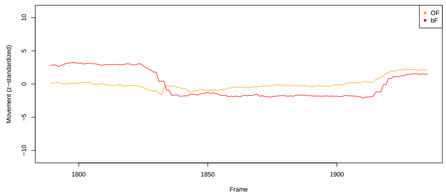

Participant 6 Sadness, X_48, CL7_x

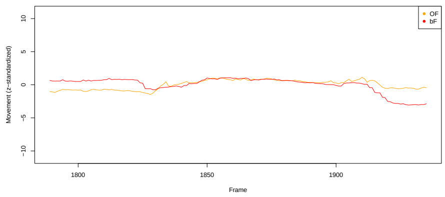

Participant 6 Sadness, X_54, CR7_x

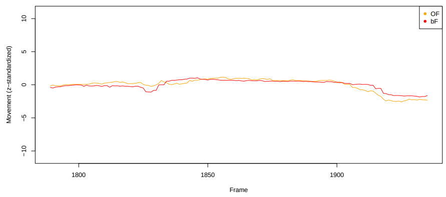

Participant 6 Sadness, Y_48, CL7_y

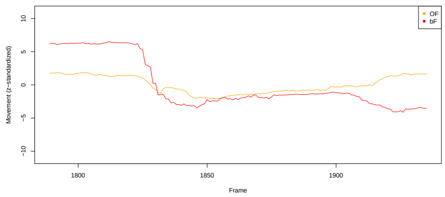

Participant 6 Sadness, Y_54, CR7_y

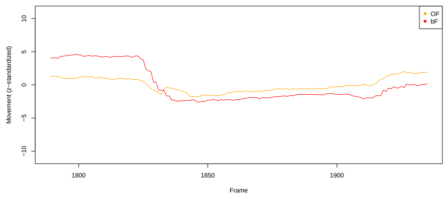

Participant 6 Sadness, X_51, A7_x

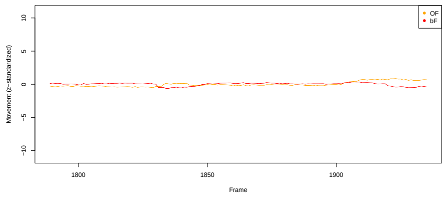

Participant 6 Sadness, Y_51, A7_y

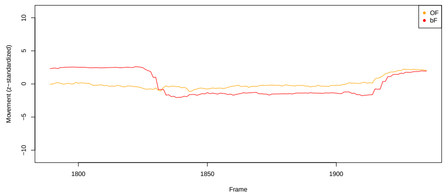

Subject 7, Anger

Participant 7 Anger, X_21, BL2_x

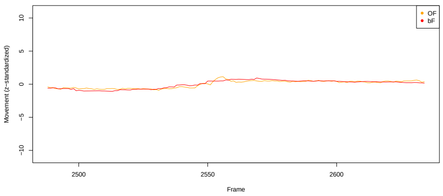

Participant 7 Anger, X_22, BR2_x

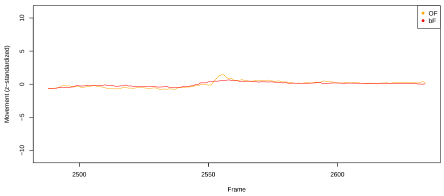

Participant 7 Anger, Y_21, BL2_y

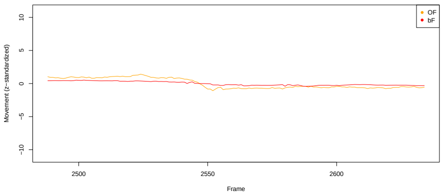

Participant 7 Anger, Y_22, BR2_y

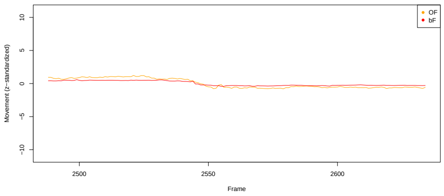

Participant 7 Anger, X_49, BL7_x

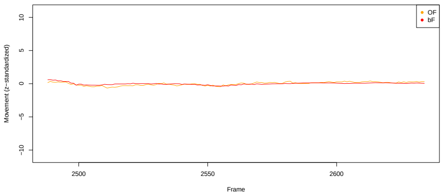

Participant 7 Anger, X_53, BR7_x

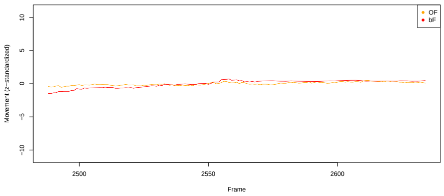

Participant 7 Anger, Y_49, BL7_y

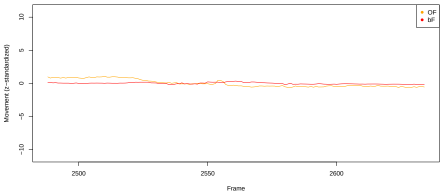

Participant 7 Anger, Y_53, BR7_y

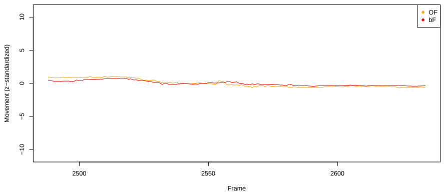

Participant 7 Anger, X_48, CL7_x

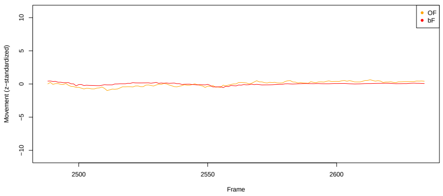

Participant 7 Anger, X_54, CR7_x

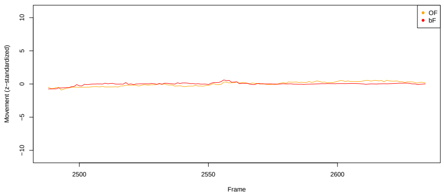

Participant 7 Anger, Y_48, CL7_y

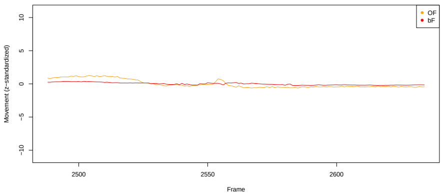

Participant 7 Anger, Y_54, CR7_y

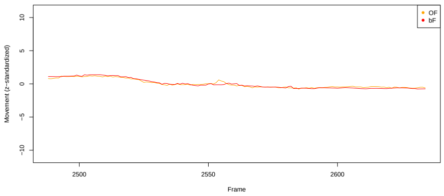

Participant 7 Anger, X_51, A7_x

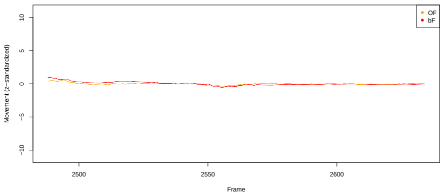

Participant 7 Anger, Y_51, A7_y

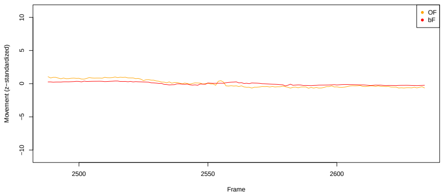

Subject 7, Anxiety

Participant 7 Anxiety, X_21, BL2_x

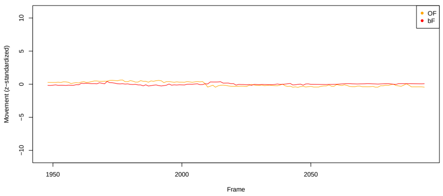

Participant 7 Anxiety, X_22, BR2_x

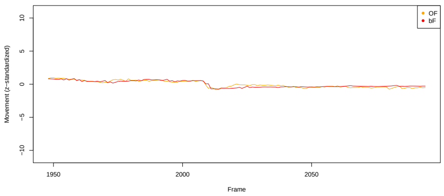

Participant 7 Anxiety, Y_21, BL2_y

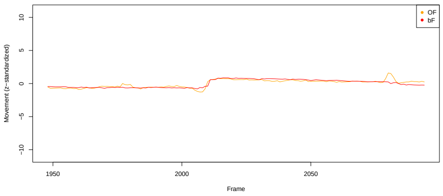

Participant 7 Anxiety, Y_22, BR2_y

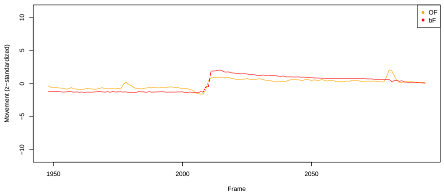

Participant 7 Anxiety, X_49, BL7_x

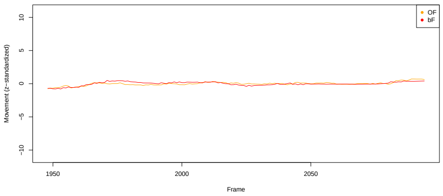

Participant 7 Anxiety, X_53, BR7_x

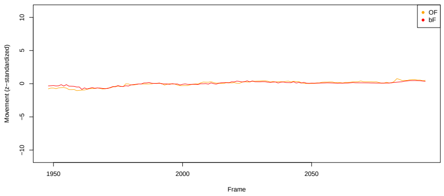

Participant 7 Anxiety, Y_49, BL7_y

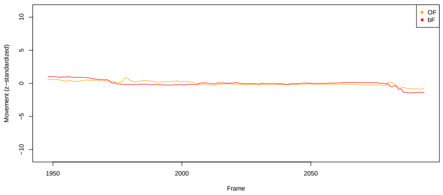

Participant 7 Anxiety, Y_53, BR7_y

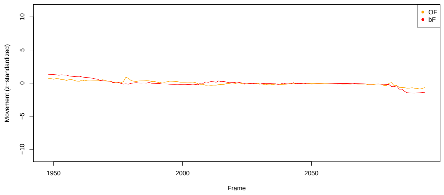

Participant 7 Anxiety, X_48, CL7_x

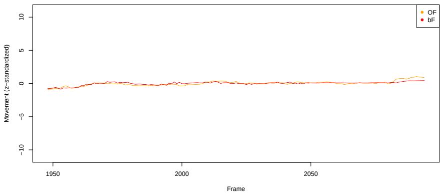

Participant 7 Anxiety, X_54, CR7_x

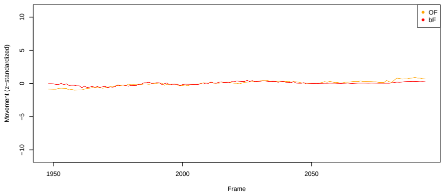

Participant 7 Anxiety, Y_48, CL7_y

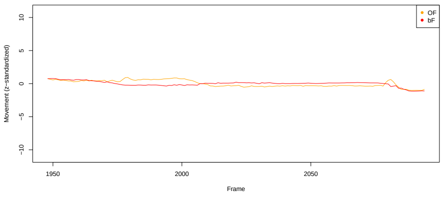

Participant 7 Anxiety, Y_54, CR7_y

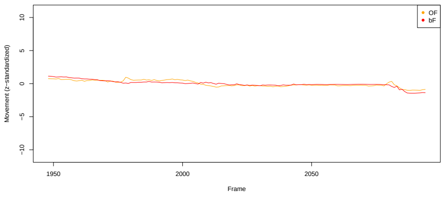

Participant 7 Anxiety, X_51, A7_x

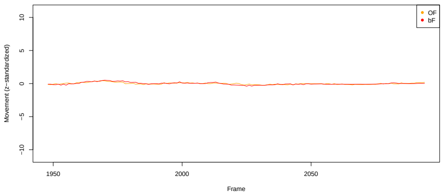

Participant 7 Anxiety, Y_51, A7_y

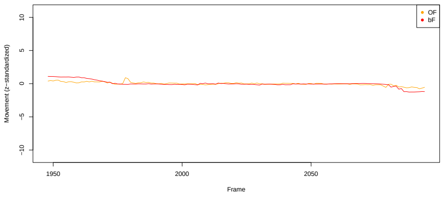

Subject 7, Disgust

Participant 7 Disgust, X_21, BL2_x

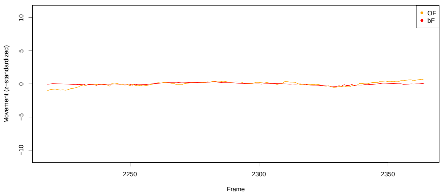

Participant 7 Disgust, X_22, BR2_x

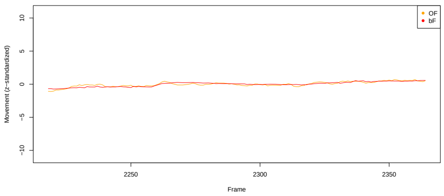

Participant 7 Disgust, Y_21, BL2_y

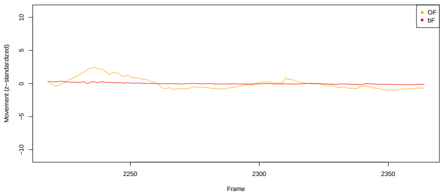

Participant 7 Disgust, Y_22, BR2_y

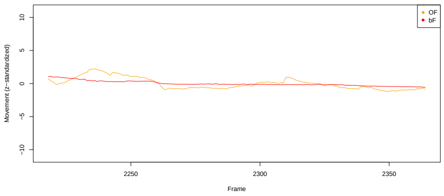

Participant 7 Disgust, X_49, BL7_x

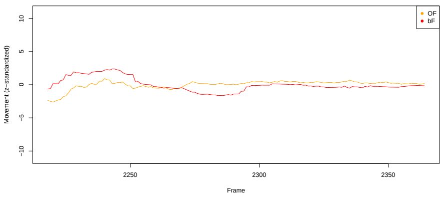

Participant 7 Disgust, X_53, BR7_x

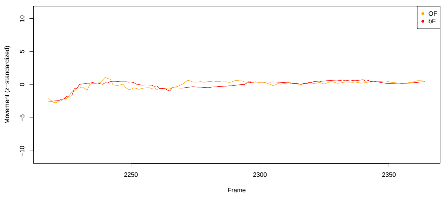

Participant 7 Disgust, Y_49, BL7_y

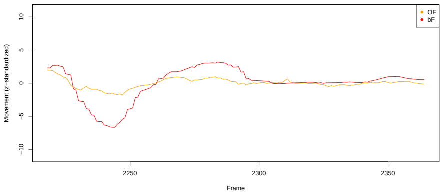

Participant 7 Disgust, Y_53, BR7_y

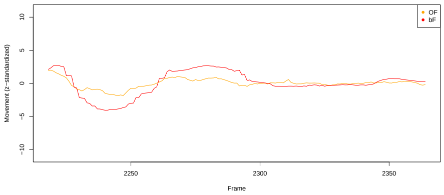

Participant 7 Disgust, X_48, CL7_x

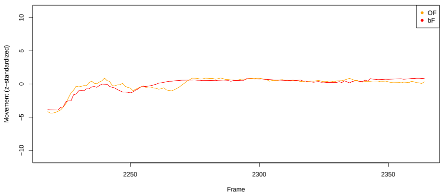

Participant 7 Disgust, X_54, CR7_x

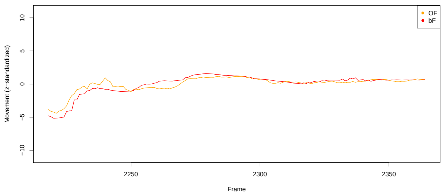

Participant 7 Disgust, Y_48, CL7_y

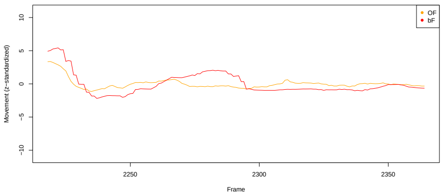

Participant 7 Disgust, Y_54, CR7_y

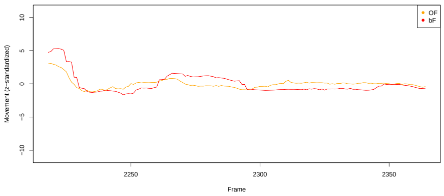

Participant 7 Disgust, X_51, A7_x

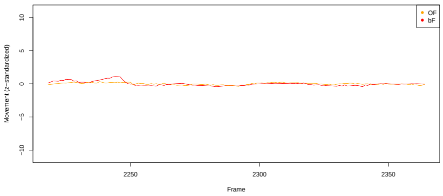

Participant 7 Disgust, Y_51, A7_y

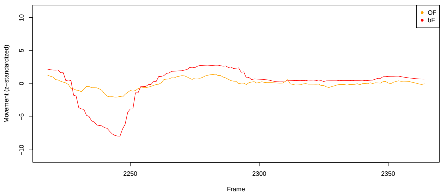

Subject 7, Happiness

Participant 7 Happiness, X_21, BL2_x

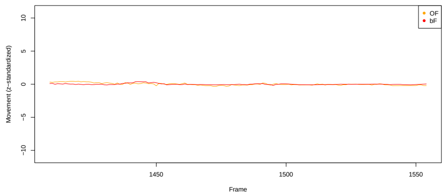

Participant 7 Happiness, X_22, BR2_x

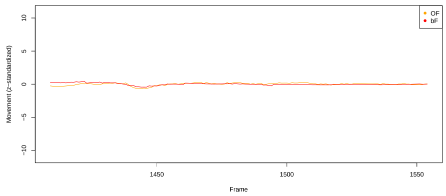

Participant 7 Happiness, Y_21, BL2_y

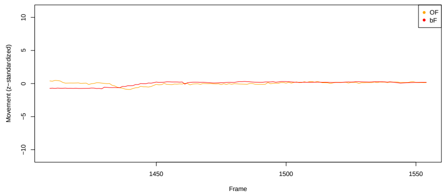

Participant 7 Happiness, Y_22, BR2_y

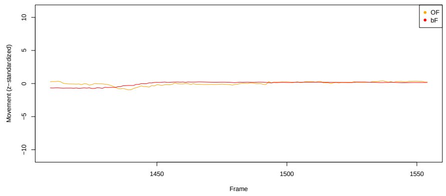

Participant 7 Happiness, X_49, BL7_x

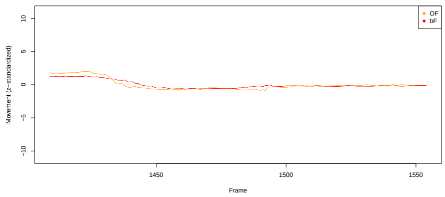

Participant 7 Happiness, X_53, BR7_x

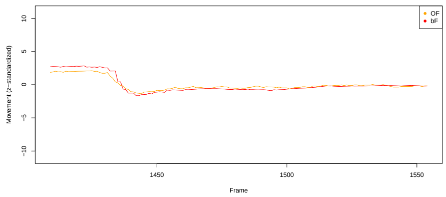

Participant 7 Happiness, Y_49, BL7_y

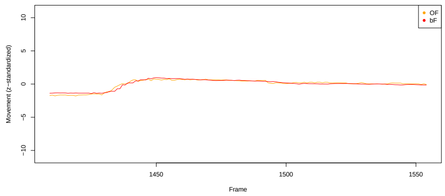

Participant 7 Happiness, Y_53, BR7_y

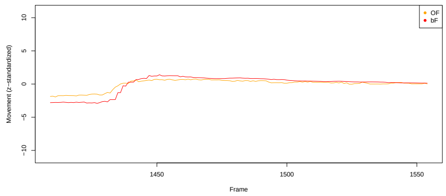

Participant 7 Happiness, X_48, CL7_x

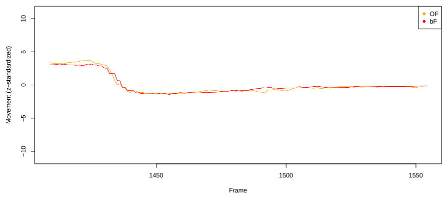

Participant 7 Happiness, X_54, CR7_x

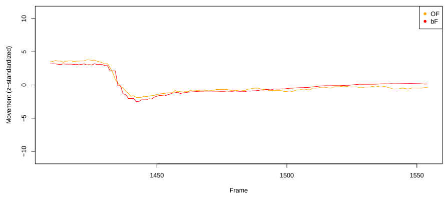

Participant 7 Happiness, Y_48, CL7_y

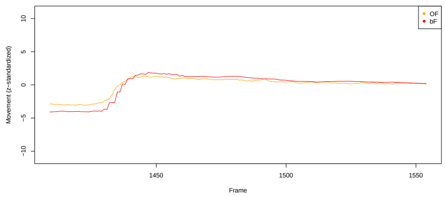

Participant 7 Happiness, Y_54, CR7_y

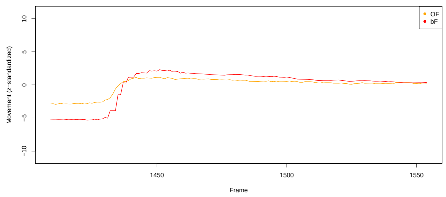

Participant 7 Happiness, X_51, A7_x

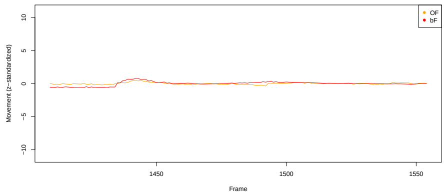

Participant 7 Happiness, Y_51, A7_y

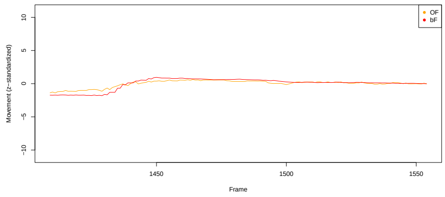

Subject 7, Sadness

Participant 7 Sadness, X_21, BL2_x

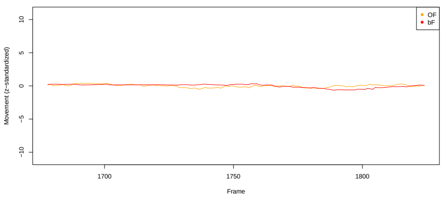

Participant 7 Sadness, X_22, BR2_x

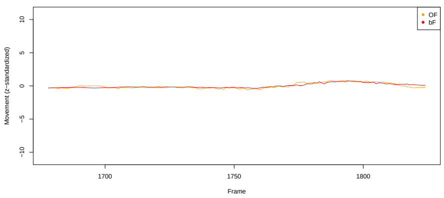

Participant 7 Sadness, Y_21, BL2_y

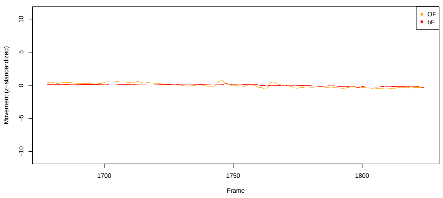

Participant 7 Sadness, Y_22, BR2_y

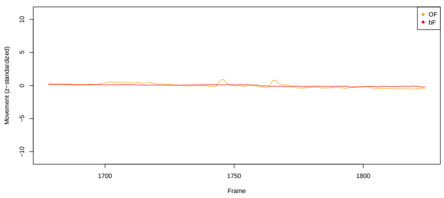

Participant 7 Sadness, X_49, BL7_x

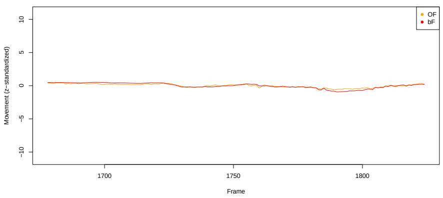

Participant 7 Sadness, X_53, BR7_x

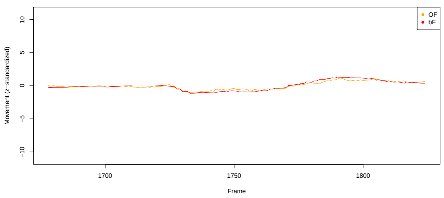

Participant 7 Sadness, Y_49, BL7_y

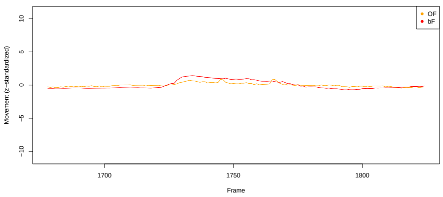

Participant 7 Sadness, Y_53, BR7_y

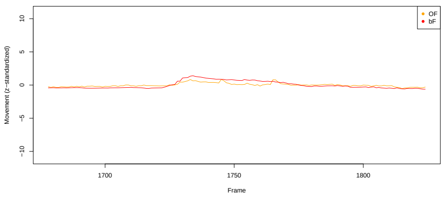

Participant 7 Sadness, X_48, CL7_x

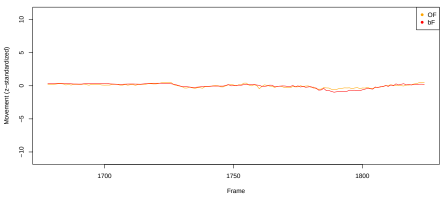

Participant 7 Sadness, X_54, CR7_x

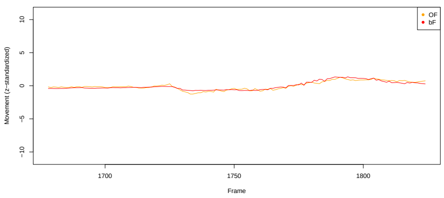

Participant 7 Sadness, Y_48, CL7_y

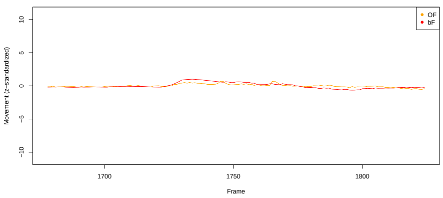

Participant 7 Sadness, Y_54, CR7_y

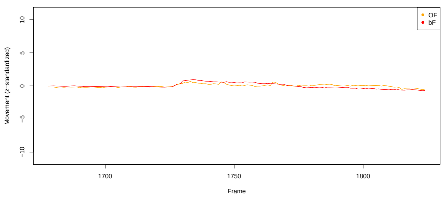

Participant 7 Sadness, X_51, A7_x

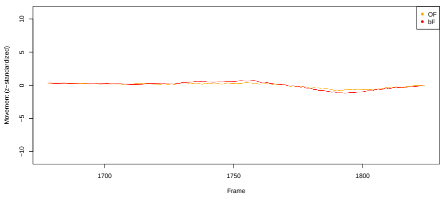

Participant 7 Sadness, Y_51, A7_y

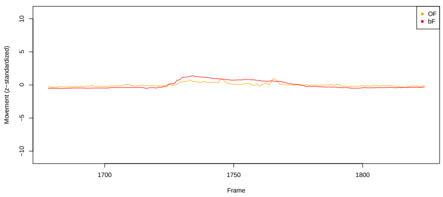

Subject 8, Anger

Participant 8 Anger, X_21, BL2_x

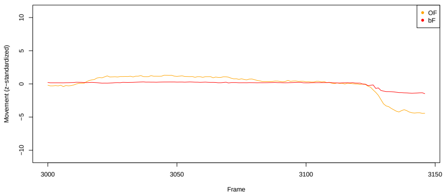

Participant 8 Anger, X_22, BR2_x

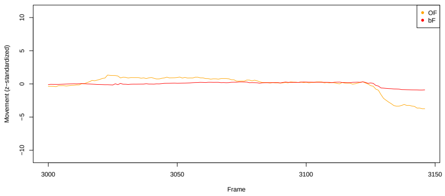

Participant 8 Anger, Y_21, BL2_y

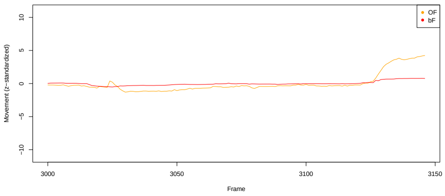

Participant 8 Anger, Y_22, BR2_y

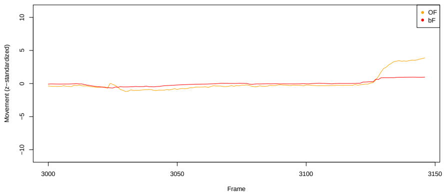

Participant 8 Anger, X_49, BL7_x

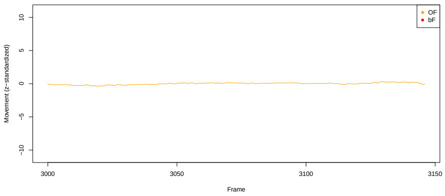

Participant 8 Anger, X_53, BR7_x

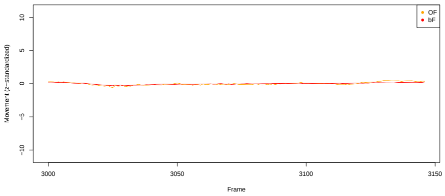

Participant 8 Anger, Y_49, BL7_y

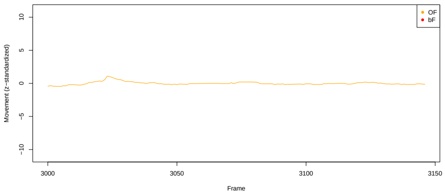

Participant 8 Anger, Y_53, BR7_y

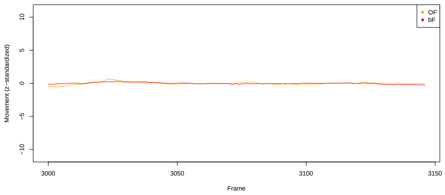

Participant 8 Anger, X_48, CL7_x

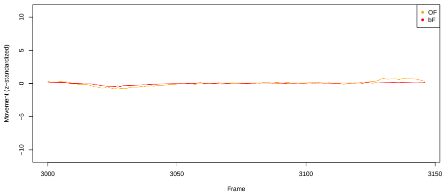

Participant 8 Anger, X_54, CR7_x

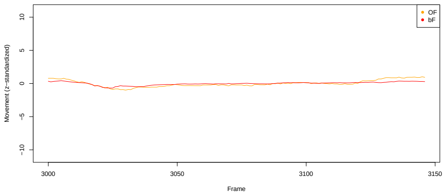

Participant 8 Anger, Y_48, CL7_y

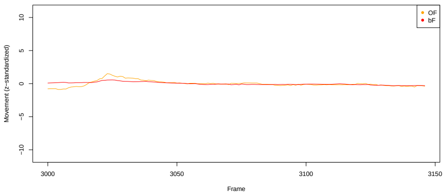

Participant 8 Anger, Y_54, CR7_y

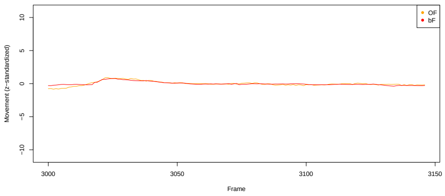

Participant 8 Anger, X_51, A7_x

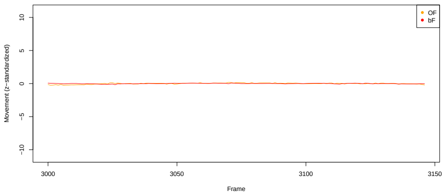

Participant 8 Anger, Y_51, A7_y

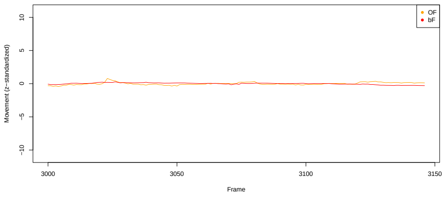

Subject 8, Anxiety

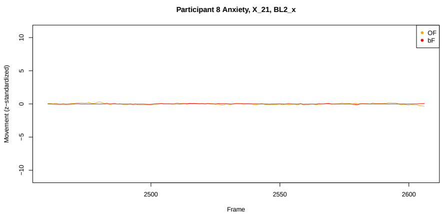

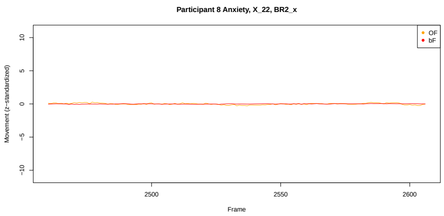

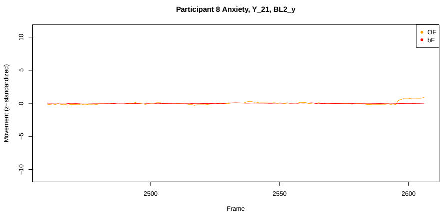

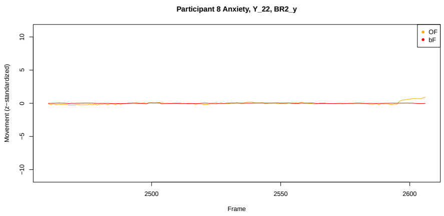

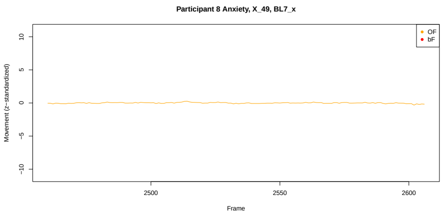

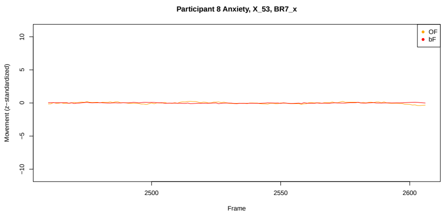

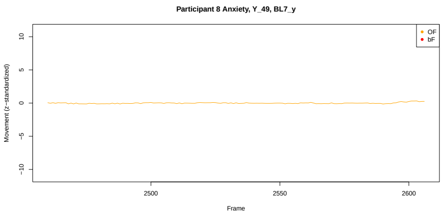

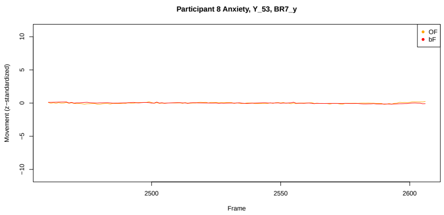

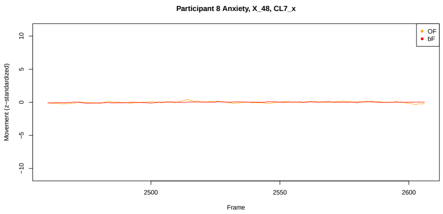

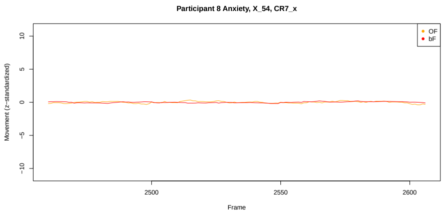

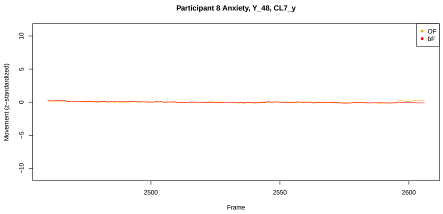

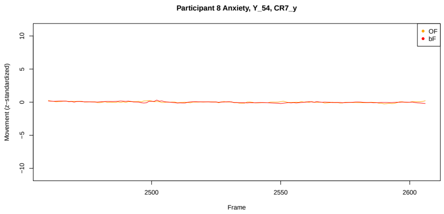

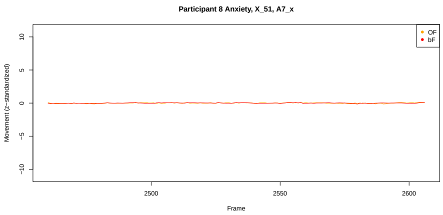

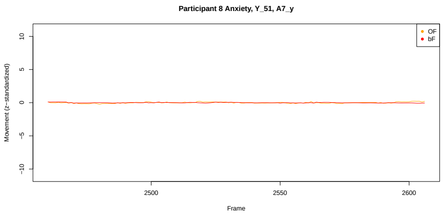

Subject 8, Disgust

Participant 8 Disgust, X_21, BL2_x

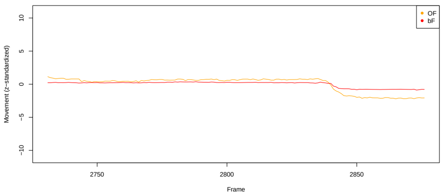

Participant 8 Disgust, X_22, BR2_x

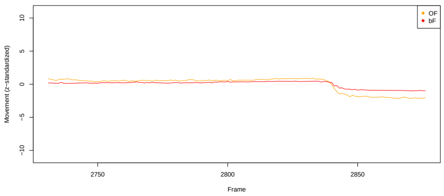

Participant 8 Disgust, Y_21, BL2_y

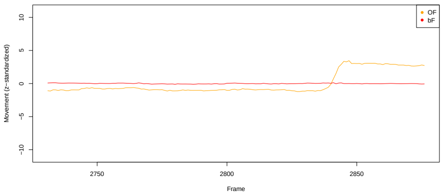

Participant 8 Disgust, Y_22, BR2_y

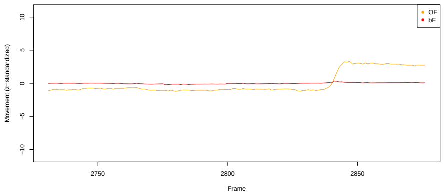

Participant 8 Disgust, X_49, BL7_x

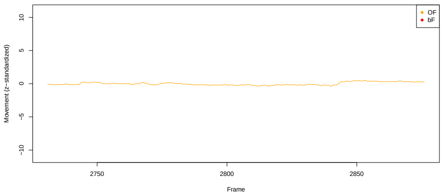

Participant 8 Disgust, X_53, BR7_x

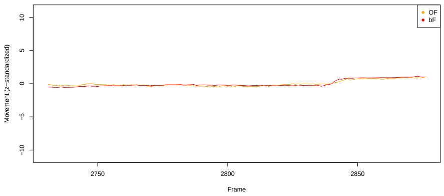

Participant 8 Disgust, Y_49, BL7_y

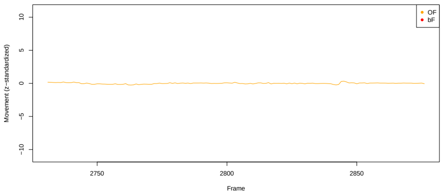

Participant 8 Disgust, Y_53, BR7_y

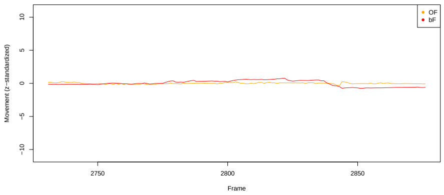

Participant 8 Disgust, X_48, CL7_x

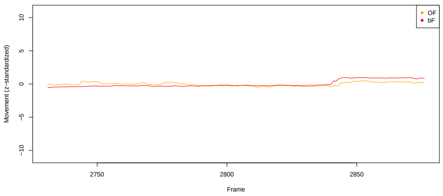

Participant 8 Disgust, X_54, CR7_x

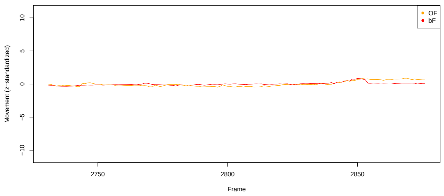

Participant 8 Disgust, Y_48, CL7_y

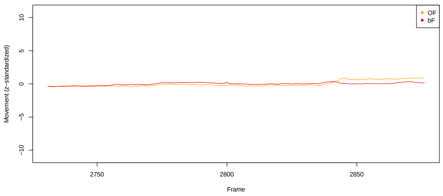

Participant 8 Disgust, Y_54, CR7_y

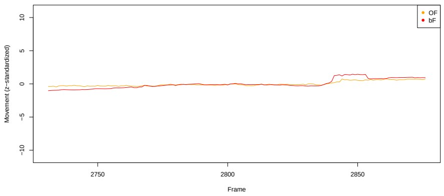

Participant 8 Disgust, X_51, A7_x

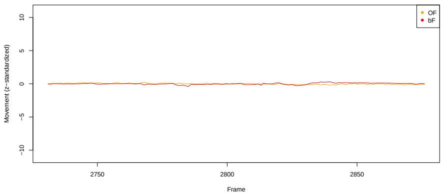

Participant 8 Disgust, Y_51, A7_y

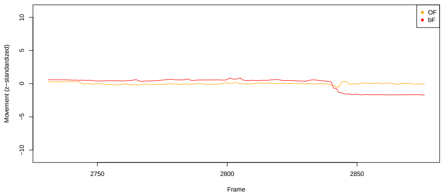

Subject 8, Happiness

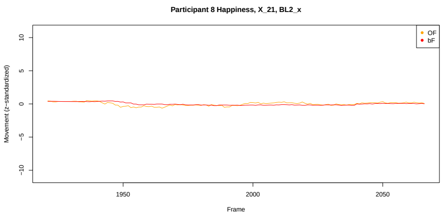

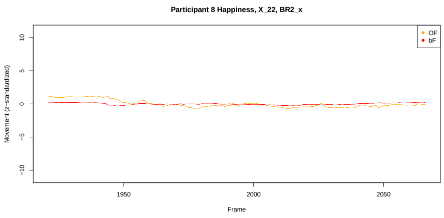

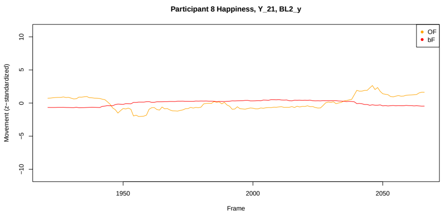

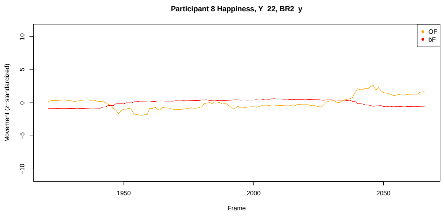

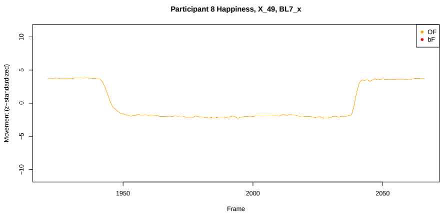

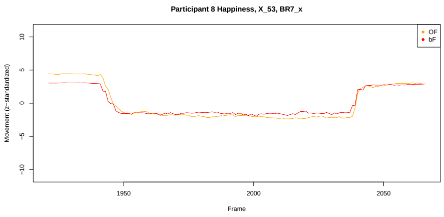

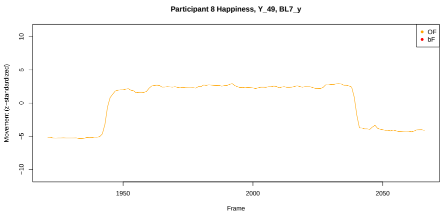

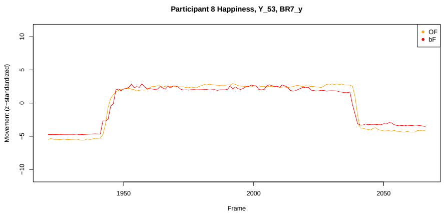

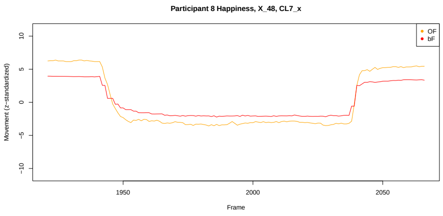

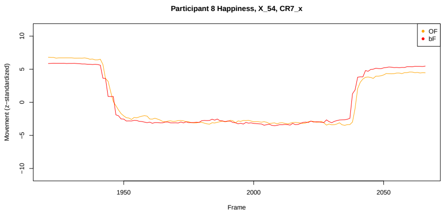

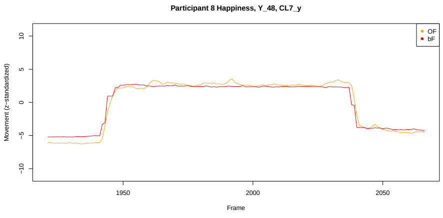

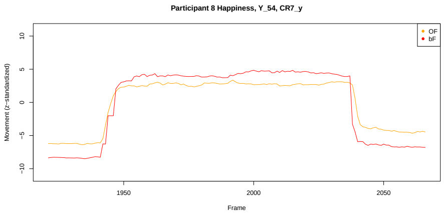

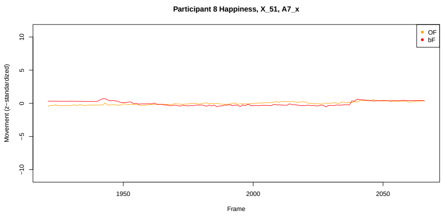

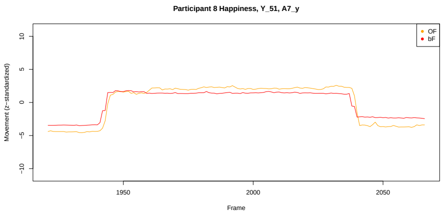

Subject 8, Sadness

Participant 8 Sadness, X_21, BL2_x

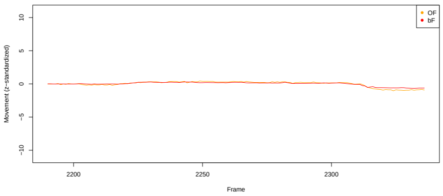

Participant 8 Sadness, X_22, BR2_x

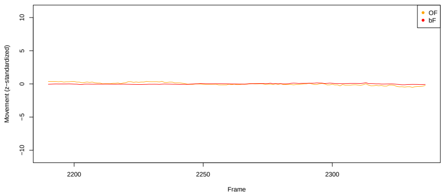

Participant 8 Sadness, Y_21, BL2_y

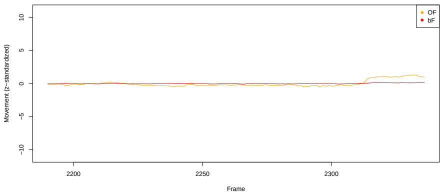

Participant 8 Sadness, Y_22, BR2_y

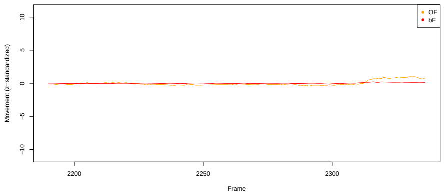

Participant 8 Sadness, X_49, BL7_x

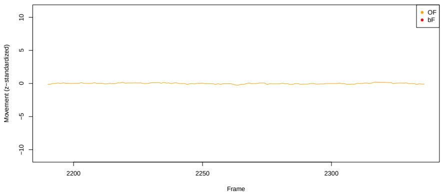

Participant 8 Sadness, X_53, BR7_x

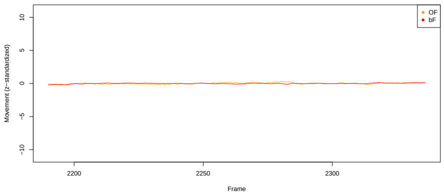

Participant 8 Sadness, Y_49, BL7_y

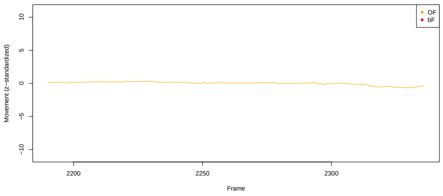

Participant 8 Sadness, Y_53, BR7_y

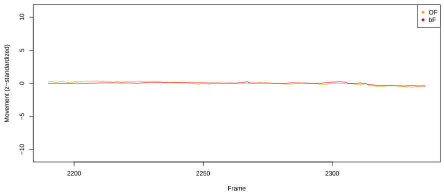

Participant 8 Sadness, X_48, CL7_x

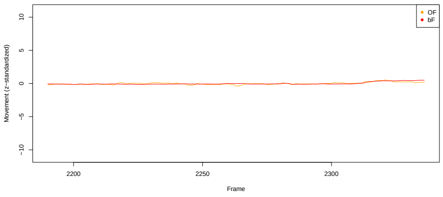

Participant 8 Sadness, X_54, CR7_x

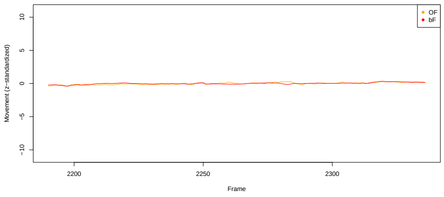

Participant 8 Sadness, Y_48, CL7_y

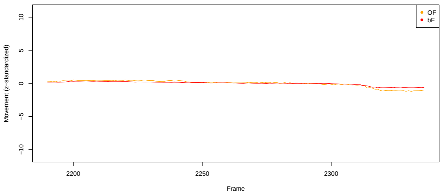

Participant 8 Sadness, Y_54, CR7_y

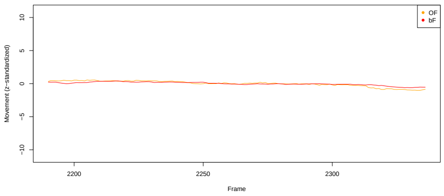

Participant 8 Sadness, X_51, A7_x

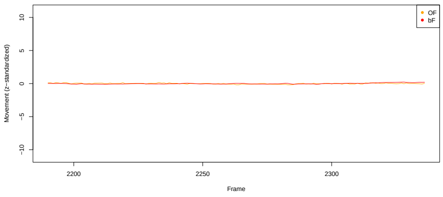

Participant 8 Sadness, Y_51, A7_y

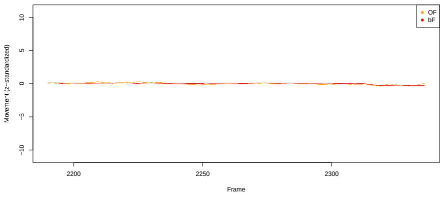

Subject 14, Anger

Participant 14 Anger, X_21, BL2_x

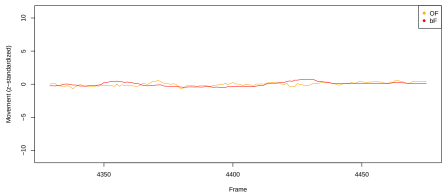

Participant 14 Anger, X_22, BR2_x

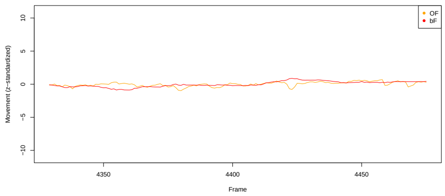

Participant 14 Anger, Y_21, BL2_y

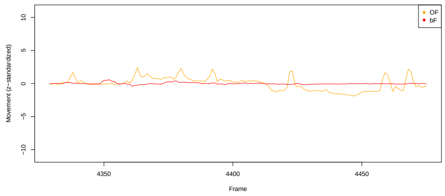

Participant 14 Anger, Y_22, BR2_y

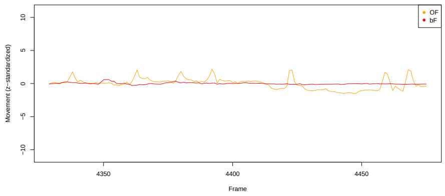

Participant 14 Anger, X_49, BL7_x

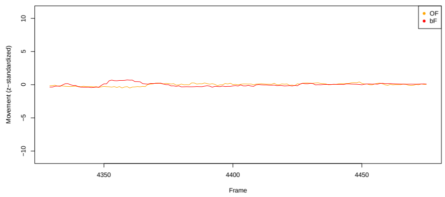

Participant 14 Anger, X_53, BR7_x

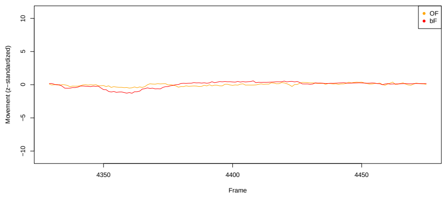

Participant 14 Anger, Y_49, BL7_y

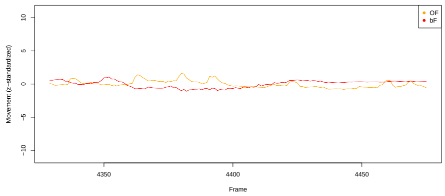

Participant 14 Anger, Y_53, BR7_y

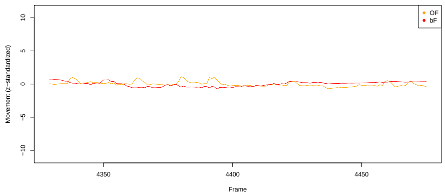

Participant 14 Anger, X_48, CL7_x

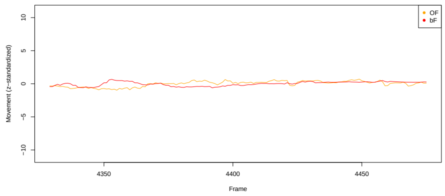

Participant 14 Anger, X_54, CR7_x

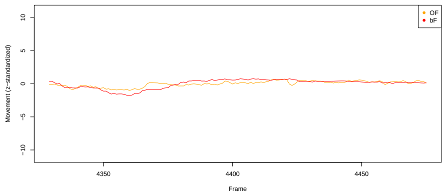

Participant 14 Anger, Y_48, CL7_y

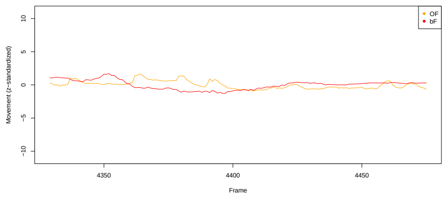

Participant 14 Anger, Y_54, CR7_y

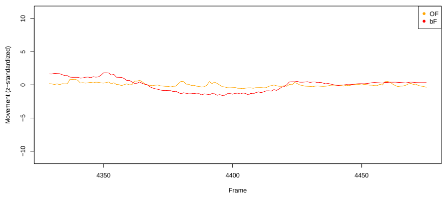

Participant 14 Anger, X_51, A7_x

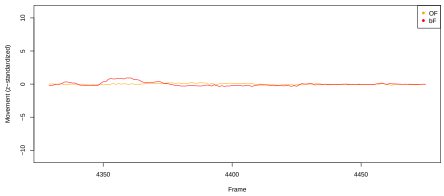

Participant 14 Anger, Y_51, A7_y

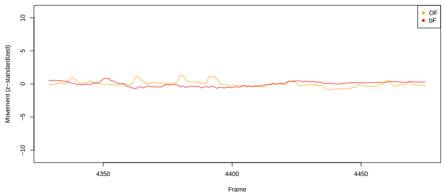

Subject 14, Anxiety

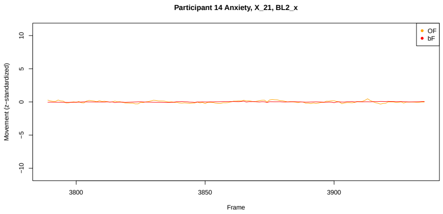

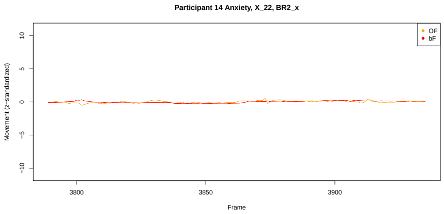

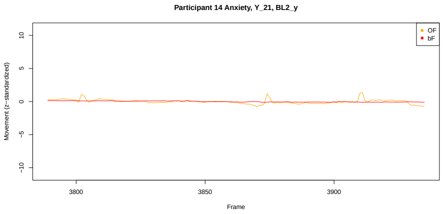

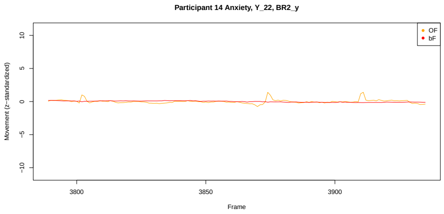

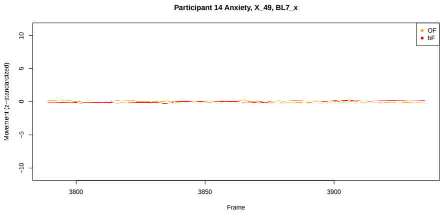

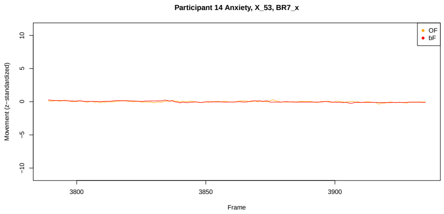

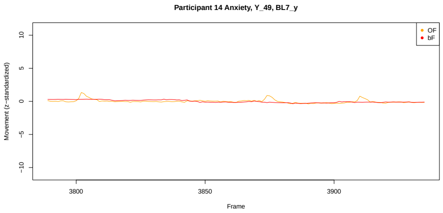

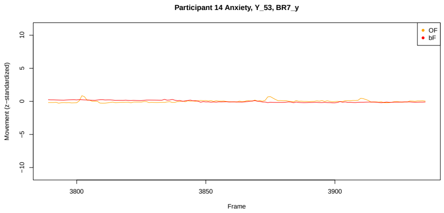

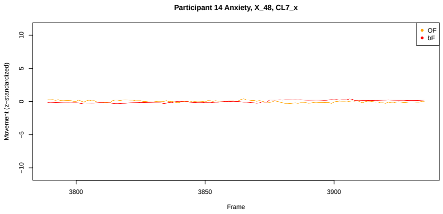

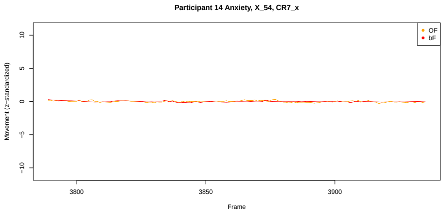

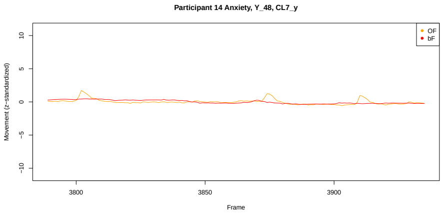

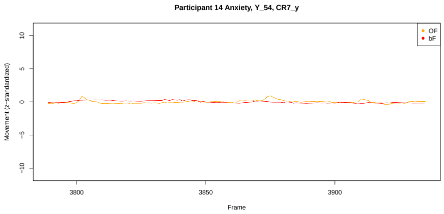

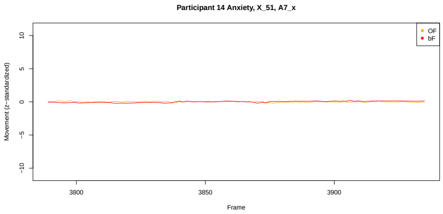

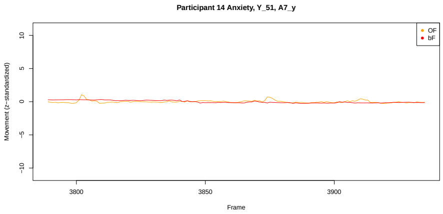

Subject 14, Disgust

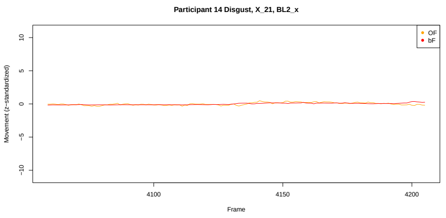

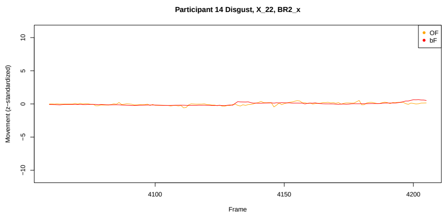

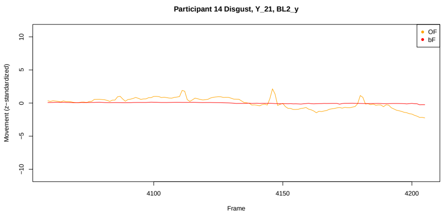

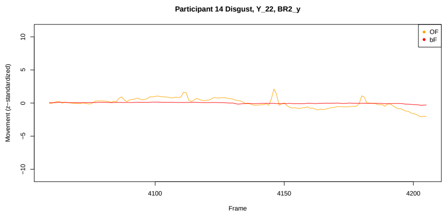

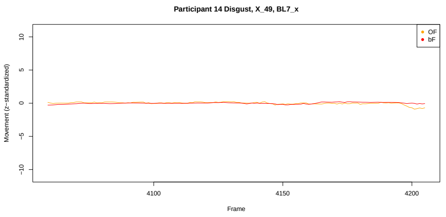

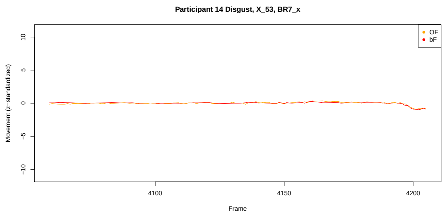

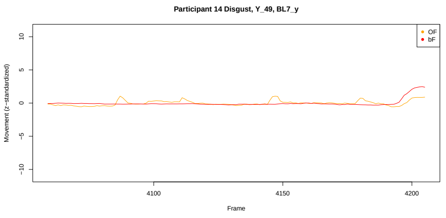

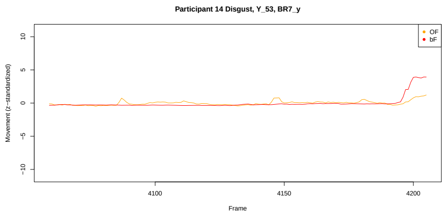

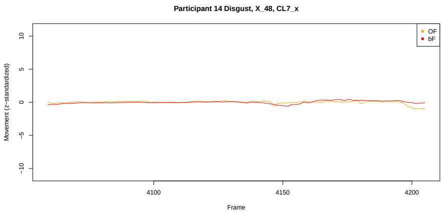

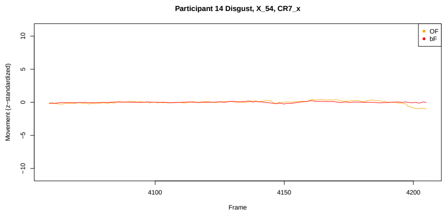

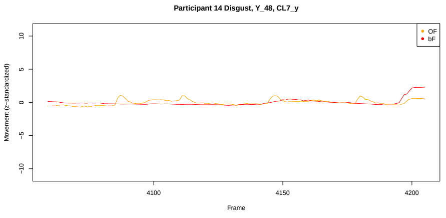

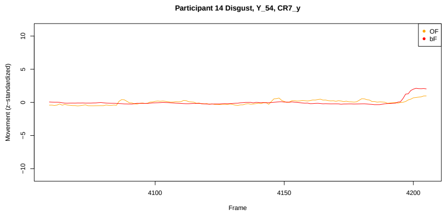

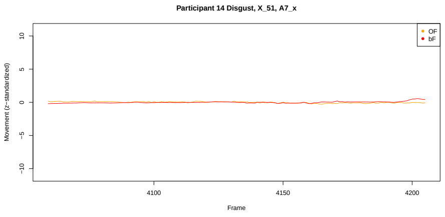

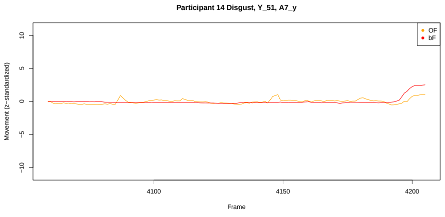

Subject 14, Happiness

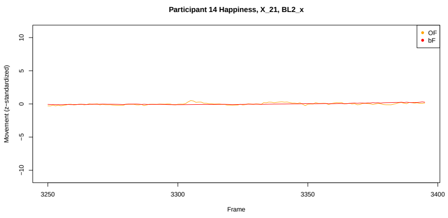

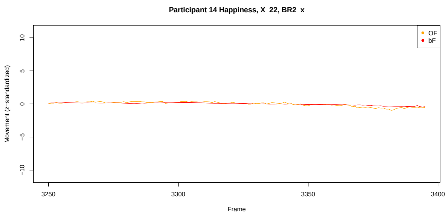

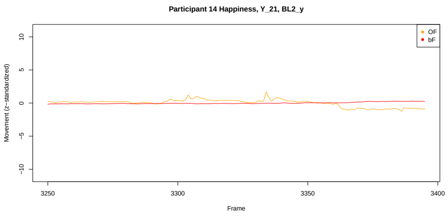

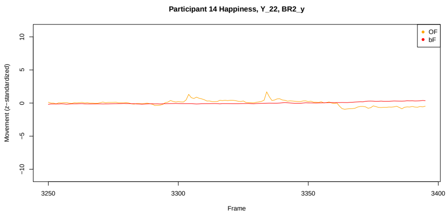

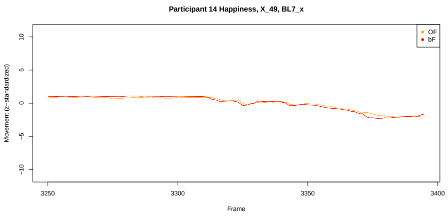

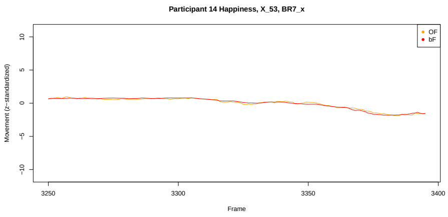

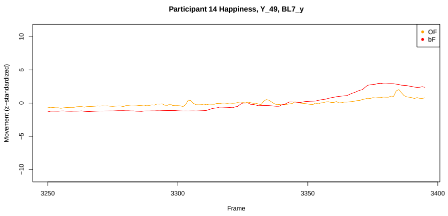

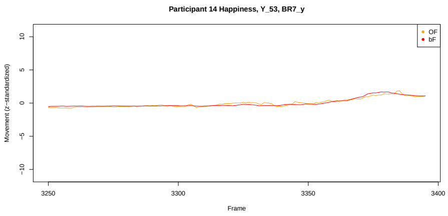

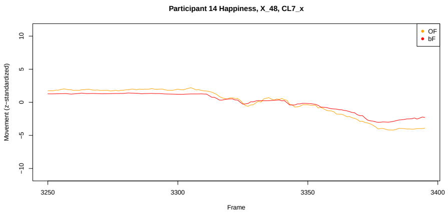

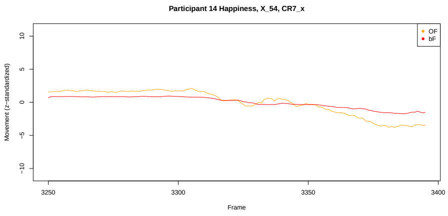

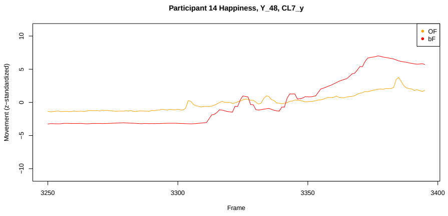

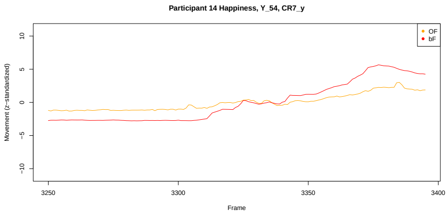

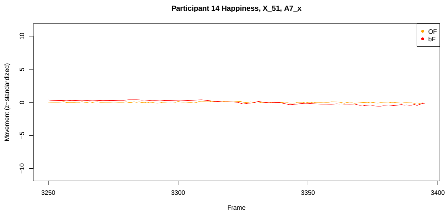

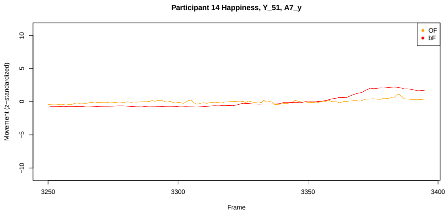

Subject 14, Sadness

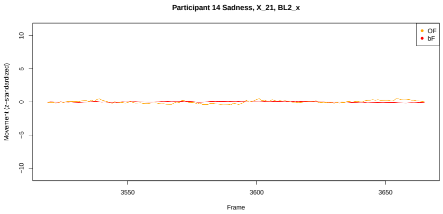

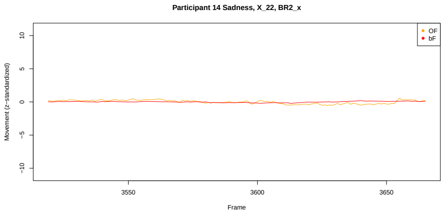

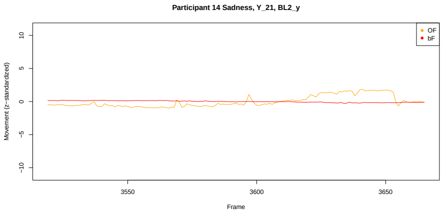

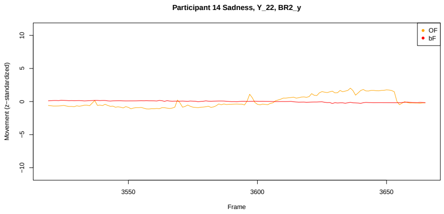

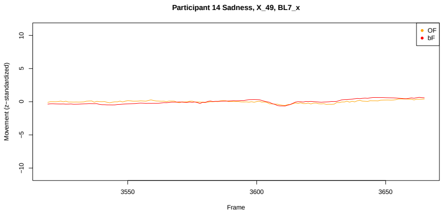

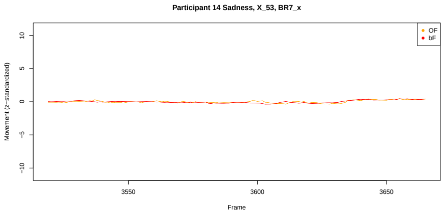

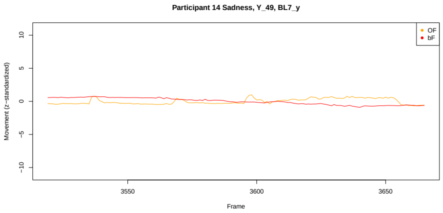

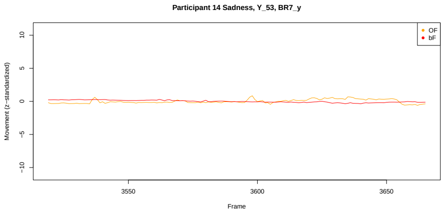

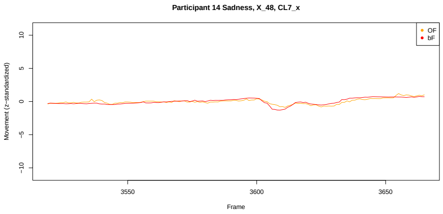

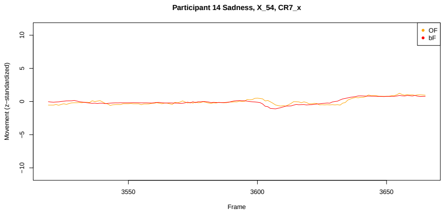

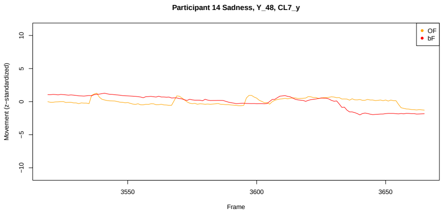

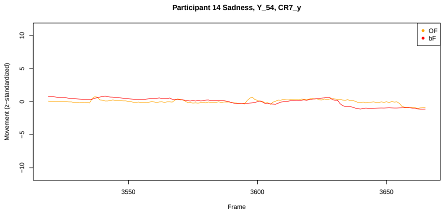

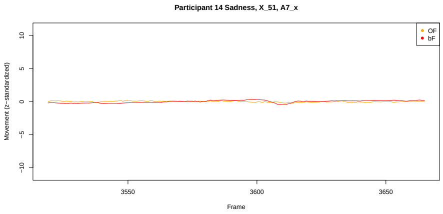

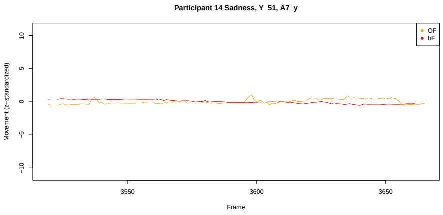

Subject 15, Anger

Participant 15 Anger, X_21, BL2_x

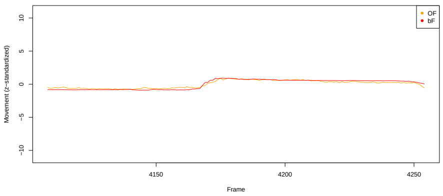

Participant 15 Anger, X_22, BR2_x

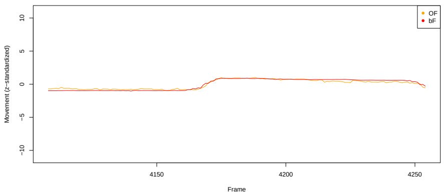

Participant 15 Anger, Y_21, BL2_y

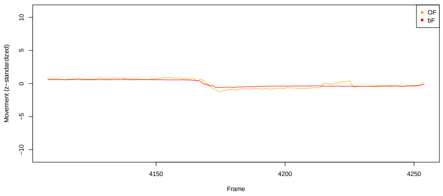

Participant 15 Anger, Y_22, BR2_y

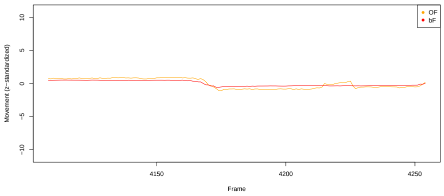

Participant 15 Anger, X_49, BL7_x

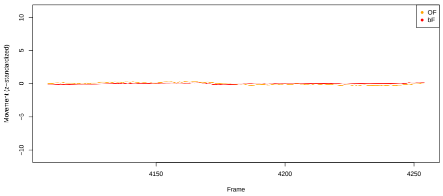

Participant 15 Anger, X_53, BR7_x

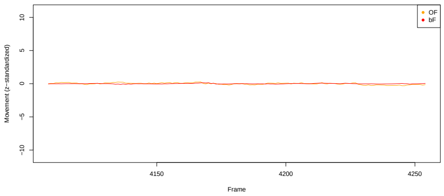

Participant 15 Anger, Y_49, BL7_y

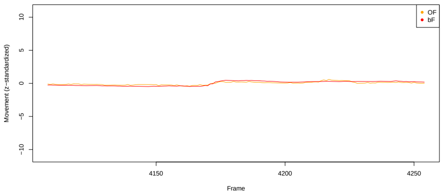

Participant 15 Anger, Y_53, BR7_y

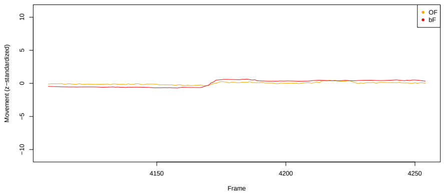

Participant 15 Anger, X_48, CL7_x

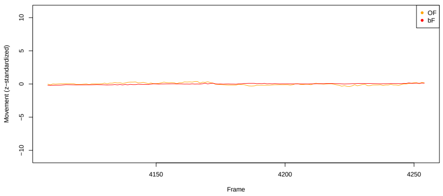

Participant 15 Anger, X_54, CR7_x

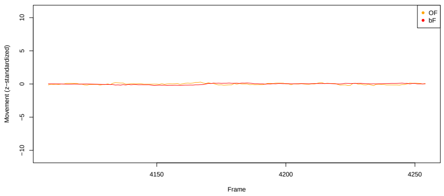

Participant 15 Anger, Y_48, CL7_y

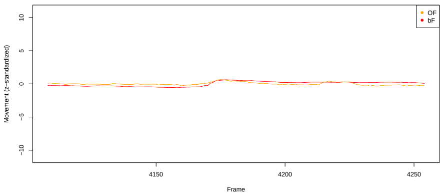

Participant 15 Anger, Y_54, CR7_y

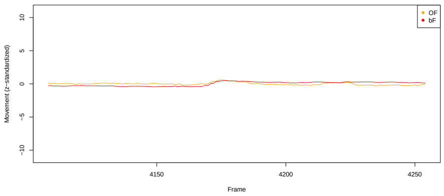

Participant 15 Anger, X_51, A7_x

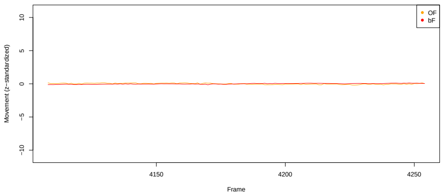

Participant 15 Anger, Y_51, A7_y

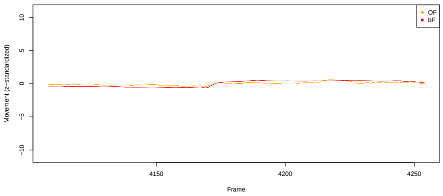

Subject 15, Anxiety

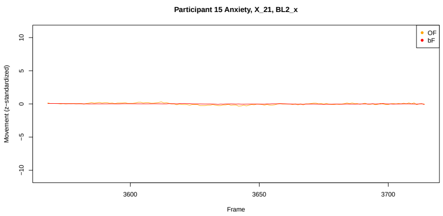

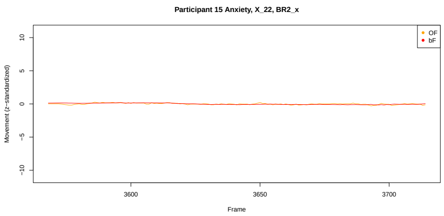

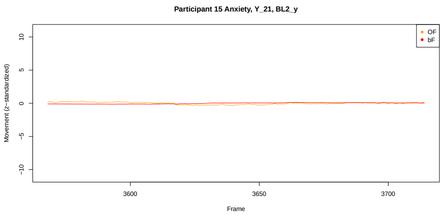

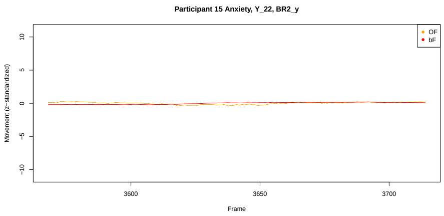

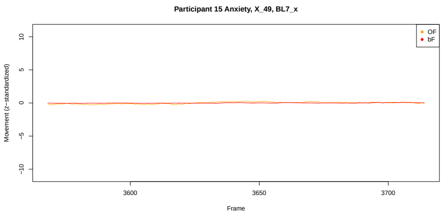

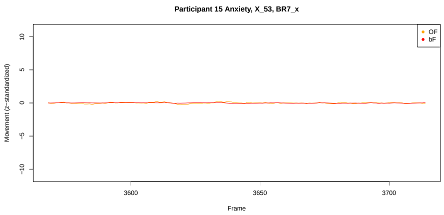

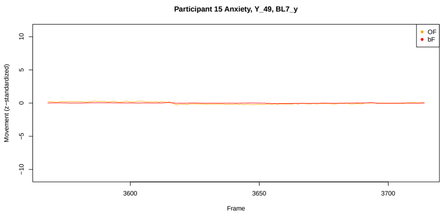

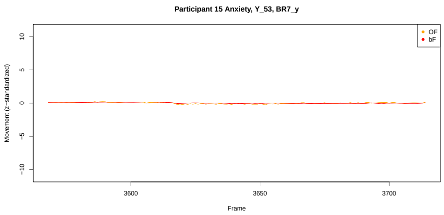

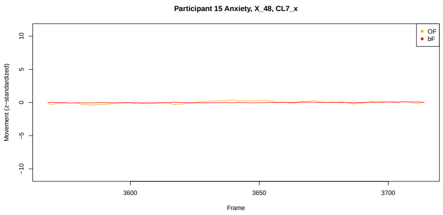

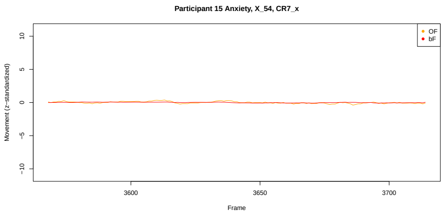

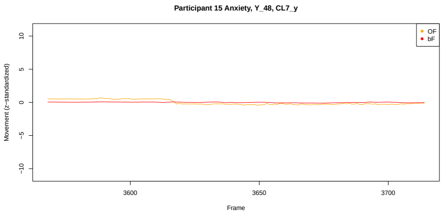

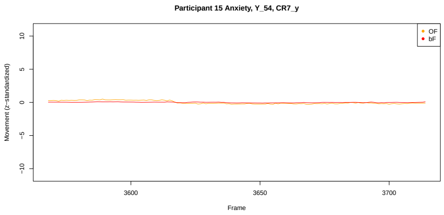

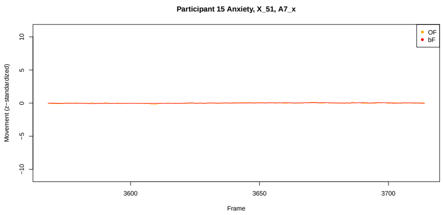

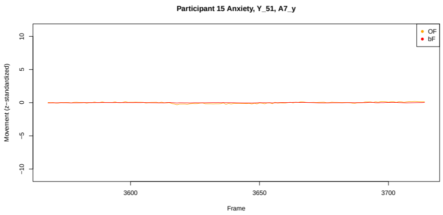

Subject 15, Disgust

Participant 15 Disgust, X_21, BL2_x

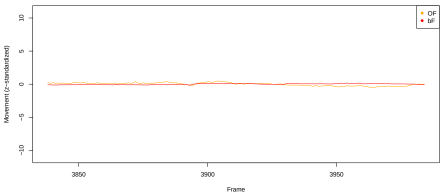

Participant 15 Disgust, X_22, BR2_x

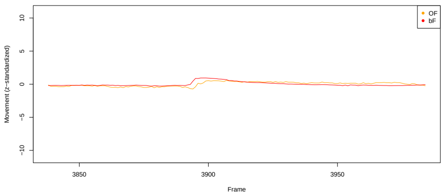

Participant 15 Disgust, Y_21, BL2_y

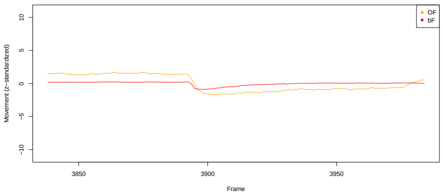

Participant 15 Disgust, Y_22, BR2_y

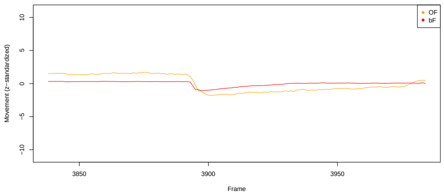

Participant 15 Disgust, X_49, BL7_x

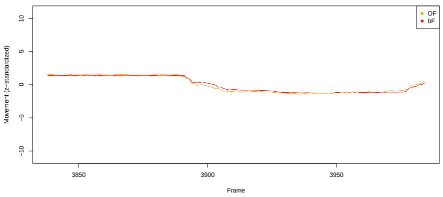

Participant 15 Disgust, X_53, BR7_x

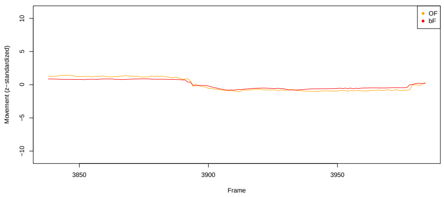

Participant 15 Disgust, Y_49, BL7_y

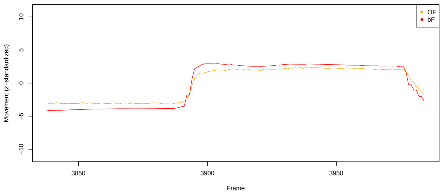

Participant 15 Disgust, Y_53, BR7_y

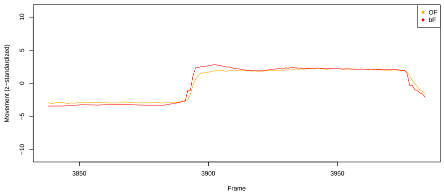

Participant 15 Disgust, X_48, CL7_x

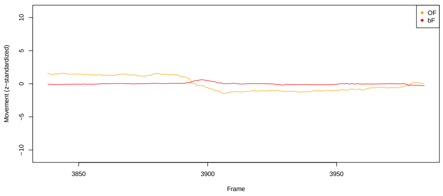

Participant 15 Disgust, X_54, CR7_x

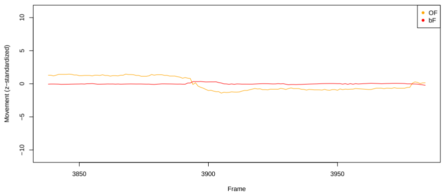

Participant 15 Disgust, Y_48, CL7_y

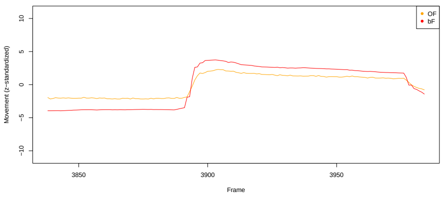

Participant 15 Disgust, Y_54, CR7_y

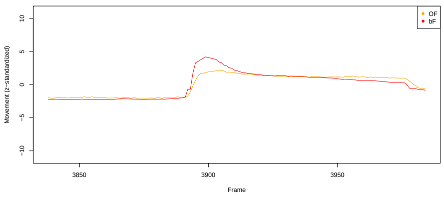

Participant 15 Disgust, X_51, A7_x

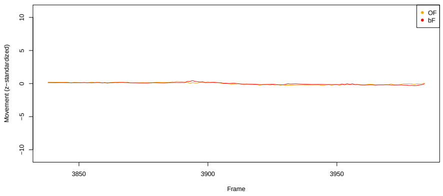

Participant 15 Disgust, Y_51, A7_y

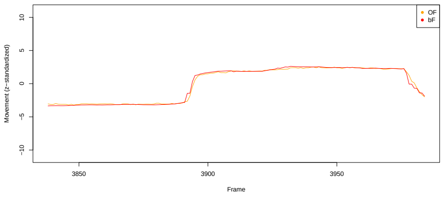

Subject 15, Happiness

Participant 15 Happiness, X_21, BL2_x

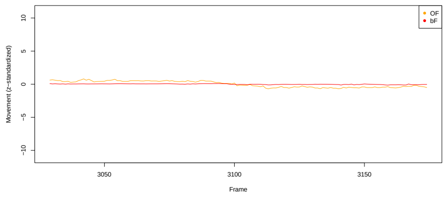

Participant 15 Happiness, X_22, BR2_x

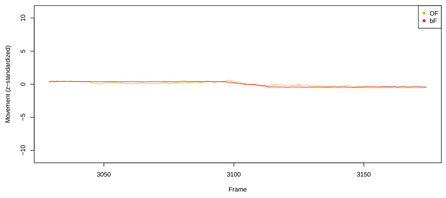

Participant 15 Happiness, Y_21, BL2_y

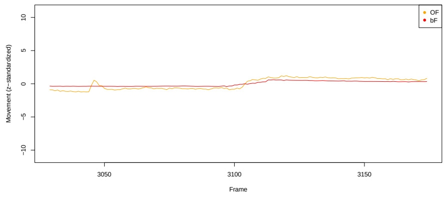

Participant 15 Happiness, Y_22, BR2_y

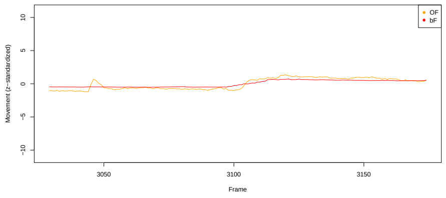

Participant 15 Happiness, X_49, BL7_x

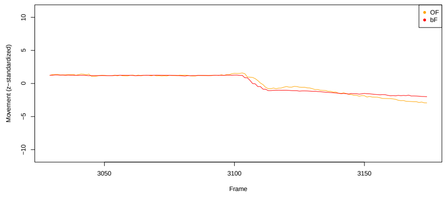

Participant 15 Happiness, X_53, BR7_x

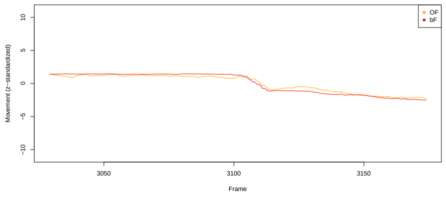

Participant 15 Happiness, Y_49, BL7_y

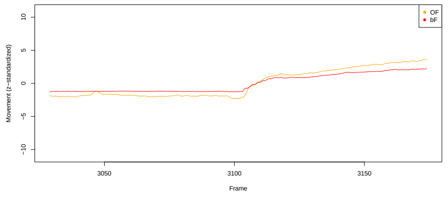

Participant 15 Happiness, Y_53, BR7_y

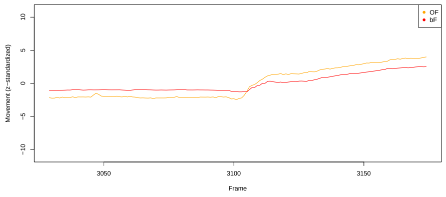

Participant 15 Happiness, X_48, CL7_x

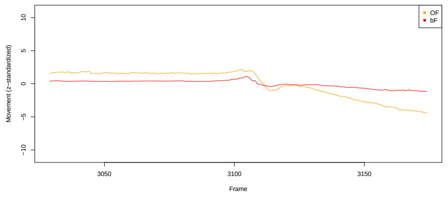

Participant 15 Happiness, X_54, CR7_x

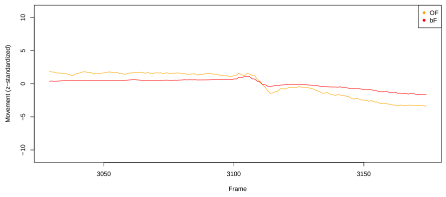

Participant 15 Happiness, Y_48, CL7_y

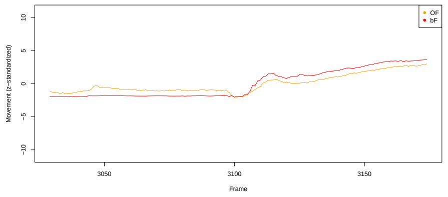

Participant 15 Happiness, Y_54, CR7_y

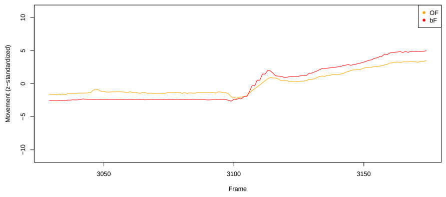

Participant 15 Happiness, X_51, A7_x

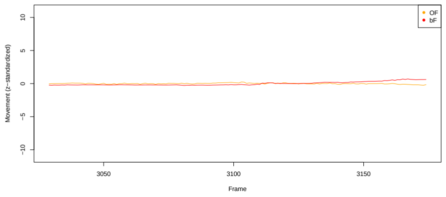

Participant 15 Happiness, Y_51, A7_y

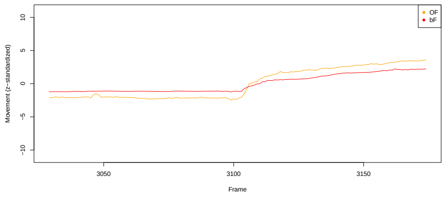

Subject 15, Sadness

Participant 15 Sadness, X_21, BL2_x

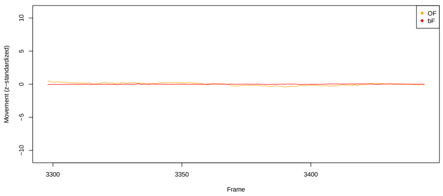

Participant 15 Sadness, X_22, BR2_x

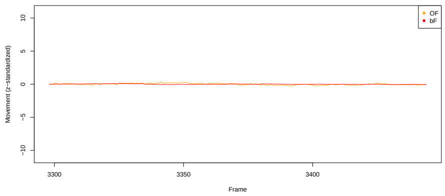

Participant 15 Sadness, Y_21, BL2_y

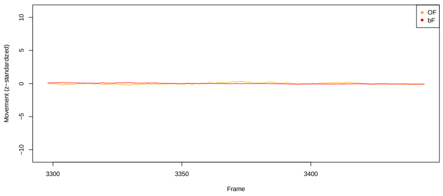

Participant 15 Sadness, Y_22, BR2_y

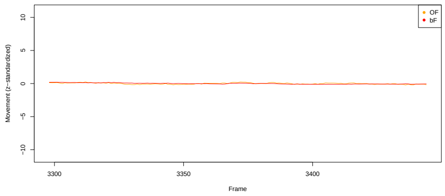

Participant 15 Sadness, X_49, BL7_x

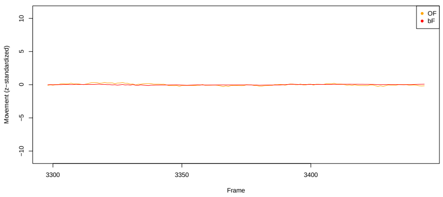

Participant 15 Sadness, X_53, BR7_x

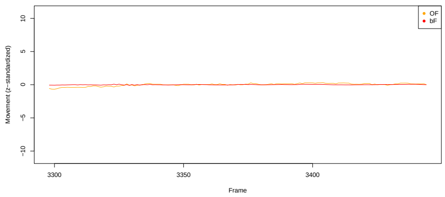

Participant 15 Sadness, Y_49, BL7_y

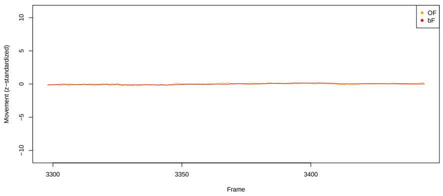

Participant 15 Sadness, Y_53, BR7_y

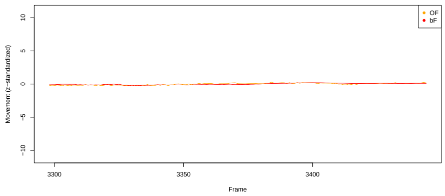

Participant 15 Sadness, X_48, CL7_x

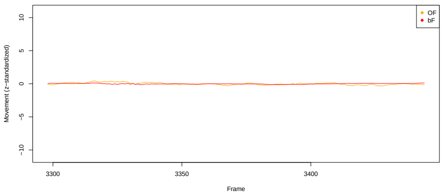

Participant 15 Sadness, X_54, CR7_x

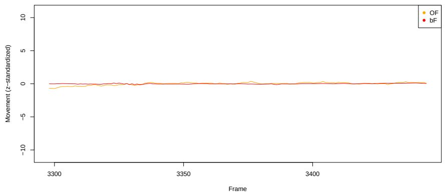

Participant 15 Sadness, Y_48, CL7_y

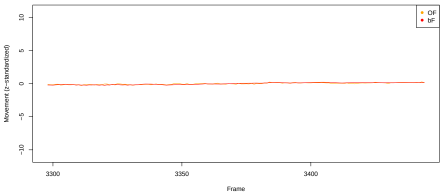

Participant 15 Sadness, Y_54, CR7_y

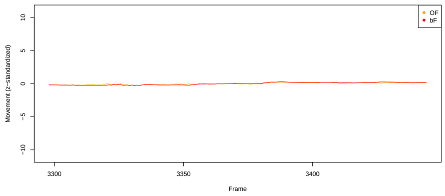

Participant 15 Sadness, X_51, A7_x

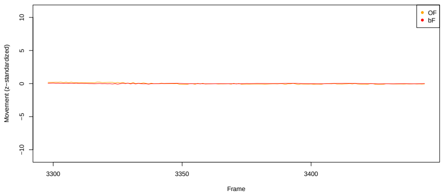

Participant 15 Sadness, Y_51, A7_y

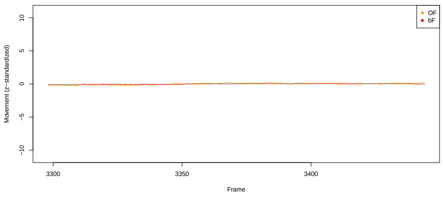

Subject 17, Anger

Participant 17 Anger, X_21, BL2_x

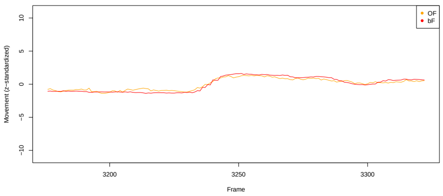

Participant 17 Anger, X_22, BR2_x

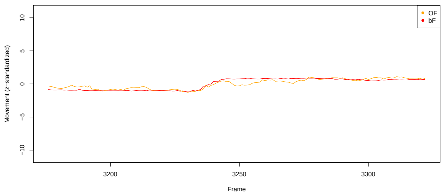

Participant 17 Anger, Y_21, BL2_y

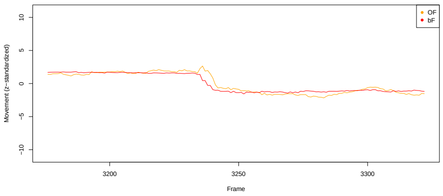

Participant 17 Anger, Y_22, BR2_y

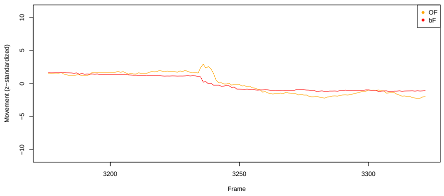

Participant 17 Anger, X_49, BL7_x

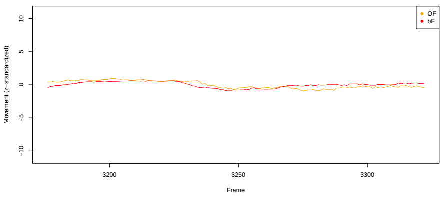

Participant 17 Anger, X_53, BR7_x

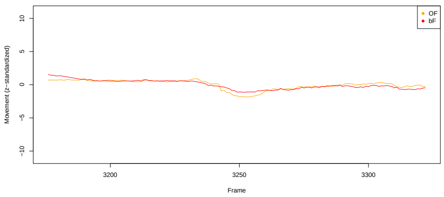

Participant 17 Anger, Y_49, BL7_y

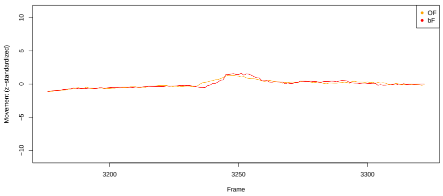

Participant 17 Anger, Y_53, BR7_y

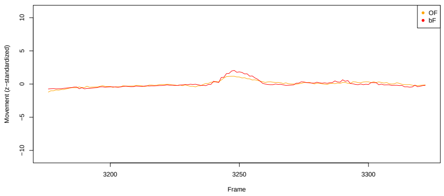

Participant 17 Anger, X_48, CL7_x

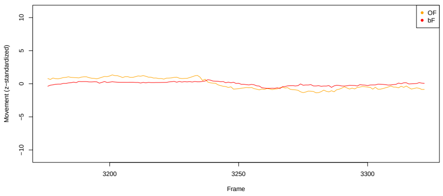

Participant 17 Anger, X_54, CR7_x

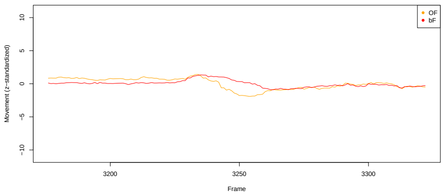

Participant 17 Anger, Y_48, CL7_y

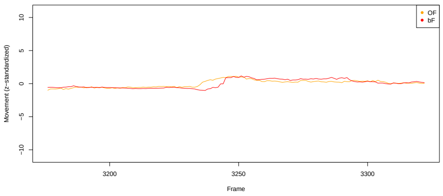

Participant 17 Anger, Y_54, CR7_y

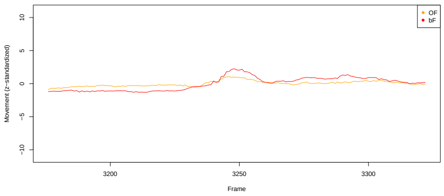

Participant 17 Anger, X_51, A7_x

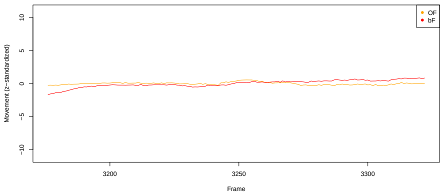

Participant 17 Anger, Y_51, A7_y

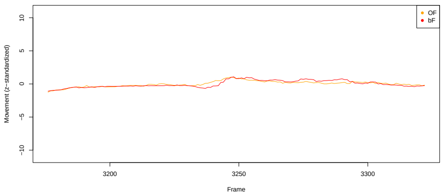

Subject 17, Anxiety

Participant 17 Anxiety, X_21, BL2_x

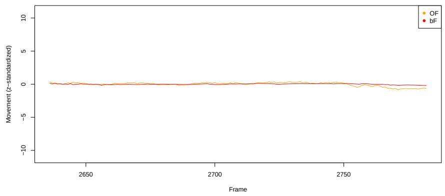

Participant 17 Anxiety, X_22, BR2_x

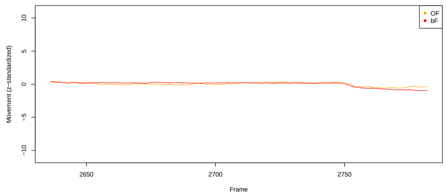

Participant 17 Anxiety, Y_21, BL2_y

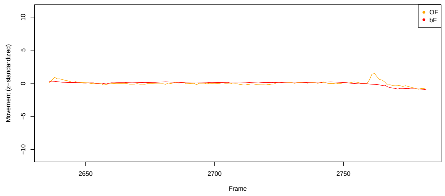

Participant 17 Anxiety, Y_22, BR2_y

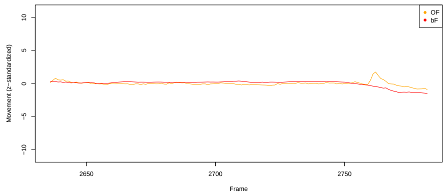

Participant 17 Anxiety, X_49, BL7_x

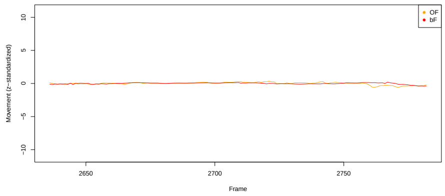

Participant 17 Anxiety, X_53, BR7_x

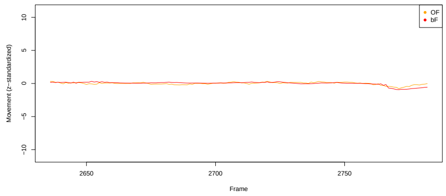

Participant 17 Anxiety, Y_49, BL7_y

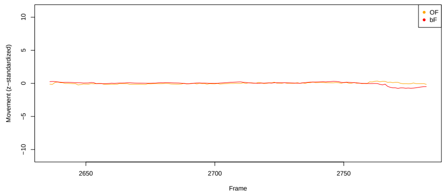

Participant 17 Anxiety, Y_53, BR7_y

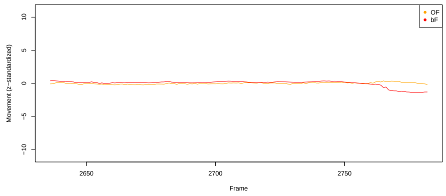

Participant 17 Anxiety, X_48, CL7_x

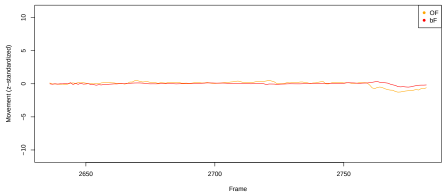

Participant 17 Anxiety, X_54, CR7_x

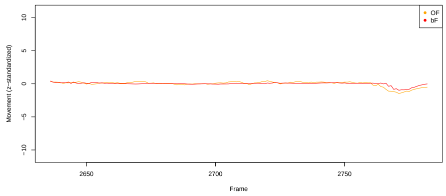

Participant 17 Anxiety, Y_48, CL7_y

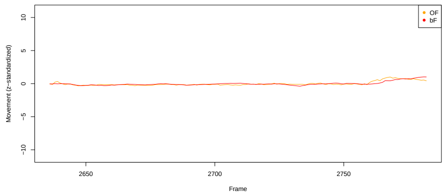

Participant 17 Anxiety, Y_54, CR7_y

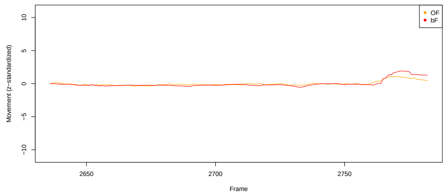

Participant 17 Anxiety, X_51, A7_x

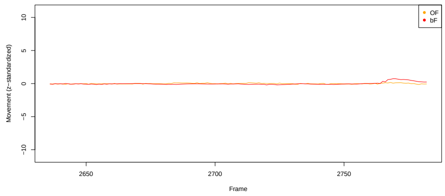

Participant 17 Anxiety, Y_51, A7_y

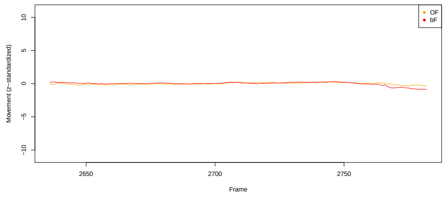

Subject 17, Disgust

Participant 17 Disgust, X_21, BL2_x

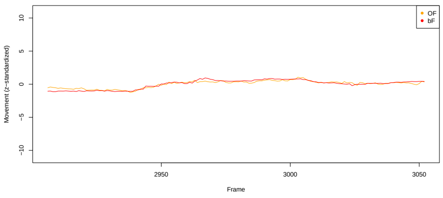

Participant 17 Disgust, X_22, BR2_x

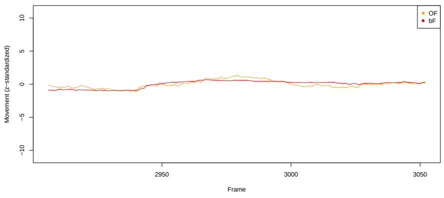

Participant 17 Disgust, Y_21, BL2_y

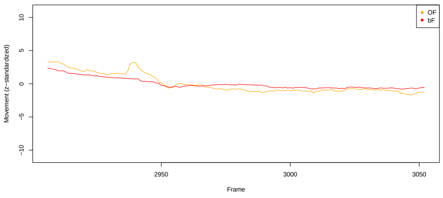

Participant 17 Disgust, Y_22, BR2_y

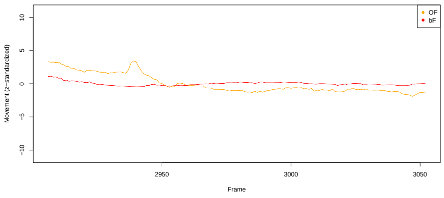

Participant 17 Disgust, X_49, BL7_x

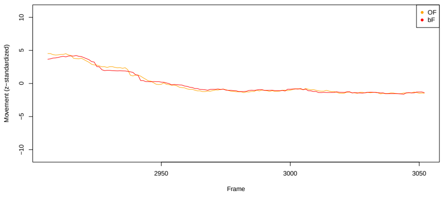

Participant 17 Disgust, X_53, BR7_x

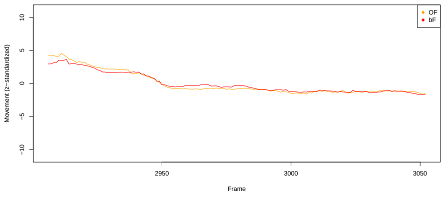

Participant 17 Disgust, Y_49, BL7_y

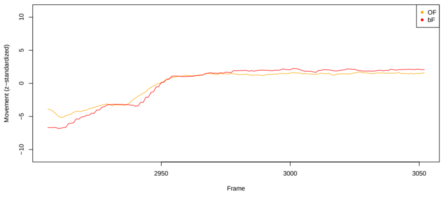

Participant 17 Disgust, Y_53, BR7_y

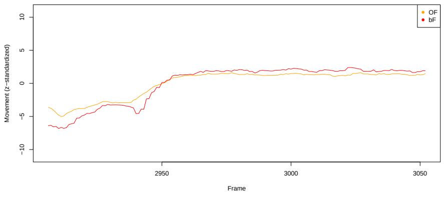

Participant 17 Disgust, X_48, CL7_x

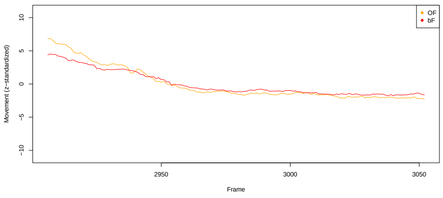

Participant 17 Disgust, X_54, CR7_x

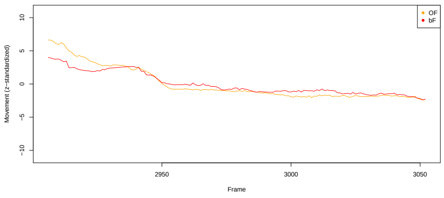

Participant 17 Disgust, Y_48, CL7_y

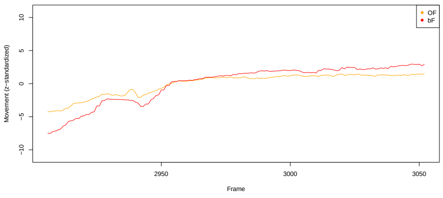

Participant 17 Disgust, Y_54, CR7_y

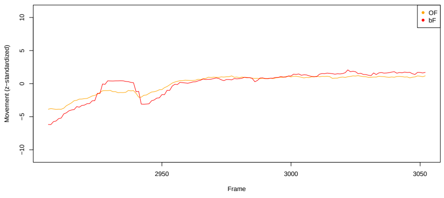

Participant 17 Disgust, X_51, A7_x

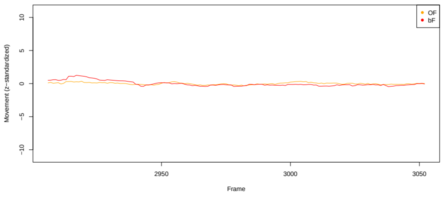

Participant 17 Disgust, Y_51, A7_y

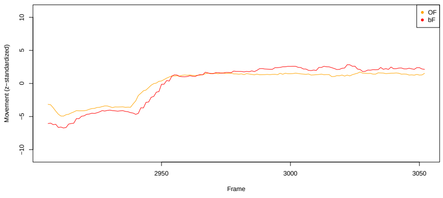

Subject 17, Happiness

Participant 17 Happiness, X_21, BL2_x

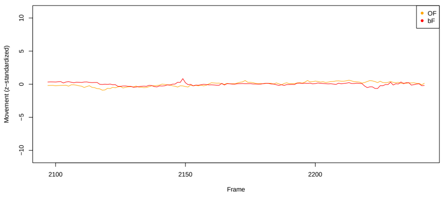

Participant 17 Happiness, X_22, BR2_x

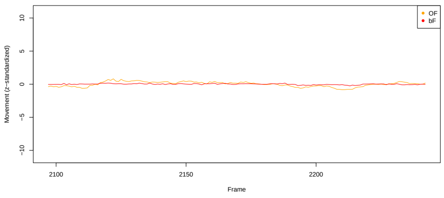

Participant 17 Happiness, Y_21, BL2_y

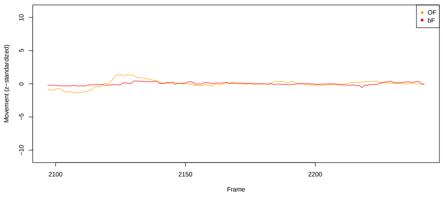

Participant 17 Happiness, Y_22, BR2_y

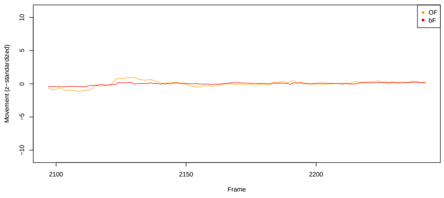

Participant 17 Happiness, X_49, BL7_x

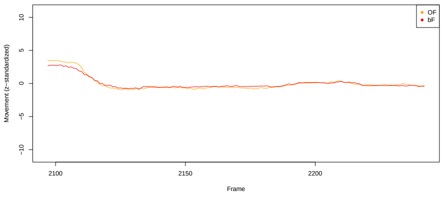

Participant 17 Happiness, X_53, BR7_x

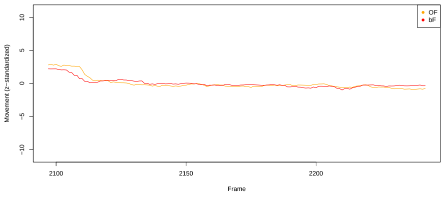

Participant 17 Happiness, Y_49, BL7_y

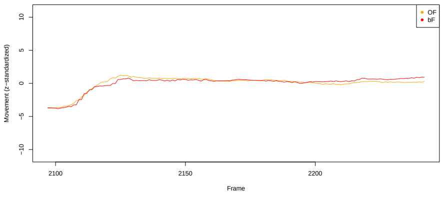

Participant 17 Happiness, Y_53, BR7_y

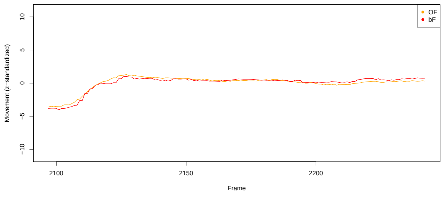

Participant 17 Happiness, X_48, CL7_x

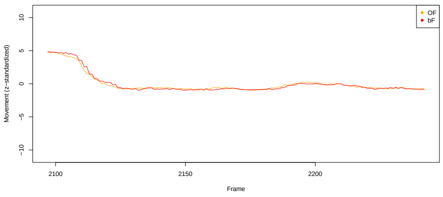

Participant 17 Happiness, X_54, CR7_x

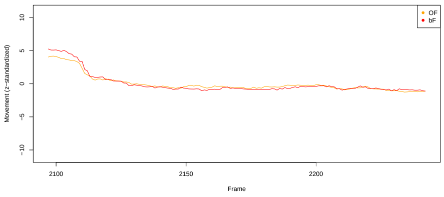

Participant 17 Happiness, Y_48, CL7_y

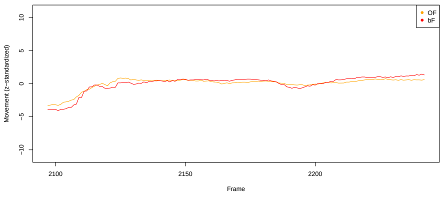

Participant 17 Happiness, Y_54, CR7_y

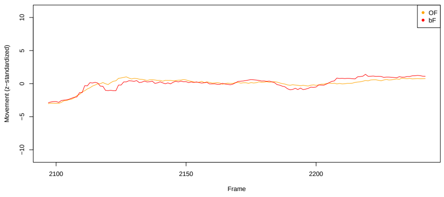

Participant 17 Happiness, X_51, A7_x

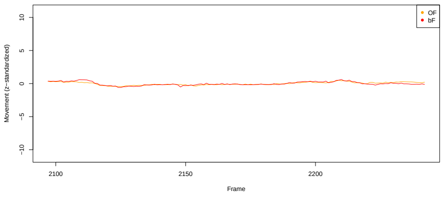

Participant 17 Happiness, Y_51, A7_y

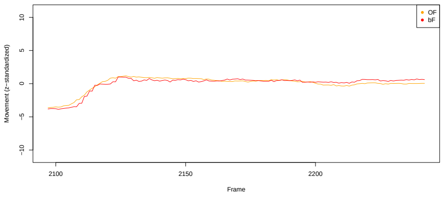

Subject 17, Sadness

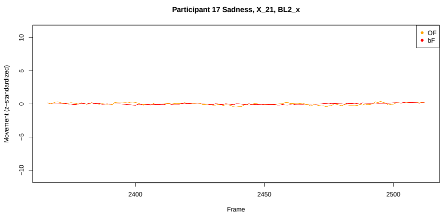

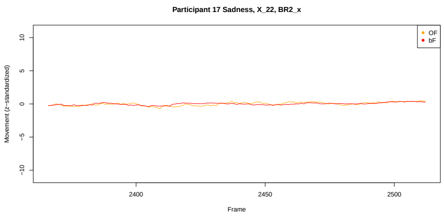

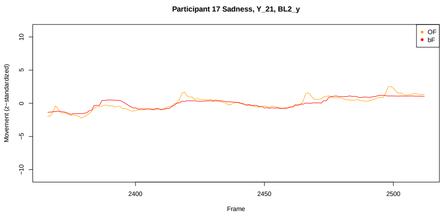

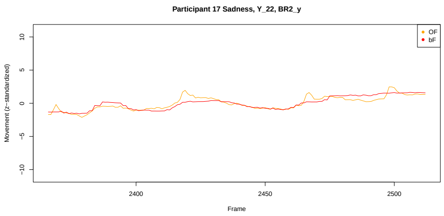

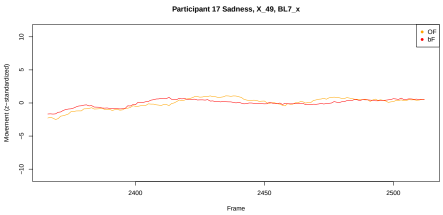

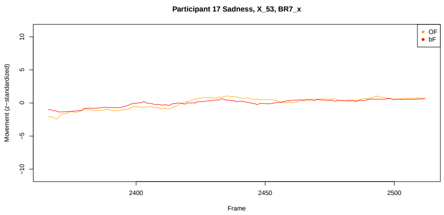

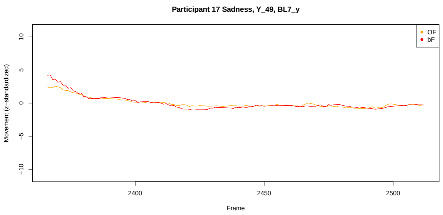

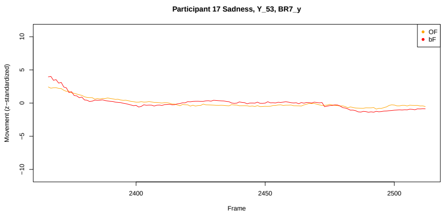

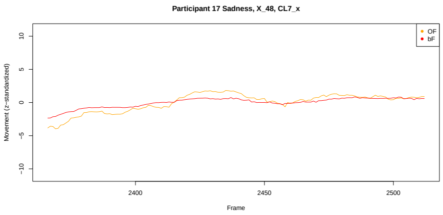

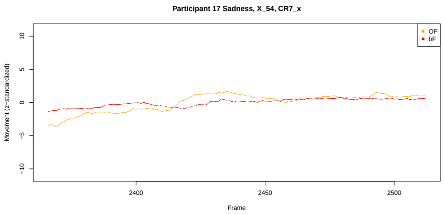

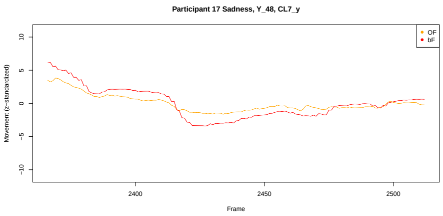

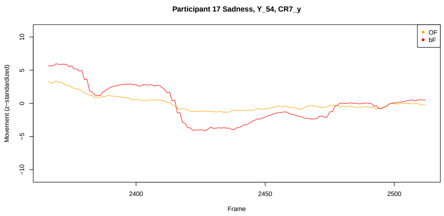

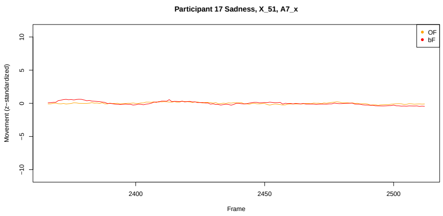

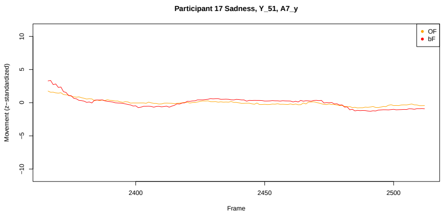

Subject 18, Anger

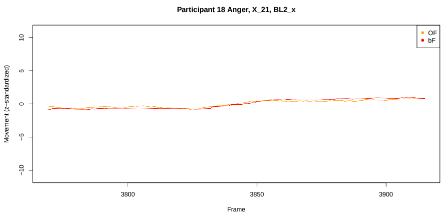

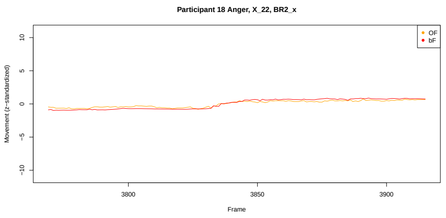

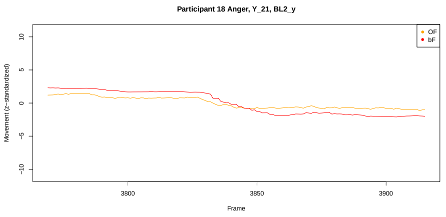

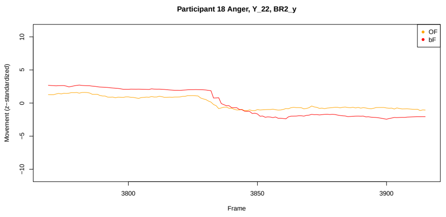

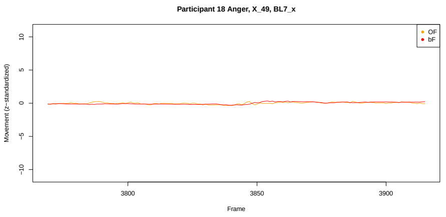

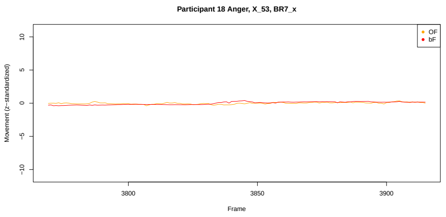

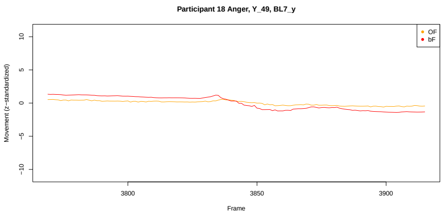

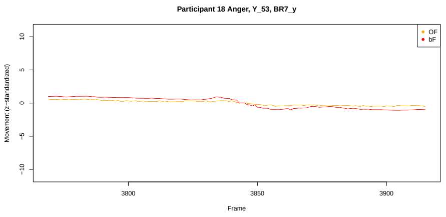

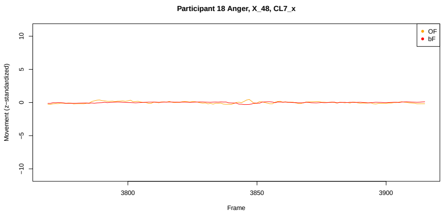

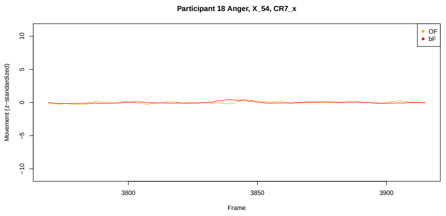

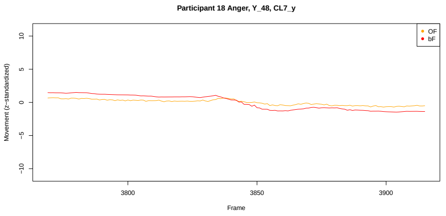

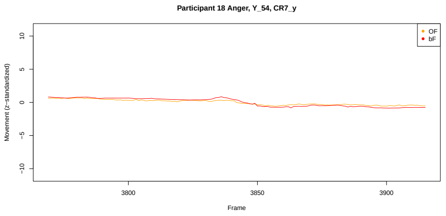

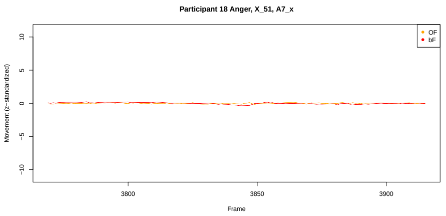

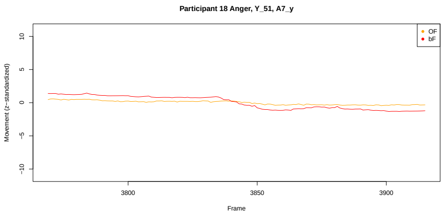

Subject 18, Anxiety

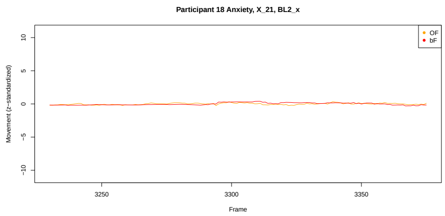

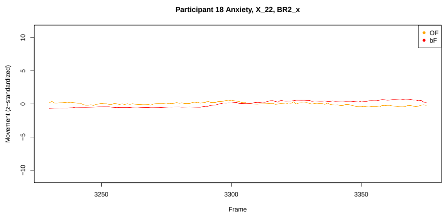

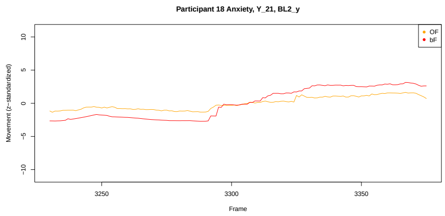

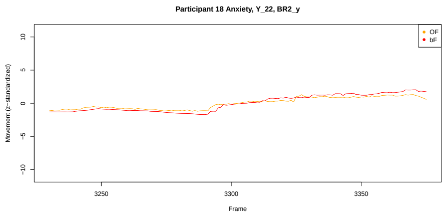

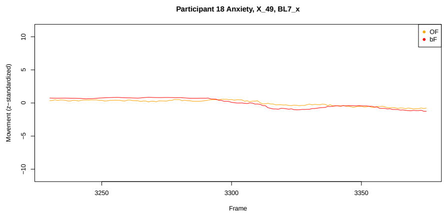

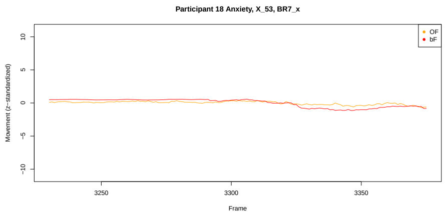

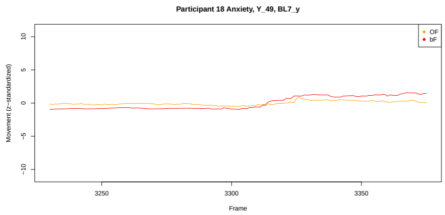

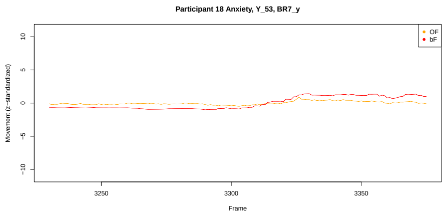

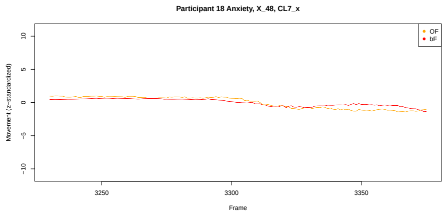

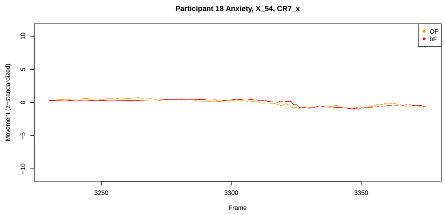

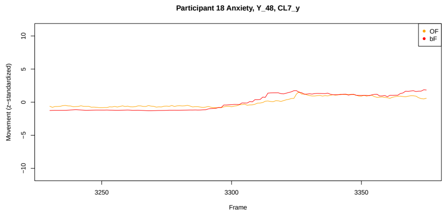

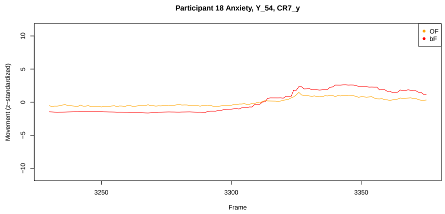

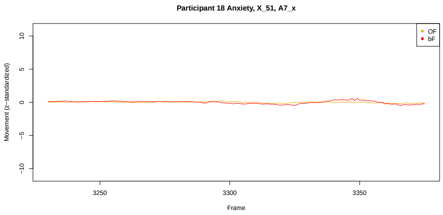

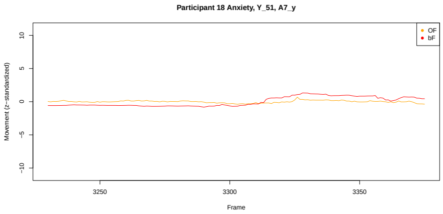

Subject 18, Disgust

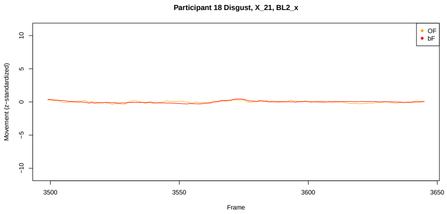

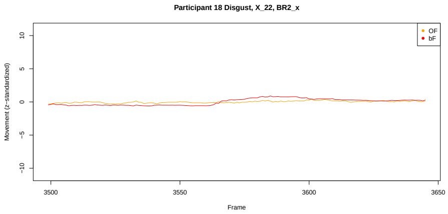

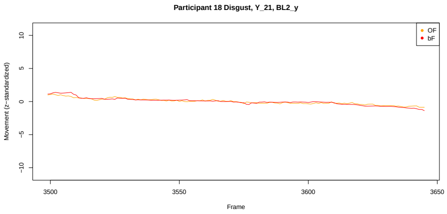

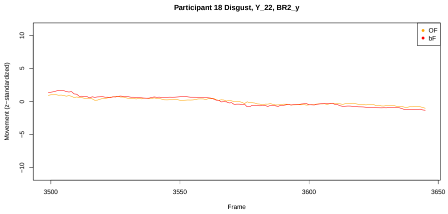

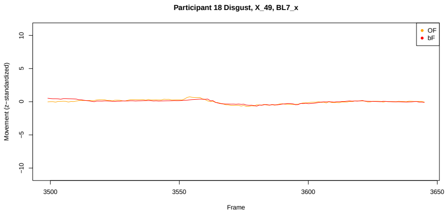

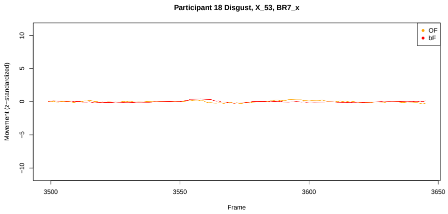

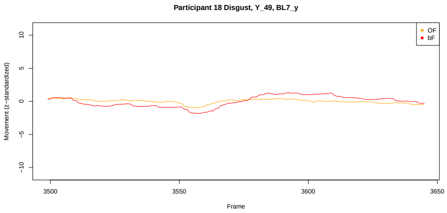

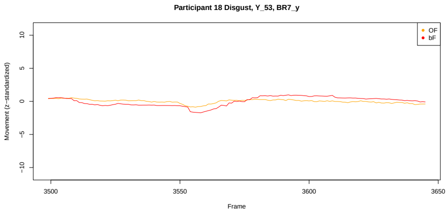

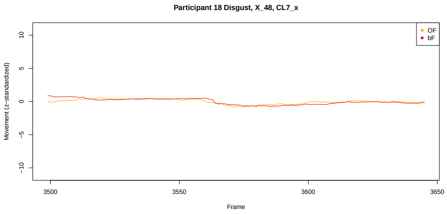

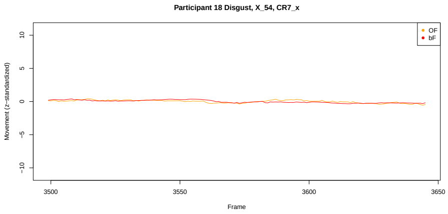

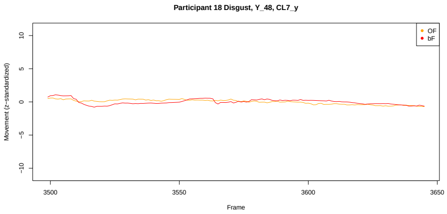

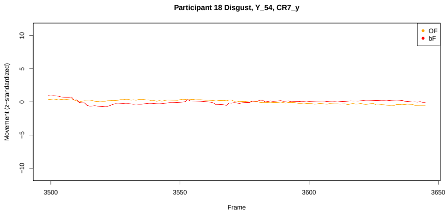

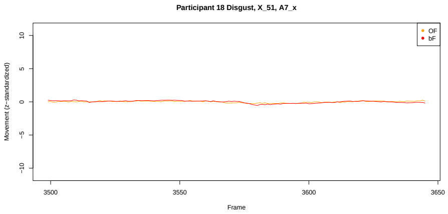

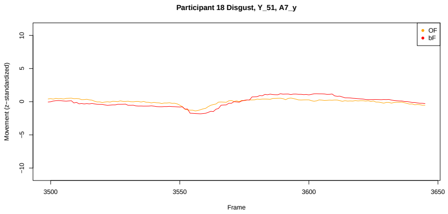

Subject 18, Happiness

Participant 18 Happiness, X_21, BL2_x

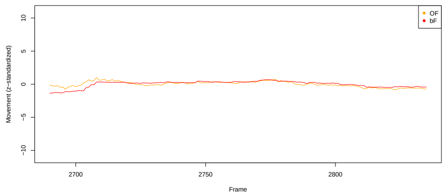

Participant 18 Happiness, X_22, BR2_x

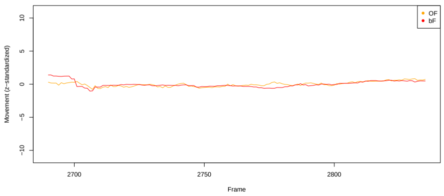

Participant 18 Happiness, Y_21, BL2_y

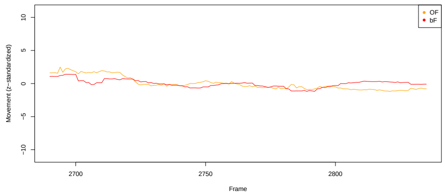

Participant 18 Happiness, Y_22, BR2_y

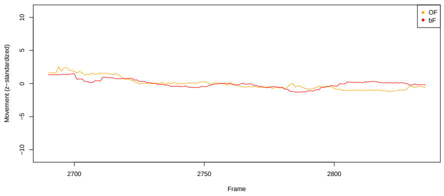

Participant 18 Happiness, X_49, BL7_x

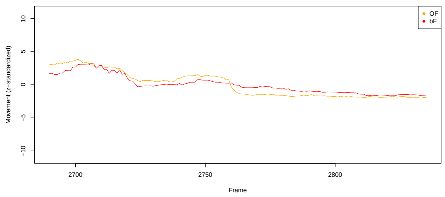

Participant 18 Happiness, X_53, BR7_x

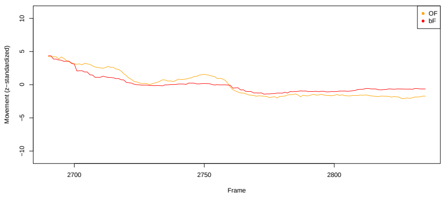

Participant 18 Happiness, Y_49, BL7_y

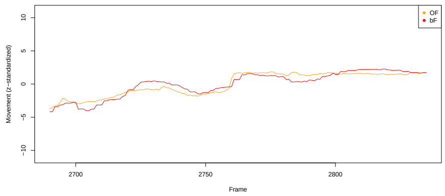

Participant 18 Happiness, Y_53, BR7_y

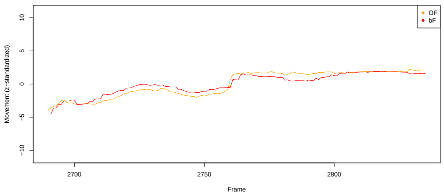

Participant 18 Happiness, X_48, CL7_x

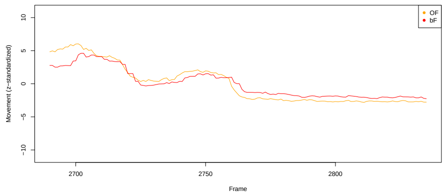

Participant 18 Happiness, X_54, CR7_x

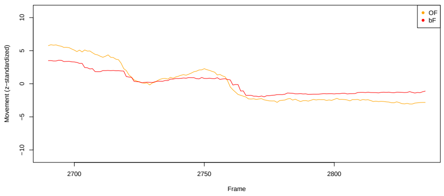

Participant 18 Happiness, Y_48, CL7_y

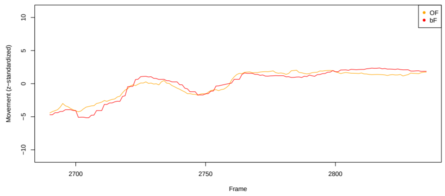

Participant 18 Happiness, Y_54, CR7_y

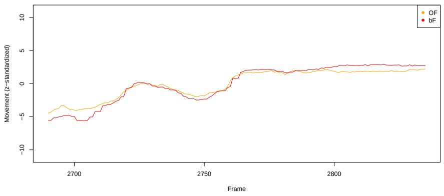

Participant 18 Happiness, X_51, A7_x

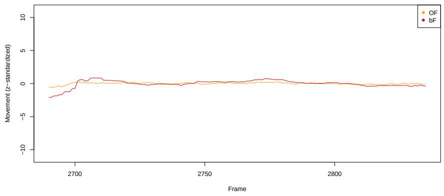

Participant 18 Happiness, Y_51, A7_y

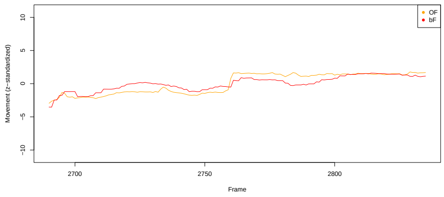

Subject 18, Sadness

Participant 18 Sadness, X_21, BL2_x

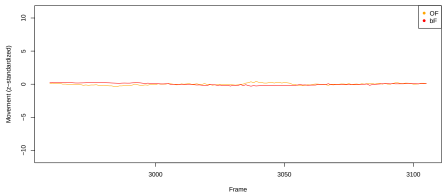

Participant 18 Sadness, X_22, BR2_x

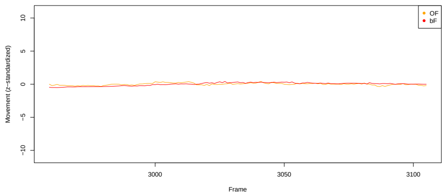

Participant 18 Sadness, Y_21, BL2_y

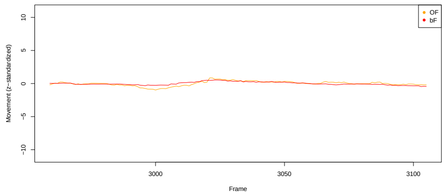

Participant 18 Sadness, Y_22, BR2_y

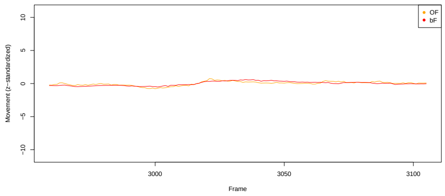

Participant 18 Sadness, X_49, BL7_x

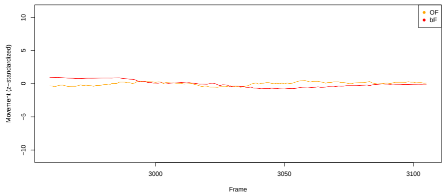

Participant 18 Sadness, X_53, BR7_x

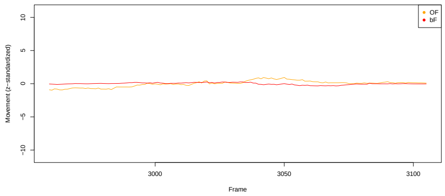

Participant 18 Sadness, Y_49, BL7_y

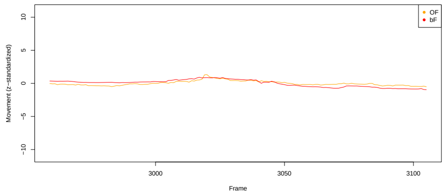

Participant 18 Sadness, Y_53, BR7_y

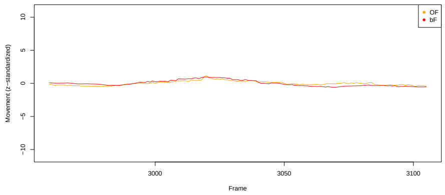

Participant 18 Sadness, X_48, CL7_x

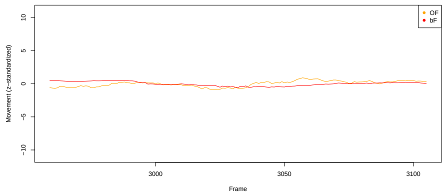

Participant 18 Sadness, X_54, CR7_x

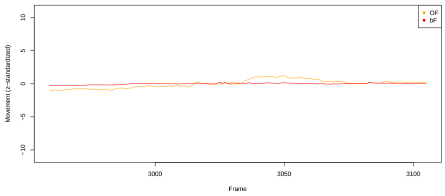

Participant 18 Sadness, Y_48, CL7_y

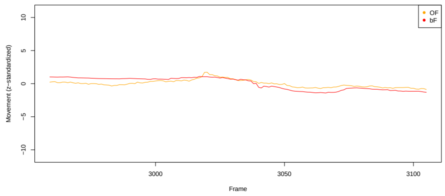

Participant 18 Sadness, Y_54, CR7_y

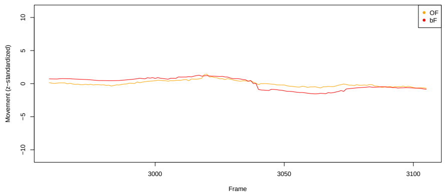

Participant 18 Sadness, X_51, A7_x

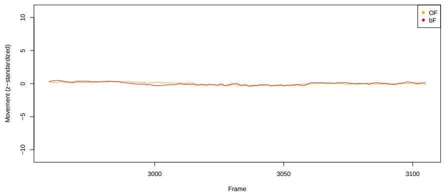

Participant 18 Sadness, Y_51, A7_y

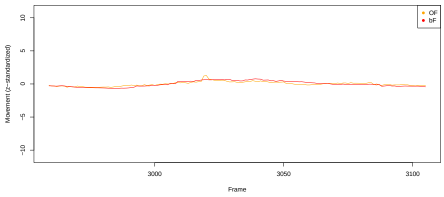

Subject 19, Anger

Participant 19 Anger, X_21, BL2_x

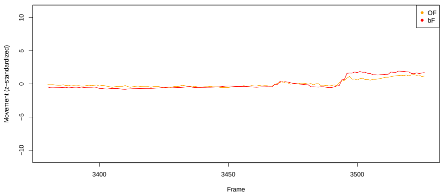

Participant 19 Anger, X_22, BR2_x

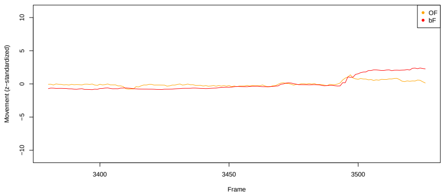

Participant 19 Anger, Y_21, BL2_y

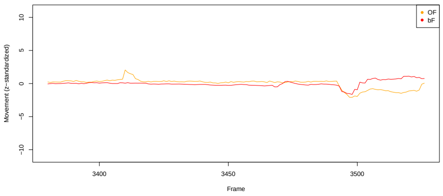

Participant 19 Anger, Y_22, BR2_y

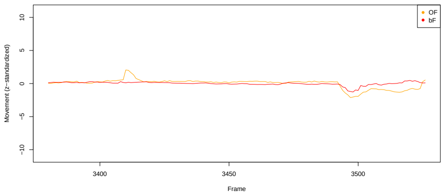

Participant 19 Anger, X_49, BL7_x

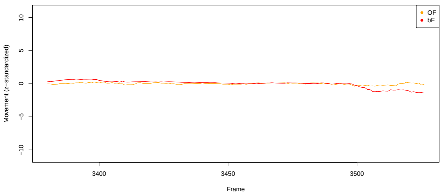

Participant 19 Anger, X_53, BR7_x

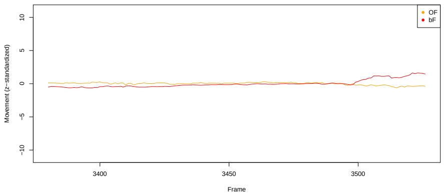

Participant 19 Anger, Y_49, BL7_y

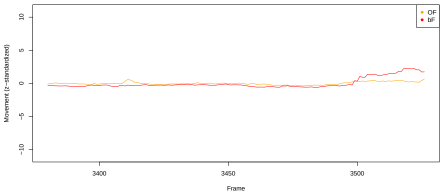

Participant 19 Anger, Y_53, BR7_y

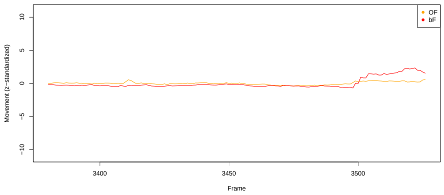

Participant 19 Anger, X_48, CL7_x

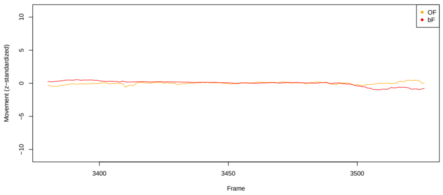

Participant 19 Anger, X_54, CR7_x

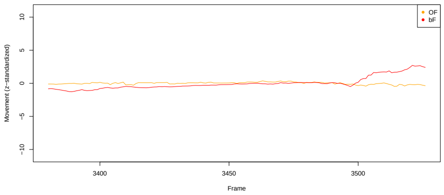

Participant 19 Anger, Y_48, CL7_y

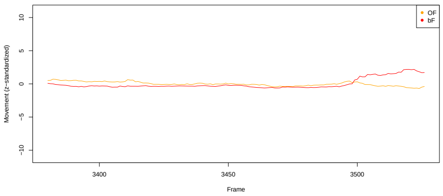

Participant 19 Anger, Y_54, CR7_y

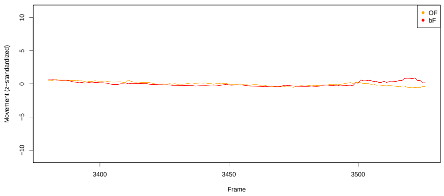

Participant 19 Anger, X_51, A7_x

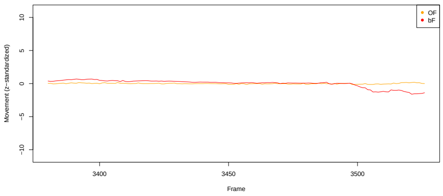

Participant 19 Anger, Y_51, A7_y

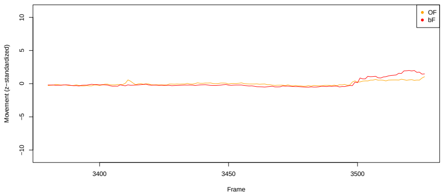

Subject 19, Anxiety

Participant 19 Anxiety, X_21, BL2_x

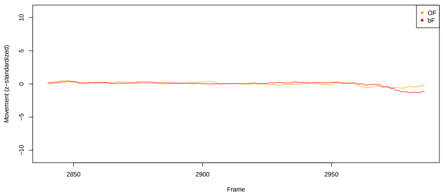

Participant 19 Anxiety, X_22, BR2_x

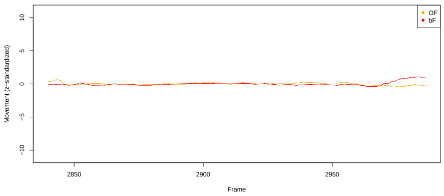

Participant 19 Anxiety, Y_21, BL2_y

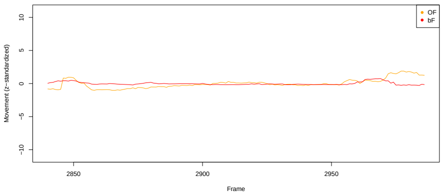

Participant 19 Anxiety, Y_22, BR2_y

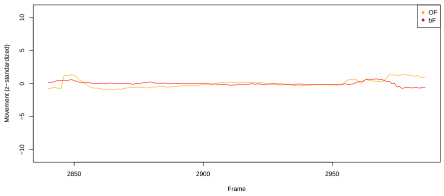

Participant 19 Anxiety, X_49, BL7_x

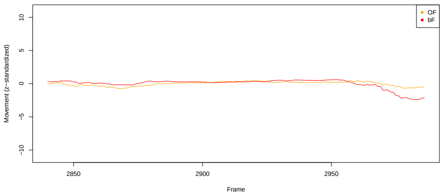

Participant 19 Anxiety, X_53, BR7_x

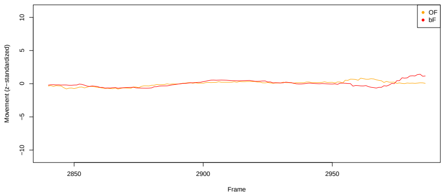

Participant 19 Anxiety, Y_49, BL7_y

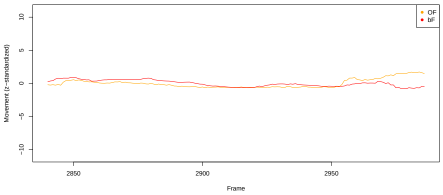

Participant 19 Anxiety, Y_53, BR7_y

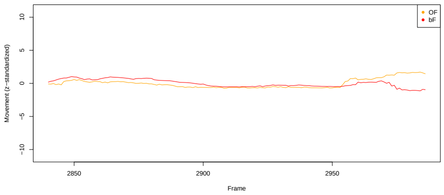

Participant 19 Anxiety, X_48, CL7_x

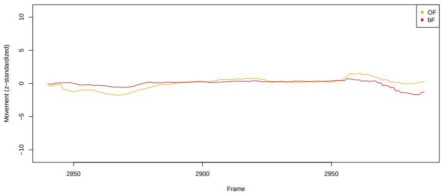

Participant 19 Anxiety, X_54, CR7_x

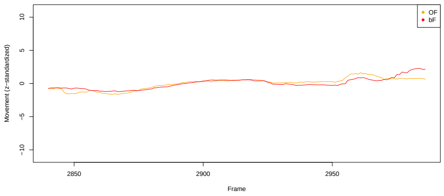

Participant 19 Anxiety, Y_48, CL7_y

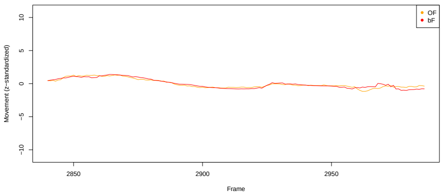

Participant 19 Anxiety, Y_54, CR7_y

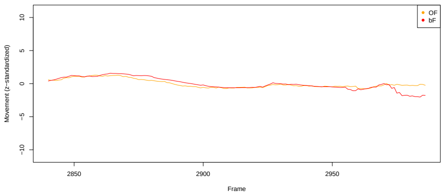

Participant 19 Anxiety, X_51, A7_x

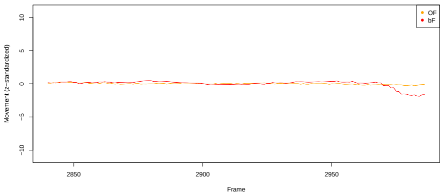

Participant 19 Anxiety, Y_51, A7_y

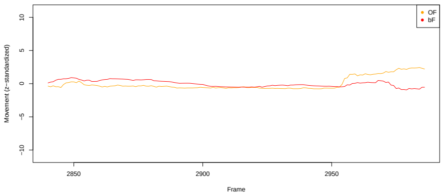

Subject 19, Disgust

Participant 19 Disgust, X_21, BL2_x

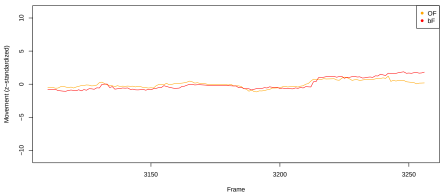

Participant 19 Disgust, X_22, BR2_x

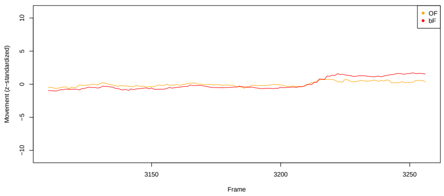

Participant 19 Disgust, Y_21, BL2_y

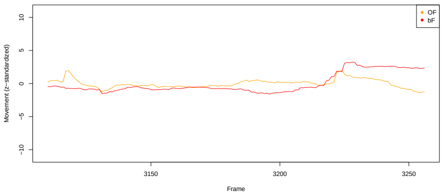

Participant 19 Disgust, Y_22, BR2_y

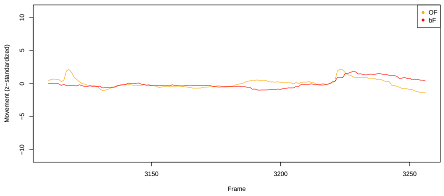

Participant 19 Disgust, X_49, BL7_x

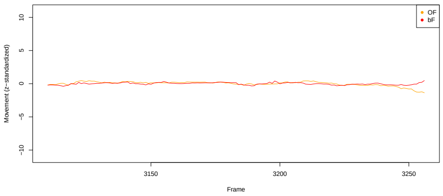

Participant 19 Disgust, X_53, BR7_x

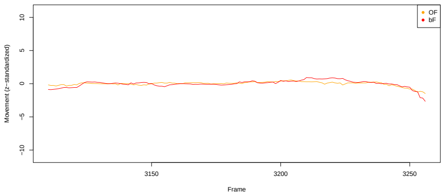

Participant 19 Disgust, Y_49, BL7_y

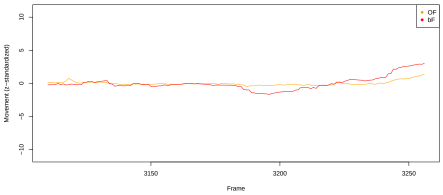

Participant 19 Disgust, Y_53, BR7_y

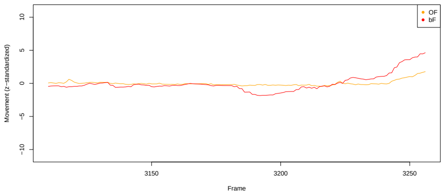

Participant 19 Disgust, X_48, CL7_x

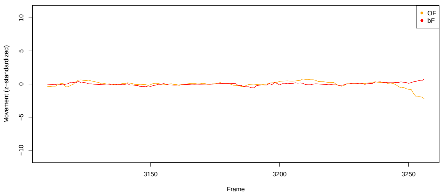

Participant 19 Disgust, X_54, CR7_x

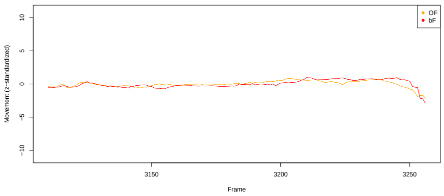

Participant 19 Disgust, Y_48, CL7_y

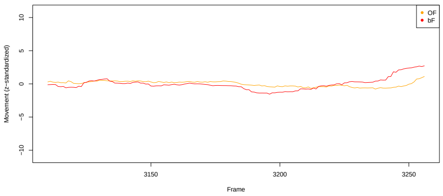

Participant 19 Disgust, Y_54, CR7_y

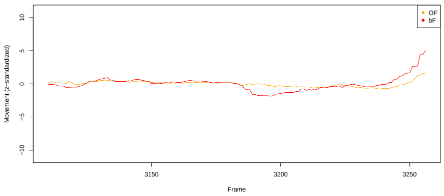

Participant 19 Disgust, X_51, A7_x

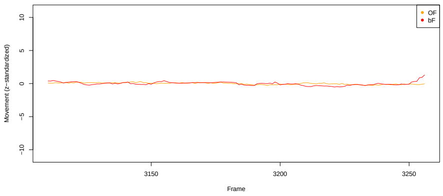

Participant 19 Disgust, Y_51, A7_y

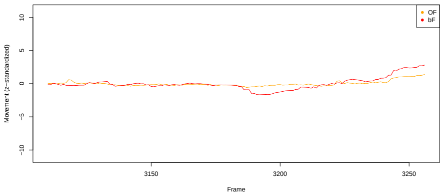

Subject 19, Happiness

Participant 19 Happiness, X_21, BL2_x

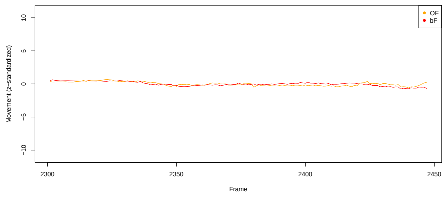

Participant 19 Happiness, X_22, BR2_x

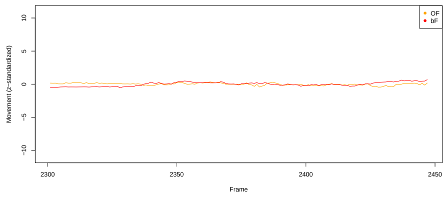

Participant 19 Happiness, Y_21, BL2_y

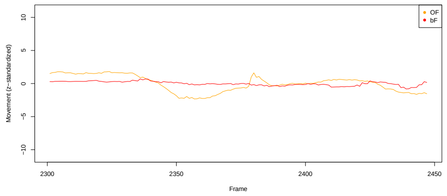

Participant 19 Happiness, Y_22, BR2_y

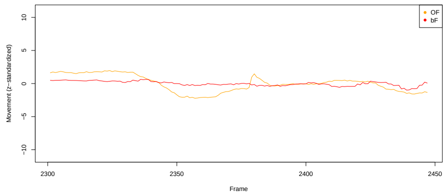

Participant 19 Happiness, X_49, BL7_x

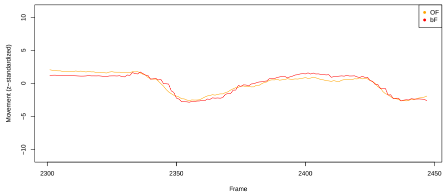

Participant 19 Happiness, X_53, BR7_x

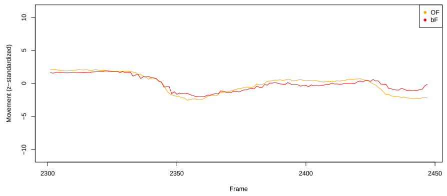

Participant 19 Happiness, Y_49, BL7_y

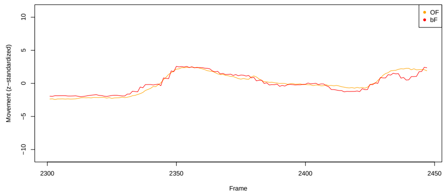

Participant 19 Happiness, Y_53, BR7_y

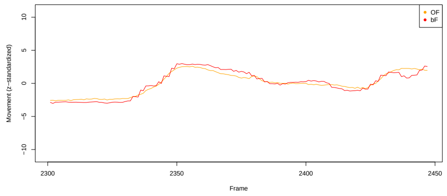

Participant 19 Happiness, X_48, CL7_x

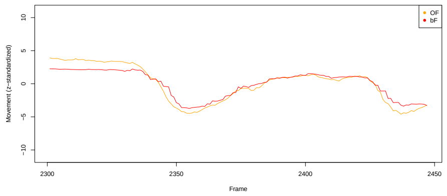

Participant 19 Happiness, X_54, CR7_x

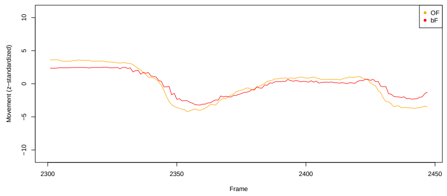

Participant 19 Happiness, Y_48, CL7_y

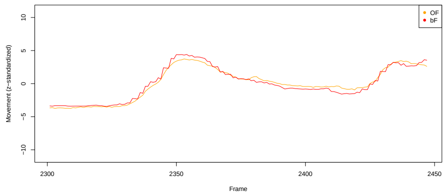

Participant 19 Happiness, Y_54, CR7_y

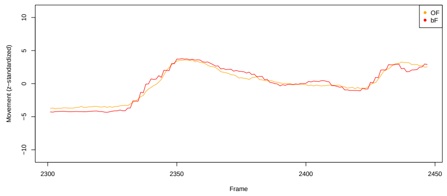

Participant 19 Happiness, X_51, A7_x

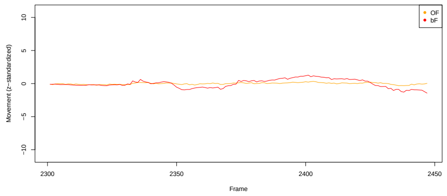

Participant 19 Happiness, Y_51, A7_y

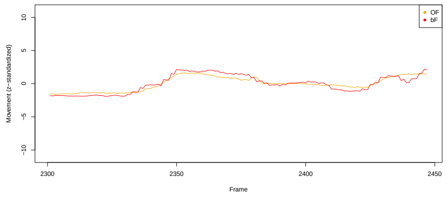

Subject 19, Sadness

Participant 19 Sadness, X_21, BL2_x

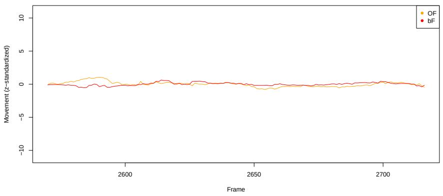

Participant 19 Sadness, X_22, BR2_x

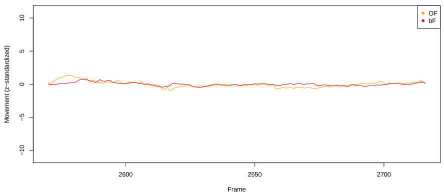

Participant 19 Sadness, Y_21, BL2_y

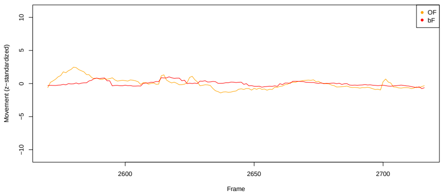

Participant 19 Sadness, Y_22, BR2_y

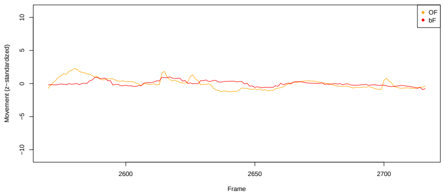

Participant 19 Sadness, X_49, BL7_x

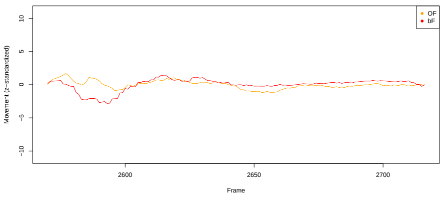

Participant 19 Sadness, X_53, BR7_x

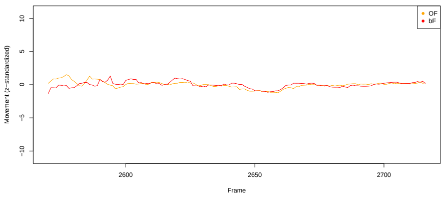

Participant 19 Sadness, Y_49, BL7_y

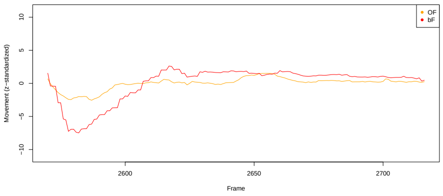

Participant 19 Sadness, Y_53, BR7_y

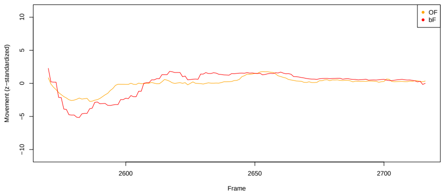

Participant 19 Sadness, X_48, CL7_x

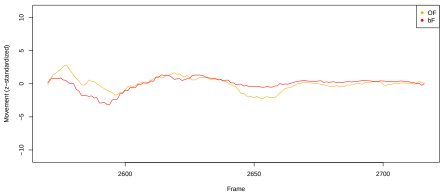

Participant 19 Sadness, X_54, CR7_x

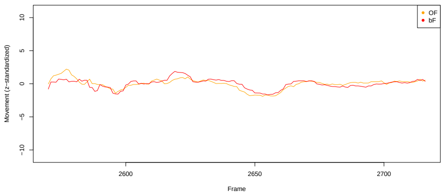

Participant 19 Sadness, Y_48, CL7_y

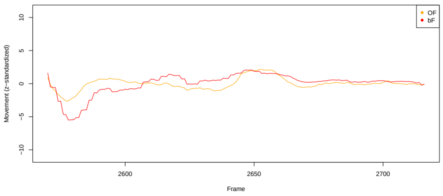

Participant 19 Sadness, Y_54, CR7_y

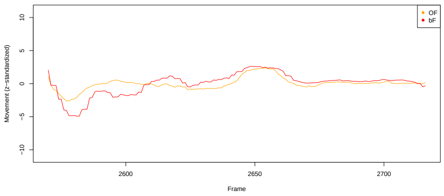

Participant 19 Sadness, X_51, A7_x

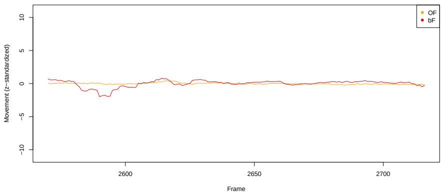

Participant 19 Sadness, Y_51, A7_y

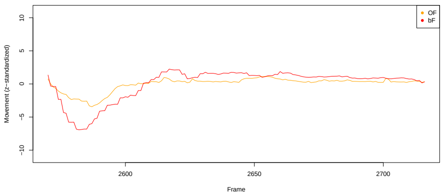

Subject 20, Anger

Participant 20 Anger, X_21, BL2_x

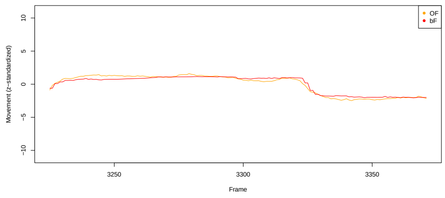

Participant 20 Anger, X_22, BR2_x

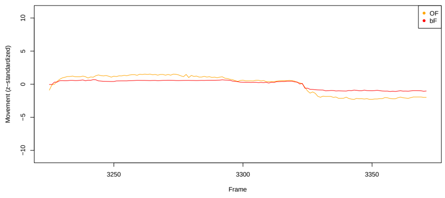

Participant 20 Anger, Y_21, BL2_y

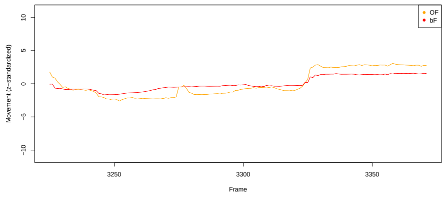

Participant 20 Anger, Y_22, BR2_y

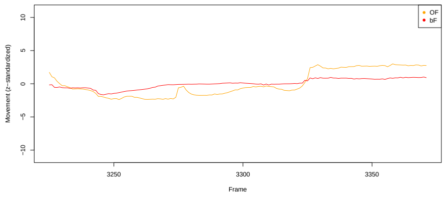

Participant 20 Anger, X_49, BL7_x

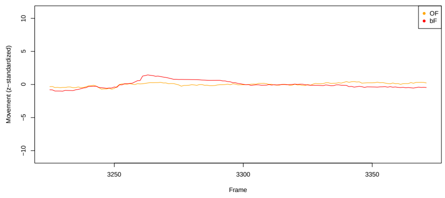

Participant 20 Anger, X_53, BR7_x

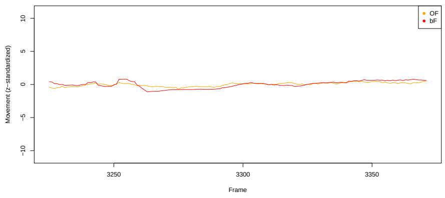

Participant 20 Anger, Y_49, BL7_y

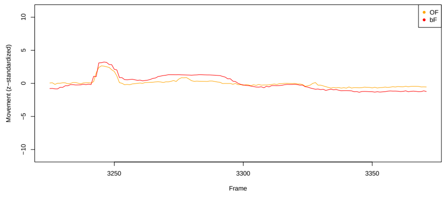

Participant 20 Anger, Y_53, BR7_y

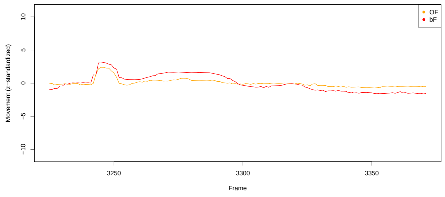

Participant 20 Anger, X_48, CL7_x

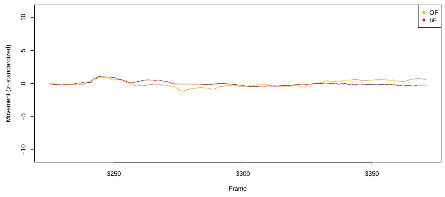

Participant 20 Anger, X_54, CR7_x

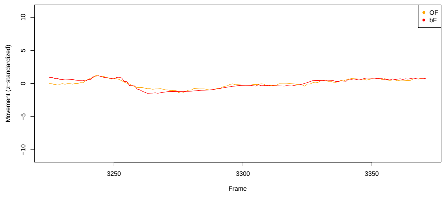

Participant 20 Anger, Y_48, CL7_y

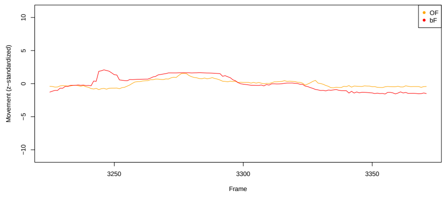

Participant 20 Anger, Y_54, CR7_y

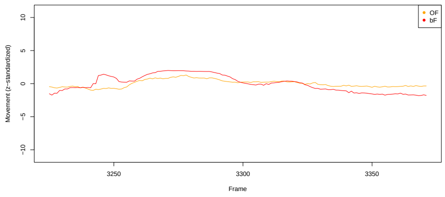

Participant 20 Anger, X_51, A7_x

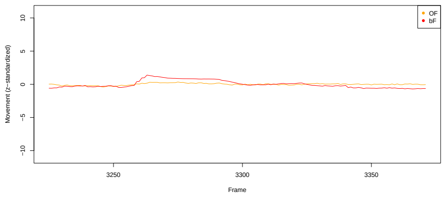

Participant 20 Anger, Y_51, A7_y

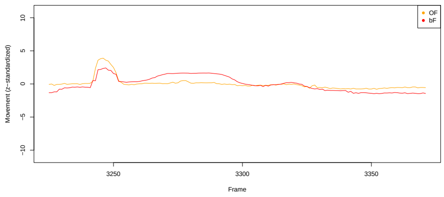

Subject 20, Anxiety

Participant 20 Anxiety, X_21, BL2_x

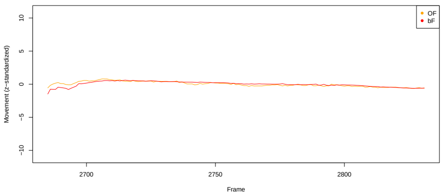

Participant 20 Anxiety, X_22, BR2_x

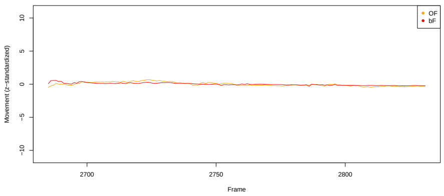

Participant 20 Anxiety, Y_21, BL2_y

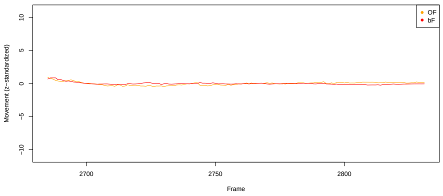

Participant 20 Anxiety, Y_22, BR2_y

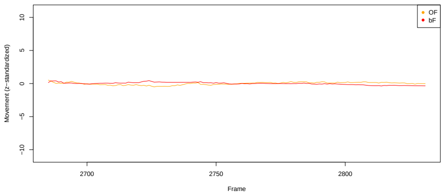

Participant 20 Anxiety, X_49, BL7_x

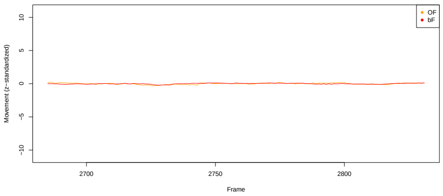

Participant 20 Anxiety, X_53, BR7_x

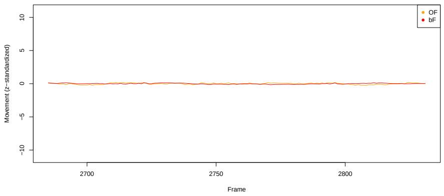

Participant 20 Anxiety, Y_49, BL7_y

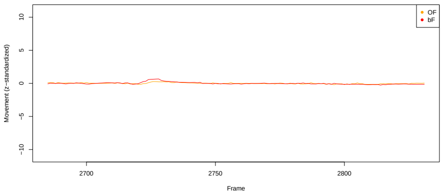

Participant 20 Anxiety, Y_53, BR7_y

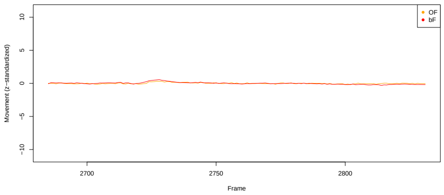

Participant 20 Anxiety, X_48, CL7_x

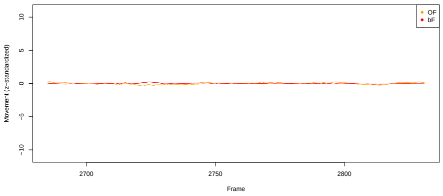

Participant 20 Anxiety, X_54, CR7_x

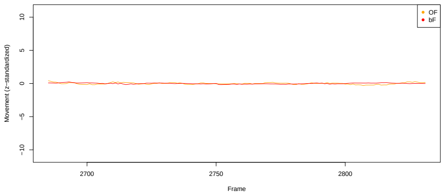

Participant 20 Anxiety, Y_48, CL7_y

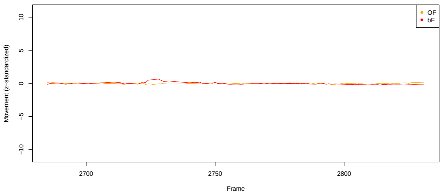

Participant 20 Anxiety, Y_54, CR7_y

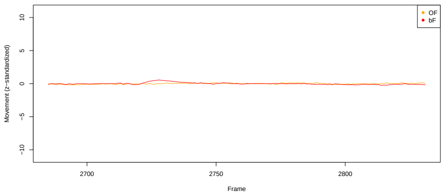

Participant 20 Anxiety, X_51, A7_x

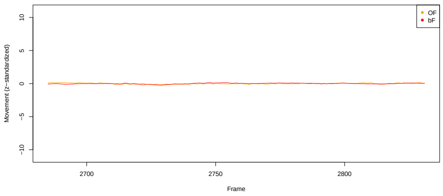

Participant 20 Anxiety, Y_51, A7_y

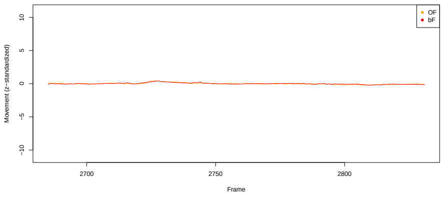

Subject 20, Disgust

Participant 20 Disgust, X_21, BL2_x

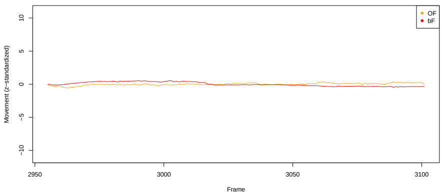

Participant 20 Disgust, X_22, BR2_x

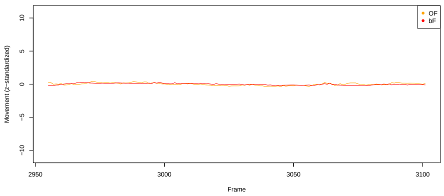

Participant 20 Disgust, Y_21, BL2_y

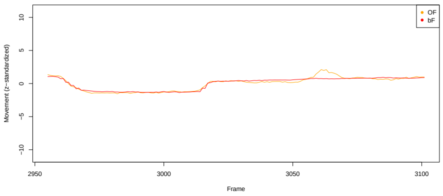

Participant 20 Disgust, Y_22, BR2_y

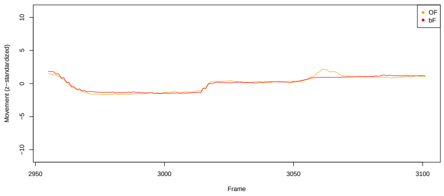

Participant 20 Disgust, X_49, BL7_x

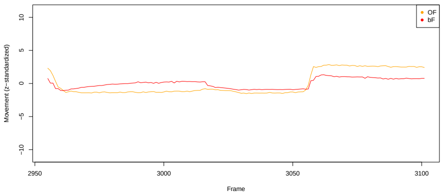

Participant 20 Disgust, X_53, BR7_x

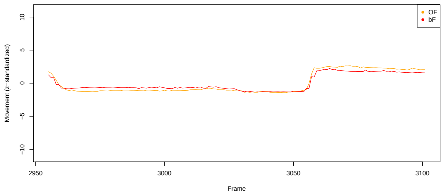

Participant 20 Disgust, Y_49, BL7_y

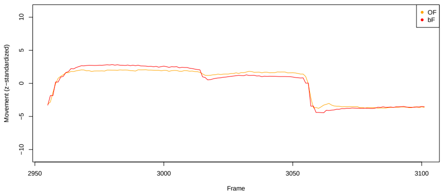

Participant 20 Disgust, Y_53, BR7_y

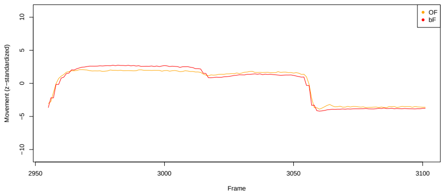

Participant 20 Disgust, X_48, CL7_x

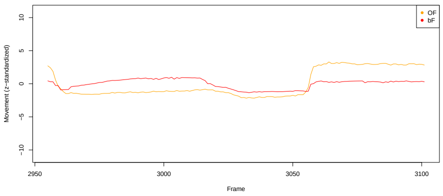

Participant 20 Disgust, X_54, CR7_x

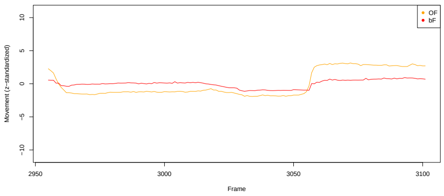

Participant 20 Disgust, Y_48, CL7_y

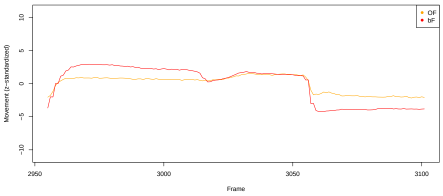

Participant 20 Disgust, Y_54, CR7_y

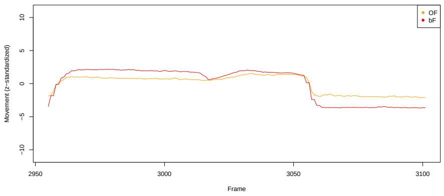

Participant 20 Disgust, X_51, A7_x

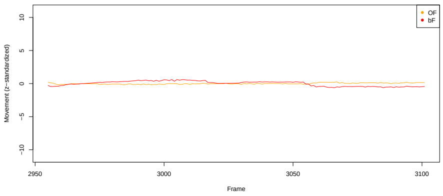

Participant 20 Disgust, Y_51, A7_y

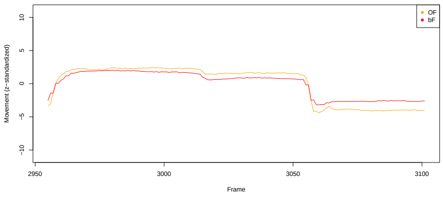

Subject 20, Happiness

Participant 20 Happiness, X_21, BL2_x

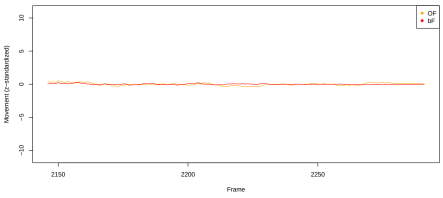

Participant 20 Happiness, X_22, BR2_x

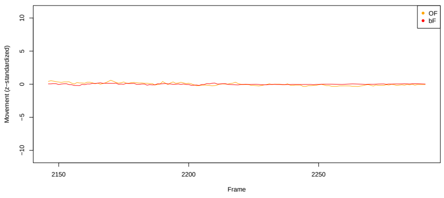

Participant 20 Happiness, Y_21, BL2_y

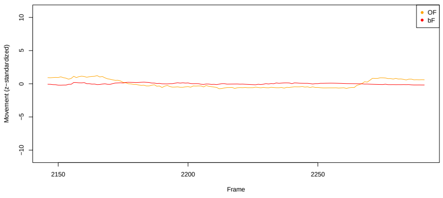

Participant 20 Happiness, Y_22, BR2_y

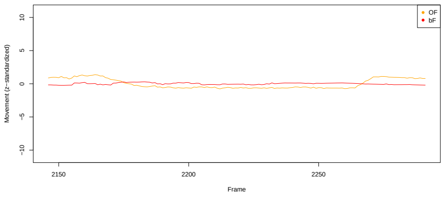

Participant 20 Happiness, X_49, BL7_x

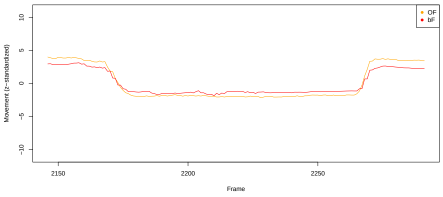

Participant 20 Happiness, X_53, BR7_x

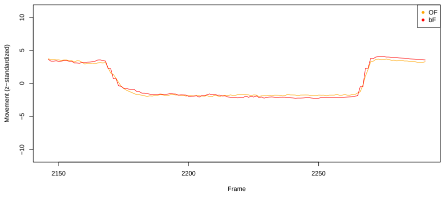

Participant 20 Happiness, Y_49, BL7_y

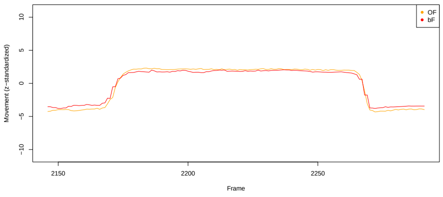

Participant 20 Happiness, Y_53, BR7_y

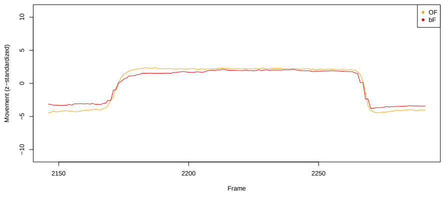

Participant 20 Happiness, X_48, CL7_x

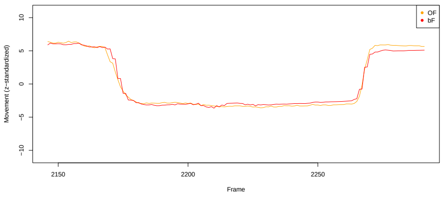

Participant 20 Happiness, X_54, CR7_x

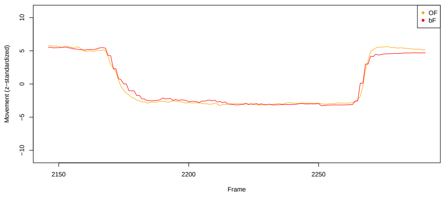

Participant 20 Happiness, Y_48, CL7_y

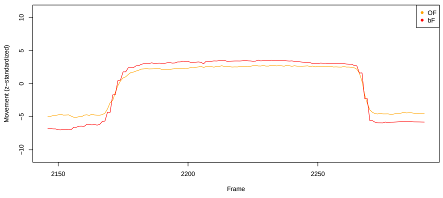

Participant 20 Happiness, Y_54, CR7_y

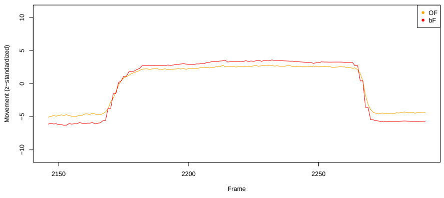

Participant 20 Happiness, X_51, A7_x

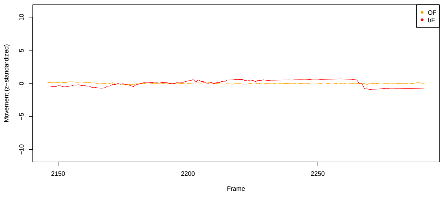

Participant 20 Happiness, Y_51, A7_y

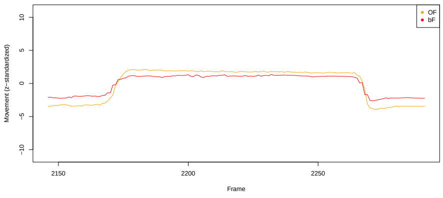

Subject 20, Sadness

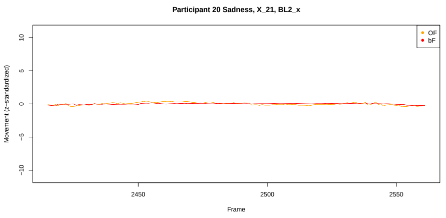

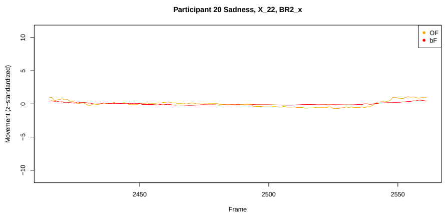

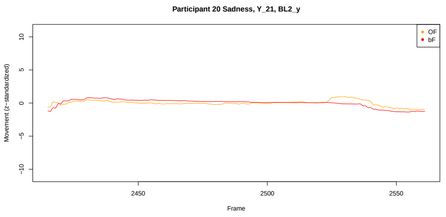

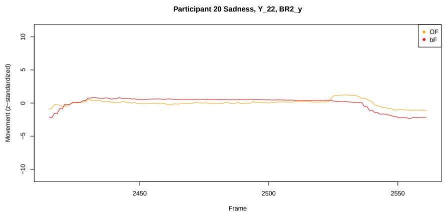

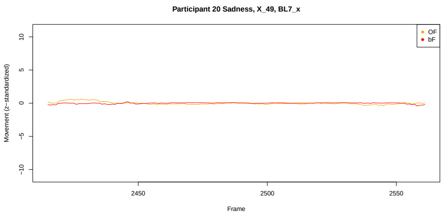

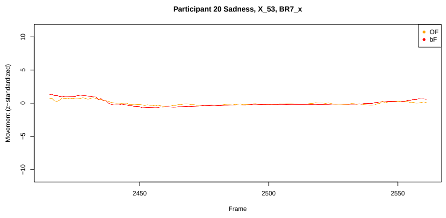

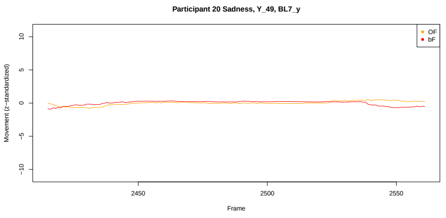

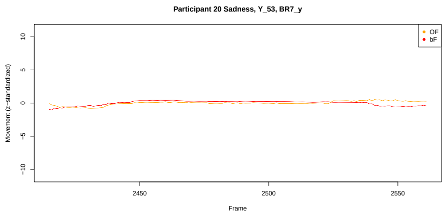

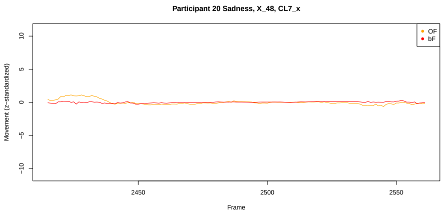

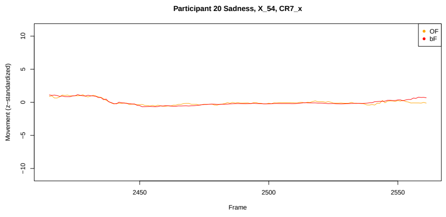

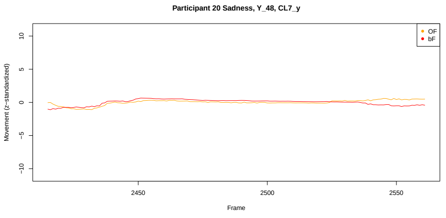

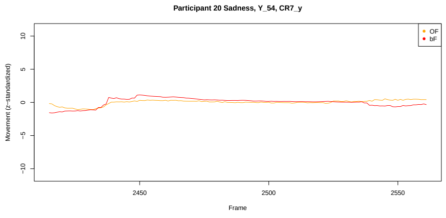

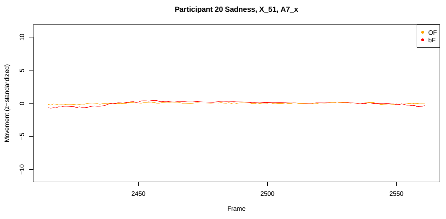

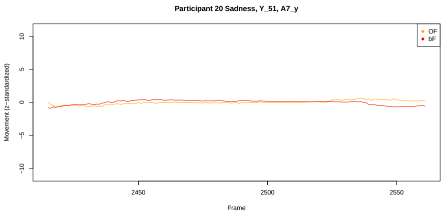

Subject 21, Anger

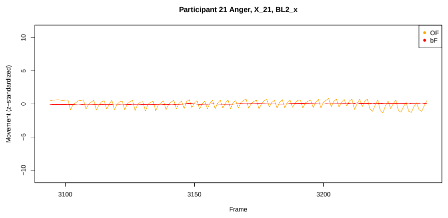

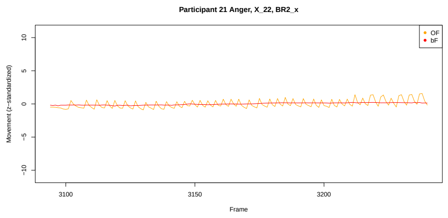

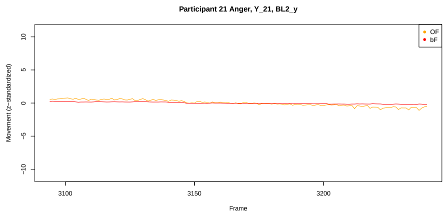

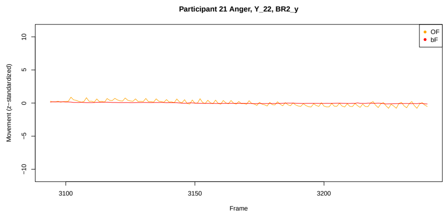

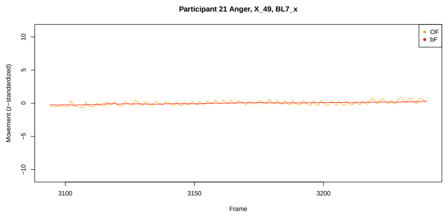

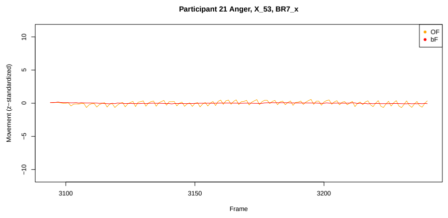

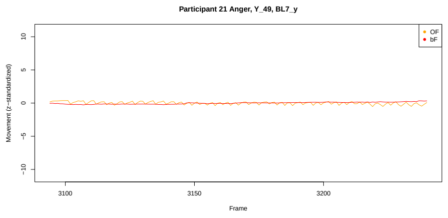

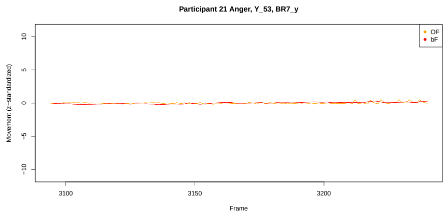

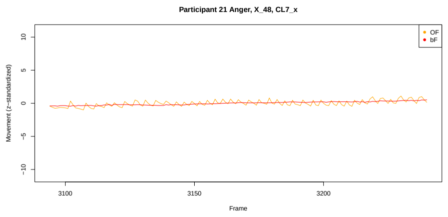

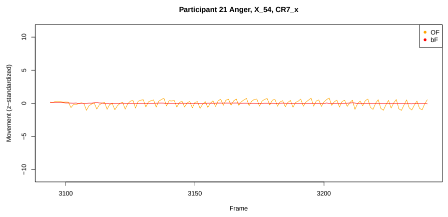

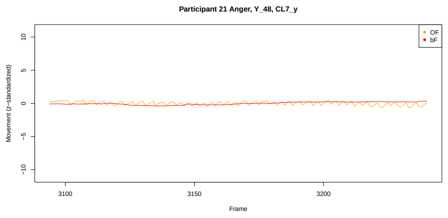

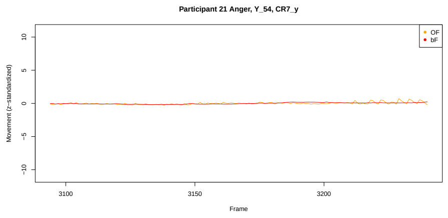

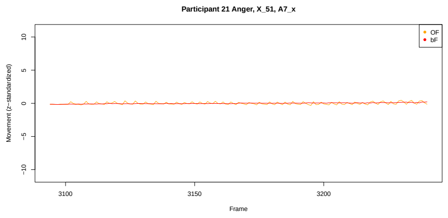

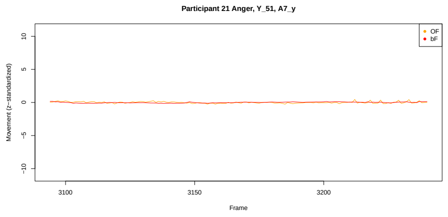

Subject 21, Anxiety

Participant 21 Anxiety, X_21, BL2_x

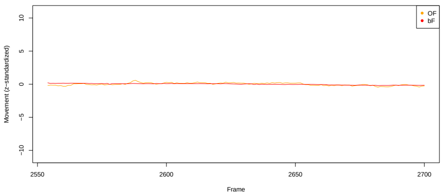

Participant 21 Anxiety, X_22, BR2_x

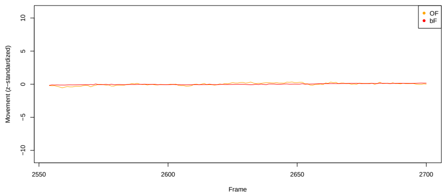

Participant 21 Anxiety, Y_21, BL2_y

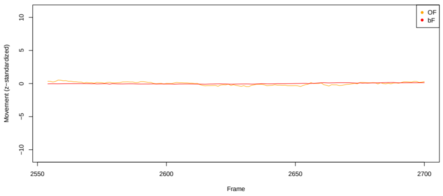

Participant 21 Anxiety, Y_22, BR2_y

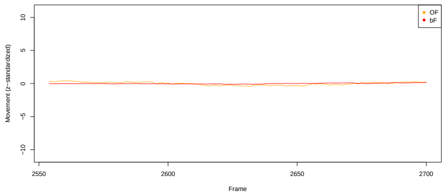

Participant 21 Anxiety, X_49, BL7_x

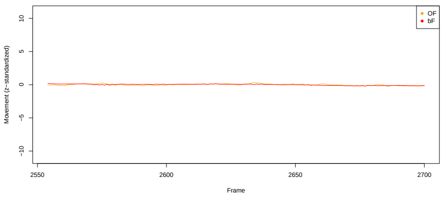

Participant 21 Anxiety, X_53, BR7_x

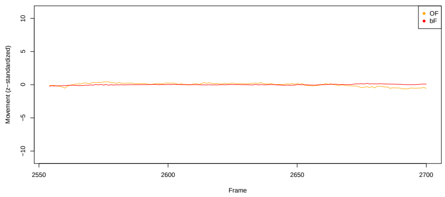

Participant 21 Anxiety, Y_49, BL7_y

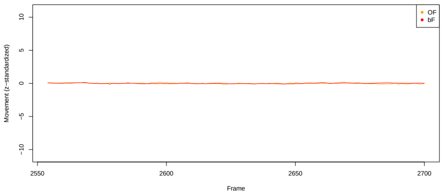

Participant 21 Anxiety, Y_53, BR7_y

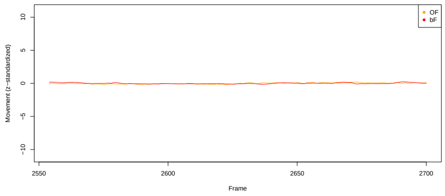

Participant 21 Anxiety, X_48, CL7_x

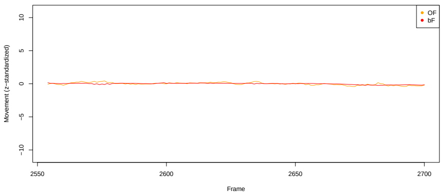

Participant 21 Anxiety, X_54, CR7_x

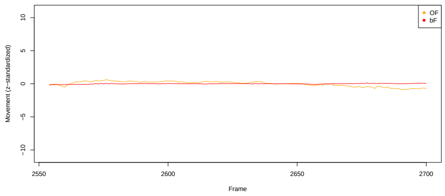

Participant 21 Anxiety, Y_48, CL7_y

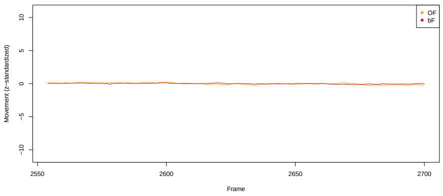

Participant 21 Anxiety, Y_54, CR7_y

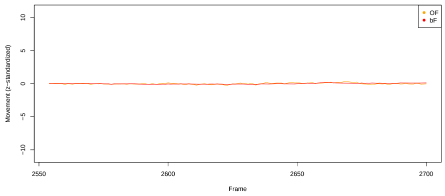

Participant 21 Anxiety, X_51, A7_x

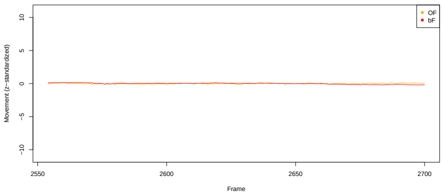

Participant 21 Anxiety, Y_51, A7_y

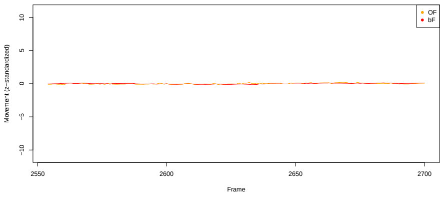

Subject 21, Disgust

Participant 21 Disgust, X_21, BL2_x

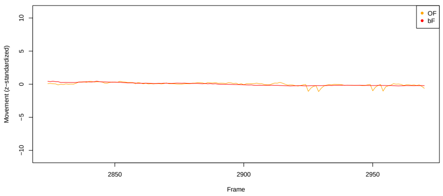

Participant 21 Disgust, X_22, BR2_x

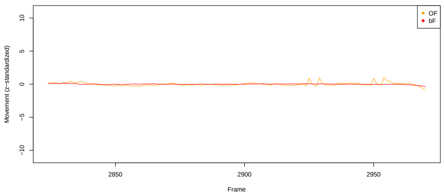

Participant 21 Disgust, Y_21, BL2_y

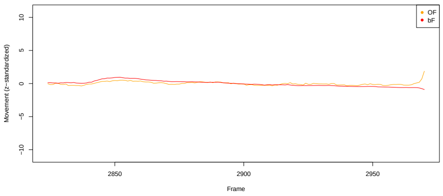

Participant 21 Disgust, Y_22, BR2_y

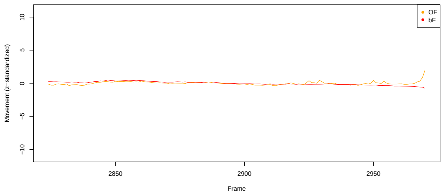

Participant 21 Disgust, X_49, BL7_x

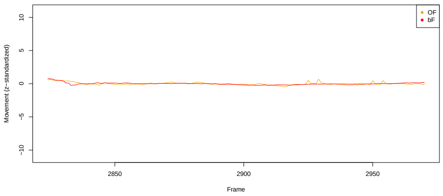

Participant 21 Disgust, X_53, BR7_x

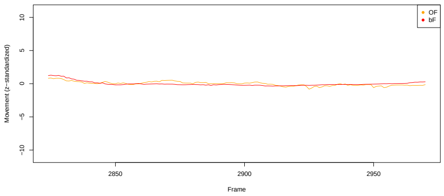

Participant 21 Disgust, Y_49, BL7_y

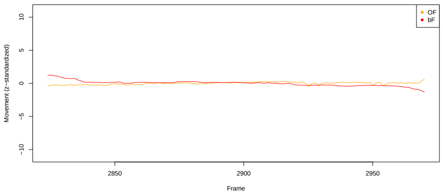

Participant 21 Disgust, Y_53, BR7_y

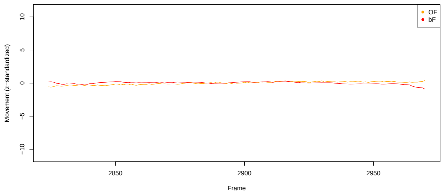

Participant 21 Disgust, X_48, CL7_x

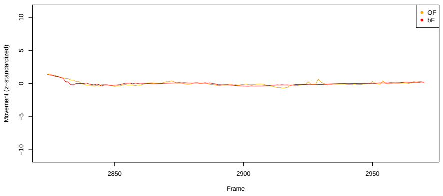

Participant 21 Disgust, X_54, CR7_x

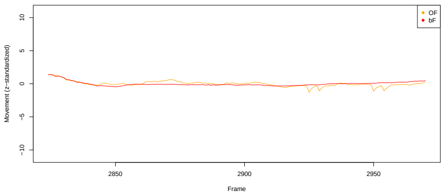

Participant 21 Disgust, Y_48, CL7_y

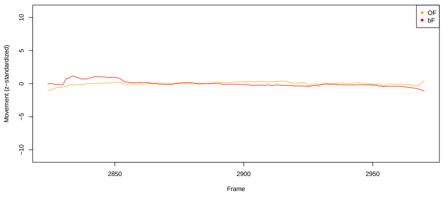

Participant 21 Disgust, Y_54, CR7_y

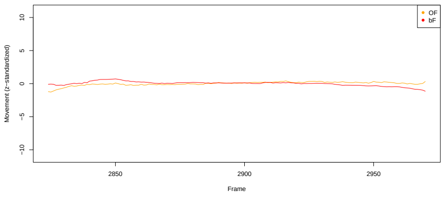

Participant 21 Disgust, X_51, A7_x

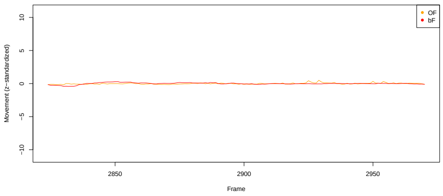

Participant 21 Disgust, Y_51, A7_y

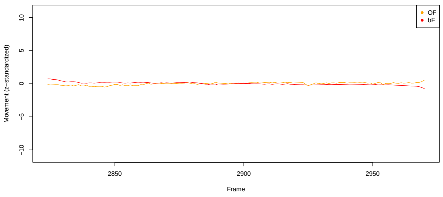

Subject 21, Happiness

Participant 21 Happiness, X_21, BL2_x

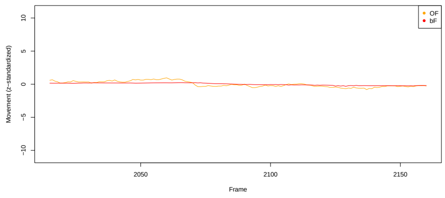

Participant 21 Happiness, X_22, BR2_x

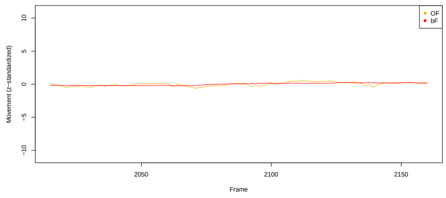

Participant 21 Happiness, Y_21, BL2_y

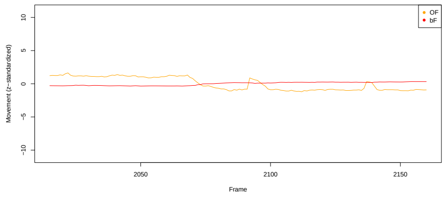

Participant 21 Happiness, Y_22, BR2_y

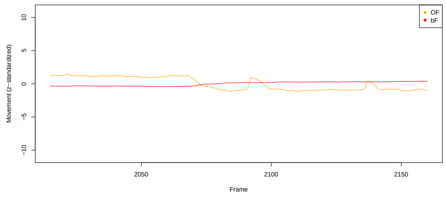

Participant 21 Happiness, X_49, BL7_x

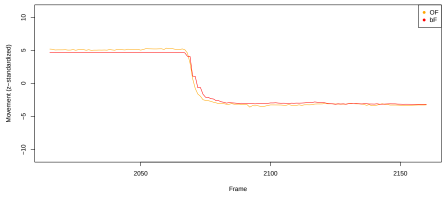

Participant 21 Happiness, X_53, BR7_x

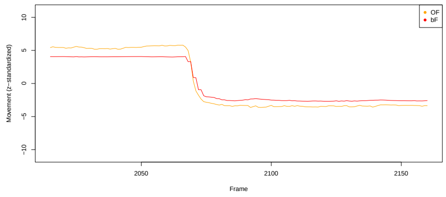

Participant 21 Happiness, Y_49, BL7_y

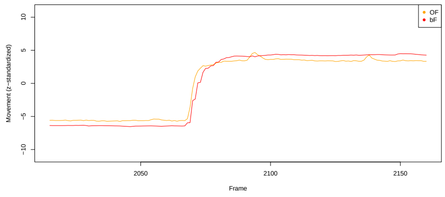

Participant 21 Happiness, Y_53, BR7_y

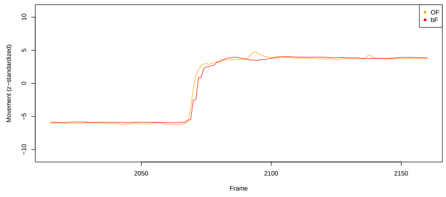

Participant 21 Happiness, X_48, CL7_x

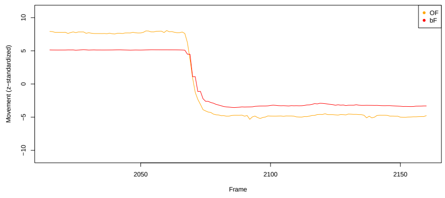

Participant 21 Happiness, X_54, CR7_x

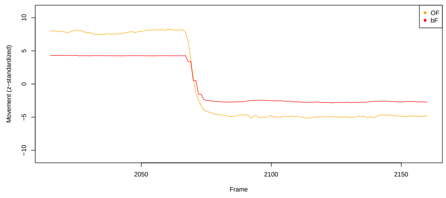

Participant 21 Happiness, Y_48, CL7_y

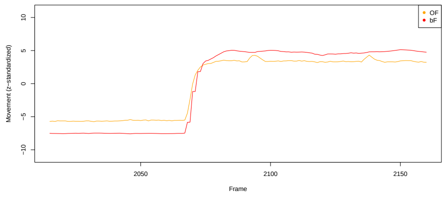

Participant 21 Happiness, Y_54, CR7_y

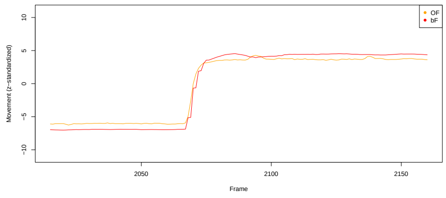

Participant 21 Happiness, X_51, A7_x

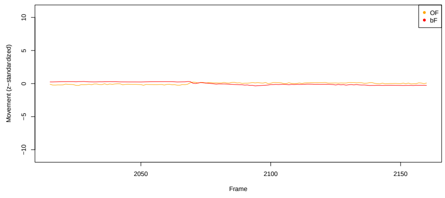

Participant 21 Happiness, Y_51, A7_y

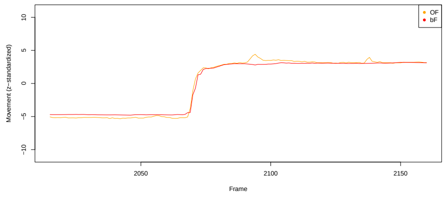

Subject 21, Sadness

Participant 21 Sadness, X_21, BL2_x

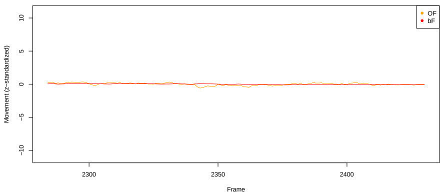

Participant 21 Sadness, X_22, BR2_x

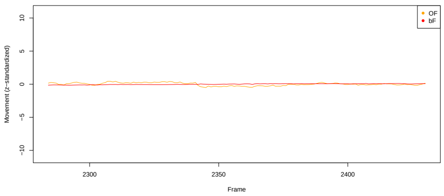

Participant 21 Sadness, Y_21, BL2_y

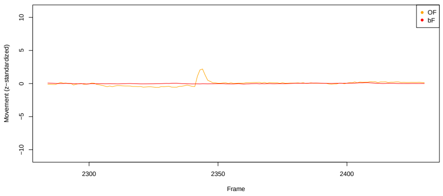

Participant 21 Sadness, Y_22, BR2_y

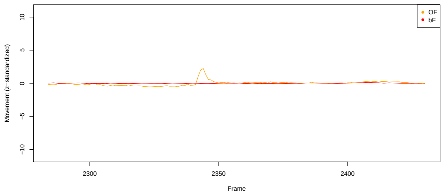

Participant 21 Sadness, X_49, BL7_x

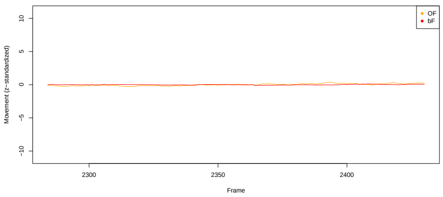

Participant 21 Sadness, X_53, BR7_x

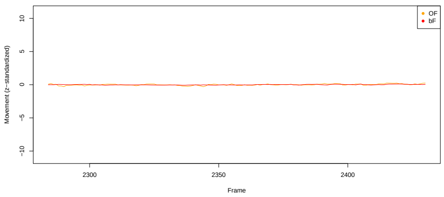

Participant 21 Sadness, Y_49, BL7_y

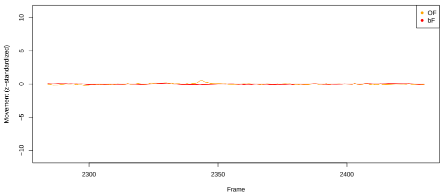

Participant 21 Sadness, Y_53, BR7_y

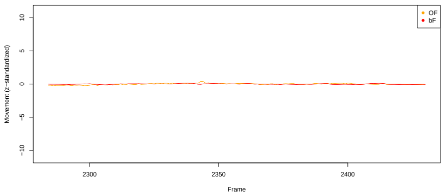

Participant 21 Sadness, X_48, CL7_x

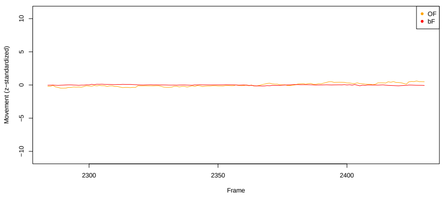

Participant 21 Sadness, X_54, CR7_x

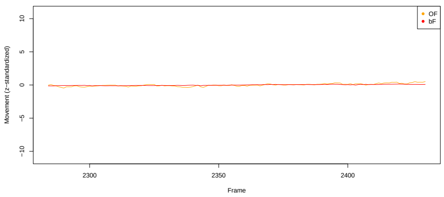

Participant 21 Sadness, Y_48, CL7_y

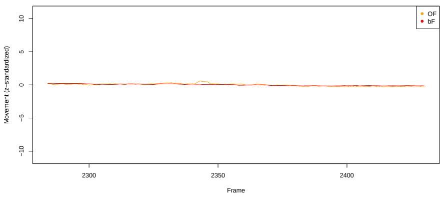

Participant 21 Sadness, Y_54, CR7_y

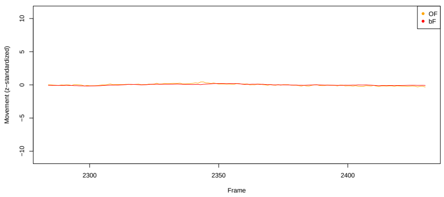

Participant 21 Sadness, X_51, A7_x

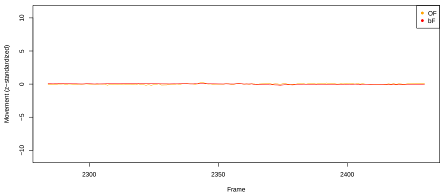

Participant 21 Sadness, Y_51, A7_y

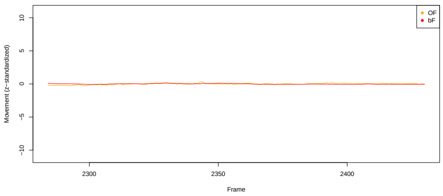

Subject 23, Anger

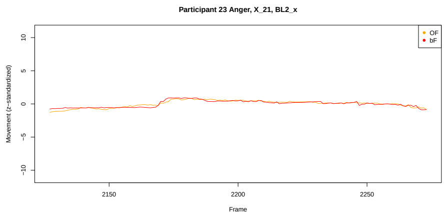

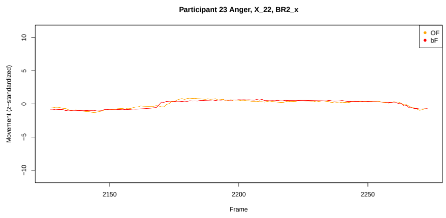

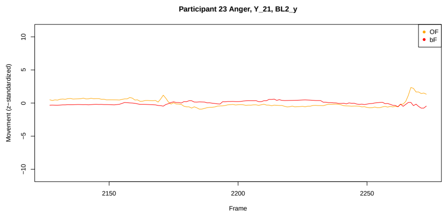

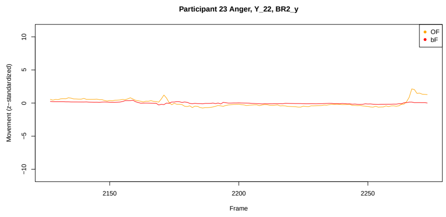

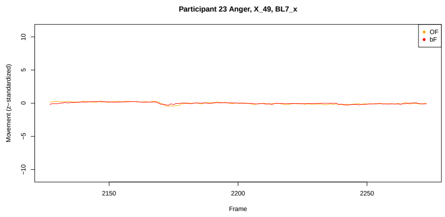

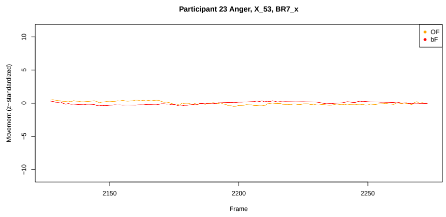

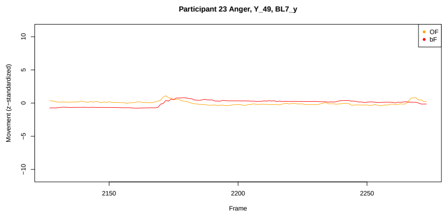

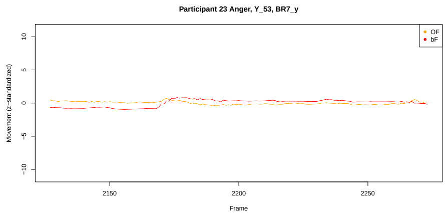

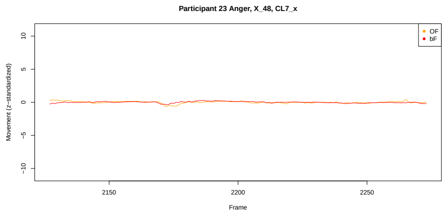

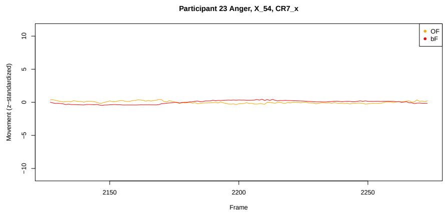

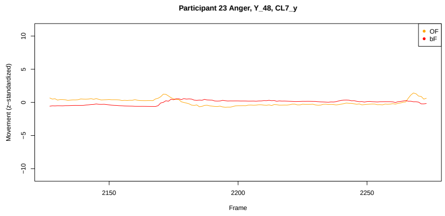

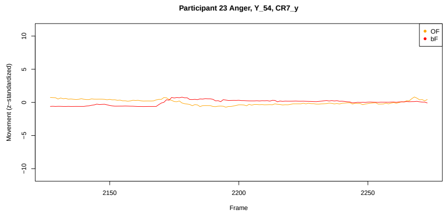

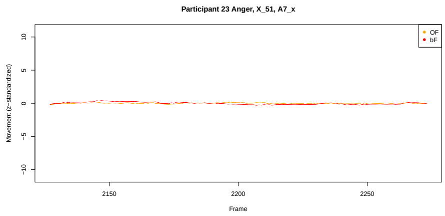

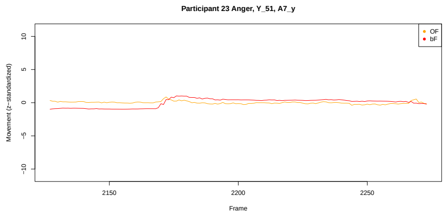

Subject 23, Anxiety

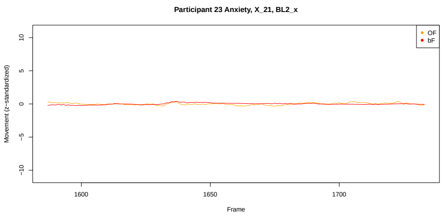

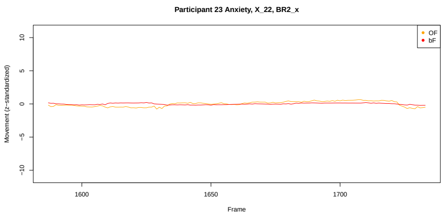

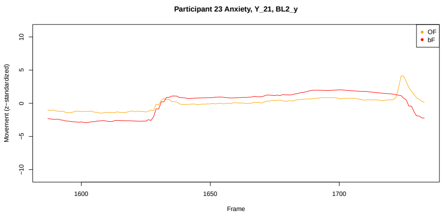

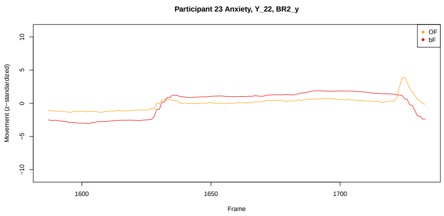

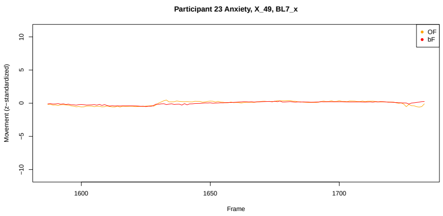

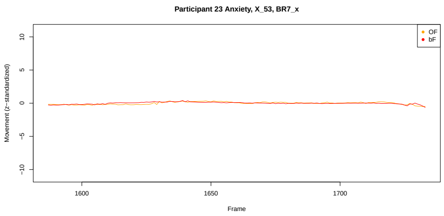

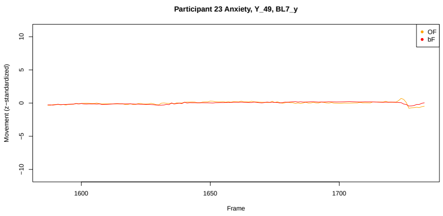

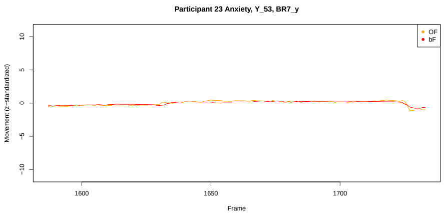

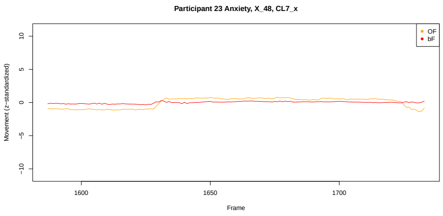

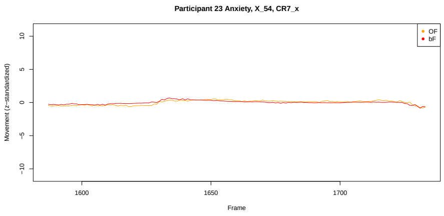

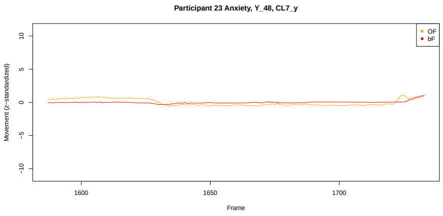

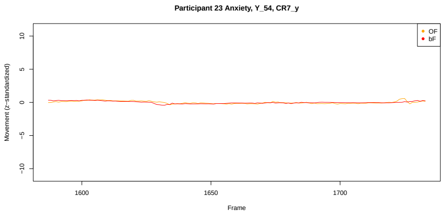

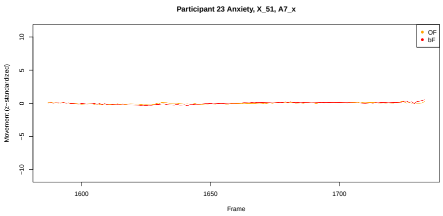

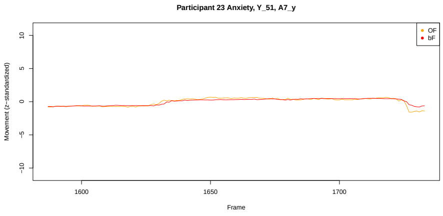

Subject 23, Disgust

Participant 23 Disgust, X_21, BL2_x

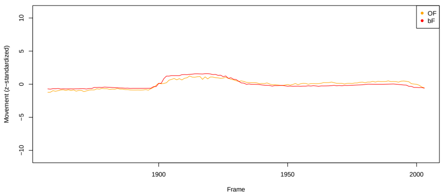

Participant 23 Disgust, X_22, BR2_x

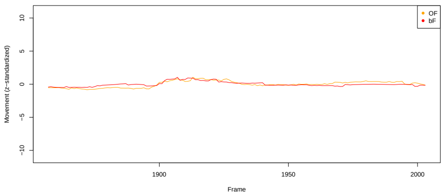

Participant 23 Disgust, Y_21, BL2_y

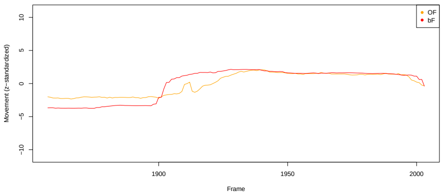

Participant 23 Disgust, Y_22, BR2_y

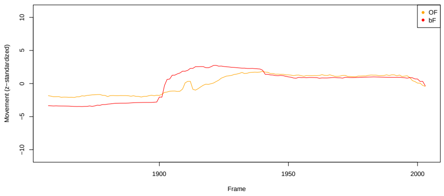

Participant 23 Disgust, X_49, BL7_x

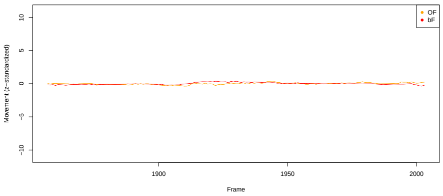

Participant 23 Disgust, X_53, BR7_x

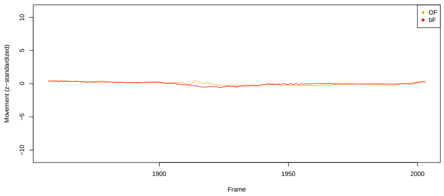

Participant 23 Disgust, Y_49, BL7_y

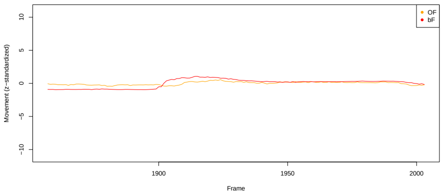

Participant 23 Disgust, Y_53, BR7_y

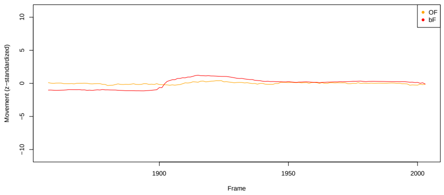

Participant 23 Disgust, X_48, CL7_x

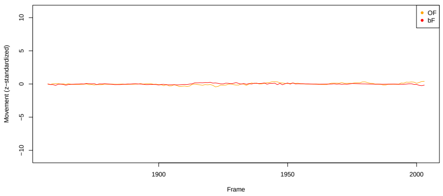

Participant 23 Disgust, X_54, CR7_x

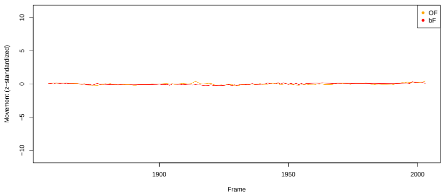

Participant 23 Disgust, Y_48, CL7_y

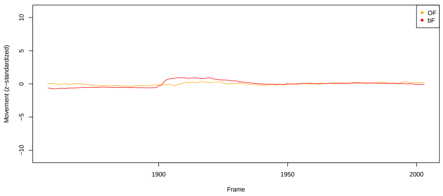

Participant 23 Disgust, Y_54, CR7_y

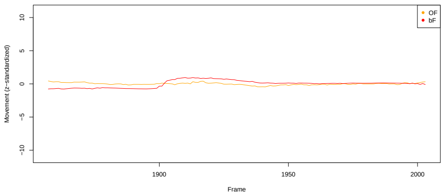

Participant 23 Disgust, X_51, A7_x

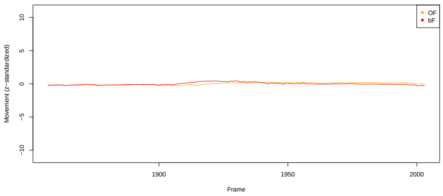

Participant 23 Disgust, Y_51, A7_y

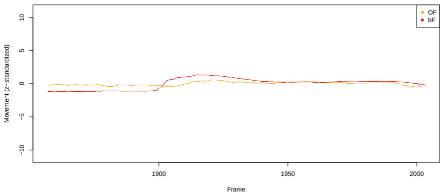

Subject 23, Happiness

Participant 23 Happiness, X_21, BL2_x

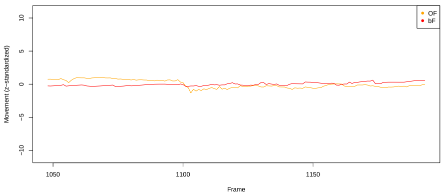

Participant 23 Happiness, X_22, BR2_x

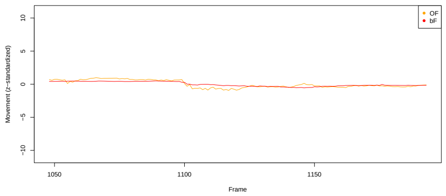

Participant 23 Happiness, Y_21, BL2_y

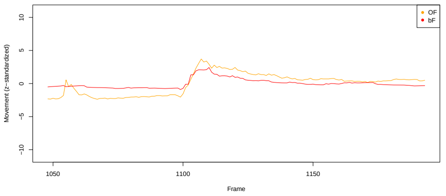

Participant 23 Happiness, Y_22, BR2_y

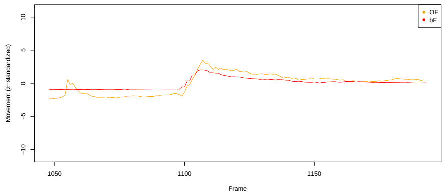

Participant 23 Happiness, X_49, BL7_x

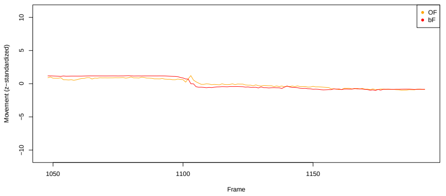

Participant 23 Happiness, X_53, BR7_x

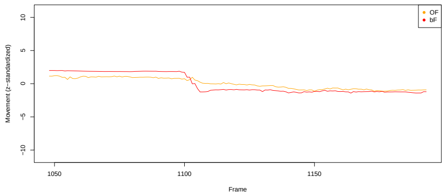

Participant 23 Happiness, Y_49, BL7_y

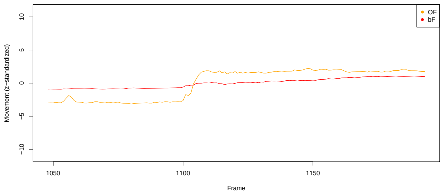

Participant 23 Happiness, Y_53, BR7_y

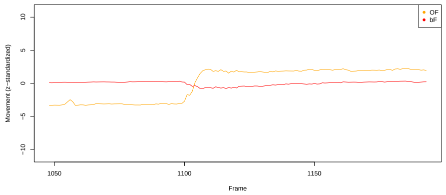

Participant 23 Happiness, X_48, CL7_x

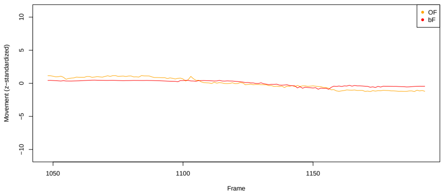

Participant 23 Happiness, X_54, CR7_x

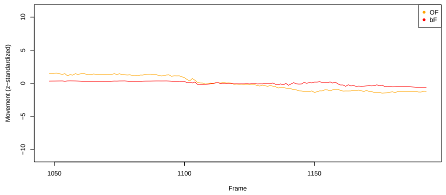

Participant 23 Happiness, Y_48, CL7_y

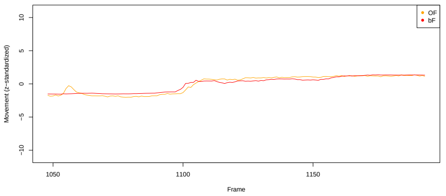

Participant 23 Happiness, Y_54, CR7_y

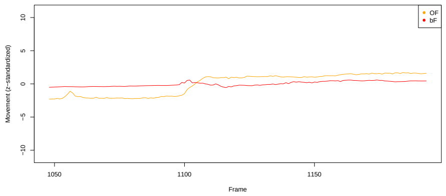

Participant 23 Happiness, X_51, A7_x

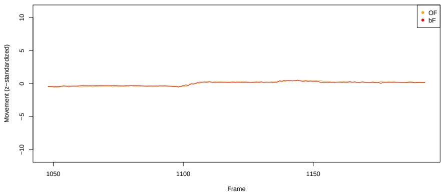

Participant 23 Happiness, Y_51, A7_y

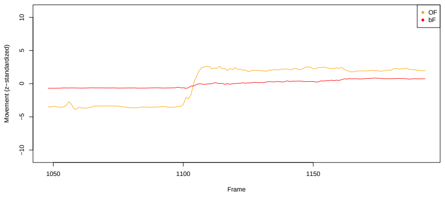

Subject 23, Sadness

Participant 23 Sadness, X_21, BL2_x

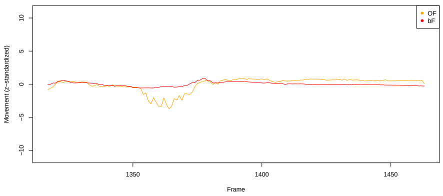

Participant 23 Sadness, X_22, BR2_x

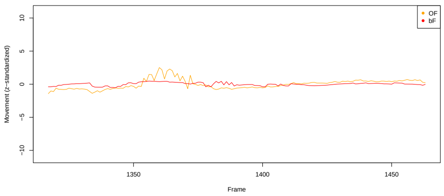

Participant 23 Sadness, Y_21, BL2_y

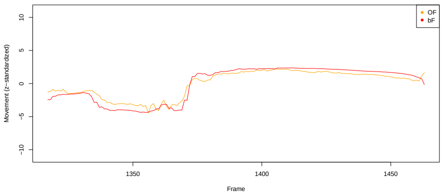

Participant 23 Sadness, Y_22, BR2_y

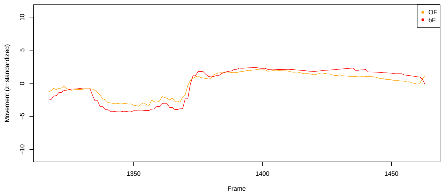

Participant 23 Sadness, X_49, BL7_x

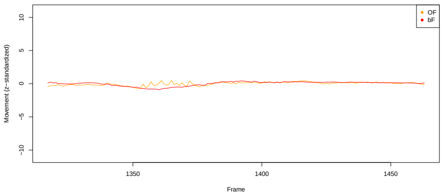

Participant 23 Sadness, X_53, BR7_x

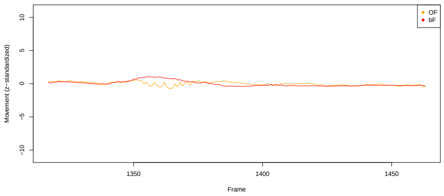

Participant 23 Sadness, Y_49, BL7_y

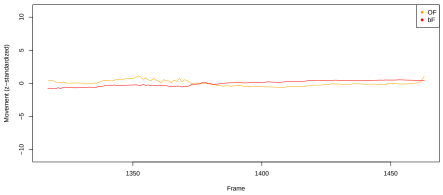

Participant 23 Sadness, Y_53, BR7_y

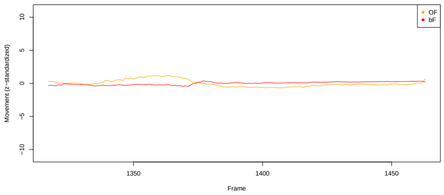

Participant 23 Sadness, X_48, CL7_x

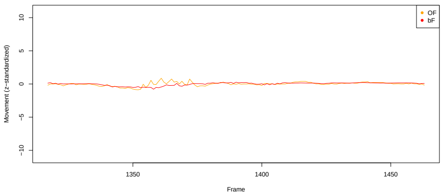

Participant 23 Sadness, X_54, CR7_x

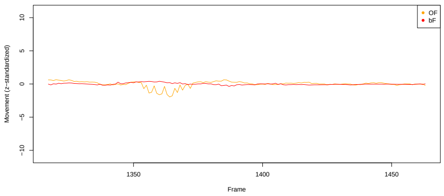

Participant 23 Sadness, Y_48, CL7_y

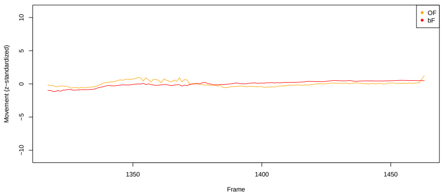

Participant 23 Sadness, Y_54, CR7_y

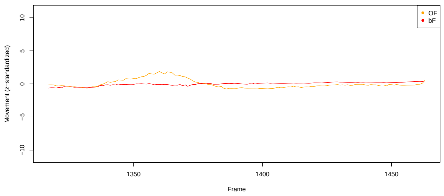

Participant 23 Sadness, X_51, A7_x

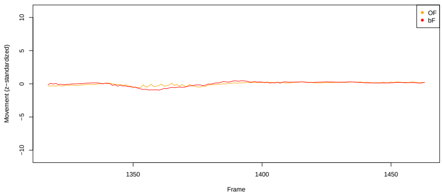

Participant 23 Sadness, Y_51, A7_y

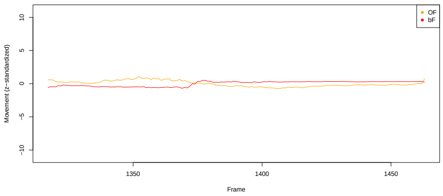

Subject 24, Anger

Participant 24 Anger, X_21, BL2_x

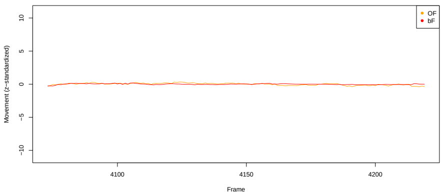

Participant 24 Anger, X_22, BR2_x

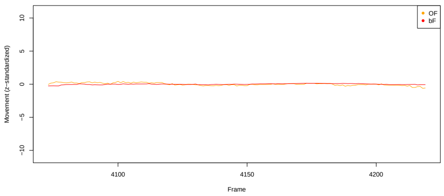

Participant 24 Anger, Y_21, BL2_y

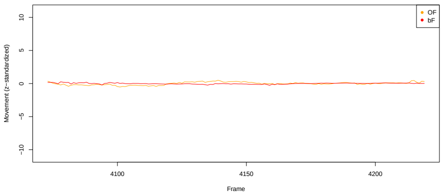

Participant 24 Anger, Y_22, BR2_y

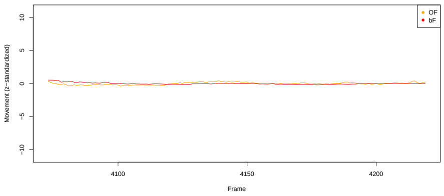

Participant 24 Anger, X_49, BL7_x

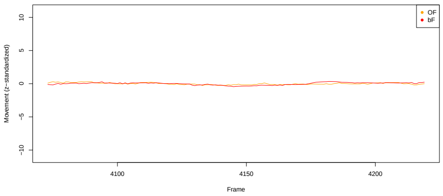

Participant 24 Anger, X_53, BR7_x

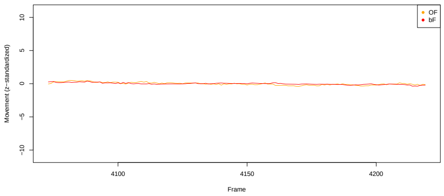

Participant 24 Anger, Y_49, BL7_y

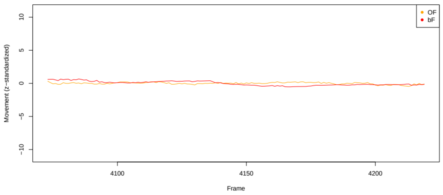

Participant 24 Anger, Y_53, BR7_y

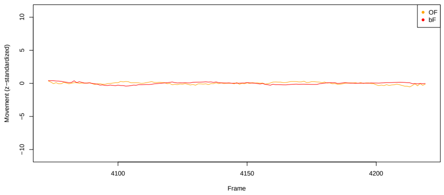

Participant 24 Anger, X_48, CL7_x

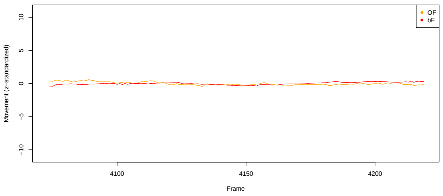

Participant 24 Anger, X_54, CR7_x

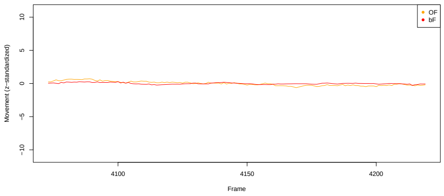

Participant 24 Anger, Y_48, CL7_y

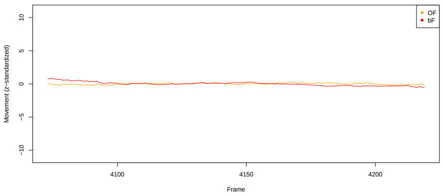

Participant 24 Anger, Y_54, CR7_y

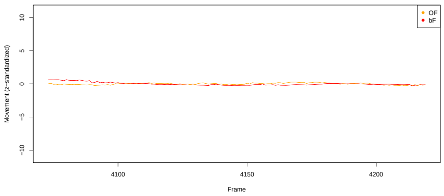

Participant 24 Anger, X_51, A7_x

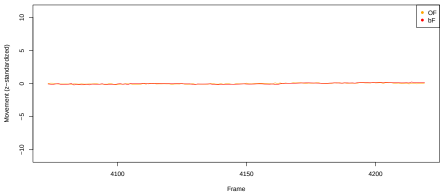

Participant 24 Anger, Y_51, A7_y

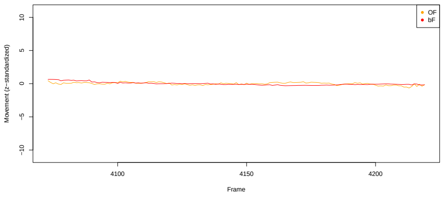

Subject 24, Anxiety

Participant 24 Anxiety, X_21, BL2_x

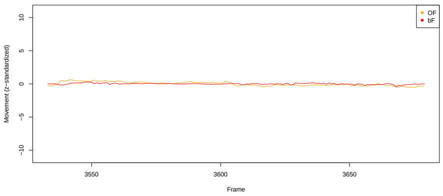

Participant 24 Anxiety, X_22, BR2_x

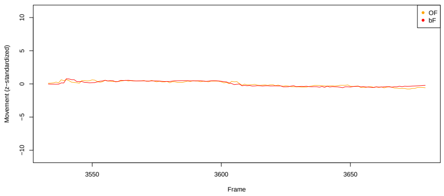

Participant 24 Anxiety, Y_21, BL2_y

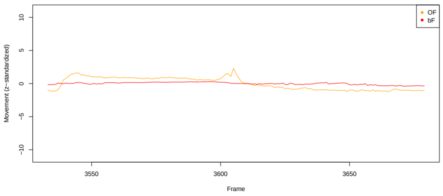

Participant 24 Anxiety, Y_22, BR2_y

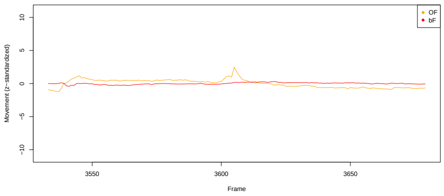

Participant 24 Anxiety, X_49, BL7_x

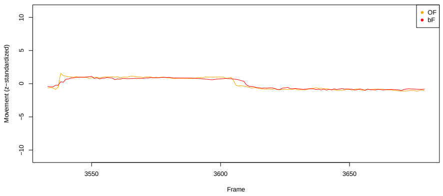

Participant 24 Anxiety, X_53, BR7_x

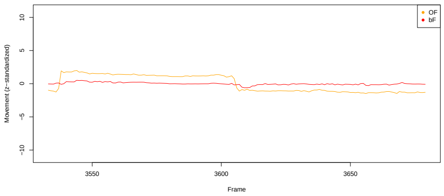

Participant 24 Anxiety, Y_49, BL7_y

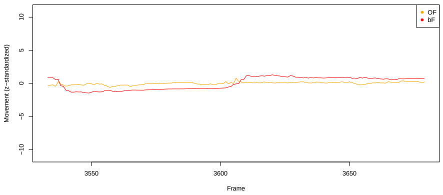

Participant 24 Anxiety, Y_53, BR7_y

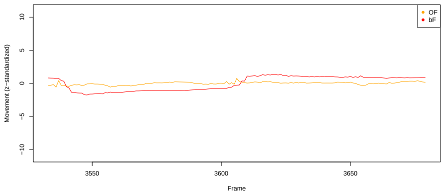

Participant 24 Anxiety, X_48, CL7_x

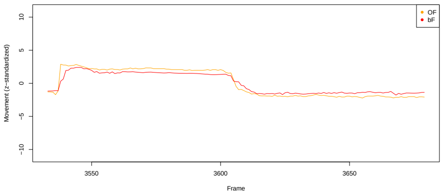

Participant 24 Anxiety, X_54, CR7_x

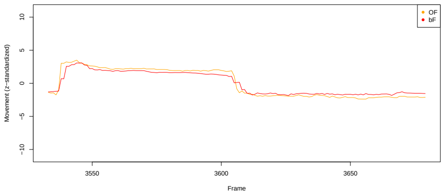

Participant 24 Anxiety, Y_48, CL7_y

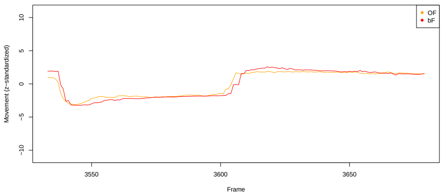

Participant 24 Anxiety, Y_54, CR7_y

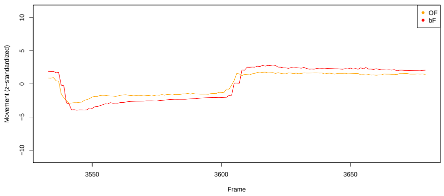

Participant 24 Anxiety, X_51, A7_x

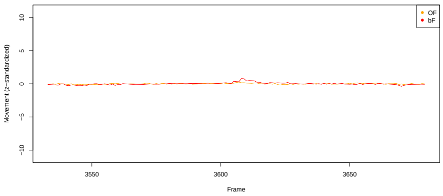

Participant 24 Anxiety, Y_51, A7_y

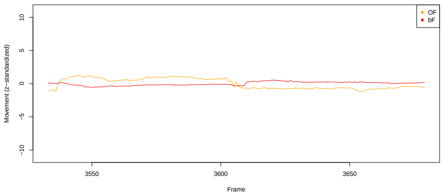

Subject 24, Disgust

Participant 24 Disgust, X_21, BL2_x

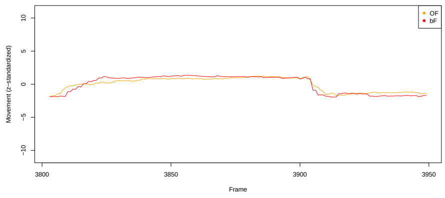

Participant 24 Disgust, X_22, BR2_x

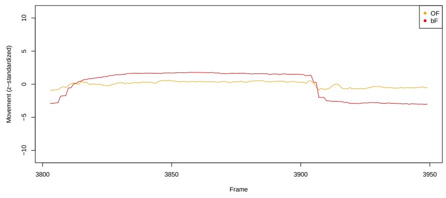

Participant 24 Disgust, Y_21, BL2_y

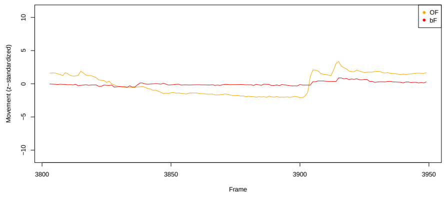

Participant 24 Disgust, Y_22, BR2_y

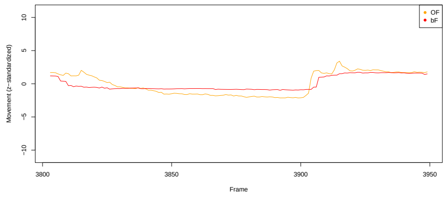

Participant 24 Disgust, X_49, BL7_x

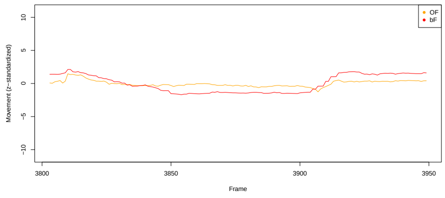

Participant 24 Disgust, X_53, BR7_x

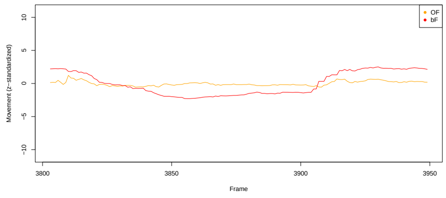

Participant 24 Disgust, Y_49, BL7_y

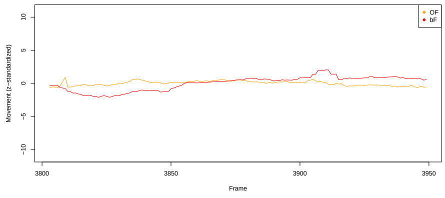

Participant 24 Disgust, Y_53, BR7_y

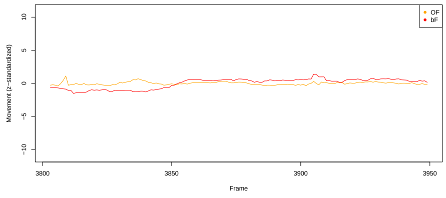

Participant 24 Disgust, X_48, CL7_x

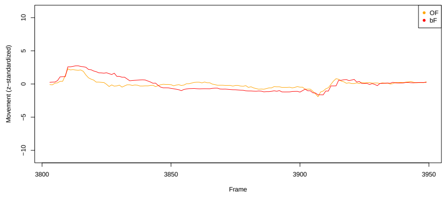

Participant 24 Disgust, X_54, CR7_x

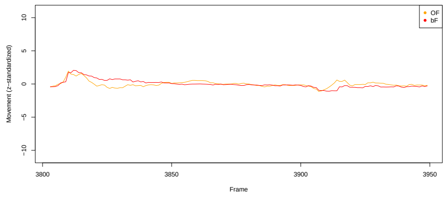

Participant 24 Disgust, Y_48, CL7_y

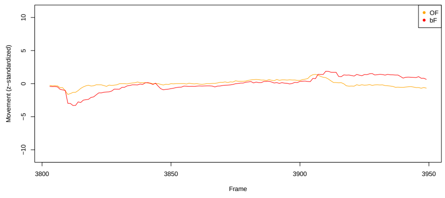

Participant 24 Disgust, Y_54, CR7_y

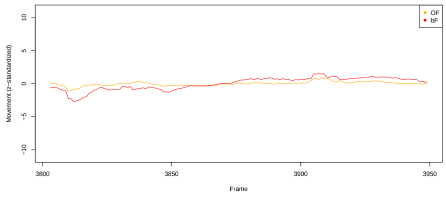

Participant 24 Disgust, X_51, A7_x

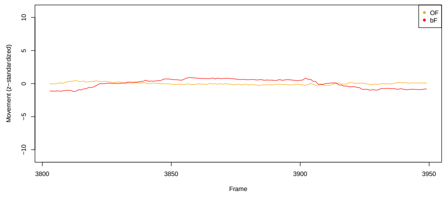

Participant 24 Disgust, Y_51, A7_y

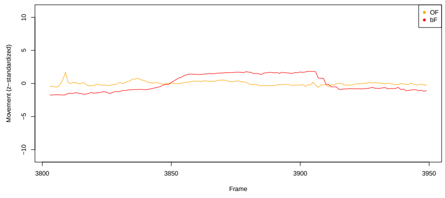

Subject 24, Happiness

Participant 24 Happiness, X_21, BL2_x

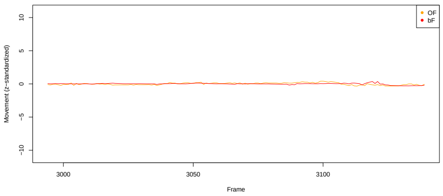

Participant 24 Happiness, X_22, BR2_x

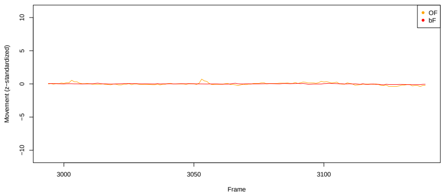

Participant 24 Happiness, Y_21, BL2_y

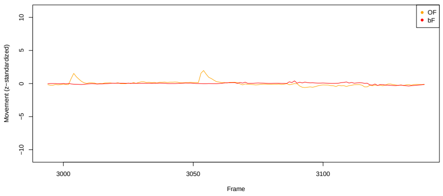

Participant 24 Happiness, Y_22, BR2_y

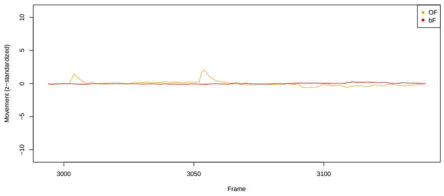

Participant 24 Happiness, X_49, BL7_x

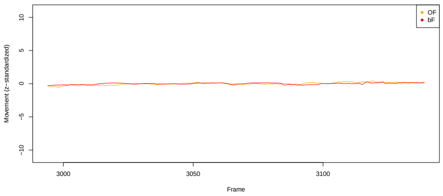

Participant 24 Happiness, X_53, BR7_x

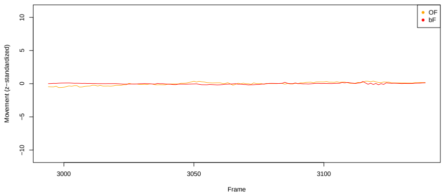

Participant 24 Happiness, Y_49, BL7_y

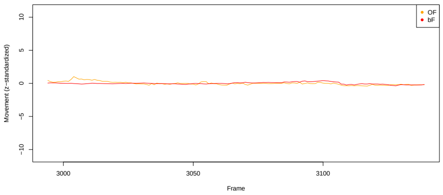

Participant 24 Happiness, Y_53, BR7_y

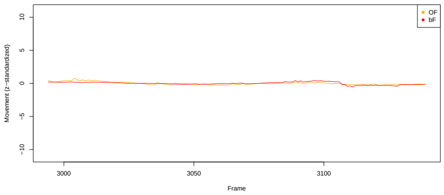

Participant 24 Happiness, X_48, CL7_x

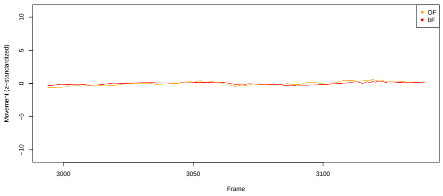

Participant 24 Happiness, X_54, CR7_x

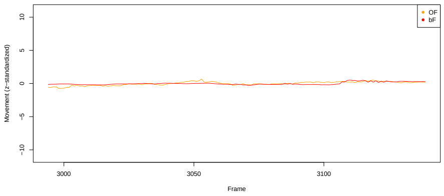

Participant 24 Happiness, Y_48, CL7_y

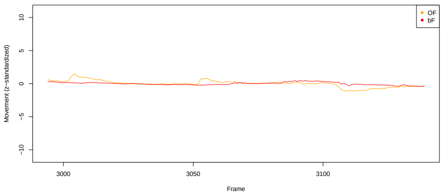

Participant 24 Happiness, Y_54, CR7_y

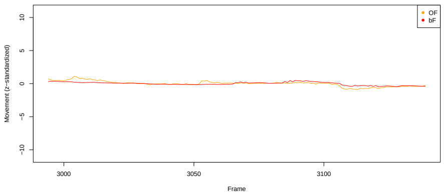

Participant 24 Happiness, X_51, A7_x

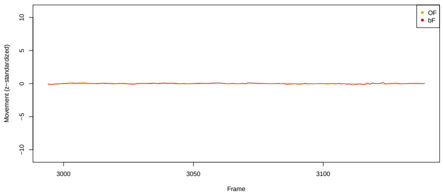

Participant 24 Happiness, Y_51, A7_y

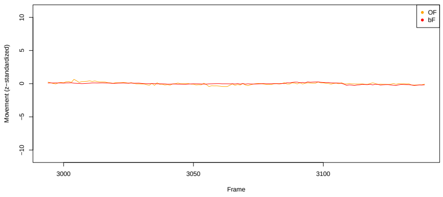

Subject 24, Sadness

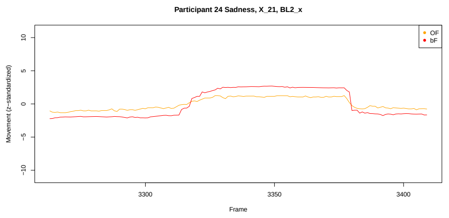

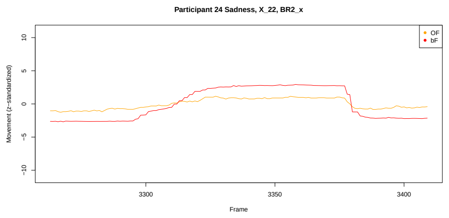

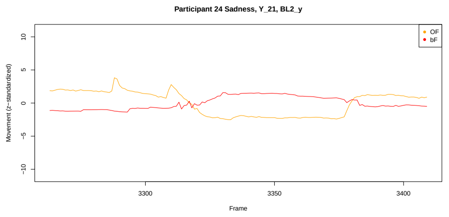

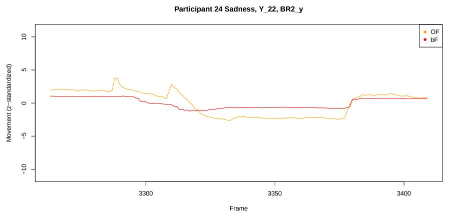

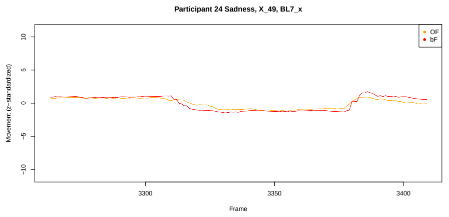

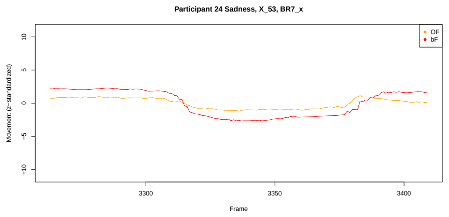

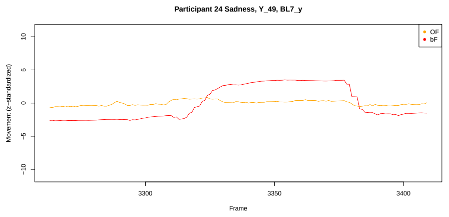

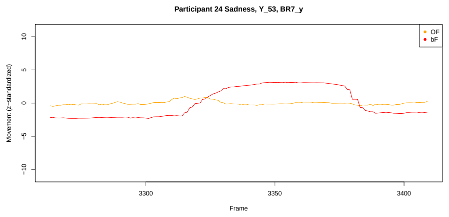

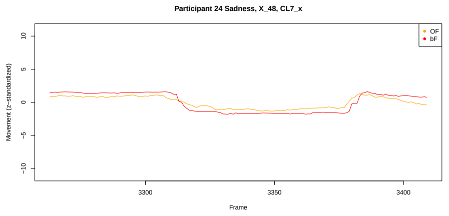

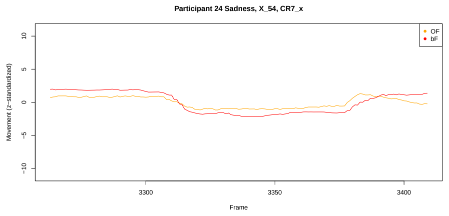

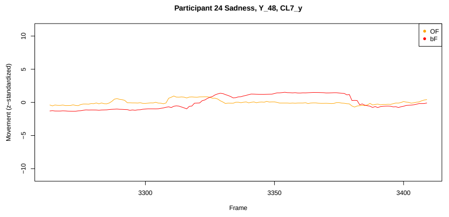

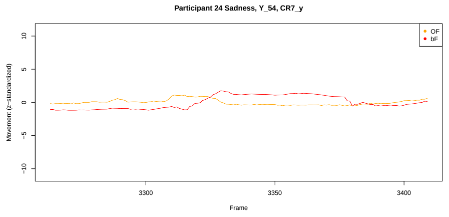

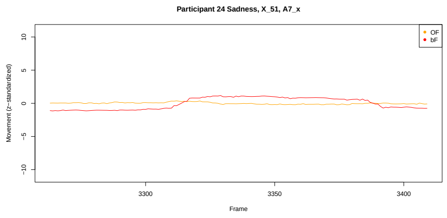

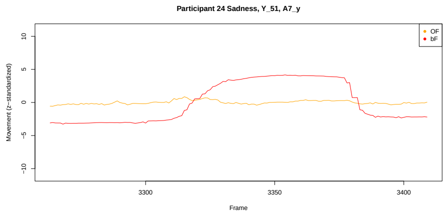

Subject 25, Anger

Participant 25 Anger, X_21, BL2_x

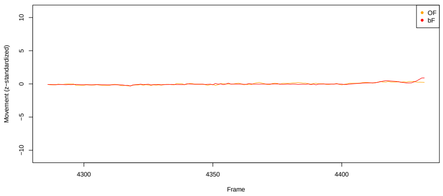

Participant 25 Anger, X_22, BR2_x

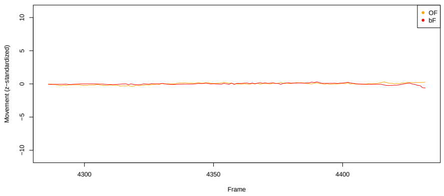

Participant 25 Anger, Y_21, BL2_y

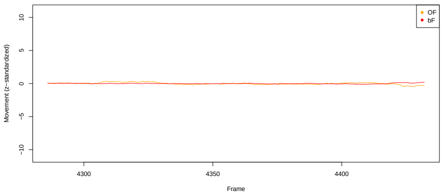

Participant 25 Anger, Y_22, BR2_y

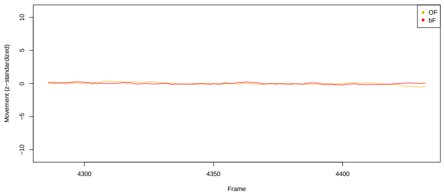

Participant 25 Anger, X_49, BL7_x

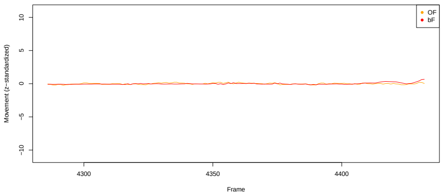

Participant 25 Anger, X_53, BR7_x

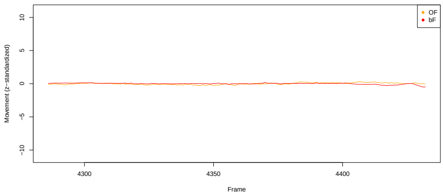

Participant 25 Anger, Y_49, BL7_y

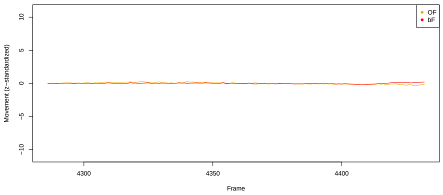

Participant 25 Anger, Y_53, BR7_y

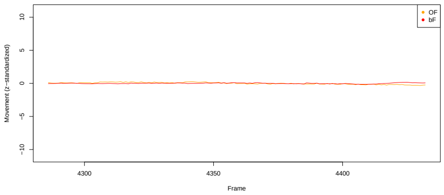

Participant 25 Anger, X_48, CL7_x

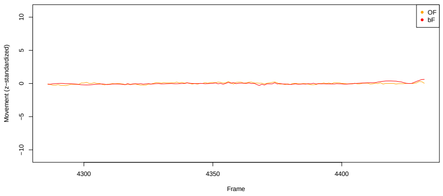

Participant 25 Anger, X_54, CR7_x

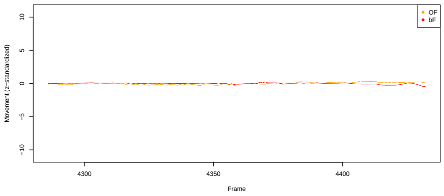

Participant 25 Anger, Y_48, CL7_y

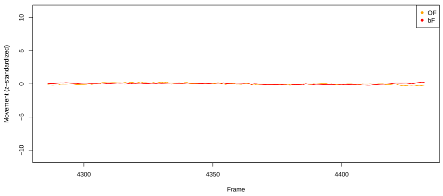

Participant 25 Anger, Y_54, CR7_y

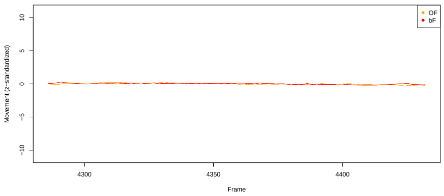

Participant 25 Anger, X_51, A7_x

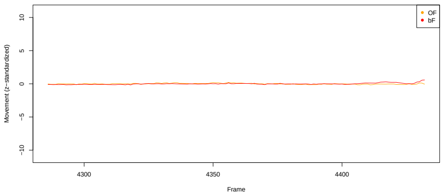

Participant 25 Anger, Y_51, A7_y

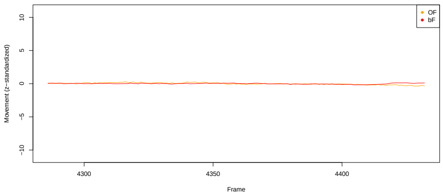

Subject 25, Anxiety

Participant 25 Anxiety, X_21, BL2_x

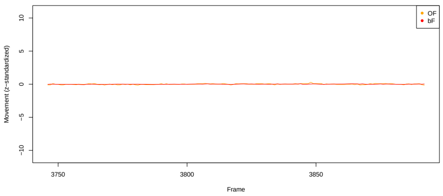

Participant 25 Anxiety, X_22, BR2_x

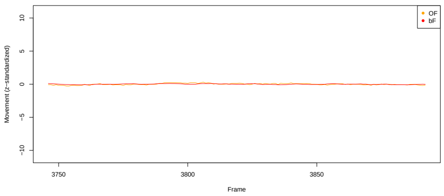

Participant 25 Anxiety, Y_21, BL2_y

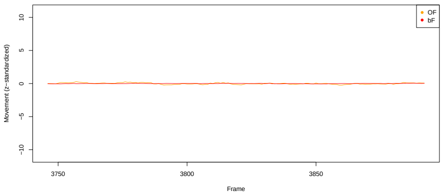

Participant 25 Anxiety, Y_22, BR2_y

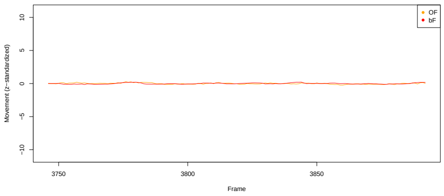

Participant 25 Anxiety, X_49, BL7_x

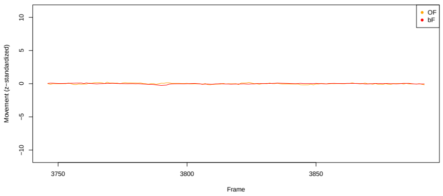

Participant 25 Anxiety, X_53, BR7_x

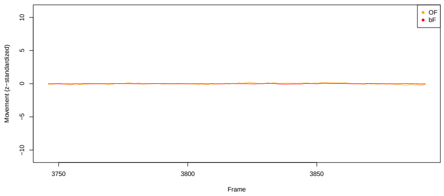

Participant 25 Anxiety, Y_49, BL7_y

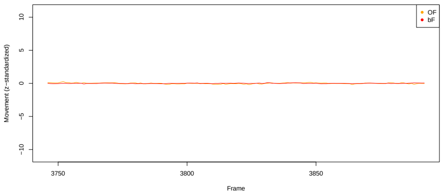

Participant 25 Anxiety, Y_53, BR7_y

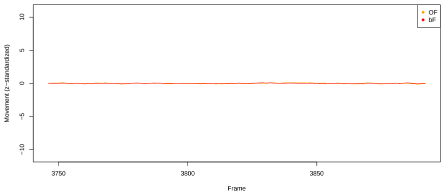

Participant 25 Anxiety, X_48, CL7_x

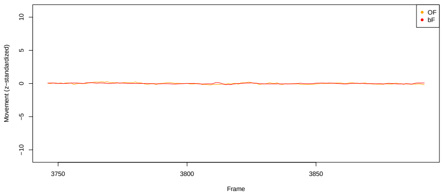

Participant 25 Anxiety, X_54, CR7_x

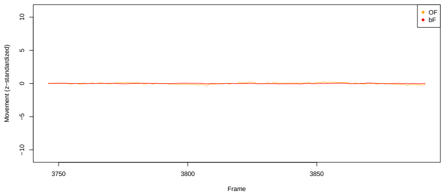

Participant 25 Anxiety, Y_48, CL7_y

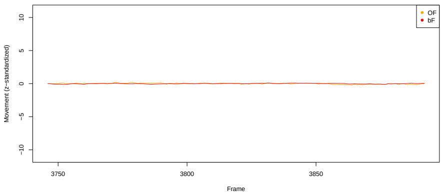

Participant 25 Anxiety, Y_54, CR7_y

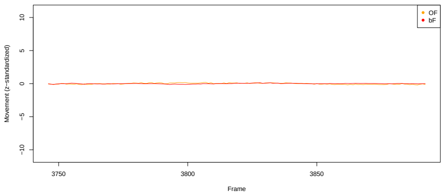

Participant 25 Anxiety, X_51, A7_x

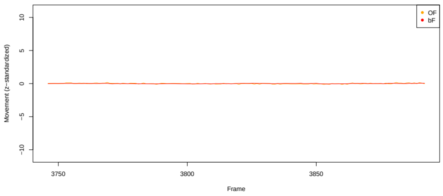

Participant 25 Anxiety, Y_51, A7_y

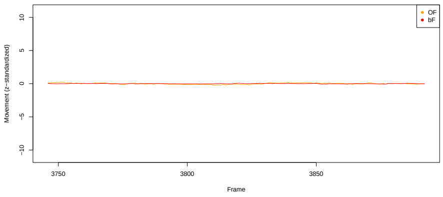

Subject 25, Disgust

Participant 25 Disgust, X_21, BL2_x

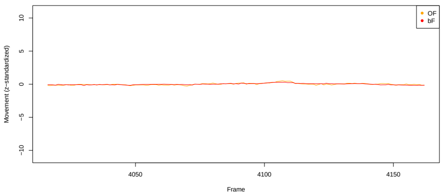

Participant 25 Disgust, X_22, BR2_x

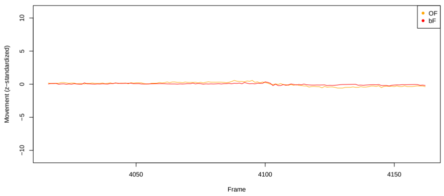

Participant 25 Disgust, Y_21, BL2_y

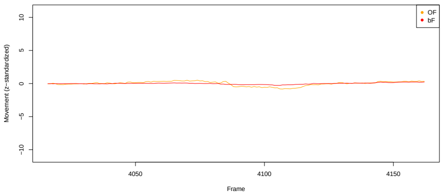

Participant 25 Disgust, Y_22, BR2_y

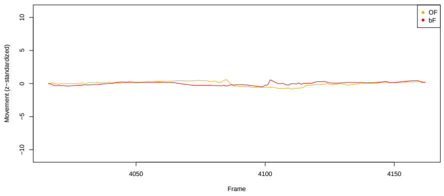

Participant 25 Disgust, X_49, BL7_x

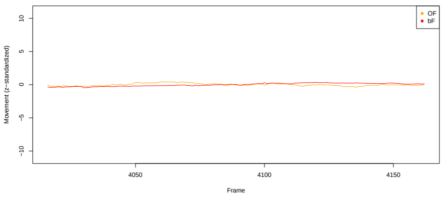

Participant 25 Disgust, X_53, BR7_x

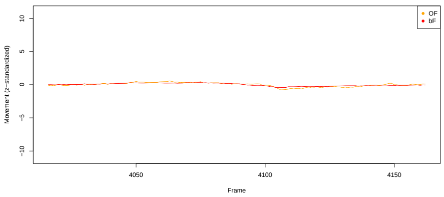

Participant 25 Disgust, Y_49, BL7_y

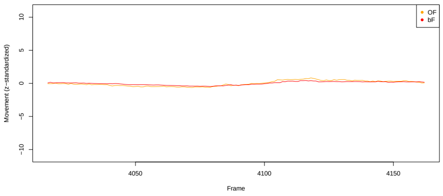

Participant 25 Disgust, Y_53, BR7_y

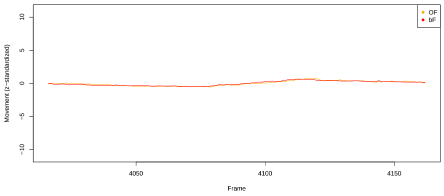

Participant 25 Disgust, X_48, CL7_x

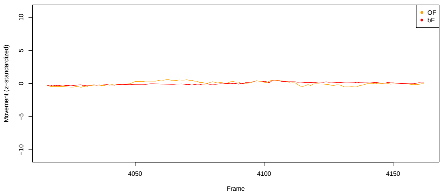

Participant 25 Disgust, X_54, CR7_x

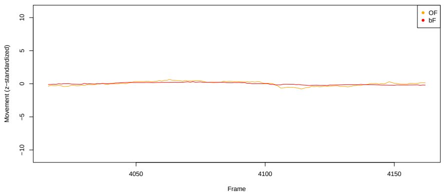

Participant 25 Disgust, Y_48, CL7_y

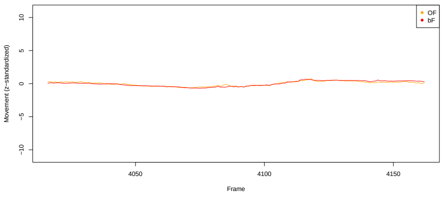

Participant 25 Disgust, Y_54, CR7_y

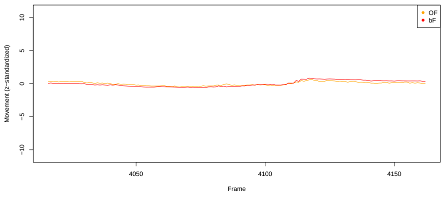

Participant 25 Disgust, X_51, A7_x

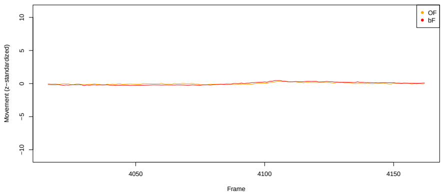

Participant 25 Disgust, Y_51, A7_y

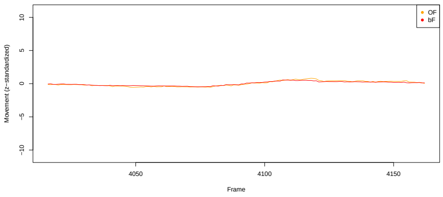

Subject 25, Happiness

Participant 25 Happiness, X_21, BL2_x

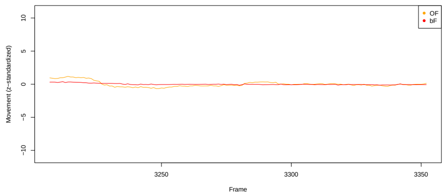

Participant 25 Happiness, X_22, BR2_x

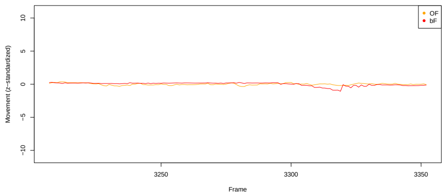

Participant 25 Happiness, Y_21, BL2_y

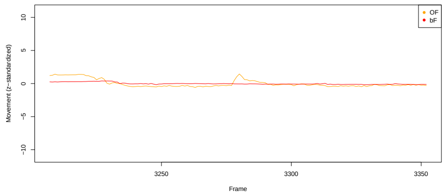

Participant 25 Happiness, Y_22, BR2_y

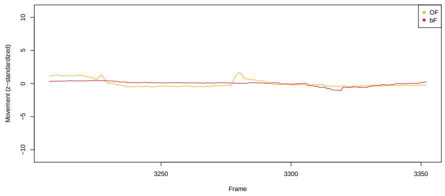

Participant 25 Happiness, X_49, BL7_x

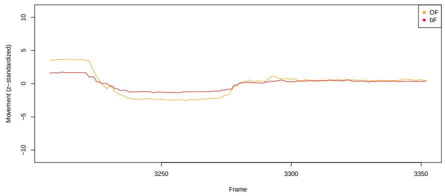

Participant 25 Happiness, X_53, BR7_x

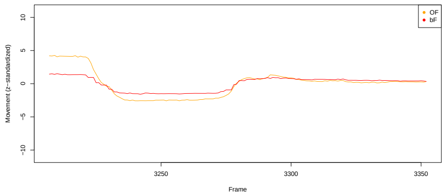

Participant 25 Happiness, Y_49, BL7_y

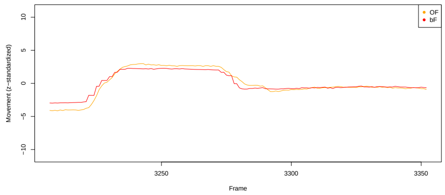

Participant 25 Happiness, Y_53, BR7_y

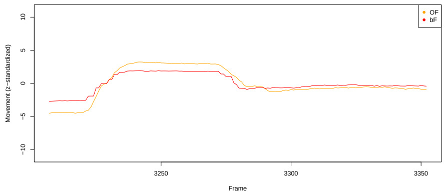

Participant 25 Happiness, X_48, CL7_x

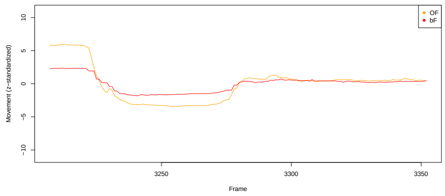

Participant 25 Happiness, X_54, CR7_x

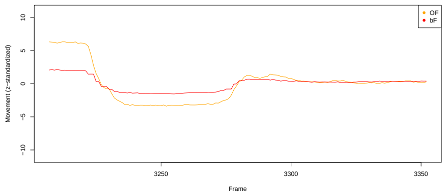

Participant 25 Happiness, Y_48, CL7_y

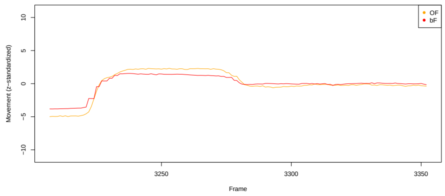

Participant 25 Happiness, Y_54, CR7_y

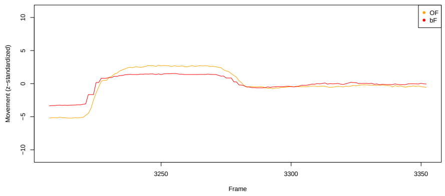

Participant 25 Happiness, X_51, A7_x

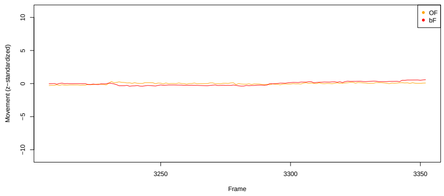

Participant 25 Happiness, Y_51, A7_y

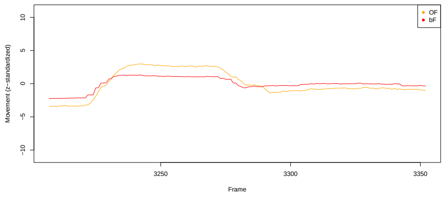

Subject 25, Sadness

Participant 25 Sadness, X_21, BL2_x

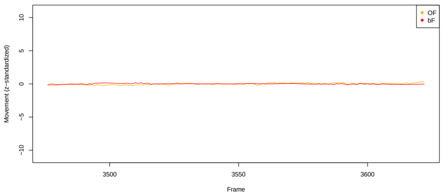

Participant 25 Sadness, X_22, BR2_x

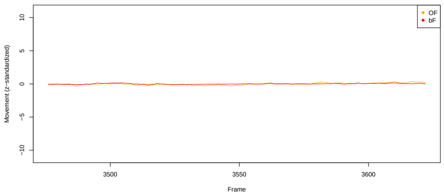

Participant 25 Sadness, Y_21, BL2_y

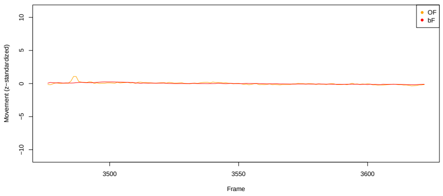

Participant 25 Sadness, Y_22, BR2_y

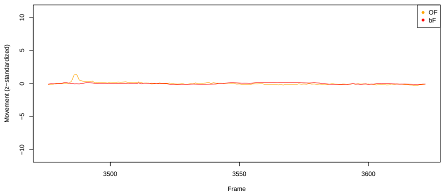

Participant 25 Sadness, X_49, BL7_x

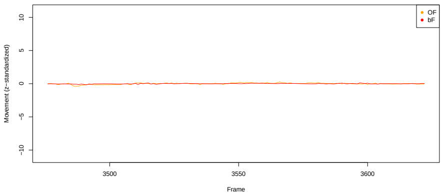

Participant 25 Sadness, X_53, BR7_x

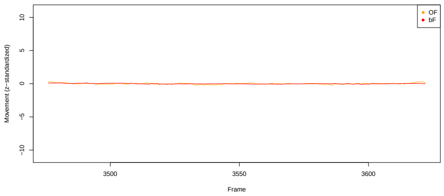

Participant 25 Sadness, Y_49, BL7_y

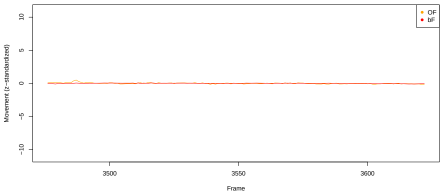

Participant 25 Sadness, Y_53, BR7_y

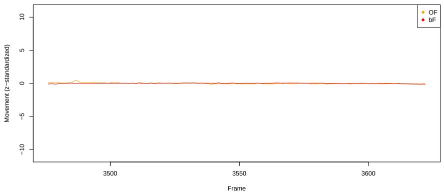

Participant 25 Sadness, X_48, CL7_x

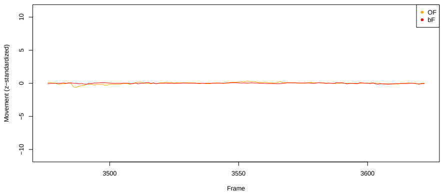

Participant 25 Sadness, X_54, CR7_x

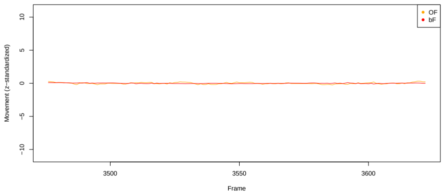

Participant 25 Sadness, Y_48, CL7_y

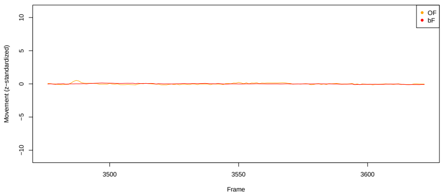

Participant 25 Sadness, Y_54, CR7_y

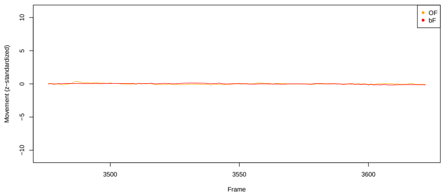

Participant 25 Sadness, X_51, A7_x

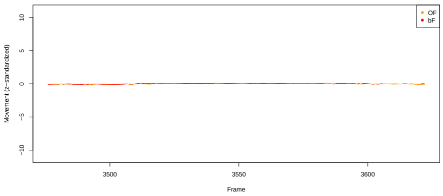

Participant 25 Sadness, Y_51, A7_y

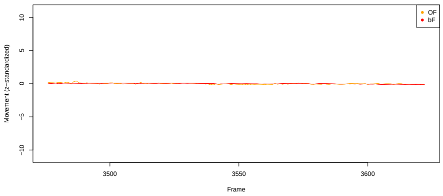

Subject 26, Anger

Participant 26 Anger, X_21, BL2_x

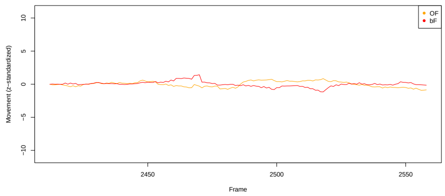

Participant 26 Anger, X_22, BR2_x

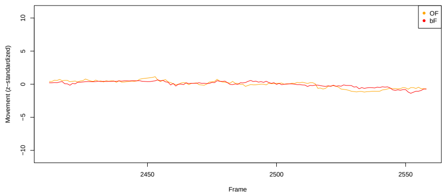

Participant 26 Anger, Y_21, BL2_y

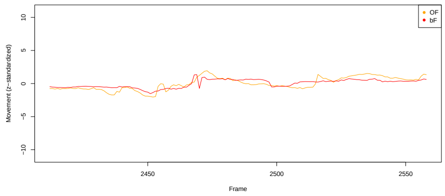

Participant 26 Anger, Y_22, BR2_y

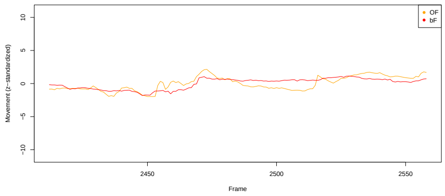

Participant 26 Anger, X_49, BL7_x

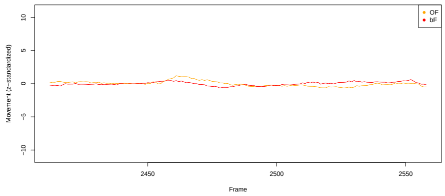

Participant 26 Anger, X_53, BR7_x

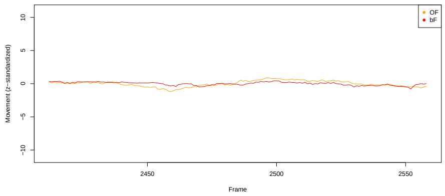

Participant 26 Anger, Y_49, BL7_y

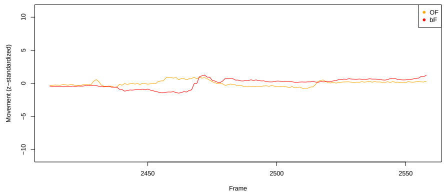

Participant 26 Anger, Y_53, BR7_y

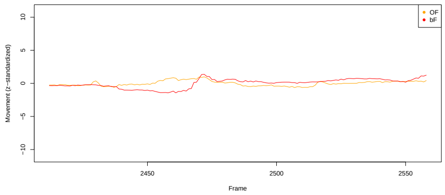

Participant 26 Anger, X_48, CL7_x

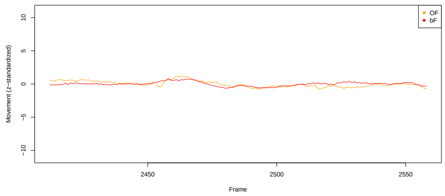

Participant 26 Anger, X_54, CR7_x

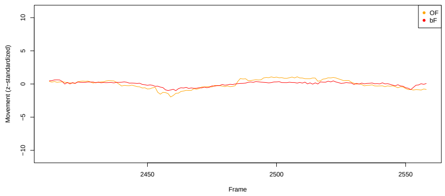

Participant 26 Anger, Y_48, CL7_y

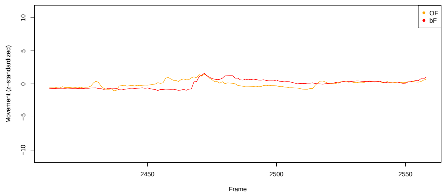

Participant 26 Anger, Y_54, CR7_y

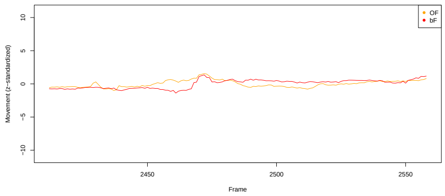

Participant 26 Anger, X_51, A7_x

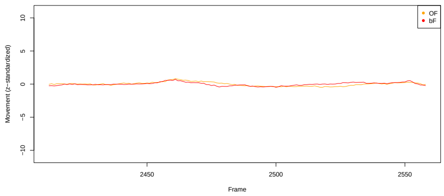

Participant 26 Anger, Y_51, A7_y

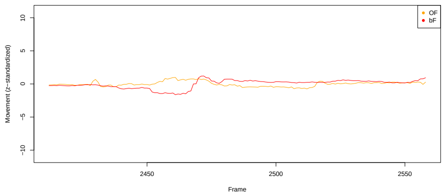

Subject 26, Anxiety

Participant 26 Anxiety, X_21, BL2_x

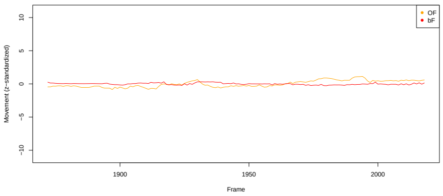

Participant 26 Anxiety, X_22, BR2_x

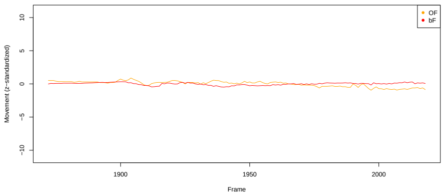

Participant 26 Anxiety, Y_21, BL2_y

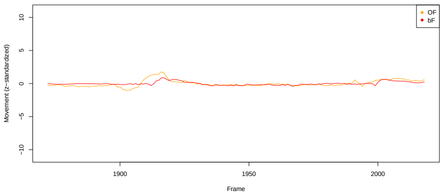

Participant 26 Anxiety, Y_22, BR2_y

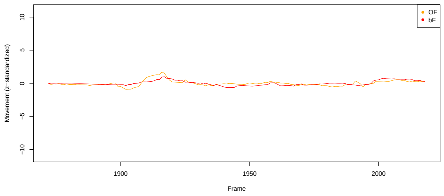

Participant 26 Anxiety, X_49, BL7_x

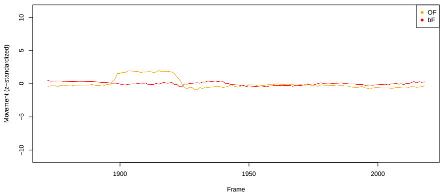

Participant 26 Anxiety, X_53, BR7_x

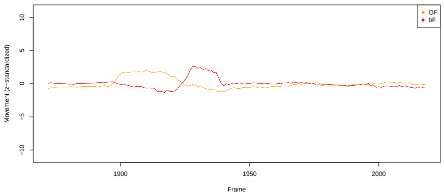

Participant 26 Anxiety, Y_49, BL7_y

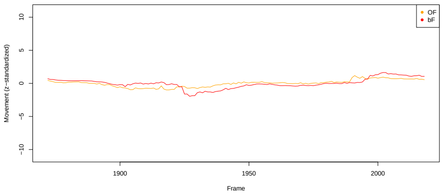

Participant 26 Anxiety, Y_53, BR7_y

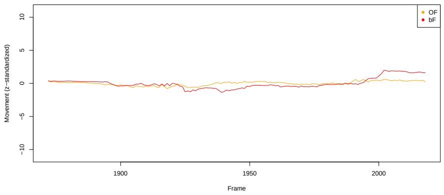

Participant 26 Anxiety, X_48, CL7_x

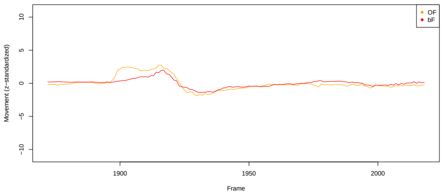

Participant 26 Anxiety, X_54, CR7_x

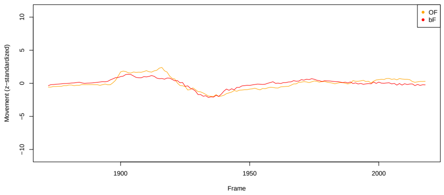

Participant 26 Anxiety, Y_48, CL7_y

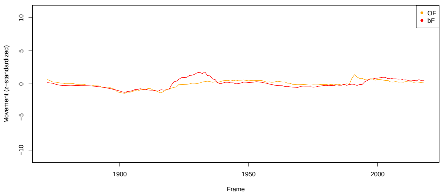

Participant 26 Anxiety, Y_54, CR7_y

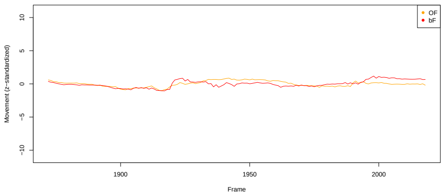

Participant 26 Anxiety, X_51, A7_x

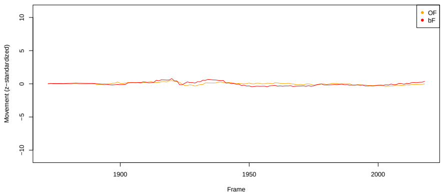

Participant 26 Anxiety, Y_51, A7_y

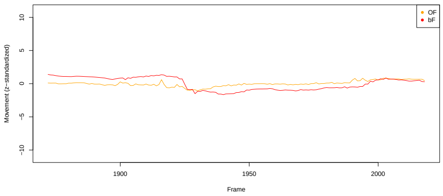

Subject 26, Disgust

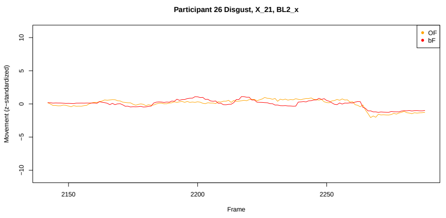

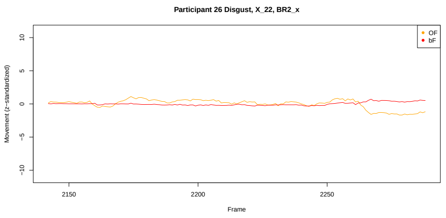

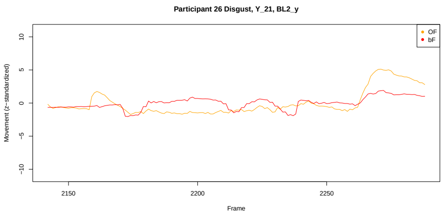

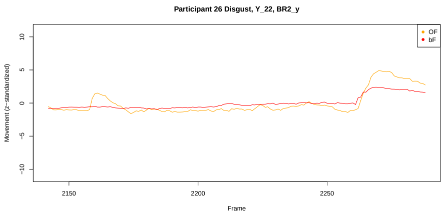

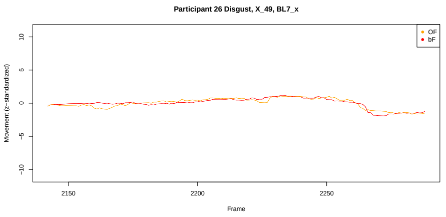

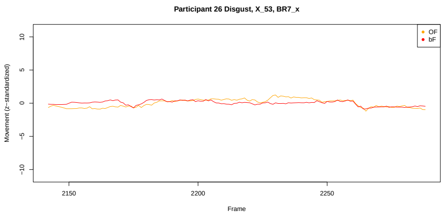

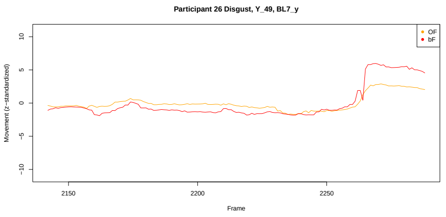

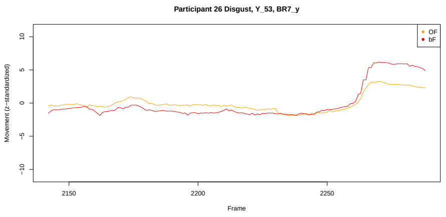

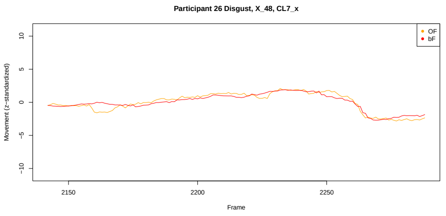

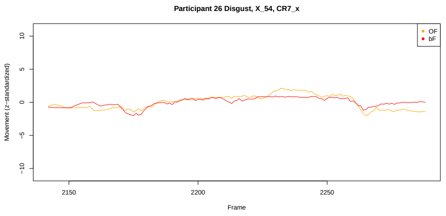

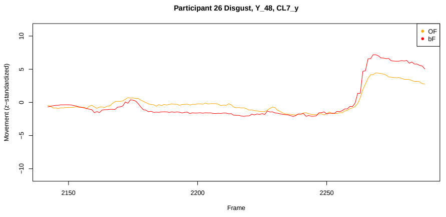

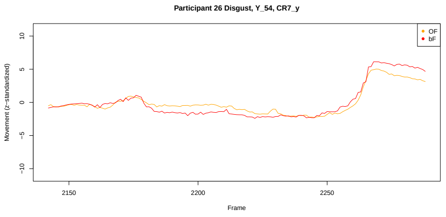

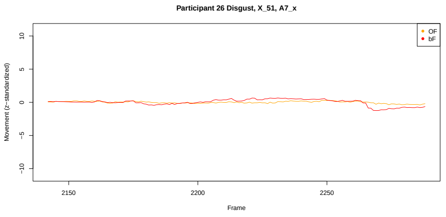

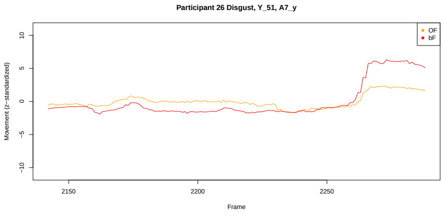

Subject 26, Happiness

Participant 26 Happiness, X_21, BL2_x

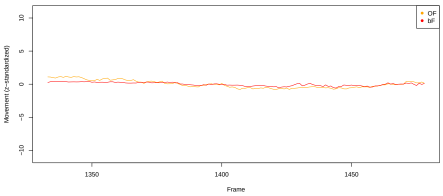

Participant 26 Happiness, X_22, BR2_x

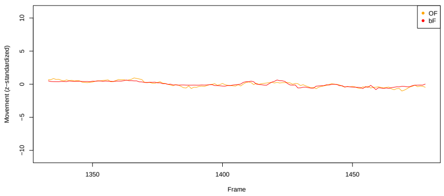

Participant 26 Happiness, Y_21, BL2_y

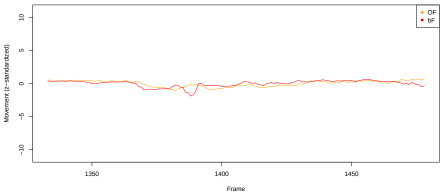

Participant 26 Happiness, Y_22, BR2_y

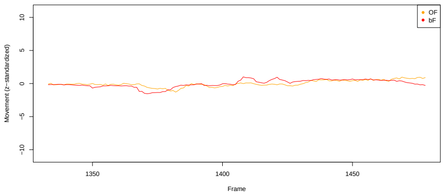

Participant 26 Happiness, X_49, BL7_x

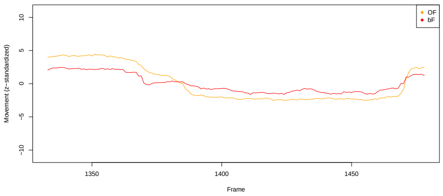

Participant 26 Happiness, X_53, BR7_x

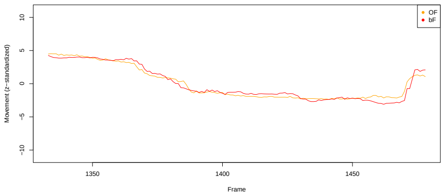

Participant 26 Happiness, Y_49, BL7_y

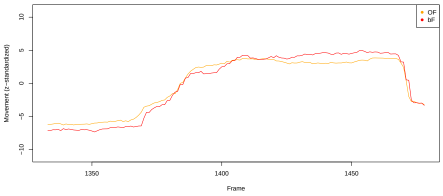

Participant 26 Happiness, Y_53, BR7_y

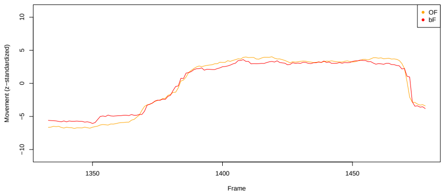

Participant 26 Happiness, X_48, CL7_x

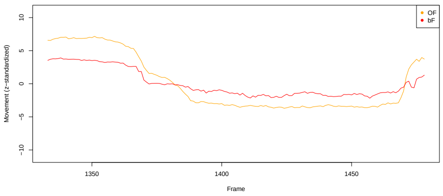

Participant 26 Happiness, X_54, CR7_x

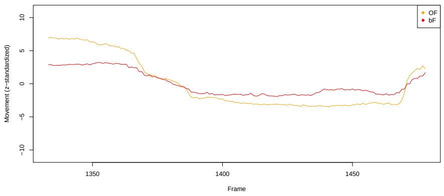

Participant 26 Happiness, Y_48, CL7_y

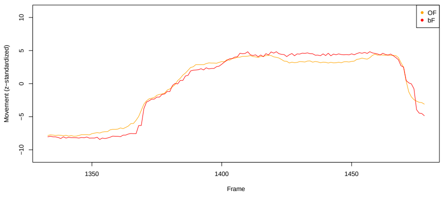

Participant 26 Happiness, Y_54, CR7_y

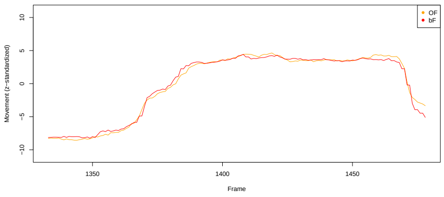

Participant 26 Happiness, X_51, A7_x

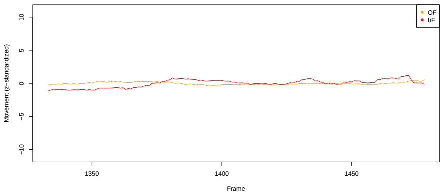

Participant 26 Happiness, Y_51, A7_y

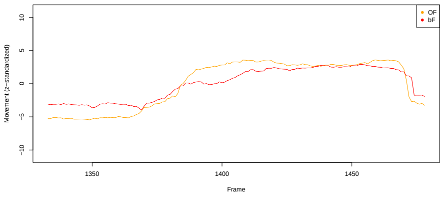

Subject 26, Sadness

Participant 26 Sadness, X_21, BL2_x

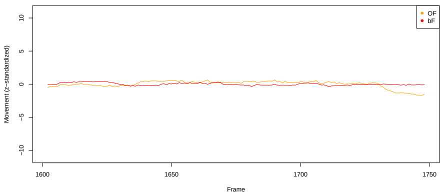

Participant 26 Sadness, X_22, BR2_x

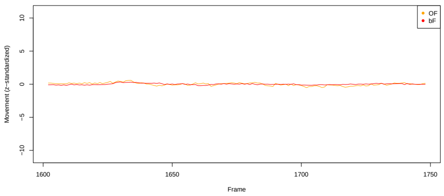

Participant 26 Sadness, Y_21, BL2_y

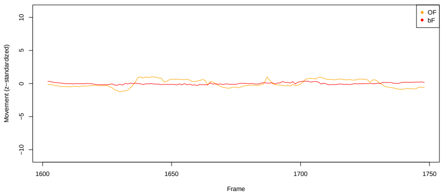

Participant 26 Sadness, Y_22, BR2_y

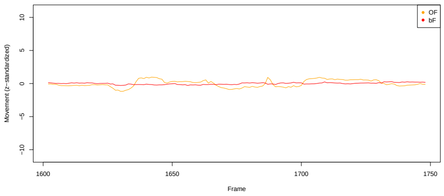

Participant 26 Sadness, X_49, BL7_x

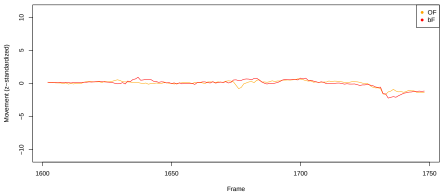

Participant 26 Sadness, X_53, BR7_x

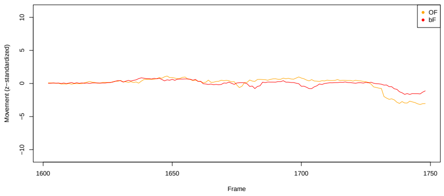

Participant 26 Sadness, Y_49, BL7_y

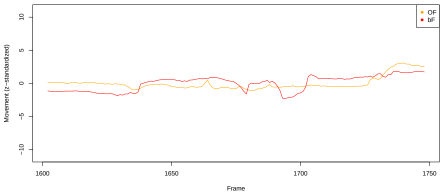

Participant 26 Sadness, Y_53, BR7_y

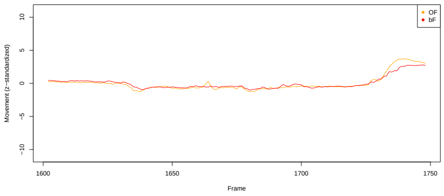

Participant 26 Sadness, X_48, CL7_x

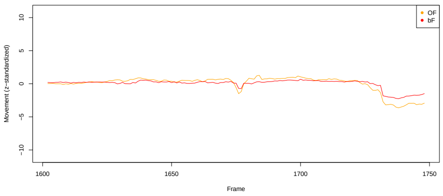

Participant 26 Sadness, X_54, CR7_x

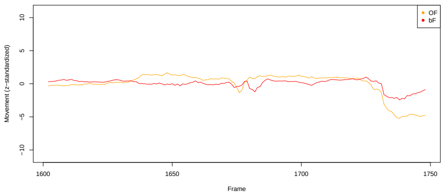

Participant 26 Sadness, Y_48, CL7_y

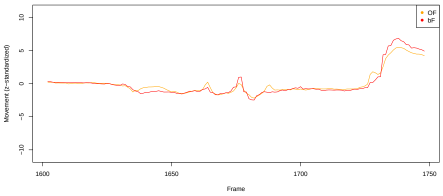

Participant 26 Sadness, Y_54, CR7_y

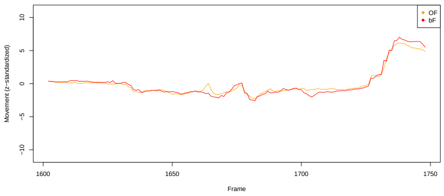

Participant 26 Sadness, X_51, A7_x

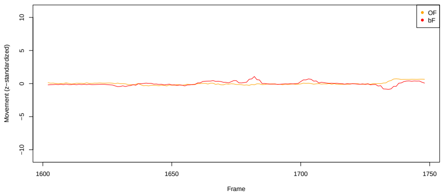

Participant 26 Sadness, Y_51, A7_y

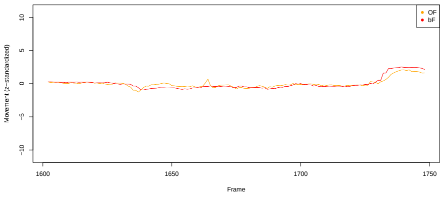

Subject 30, Anger

Participant 30 Anger, X_21, BL2_x

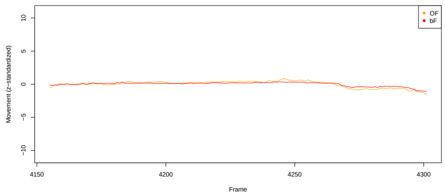

Participant 30 Anger, X_22, BR2_x

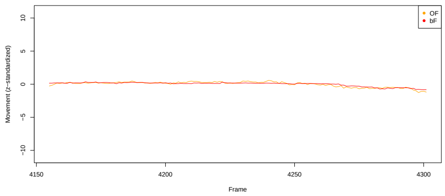

Participant 30 Anger, Y_21, BL2_y

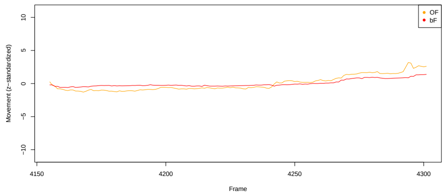

Participant 30 Anger, Y_22, BR2_y

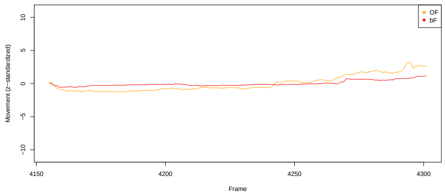

Participant 30 Anger, X_49, BL7_x

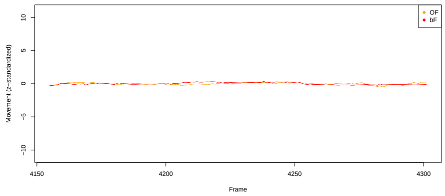

Participant 30 Anger, X_53, BR7_x

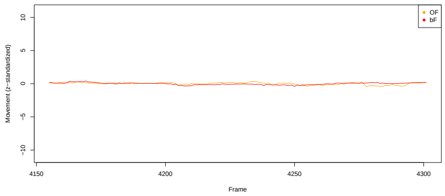

Participant 30 Anger, Y_49, BL7_y

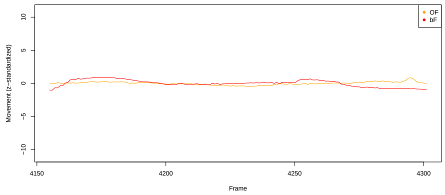

Participant 30 Anger, Y_53, BR7_y

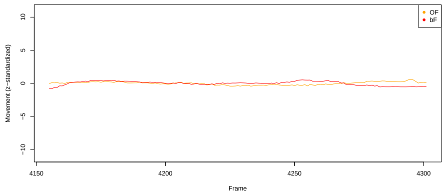

Participant 30 Anger, X_48, CL7_x

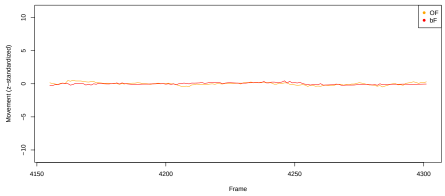

Participant 30 Anger, X_54, CR7_x

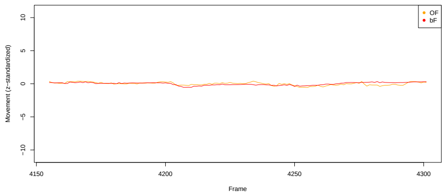

Participant 30 Anger, Y_48, CL7_y

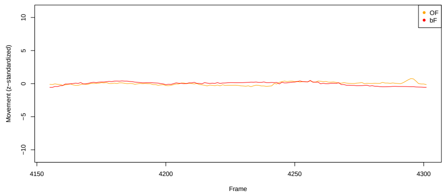

Participant 30 Anger, Y_54, CR7_y

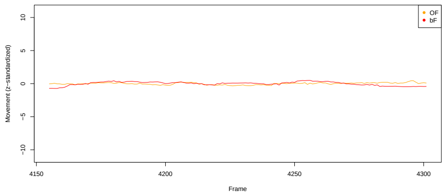

Participant 30 Anger, X_51, A7_x

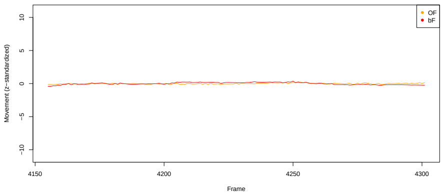

Participant 30 Anger, Y_51, A7_y

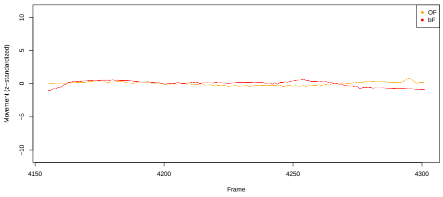

Subject 30, Anxiety

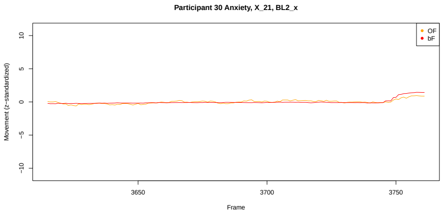

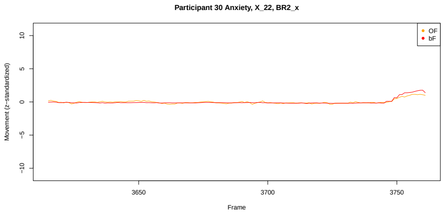

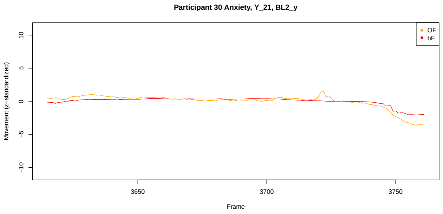

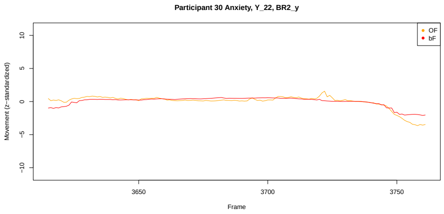

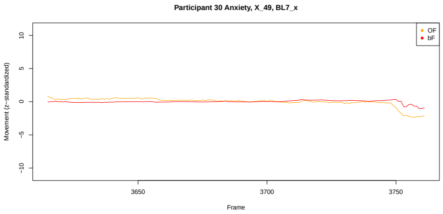

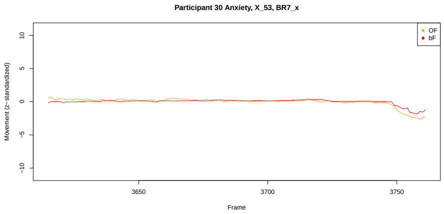

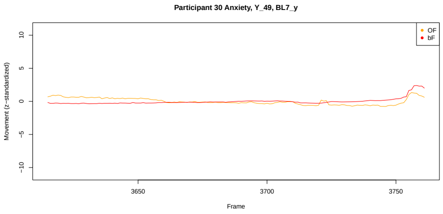

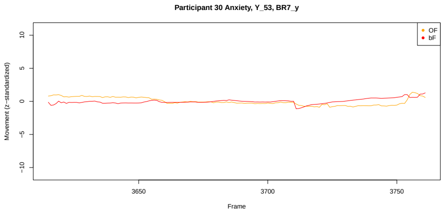

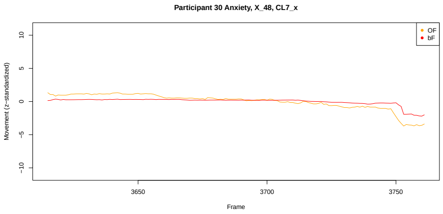

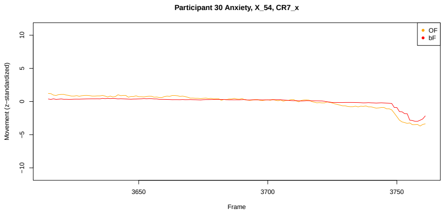

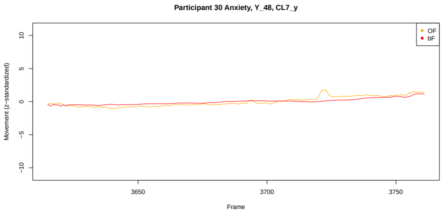

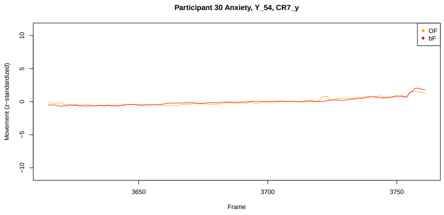

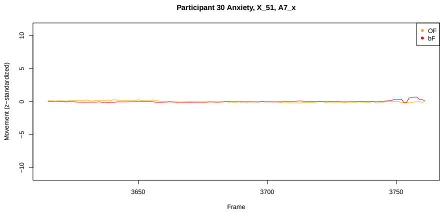

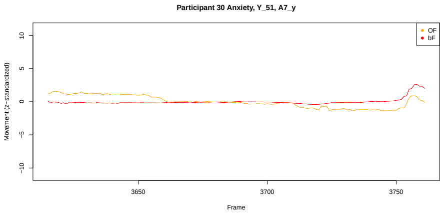

Subject 30, Disgust

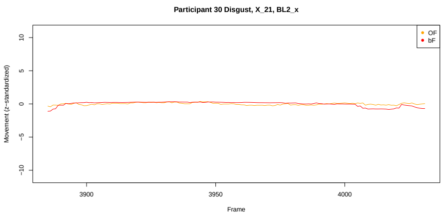

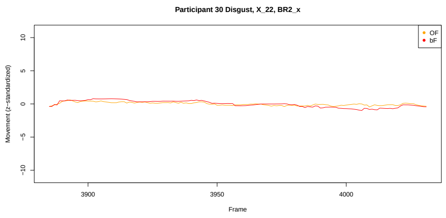

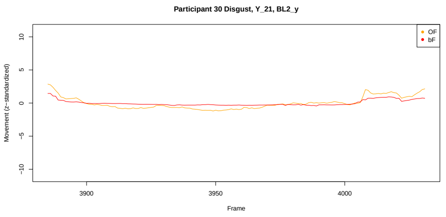

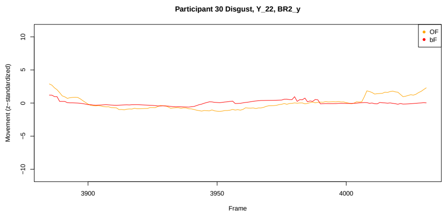

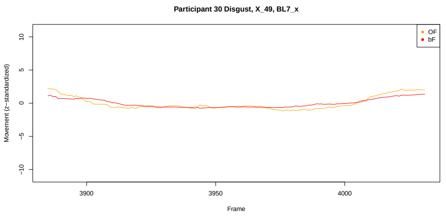

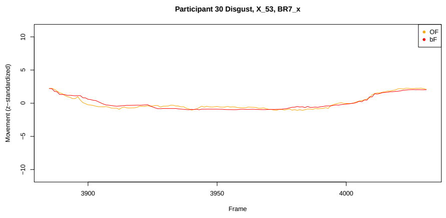

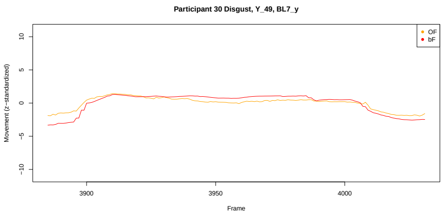

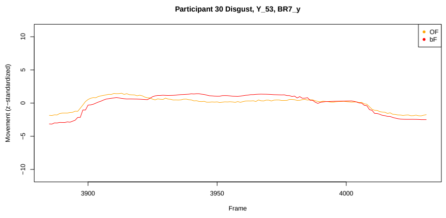

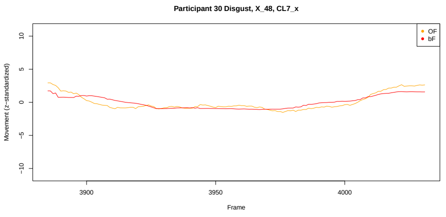

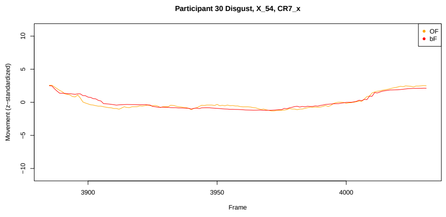

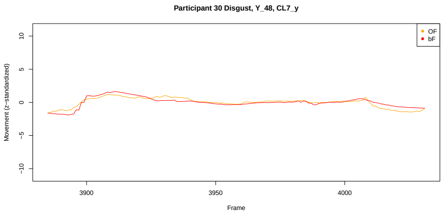

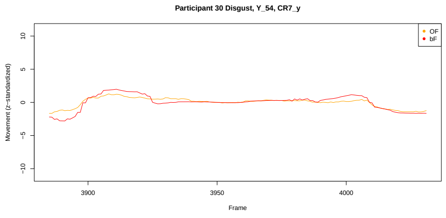

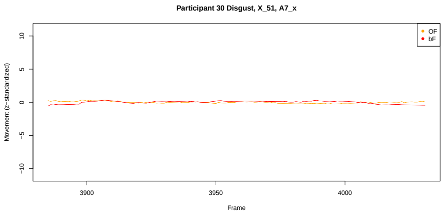

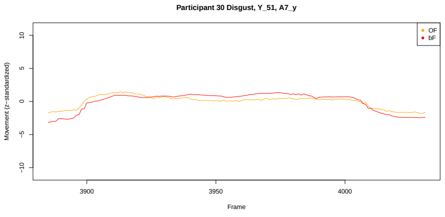

Subject 30, Happiness

Participant 30 Happiness, X_21, BL2_x

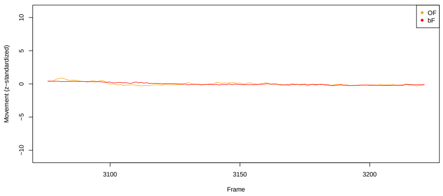

Participant 30 Happiness, X_22, BR2_x

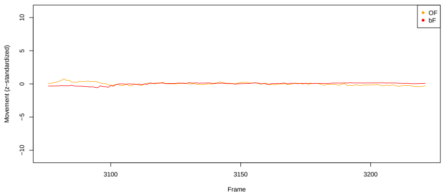

Participant 30 Happiness, Y_21, BL2_y

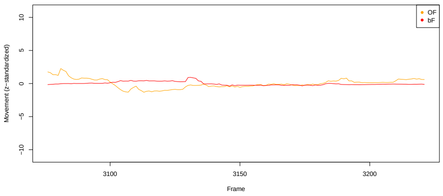

Participant 30 Happiness, Y_22, BR2_y

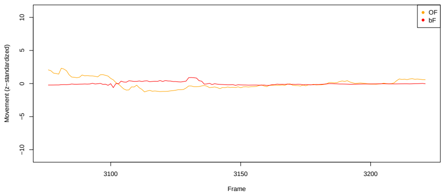

Participant 30 Happiness, X_49, BL7_x

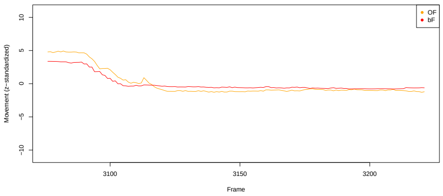

Participant 30 Happiness, X_53, BR7_x

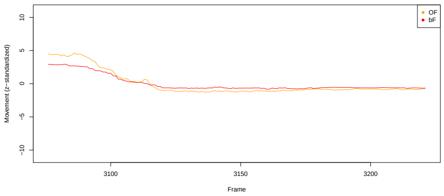

Participant 30 Happiness, Y_49, BL7_y

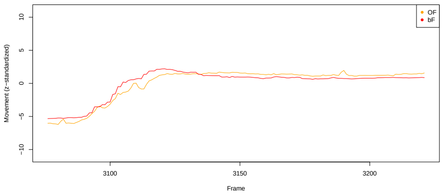

Participant 30 Happiness, Y_53, BR7_y

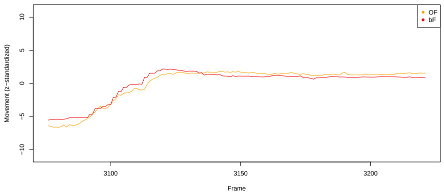

Participant 30 Happiness, X_48, CL7_x

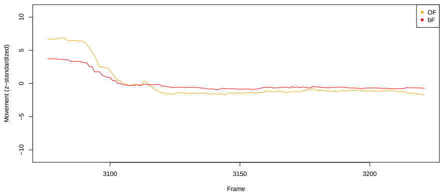

Participant 30 Happiness, X_54, CR7_x

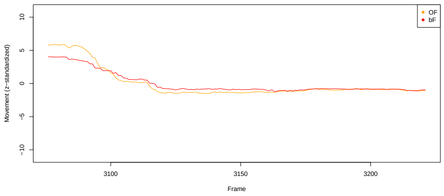

Participant 30 Happiness, Y_48, CL7_y

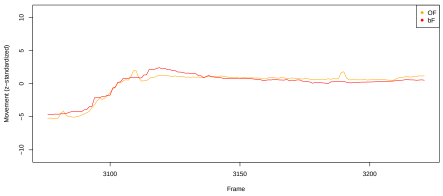

Participant 30 Happiness, Y_54, CR7_y

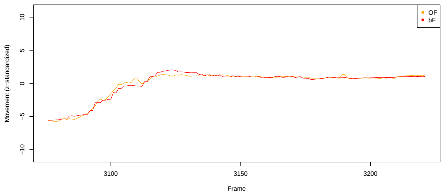

Participant 30 Happiness, X_51, A7_x

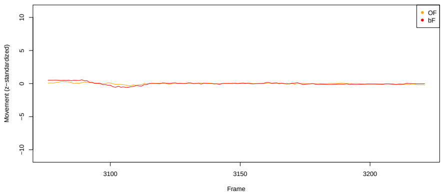

Participant 30 Happiness, Y_51, A7_y

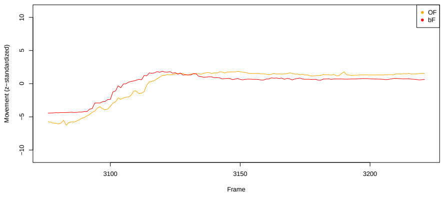

Subject 30, Sadness

Participant 30 Sadness, X_21, BL2_x

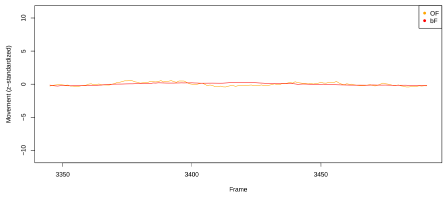

Participant 30 Sadness, X_22, BR2_x

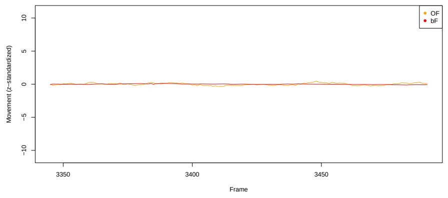

Participant 30 Sadness, Y_21, BL2_y

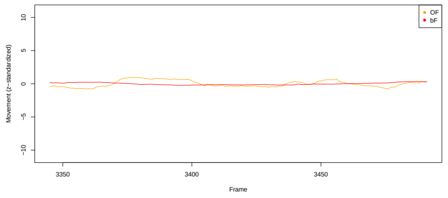

Participant 30 Sadness, Y_22, BR2_y

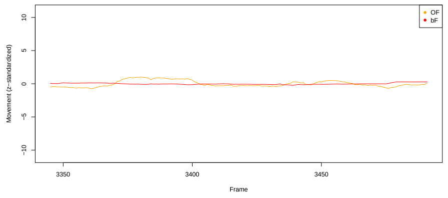

Participant 30 Sadness, X_49, BL7_x

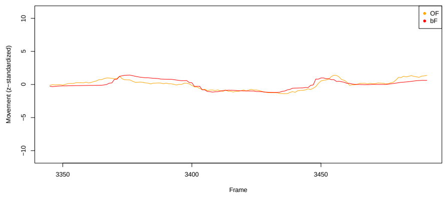

Participant 30 Sadness, X_53, BR7_x

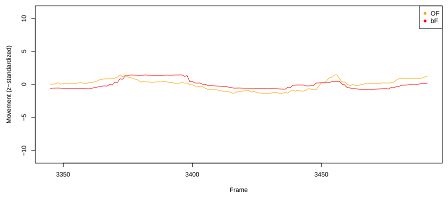

Participant 30 Sadness, Y_49, BL7_y

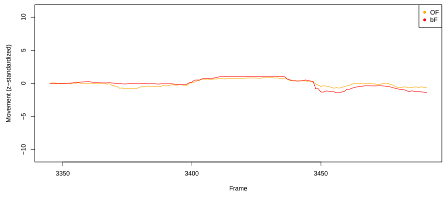

Participant 30 Sadness, Y_53, BR7_y

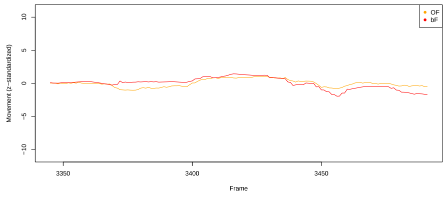

Participant 30 Sadness, X_48, CL7_x

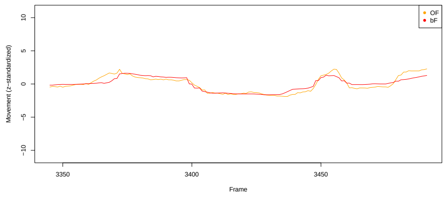

Participant 30 Sadness, X_54, CR7_x

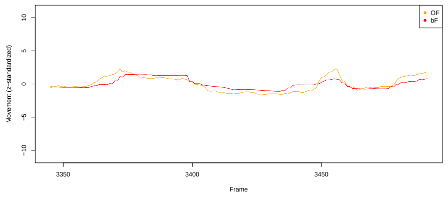

Participant 30 Sadness, Y_48, CL7_y

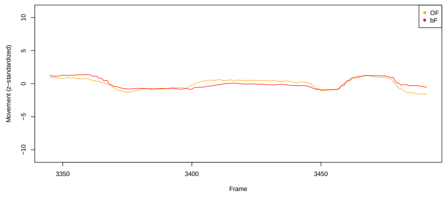

Participant 30 Sadness, Y_54, CR7_y

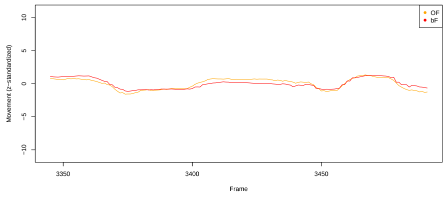

Participant 30 Sadness, X_51, A7_x

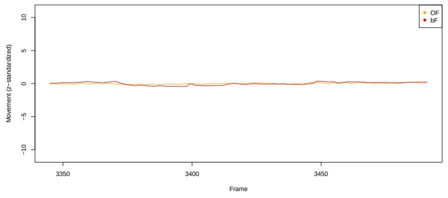

Participant 30 Sadness, Y_51, A7_y

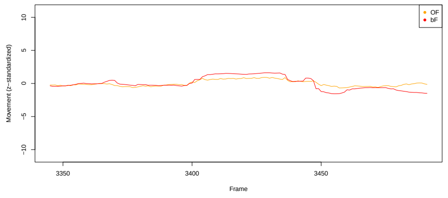

Subject 31, Anger

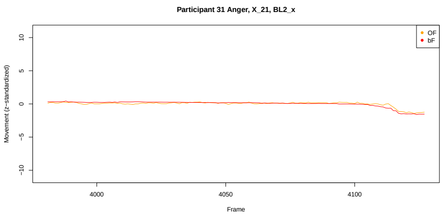

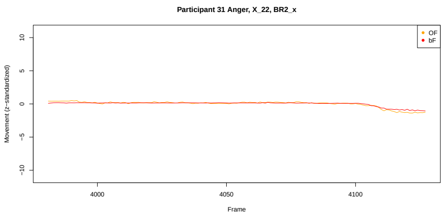

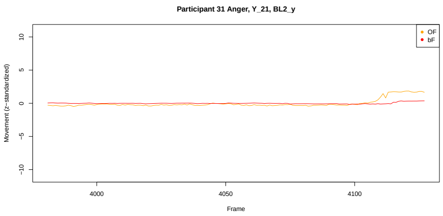

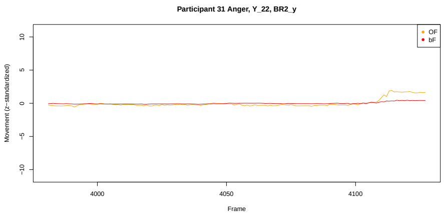

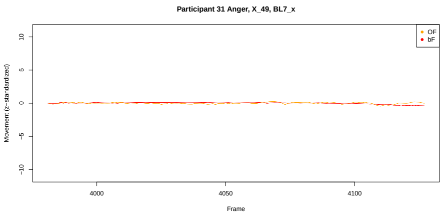

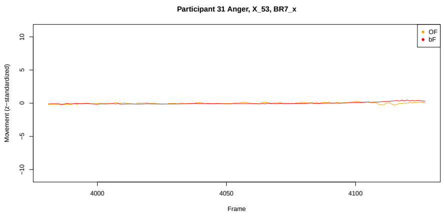

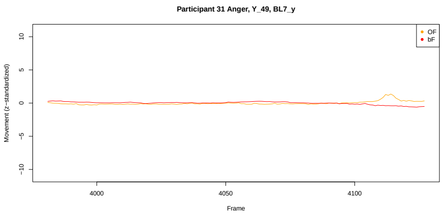

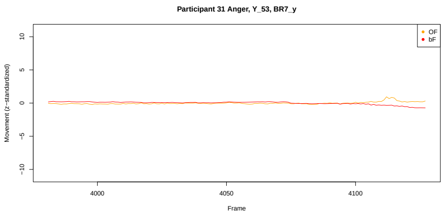

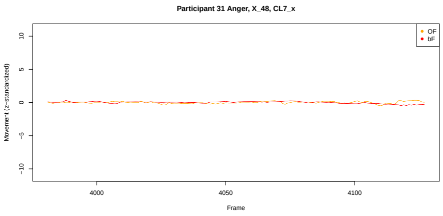

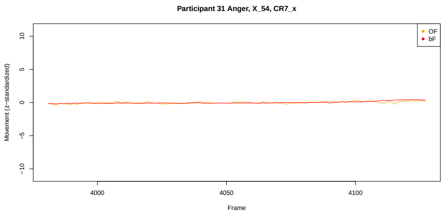

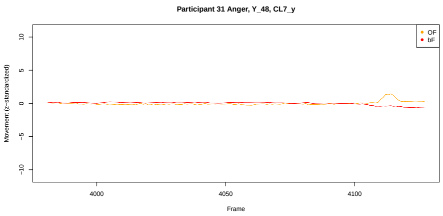

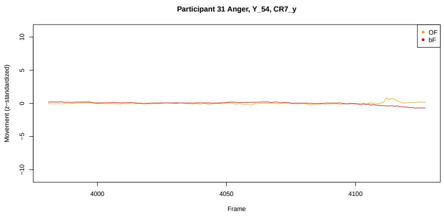

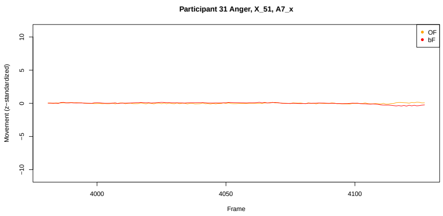

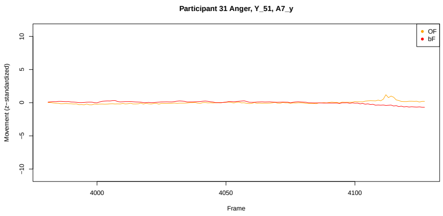

Subject 31, Anxiety

Participant 31 Anxiety, X_21, BL2_x

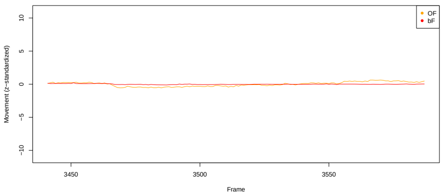

Participant 31 Anxiety, X_22, BR2_x

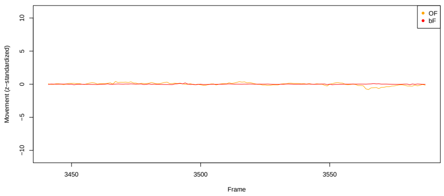

Participant 31 Anxiety, Y_21, BL2_y

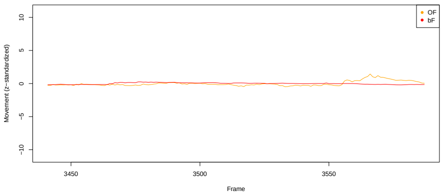

Participant 31 Anxiety, Y_22, BR2_y

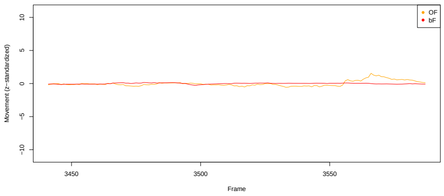

Participant 31 Anxiety, X_49, BL7_x

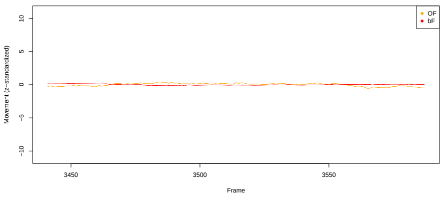

Participant 31 Anxiety, X_53, BR7_x

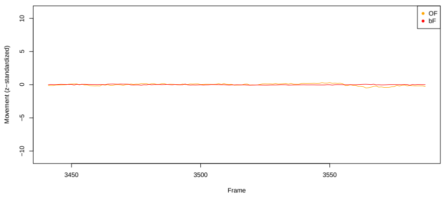

Participant 31 Anxiety, Y_49, BL7_y

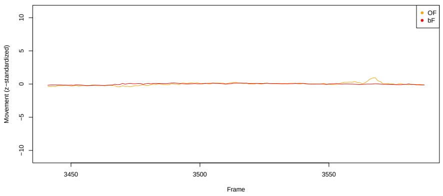

Participant 31 Anxiety, Y_53, BR7_y

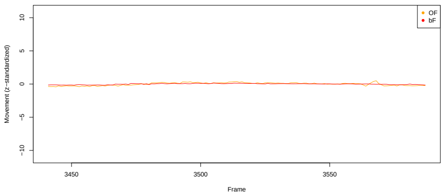

Participant 31 Anxiety, X_48, CL7_x

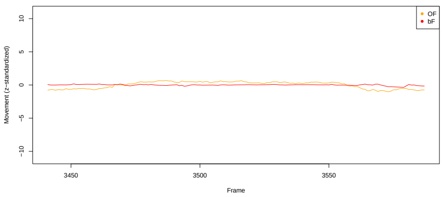

Participant 31 Anxiety, X_54, CR7_x

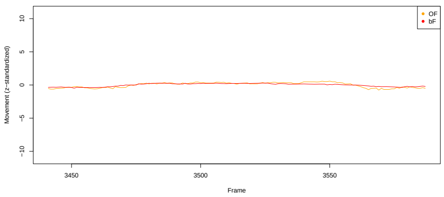

Participant 31 Anxiety, Y_48, CL7_y

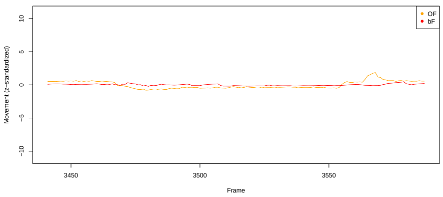

Participant 31 Anxiety, Y_54, CR7_y

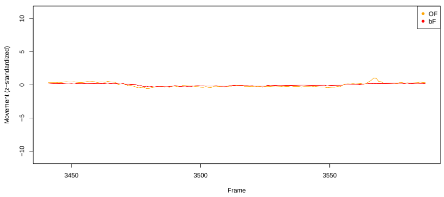

Participant 31 Anxiety, X_51, A7_x

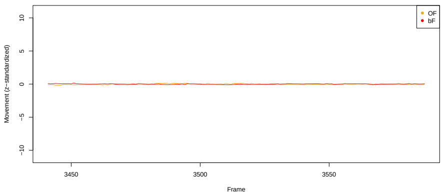

Participant 31 Anxiety, Y_51, A7_y

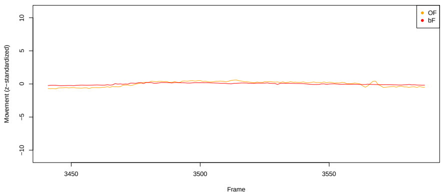

Subject 31, Disgust

Participant 31 Disgust, X_21, BL2_x

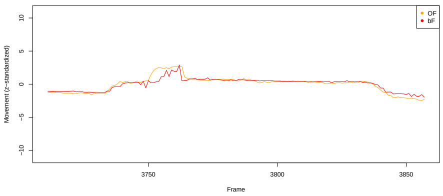

Participant 31 Disgust, X_22, BR2_x

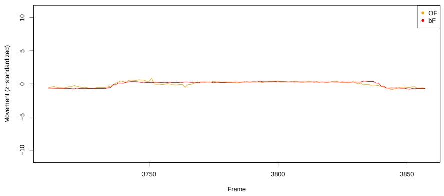

Participant 31 Disgust, Y_21, BL2_y

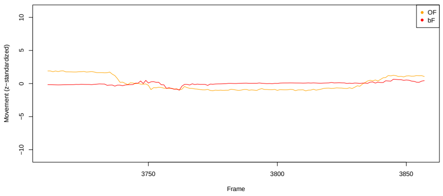

Participant 31 Disgust, Y_22, BR2_y

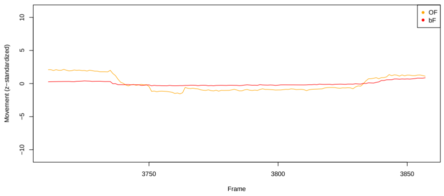

Participant 31 Disgust, X_49, BL7_x

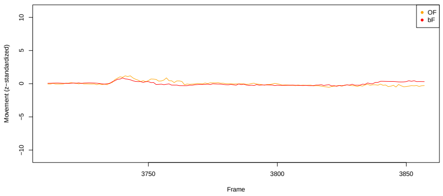

Participant 31 Disgust, X_53, BR7_x

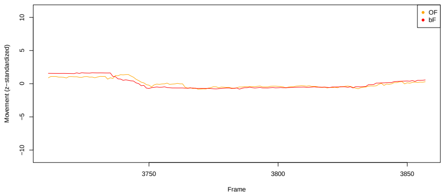

Participant 31 Disgust, Y_49, BL7_y

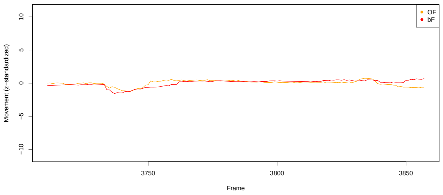

Participant 31 Disgust, Y_53, BR7_y

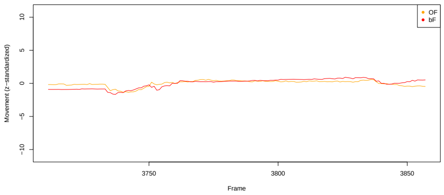

Participant 31 Disgust, X_48, CL7_x

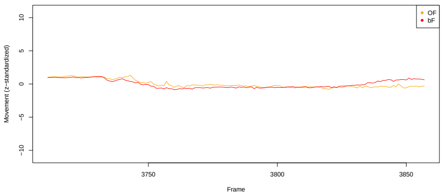

Participant 31 Disgust, X_54, CR7_x

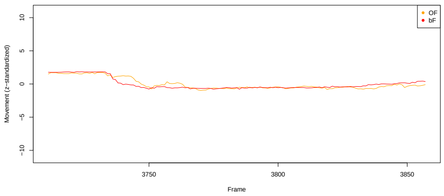

Participant 31 Disgust, Y_48, CL7_y

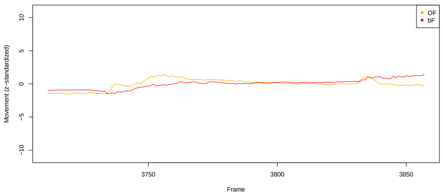

Participant 31 Disgust, Y_54, CR7_y

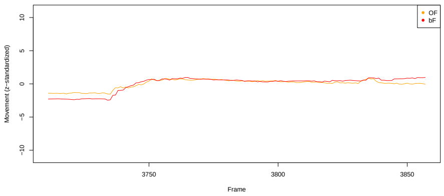

Participant 31 Disgust, X_51, A7_x

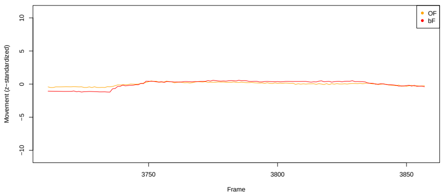

Participant 31 Disgust, Y_51, A7_y

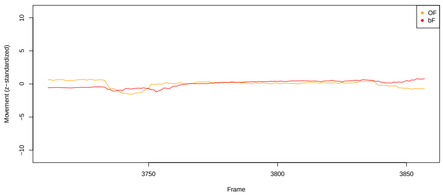

Subject 31, Happiness

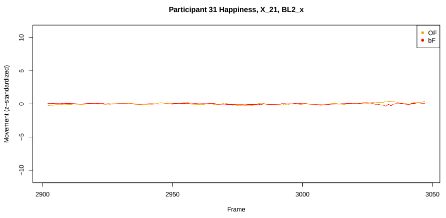

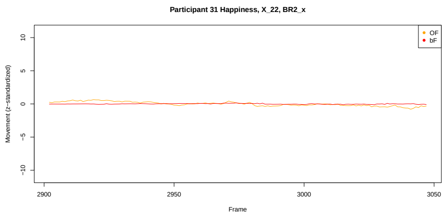

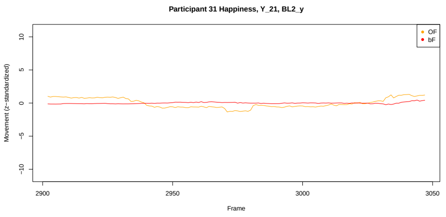

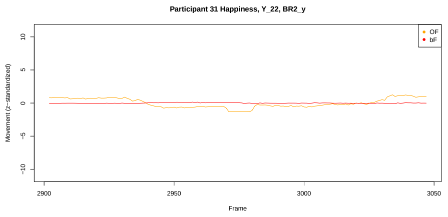

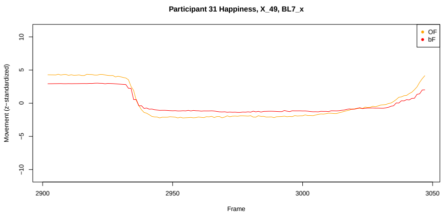

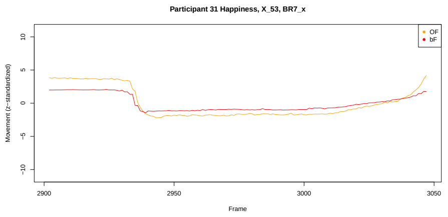

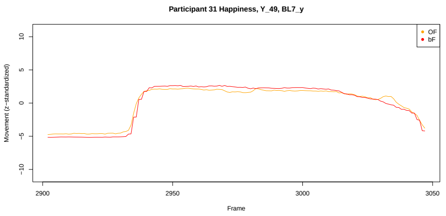

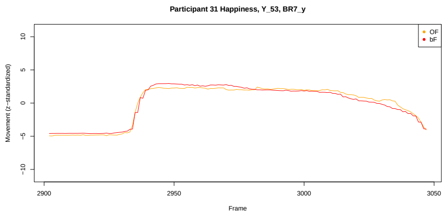

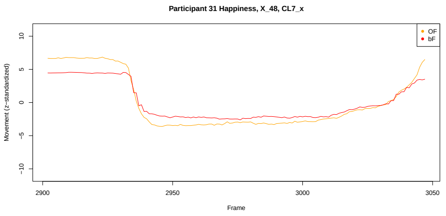

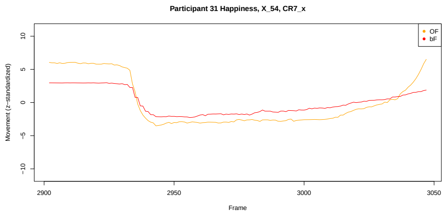

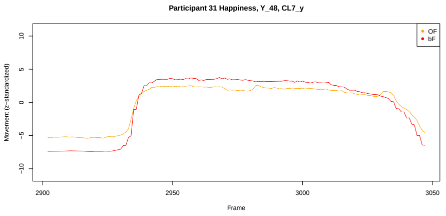

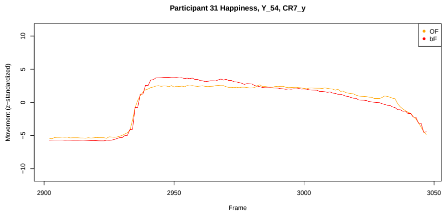

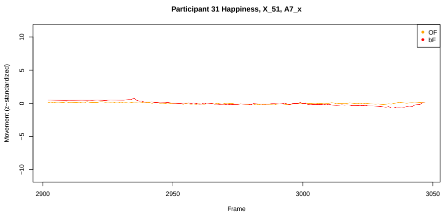

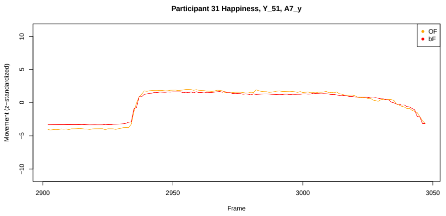

Subject 31, Sadness

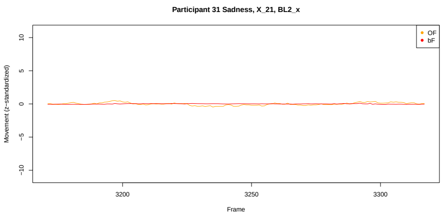

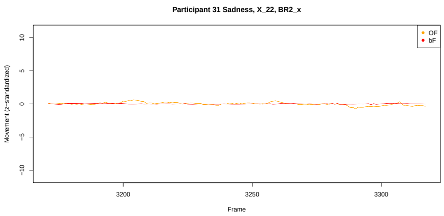

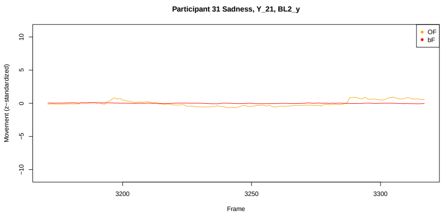

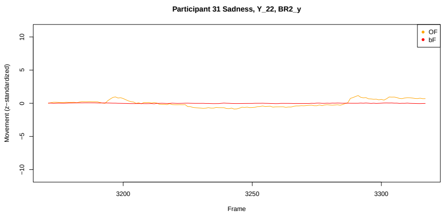

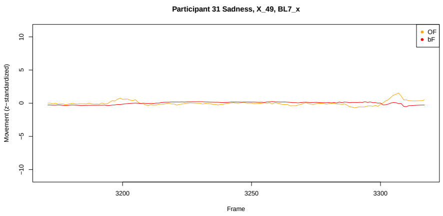

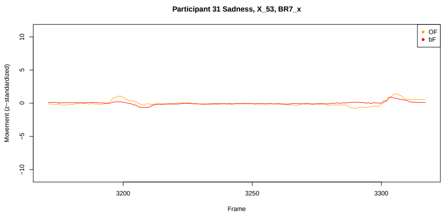

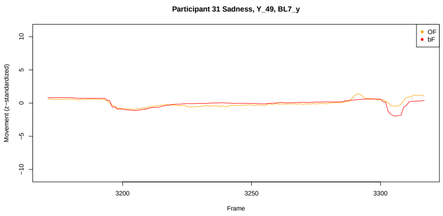

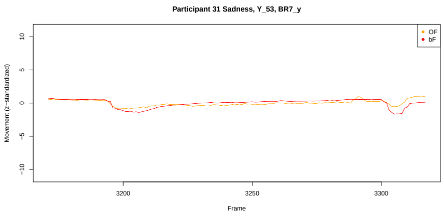

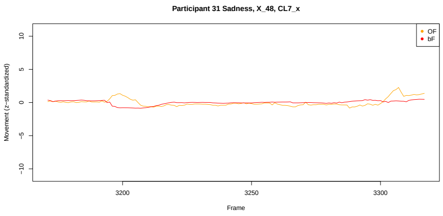

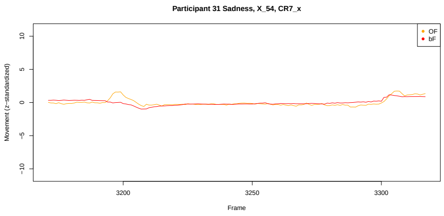

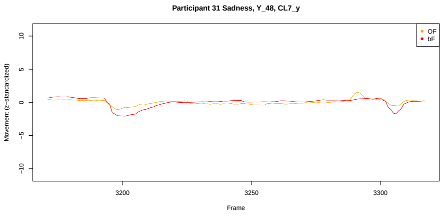

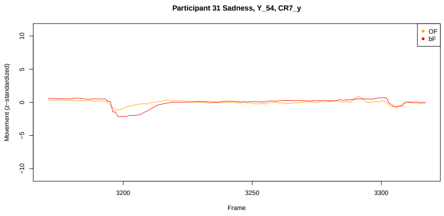

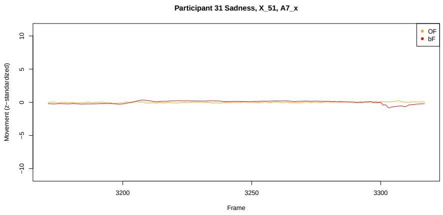

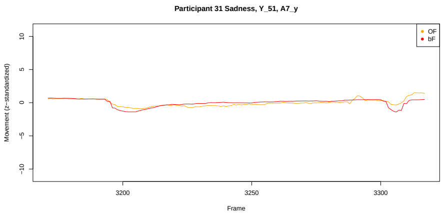

Supplement: Supplementary file 3 [file PSYP-63-e70394-s001.zip › Study 2/Plots/02_OpenFace_blenderFaceUV_Comparison/OF_bF_PlotsPerEmotionParticipant.pdf]
