# Supplementary material for: blenderFaceUV: Toward Marker‐Based Precision and Flexibility in Facial Tracking With “Invisible” Markers
Source: Psychophysiology. 2026 Sep 21;63(9):e70394. doi: 10.1111/psyp.70394 (PMC13594373; doi:10.1111/psyp.70394)
Supplement: Supplementary file 3 [file PSYP-63-e70394-s001.zip › Study 2/Plots/02_OpenFace_blenderFaceUV_Comparison/Of_bF_MouthCornerComparison.pdf]

Comparison of OpenFace (OF) and blenderFace (bF) coordinates X axis

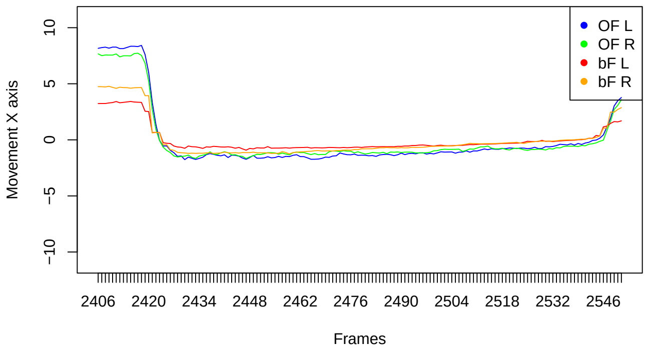

Left and right corners of the mouth posing happiness Participant 3

Comparison of OpenFace (OF) and blenderFace (bF) coordinates Y axis

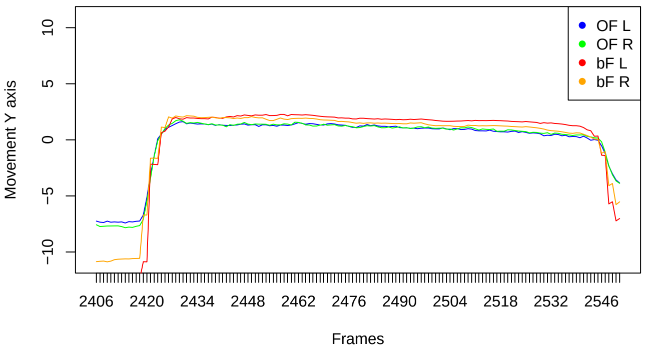

Left and right corners of the mouth posing happiness Participant 3

Comparison of OpenFace (OF) and blenderFace (bF) coordinates X axis

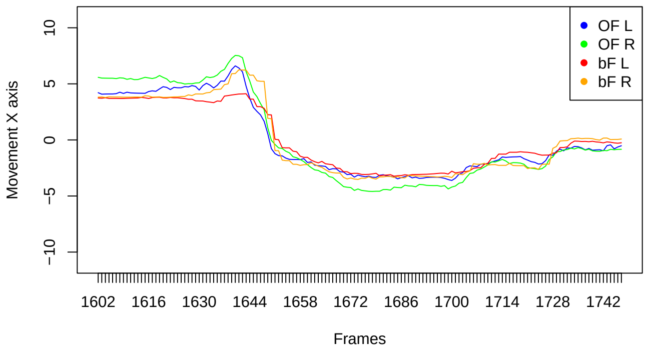

Left and right corners of the mouth posing happiness Participant 4

Comparison of OpenFace (OF) and blenderFace (bF) coordinates Y axis

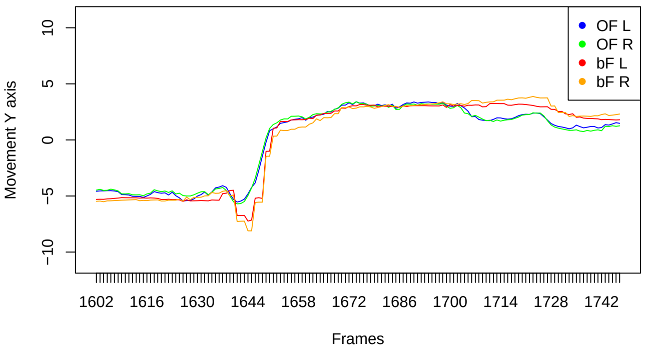

Left and right corners of the mouth posing happiness Participant 4

Comparison of OpenFace (OF) and blenderFace (bF) coordinates X axis

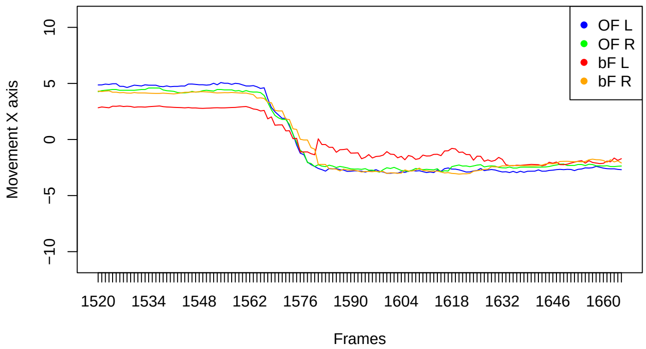

Left and right corners of the mouth posing happiness Participant 6

Comparison of OpenFace (OF) and blenderFace (bF) coordinates Y axis

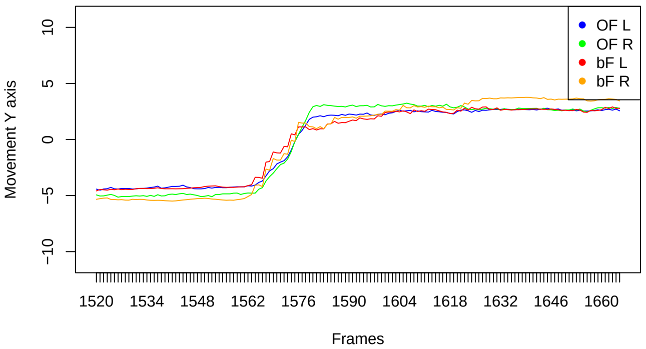

Left and right corners of the mouth posing happiness Participant 6

Comparison of OpenFace (OF) and blenderFace (bF) coordinates X axis

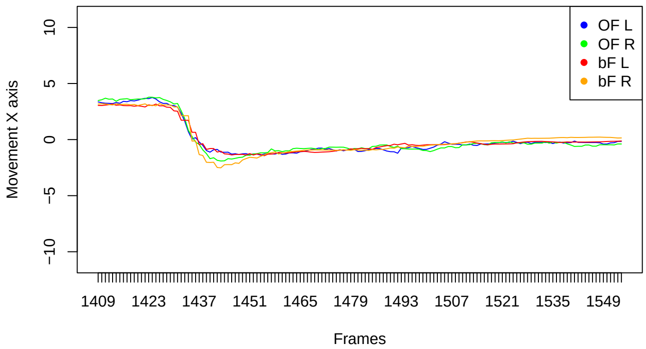

Left and right corners of the mouth posing happiness Participant 7

Comparison of OpenFace (OF) and blenderFace (bF) coordinates Y axis

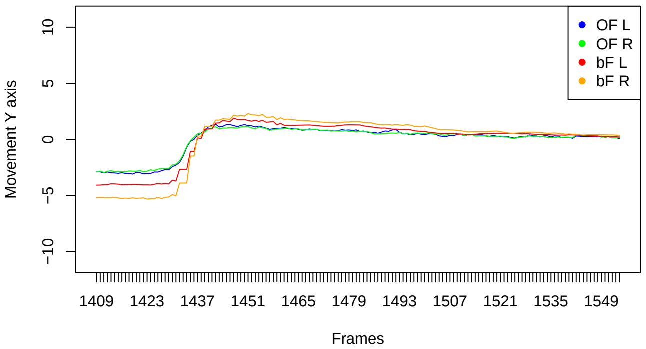

Left and right corners of the mouth posing happiness Participant 7

Comparison of OpenFace (OF) and blenderFace (bF) coordinates X axis

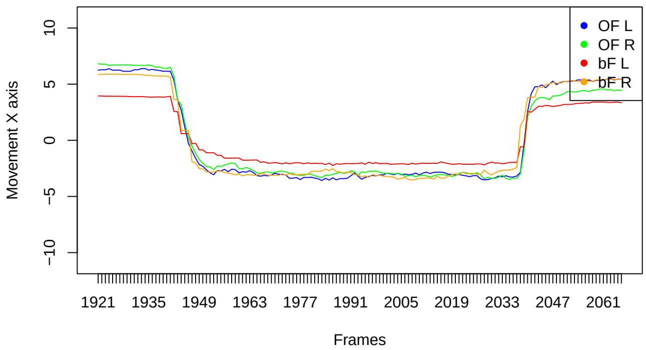

Left and right corners of the mouth posing happiness Participant 8

Comparison of OpenFace (OF) and blenderFace (bF) coordinates Y axis

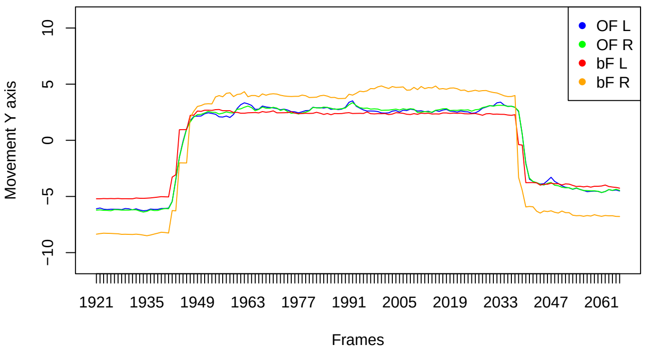

Left and right corners of the mouth posing happiness Participant 8

Comparison of OpenFace (OF) and blenderFace (bF) coordinates X axis

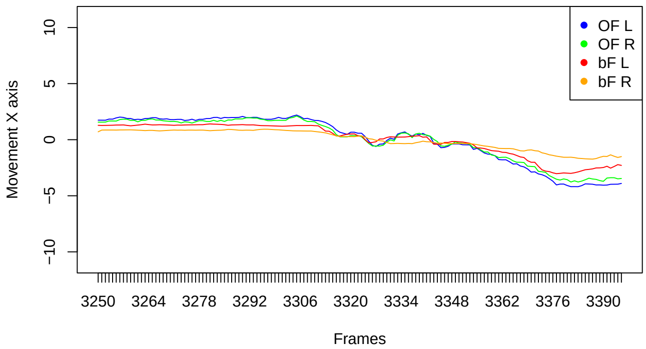

Left and right corners of the mouth posing happiness Participant 14

Comparison of OpenFace (OF) and blenderFace (bF) coordinates Y axis

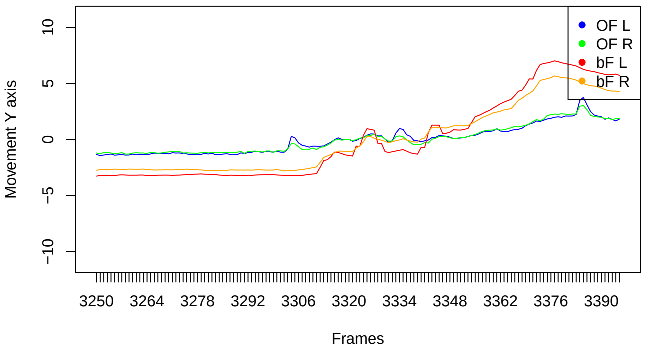

Left and right corners of the mouth posing happiness Participant 14

Comparison of OpenFace (OF) and blenderFace (bF) coordinates X axis

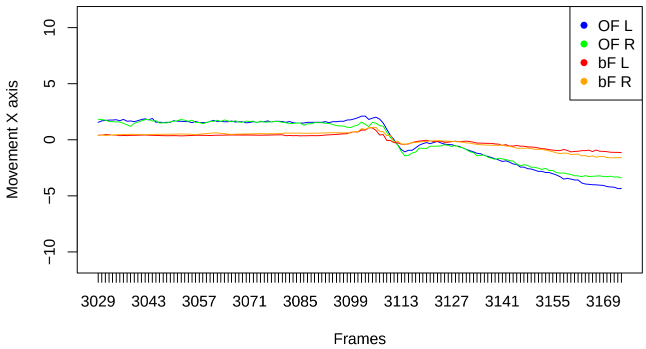

Left and right corners of the mouth posing happiness Participant 15

Comparison of OpenFace (OF) and blenderFace (bF) coordinates Y axis

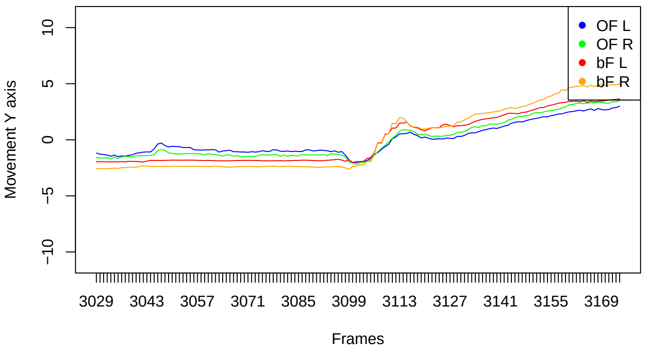

Left and right corners of the mouth posing happiness Participant 15

Comparison of OpenFace (OF) and blenderFace (bF) coordinates X axis

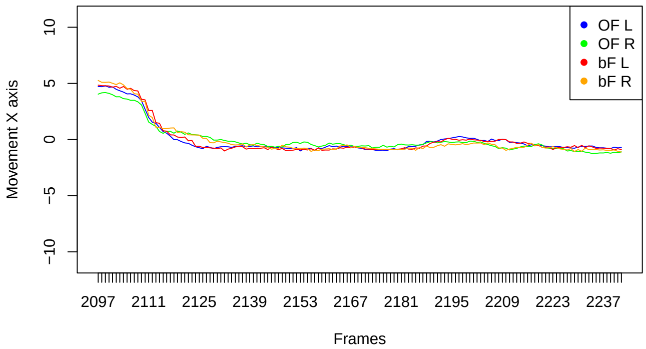

Left and right corners of the mouth posing happiness Participant 17

Comparison of OpenFace (OF) and blenderFace (bF) coordinates Y axis

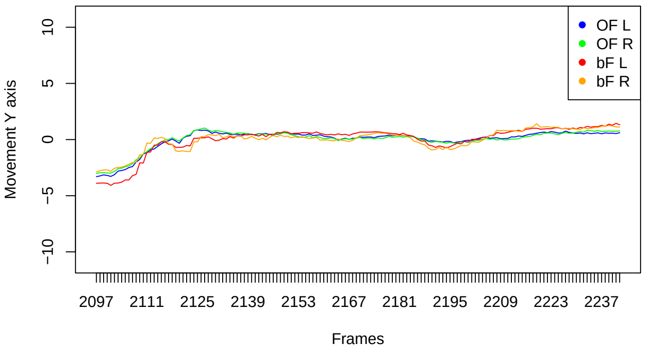

Left and right corners of the mouth posing happiness Participant 17

Comparison of OpenFace (OF) and blenderFace (bF) coordinates X axis

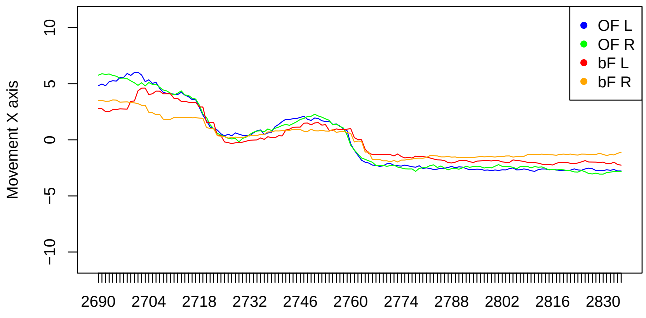

Frames
Left and right corners of the mouth posing happiness Participant 18

Comparison of OpenFace (OF) and blenderFace (bF) coordinates Y axis

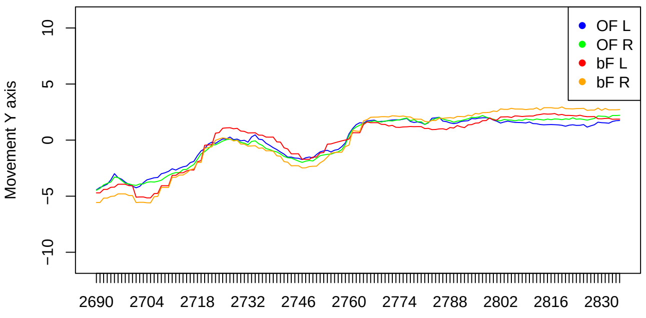

Frames
Left and right corners of the mouth posing happiness Participant 18

Comparison of OpenFace (OF) and blenderFace (bF) coordinates X axis

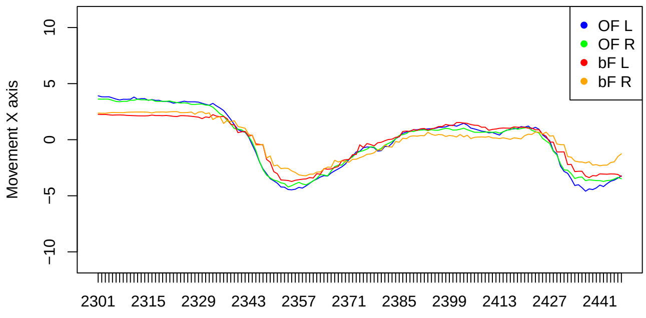

Frames
Left and right corners of the mouth posing happiness Participant 19

Comparison of OpenFace (OF) and blenderFace (bF) coordinates Y axis

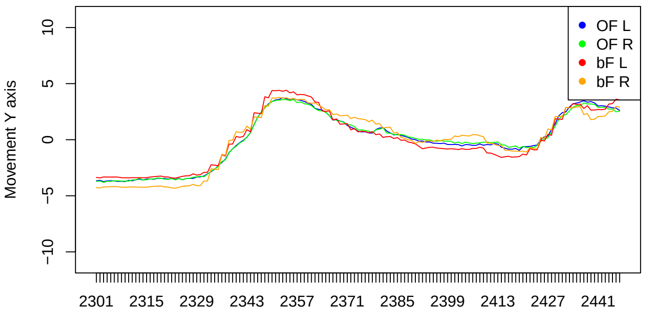

Frames
Left and right corners of the mouth posing happiness Participant 19

Comparison of OpenFace (OF) and blenderFace (bF) coordinates X axis

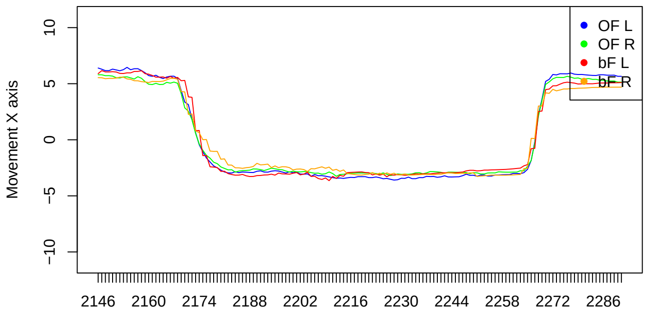

Frames
Left and right corners of the mouth posing happiness Participant 20

Comparison of OpenFace (OF) and blenderFace (bF) coordinates Y axis

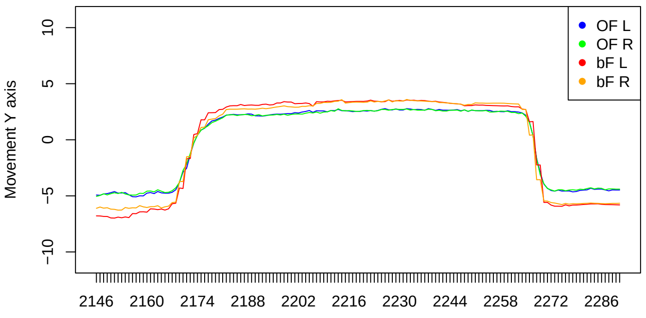

Frames
Left and right corners of the mouth posing happiness Participant 20

Comparison of OpenFace (OF) and blenderFace (bF) coordinates X axis

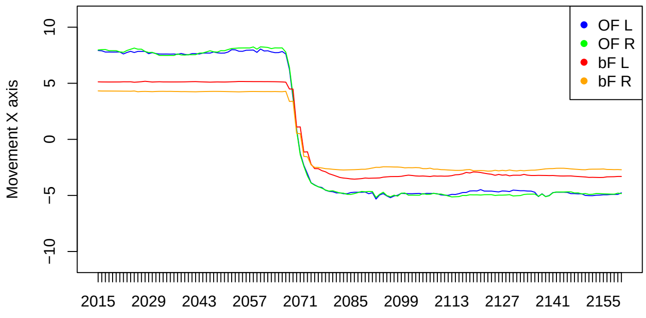

Frames
Left and right corners of the mouth posing happiness Participant 21

Comparison of OpenFace (OF) and blenderFace (bF) coordinates Y axis

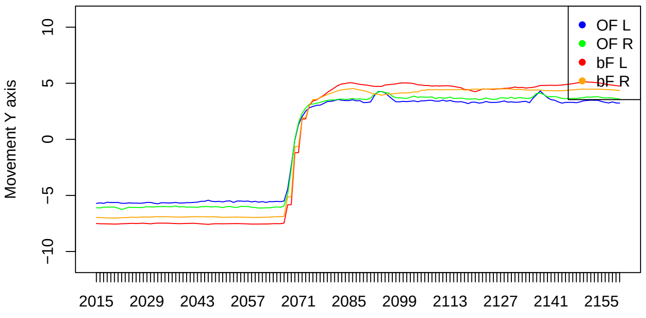

Frames
Left and right corners of the mouth posing happiness Participant 21

Comparison of OpenFace (OF) and blenderFace (bF) coordinates X axis

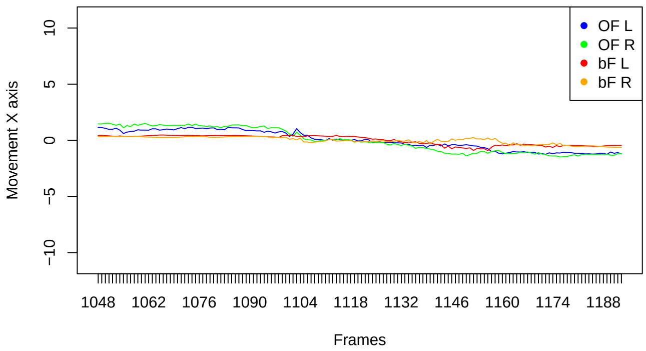

Left and right corners of the mouth posing happiness Participant 23

Comparison of OpenFace (OF) and blenderFace (bF) coordinates Y axis

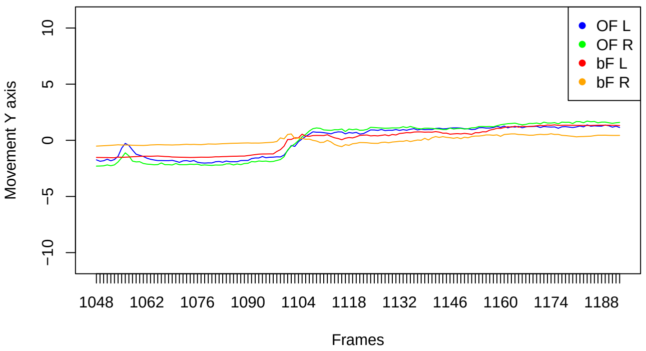

Left and right corners of the mouth posing happiness Participant 23

Comparison of OpenFace (OF) and blenderFace (bF) coordinates X axis

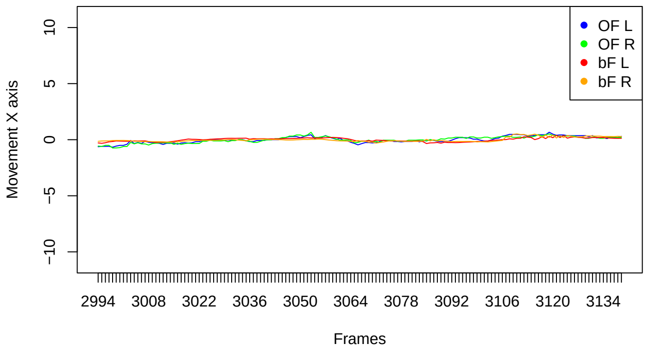

Left and right corners of the mouth posing happiness Participant 24

Comparison of OpenFace (OF) and blenderFace (bF) coordinates Y axis

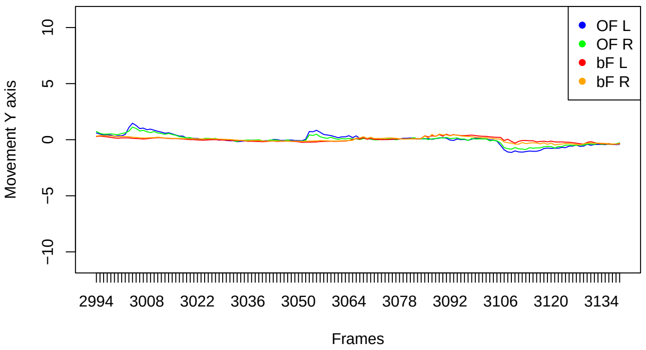

Left and right corners of the mouth posing happiness Participant 24

Comparison of OpenFace (OF) and blenderFace (bF) coordinates X axis

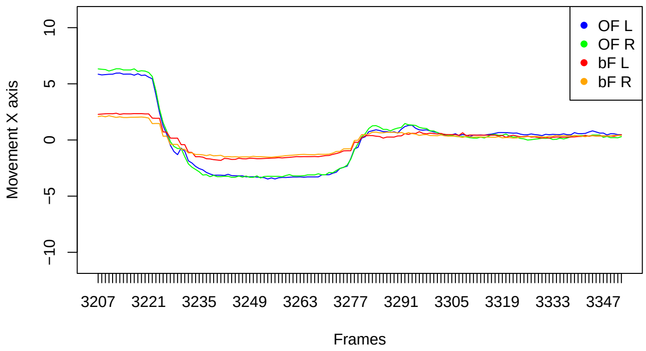

Left and right corners of the mouth posing happiness Participant 25

Comparison of OpenFace (OF) and blenderFace (bF) coordinates Y axis

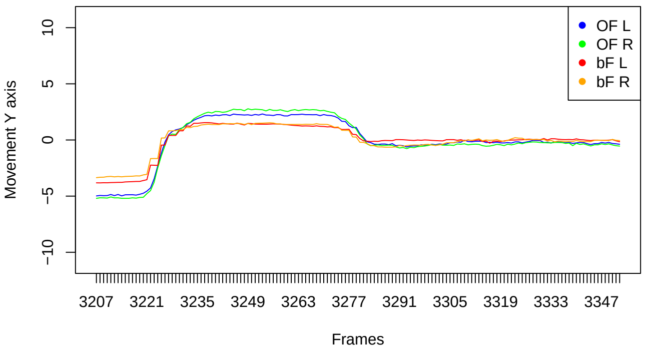

Left and right corners of the mouth posing happiness Participant 25

Comparison of OpenFace (OF) and blenderFace (bF) coordinates X axis

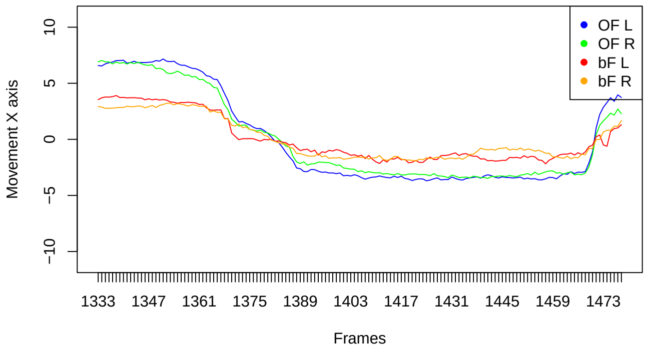

Left and right corners of the mouth posing happiness Participant 26

Comparison of OpenFace (OF) and blenderFace (bF) coordinates Y axis

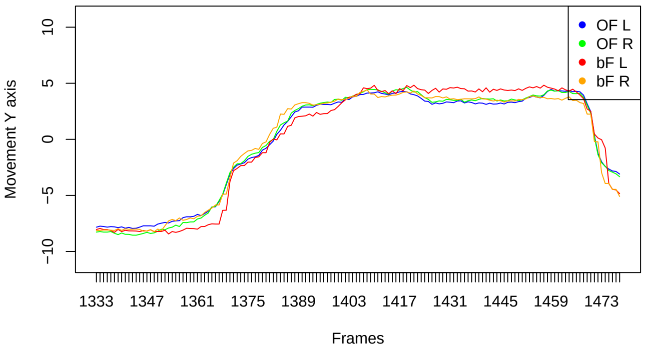

Left and right corners of the mouth posing happiness Participant 26

Comparison of OpenFace (OF) and blenderFace (bF) coordinates X axis

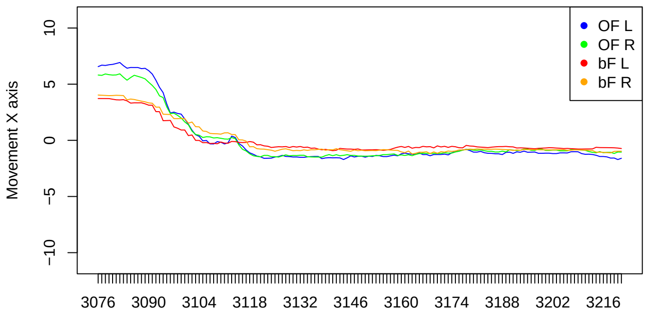

Frames
Left and right corners of the mouth posing happiness Participant 30

Comparison of OpenFace (OF) and blenderFace (bF) coordinates Y axis

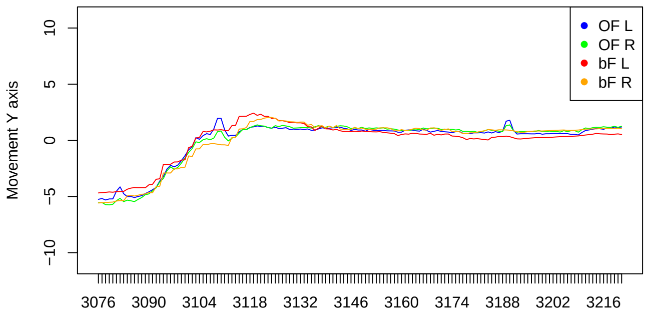

Frames
Left and right corners of the mouth posing happiness Participant 30

Comparison of OpenFace (OF) and blenderFace (bF) coordinates X axis

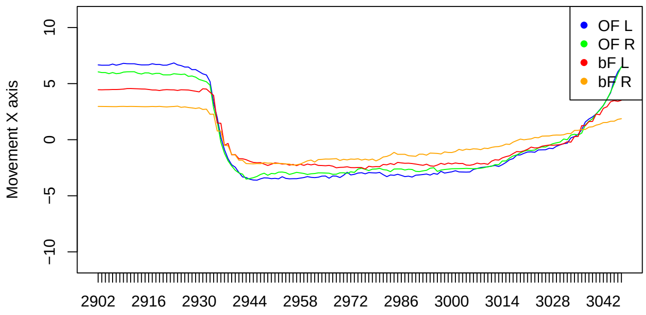

Frames
Left and right corners of the mouth posing happiness Participant 31

Comparison of OpenFace (OF) and blenderFace (bF) coordinates Y axis

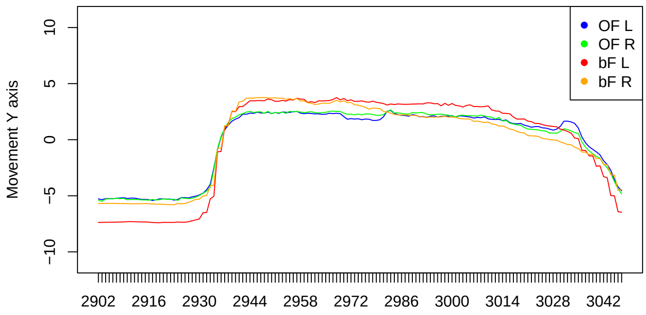

Frames
Left and right corners of the mouth posing happiness Participant 31
